# The Potential Diagnostic Value of Immune-Related Genes in Interstitial Fibrosis and Tubular Atrophy after Kidney Transplantation

**DOI:** 10.1155/2022/7212852

**Published:** 2022-06-17

**Authors:** Bin Yang, Dike Shi, Yahong Chen, Yi Zhu

**Affiliations:** ^1^Department of Hepato-Pancreato-Biliary Surgery, The Second Affiliated Hospital, Zhejiang University School of Medicine, Zhejiang, China; ^2^Department of Gastrointestinal Surgery, The Second Affiliated Hospital, Zhejiang University School of Medicine, Zhejiang, China

## Abstract

**Background:**

Inflammation within areas of interstitial fibrosis and tubular atrophy (IF/TA) is associated with kidney allograft failure. The aim of this study was to reveal new diagnostic markers of IF/TA based on bioinformatics analysis.

**Methods:**

Raw data of IF/TA samples after kidney transplantation and control samples after kidney transplantation were extracted from the Gene Expression Omnibus (GEO) database (GSE76882 and GSE120495 datasets), and genes that were differentially expressed between the two groups (DEGs) were screened. Gene Set Enrichment Analysis (GSEA), ESTIMATE and single sample GSEA (ssGSEA), least absolute shrinkage and selection operator (LASSO) regression analysis, and competing endogenous RNA (ceRNA) network were used to analyze the data.

**Results:**

The results of GSEA revealed that multiple immune-related pathways were enriched in the IF/TA group, and subsequent immune landscape analysis also showed that the IF/TA group had higher immune and stromal scores and up to 15 types of immune cells occupied them, such as B cells, cytotoxic cells, and T cells. LASSO regression analysis selected 6 (including *ANGPTL3*, *APOH*, *LTF*, *FCGR2B*, *HLA-DQA2*, and *EGF*) out of 14 DE-IRGs as diagnostic genes to construct a diagnostic model. Then, receiver operating characteristic (ROC) curve analysis showed the powerful diagnostic value of the model, and the area under the curve (AUC) of a single diagnostic gene was greater than 0.75. The results of ingenuity pathway analysis (IPA) also indicated that DEGs were involved in the immune system and kidney disease-related pathways. Finally, we found multiple miRNAs that could regulate diagnostic genes from the ceRNA network.

**Conclusion:**

This study identified 6 IF/TA-related genes, which might be used as a new diagnosis model in the clinical practice.

## 1. Introduction

Kidney transplantation is the primary treatment for patients with end-stage renal disease [1]. Compared with the dialysis therapy, it can significantly improve the life quality of patients. However, due to the shortage of organ donors, only a limited percentage of patients could receive kidney transplant [2]. In the past few decades, there have been spectacular improvements in the short-term survival of kidney grafts owing to the evolution of immunosuppressive and surgical techniques. Nevertheless, these advances have made a modest contribution to its long-term survival rate, which reflects the need to improve and maintain the long-term function of allografts [3].

Interstitial fibrosis and tubular atrophy (IF/TA), a nonspecific lesion induced by various immune and nonimmune injuries to the graft, largely limits the longevity of graft survival and function [4]. The clinical symptoms of IF/TA are not obvious, and this persistent dysfunction may cause irreversible damage to the allograft. The detection rate of IF/TA is as high as 40% in the biopsy at 3-6 months and increases to 65% at 2 years after kidney transplantation [5]. Moreover, it has also been reported that the onset of IF/TA is found within 6 months after transplantation, indicating that earlier warning methods were required for prolonging graft function [6]. Recent discovery of some biomarkers, which can be loosely grouped into those that mark tubule cell injury (e.g., kidney injury molecule 1 and monocyte chemoattractant protein 1) and those that mark tubule cell dysfunction (e.g., *α*1-microglobulin and uromodulin), can provide additional information on risk of chronic kidney disease progression, and these biomarkers provide new opportunities to monitor response to therapeutics used to treat chronic kidney disease patients, while at present, the clinically wide accepted diagnosis of IF/TA mainly depends on the biopsy results of the transplanted kidney. Herein, more accurate, noninvasive, and repeatable models for IF/TA diagnosis by biomarker panels are still needed.

In this study, we observed that immune-related pathways were significantly enriched in the IF/TA group through Gene Set Enrichment Analysis (GSEA) and used public databases to screen for immune-related differentially expressed genes related to IF/TA. Using the least absolute shrinkage and selection operator (LASSO) regression analysis and receiver operating characteristic (ROC) analysis, we obtained a highly accurate 6 gene diagnostic models (*ANGPTL3*, *APOH*, *LTF*, *FCGR2B*, *HLA-DQA2*, and *EGF*) with an area under the curve (AUC) of 0.821. Also, our study suggested that these diagnostic genes might regulate several pathways such as amino acid metabolism, inducing immune responses, and cell activation. It could be assumed that these biomarkers might be used for early diagnosis of IF/TA in the future.

## 2. Materials and Methods

### 2.1. Data Source

**Figure 1:**
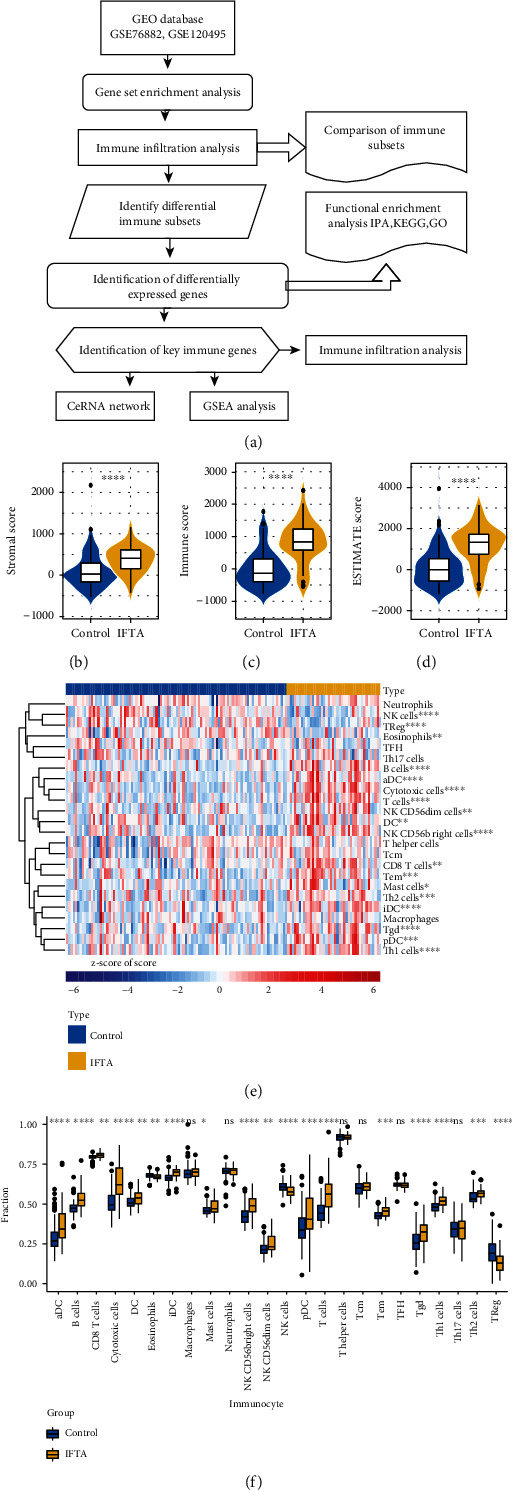
Work flow chart and immune landscape analysis of IF/TA group and control group. (a) Work flow chart of this study, drawn with online tool SangerBox (http://vip.sangerbox.com). (b–d) The immune and stromal scores were analyzed using the ESTIMATE algorithm (vs. control, *P* < 0.0001). (e) Heatmap of immune cell infiltration. Each small square represents each immune cell gene set and its color represents the size of the gene expression. The greater the expression, the deeper the color (red is high expression and blue is low expression). The first line represents sample grouping, lake blue represents control sample, and pink represents IF/TA sample. Each row represents the expression of each gene set in different samples, and each column represents the expression of all gene sets in each sample. (f) The abundance of immune cells in ssGSEA. ∗ in the figure is a significance marker, ∗ stands for *P* < 0.05, ∗∗ stands for *P* < 0.01, ∗∗∗ stands for *P* < 0.001, and ∗∗∗∗ stands for *P* < 0.0001.

The mRNA sequencing data of IF/TA samples after kidney transplantation were obtained from the Gene Expression Omnibus (GEO) database, which included GSE76882 [1] and GSE120495 [2] datasets. The GSE76882 dataset was used for the main analysis of this study, and the GSE120495 dataset was used to validate the validity of the diagnostic model and the expression patterns of the diagnostic markers. The GSE76882 dataset included samples of IF/TA with acute rejection (*n* = 29), IF/TA with inflammation (*n* = 10), IF/TA without inflammation (*n* = 42), biopsy-proven AR (*n* = 54), and normal functioning transplants (normal; *n* = 99). Here, only the 42 samples of IF/TA without inflammation and 99 samples of normal functioning transplants in this dataset were utilized in this study. For the GSE76882 dataset, which contains 25 renal allogeneic biopsies (5 with a diagnosis of acute tubular injury, 5 with T cell-mediated rejection, 5 with IF/TA, 5 with BK virus-associated nephropathy, and 5 with a functionally stable allograft recipients), five native kidney biopsies with interstitial nephritis are also included. The statistics of clinical characteristics of each data set are shown in Table 1. However, only the relevant sequencing data from the five IFTA samples and the five recipients with functionally stable allografts in this dataset were used in the validation analysis of this study. A total of 2483 IRGs were obtained from the ImmPort database (Supplementary Table 1). The GEO database is freely available to the public, and this research also strictly followed access policies and publication guidelines; therefore, this study did not require ethics review and approval by the ethics committee.  Figure 1(a) shows the workflow of this study.

### 2.2. Gene Set Enrichment Analyses

To explore the related pathways of the IF/TA group, GSEA was executed to grabble the enrichment terms related to the Kyoto Encyclopedia of Genes and Genomes (KEGG) pathway. The predefined KEGG pathway gene set (c2.cp.kegg.v7.2.symbols.gmt) was downloaded from the Molecular Signatures Database (MSigDB) (http://www.gsea-msigdb.org/gsea/msigdb/). Briefly, based on the R package GSEA (version 4.1.0), the genes between the two groups were sorted by the algorithm's default Signal2Noise, and the enrichment analysis of the sorted genes was subsequently performed on the predefined gene set. *P* < 0.05 was considered statistically significant.

### 2.3. Estimation of Immune Cell Type Infiltrations

Estimation of STromal and Immune cells in Malignant Tumor tissues using Expression (ESTIMATE) data algorithm was applied to show the presence of infiltrated immune cells and stromal cells in tumor tissues, which is denoted by immune score and stromal score, respectively [7, 8]. And the Wilcoxon test was used to compare differences between groups. We utilized ssGSEA to estimate the infiltrations of immune cell types between control and IF/TA groups. Among them, the immune cells used were derived from research published by Bindea et al. [9].

### 2.4. Identification of DEGs in IF/TA

The DEGs were calculated using the “Limma” version 3.46.0 R package in the GSE76882 and GSE120495 datasets. DEGs with an absolute log_2_ fold change (FC) > 1 and *P* < 0.05 were considered for subsequent analysis. Besides, DE-IRGs were obtained by the intersection analysis of DEGs (in GSE76882 and GSE120495 datasets) and IRGs in the ImmPort database.

### 2.5. Selection of Significant Diagnostic Features and Model Construction with the Training Set

The patients of the GSE76882 dataset were separated into the training and validation sets in a ratio of 7 : 3 using the “set.seed” package in the R. The penalized Cox regression model with LASSO penalty was used to select the most useful diagnostic markers among 14 DE-IRGs, and the optimal values of the penalty parameter *λ* were determined by tenfold cross-validations [10, 11]. After the feature selection, the diagnostic model was constructed. In addition, the Pearson correlation analysis was conducted on the expression levels of key genes.

### 2.6. Model Performance and Validation

The area under the curve (AUC) from a receiver operating characteristic curve (ROC) analysis was calculated to test the diagnostic performance of the model in the training and validation sets. Also, the diagnostic performance of each diagnostic gene was tested by ROC. R package “pROC” was used for drawing ROC curves [3].

### 2.7. Single-Gene GSEA

Similarly, c2.cp.kegg.v7.2.symbols.gmt was downloaded from MSigDB as the target set and single-gene GSEA was detected using GSVA (version 1.38.0) software [4]. In this study, we calculated the correlations of diagnostic genes with all other genes separately and ranked all genes according to their correlations from the highest to the lowest, and the ranked genes were used as the set of genes to be tested to detect the enrichment of KEGG signaling pathway. Only gene sets with *P* < 0.05 were considered as significant.

### 2.8. Ingenuity Pathway Analysis (IPA)

To highlight the underlying mechanisms regulating the observed changes in gene expression profiles, Ingenuity Pathway Analysis (IPA) (version 1-19-00, Qiagen) [5] was performed. The DEGs in the GSE76882 dataset were first uploaded into Qiagen's IPA system for core and disease and function analysis. The ingenuity knowledge base (genes only) was selected as the reference set. IPA was performed to identify the canonical and disease and function pathways associated with the common DEGs [12]. The results were expressed as *z*-score [13, 14].

### 2.9. Construction of ceRNA Network

To predict miRNAs and lncRNAs that may be related to diagnostic genes, we downloaded the transcript sequences of 6 diagnostic genes from the National Center for Biotechnology Information (NCBI). Meanwhile, we obtained human microRNA sequences from the miRbase (version 22, https://www.mirbase.org/) [6]. Then, we used miRanda tool (http://www.microrna.org/microrna/home.do) to predict the combination of diagnostic genes and microRNAs. In this case, the combined score threshold was set to 170 (the default is 140). Next, we used the starBase (version 2.0, http://starbase.sysu.edu.cn/starbase2/) [7] to predict the lncRNA that may bind to the microRNA obtained above. In this way, we had a molecular interaction network of mRNA-microRNA-lncRNA.

### 2.10. Statistical Analysis

All analyses were conducted using R software. ssGSEA and single-gene GSEA were constructed using the R package GSVA [15]. The two-tailed paired *t*-test was used for data of *LTF*, *FVGR2B*, *HLA-DQA2*, *EGF*, *ANGPTL3*, and *APOH* expressions at mRNA level. All statistical tests were two-sided and *P* < 0.05 were considered statistically significant.

## 3. Results

### 3.1. IF/TA Group Closely Related to Immune-Related Pathways

To explore the underlying pathways of the IF/TA, we conduct GSEA-KEGG comparing the control group (*n* = 99) with the IF/TA group (*n* = 42) in 141 GSE76882 samples of the whole set. In the IF/TA group, 15 immunological characteristics including “antigen processing and presentation,” “autoimmune thyroid disease,” “B cell receptor signaling pathway,” “cell adhesion molecules (CAMs),” “chemokine signaling pathway,” “cytokine-cytokine receptor interaction,” “Fc epsilon RI signaling pathway,” “Fc gamma R-mediated phagocytosis,” “JAK-STAT signaling pathway,” “MAPK signaling pathway,” “natural killer cell-mediated cytotoxicity,” “NOD-like receptor signaling pathway,” “primary immunodeficiency,” “T cell receptor signaling pathway,” and “Toll-like receptor signaling pathway” were enriched. Not surprisingly, it was also found that “allograft rejection” and “graft versus host disease” pathways were significantly enriched. Also, the “pathways in cancer” was closely related to it (Supplementary Figure 1). In the control group, the enriched Kyoto Encyclopedia of Genes and Genomes (KEGG) pathways were mainly focused on the amino acid metabolism process (including valine leucine, isoleucine, lysine, arginine, proline, and histidine). However, there was no obvious enrichment of oncology features (Supplementary Table 2).

### 3.2. Immune Landscape Analysis of IF/TA Group and Control Group

Inspired by the above results, we turned our attention to the immune microenvironment of IF/TA. The immune and stromal scores were analyzed using the Estimation of STromal and Immune cells in Malignant Tumor tissues using Expression (ESTIMATE) data algorithm. These results show that the higher immune, stromal, and ESTIMATE scores were associated with IF/TA (vs. control, Figures 1(a)–1(c), *P* < 0.0001).

We employed the ssGSEA algorithm to analyze the immune cell infiltration of each sample in the GSE76882 dataset (*n* = 141) based on the 24 immune cells. The abundance of immune cells in ssGSEA is shown in Figures 1(d) and 1(e). The results revealed that activated dendritic cells (aDC), B cells, CD8 T cells, cytotoxic cells, DC, interdigitating DC (iDC), mast cells, NK CD56bright cells, NK CD56dim cells, plasmacytoid DC (pDC), T cells, effector memory (Tem), Gamma delta T cells (Tgd), T helper1 (Th1) cells, and Th2 cells were increased in the IF/TA group and eosinophils, NK cells, and regulatory cells (TReg) were depleted in number, which implied that immune-related genes (IRGs) may play an essential role in the IF/TA population.

### 3.3. Identification of IF/TA-Related DE-IRGs

**Figure 2:**
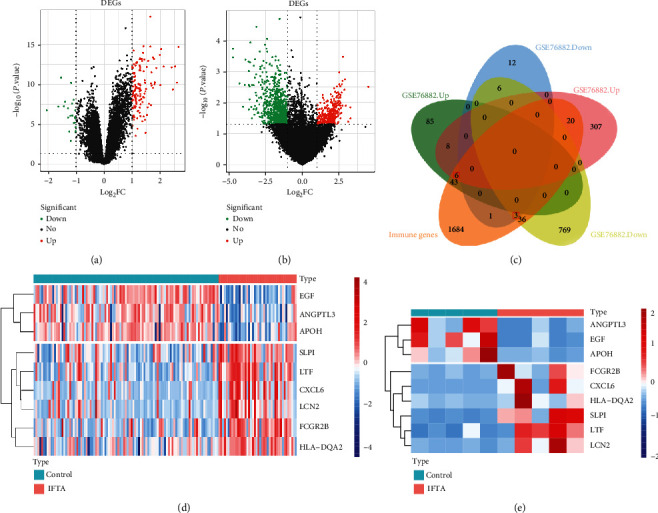
Identification of IF/TA-related DE-IRGs. (a and b) DEGs were identified from the GSE76882 and GSE120495 datasets. In the figures, each point represents a gene, and the green and red points represent the significantly differentially expressed genes, and the red points represent upregulated differentially expressed genes, while the green points represent downregulated differentially expressed genes, and the black points represent no significantly differentially expressed genes. (c) IRGs from the ImmPort database were compared to DEGs from above two datasets. The red box in the figure is the final IFTA DE-IRGs, including 6 upregulated differential immunity genes and 3 downregulated differential immunity genes. (d and e) The heatmap of *EGF*, *ANGPTL3*, *APOH FCGR2B*, *CXCL6*, *HLA-DQA2*, *SLPI*, *LTF*, and *LCN2* genes in the IF/TA group. Each small square represents each gene, and its color represents the size of the gene expression. The greater the expression, the deeper the color (red is high expression and blue is low expression). The first line represents sample grouping, lake blue represents control sample, and pink represents IF/TA sample. Each row represents the expression of each gene in different samples, and each column represents the expression of all genes in each sample.

A total of 164 and 1155 DEGs were identified from the GSE76882 and GSE120495 datasets, respectively (Figures 2(a) and 2(b)). We downloaded 2483 IRGs from the ImmPort database. By the intersection analysis, nine cogenes were found among the above genes (164 DEGs from the GSE76882 dataset, 1155 DEGs identified from the GSE120495 dataset, and 2483 IRGs), which were considered as DE-IRG (Figure 2(c)). Interestingly, the trends of these gene expressions were similar in GSE76882 and GSE120495 datasets. Among them, *EGF*, *ANGPTL3*, and *APOH* showed downregulated expression and 6 genes had upregulated expression in the IF/TA as compared to control samples as shown in the heatmap (Figures 2(d) and 2(e)).

### 3.4. Identification and Evaluation of IF/TA Diagnostic Genes

**Figure 3:**
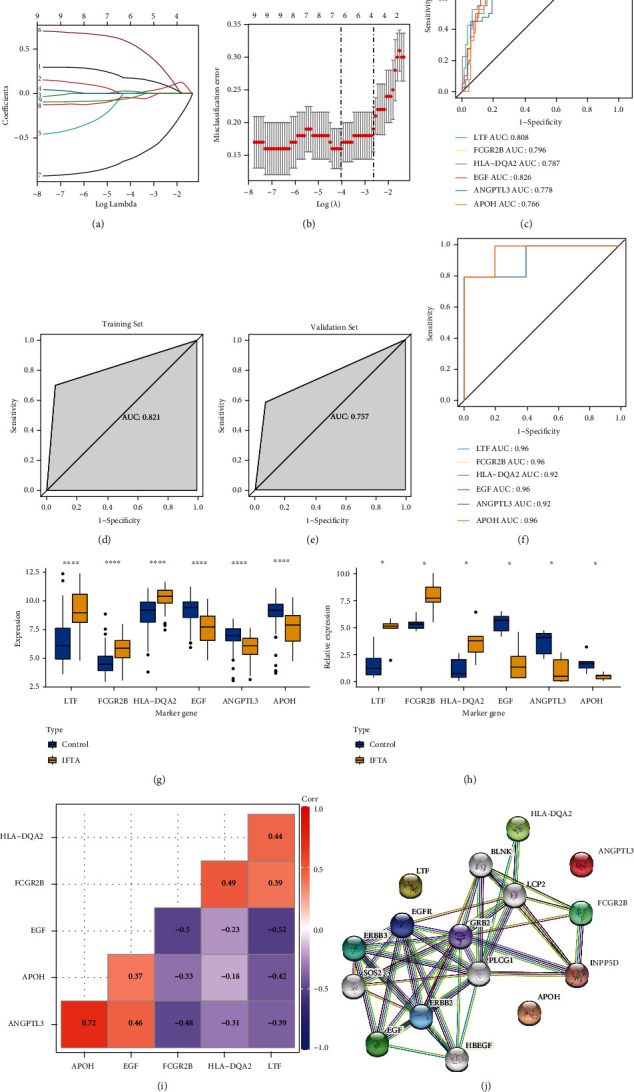
Identification and evaluation of IF/TA diagnostic genes. (a and b) The LASSO regression with 10-fold cross-validation of *LTF*, *FCGR2B*, *HLA-DQA2*, *EGF*, *ANGPTL3*, and *APOH*. (c) The AUC of diagnostic model in the training set. (d) The AUC of diagnostic model in the validation set. (e) The AUCs of all 6 diagnostic genes in the GSE76882 dataset. (f) The AUCs of all 6 diagnostic genes in the GSE120495 dataset. (g and h) The expression patterns of 6 diagnostic genes from the GSE76882 and GSE120495 datasets. ∗ in the figure is a significance marker, ∗ stands for *P* < 0.05, ∗∗ stands for *P* < 0.01, ∗∗∗ stands for *P* < 0.001, and ∗∗∗∗ stands for *P* < 0.0001. (i) Correlations between six key genes. The greater the correlation, the deeper the color (red is positive correlation and blue is negative correlation). (j) Protein interaction network of six genes.

As the diagnosis is of great importance for IF/TA patients, we further analyzed whether DE-IRGs contributed to the accurate diagnosis of IF/TA. The 141 samples in the GSE76882 data set were randomly divided into a training set and a validation set in a 7 : 3 ratio by the glmnet package of R. Based on the LASSO regression with 10-fold cross-validation, we finally determined that 6 genes (*LTF*, *FCGR2B*, *HLA-DQA2*, *EGF*, *ANGPTL3*, and *APOH*) were enrolled in the final diagnostic model for IF/TA (Figures 3(a) and 3(b)). As shown in ROC analysis, the AUCs of our model reached 0.821 and 0.757 in the training set and validation set, indicating a satisfactory accuracy of prediction (Figures 3(c) and 3(d)). Then, we checked the ability of a single gene to distinguish IF/TA from normal samples in the GSE76882 dataset. The results were proved to be satisfactory as the AUC of all diagnostic genes was greater than 0.75 (Figure 3(e)). Moreover, IF/TA and normal samples in the GSE120495 dataset were also clearly distinguishable by these genes (all AUC > 0.9; Figure 3(f)). Here, based on the previous findings (Figures 2(d) and 2(e)), we further plotted box plots designed to clearly demonstrate the expression variations of the six diagnostic genes in the GSE76882 (Figure 3(g)) and GSE120495 (Figure 3(h)) datasets. The results showed that *LTF*, *FCGR2B*, and *HLA-DQA2* were elevated in the IF/TA group of the above two datasets, while the remaining 3 diagnostic genes (including *EGF*, *ANGPTL3*, and *APOH*) were overexpressed in the control group. In addition, the Pearson correlation analysis was conducted on the expression levels of six key genes. The results showed that ANGPTL3 was significantly positively correlated with *APOH*, and genes *EGF*, *APOH*, and *ANGPYL3* were negatively correlated with *LTF*, *FCGR2B*, and *HLA-DQA2*. Moreover, *HLA-DQA2* positively correlated with *LTF* and *FCGR2B*, and gene *EGF* positively correlated with *ANGPTL3* and *APOH*, respectively (Figure 3(i)). Mapping these six genes into the string database, we can observe that there is less direct interaction between these genes (Figure 3(j)), which suggests that these genes may carry different information and play a role in their respective biological pathways.

### 3.5. Pathway Enrichment Analysis of Diagnostic Genes

To further explore the potential pathways of the six diagnostic genes, we performed a single-gene GSEA-KEGG analysis in the GSE76882 dataset.

**Figure 4:**
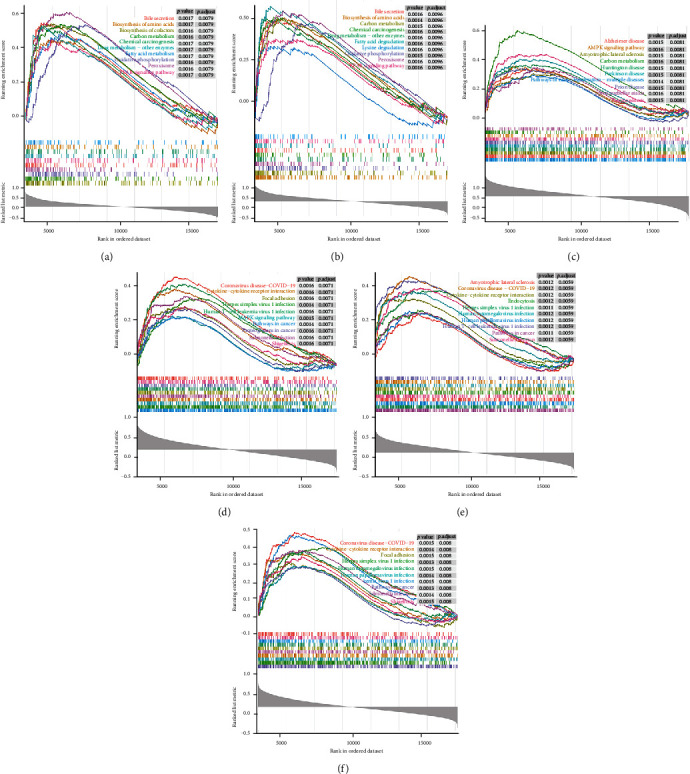
Pathway enrichment analysis of diagnostic genes. A single-gene GSEA-KEGG analysis in the GSE76882 dataset was performed to explore the potential pathways of the six diagnostic genes. (a) *ANGPTL3*, (b) *APOH*, (c) *EGF*, (d) *FCGR2B*, (e) *HLA-DQA2*, and (f) *LTF*.

For the highly expressed *ANGPTL3* in the control group, single-gene GSEA revealed a total of 163 KEGG pathways, of which 97 pathways were significantly enriched in the IF/TA samples (NES > 0, *P* < 0.05), and 66 pathways were enriched in the control group (NES < 0, *P* < 0.05). The first 10 pathways are shown in Figure 4(a).  Figure 4(b) shows the top ten pathways related to the diagnostic gene *APOH*, which was also highly expressed in the control group. A total of 101 related pathways were enriched in IF/TA samples, and 69 were enriched in normal samples. In addition, a total of 73 *EGF*-related pathways were enriched in IF/TA samples, and 81 were in normal samples (Figure 4(c)). In a comprehensive analysis, we found that the 3 diagnostic genes overexpressed in the control group were significantly related to the metabolic process of multiple amino acids (such as cysteine, methionine, valine, leucine, lysine, and arginine) in the IF/TA samples. In the control group, these genes were significantly related to the differentiation of a variety of immune cells, especially Th1, Th2, and Th17 cells. Meanwhile, we also found that *ANGPTL3*, *APOH*, and *EGF* were involved in the “allograft rejection” and “graft versus host disease” pathways in the control group (Supplementary Tables 3–5).

Figures 4(d)–4(f), respectively, show the top 10 significantly enriched pathways of the 3 diagnostic genes that were highly expressed in the IF/TA group. The results cleared that they were all involved in the “pathways in cancer,” “Herpes simplex virus 1 infection,” “cytokine-cytokine receptor interaction,” “human T cell leukemia virus 1 infection,” and “Salmonella infection” pathways. In the IF/TA group, on the other hand, *FCGR2B*, *HLA-DQA2*, and *LTF* had participated in a variety of immune-related pathways, such as “MAPK signaling pathway,” “NOD-like receptor signaling pathway,” “JAK-STAT signaling pathway,” and “PI3K-Akt signaling pathway. These genes were related to the metabolism of multiple amino acids in the control group.

### 3.6. Correlation Analysis between Diagnostic Genes and Differentially Expressed Immune Infiltrating Cells

**Figure 5:**
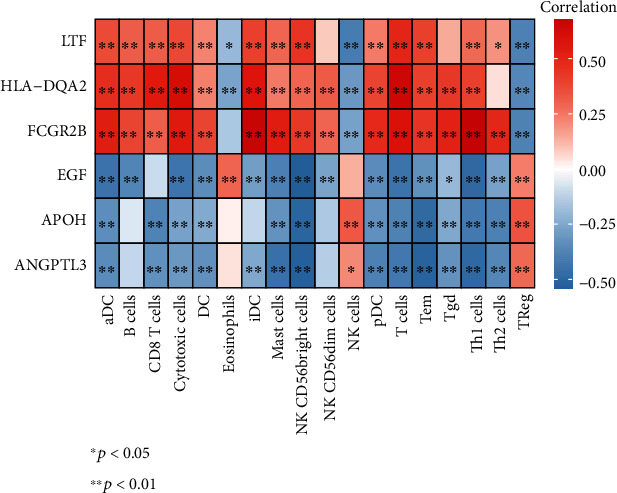
Correlation analysis between diagnostic genes and differentially expressed immune infiltrating cells. The correlations between diagnostic genes and differentially expressed immune infiltrating cells. The greater the correlation, the deeper the color (red is positive correlation and blue is negative correlation; ∗*P* < 0.05 and ^∗∗^*P* < 0.01).

Inspired by the above results, the Pearson correlation analysis showed that the diagnostic genes with increased expression in the IF/TA group had a significant positive correlation with all differentially expressed immune infiltrating cells except eosinophils, NK cells, and TReg. Conversely, compared to the control group, the downregulated diagnostic genes were significantly negatively correlated with almost all differentially expressed immune infiltrating cells (except eosinophils, NK cells, and TReg) (Figure 5). A clearer scatter plot of the relationship between diagnostic genes and differentially expressed immune infiltrating cells is shown in Supplementary Figures 2–7.

### 3.7. IPA of the IF/TA-Related DEGs

**Figure 6:**
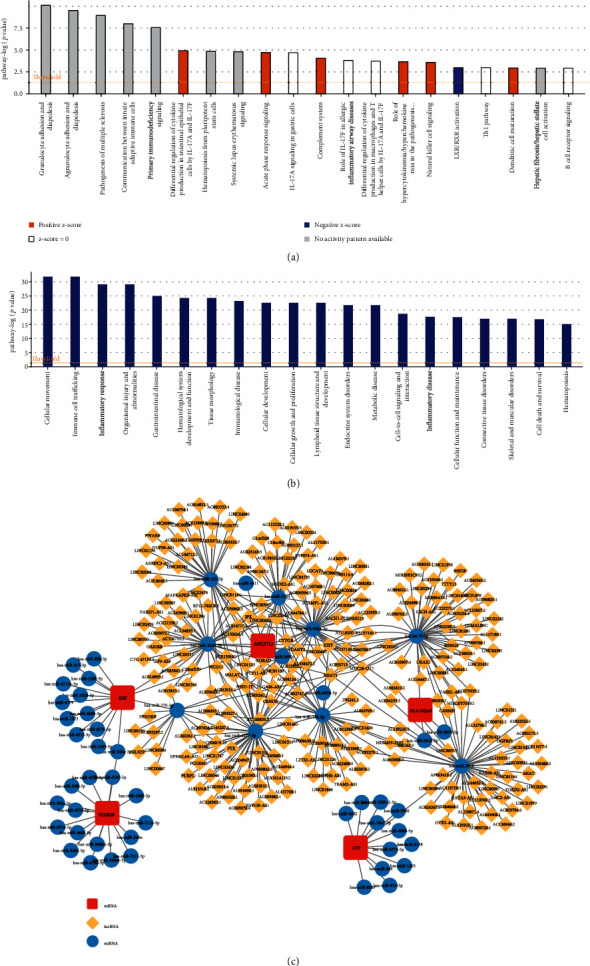
IPA of the IF/TA-related DEGs and construction of ceRNA network for diagnostic genes. (a) Canonical pathway analysis. Blue indicates that the corresponding pathway is inhibited and orange indicates activation. (b) Disease and functional pathway analysis. (c) The ceRNA network with 310 nodes and 392 edges were reconstructed to further reveal the possible role network of diagnostic genes. (red square, mRNA; yellow diamond, lncRNA; and blue circles, miRNA).

To fully understand the potential functions of diagnostic genes, we further performed IPA on IF/TA-related DEGs in the GSE76882 dataset. Canonical pathway analysis showed that these DEGs were involved in the activation and inhibition of many immune-related pathways (Figure 6(a)), such as “differential regulation of cytokine production in intestinal epithelial cells by IL-17A and IL-17F” (*z* − score = 2), “acute phase response signaling” (*z* − score = 2.449), “complement system” (*z* − score = 1), “role of hypercytokinemia/hyperchemokinemia in the pathogenesis of influenza” (*z* − score = 2.236), “natural killer cell signaling” (*z* − score = 2.449), “LXR/RXR activation” (*z* − score = −1.342), and “dendritic cell maturation” (*z* − score = 2). We found that *APOH* was involved in the regulation of “LXR/RXR activation” and “cute phase response signaling.” *HLA-DQA2* played a key role in “MSP-RON signaling in macrophage pathway” and “systemic lupus erythematosus in T cell signaling pathway.” Furthermore, *EGF* was also related to many pathways, such as “breast cancer regulation by Stathmin1,” “HER-2 signaling in breast cancer,” and “CREB signaling in neurons” (Supplementary Table 62). Moreover, disease and functional pathway analysis showed that IF/TA-related DEGs were closely related to “cellular movement,” “immune cell trafficking,” “inflammatory response,” and “organic injury and abnormalities (Figure 6(b)). Interestingly, they also played an indispensable role in “renal and urological disease” and “renal and urological system development and function” (Supplementary Table 7).

Additionally, we used IPA to predict the interaction network of diagnostic genes (*EGF* and *LTF* (Supplementary Figure 8A), *ANGPTL3* (Supplementary Figure 8B), *FCGR2B* and *APOH* (Supplementary Figure 8C), and *HLA-DQA2* (Supplementary Figure 8D)) and other DEGs. To comprehensively analyze the interaction of diagnostic genes, we merged the above four independent networks (Supplementary Figure 8E). The results showed that the relationship between these diagnostic genes was intricated, and they could form an interaction network through directed or undirected interaction with other DEGs, which was worthy of further study.

### 3.8. Construction of ceRNA Network for Diagnostic Genes

In addition, to further reveal the possible role network of diagnostic genes, we had also reconstructed the ceRNA network. Through prediction, we finally obtained a ceRNA network with 310 nodes and 392 edges. The result showed that *has-let-7f-5p*, *hsa-miR-155-5p*, *has-miR-770-5p*, *has-miR-3164*, *has-miR-1277-5p*, has-miR-136-5p, *hsa-miR-3145-3p*, *has-miR-642a-3p*, and *has-miR-3918* were linked to multiple diagnostic genes and lncRNAs and may play an important role in IF/TA. Furthermore, we also found that *hsa-miR-548c-3p* could simultaneously regulate *EGF* and *FCGR2B* (Figure 6(c)). Unfortunately, *APOH* did not appear in the ceRNA network, and it may have a unique network of relationships, which needed to be further explored.

## 4. Discussion

IF/TA occurs in the early stage after kidney transplantation, gradually induces chronic fibrosis of the transplanted kidney, and ultimately causes renal failure [4]. Although there have been many studies on the molecular mechanism involved in this pathophysiological process, the detection method of the process is rare [16]. Meanwhile, a biopsy of the transplanted kidney, a relatively reliable detection method, obviously does not suitable for multiple tests. Therefore, there is an urgent need for diagnostic molecule markers.

As widely accepted, the occurrence of IF/TA is closely related to inflammation, and the inflammation in fibrosis areas (i)-IF/TA score which derived from the BANFF score is used to evaluate the degree of fibrosis after kidney transplantation [17]. Multiple inflammation interaction and related pathways consequently induced fibroblasts infiltration, which promotes the formation of extracellular matrix and irreversible fibrosis and ultimately leads to the loss of renal function [18]. In the initial stage of the profibrotic process, the intragraft inflammation is activated and several proinflammatory and profibrotic cytokines and adhesion molecules are secreted by tubular cells with the recruitment of inflammatory infiltrate (lymphocytes, macrophages, and neutrophils) that activate peritubular capillary endothelial cells and facilitate the recruitment of new interstitial mononuclear cells [19]. Besides, ischemia/reperfusion injury during transplantation contributes to inflammation and fibrosis through reactive oxygen species (ROS) production, mitochondrial dysfunction, and activation of heparanase that induces epithelial to mesenchymal transition [20]. Our results of ssGSEA analysis presented that there were 24 types of immune infiltrating cells increased in IF/TA samples including macrophages and myofibroblasts, except eosinophils, NK cells, and Treg cells. Macrophages are a major source of TGF-*β*1, which is significantly higher in IF/TA tissue and induces myofibroblast differentiation and the production of extracellular matrix (ECM) proteins [21, 22]. Previous study reported that insufficient degradation of ECM production-deposition could change the balance in the direction of IF/TA [23, 24]. This was consistent with our ssGSEA analysis, in which macrophages did infiltrate a lot in the IF/TA group, accompanied by the release of a variety of cytokines. Consequently, these cytokines (such as MIP-1, MIP-2, and MCP-1) can promote the transformation of mesenchymal fibroblasts and tubular epithelial cells into myofibroblasts [25]. Meanwhile, the inactivation of matrix protein degrading enzymes causes the enhanced activity of protease inhibitors and induces tubular interstitial fibrosis that occurs finally [26].

For early diagnosis and detection of IF/TA, we found that *EGF*, *LTF*, *ANGPTL3*, *FCGR2B*, *ApoH*, and *HLA-DQA2* genes might be worthy of further investigation. They were closely related to immune cells other than eosinophils, NK cells, and Treg cells, and complete consistency in each immune cell whatever was high or low expression. Among them, we noticed that the expression of ANGPTL3 and ApoH genes were both reduced in IF/TA group. ANGPTLs are a family of secreted glycoproteins expressed in the liver that share common domain characteristics with angiopoietins [27]. ANGPTL3, as one of the ANGPTLs family, has a proinflammatory, proangiogenic effect and a negative effect on cholesterol efflux, implying additional proatherosclerotic properties [28]. Genetic and clinical studies have demonstrated that loss-of-function variants in ANGPTL3 are associated with decreased plasma levels of triglycerides (TGs), low-density lipoprotein cholesterol (LDL-C), and high-density lipoprotein cholesterol (HDL-C) [29]. Moreover, researchers report that ANGPTL3 is downregulated in IF/TA [23]. On the other hand, ApoH is a multifunctional plasma glycoprotein that has been associated with negative health outcomes. ApoH regulation may contribute to chronic inflammatory disease, diabetes type 2, and age-related cognitive performance [30]. In our study, through the enrichment signaling pathway analysis of diagnostic genes, we found that ANGPTL3 and ApoH both exist in the pathways related to fatty acid degradation, and both low expressed in the IF/TA group with negative correlation. Fatty acid metabolism is reported to be altered with the downregulation of enzymes and regulators of fatty acid oxidation which is mediated by the reduced activity of peroxisome proliferator-activated receptor-*α* (PPAR*α*) and peroxisome proliferator-activated receptor-gamma coactivator-1*α* (PGC1*α*) [31]. Consistently, in patients with hyperlipidemia, the expression of ANGPTL3, APOA1, TG, and LDL is increased, while PPAR*α* is decreased [32]. Our result presented the same trend as ANGPTL3 and ApoH genes were both reduced in IF/TA group, and we observed that those genes were both related to allograft rejection and graft-versus-host disease. Based on these, we suggested that the two genes might have a synergistic protective effect through PPAR*α* regulation in the fatty acid metabolism pathway in IF/TA group, and the combined analysis of them might improve the diagnostic specificity. Moreover, FCGR2B, HLA-DQA2, and LTF are involved in tumor or organ transplantation [33–35]. It is reported that HLA-DQA2 is a HLA class II molecule expressed in the surface of antigen-presenting cells and involved in the recognition of peptide antigens by CD41 T cells [36]. Our results presented that FCGR2B, HLA-DQA2, and LTF were highly expressed in the IF/TA group and involved in related pathways such as APK, NOD-LIKE, SAK-STAT, and PI3K-AKT. These classical pathways have been validated to affect the kidney function after transplant. For instance, carbamylated erythropoietin can promote long-term kidney allograft survival through activation of PI3K/AKT signaling [37].

Nowadays, the importance of miRNA has been stressed in the development of all kinds of diseases including kidney transplantation. We predicted the ceRNA network based on above 6 diagnostic genes and presented that has-let-7f-5p, hsa-mir-155-5p, has-mir-770-5p, has-mir-3164, has-mir-1277-5p, has-mir-136-5p, hsa-mir-3145-3p, has-mir-642a-3p, and has-mir-3918 might be the core microRNAs. Among them, urinary hsa-mir-155-5p has been found as a prognostic and predictive biomarker of rejection, graft outcome, and treatment response in kidney transplantation [38]. hsa-mir-155-5p can also regulate the pathogenesis of renal fibrosis via targeting SOCS1 and SOCS6 [39]. Moreover, has-mir-136-5p can improve renal fibrosis by targeting SYK and inhibition of TGF-*β*1/Smad3 signaling pathway [40]. has-mir-770-5p can cause podocyte injury via targeting E2F3 in diabetic nephropathy [41]. However, the relationship between other microRNAs and renal dysfunction needs further investigations.

Unfortunately, the limitations of this study cannot be ignored. We regretted that we were unable to obtain a sufficient quantity of clinical samples of IF/TA within a tight timeframe, and therefore, in vitro experimental validation could not be carried out in the present. Certainly, we will spare no effort to further reveal the mystery behind IF/TA after kidney transplantation.

## 5. Conclusion

In summary, this study focused on bioinformatics analysis of the DE-IRGs in IF/TA after renal transplantation. Six diagnostic genes (ANGPTL3, APOH, EGF, FCGR2B, HLA-DQA2, and LTF) were confirmed as the possible candidates for future applications.

## Figures and Tables

**Table 1:** The clinical characteristics of patients in the GSE120459 and GSE76882 datasets.

Datasets	Characteristics	No.
GSE120459	STA (stable)	5
	ATI (acute tubular injury)	5
	TCMR (T cell-mediated rejection)	5
	IFTA	5
	BKVN (BK-virus nephropathy)	5
	ISN	5
GSE76882	ANDR (andrographolide)	40
	AR (acute rejection)	54
	IFTA	42
	IFTA_AR	29
	IFTA_i (with inflammation)	10
	TX (transplants)	99

## Data Availability

The GSE76882 (https://www.ncbi.nlm.nih.gov/geo/query/acc.cgi?acc=GSE76882) and GSE120495 (https://www.ncbi.nlm.nih.gov/geo/query/acc.cgi?acc=GSE120495) datasets used for the current study period were available in the GEO repository. The data analyzed during the current study were available from the corresponding authors upon reasonable request.

## Supplementary Materials

Supplementary Materials Supplementary Figure 1: GSEA enrichment analysis of the IF/TA group. Supplementary Figure 2: correlation analysis between ANGPTL3 and differentially expressed immune infiltrating cells. Supplementary Figure 3: correlation analysis between APOH and differentially expressed immune infiltrating cells. Supplementary Figure 4: correlation analysis between EGF and differentially expressed immune infiltrating cells. Supplementary Figure 5: correlation analysis between FCGR2B and differentially expressed immune infiltrating cells. Supplementary Figure 6: correlation analysis between HLA-DQA2 and differentially expressed immune infiltrating cells. Supplementary Figure 7: correlation analysis between LTF and differentially expressed immune infiltrating cells. Supplementary Figure 8: IPA analysis shows the interaction network of diagnostic genes: EGF and LTF (8A), ANGPTL3 (8B), FCGR2B and APOH (8C), and HLA-DQA2 (8D). Merged the above four independent networks to comprehensively analyze the interaction of diagnostic genes (8E). Supplementary Table 1: immune-related genes. Supplementary Table 2: KEGG pathway in normal group. Supplementary Table 3: pathway of ANGPTL3 gene. Supplementary Table 4: pathway of APOH gene. Supplementary Table 5: pathway of EGF gene. Supplementary Table 6: ingenuity canonical pathways. Supplementary Table 7: category.

## References

[1] Pesavento T. E. (2009). Kidney transplantation in the context of renal replacement therapy. *Clinical Journal of the American Society of Nephrology*.

[2] Wijkstrom M., Iwase H., Paris W., Hara H., Ezzelarab M., Cooper D. K. (2017). Renal xenotransplantation: experimental progress and clinical prospects. *Kidney International*.

[3] Banas B., Kramer B. K., Kruger B., Kamar N., Undre N. (2020). Long-term kidney transplant outcomes: role of prolonged-release tacrolimus. *Transplantation Proceedings*.

[4] Brouard S., Renaudin K., Soulillou J. P. (2011). Revisiting the natural history of IF/TA in renal transplantation. *American Journal of Transplantation*.

[5] Granata S., Benedetti C., Gambaro G., Zaza G. (2020). Kidney allograft fibrosis: what we learned from latest translational research studies. *Journal of Nephrology*.

[6] Boor P., Floege J. (2015). Renal allograft fibrosis: biology and therapeutic targets. *American Journal of Transplantation*.

[7] Yoshihara K., Shahmoradgoli M., Martinez E. (2013). Inferring tumour purity and stromal and immune cell admixture from expression data. *Nature Communications*.

[8] Ge P. L., Li S. F., Wang W. W. (2020). Prognostic values of immune scores and immune microenvironment-related genes for hepatocellular carcinoma. *Aging (Albany NY)*.

[9] Bindea G., Mlecnik B., Tosolini M. (2013). Spatiotemporal dynamics of intratumoral immune cells reveal the immune landscape in human cancer. *Immunity*.

[10] Goeman J. J. (2010). L1 penalized estimation in the Cox proportional hazards model. *Biometrical Journal*.

[11] Zeng D., Zhou R., Yu Y. (2018). Gene expression profiles for a prognostic immunoscore in gastric cancer. *The British Journal of Surgery*.

[12] Zhao X., Sun S., Zeng X., Cui L. (2018). Expression profiles analysis identifies a novel three-mRNA signature to predict overall survival in oral squamous cell carcinoma. *American Journal of Cancer Research*.

[13] Orlando N., Babini G., Chiusolo P., Valentini C. G., De Stefano V., Teofili L. (2020). Pre-exposure to defibrotide prevents endothelial cell activation by lipopolysaccharide: an ingenuity pathway analysis. *Frontiers in Immunology*.

[14] Kramer A., Green J., Pollard J., Tugendreich S. (2014). Causal analysis approaches in Ingenuity Pathway Analysis. *Bioinformatics*.

[15] Hanzelmann S., Castelo R., Guinney J. (2013). GSVA: gene set variation analysis for microarray and RNA-seq data. *BMC Bioinformatics*.

[16] Miettinen J., Helin H., Pakarinen M., Jalanko H., Lauronen J. (2014). Histopathology and biomarkers in prediction of renal function in children after kidney transplantation. *Transplant Immunology*.

[17] Hara S. (2018). Cell mediated rejection revisited: past, current, and future directions. *Nephrology*.

[18] Rodder S., Scherer A., Raulf F. (2009). Renal allografts with IF/TA display distinct expression profiles of metzincins and related genes. *American Journal of Transplantation*.

[19] Solhjou Z., Athar H., Xu Q., Abdi R. (2015). Emerging therapies targeting intra-organ inflammation in transplantation. *American Journal of Transplantation*.

[20] Smith S. F., Hosgood S. A., Nicholson M. L. (2019). Ischemia-reperfusion injury in renal transplantation: 3 key signaling pathways in tubular epithelial cells. *Kidney International*.

[21] Nogare A. L., Dalpiaz T., Pedroso J. A. (2013). Expression of fibrosis-related genes in human renal allografts with interstitial fibrosis and tubular atrophy. *Journal of Nephrology*.

[22] Wang S., Meng X. M., Ng Y. Y. (2016). TGF-*β*/Smad3 signalling regulates the transition of bone marrow-derived macrophages into myofibroblasts during tissue fibrosis. *Oncotarget*.

[23] Maluf D. G., Mas V. R., Archer K. J. (2008). Molecular pathways involved in loss of kidney graft function with tubular atrophy and interstitial fibrosis. *Molecular Medicine*.

[24] Mengel M., Bock O., Priess M., Haller H., Kreipe H., Gwinner W. (2008). Expression of pro- and antifibrotic genes in protocol biopsies from renal allografts with interstitial fibrosis and tubular atrophy. *Clinical Nephrology*.

[25] Xu L., Sharkey D., Cantley L. G. (2019). Tubular GM-CSF promotes late MCP-1/CCR2-mediated fibrosis and inflammation after ischemia/reperfusion injury. *Journal of the American Society of Nephrology*.

[26] Clotet-Freixas S., McEvoy C. M., Batruch I. (2020). Extracellular matrix injury of kidney allografts in antibody-mediated rejection: a proteomics study. *Journal of the American Society of Nephrology*.

[27] Zheng J., Umikawa M., Cui C. (2012). Inhibitory receptors bind ANGPTLs and support blood stem cells and leukaemia development. *Nature*.

[28] Jiang S., Qiu G. H., Zhu N., Hu Z. Y., Liao D. F., Qin L. (2019). ANGPTL3: a novel biomarker and promising therapeutic target. *Journal of Drug Targeting*.

[29] Lupo M. G., Ferri N. (2018). Angiopoietin-like 3 (ANGPTL3) and atherosclerosis: lipid and non-lipid related effects. *Journal of Cardiovascular Development and Disease*.

[30] Castro A., Lazaro I., Selva D. M. (2010). APOH is increased in the plasma and liver of type 2 diabetic patients with metabolic syndrome. *Atherosclerosis*.

[31] Chowdhury P. S., Chamoto K., Kumar A., Honjo T. (2018). PPAR-induced fatty acid oxidation in T cells increases the number of tumor-reactive CD8+T cells and facilitates anti-PD-1 therapy. *Cancer Immunology Research*.

[32] Su X., Peng D. Q. (2018). New insights into ANGPLT3 in controlling lipoprotein metabolism and risk of cardiovascular diseases. *Lipids in Health and Disease*.

[33] Cheng W., Ren X., Zhang C. (2016). Bioinformatic profiling identifies an immune-related risk signature for glioblastoma. *Neurology*.

[34] Berchtold L., Letouze E., Alexander M. P. (2021). HLA-D and PLA2R1 risk alleles associate with recurrent primary membranous nephropathy in kidney transplant recipients. *Kidney International*.

[35] Ni L., Yuan C., Zhang C. (2020). Co-expression network analysis identified LTF in association with metastasis risk and prognosis in clear cell renal cell carcinoma. *Oncotargets and Therapy*.

[36] Lenormand C., Bausinger H., Gross F. (2012). HLA-DQA2 and HLA-DQB2 genes are specifically expressed in human Langerhans cells and encode a new HLA class II molecule. *Journal of Immunology*.

[37] Na N., Zhao D., Zhang J. (2020). Carbamylated erythropoietin regulates immune responses and promotes long-term kidney allograft survival through activation of PI3K/AKT signaling. *Signal Transduction and Targeted Therapy*.

[38] Millan O., Budde K., Sommerer C. (2017). Urinary miR-155-5p and CXCL10 as prognostic and predictive biomarkers of rejection, graft outcome and treatment response in kidney transplantation. *British Journal of Clinical Pharmacology*.

[39] Zhang W., Li X., Tang Y., Chen C., Jing R., Liu T. (2020). miR-155-5p implicates in the pathogenesis of renal fibrosis via targeting SOCS1 and SOCS6. *Oxidative Medicine and Cellular Longevity*.

[40] Liu L., Pang X., Shang W., Feng G., Wang Z., Wang J. (2020). miR-136 improves renal fibrosis in diabetic rats by targeting down-regulation of tyrosine kinase SYK and inhibition of TGF-*β*1/Smad3 signaling pathway. *Renal Failure*.

[41] Guo J., Han J., Liu J., Wang S. (2020). MicroRNA-770-5p contributes to podocyte injury via targeting E2F3 in diabetic nephropathy. *Brazilian Journal of Medical and Biological Research*.

