# Supplementary material for: The Potential Diagnostic Value of Immune-Related Genes in Interstitial Fibrosis and Tubular Atrophy after Kidney Transplantation
Source: J Immunol Res. 2022 Jun 17;2022:7212852. doi: 10.1155/2022/7212852 (PMC9232312; doi:10.1155/2022/7212852)
Supplement: Supplementary Materials — Supplementary Figure 1: GSEA enrichment analysis of the IF/TA group. Supplementary Figure 2: correlation analysis between ANGPTL3 and differentially expressed immune infiltrating cells. Supplementary Figure 3: correlation analysis between APOH and differentially expressed immune infiltrating cells. Supplementary Figure 4: correlation analysis between EGF and differentially expressed immune infiltrating cells. Supplementary Figure 5: correlation analysis between FCGR2B and differentially expressed immune infiltrating cells. Supplementary Figure 6: correlation analysis between HLA-DQA2 and differentially expressed immune infiltrating cells. Supplementary Figure 7: correlation analysis between LTF and differentially expressed immune infiltrating cells. Supplementary Figure 8: IPA analysis shows the interaction network of diagnostic genes: EGF and LTF (8A), ANGPTL3 (8B), FCGR2B and APOH (8C), and HLA-DQA2 (8D). Merged the above four independent networks to comprehensively analyze the interaction of diagnostic genes (8E). Supplementary Table 1: immune-related genes. Supplementary Table 2: KEGG pathway in normal group. Supplementary Table 3: pathway of ANGPTL3 gene. Supplementary Table 4: pathway of APOH gene. Supplementary Table 5: pathway of EGF gene. Supplementary Table 6: ingenuity canonical pathways. Supplementary Table 7: category. [file 7212852.f1.zip › 7212852.f1/supplementary figure1.pdf]

Supplmentary figure 1: KEGG enrichment plot

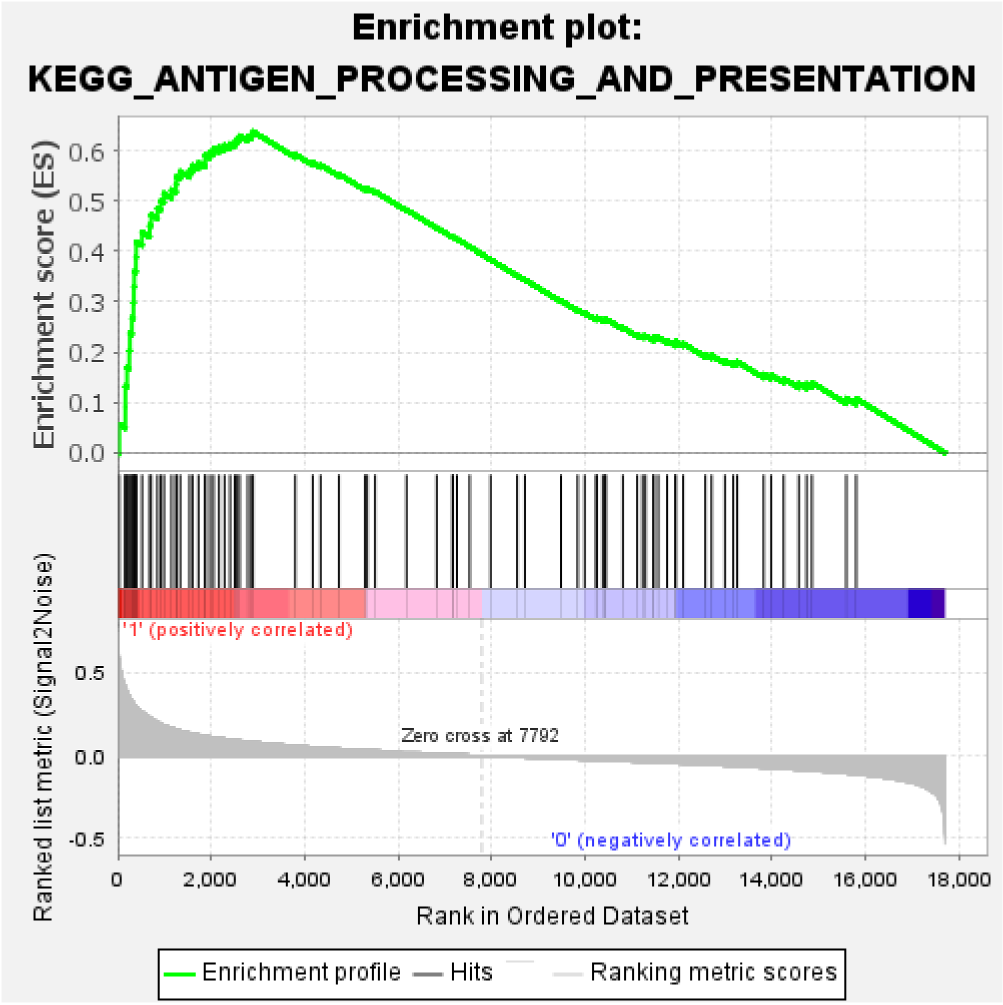

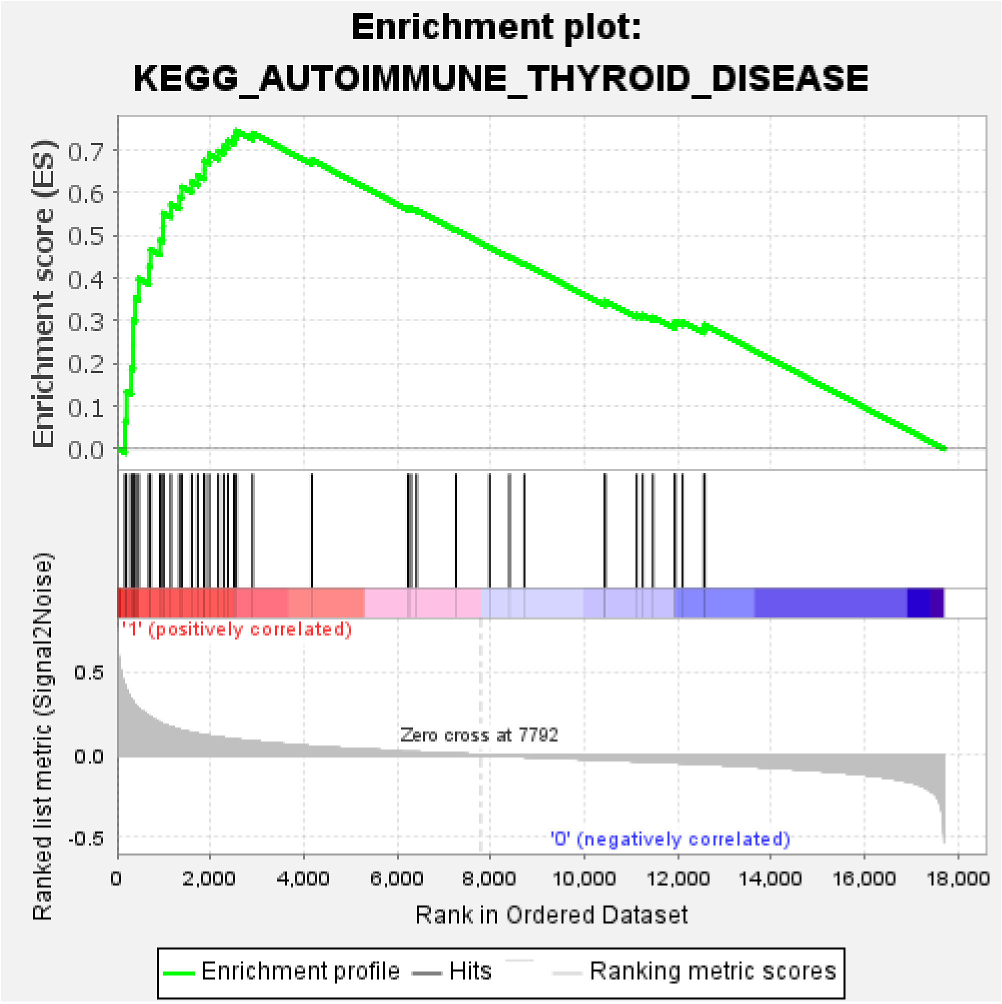

Enrichment plot:
KEGG_AUTOIMMUNE_THYROID_DISEASE
Enrichment score (ES)
0.7
0.6
0.5
0.4
0.3
0.2
0.1
0.0
'1' (positively correlated)
Zero cross at 7792
'0' (negatively correlated)
Ranked list metric (Signal2Noise)
0.5
0.0
-0.5
0
2,000
4,000
6,000
8,000
10,000
12,000
14,000
16,000
18,000
Rank in Ordered Dataset
Enrichment profile
Hits
Ranking metric scores

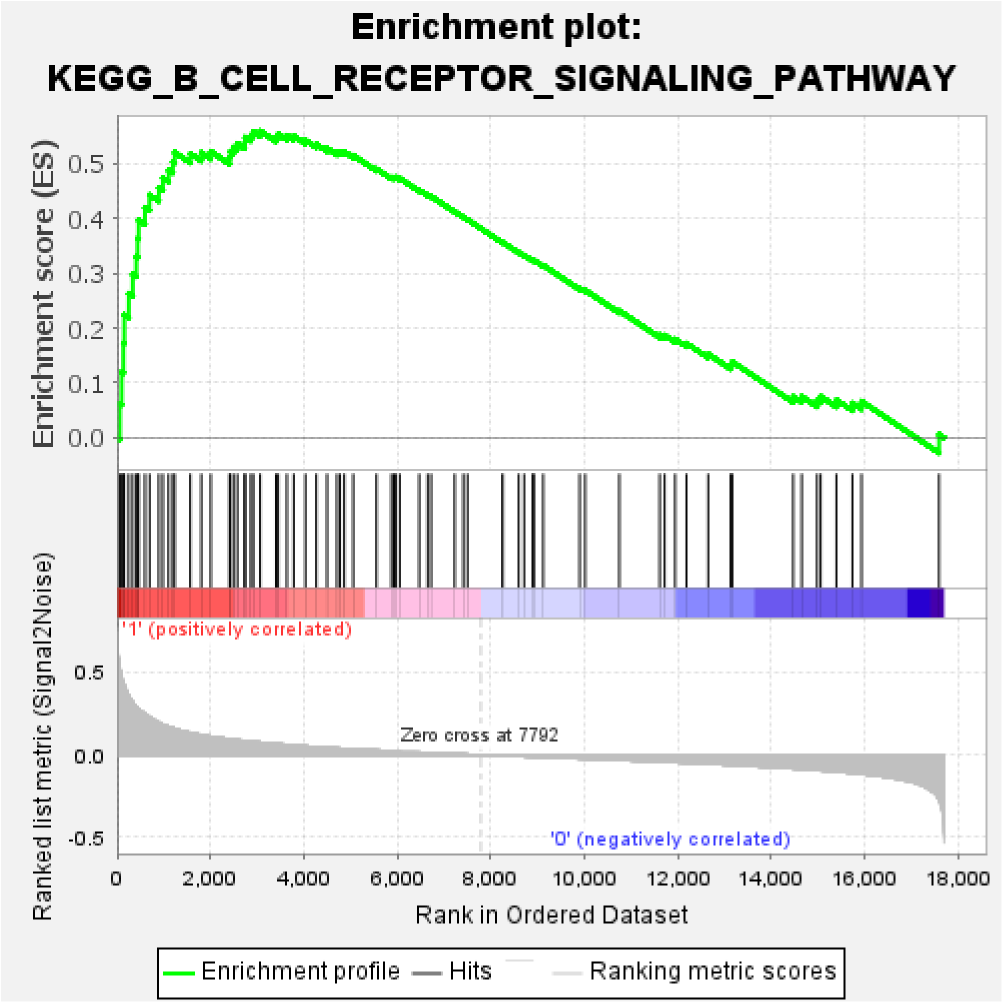

Enrichment plot:
KEGG_B_CELL_RECEPTOR_SIGNALING_PATHWAY
Enrichment score (ES)
0.5
0.4
0.3
0.2
0.1
0.0
'1' (positively correlated)
Zero cross at 7792
'0' (negatively correlated)
Ranked list metric (Signal2Noise)
0.5
0.0
-0.5
0
2,000
4,000
6,000
8,000
10,000
12,000
14,000
16,000
18,000
Rank in Ordered Dataset
Enrichment profile
Hits
Ranking metric scores

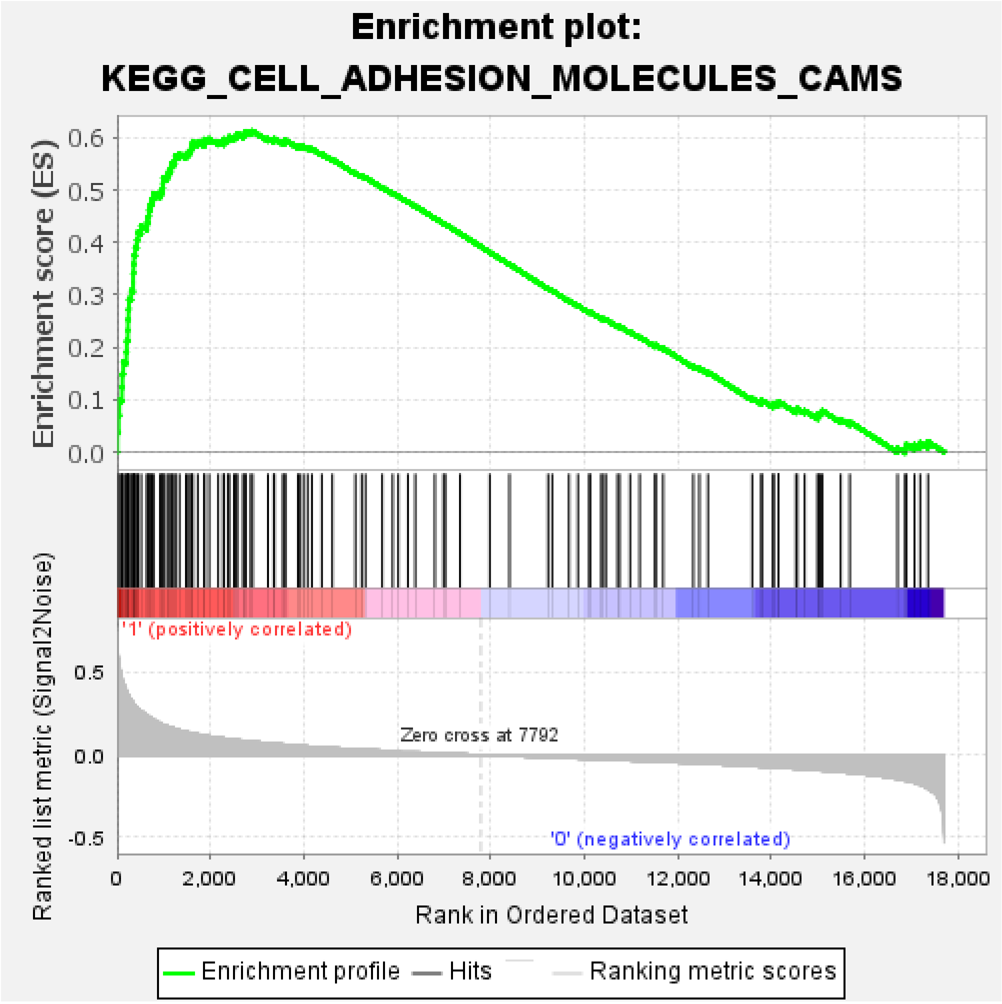
Enrichment plot:
KEGG_CELL_ADHESION_MOLECULES_CAMS
Enrichment score (ES)
0.6
0.5
0.4
0.3
0.2
0.1
0.0
'1' (positively correlated)
Zero cross at 7792
'0' (negatively correlated)
Ranked list metric (Signal2Noise)
0.5
0.0
-0.5
0
2,000
4,000
6,000
8,000
10,000
12,000
14,000
16,000
18,000
Rank in Ordered Dataset
Enrichment profile
Hits
Ranking metric scores

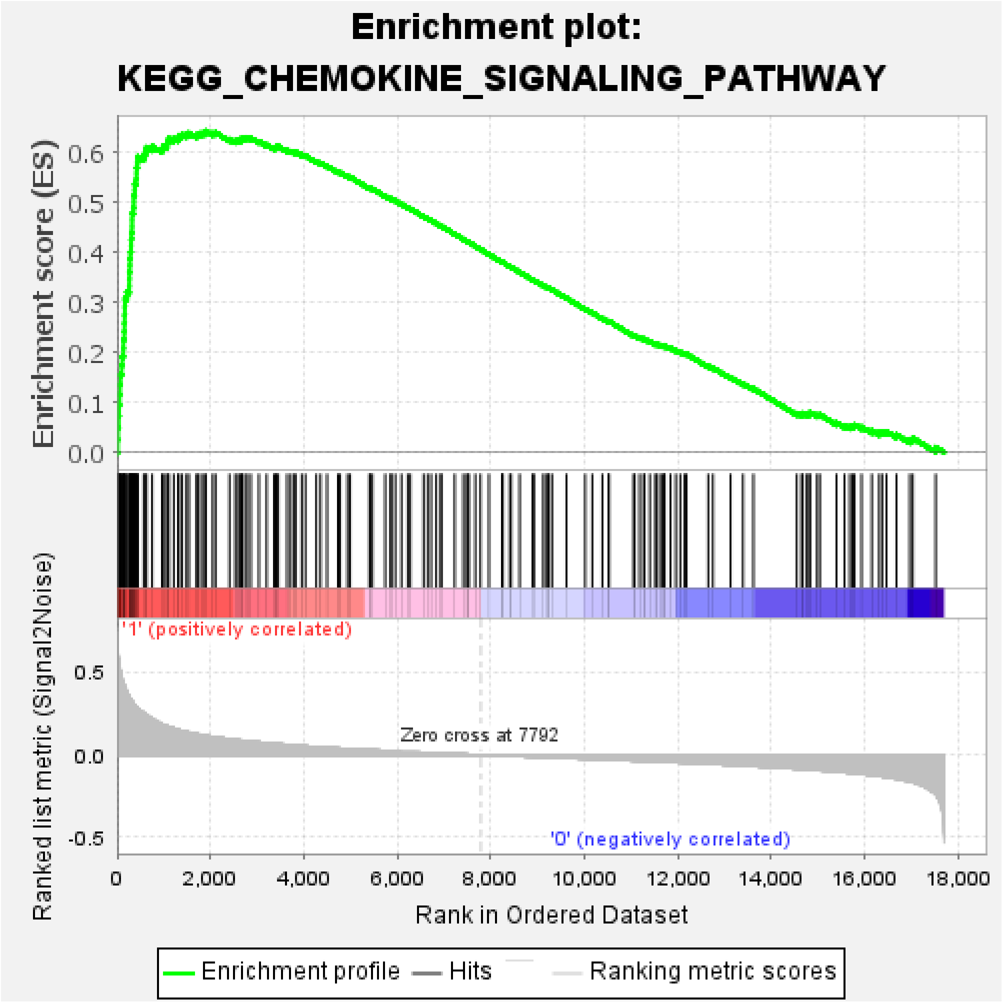

Enrichment plot:
KEGG_CHEMOKINE_SIGNALING_PATHWAY
Enrichment score (ES)
0.6
0.5
0.4
0.3
0.2
0.1
0.0
'1' (positively correlated)
Zero cross at 7792
'0' (negatively correlated)
Ranked list metric (Signal2Noise)
0.5
0.0
-0.5
0
2,000
4,000
6,000
8,000
10,000
12,000
14,000
16,000
18,000
Rank in Ordered Dataset
Enrichment profile
Hits
Ranking metric scores

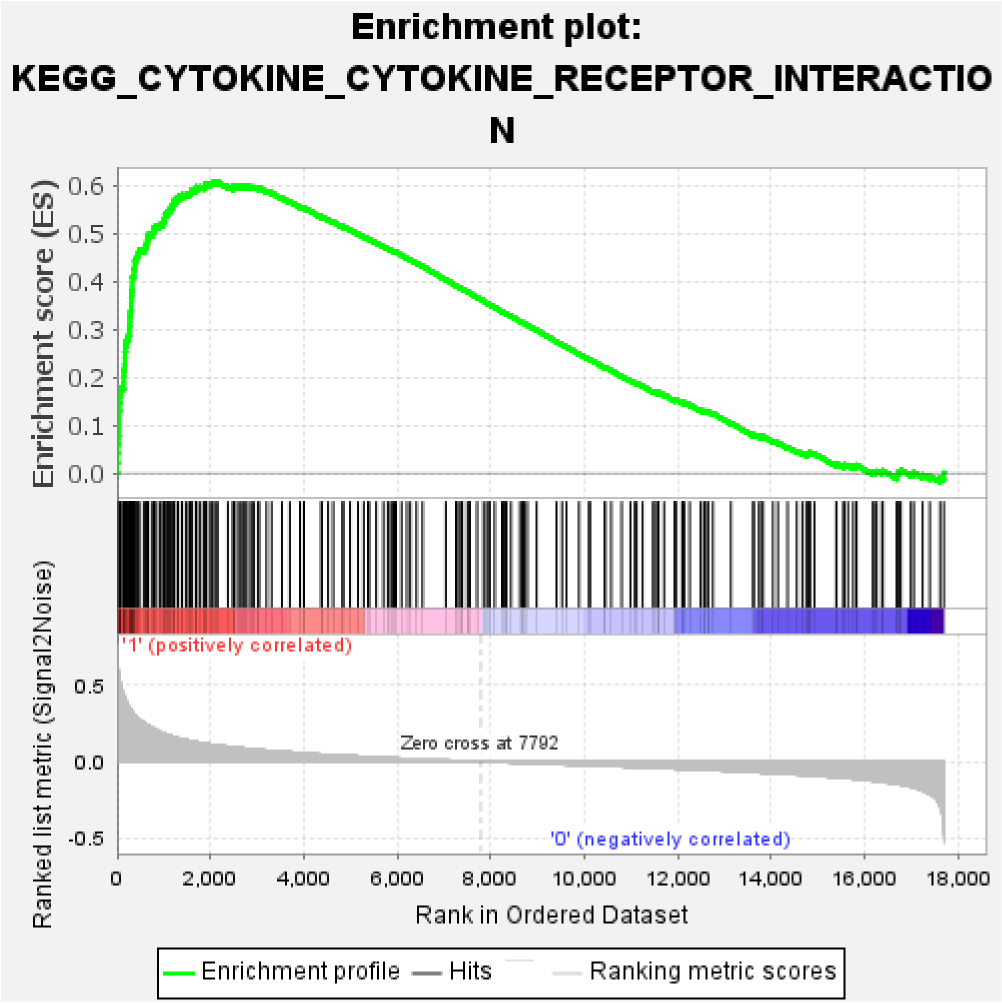
Enrichment plot:
KEGG_CYTOKINE_CYTOKINE_RECEPTOR_INTERACTIO
N
Enrichment score (ES)
0.6
0.5
0.4
0.3
0.2
0.1
0.0
'1' (positively correlated)
Zero cross at 7792
'0' (negatively correlated)
Ranked list metric (Signal2Noise)
0.5
0.0
-0.5
0
2,000
4,000
6,000
8,000
10,000
12,000
14,000
16,000
18,000
Rank in Ordered Dataset
Enrichment profile
Hits
Ranking metric scores

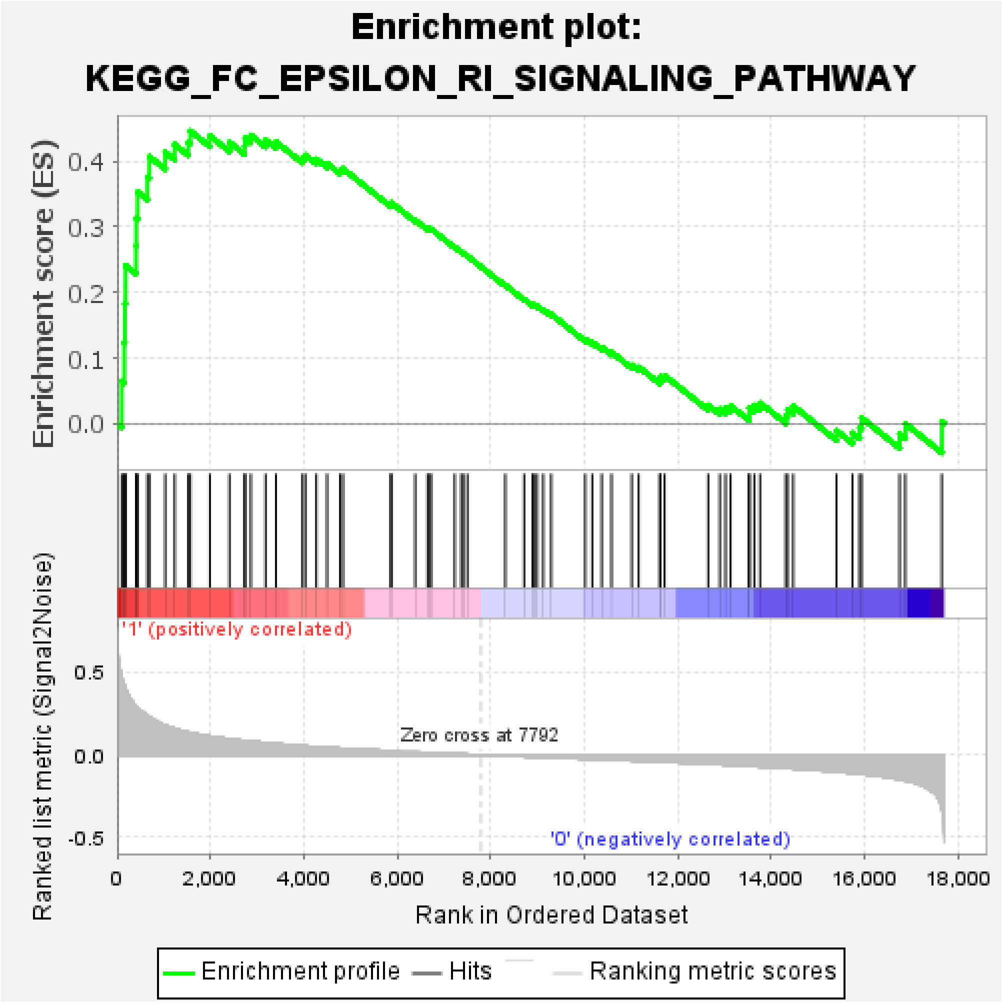

Enrichment plot:
KEGG_FC_EPSILON_RI_SIGNALING_PATHWAY
Enrichment score (ES)
0.4
0.3
0.2
0.1
0.0
'1' (positively correlated)
Ranked list metric (Signal2Noise)
0.5
0.0
-0.5
Zero cross at 7792
'0' (negatively correlated)
0
2,000
4,000
6,000
8,000
10,000
12,000
14,000
16,000
18,000
Rank in Ordered Dataset
Enrichment profile
Hits
Ranking metric scores

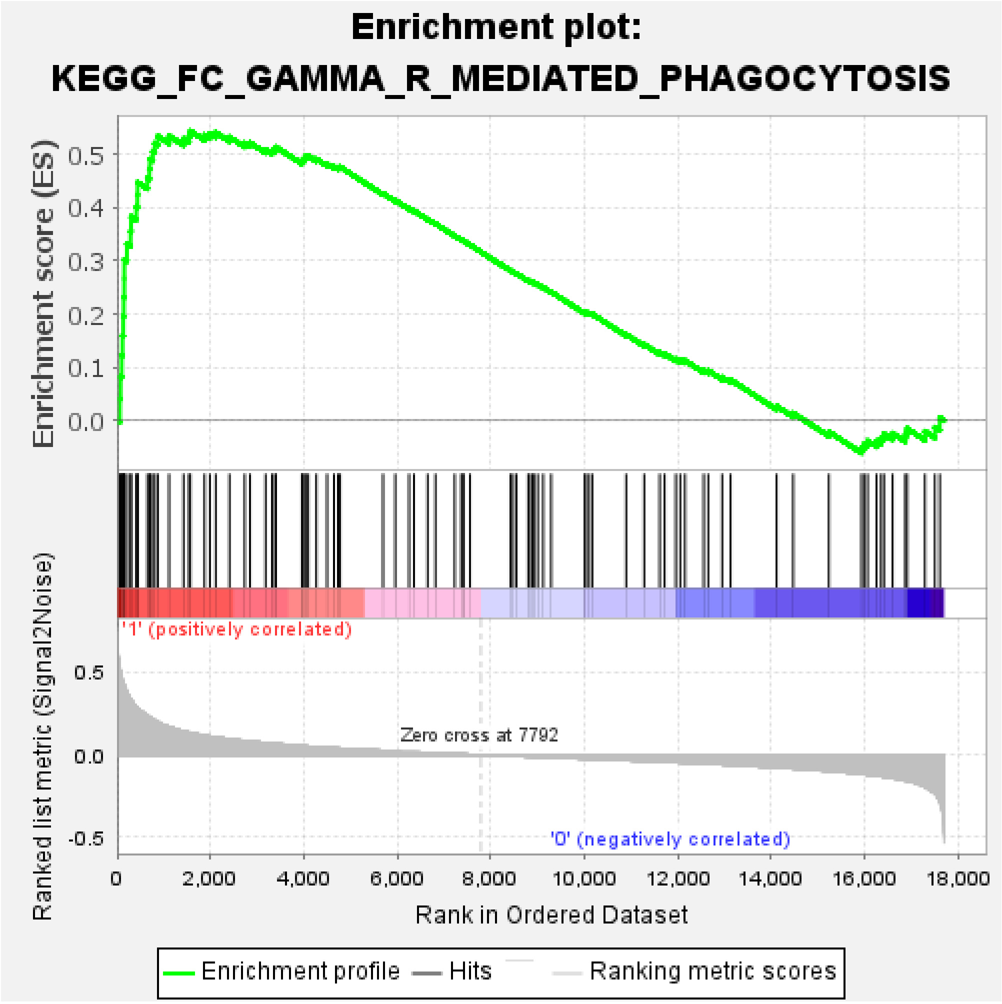

Enrichment plot:
KEGG_FC_GAMMA_R_MEDIATED_PHAGOCYTOSIS
Enrichment score (ES)
0.5
0.4
0.3
0.2
0.1
0.0
'1' (positively correlated)
Zero cross at 7792
'0' (negatively correlated)
Ranked list metric (Signal2Noise)
0.5
0.0
-0.5
0
2,000
4,000
6,000
8,000
10,000
12,000
14,000
16,000
18,000
Rank in Ordered Dataset
Enrichment profile
Hits
Ranking metric scores

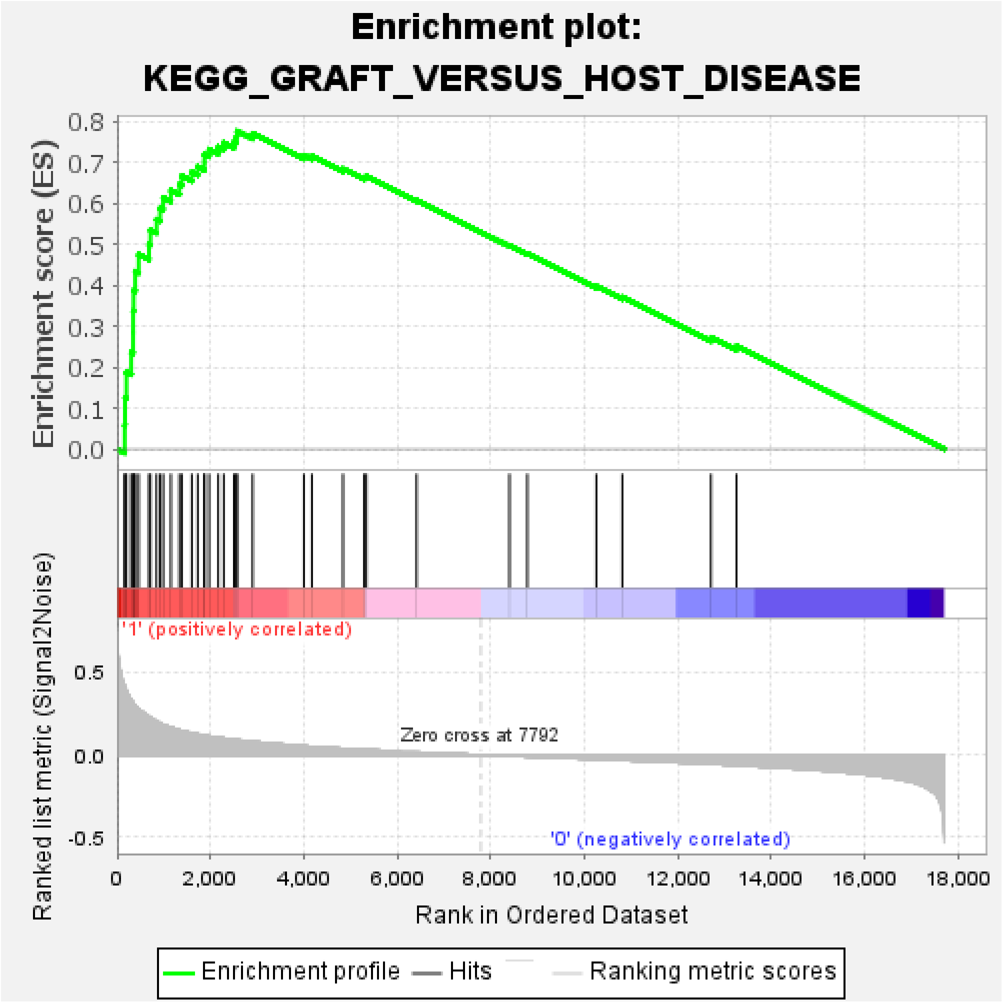

Enrichment plot:
KEGG_GRAFT_VERSUS_HOST_DISEASE
Enrichment score (ES)
0.8
0.7
0.6
0.5
0.4
0.3
0.2
0.1
0.0
Ranked list metric (Signal2Noise)
'1' (positively correlated)
Zero cross at 7792
'0' (negatively correlated)
0.5
0.0
-0.5
0
2,000
4,000
6,000
8,000
10,000
12,000
14,000
16,000
18,000
Rank in Ordered Dataset
Enrichment profile
Hits
Ranking metric scores

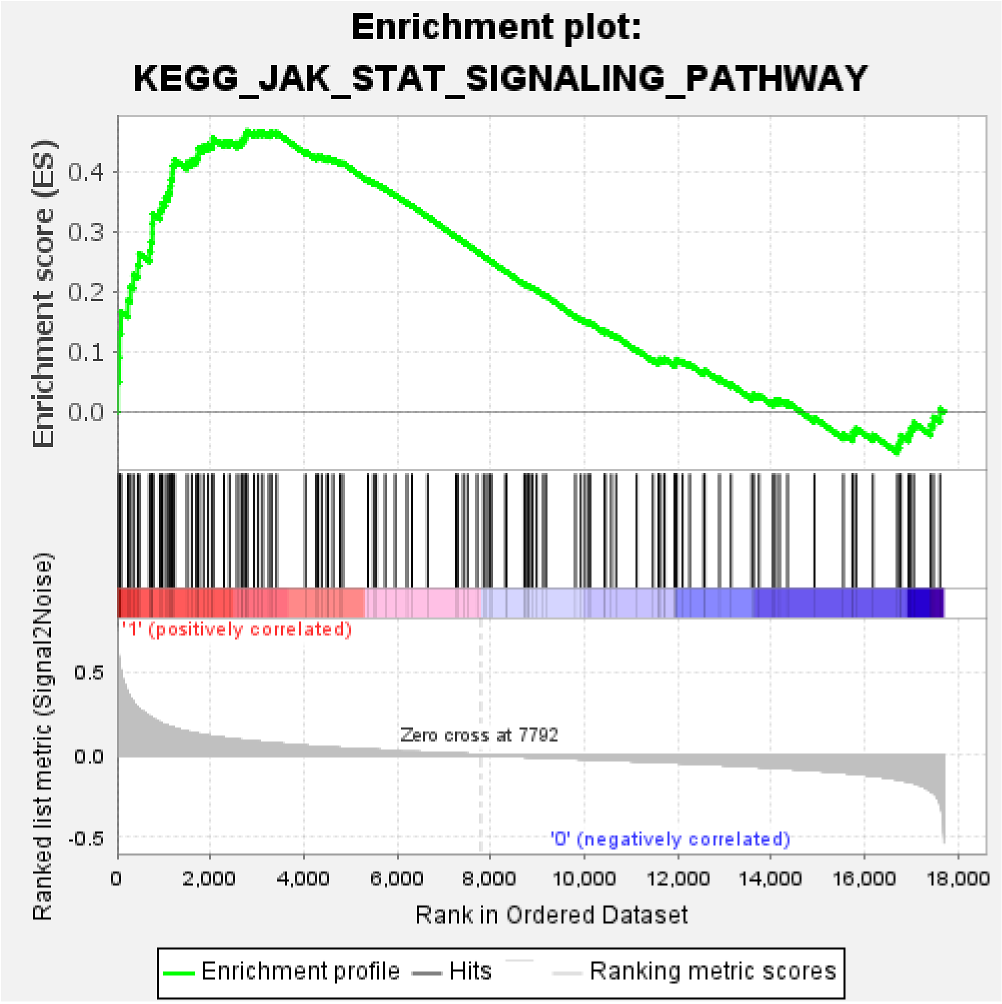

Enrichment plot:
KEGG_JAK_STAT_SIGNALING_PATHWAY
Enrichment score (ES)
0.4
0.3
0.2
0.1
0.0
'1' (positively correlated)
Zero cross at 7792
'0' (negatively correlated)
Ranked list metric (Signal2Noise)
0.5
0.0
-0.5
0
2,000
4,000
6,000
8,000
10,000
12,000
14,000
16,000
18,000
Rank in Ordered Dataset
Enrichment profile
Hits
Ranking metric scores

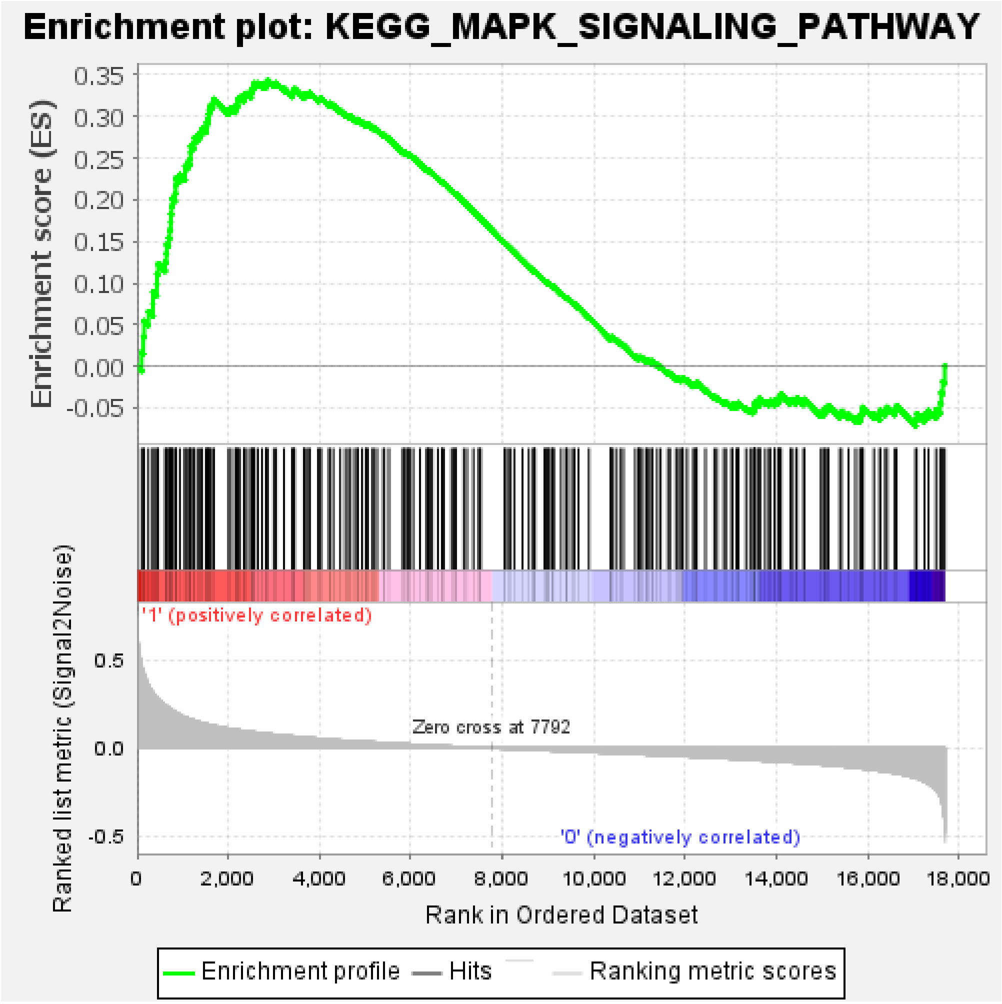

Enrichment plot: KEGG_MAPK_SIGNALING_PATHWAY
Enrichment score (ES)
0.35
0.30
0.25
0.20
0.15
0.10
0.05
0.00
-0.05
Ranked list metric (Signal2Noise)
'1' (positively correlated)
0.5
0.0
-0.5
Zero cross at 7792
'0' (negatively correlated)
0
2,000
4,000
6,000
8,000
10,000
12,000
14,000
16,000
18,000
Rank in Ordered Dataset
Enrichment profile
Hits
Ranking metric scores

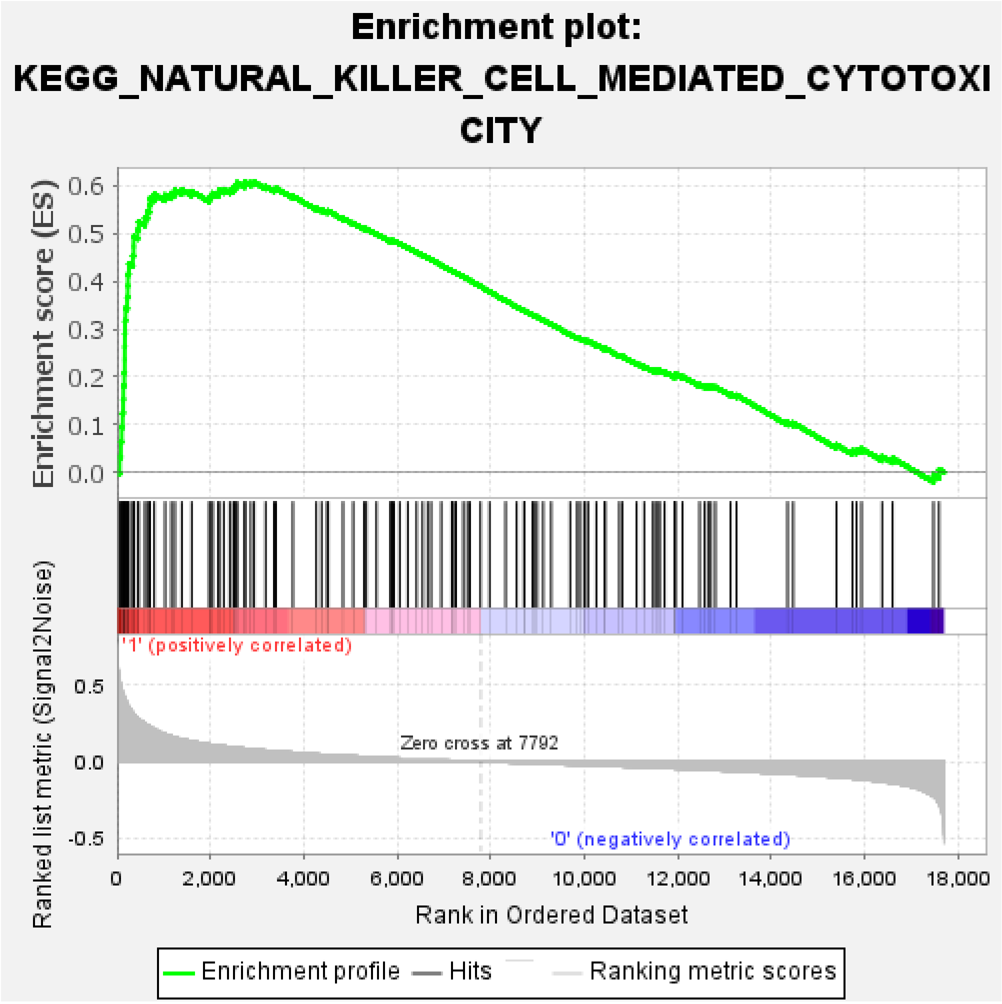

Enrichment plot:
KEGG_NATURAL_KILLER_CELL_MEDIATED_CYTOTOXICITY
Enrichment score (ES)
0.6
0.5
0.4
0.3
0.2
0.1
0.0
Ranked list metric (Signal2Noise)
'1' (positively correlated)
Zero cross at 7792
'0' (negatively correlated)
0.5
0.0
-0.5
0
2,000
4,000
6,000
8,000
10,000
12,000
14,000
16,000
18,000
Rank in Ordered Dataset
Enrichment profile
Hits
Ranking metric scores

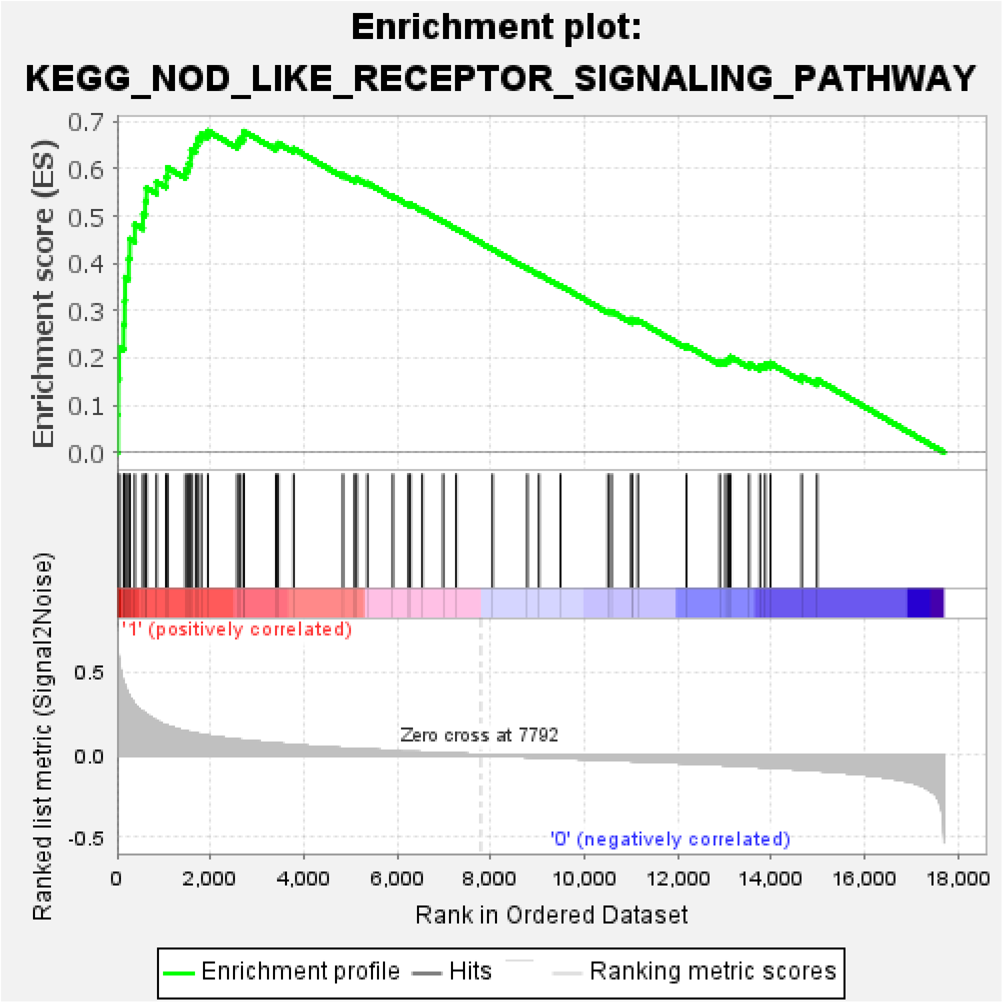
Enrichment plot:
KEGG_NOD_LIKE_RECEPTOR_SIGNALING_PATHWAY
Enrichment score (ES)
0.7
0.6
0.5
0.4
0.3
0.2
0.1
0.0
Ranked list metric (Signal2Noise)
'1' (positively correlated)
0.5
0.0
-0.5
Zero cross at 7792
'0' (negatively correlated)
0
2,000
4,000
6,000
8,000
10,000
12,000
14,000
16,000
18,000
Rank in Ordered Dataset
Enrichment profile
Hits
Ranking metric scores

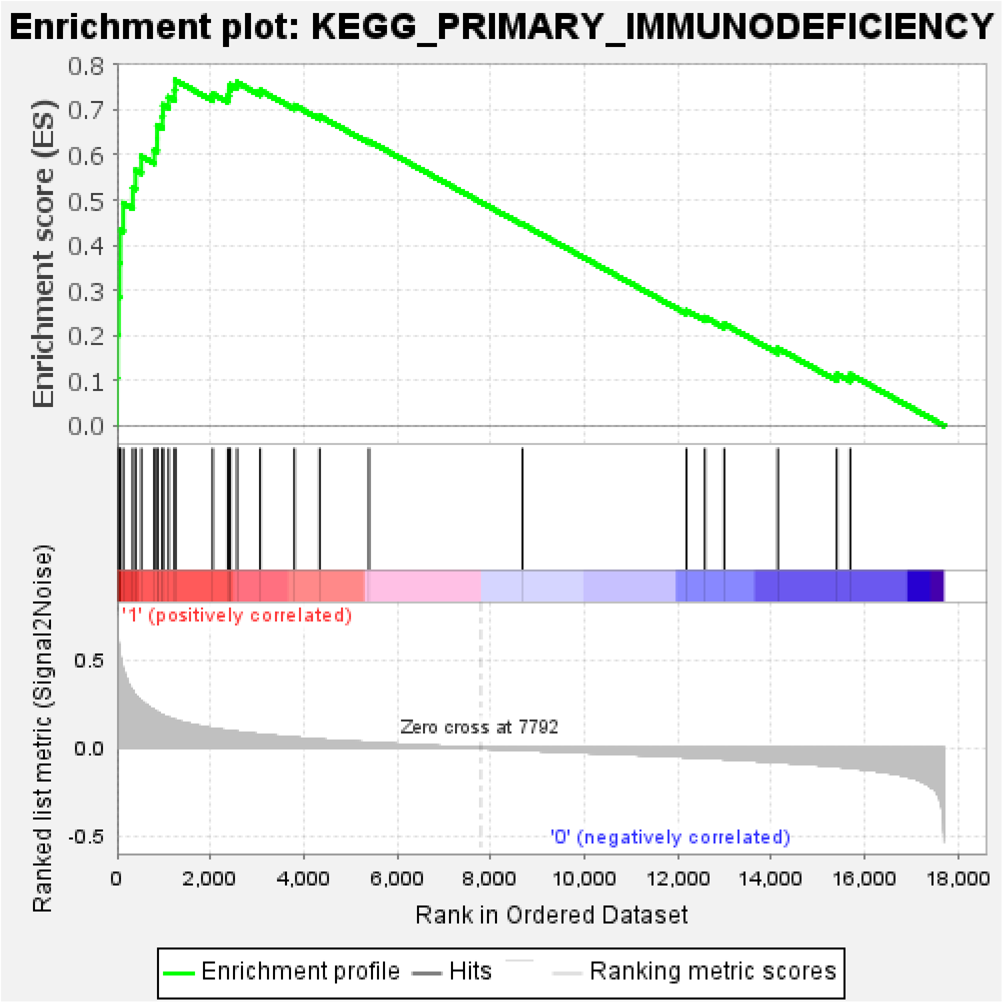
Enrichment plot: KEGG_PRIMARY_IMMUNODEFICIENCY
Enrichment score (ES)
0.8
0.7
0.6
0.5
0.4
0.3
0.2
0.1
0.0
Ranked list metric (Signal2Noise)
'1' (positively correlated)
0.5
0.0
-0.5
Zero cross at 7792
'0' (negatively correlated)
0
2,000
4,000
6,000
8,000
10,000
12,000
14,000
16,000
18,000
Rank in Ordered Dataset
Enrichment profile
Hits
Ranking metric scores

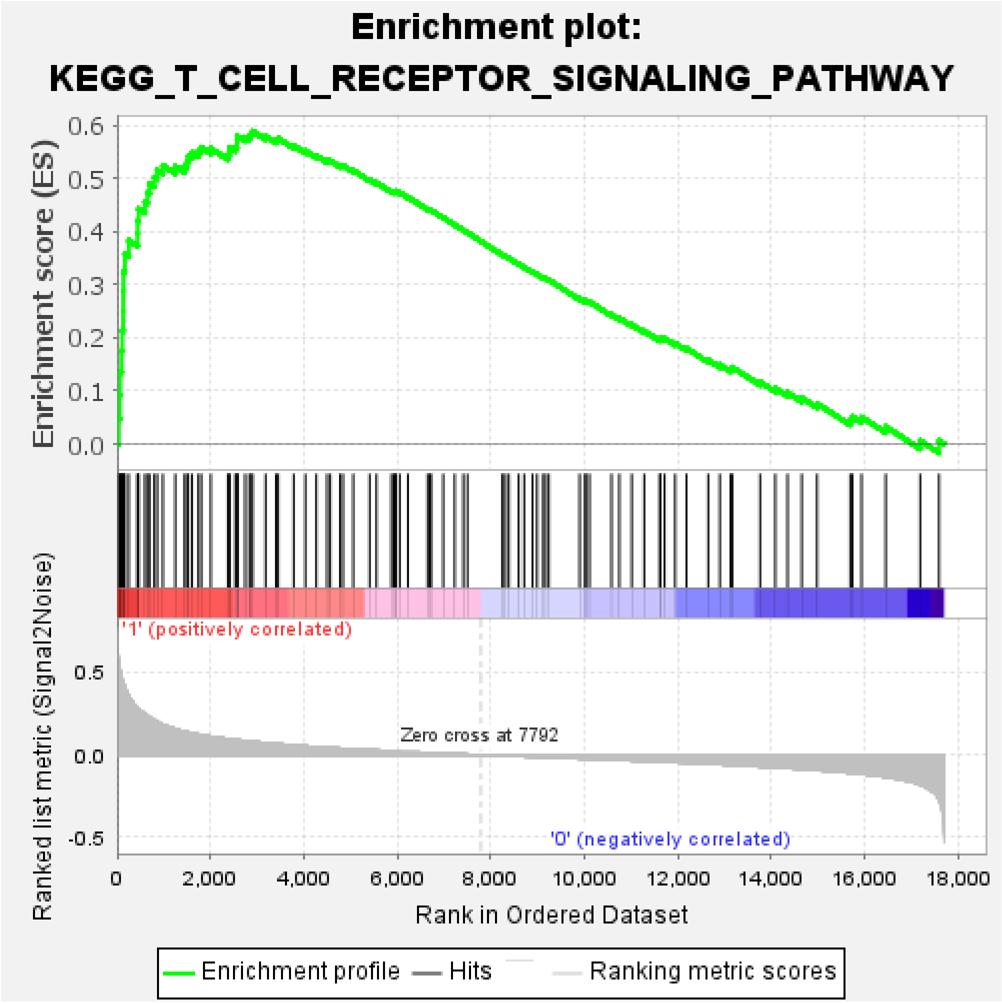
Enrichment plot:
KEGG_T_CELL_RECEPTOR_SIGNALING_PATHWAY
Enrichment score (ES)
0.6
0.5
0.4
0.3
0.2
0.1
0.0
'1' (positively correlated)
Zero cross at 7792
'0' (negatively correlated)
Ranked list metric (Signal2Noise)
0.5
0.0
-0.5
0
2,000
4,000
6,000
8,000
10,000
12,000
14,000
16,000
18,000
Rank in Ordered Dataset
Enrichment profile
Hits
Ranking metric scores

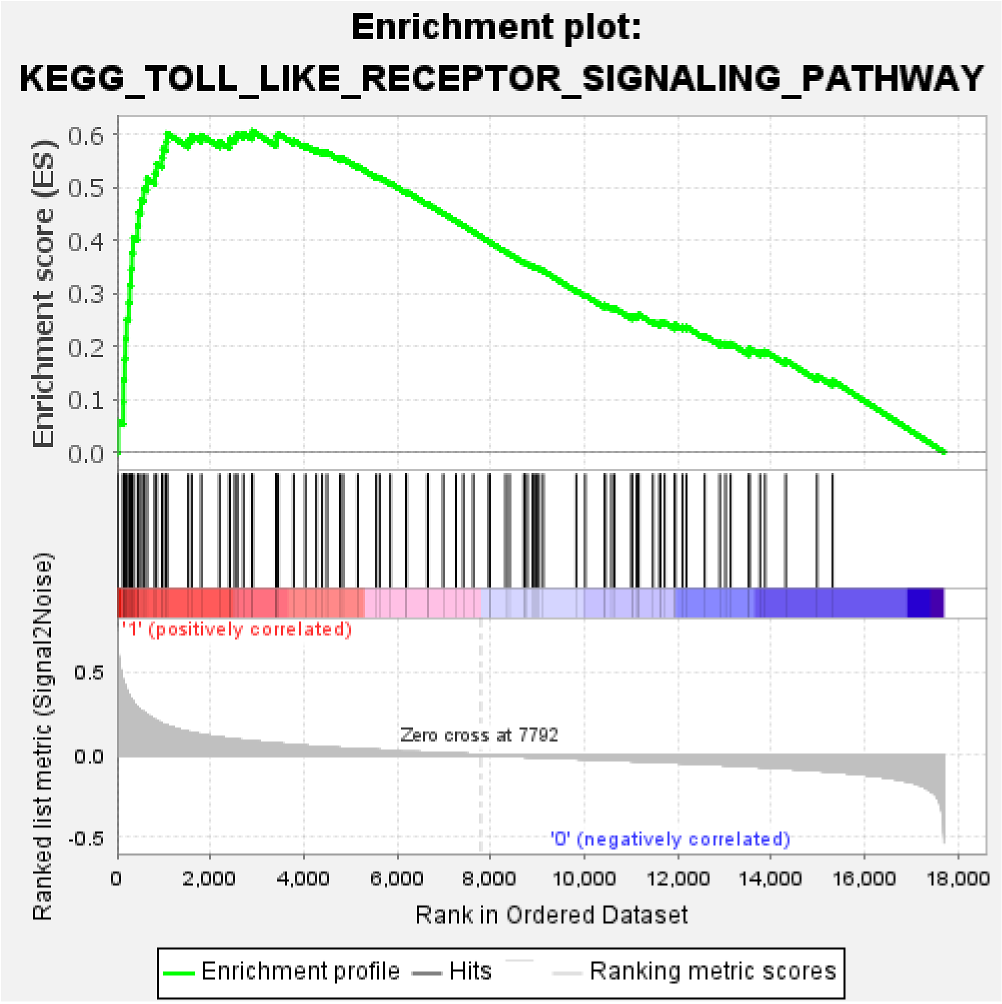
Enrichment plot:
KEGG_TOLL_LIKE_RECEPTOR_SIGNALING_PATHWAY
Enrichment score (ES)
0.6
0.5
0.4
0.3
0.2
0.1
0.0
'1' (positively correlated)
Zero cross at 7792
'0' (negatively correlated)
Ranked list metric (Signal2Noise)
0.5
0.0
-0.5
0
2,000
4,000
6,000
8,000
10,000
12,000
14,000
16,000
18,000
Rank in Ordered Dataset
Enrichment profile
Hits
Ranking metric scores

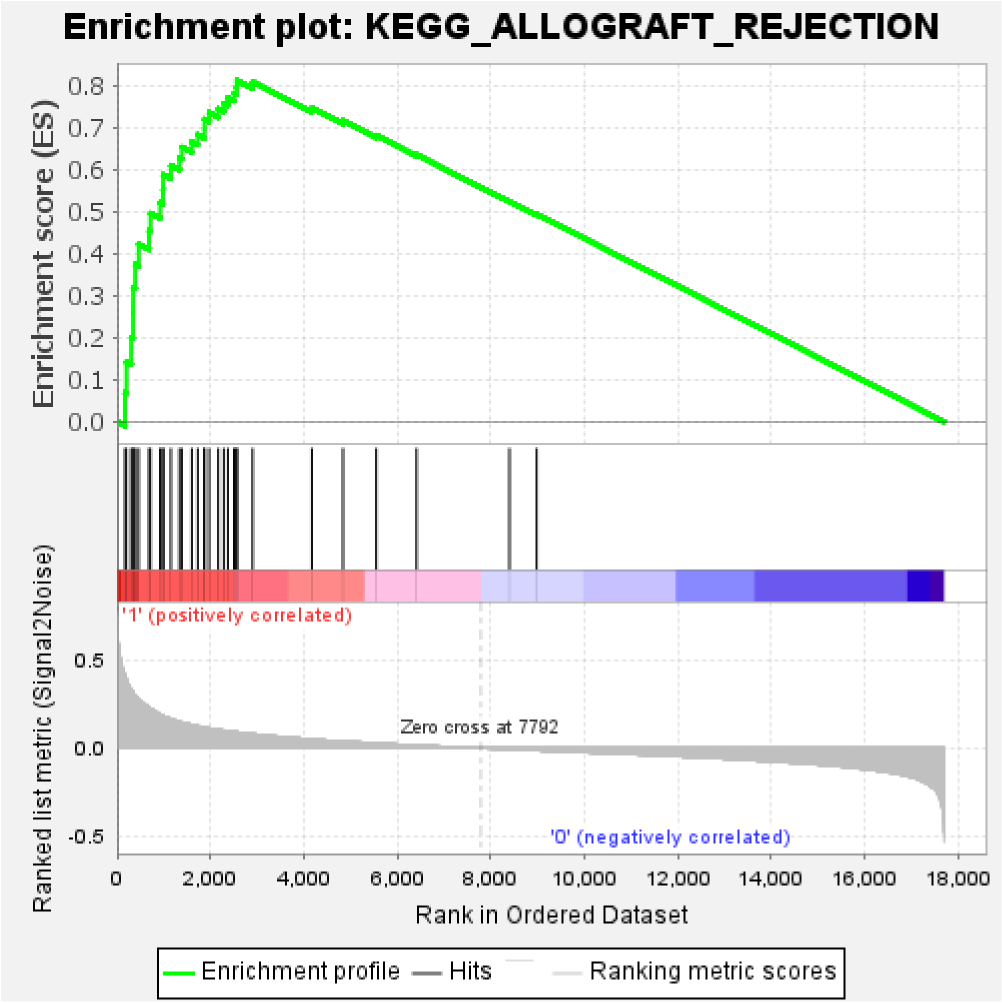
Enrichment plot: KEGG_ALLOGRAFT_REJECTION
Enrichment score (ES)
0.8
0.7
0.6
0.5
0.4
0.3
0.2
0.1
0.0
'1' (positively correlated)
Ranked list metric (Signal2Noise)
0.5
0.0
-0.5
Zero cross at 7792
'0' (negatively correlated)
0
2,000
4,000
6,000
8,000
10,000
12,000
14,000
16,000
18,000
Rank in Ordered Dataset
Enrichment profile
Hits
Ranking metric scores

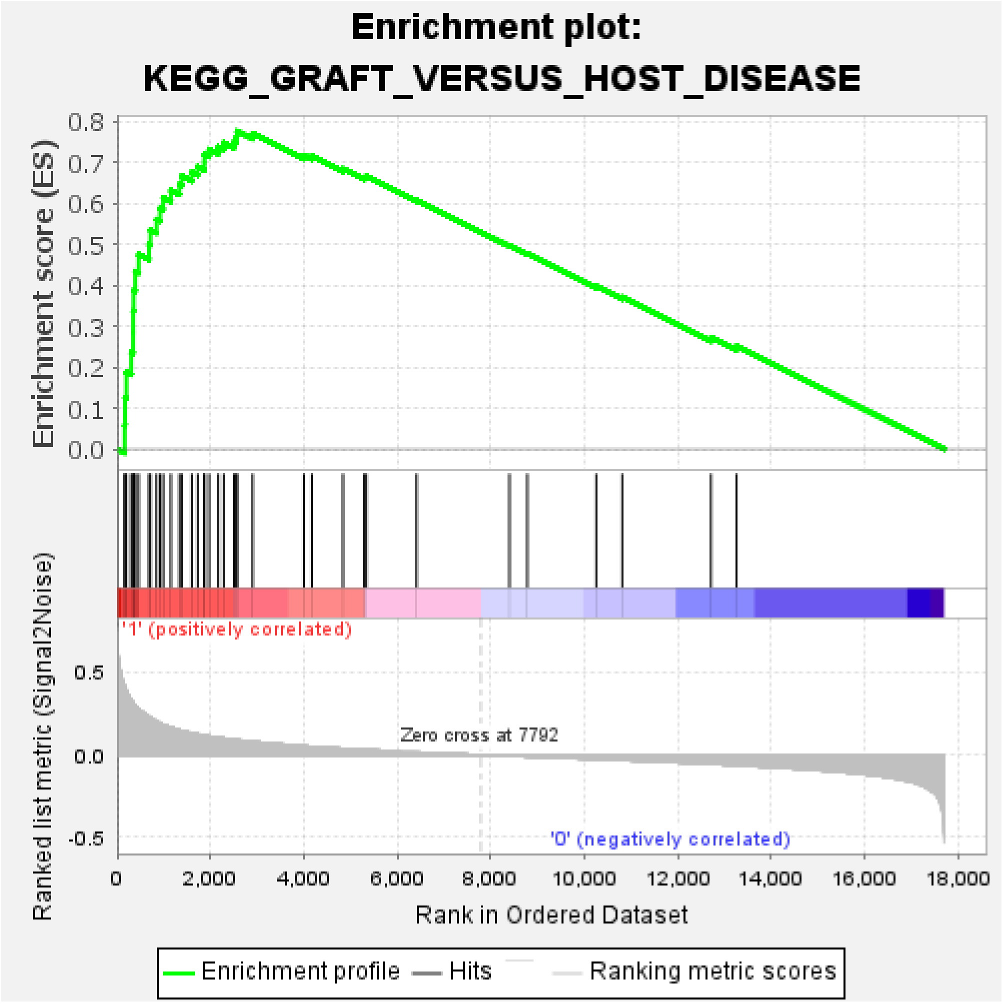
Enrichment plot:
KEGG_GRAFT_VERSUS_HOST_DISEASE
Enrichment score (ES)
0.8
0.7
0.6
0.5
0.4
0.3
0.2
0.1
0.0
'1' (positively correlated)
Ranked list metric (Signal2Noise)
0.5
0.0
-0.5
Zero cross at 7792
'0' (negatively correlated)
0
2,000
4,000
6,000
8,000
10,000
12,000
14,000
16,000
18,000
Rank in Ordered Dataset
Enrichment profile
Hits
Ranking metric scores

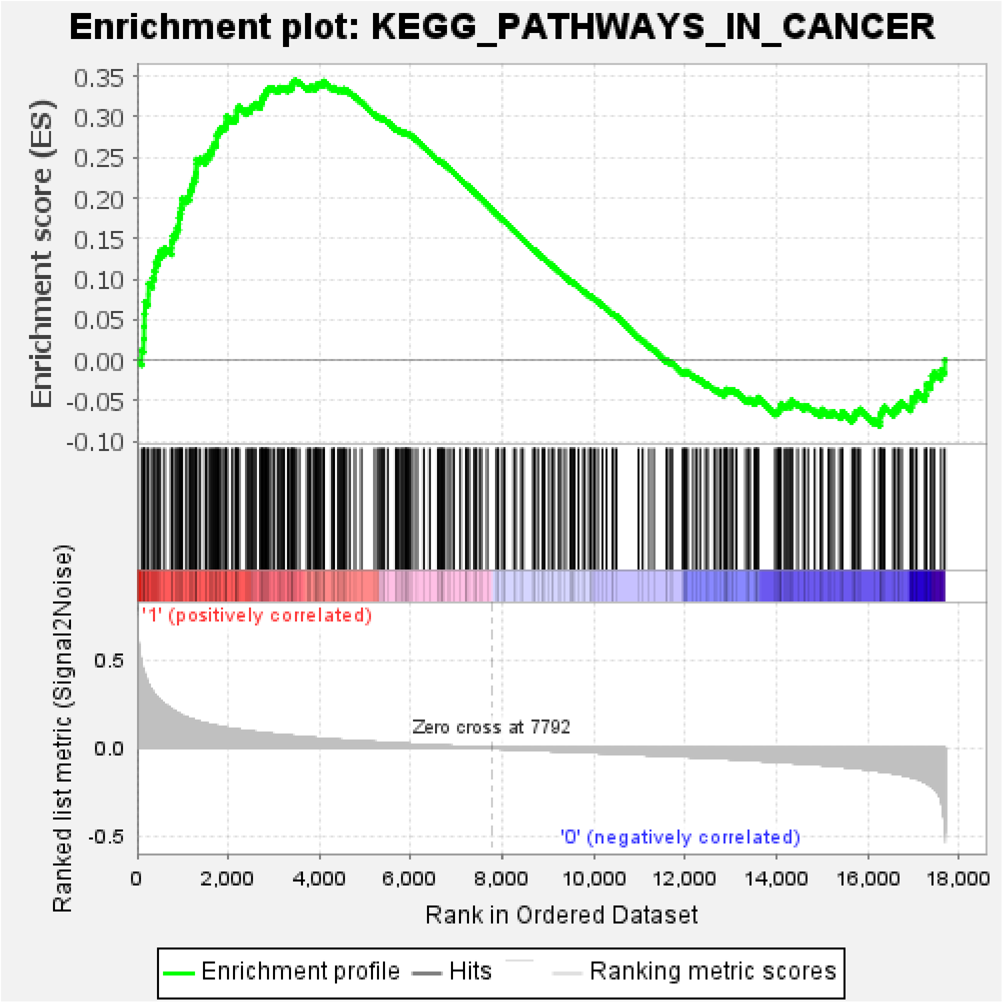

Enrichment plot: KEGG_PATHWAYS_IN_CANCER
Enrichment score (ES)
0.35
0.30
0.25
0.20
0.15
0.10
0.05
0.00
-0.05
-0.10
'1' (positively correlated)
Ranked list metric (Signal2Noise)
0.5
0.0
-0.5
Zero cross at 7792
'0' (negatively correlated)
0
2,000
4,000
6,000
8,000
10,000
12,000
14,000
16,000
18,000
Rank in Ordered Dataset
Enrichment profile
Hits
Ranking metric scores
