# Supplementary material for: The Potential Diagnostic Value of Immune-Related Genes in Interstitial Fibrosis and Tubular Atrophy after Kidney Transplantation
Source: J Immunol Res. 2022 Jun 17;2022:7212852. doi: 10.1155/2022/7212852 (PMC9232312; doi:10.1155/2022/7212852)
Supplement: Supplementary Materials — Supplementary Figure 1: GSEA enrichment analysis of the IF/TA group. Supplementary Figure 2: correlation analysis between ANGPTL3 and differentially expressed immune infiltrating cells. Supplementary Figure 3: correlation analysis between APOH and differentially expressed immune infiltrating cells. Supplementary Figure 4: correlation analysis between EGF and differentially expressed immune infiltrating cells. Supplementary Figure 5: correlation analysis between FCGR2B and differentially expressed immune infiltrating cells. Supplementary Figure 6: correlation analysis between HLA-DQA2 and differentially expressed immune infiltrating cells. Supplementary Figure 7: correlation analysis between LTF and differentially expressed immune infiltrating cells. Supplementary Figure 8: IPA analysis shows the interaction network of diagnostic genes: EGF and LTF (8A), ANGPTL3 (8B), FCGR2B and APOH (8C), and HLA-DQA2 (8D). Merged the above four independent networks to comprehensively analyze the interaction of diagnostic genes (8E). Supplementary Table 1: immune-related genes. Supplementary Table 2: KEGG pathway in normal group. Supplementary Table 3: pathway of ANGPTL3 gene. Supplementary Table 4: pathway of APOH gene. Supplementary Table 5: pathway of EGF gene. Supplementary Table 6: ingenuity canonical pathways. Supplementary Table 7: category. [file 7212852.f1.zip › 7212852.f1/supplementary figure4.pdf]

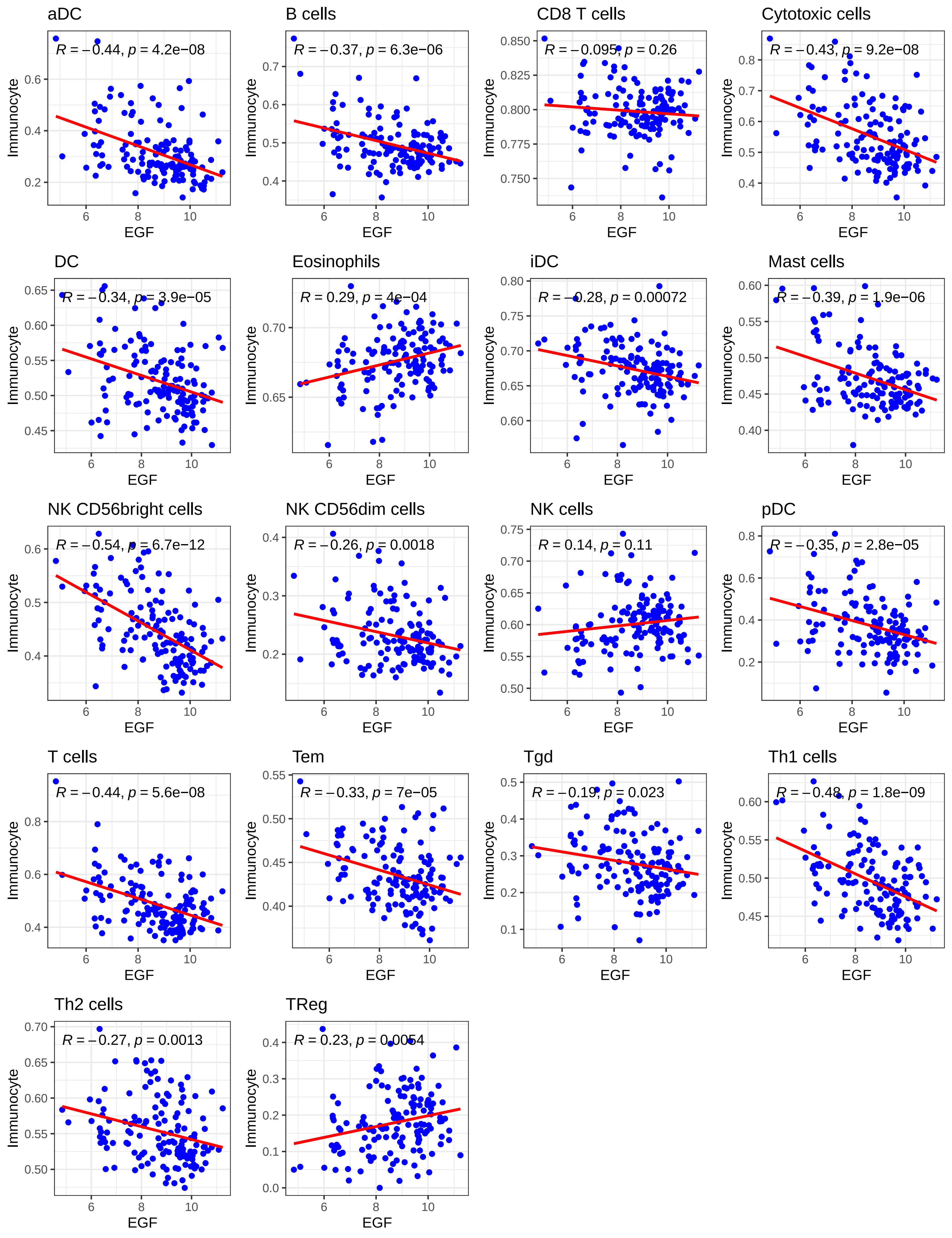
aDC
R = −0.44, p = 4.2e−08
B cells
R = −0.37, p = 6.3e−06
CD8 T cells
R = −0.095, p = 0.26
Cytotoxic cells
R = −0.43, p = 9.2e−08
DC
R = −0.34, p = 3.9e−05
Eosinophils
R = 0.29, p = 4e−04
iDC
R = −0.28, p = 0.00072
Mast cells
R = −0.39, p = 1.9e−06
NK CD56bright cells
R = −0.54, p = 6.7e−12
NK CD56dim cells
R = −0.26, p = 0.0018
NK cells
R = 0.14, p = 0.11
pDC
R = −0.35, p = 2.8e−05
T cells
R = −0.44, p = 5.6e−08
Tem
R = −0.33, p = 7e−05
Tgd
R = −0.19, p = 0.023
Th1 cells
R = −0.48, p = 1.8e−09
Th2 cells
R = −0.27, p = 0.0013
TReg
R = 0.23, p = 0.0054
Immunocyte
EGF
