# Supplementary material for: The Potential Diagnostic Value of Immune-Related Genes in Interstitial Fibrosis and Tubular Atrophy after Kidney Transplantation
Source: J Immunol Res. 2022 Jun 17;2022:7212852. doi: 10.1155/2022/7212852 (PMC9232312; doi:10.1155/2022/7212852)
Supplement: Supplementary Materials — Supplementary Figure 1: GSEA enrichment analysis of the IF/TA group. Supplementary Figure 2: correlation analysis between ANGPTL3 and differentially expressed immune infiltrating cells. Supplementary Figure 3: correlation analysis between APOH and differentially expressed immune infiltrating cells. Supplementary Figure 4: correlation analysis between EGF and differentially expressed immune infiltrating cells. Supplementary Figure 5: correlation analysis between FCGR2B and differentially expressed immune infiltrating cells. Supplementary Figure 6: correlation analysis between HLA-DQA2 and differentially expressed immune infiltrating cells. Supplementary Figure 7: correlation analysis between LTF and differentially expressed immune infiltrating cells. Supplementary Figure 8: IPA analysis shows the interaction network of diagnostic genes: EGF and LTF (8A), ANGPTL3 (8B), FCGR2B and APOH (8C), and HLA-DQA2 (8D). Merged the above four independent networks to comprehensively analyze the interaction of diagnostic genes (8E). Supplementary Table 1: immune-related genes. Supplementary Table 2: KEGG pathway in normal group. Supplementary Table 3: pathway of ANGPTL3 gene. Supplementary Table 4: pathway of APOH gene. Supplementary Table 5: pathway of EGF gene. Supplementary Table 6: ingenuity canonical pathways. Supplementary Table 7: category. [file 7212852.f1.zip › 7212852.f1/supplementary figure5.pdf]

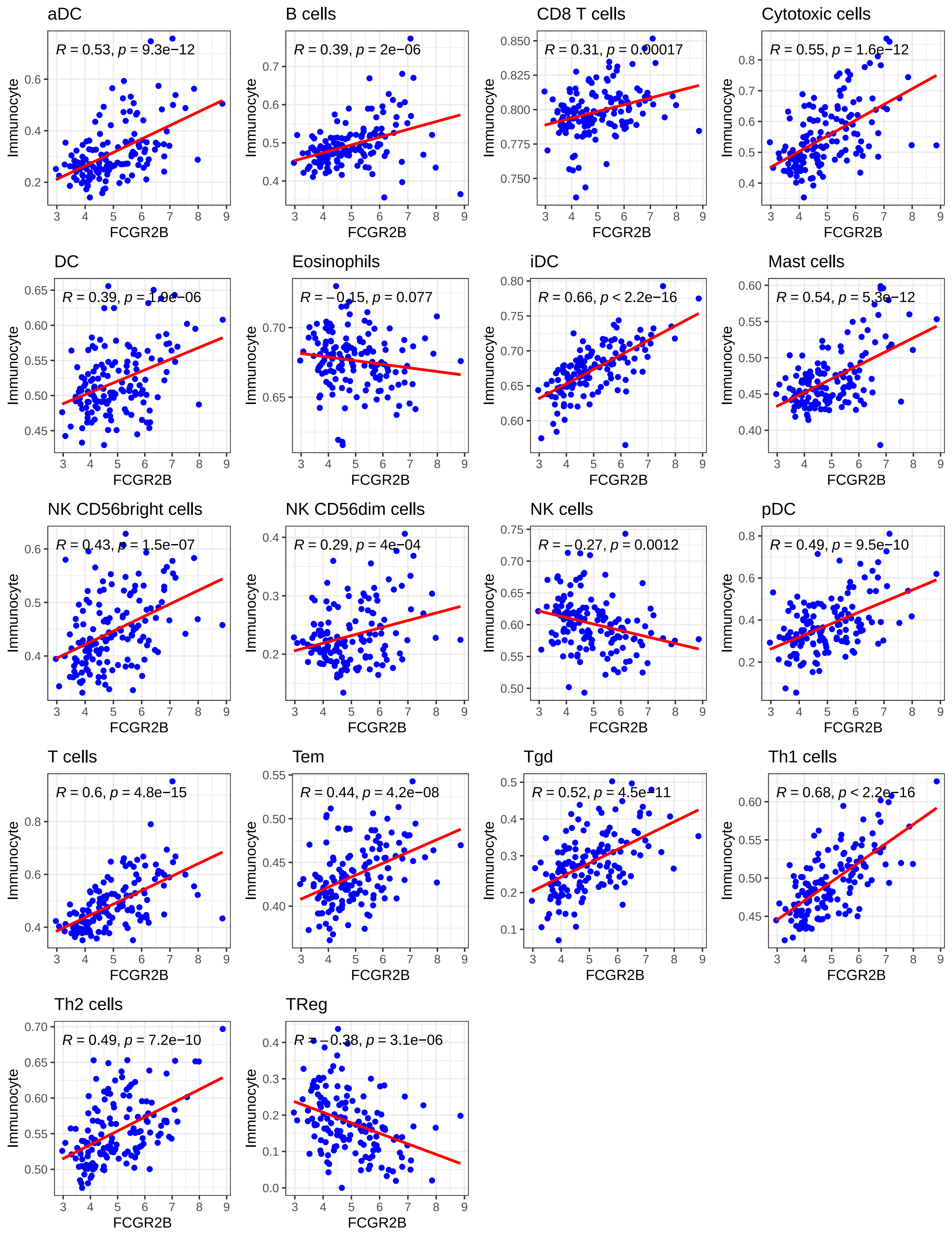

aDC
R = 0.53, p = 9.3e−12
B cells
R = 0.39, p = 2e−06
CD8 T cells
R = 0.31, p = 0.00017
Cytotoxic cells
R = 0.55, p = 1.6e−12
DC
R = 0.39, p = 1.9e−06
Eosinophils
R = −0.15, p = 0.077
iDC
R = 0.66, p < 2.2e−16
Mast cells
R = 0.54, p = 5.3e−12
NK CD56bright cells
R = 0.43, p = 1.5e−07
NK CD56dim cells
R = 0.29, p = 4e−04
NK cells
R = −0.27, p = 0.0012
pDC
R = 0.49, p = 9.5e−10
T cells
R = 0.6, p = 4.8e−15
Tem
R = 0.44, p = 4.2e−08
Tgd
R = 0.52, p = 4.5e−11
Th1 cells
R = 0.68, p < 2.2e−16
Th2 cells
R = 0.49, p = 7.2e−10
TReg
R = −0.38, p = 3.1e−06
Immunocyte
FCGR2B
