# Supplementary material for: The Potential Diagnostic Value of Immune-Related Genes in Interstitial Fibrosis and Tubular Atrophy after Kidney Transplantation
Source: J Immunol Res. 2022 Jun 17;2022:7212852. doi: 10.1155/2022/7212852 (PMC9232312; doi:10.1155/2022/7212852)
Supplement: Supplementary Materials — Supplementary Figure 1: GSEA enrichment analysis of the IF/TA group. Supplementary Figure 2: correlation analysis between ANGPTL3 and differentially expressed immune infiltrating cells. Supplementary Figure 3: correlation analysis between APOH and differentially expressed immune infiltrating cells. Supplementary Figure 4: correlation analysis between EGF and differentially expressed immune infiltrating cells. Supplementary Figure 5: correlation analysis between FCGR2B and differentially expressed immune infiltrating cells. Supplementary Figure 6: correlation analysis between HLA-DQA2 and differentially expressed immune infiltrating cells. Supplementary Figure 7: correlation analysis between LTF and differentially expressed immune infiltrating cells. Supplementary Figure 8: IPA analysis shows the interaction network of diagnostic genes: EGF and LTF (8A), ANGPTL3 (8B), FCGR2B and APOH (8C), and HLA-DQA2 (8D). Merged the above four independent networks to comprehensively analyze the interaction of diagnostic genes (8E). Supplementary Table 1: immune-related genes. Supplementary Table 2: KEGG pathway in normal group. Supplementary Table 3: pathway of ANGPTL3 gene. Supplementary Table 4: pathway of APOH gene. Supplementary Table 5: pathway of EGF gene. Supplementary Table 6: ingenuity canonical pathways. Supplementary Table 7: category. [file 7212852.f1.zip › 7212852.f1/supplementary figure6.pdf]

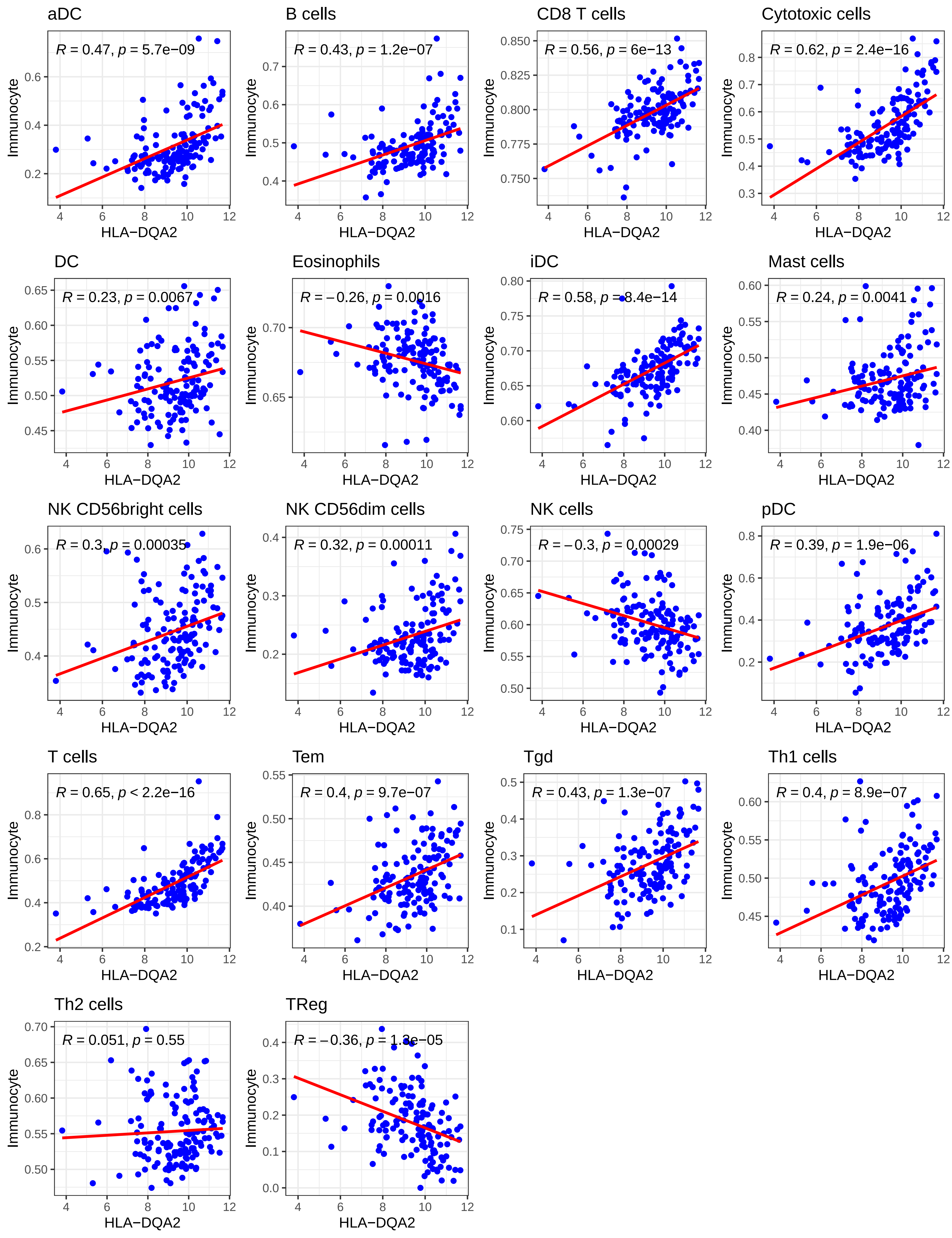

aDC
R = 0.47, p = 5.7e−09
B cells
R = 0.43, p = 1.2e−07
CD8 T cells
R = 0.56, p = 6e−13
Cytotoxic cells
R = 0.62, p = 2.4e−16
DC
R = 0.23, p = 0.0067
Eosinophils
R = −0.26, p = 0.0016
iDC
R = 0.58, p = 8.4e−14
Mast cells
R = 0.24, p = 0.0041
NK CD56bright cells
R = 0.3, p = 0.00035
NK CD56dim cells
R = 0.32, p = 0.00011
NK cells
R = −0.3, p = 0.00029
pDC
R = 0.39, p = 1.9e−06
T cells
R = 0.65, p < 2.2e−16
Tem
R = 0.4, p = 9.7e−07
Tgd
R = 0.43, p = 1.3e−07
Th1 cells
R = 0.4, p = 8.9e−07
Th2 cells
R = 0.051, p = 0.55
TReg
R = −0.36, p = 1.3e−05
Immunocyte
HLA−DQA2
