# Supplementary material for: The Potential Diagnostic Value of Immune-Related Genes in Interstitial Fibrosis and Tubular Atrophy after Kidney Transplantation
Source: J Immunol Res. 2022 Jun 17;2022:7212852. doi: 10.1155/2022/7212852 (PMC9232312; doi:10.1155/2022/7212852)
Supplement: Supplementary Materials — Supplementary Figure 1: GSEA enrichment analysis of the IF/TA group. Supplementary Figure 2: correlation analysis between ANGPTL3 and differentially expressed immune infiltrating cells. Supplementary Figure 3: correlation analysis between APOH and differentially expressed immune infiltrating cells. Supplementary Figure 4: correlation analysis between EGF and differentially expressed immune infiltrating cells. Supplementary Figure 5: correlation analysis between FCGR2B and differentially expressed immune infiltrating cells. Supplementary Figure 6: correlation analysis between HLA-DQA2 and differentially expressed immune infiltrating cells. Supplementary Figure 7: correlation analysis between LTF and differentially expressed immune infiltrating cells. Supplementary Figure 8: IPA analysis shows the interaction network of diagnostic genes: EGF and LTF (8A), ANGPTL3 (8B), FCGR2B and APOH (8C), and HLA-DQA2 (8D). Merged the above four independent networks to comprehensively analyze the interaction of diagnostic genes (8E). Supplementary Table 1: immune-related genes. Supplementary Table 2: KEGG pathway in normal group. Supplementary Table 3: pathway of ANGPTL3 gene. Supplementary Table 4: pathway of APOH gene. Supplementary Table 5: pathway of EGF gene. Supplementary Table 6: ingenuity canonical pathways. Supplementary Table 7: category. [file 7212852.f1.zip › 7212852.f1/supplementary figure7.pdf]

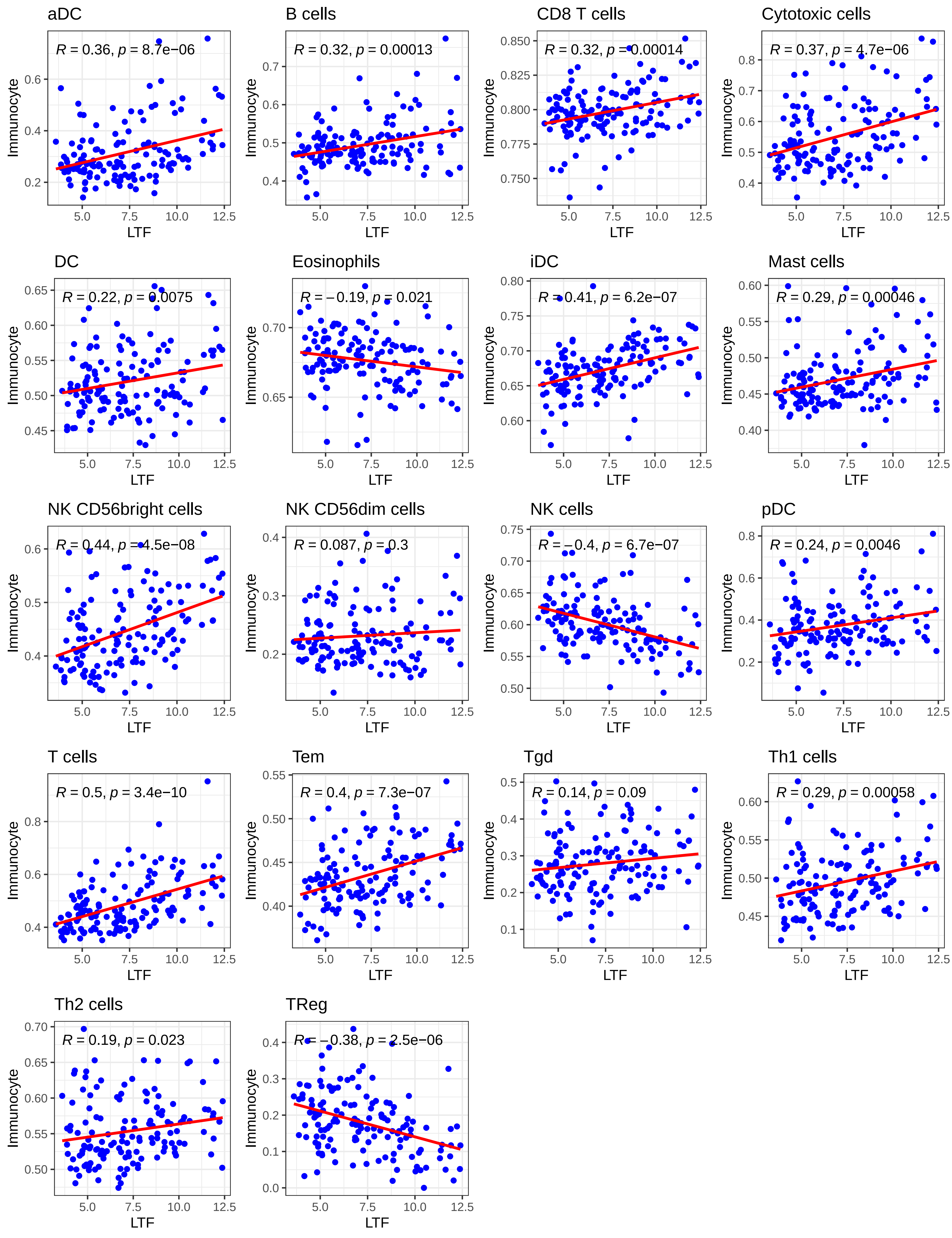

aDC
R = 0.36, p = 8.7e−06
B cells
R = 0.32, p = 0.00013
CD8 T cells
R = 0.32, p = 0.00014
Cytotoxic cells
R = 0.37, p = 4.7e−06
DC
R = 0.22, p = 0.0075
Eosinophils
R = −0.19, p = 0.021
iDC
R = 0.41, p = 6.2e−07
Mast cells
R = 0.29, p = 0.00046
NK CD56bright cells
R = 0.44, p = 4.5e−08
NK CD56dim cells
R = 0.087, p = 0.3
NK cells
R = −0.4, p = 6.7e−07
pDC
R = 0.24, p = 0.0046
T cells
R = 0.5, p = 3.4e−10
Tem
R = 0.4, p = 7.3e−07
Tgd
R = 0.14, p = 0.09
Th1 cells
R = 0.29, p = 0.00058
Th2 cells
R = 0.19, p = 0.023
TReg
R = −0.38, p = 2.5e−06
Immunocyte
LTF
