# Supplementary material for: The Potential Diagnostic Value of Immune-Related Genes in Interstitial Fibrosis and Tubular Atrophy after Kidney Transplantation
Source: J Immunol Res. 2022 Jun 17;2022:7212852. doi: 10.1155/2022/7212852 (PMC9232312; doi:10.1155/2022/7212852)
Supplement: Supplementary Materials — Supplementary Figure 1: GSEA enrichment analysis of the IF/TA group. Supplementary Figure 2: correlation analysis between ANGPTL3 and differentially expressed immune infiltrating cells. Supplementary Figure 3: correlation analysis between APOH and differentially expressed immune infiltrating cells. Supplementary Figure 4: correlation analysis between EGF and differentially expressed immune infiltrating cells. Supplementary Figure 5: correlation analysis between FCGR2B and differentially expressed immune infiltrating cells. Supplementary Figure 6: correlation analysis between HLA-DQA2 and differentially expressed immune infiltrating cells. Supplementary Figure 7: correlation analysis between LTF and differentially expressed immune infiltrating cells. Supplementary Figure 8: IPA analysis shows the interaction network of diagnostic genes: EGF and LTF (8A), ANGPTL3 (8B), FCGR2B and APOH (8C), and HLA-DQA2 (8D). Merged the above four independent networks to comprehensively analyze the interaction of diagnostic genes (8E). Supplementary Table 1: immune-related genes. Supplementary Table 2: KEGG pathway in normal group. Supplementary Table 3: pathway of ANGPTL3 gene. Supplementary Table 4: pathway of APOH gene. Supplementary Table 5: pathway of EGF gene. Supplementary Table 6: ingenuity canonical pathways. Supplementary Table 7: category. [file 7212852.f1.zip › 7212852.f1/Supplementary Files.docx]

**Supplementary Files**

**Supplementary figure1** GSEA enrichment analysis of IF/TA Group

**Supplementary figure2** Correlation analysis between ANGPTL3 and differentially expressed immune infiltrating cells

**Supplementary figure3** Correlation analysis between APOH and differentially expressed immune infiltrating cells

**Supplementary figure4** Correlation analysis between EGF and differentially expressed immune infiltrating cells

**Supplementary figure5** Correlation analysis between FCGR2B and differentially expressed immune infiltrating cells

**Supplementary figure6** Correlation analysis between HLA-DQA2 and differentially expressed immune infiltrating cells

**Supplementary figure7** Correlation analysis between LTF and differentially expressed immune infiltrating cells

**Supplemeantary figure8** IPA analysis shows the interaction network of diagnostic genes：EGF and LTF (8A), ANGPTL3 (8B), FCGR2B, and APOH (8C), and HLA-DQA2 (8D). Merged the above four independent networks to comprehensively analyze the interaction of diagnostic genes (8E)

**Supplementary Table 1** Immune related genes

**Supplementary Table 2** KEGG pathway in normal group

**Supplementary Table 3** Pathway of ANGPTL3 gene

**Supplementary Table 4** Pathway of APOH gene

**Supplementary Table 5** Pathway of EGF gene

**Supplementary Table 6** Ingenuity Canonical Pathways

**Supplementary Table 7** Category
