# Supplementary material for: The Potential Diagnostic Value of Immune-Related Genes in Interstitial Fibrosis and Tubular Atrophy after Kidney Transplantation
Source: J Immunol Res. 2022 Jun 17;2022:7212852. doi: 10.1155/2022/7212852 (PMC9232312; doi:10.1155/2022/7212852)
Supplement: Supplementary Materials — Supplementary Figure 1: GSEA enrichment analysis of the IF/TA group. Supplementary Figure 2: correlation analysis between ANGPTL3 and differentially expressed immune infiltrating cells. Supplementary Figure 3: correlation analysis between APOH and differentially expressed immune infiltrating cells. Supplementary Figure 4: correlation analysis between EGF and differentially expressed immune infiltrating cells. Supplementary Figure 5: correlation analysis between FCGR2B and differentially expressed immune infiltrating cells. Supplementary Figure 6: correlation analysis between HLA-DQA2 and differentially expressed immune infiltrating cells. Supplementary Figure 7: correlation analysis between LTF and differentially expressed immune infiltrating cells. Supplementary Figure 8: IPA analysis shows the interaction network of diagnostic genes: EGF and LTF (8A), ANGPTL3 (8B), FCGR2B and APOH (8C), and HLA-DQA2 (8D). Merged the above four independent networks to comprehensively analyze the interaction of diagnostic genes (8E). Supplementary Table 1: immune-related genes. Supplementary Table 2: KEGG pathway in normal group. Supplementary Table 3: pathway of ANGPTL3 gene. Supplementary Table 4: pathway of APOH gene. Supplementary Table 5: pathway of EGF gene. Supplementary Table 6: ingenuity canonical pathways. Supplementary Table 7: category. [file 7212852.f1.zip › 7212852.f1/supplementary table1.pdf]

Symbol	ID	Name	Synonyms	Chromoso	Category
AZGP1	563	alpha-2-gl	ZA2G|ZAG	7	Antigen_Processing_and_Presentation
B2M	567	beta-2-mi	IMD43	15	Antigen_Processing_and_Presentation
CALR	811	calreticulin	CRT|HEL-S	19	Antigen_Processing_and_Presentation
CANX	821	calnexin	CNX|IP90|F	5	Antigen_Processing_and_Presentation
CD1A	909	CD1a mole	CD1|FCB6|	1	Antigen_Processing_and_Presentation
CD1B	910	CD1b mole	CD1|CD1A	1	Antigen_Processing_and_Presentation
CD1C	911	CD1c mole	BDCA1|CD	1	Antigen_Processing_and_Presentation
CD1D	912	CD1d mole	CD1A|R3|R	1	Antigen_Processing_and_Presentation
CD1E	913	CD1e mole	CD1A|R2	1	Antigen_Processing_and_Presentation
CD4	920	CD4 molec	CD4mut	12	Antigen_Processing_and_Presentation
CD8A	925	CD8a mole	CD8|Leu2|p	2	Antigen_Processing_and_Presentation
CD8B	926	CD8b mole	CD8B1|LEU	2	Antigen_Processing_and_Presentation
CD74	972	CD74 mole	DHLAG|HL	5	Antigen_Processing_and_Presentation
CREB1	1385	cAMP resp	CREB|CREE	2	Antigen_Processing_and_Presentation
CTSB	1508	cathepsin I	APPS|CPSE	8	Antigen_Processing_and_Presentation
CTSE	1510	cathepsin I	CATE	1	Antigen_Processing_and_Presentation
CTSL	1514	cathepsin I	CATL|CTSL	9	Antigen_Processing_and_Presentation
CTSS	1520	cathepsin S	-	1	Antigen_Processing_and_Presentation
FCER1G	2207	Fc fragmer	FCRG	1	Antigen_Processing_and_Presentation
FCGRT	2217	Fc fragmer	FCRN|alph	19	Antigen_Processing_and_Presentation
PDIA3	2923	protein dis	ER60|ERp5	15	Antigen_Processing_and_Presentation
HFE	3077	homeostat	HFE1|HH|H	6	Antigen_Processing_and_Presentation
HLA-A	3105	major histo	HLAA	6	Antigen_Processing_and_Presentation
HLA-B	3106	major histo	AS|B-4901	6	Antigen_Processing_and_Presentation
HLA-C	3107	major histo	D6S204|HL	6	Antigen_Processing_and_Presentation
HLA-DMA	3108	major histo	D6S222E|D	6	Antigen_Processing_and_Presentation
HLA-DMB	3109	major histo	D6S221E|R	6	Antigen_Processing_and_Presentation
HLA-DOA	3111	major histo	HLA-DNA|	6	Antigen_Processing_and_Presentation
HLA-DOB	3112	major histo	DOB|HLA_	6	Antigen_Processing_and_Presentation
HLA-DPA1	3113	major histo	DP(W3)|DF	6	Antigen_Processing_and_Presentation
HLA-DPB1	3115	major histo	DPB1|HLA-	6	Antigen_Processing_and_Presentation
HLA-DQA1	3117	major histo	CELIAC1|D	6	Antigen_Processing_and_Presentation
HLA-DQA2	3118	major histo	DC-alpha|I	6	Antigen_Processing_and_Presentation
HLA-DQB1	3119	major histo	CELIAC1|H	6	Antigen_Processing_and_Presentation
HLA-DRA	3122	major histo	HLA-DRA1	6	Antigen_Processing_and_Presentation
HLA-DRB1	3123	major histo	DRB1|HLA-	6	Antigen_Processing_and_Presentation
HLA-DRB3	3125	major histo	DRB3|HLA-	6	Antigen_Processing_and_Presentation
HLA-DRB4	3126	major histo	DR4|DRB4|	6	Antigen_Processing_and_Presentation
HLA-DRB5	3127	major histo	-	6	Antigen_Processing_and_Presentation
HLA-E	3133	major histo	HLA-6.2|Q	6	Antigen_Processing_and_Presentation
HLA-F	3134	major histo	CDA12|HL	6	Antigen_Processing_and_Presentation
HLA-G	3135	major histo	MHC-G	6	Antigen_Processing_and_Presentation
HLA-H	3136	major histo	HLAHP	6	Antigen_Processing_and_Presentation
MR1	3140	major histo	HLALS	1	Antigen_Processing_and_Presentation
HSPA1A	3303	heat shock	HEL-S-103	6	Antigen_Processing_and_Presentation
HSPA1B	3304	heat shock	HSP70-1|H	6	Antigen_Processing_and_Presentation
HSPA1L	3305	heat shock	HSP70-1L|	6	Antigen_Processing_and_Presentation
HSPA2	3306	heat shock	HSP70-2|H	14	Antigen_Processing_and_Presentation
HSPA4	3308	heat shock	APG-2|HEL	5	Antigen_Processing_and_Presentation
HSPA5	3309	heat shock	BIP|GRP78|	9	Antigen_Processing_and_Presentation
HSPA6	3310	heat shock	HSP70B'	1	Antigen_Processing_and_Presentation
HSPA8	3312	heat shock	HEL-33|HE	11	Antigen_Processing_and_Presentation
HSP90AA1	3320	heat shock	EL52|HEL-S	14	Antigen_Processing_and_Presentation
HSP90AB1	3326	heat shock	D6S182|HS	6	Antigen_Processing_and_Presentation
ICAM1	3383	intercellula	BB2|CD54|I	19	Antigen_Processing_and_Presentation
IFNA1	3439	interferon	IFL|IFN|IFN	9	Antigen_Processing_and_Presentation
IFNA2	3440	interferon	IFN-alpha-	9	Antigen_Processing_and_Presentation

IFNA4	3441	interferon a	IFN-alpha4	9	Antigen_Processing_and_Presentation
IFNA5	3442	interferon a	IFN-alpha-	9	Antigen_Processing_and_Presentation
IFNA6	3443	interferon a	IFN-alphaK	9	Antigen_Processing_and_Presentation
IFNA7	3444	interferon a	IFN-alphaJ	9	Antigen_Processing_and_Presentation
IFNA8	3445	interferon a	IFN-alphaB	9	Antigen_Processing_and_Presentation
IFNA10	3446	interferon a	IFN-alphaC	9	Antigen_Processing_and_Presentation
IFNA13	3447	interferon a	-	9	Antigen_Processing_and_Presentation
IFNA14	3448	interferon a	IFN-alphaH	9	Antigen_Processing_and_Presentation
IFNA16	3449	interferon a	IFN-alpha-	9	Antigen_Processing_and_Presentation
IFNA17	3451	interferon a	IFN-alphaI	9	Antigen_Processing_and_Presentation
IFNA21	3452	interferon a	IFN-alphaI	9	Antigen_Processing_and_Presentation
IFNG	3458	interferon g	IFG|IFI	12	Antigen_Processing_and_Presentation
KIR2DL1	3802	killer cell ir	CD158A|KI	19	Antigen_Processing_and_Presentation
KIR2DL2	3803	killer cell ir	CD158B1|C	19	Antigen_Processing_and_Presentation
KIR2DL3	3804	killer cell ir	CD158B2|C	19	Antigen_Processing_and_Presentation
KIR2DL4	3805	killer cell ir	CD158D|G	19	Antigen_Processing_and_Presentation
KIR2DS1	3806	killer cell ir	CD158H|CI	19	Antigen_Processing_and_Presentation
KIR2DS3	3808	killer cell ir	NKAT7	19	Antigen_Processing_and_Presentation
KIR2DS4	3809	killer cell ir	CD158I|KIR	19	Antigen_Processing_and_Presentation
KIR2DS5	3810	killer cell ir	CD158G|N	19	Antigen_Processing_and_Presentation
KIR3DL1	3811	killer cell ir	CD158E1|K	19	Antigen_Processing_and_Presentation
KIR3DL2	3812	killer cell ir	3DL2|CD15	19	Antigen_Processing_and_Presentation
KLRC1	3821	killer cell le	CD159A|N	12	Antigen_Processing_and_Presentation
KLRC2	3822	killer cell le	CD159c|NK	12	Antigen_Processing_and_Presentation
KLRC3	3823	killer cell le	NKG2-E|NI	12	Antigen_Processing_and_Presentation
KLRD1	3824	killer cell le	CD94	12	Antigen_Processing_and_Presentation
LTA	4049	lymphotox	LT|TNFB|TN	6	Antigen_Processing_and_Presentation
CIITA	4261	class II maj	C2TA|CIITA	16	Antigen_Processing_and_Presentation
MICA	1.01E+08	MHC class	MIC-A|PER	6	Antigen_Processing_and_Presentation
MICB	4277	MHC class	PERB11.2	6	Antigen_Processing_and_Presentation
NFYA	4800	nuclear tra	CBF-A|CBF	6	Antigen_Processing_and_Presentation
NFYB	4801	nuclear tra	CBF-A|CBF	12	Antigen_Processing_and_Presentation
NFYC	4802	nuclear tra	CBF-C|CBF	1	Antigen_Processing_and_Presentation
LGMN	5641	legumain	AEP|LGMN	14	Antigen_Processing_and_Presentation
PSMB8	5696	proteasom	ALDD|D6S	6	Antigen_Processing_and_Presentation
PSMC1	5700	proteasom	P26S4|S4|p	14	Antigen_Processing_and_Presentation
PSMC2	5701	proteasom	MSS1|Nbla	7	Antigen_Processing_and_Presentation
PSMC3	5702	proteasom	TBP1	11	Antigen_Processing_and_Presentation
PSMC4	5704	proteasom	MIP224|RP	19	Antigen_Processing_and_Presentation
PSMC5	5705	proteasom	S8|SUG-1|S	17	Antigen_Processing_and_Presentation
PSMC6	5706	proteasom	SUG2|p42	14	Antigen_Processing_and_Presentation
PSMD1	5707	proteasom	P112|Rpn2	2	Antigen_Processing_and_Presentation
PSMD2	5708	proteasom	P97|RPN1|S	3	Antigen_Processing_and_Presentation
PSMD3	5709	proteasom	P58|RPN3|S	17	Antigen_Processing_and_Presentation
PSMD4	5710	proteasom	AF|AF-1|AS	1	Antigen_Processing_and_Presentation
PSMD5	5711	proteasom	S5B	9	Antigen_Processing_and_Presentation
PSMD7	5713	proteasom	MOV34|P4	16	Antigen_Processing_and_Presentation
PSMD8	5714	proteasom	HEL-S-91n	19	Antigen_Processing_and_Presentation
PSMD10	5716	proteasom	dJ889N15.2	X	Antigen_Processing_and_Presentation
PSMD11	5717	proteasom	Rpn6|S9|p4	17	Antigen_Processing_and_Presentation
PSMD13	5719	proteasom	HSPC027|R	11	Antigen_Processing_and_Presentation
PSME1	5720	proteasom	HEL-S-129	14	Antigen_Processing_and_Presentation
PSME1	5720	proteasom	HEL-S-129	14	Antigen_Processing_and_Presentation
PSME2	5721	proteasom	PA28B|PA2	14	Antigen_Processing_and_Presentation
PSME2	5721	proteasom	PA28B|PA2	14	Antigen_Processing_and_Presentation
RELB	5971	RELB proto	I-REL|IMD5	19	Antigen_Processing_and_Presentation
RFX5	5993	regulatory	-	1	Antigen_Processing_and_Presentation
RFXAP	5994	regulatory	-	13	Antigen_Processing_and_Presentation

SLC10A2	6555	solute carr	ASBT|IBAT|	13	Antigen_Processing_and_Presentation
TAP1	6890	transporte	ABC17|AB(	6	Antigen_Processing_and_Presentation
TAP2	6891	transporte	ABC18|AB(	6	Antigen_Processing_and_Presentation
TAPBP	6892	TAP bindir	NGS17|TAI	6	Antigen_Processing_and_Presentation
THBS1	7057	thrombosp	THBS|THBS	15	Antigen_Processing_and_Presentation
SEM1	7979	SEM1 26S	C7orf76|DS	7	Antigen_Processing_and_Presentation
KLRC4	8302	killer cell le	NKG2-F|NI	12	Antigen_Processing_and_Presentation
AP3B1	8546	adaptor re	ADTB3|AD	5	Antigen_Processing_and_Presentation
RFXANK	8625	regulatory	ANKRA1|B	19	Antigen_Processing_and_Presentation
PSMD6	9861	proteasom	Rpn7|S10|S	3	Antigen_Processing_and_Presentation
PSME3	10197	proteasom	HEL-S-283	17	Antigen_Processing_and_Presentation
PSMD14	10213	proteasom	PAD1|POH	2	Antigen_Processing_and_Presentation
CLEC4M	10332	C-type lec	CD209L|CD	19	Antigen_Processing_and_Presentation
IFI30	10437	IFI30 lysos	GILT|IFI-30	19	Antigen_Processing_and_Presentation
PROCR	10544	protein C r	CCCA|CCD	20	Antigen_Processing_and_Presentation
ADRM1	11047	adhesion r	ARM-1|AR	20	Antigen_Processing_and_Presentation
ECPAS	23392	Ecm29 pro	ECM29|KIA	9	Antigen_Processing_and_Presentation
TRPC4AP	26133	transient re	C20orf188|	20	Antigen_Processing_and_Presentation
CD209	30835	CD209 mo	CDSIGN|CI	19	Antigen_Processing_and_Presentation
UBXN1	51035	UBX doma	2B28|SAKS	11	Antigen_Processing_and_Presentation
ERAP1	51752	endoplasm	A-LAP|ALA	5	Antigen_Processing_and_Presentation
TAPBPL	55080	TAP bindir	TAPBP-R|T	12	Antigen_Processing_and_Presentation
KIR2DL5A	57292	killer cell ir	CD158F|KII	19	Antigen_Processing_and_Presentation
ERAP2	64167	endoplasm	L-RAP|LRA	5	Antigen_Processing_and_Presentation
ULBP3	79465	UL16 bindi	N2DL-3|NI	6	Antigen_Processing_and_Presentation
ULBP2	80328	UL16 bindi	ALCAN-alp	6	Antigen_Processing_and_Presentation
ULBP1	80329	UL16 bindi	N2DL-1|NI	6	Antigen_Processing_and_Presentation
KIR3DL3	115653	killer cell ir	CD158Z|KI	19	Antigen_Processing_and_Presentation
RAET1E	135250	retinoic aci	LETAL|N2D	6	Antigen_Processing_and_Presentation
RAET1L	154064	retinoic aci	ULBP6	6	Antigen_Processing_and_Presentation
UBR1	197131	ubiquitin p	JBS	15	Antigen_Processing_and_Presentation
RAET1G	353091	retinoic aci	ULBP5	6	Antigen_Processing_and_Presentation
PDIA2	64714	protein dis	PDA2|PDI|I	16	Antigen_Processing_and_Presentation
HAMP	57817	hepcidin a	HEPC|HFE2	19	Antimicrobials
PI3	5266	peptidase i	ESI|SKALP|	20	Antimicrobials
CAMP	820	cathelicidir	CAP-18|CA	3	Antimicrobials
DEFB4A	1673	defensin b	BD-2|DEFE	8	Antimicrobials
PPBP	5473	pro-platele	B-TG1|Beta	4	Antimicrobials
REG3G	130120	regenerati	LPPM429|F	2	Antimicrobials
CXCL14	9547	C-X-C mo	BMAC|BRA	5	Antimicrobials
CXCL16	58191	C-X-C mo	CXCLG16|S	17	Antimicrobials
SLPI	6590	secretory le	ALK1|ALP|E	20	Antimicrobials
CXCL8	3576	C-X-C mo	GCP-1|GCI	4	Antimicrobials
CXCL10	3627	C-X-C mo	C7|IFI10|IN	4	Antimicrobials
CXCL9	4283	C-X-C mo	CMK|Humi	4	Antimicrobials
CXCL5	6374	C-X-C mo	ENA-78|SC	4	Antimicrobials
CXCL11	6373	C-X-C mo	H174|I-TA(	4	Antimicrobials
CXCL6	6372	C-X-C mo	CKA-3|GCI	4	Antimicrobials
CXCL1	2919	C-X-C mo	FSP|GRO1|	4	Antimicrobials
CXCL12	6387	C-X-C mo	IRH|PBSF|S	10	Antimicrobials
CXCL13	10563	C-X-C mo	ANGIE|AN(	4	Antimicrobials
CXCL2	2920	C-X-C mo	CINC-2a|G	4	Antimicrobials
PF4	5196	platelet fac	CXCL4|PF-	4	Antimicrobials
XCL1	6375	X-C motif	ATAC|LPTN	1	Antimicrobials
CXCL3	2921	C-X-C mo	CINC-2b|G	4	Antimicrobials
DEFB103B	55894	defensin b	BD-3|DEFE	8	Antimicrobials
CCL13	6357	C-C motif	CKb10|MC	17	Antimicrobials
CCL1	6346	C-C motif	I-309|P500	17	Antimicrobials

DEFB1	1672	defensin b	BD1|DEFB-	8	Antimicrobials
CCL8	6355	C-C motif	HC14|MCP	17	Antimicrobials
ELANE	1991	elastase, n	ELA2|GE|H	19	Antimicrobials
DEFB103A	414325	defensin b	BD-3|DEFB	8	Antimicrobials
DEFA3	1668	defensin al	DEF3|HNP-	8	Antimicrobials
DEFA1	1667	defensin al	DEF1|DEFA	8	Antimicrobials
TMSB10	9168	thymosin b	MIG12|TB1	2	Antimicrobials
DEFA6	1671	defensin al	DEF6|HD-6	8	Antimicrobials
DEFA5	1670	defensin al	DEF5|HD-5	8	Antimicrobials
DEFA4	1669	defensin al	DEF4|HNP-	8	Antimicrobials
LCN2	3934	lipocalin 2	24p3|MSFI	9	Antimicrobials
LCN1	3933	lipocalin 1	PMFA|TLC|	9	Antimicrobials
COLEC10	10584	collectin su	3MC3|CL-3	8	Antimicrobials
BPI	671	bactericida	BPIFD1|rBP	20	Antimicrobials
S100A9	6280	S100 calciu	60B8AG|CA	1	Antimicrobials
S100A8	6279	S100 calciu	60B8AG|CA	1	Antimicrobials
DCD	117159	dermcidin	AIDD|DCD	12	Antimicrobials
LCN6	158062	lipocalin 6	LCN5|UNQ	9	Antimicrobials
S100A12	6283	S100 calciu	CAAF1|CAG	1	Antimicrobials
HTN3	3347	histatin 3	HIS2|HTN2	4	Antimicrobials
LCN8	138307	lipocalin 8	EP17|LCN5	9	Antimicrobials
DEFA1B	728358	defensin al	HNP-1|HP	8	Antimicrobials
CCR10	2826	C-C motif	GPR2	17	Antimicrobials
CELA1	1990	chymotryp	ELA1	12	Antimicrobials
DEFB106A	245909	defensin b	BD-6|DEFB	8	Antimicrobials
PENK	5179	proenkeph	PE|PENK-A	8	Antimicrobials
BPIFC	254240	BPI fold co	BPIL2	22	Antimicrobials
MMP12	4321	matrix met	HME|ME|M	11	Antimicrobials
BPIFB6	128859	BPI fold co	BPIL3|LPLU	20	Antimicrobials
LEAP2	116842	liver enrich	LEAP-2	5	Antimicrobials
SFTPD	6441	surfactant	COLEC7|PS	10	Antimicrobials
LCN9	392399	lipocalin 9	HEL129	9	Antimicrobials
BPIFB2	80341	BPI fold co	BPIL1|C20o	20	Antimicrobials
PTGDS	5730	prostaglan	L-PGDS|LP	9	Antimicrobials
TMSB4X	7114	thymosin b	FX|PTMB4|	X	Antimicrobials
PGLYRP1	8993	peptidogly	PGLYRP|PG	19	Antimicrobials
ZC3HAV1	56829	zinc finger	ARTD13|FL	7	Antimicrobials
TMSB15A	11013	thymosin b	TMSB15|TM	X	Antimicrobials
S100B	6285	S100 calciu	NEF|S100|S	21	Antimicrobials
S100A13	6284	S100 calciu	-	1	Antimicrobials
S100A6	6277	S100 calciu	2A9|5B10|C	1	Antimicrobials
DEFB119	245932	defensin b	DEFB-19|D	20	Antimicrobials
DEFB107A	245910	defensin b	BD-7|DEFB	8	Antimicrobials
DEFB105A	245908	defensin b	BD-5|DEFB	8	Antimicrobials
SERPIND1	3053	serpin fam	D22S673|H	22	Antimicrobials
DEFB129	140881	defensin b	C20orf87|D	20	Antimicrobials
DEFB127	140850	defensin b	C20orf73|D	20	Antimicrobials
S100P	6286	S100 calciu	MIG9	4	Antimicrobials
S100A7	6278	S100 calciu	PSOR1|S10	1	Antimicrobials
DEFB104A	140596	defensin b	BD-4|DEFB	8	Antimicrobials
DEFB126	81623	defensin b	C20orf8|DE	20	Antimicrobials
DEFB106B	503841	defensin b	BD-6|DEFB	8	Antimicrobials
DEFB104B	503618	defensin b	BD-4|DEFB	8	Antimicrobials
DEFB107B	503614	defensin b	HsT21816	8	Antimicrobials
PGLYRP3	114771	peptidogly	PGLYRPIalp	1	Antimicrobials
PGLYRP2	114770	peptidogly	HMFT0141	19	Antimicrobials
S100A10	6281	S100 calciu	42C|ANX2L	1	Antimicrobials
S100A2	6273	S100 calciu	CAN19|S10	1	Antimicrobials

DEFB125	245938	defensin b	DEFB-25	20	Antimicrobials
DEFB123	245936	defensin b	DEFB-23|D	20	Antimicrobials
DEFB105B	504180	defensin b	BD-5|DEFE	8	Antimicrobials
DEFB132	400830	defensin b	BD-32|DEF	20	Antimicrobials
BPIFB3	359710	BPI fold co	C20orf185|	20	Antimicrobials
LCN12	286256	lipocalin 1	-	9	Antimicrobials
PGLYRP4	57115	peptidogly	PGLYRPIbe	1	Antimicrobials
S100A11	6282	S100 calciu	HEL-S-43|	1	Antimicrobials
S100A5	6276	S100 calciu	S100D	1	Antimicrobials
S100A3	6274	S100 calciu	S100E	1	Antimicrobials
S100A1	6271	S100 calciu	S100|S100	1	Antimicrobials
DEFB128	245939	defensin b	DEFB-28|D	20	Antimicrobials
DEFB108B	245911	defensin b	DEFB-8|hB	11	Antimicrobials
HTN1	3346	histatin 1	HIS1	4	Antimicrobials
LMBR1L	55716	limb devel	LIMR	12	Antimicrobials
S100A7A	338324	S100 calciu	NICE-2|NI	1	Antimicrobials
DEFB118	117285	defensin b	C20orf63|D	20	Antimicrobials
COLEC12	81035	collectin su	CLP1|NSR2	18	Antimicrobials
TMSB4Y	9087	thymosin b	TB4Y	Y	Antimicrobials
DEFB131A	644414	defensin b	DEFB-31|D	4	Antimicrobials
DEFB134	613211	defensin b	-	8	Antimicrobials
DEFB130A	245940	defensin b	DEFB-30|D	8	Antimicrobials
DEFB124	245937	defensin b	DEFB-24	20	Antimicrobials
DEFB121	245934	defensin b	DEFB21|ES	20	Antimicrobials
DEFB116	245930	defensin b	DEFB-16	20	Antimicrobials
DEFB115	245929	defensin b	DEFB-15	20	Antimicrobials
DEFB114	245928	defensin b	DEFB-14|D	6	Antimicrobials
DEFB113	245927	defensin b	DEFB-13	6	Antimicrobials
DEFB112	245915	defensin b	DEFB-12	6	Antimicrobials
DEFB110	245913	defensin b	DEFB-10|D	6	Antimicrobials
TMSB15B	286527	thymosin b	TMSB15A|T	X	Antimicrobials
DEFB133	403339	defensin b	-	6	Antimicrobials
S100Z	170591	S100 calciu	Gm625|S1	5	Antimicrobials
MAVS	57506	mitochond	CARDIF|IPS	20	Antimicrobials
TMSB4XP8	7117	TMSB4X ps	TMSL3	4	Antimicrobials
S100A14	57402	S100 calciu	BCMP84|S	1	Antimicrobials
LCN10	414332	lipocalin 1	-	9	Antimicrobials
S100A16	140576	S100 calciu	AAG13|DT	1	Antimicrobials
DEFB136	613210	defensin b	DEFB137	8	Antimicrobials
DEFB135	613209	defensin b	DEFB136	8	Antimicrobials
DEFB117	245931	defensin b	DEFB-17	20	Antimicrobials
DEFB110	245913	defensin b	DEFB-10|D	6	Antimicrobials
ZC3HAV1L	92092	zinc finger	C7orf39	7	Antimicrobials
S100A7L2	645922	S100 calciu	S100a7b	1	Antimicrobials
MBL3P	50639	mannose-l	COLEC2|M	10	Antimicrobials
DEFB4B	1E+08	defensin b	DEFB4P	8	Antimicrobials
BPIFB4	149954	BPI fold co	C20orf186|	20	Antimicrobials
IFNAR1	3454	interferon	AVP|IFN-a	21	Antimicrobials
AZU1	566	azurocidin	AZAMP|AZ	19	Antimicrobials
DEFB131B	1E+08	defensin b	-	11	Antimicrobials
DEFA1A3	613253	defensin al	DEFA1|DEF	8	Antimicrobials
LCN1P1	286310	lipocalin 1	LCN1L1|bA	9	Antimicrobials
S100G	795	S100 calciu	CABP|CABI	X	Antimicrobials
DEFA7P	724067	defensin al	DEFA7	8	Antimicrobials
DEFB130B	1E+08	defensin b	-	8	Antimicrobials
DEFB108F	1E+08	defensin b	DEFB108P	4	Antimicrobials
DEFB131C	1E+08	defensin b	-	8	Antimicrobials
TCHHL1	126637	trichohyali	S100A17|T	1	Antimicrobials

TINAGL1	64129	tubulointe	ARG1|LCN	1	Antimicrobials
IFNGR1	3459	interferon	CD119|IFN	6	Antimicrobials
SLC22A17	51310	solute carr	24p3R|BO	14	Antimicrobials
WFIKKN1	117166	WAP, follis	C16orf12|F	16	Antimicrobials
WFDC2	10406	WAP four-	EDDM4|HE	20	Antimicrobials
IL6	3569	interleukin	BSF-2|BSF2	7	Antimicrobials
UMODL1	89766	uromoduli	-	21	Antimicrobials
TGFB1	7040	transformi	CED|DPD1	19	Antimicrobials
PF4V1	5197	platelet fac	CXCL4L1|C	4	Antimicrobials
MMP9	4318	matrix met	CLG4B|GEL	20	Antimicrobials
ANOS1	3730	anosmin 1	ADMLX|HHX		Antimicrobials
TLR4	7099	toll like rec	ARMD10|C	9	Antimicrobials
IFNG	3458	interferon	IFG|IFI	12	Antimicrobials
SPAG11B	10407	sperm assc	EDDM2B|E	8	Antimicrobials
A2M	2	alpha-2-m	A2MD|CPA	12	Antimicrobials
CTSL	1514	cathepsin I	CATL|CTSL	9	Antimicrobials
NFKB1	4790	nuclear fac	CVID12|EB	4	Antimicrobials
APOBEC3G	60489	apolipopro	A3G|ARCD	22	Antimicrobials
FABP6	2172	fatty acid b	I-15P|I-BA	5	Antimicrobials
NOD2	64127	nucleotide	ACUG|BLA	16	Antimicrobials
MBL2	4153	mannose b	COLEC1|HS	10	Antimicrobials
SFTPA1	653509	surfactant	COLEC4|PS	10	Antimicrobials
RBP1	5947	retinol bin	CRABP-I|C	3	Antimicrobials
TLR2	7097	toll like rec	CD282|TIL4	4	Antimicrobials
SLC40A1	30061	solute carr	FPN1|HFE4	2	Antimicrobials
PLAU	5328	plasminog	ATF|BDPLT	10	Antimicrobials
IL1B	3553	interleukin	IL-1|IL1-BE	2	Antimicrobials
PAEP	5047	progestage	GD|GdA|Gc	9	Antimicrobials
HJV	148738	hemojuveli	HFE2|HFE2	1	Antimicrobials
MUC5AC	4586	mucin 5AC	MUC5|TBM	11	Antimicrobials
CTSS	1520	cathepsin S	-	1	Antimicrobials
OBP2A	29991	odorant bi	LCN13|OBI	9	Antimicrobials
PLTP	5360	phospholip	BPIFE|HDL	20	Antimicrobials
MX1	4599	MX dynam	IFI-78K|IFI7	21	Antimicrobials
DDX58	23586	DExD/H-b	RIG-I|RIG1	9	Antimicrobials
IFNL1	282618	interferon	IL-29|IL29	19	Antimicrobials
IRF3	3661	interferon	IIAE7	19	Antimicrobials
SFTPA2	729238	surfactant	COLEC5|PS	10	Antimicrobials
LPA	4018	lipoprotein	AK38|APO/	6	Antimicrobials
LBP	3929	lipopolysa	BPIFD2	20	Antimicrobials
RBP4	5950	retinol bin	MCOPCB1	10	Antimicrobials
SFTPA1	653509	surfactant	COLEC4|PS	10	Antimicrobials
NOX4	50507	NADPH ox	KOX|KOX-	11	Antimicrobials
LTF	4057	lactotransf	GIG12|HEL	3	Antimicrobials
IFNB1	3456	interferon	IFB|IFF|IFN	9	Antimicrobials
RBP5	83758	retinol bin	CRBP-III|CI	12	Antimicrobials
FABP7	2173	fatty acid b	B-FABP|BL	6	Antimicrobials
FABP5	2171	fatty acid b	E-FABP|EF/	8	Antimicrobials
FABP3	2170	fatty acid b	FABP11|H-	1	Antimicrobials
FABP2	2169	fatty acid b	FABPI|I-FA	4	Antimicrobials
FABP4	2167	fatty acid b	A-FABP|AF	8	Antimicrobials
R3HDML	140902	R3H doma	dJ881L22.3	20	Antimicrobials
BPIFA3	128861	BPI fold co	C20orf71|S	20	Antimicrobials
BPIFB1	92747	BPI fold co	C20orf114|	20	Antimicrobials
OASL	8638	2'-5'-oligo	OASL1|OA	12	Antimicrobials
CRABP2	1382	cellular ret	CRABP-II|F	1	Antimicrobials
CRABP1	1381	cellular ret	CRABP|CR/	15	Antimicrobials
RBP7	116362	retinol bin	CRABP4|CF	1	Antimicrobials

DUOX1	53905	dual oxidas	LNOX1|NC	15	Antimicrobials
OBP2B	29989	odorant bi	LCN14|OBI	9	Antimicrobials
RBP2	5948	retinol bin	CRABP-II|C	3	Antimicrobials
LCN15	389812	lipocalin 1!	PRO6093|U	9	Antimicrobials
CETP	1071	cholesteryl	BPIFF|HDL(	16	Antimicrobials
FABP12	646486	fatty acid b	-	8	Antimicrobials
FABP9	646480	fatty acid b	PERF|PERF	8	Antimicrobials
BPIFA1	51297	BPI fold co	LUNX|NAS	20	Antimicrobials
LCNL1	401562	lipocalin lik	-	9	Antimicrobials
C8G	733	compleme	C8C	9	Antimicrobials
SPAG11A	653423	sperm asso	EDDM2A|H	8	Antimicrobials
PI15	51050	peptidase i	CRISP8|P24	8	Antimicrobials
NOX1	27035	NADPH ox	GP91-2|M(	X	Antimicrobials
PMP2	5375	peripheral	CMT1G|FA	8	Antimicrobials
APOD	347	apolipopro	-	3	Antimicrobials
ORM2	5005	orosomuco	AGP-B|AG	9	Antimicrobials
ORM1	5004	orosomuco	AGP-A|AG	9	Antimicrobials
TNF	7124	tumor necı	DIF|TNF-al	6	Antimicrobials
CTSG	1511	cathepsin (	CATG|CG	14	Antimicrobials
PRTN3	5657	proteinase	ACPA|AGP	19	Antimicrobials
MAPK1	5594	mitogen-a	ERK|ERK-2	22	Antimicrobials
PML	5371	PML nucle	MYL|PP867	15	Antimicrobials
AEN	64782	apoptosis (	ISG20L1|pp	15	Antimicrobials
CYBB	1536	cytochrom	AMCBX2|C	X	Antimicrobials
BPIFA2	140683	BPI fold co	C20orf70|F	20	Antimicrobials
ISG20	3669	interferon :	CD25|HEM	15	Antimicrobials
BCL3	602	BCL3 trans	BCL4|D19S	19	Antimicrobials
ISG20L2	81875	interferon :	HSD38	1	Antimicrobials
NOX5	79400	NADPH ox	-	15	Antimicrobials
NOX3	50508	NADPH ox	GP91-3|M(	6	Antimicrobials
DUOX2	50506	dual oxidas	LNOX2|NC	15	Antimicrobials
TLR3	7098	toll like rec	CD283|IIAE	4	Antimicrobials
TFRC	7037	transferrin	CD71|IMD4	3	Antimicrobials
IFIH1	64135	interferon	AGS7|Hlcd	2	Antimicrobials
LRP1	4035	LDL recept	A2MR|APC	12	Antimicrobials
TRIM5	85363	tripartite m	RNF88|TRII	11	Antimicrobials
IDO1	3620	indoleamir	IDO|IDO-1	8	Antimicrobials
GDF15	9518	growth dif	GDF-15|M	19	Antimicrobials
NEDD4	4734	NEDD4 E3	NEDD4-1|I	15	Antimicrobials
ADIPOQ	9370	adiponecti	ACDC|ACR	3	Antimicrobials
STAT3	6774	signal tran:	ADMIO|AD	17	Antimicrobials
STAT1	6772	signal tran:	CANDF7|IN	2	Antimicrobials
IFNL2	282616	interferon	IL-28A|IL28	19	Antimicrobials
SOCS3	9021	suppressor	ATOD4|CIS	17	Antimicrobials
SEMG1	6406	semenogel	CT103|SEM	20	Antimicrobials
TNFSF10	8743	TNF super	APO2L|Apo	3	Antimicrobials
CCL20	6364	C-C motif	CKb4|Exod	2	Antimicrobials
SOCS1	8651	suppressor	CIS1|CISH1	16	Antimicrobials
RNASEL	6041	ribonuclea	PRCA1|RN:	1	Antimicrobials
IRF1	3659	interferon	IRF-1|MAR	5	Antimicrobials
IL15	3600	interleukin	IL-15	4	Antimicrobials
APOBEC3F	200316	apolipopro	A3F|ARP8|I	22	Antimicrobials
PLAAT4	5920	phospholip	HRASLS4|H	11	Antimicrobials
CHIT1	1118	chitinase 1	CHI3|CHIT|	1	Antimicrobials
IFNA1	3439	interferon	IFL|IFN|IFN	9	Antimicrobials
CD40	958	CD40 mole	Bp50|CDW	20	Antimicrobials
TLR7	51284	toll like rec	TLR7-like	X	Antimicrobials
PPIA	5478	peptidylpro	CYPA|CYPI	7	Antimicrobials

HFE	3077	homeostat	HFE1|HH|H		6	Antimicrobials
ZYX	7791	zyxin	ESP-2|HED		7	Antimicrobials
NLRX1	79671	NLR family	CLR11.3|DI		11	Antimicrobials
PGC	5225	progastrics	PEPC|PGII		6	Antimicrobials
VEGFA	7422	vascular er	MVCD1|VE		6	Antimicrobials
IKBKE	9641	inhibitor of	IKK-E|IKK-i		1	Antimicrobials
ISG15	9636	ISG15 ubiq	G1P2|IFI15		1	Antimicrobials
DHX58	79132	DExH-box	D11LGP2|D		17	Antimicrobials
TNFAIP3	7128	TNF alpha	A20|AISBL|		6	Antimicrobials
TFR2	7036	transferrin	HFE3|TFRC		7	Antimicrobials
FCN2	2220	ficolin 2	EBP-37|FC		9	Antimicrobials
MUC4	4585	mucin 4, ce	ASGP|HSA		3	Antimicrobials
F2R	2149	coagulatio	CF2R|HTR|		5	Antimicrobials
ELN	2006	elastin	ADCL1|SVA		7	Antimicrobials
IL27	246778	interleukin	IL-27|IL-27		16	Antimicrobials
MAPT	4137	microtubul	DDPAC|FTI		17	Antimicrobials
LYZ	4069	lysozyme	LYZF1|LZM		12	Antimicrobials
CCL5	6352	C-C motif	D17S136E|		17	Antimicrobials
LEP	3952	leptin	LEPD|OB|C		7	Antimicrobials
CYLD	1540	CYLD lysin	BRSS|CDM		16	Antimicrobials
KLKB1	3818	kallikrein B	KLK3|PKK|F		4	Antimicrobials
CST4	1472	cystatin S	-		20	Antimicrobials
CSRP1	1465	cysteine ar	CRP|CRP1|		1	Antimicrobials
MAPK14	1432	mitogen-a	CSBP|CSBF		6	Antimicrobials
JUN	3725	Jun proto-	AP-1|AP1|		1	Antimicrobials
ITGAV	3685	integrin su	CD51|MSK		2	Antimicrobials
IRF5	3663	interferon	SLEB10		7	Antimicrobials
CCR6	1235	C-C motif	BN-1|C-C		6	Antimicrobials
IL12B	3593	interleukin	CLMF|CLM		5	Antimicrobials
TLR8	51311	toll like rec	CD288	X		Antimicrobials
GNLY	10578	granulysin	D2S69E|LA		2	Antimicrobials
CD81	975	CD81 mole	CVID6|S5.7		11	Antimicrobials
EIF2AK2	5610	eukaryotic	EIF2AK1|LE		2	Antimicrobials
APOM	55937	apolipopro	G3a|HSPC3		6	Antimicrobials
CACYBP	27101	calcyclin bi	GIG5|PNAS		1	Antimicrobials
NOD1	10392	nucleotide	CARD4|CLI		7	Antimicrobials
MAPK8	5599	mitogen-a	JNK|JNK-4		10	Antimicrobials
MAPK3	5595	mitogen-a	ERK-1|ERK		16	Antimicrobials
BST2	684	bone marr	CD317|TET		19	Antimicrobials
BPHL	670	biphenyl h	BPH-RP|M		6	Antimicrobials
PLA2G2A	5320	phospholip	MOM1|PLA		1	Antimicrobials
GRN	2896	granulin pr	CLN11|GEF		17	Antimicrobials
NEWENTRY	192343	-	-	-		Antimicrobials
PDGFRA	5156	platelet de	CD140A|PI		4	Antimicrobials
GNAI1	2770	G protein s	Gi		7	Antimicrobials
WNT5A	7474	Wnt family	hWNT5A		3	Antimicrobials
FURIN	5045	furin, paire	FUR|PACE|		15	Antimicrobials
ADAR	103	adenosine	ADAR1|AG		1	Antimicrobials
TYK2	7297	tyrosine kir	IMD35|JTK		19	Antimicrobials
NOS2	4843	nitric oxide	HEP-NOS|		17	Antimicrobials
TRAF3	7187	TNF recept	CAP-1|CAF		14	Antimicrobials
TPT1	7178	tumor prot	HRF|TCTP|		13	Antimicrobials
TPM2	7169	tropomyos	AMCD1|DA		9	Antimicrobials
NEO1	4756	neogenin 1	IGDCC2|NC		15	Antimicrobials
AHNAK	79026	AHNAK nu	AHNAKRS|		11	Antimicrobials
TLR1	7096	toll like rec	CD281|TIL|		4	Antimicrobials
TK2	7084	thymidine	MTDPS2|M		16	Antimicrobials
PRDX2	7001	peroxiredo	HEL-S-2a|I		19	Antimicrobials

MX2	4600	MX dynam	MXB	21	Antimicrobials
FGF2	2247	fibroblast g	BFGF|FGF-	4	Antimicrobials
FGA	2243	fibrinogen	Fib2	4	Antimicrobials
TCF7L2	6934	transcriptic	TCF-4|TCF	10	Antimicrobials
F2RL1	2150	F2R like try	GPR11|PAF	5	Antimicrobials
TKFC	26007	triokinase a	DAK|NET45	11	Antimicrobials
MSR1	4481	macrophag	CD204|SCA	8	Antimicrobials
NFKBIZ	64332	NFKB inhib	IKBZ|INAP|	3	Antimicrobials
LMBR1	64327	limb devel	ACHP|C7or	7	Antimicrobials
EPPIN	57119	epididyma	CT71|CT72	20	Antimicrobials
SRC	6714	SRC proto-	ASV|SRC1|	20	Antimicrobials
MPO	4353	myelopero	-	17	Antimicrobials
ELAVL1	1994	ELAV like F	ELAV1|HUF	19	Antimicrobials
ROBO3	64221	roundabou	HGPPS|HG	11	Antimicrobials
SP1	6667	Sp1 transc	-	12	Antimicrobials
SOD1	6647	superoxide	ALS|ALS1|H	21	Antimicrobials
PDF	64146	peptide de	-	16	Antimicrobials
DLL4	54567	delta like c	AOS6|delta	15	Antimicrobials
ECD	11319	ecdysonele	GCR2|HSG	10	Antimicrobials
SLC11A1	6556	solute carr	LSH|NRAM	2	Antimicrobials
DMBT1	1755	deleted in	GP340|SAG	10	Antimicrobials
STING1	340061	stimulator	ERIS|MITA|	5	Antimicrobials
SKIV2L	6499	Ski2 like RI	170A|DDX	6	Antimicrobials
SEMG2	6407	semenogel	SGII	20	Antimicrobials
LTA	4049	lymphotox	LT|TNFB|TN	6	Antimicrobials
DES	1674	desmin	CDCD3|CS	2	Antimicrobials
DCK	1633	deoxycytid	-	4	Antimicrobials
DAXX	1616	death dom	BING2|DAF	6	Antimicrobials
TNFRSF10A	8797	TNF recept	APO2|CD2	8	Antimicrobials
TNFRSF10B	8795	TNF recept	CD262|DR5	8	Antimicrobials
EED	8726	embryonic	COGIS|HEE	11	Antimicrobials
CCL4	6351	C-C motif	ACT2|AT74	17	Antimicrobials
LIMS1	3987	LIM zinc fir	PINCH|PIN	2	Antimicrobials
LALBA	3906	lactalbumi	LYZG	12	Antimicrobials
APOBEC3H	164668	apolipopro	A3H|ARP-1	22	Antimicrobials
TMPRSS6	164656	transmemb	IRIDA|MT2	22	Antimicrobials
SPINK5	11005	serine pep	LEKTI|LETK	5	Antimicrobials
MARCO	8685	macrophag	SCARA2|SF	2	Antimicrobials
BECN1	8678	beclin 1	ATG6|VPS3	17	Antimicrobials
TNFSF11	8600	TNF superf	CD254|OD	13	Antimicrobials
KNG1	3827	kininogen	BDK|BK|HM	3	Antimicrobials
CSK	1445	C-terminal	-	15	Antimicrobials
KLRK1	22914	killer cell le	CD314|D12	12	Antimicrobials
KCNH2	3757	potassium	ERG-1|ERG	7	Antimicrobials
JUND	3727	JunD proto	AP-1	19	Antimicrobials
JAK1	3716	Janus kinas	JAK1A|JAK	1	Antimicrobials
CREB1	1385	cAMP resp	CREB|CREE	2	Antimicrobials
CLDN4	1364	claudin 4	CPE-R|CPE	7	Antimicrobials
CCL28	56477	C-C motif	CCK1|MEC	5	Antimicrobials
RNASE3	6037	ribonuclea	ECP|RAF1|F	14	Antimicrobials
RN7SL1	6029	RNA comp	7L1a|7SL|R	14	Antimicrobials
IRF7	3665	interferon	IMD39|IRF-	11	Antimicrobials
IREB2	3658	iron respo	ACO3|IRE-	15	Antimicrobials
ILK	3611	integrin lin	HEL-S-28|	11	Antimicrobials
IL18	3606	interleukin	IGIF|IL-18|	11	Antimicrobials
IL17A	3605	interleukin	CTLA-8|CT	6	Antimicrobials
LTB4R	1241	leukotrien	BLT1|BLTR|	14	Antimicrobials
APOBEC3A	200315	apolipopro	A3A|ARP3|	22	Antimicrobials

MASP2	10747	mannan bi	MAP19|MA	1	Antimicrobials
TRIM27	5987	tripartite m	RFP|RNF76	6	Antimicrobials
RELA	5970	RELA proto	CMCU|NFK	11	Antimicrobials
IL7R	3575	interleukin	CD127|CDW	5	Antimicrobials
IL1A	3552	interleukin	IL-1 alpha|	2	Antimicrobials
PTX3	5806	pentraxin 3	TNFAIP5|TS	3	Antimicrobials
IFNAR2	3455	interferon a	IFN-R|IFN-	21	Antimicrobials
IFN1@	3438	-	IFNA	9	Antimicrobials
SYTL1	84958	synaptotag	JFC1|SLP1	1	Antimicrobials
APOBEC3C	27350	apolipopro	A3C|APOB	22	Antimicrobials
DDX17	10521	DEAD-box	P72|RH70	22	Antimicrobials
PTGS2	5743	prostaglan	COX-2|CO	1	Antimicrobials
HTR1A	3350	5-hydroxyt	5-HT-1A|5	5	Antimicrobials
SEPTIN7	989	septin 7	CDC10|CD	7	Antimicrobials
CD40LG	959	CD40 ligan	CD154|CD4X		Antimicrobials
CD14	929	CD14 mole	-	5	Antimicrobials
CD8A	925	CD8a mole	CD8|Leu2|p	2	Antimicrobials
CD4	920	CD4 molec	CD4mut	12	Antimicrobials
MASP1	5648	mannan bi	3MC1|CRA	3	Antimicrobials
PROC	5624	protein C, i	APC|PC|PR	2	Antimicrobials
MAP2K2	5605	mitogen-a	CFC4|MAP	19	Antimicrobials
MAP2K1	5604	mitogen-a	CFC3|MAP	15	Antimicrobials
HRG	3273	histidine ri	HPRG|HRG	3	Antimicrobials
NDRG1	10397	N-myc dow	CAP43|CM	8	Antimicrobials
IRF9	10379	interferon	IRF-9|ISGF3	14	Antimicrobials
TRIM22	10346	tripartite m	GPSTAF50|	11	Antimicrobials
LANCL1	10314	LanC like 1	GPR69A|p4	2	Antimicrobials
PPP4C	5531	protein ph	PP-X|PP4|P	16	Antimicrobials
HMOX1	3162	heme oxyg	HMOX1D|H	22	Antimicrobials
HMGB1	3146	high mobil	HMG-1|HM	13	Antimicrobials
HLA-B	3106	major histo	AS|B-4901	6	Antimicrobials
RNASE7	84659	ribonuclea	RAE1	14	Antimicrobials
ABCC4	10257	ATP bindin	MOAT-B|M	13	Antimicrobials
HGF	3082	hepatocyte	DFNB39|F-	7	Antimicrobials
HDAC1	3065	histone de	GON-10|H	1	Antimicrobials
IFNLR1	163702	interferon	CRF2/12|IF	1	Antimicrobials
PLSCR1	5359	phospholip	MMTRA1B	3	Antimicrobials
B2M	567	beta-2-mi	IMD43	15	Antimicrobials
BACH2	60468	BTB domai	BTBD25|IM	6	Antimicrobials
TANK	10010	TRAF famil	I-TRAF|ITR	2	Antimicrobials
PIK3CG	5294	phosphatic	PI3CG|PI3K	7	Antimicrobials
ARRB1	408	arrestin be	ARB1|ARR1	11	Antimicrobials
RSAD2	91543	radical S-a	2510004L0	2	Antimicrobials
STAB2	55576	stabilin 2	FEEL2|FELE	12	Antimicrobials
TBK1	29110	TANK bind	FTDALS4|II	12	Antimicrobials
PDYN	5173	prodynorp	ADCA|PEN	20	Antimicrobials
PDGFRB	5159	platelet de	CD140B|IB	5	Antimicrobials
PDCD1	5133	programm	CD279|PD-	2	Antimicrobials
PCSK2	5126	proprotein	NEC 2|NEC	20	Antimicrobials
PCSK1	5122	proprotein	BMIQ12|NI	5	Antimicrobials
ARG2	384	arginase 2	-	14	Antimicrobials
AQP9	366	aquaporin	AQP-9|HsT	15	Antimicrobials
FASLG	356	Fas ligand	ALPS1B|AP	1	Antimicrobials
APOH	350	apolipopro	B2G1|B2GF	17	Antimicrobials
BIRC5	332	baculoviral	API4|EPR-1	17	Antimicrobials
ANXA6	309	annexin A6	ANX6|CBP6	5	Antimicrobials
IL22	50616	interleukin	IL-21|IL-22	12	Antimicrobials
VTN	7448	vitronectin	V75|VN|VN	17	Antimicrobials

VIM	7431	vimentin	-	10 Antimicrobials
VCAM1	7412	vascular ce	CD106|INC	1 Antimicrobials
PRDX1	5052	peroxiredo	MSP23|NK	1 Antimicrobials
GFAP	2670	glial fibrilla	ALXDRD	17 Antimicrobials
GBP2	2634	guanylate	-	1 Antimicrobials
ALB	213	albumin	HSA|PRO0	4 Antimicrobials
SLC29A3	55315	solute carr	ENT3|HCL	10 Antimicrobials
OAS1	4938	2'-5'-oligo	E18/E16|IFI	12 Antimicrobials
AGER	177	advanced	RAGE|SCAI	6 Antimicrobials
UNC93B1	81622	unc-93 ho	IIAE1|UNC	11 Antimicrobials
TNFSF4	7292	TNF super	CD134L|CD	1 Antimicrobials
NOS1	4842	nitric oxide	IHPS1|N-N	12 Antimicrobials
ACTG1	71	actin gamr	ACT|ACTG	17 Antimicrobials
ACTA1	58	actin alpha	ACTA|ASM	1 Antimicrobials
ACO1	48	aconitase	ACONS|HE	9 Antimicrobials
SERPINA3	12	serpin fam	AACT|ACT|	14 Antimicrobials
CXCR1	3577	C-X-C mo	C-C|C-C-	2 Antimicrobials
CCL15	6359	C-C motif	HCC-2|HM	17 Antimicrobials
CCL14	6358	C-C motif	CC-1|CC-3	17 Antimicrobials
CCL4	6351	C-C motif	ACT2|AT74	17 Antimicrobials
CCL16	6360	C-C motif	CKb12|HC	17 Antimicrobials
CCL19	6363	C-C motif	CKb11|ELC	9 Antimicrobials
CCL13	6357	C-C motif	CKb10|MC	17 Antimicrobials
CCL18	6362	C-C motif	AMAC-1|A	17 Antimicrobials
CCL17	6361	C-C motif	A-152E5.3	16 Antimicrobials
CCL26	10344	C-C motif	IMAC|MIP-	7 Antimicrobials
CCL22	6367	C-C motif	A-152E5.1	16 Antimicrobials
CCR3	1232	C-C motif	C C CKR3|	3 Antimicrobials
CCL28	56477	C-C motif	CCK1|MEC	5 Antimicrobials
CCL4L1	388372	C-C motif	AT744.2|C	17 Antimicrobials
ACKR2	1238	atypical ch	CCBP2|CC	3 Antimicrobials
CCR7	1236	C-C motif	BLR2|CC-C	17 Antimicrobials
CCL27	10850	C-C motif	ALP|CTACK	9 Antimicrobials
CCR8	1237	C-C motif	CC-CKR-8	3 Antimicrobials
ACKR4	51554	atypical ch	CC-CKR-1	3 Antimicrobials
CCR10	2826	C-C motif	GPR2	17 Antimicrobials
CCL2	6347	C-C motif	GDCF-2|H	17 Antimicrobials
CCL21	6366	C-C motif	6Ckine|CK	9 Antimicrobials
CCL7	6354	C-C motif	FIC|MARC|	17 Antimicrobials
CCL5	6352	C-C motif	D17S136E|	17 Antimicrobials
CCL3	6348	C-C motif	G0S19-1|L	17 Antimicrobials
CCL20	6364	C-C motif	CKb4|Exod	2 Antimicrobials
CCL11	6356	C-C motif	SCYA11	17 Antimicrobials
CCR5	1234	C-C motif	CC-CKR-5	3 Antimicrobials
CCL23	6368	C-C motif	CK-BETA-	17 Antimicrobials
CCL25	6370	C-C motif	Ckb15|SCY	19 Antimicrobials
CCL1	6346	C-C motif	I-309|P500	17 Antimicrobials
CCL3L3	414062	C-C motif	464.2|D17	17 Antimicrobials
CCL4L2	9560	C-C motif	AT744.2|C	17 Antimicrobials
CXCL12	6387	C-X-C mo	IRH|PBSF|S	10 Antimicrobials
XCL1	6375	X-C motif	ATAC|LPTN	1 Antimicrobials
CCL8	6355	C-C motif	HC14|MCP	17 Antimicrobials
CCL3L1	6349	C-C motif	464.2|D17	17 Antimicrobials
CCR1	1230	C-C motif	CD191|CK	3 Antimicrobials
CCL24	6369	C-C motif	Ckb-6|MPI	7 Antimicrobials
XCL2	6846	X-C motif	SCM-1b|S	1 Antimicrobials
CXCL1	2919	C-X-C mo	FSP|GRO1|	4 Antimicrobials
CXCL10	3627	C-X-C mo	C7|IFI10|IN	4 Antimicrobials

CXCR4	7852	C-X-C mo	CD184|D2S	2	Antimicrobials
CXCL2	2920	C-X-C mo	CINC-2a|G	4	Antimicrobials
CXCR6	10663	C-X-C mo	BONZO|CD	3	Antimicrobials
CCR4	1233	C-C motif	CC-CKR-4	3	Antimicrobials
CXCL11	6373	C-X-C mo	H174|I-TA	4	Antimicrobials
TAFA5	25817	TAFA chen	FAM19A5|	22	Antimicrobials
TAFA3	284467	TAFA chen	FAM19A3|	1	Antimicrobials
TAFA4	151647	TAFA chen	FAM19A4|	3	Antimicrobials
TAFA1	407738	TAFA chen	FAM19A1|	3	Antimicrobials
TAFA2	338811	TAFA chen	FAM19A2|	12	Antimicrobials
CCL15-CC	348249	CCL15-CC	CCL15|HC	17	Antimicrobials
IL6	3569	interleukin	BSF-2|BSF2	7	Antimicrobials
TNF	7124	tumor neci	DIF|TNF-al	6	Antimicrobials
IL1B	3553	interleukin	IL-1|IL1-BE	2	Antimicrobials
IL18	3606	interleukin	IGIF|IL-18|I	11	Antimicrobials
PTK2B	2185	protein tyr	CADTK|CA	8	Antimicrobials
VEGFA	7422	vascular er	MVCD1|VE	6	Antimicrobials
IL4	3565	interleukin	BCGF-1|BC	5	Antimicrobials
CDH1	999	cadherin 1	Arc-1|BCD	16	Antimicrobials
CD40	958	CD40 mole	Bp50|CDW	20	Antimicrobials
DEFB103B	55894	defensin b	BD-3|DEFE	8	Antimicrobials
F2RL1	2150	F2R like try	GPR11|PAF	5	Antimicrobials
MMP9	4318	matrix met	CLG4B|GEL	20	Antimicrobials
LTBP1	4052	latent trans	-	2	Antimicrobials
DEFB4A	1673	defensin b	BD-2|DEFE	8	Antimicrobials
TNFSF10	8743	TNF super	APO2L|Apo	3	Antimicrobials
IL13	3596	interleukin	IL-13|P600	5	Antimicrobials
IL10	3586	interleukin	CSIF|GVHD	1	Antimicrobials
IL2	3558	interleukin	IL-2|TCGF|	4	Antimicrobials
PPARG	5468	peroxisom	CIMT1|GLN	3	Antimicrobials
FGR	2268	FGR proto-	SRC2|c-fgr	1	Antimicrobials
MIF	4282	macrophag	GIF|GLIF|M	22	Antimicrobials
CRP	1401	C-reactive	PTX1	1	Antimicrobials
JAK2	3717	Janus kinas	JTK10|THC	9	Antimicrobials
IL1A	3552	interleukin	IL-1 alpha|	2	Antimicrobials
PTK2	5747	protein tyr	FADK|FAK|	8	Antimicrobials
PTGDR	5729	prostaglan	AS1|ASRT1	14	Antimicrobials
CD86	942	CD86 mole	B7-2|B7.2|	3	Antimicrobials
HCK	3055	HCK proto	JTK9|p59H	20	Antimicrobials
ARRB1	408	arrestin be	ARB1|ARR1	11	Antimicrobials
GNAI1	2770	G protein s	Gi	7	Antimicrobials
VDR	7421	vitamin D r	NR1I1|PPP	12	Antimicrobials
OLR1	4973	oxidized lo	CLEC8A|LC	12	Antimicrobials
GRK2	156	G protein-	ADRBK1|BA	11	Antimicrobials
TXK	7294	TXK tyrosir	BTKL|PSCT	4	Antimicrobials
RNASE2	6036	ribonuclea	EDN|RAF3|	14	Antimicrobials
CD79A	973	CD79a mo	IGA|MB-1	19	BCRSignalingPathway
CD79B	974	CD79b mo	AGM6|B29	17	BCRSignalingPathway
LYN	4067	LYN proto-	JTK8|p53Ly	8	BCRSignalingPathway
SYK	6850	spleen ass	p72-Syk	9	BCRSignalingPathway
BTK	695	Bruton tyro	AGMX1|ATX		BCRSignalingPathway
BLNK	29760	B cell linke	AGM4|BAS	10	BCRSignalingPathway
VAV3	10451	vav guanin	-	1	BCRSignalingPathway
VAV1	7409	vav guanin	VAV	19	BCRSignalingPathway
VAV2	7410	vav guanin	VAV-2	9	BCRSignalingPathway
RAC1	5879	Rac family	MIG5|MRD	7	BCRSignalingPathway
RAC2	5880	Rac family	EN-7|Gx|H	22	BCRSignalingPathway
RAC3	5881	Rac family	-	17	BCRSignalingPathway

PPP3CA	5530	protein ph	ACCIID|CA	4	BCRSignalingPathway
PPP3CB	5532	protein ph	CALNA2|C	10	BCRSignalingPathway
PPP3CC	5533	protein ph	CALNA3|C	8	BCRSignalingPathway
CHP1	11261	calcineurin	CHP|SLC9A	15	BCRSignalingPathway
PPP3R1	5534	protein ph	CALNB1|CI	2	BCRSignalingPathway
PPP3R2	5535	protein ph	PPP3RL	9	BCRSignalingPathway
CHP2	63928	calcineurin	-	16	BCRSignalingPathway
NFAT5	10725	nuclear fac	NF-AT5|NI	16	BCRSignalingPathway
NFATC1	4772	nuclear fac	NF-ATC|NI	18	BCRSignalingPathway
NFATC2	4773	nuclear fac	NFAT1|NF/	20	BCRSignalingPathway
NFATC3	4775	nuclear fac	NF-AT4c|N	16	BCRSignalingPathway
NFATC4	4776	nuclear fac	NF-AT3|NI	14	BCRSignalingPathway
HRAS	3265	HRas proto	C-BAS/HA	11	BCRSignalingPathway
KRAS	3845	KRAS proto	'C-K-RAS|(	12	BCRSignalingPathway
NRAS	4893	NRAS prot	ALPS4|CMI	1	BCRSignalingPathway
FOS	2353	Fos proto-	AP-1|C-FC	14	BCRSignalingPathway
JUN	3725	Jun proto-	AP-1|AP1|(	1	BCRSignalingPathway
CARD11	84433	caspase re	BENTA|BIM	7	BCRSignalingPathway
BCL10	8915	BCL10 imm	CARMEN|C	1	BCRSignalingPathway
MALT1	10892	MALT1 par	IMD12|ML	18	BCRSignalingPathway
CHUK	1147	componen	IKBKA|IKK-	10	BCRSignalingPathway
IKBKB	3551	inhibitor of	IKK-beta|Ik	8	BCRSignalingPathway
IKBKG	8517	inhibitor of	AMCBX1|E	X	BCRSignalingPathway
NFKB1	4790	nuclear fac	CVID12|EB	4	BCRSignalingPathway
RELA	5970	RELA proto	CMCU|NFk	11	BCRSignalingPathway
NFKBIA	4792	NFKB inhib	EDAID2|IKE	14	BCRSignalingPathway
NFKBIB	4793	NFKB inhib	IKBB|TRIP9	19	BCRSignalingPathway
NFKBIE	4794	NFKB inhib	IKBE	6	BCRSignalingPathway
CD81	975	CD81 mole	CVID6|S5.7	11	BCRSignalingPathway
CD19	930	CD19 mole	B4|CVID3	16	BCRSignalingPathway
CR2	1380	compleme	C3DR|CD2	1	BCRSignalingPathway
PIK3R5	23533	phosphoin	F730038I1	17	BCRSignalingPathway
PIK3R1	5295	phosphoin	AGM7|GRE	5	BCRSignalingPathway
PIK3R2	5296	phosphoin	MPPH|MPI	19	BCRSignalingPathway
PIK3R3	8503	phosphoin	p55|p55-G	1	BCRSignalingPathway
PIK3CA	5290	phosphatic	CLAPO|CL(	3	BCRSignalingPathway
PIK3CB	5291	phosphatic	P110BETA|	3	BCRSignalingPathway
PIK3CD	5293	phosphatic	APDS|IMD	1	BCRSignalingPathway
PIK3CG	5294	phosphatic	PI3CG|PI3K	7	BCRSignalingPathway
AKT3	10000	AKT serine	MPPH|MPI	1	BCRSignalingPathway
AKT1	207	AKT serine	AKT|CWS6	14	BCRSignalingPathway
AKT2	208	AKT serine	HIHGHH|P	19	BCRSignalingPathway
GSK3B	2932	glycogen s	-	3	BCRSignalingPathway
INPP5D	3635	inositol po	SHIP|SHIP-	2	BCRSignalingPathway
CD22	933	CD22 mole	SIGLEC-2|S	19	BCRSignalingPathway
CD72	971	CD72 mole	CD72b|LYE	9	BCRSignalingPathway
PTPN6	5777	protein tyr	HCP|HCPH	12	BCRSignalingPathway
LILRB3	11025	leukocyte i	CD85A|HL	19	BCRSignalingPathway
FCGR2B	2213	Fc fragmer	CD32|CD3	1	BCRSignalingPathway
RASGRP3	25780	RAS guany	GRP3	2	BCRSignalingPathway
PLCG2	5336	phospholip	APLAID|FC	16	BCRSignalingPathway
PRKCB	5579	protein kin	PKC-beta|I	16	BCRSignalingPathway
IFITM1	8519	interferon	9-27|CD22	11	BCRSignalingPathway
IGH	3492	immunogl	IGD1|IGH.1	14	BCRSignalingPathway
IGHA1	3493	immunogl	IgA1	14	BCRSignalingPathway
IGHA2	3494	immunogl	-	14	BCRSignalingPathway
IGHD	3495	immunogl	-	14	BCRSignalingPathway
IGHD1-1	28510	immunogl	IGHD11	14	BCRSignalingPathway

IGHD1-14	28508	immunogl	DM2|IGHD	14	BCRSignalingPathway
IGHD1-20	28507	immunogl	IGHD120	14	BCRSignalingPathway
IGHD1-26	28506	immunogl	IGHD126	14	BCRSignalingPathway
IGHD1-7	28509	immunogl	DM1|IGHD	14	BCRSignalingPathway
IGHD2-15	28503	immunogl	D2|IGHD21	14	BCRSignalingPathway
IGHD2-2	28505	immunogl	IGHD22	14	BCRSignalingPathway
IGHD2-21	28502	immunogl	IGHD221	14	BCRSignalingPathway
IGHD2-8	28504	immunogl	DLR1|IGHD	14	BCRSignalingPathway
IGHD3-10	28499	immunogl	DXP'1|IGH	14	BCRSignalingPathway
IGHD3-16	28498	immunogl	IGHD316	14	BCRSignalingPathway
IGHD3-22	28497	immunogl	IGHD322	14	BCRSignalingPathway
IGHD3-3	28501	immunogl	DXP4|IGHD	14	BCRSignalingPathway
IGHD3-9	28500	immunogl	DXP1|IGHD	14	BCRSignalingPathway
IGHD4-11	28495	immunogl	DA1|IGHD	14	BCRSignalingPathway
IGHD4-17	28494	immunogl	IGHD417	14	BCRSignalingPathway
IGHD4-23	28493	immunogl	IGHD423	14	BCRSignalingPathway
IGHD4-4	28496	immunogl	DA4|IGHD	14	BCRSignalingPathway
IGHD5-12	28491	immunogl	DK1|IGHD	14	BCRSignalingPathway
IGHD5-18	28490	immunogl	IGHD518	14	BCRSignalingPathway
IGHD5-24	28489	immunogl	IGHD524	14	BCRSignalingPathway
IGHD5-5	28492	immunogl	DK4|IGHD	14	BCRSignalingPathway
IGHD6-13	28487	immunogl	DN1|IGHD	14	BCRSignalingPathway
IGHD6-19	28486	immunogl	IGHD619	14	BCRSignalingPathway
IGHD6-25	28485	immunogl	IGHD625	14	BCRSignalingPathway
IGHD6-6	28488	immunogl	D(N4)|IGH	14	BCRSignalingPathway
IGHD7-27	28484	immunogl	DHQ52|IGH	14	BCRSignalingPathway
IGHE	3497	immunogl	IgE	14	BCRSignalingPathway
IGHG1	3500	immunogl	-	14	BCRSignalingPathway
IGHG2	3501	immunogl	-	14	BCRSignalingPathway
IGHG3	3502	immunogl	IgG3	14	BCRSignalingPathway
IGHG4	3503	immunogl	-	14	BCRSignalingPathway
IGHJ1	28483	immunogl	JH1	14	BCRSignalingPathway
IGHJ2	28481	immunogl	JH2	14	BCRSignalingPathway
IGHJ3	28479	immunogl	JH3b	14	BCRSignalingPathway
IGHJ4	28477	immunogl	JH4b	14	BCRSignalingPathway
IGHJ5	28476	immunogl	JH5b	14	BCRSignalingPathway
IGHJ6	28475	immunogl	JH6b	14	BCRSignalingPathway
IGHM	3507	immunogl	AGM1|MU	14	BCRSignalingPathway
IGH	3492	immunogl	IGD1|IGH.1	14	BCRSignalingPathway
IGHV1-18	28468	immunogl	IGHV118	14	BCRSignalingPathway
IGHV1-2	28474	immunogl	IGHV12|V3	14	BCRSignalingPathway
IGHV1-24	28467	immunogl	IGHV124|V	14	BCRSignalingPathway
IGHV1-3	28473	immunogl	IGHV13|VI-	14	BCRSignalingPathway
IGHV1-45	28466	immunogl	IGHV145|V	14	BCRSignalingPathway
IGHV1-46	28465	immunogl	IGHV146	14	BCRSignalingPathway
IGHV1-58	28464	immunogl	IGHV158|V	14	BCRSignalingPathway
IGHV1-69	28461	immunogl	IGHV1-E|I	14	BCRSignalingPathway
IGHV1-8	28472	immunogl	IGHV18	14	BCRSignalingPathway
IGHV1-38-	28460	immunogl	IGHV1-C|I	14	BCRSignalingPathway
IGHV1-69-	28458	immunogl	IGHV1-F|I	14	BCRSignalingPathway
IGHV2-26	28455	immunogl	IGHV226|V	14	BCRSignalingPathway
IGHV2-5	28457	immunogl	IGHV25|VH	14	BCRSignalingPathway
IGHV2-70	28454	immunogl	IGHV270|V	14	BCRSignalingPathway
IGHV3-11	28450	immunogl	IGHV311|V	14	BCRSignalingPathway
IGHV3-13	28449	immunogl	IGHV313	14	BCRSignalingPathway
IGHV3-15	28448	immunogl	IGHV315|V	14	BCRSignalingPathway
IGHV3-16	28447	immunogl	IGHV316|V	14	BCRSignalingPathway
IGHV3-20	28445	immunogl	IGHV320|V	14	BCRSignalingPathway

IGHV3-21	28444	immunogl(	IGHV321|V	14	BCRSignalingPathway
IGHV3-23	28442	immunogl(	DP47|IGHV	14	BCRSignalingPathway
IGHV3-30	28439	immunogl(	IGHV330|V	14	BCRSignalingPathway
IGHV3-30-	57290	immunogl(	IGHV3-3|IG	14	BCRSignalingPathway
IGHV3-30-	89770	immunogl(	IGHV3-3|IG	14	BCRSignalingPathway
IGHV3-33	28434	immunogl(	IGHV333|V	14	BCRSignalingPathway
IGHV3-35	28432	immunogl(	IGHV335|V	14	BCRSignalingPathway
IGHV3-38	28429	immunogl(	IGHV338|V	14	BCRSignalingPathway
IGHV3-43	28426	immunogl(	IGHV343|V	14	BCRSignalingPathway
IGHV3-48	28424	immunogl(	IGHV348|V	14	BCRSignalingPathway
IGHV3-49	28423	immunogl(	IGHV349|V	14	BCRSignalingPathway
IGHV3-53	28420	immunogl(	IGHV353|V	14	BCRSignalingPathway
IGHV3-64	28414	immunogl(	IGHV364|V	14	BCRSignalingPathway
IGHV3-66	28412	immunogl(	IGHV366|V	14	BCRSignalingPathway
IGHV3-7	28452	immunogl(	IGHV37|VH	14	BCRSignalingPathway
IGHV3-72	28410	immunogl(	IGHV372|V	14	BCRSignalingPathway
IGHV3-73	28409	immunogl(	IGHV373|V	14	BCRSignalingPathway
IGHV3-74	28408	immunogl(	IGHV374|V	14	BCRSignalingPathway
IGHV3-9	28451	immunogl(	IGHV39|VH	14	BCRSignalingPathway
IGHV3-38-	28404	immunogl(	IGHV3-D|IG	14	BCRSignalingPathway
IGHV3-69-	28402	immunogl(	IGH|IGHM|	14	BCRSignalingPathway
IGHV4-28	28400	immunogl(	IGHV428|V	14	BCRSignalingPathway
IGHV4-30-	28399	immunogl(	IGHV4-3	14	BCRSignalingPathway
IGHV4-30-	28398	immunogl(	IGHV4-3|IG	14	BCRSignalingPathway
IGHV4-30-	28397	immunogl(	IGHV4-3|IG	14	BCRSignalingPathway
IGHV4-31	28396	immunogl(	IGHV431	14	BCRSignalingPathway
IGHV4-34	28395	immunogl(	IGHV434|V	14	BCRSignalingPathway
IGHV4-39	28394	immunogl(	IGHV439|V	14	BCRSignalingPathway
IGHV4-4	28401	immunogl(	IGHV44|VH	14	BCRSignalingPathway
IGHV4-59	28392	immunogl(	IGHV459|V	14	BCRSignalingPathway
IGHV4-61	28391	immunogl(	IGHV461|V	14	BCRSignalingPathway
IGHV4-38-	28389	immunogl(	IGHV4-B|IG	14	BCRSignalingPathway
IGHV5-51	28388	immunogl(	IGHV551|V	14	BCRSignalingPathway
IGHV5-10-	28386	immunogl(	IGHV5-A|IG	14	BCRSignalingPathway
IGHV6-1	28385	immunogl(	IGHV61|VH	14	BCRSignalingPathway
IGHV7-4-1	57289	immunogl(	IGHV7-41|	14	BCRSignalingPathway
IGHV7-81	28378	immunogl(	IGHV781	14	BCRSignalingPathway
IGK	50802	immunogl(	IGK@	2	BCRSignalingPathway
IGKC	3514	immunogl(	HCAK1|IGK	2	BCRSignalingPathway
IGKDEL	3515	immunogl(	IGKDE	2	BCRSignalingPathway
IGKJ	7842	-	IGKJ@	2	BCRSignalingPathway
IGKJ1	28950	immunogl(	J1	2	BCRSignalingPathway
IGKJ2	28949	immunogl(	J2	2	BCRSignalingPathway
IGKJ3	28948	immunogl(	J3	2	BCRSignalingPathway
IGKJ4	28947	immunogl(	J4	2	BCRSignalingPathway
IGKJ5	28946	immunogl(	J5	2	BCRSignalingPathway
IGKV@	3519	-	IGKV|IGKV1	2	BCRSignalingPathway
IGKV1-12	28940	immunogl(	IGKV112|L1	2	BCRSignalingPathway
IGKV1-13	28939	immunogl(	IGKV113|L1	2	BCRSignalingPathway
IGKV1-16	28938	immunogl(	IGKV116|L1	2	BCRSignalingPathway
IGKV1-17	28937	immunogl(	A30|IGKV1	2	BCRSignalingPathway
IGKV1-27	28935	immunogl(	A20|IGKV1	2	BCRSignalingPathway
IGKV1-33	28933	immunogl(	IGKV133|O	2	BCRSignalingPathway
IGKV1-37	28931	immunogl(	IGKV137|O	2	BCRSignalingPathway
IGKV1-39	28930	immunogl(	IGKV139|O	2	BCRSignalingPathway
IGKV1-5	28299	immunogl(	IGKV|IGKV1	2	BCRSignalingPathway
IGKV1-6	28943	immunogl(	IGKV16|L11	2	BCRSignalingPathway
IGKV1-8	28942	immunogl(	IGKV18|L9	2	BCRSignalingPathway

IGKV1-9	28941	immunogl	IGKV19|L8	2	BCRSignalingPathway
IGKV1D-12	28903	immunogl	IGKV1D12|	2	BCRSignalingPathway
IGKV1D-13	28902	immunogl	IGKV1D13|	2	BCRSignalingPathway
IGKV1D-16	28901	immunogl	IGKV1D16|	2	BCRSignalingPathway
IGKV1D-17	28900	immunogl	IGKV1D17|	2	BCRSignalingPathway
IGKV1D-33	28896	immunogl	IGKV1D33|	2	BCRSignalingPathway
IGKV1D-37	28894	immunogl	IGKV1D37|	2	BCRSignalingPathway
IGKV1D-39	28893	immunogl	IGKV1D39|	2	BCRSignalingPathway
IGKV1D-42	28892	immunogl	IGKV1D42|	2	BCRSignalingPathway
IGKV1D-43	28891	immunogl	IGKV1D43|	2	BCRSignalingPathway
IGKV1D-8	28904	immunogl	IGKV1D8|L	2	BCRSignalingPathway
IGKV2-24	28923	immunogl	A23|IGKV2	2	BCRSignalingPathway
IGKV2-28	28921	immunogl	A19|IGKV2	2	BCRSignalingPathway
IGKV2-30	28919	immunogl	A17|IGKV2	2	BCRSignalingPathway
IGKV2-40	28916	immunogl	IGKV240|O	2	BCRSignalingPathway
IGKV2D-24	28885	immunogl	A7|IGKV2D	2	BCRSignalingPathway
IGKV2D-28	28883	immunogl	A3|IGKV2D	2	BCRSignalingPathway
IGKV2D-29	28882	immunogl	A2a|A2c|IG	2	BCRSignalingPathway
IGKV2D-30	28881	immunogl	A1|IGKV2D	2	BCRSignalingPathway
IGKV2D-40	28878	immunogl	IGKV2D40|	2	BCRSignalingPathway
IGKV3-11	28914	immunogl	IGKV311|L6	2	BCRSignalingPathway
IGKV3-15	28913	immunogl	IGKV315|L2	2	BCRSignalingPathway
IGKV3-20	28912	immunogl	13K18|A27	2	BCRSignalingPathway
IGKV3-7	28915	immunogl	IGKV37|L10	2	BCRSignalingPathway
IGKV3D-11	28876	immunogl	IGKV3D11|	2	BCRSignalingPathway
IGKV3D-15	28875	immunogl	IGKV3D15|	2	BCRSignalingPathway
IGKV3D-20	28874	immunogl	A11|A11a|I	2	BCRSignalingPathway
IGKV3D-7	28877	immunogl	IGKV3D7|L	2	BCRSignalingPathway
IGKV4-1	28908	immunogl	B3|IGKV41	2	BCRSignalingPathway
IGKV5-2	28907	immunogl	B2|IGKV52	2	BCRSignalingPathway
IGKV6-21	28906	immunogl	A26|IGKV6	2	BCRSignalingPathway
IGKV6D-21	28870	immunogl	A10|IGKV6	2	BCRSignalingPathway
IGKV6D-41	28869	immunogl	A14	2	BCRSignalingPathway
IGL	3535	immunogl	IGL@|IGLC	22	BCRSignalingPathway
IGLC1	3537	immunogl	IGLC	22	BCRSignalingPathway
IGLC2	3538	immunogl	IGLC	22	BCRSignalingPathway
IGLC3	3539	immunogl	IGLC	22	BCRSignalingPathway
IGLC6	3542	immunogl	IGLC	22	BCRSignalingPathway
IGLC7	28834	immunogl	C7	22	BCRSignalingPathway
IGLJ	8217	-	IGLJ@	22	BCRSignalingPathway
IGLJ1	28833	immunogl	J1	22	BCRSignalingPathway
IGLJ2	28832	immunogl	J2	22	BCRSignalingPathway
IGLJ3	28831	immunogl	J3	22	BCRSignalingPathway
IGLJ4	28830	immunogl	-	22	BCRSignalingPathway
IGLJ5	28829	immunogl	-	22	BCRSignalingPathway
IGLJ6	28828	immunogl	-	22	BCRSignalingPathway
IGLJ7	28827	immunogl	J7	22	BCRSignalingPathway
IGLV@	3546	-	IGLV	22	BCRSignalingPathway
IGLV1-36	28826	immunogl	IGLV136|V1	22	BCRSignalingPathway
IGLV1-40	28825	immunogl	IGLV140|V1	22	BCRSignalingPathway
IGLV1-44	28823	immunogl	IGLV144|V1	22	BCRSignalingPathway
IGLV1-47	28822	immunogl	IGLV147|V1	22	BCRSignalingPathway
IGLV1-50	28821	immunogl	IGLV150|V1	22	BCRSignalingPathway
IGLV1-51	28820	immunogl	IGLV151|V1	22	BCRSignalingPathway
IGLV10-54	28772	immunogl	IGLV1054|V	22	BCRSignalingPathway
IGLV11-55	28770	immunogl	IGLV1155|V	22	BCRSignalingPathway
IGLV2-11	28816	immunogl	IGLV211|V1	22	BCRSignalingPathway
IGLV2-14	28815	immunogl	IGLV214|V1	22	BCRSignalingPathway

IGLV2-18	28814	immunogl(	IGLV218|V1	22	BCRSignalingPathway
IGLV2-23	28813	immunogl(	IGLV223|V1	22	BCRSignalingPathway
IGLV2-33	28811	immunogl(	IGLV233|V1	22	BCRSignalingPathway
IGLV2-8	28817	immunogl(	IGLV28|V1-	22	BCRSignalingPathway
IGLV3-1	28809	immunogl(	IGLV31|V2-	22	BCRSignalingPathway
IGLV3-10	28803	immunogl(	IGLV310|V2	22	BCRSignalingPathway
IGLV3-12	28802	immunogl(	IGLV312|V2	22	BCRSignalingPathway
IGLV3-16	28799	immunogl(	IGLV316|V2	22	BCRSignalingPathway
IGLV3-19	28797	immunogl(	IGLV319|V2	22	BCRSignalingPathway
IGLV3-21	28796	immunogl(	IGLV321|V2	22	BCRSignalingPathway
IGLV3-22	28795	immunogl(	IGLV322|V2	22	BCRSignalingPathway
IGLV3-25	28793	immunogl(	IGLV325|V2	22	BCRSignalingPathway
IGLV3-27	28791	immunogl(	IGLV327|V2	22	BCRSignalingPathway
IGLV3-32	28787	immunogl(	IGLV332|V2	22	BCRSignalingPathway
IGLV3-9	28804	immunogl(	IGLV39|V2-	22	BCRSignalingPathway
IGLV4-3	28786	immunogl(	IGLV43|V5-	22	BCRSignalingPathway
IGLV4-60	28785	immunogl(	IGLV460|V5	22	BCRSignalingPathway
IGLV4-69	28784	immunogl(	IGLV469|V5	22	BCRSignalingPathway
IGLV5-37	28783	immunogl(	IGLV537|V4	22	BCRSignalingPathway
IGLV5-39	28782	immunogl(	IGLV539	22	BCRSignalingPathway
IGLV5-45	28781	immunogl(	IGLV545|V4	22	BCRSignalingPathway
IGLV5-48	28780	immunogl(	IGLV548|V4	22	BCRSignalingPathway
IGLV5-52	28779	immunogl(	IGLV552|V4	22	BCRSignalingPathway
IGLV6-57	28778	immunogl(	IGLV657|V1	22	BCRSignalingPathway
IGLV7-43	28776	immunogl(	IGLV743|V3	22	BCRSignalingPathway
IGLV7-46	28775	immunogl(	IGLV746|V3	22	BCRSignalingPathway
IGLV8-61	28774	immunogl(	IGLV861|V3	22	BCRSignalingPathway
IGLV9-49	28773	immunogl(	IGLV949|V5	22	BCRSignalingPathway
C3	718	compleme	AHUS5|AR	19	Chemokines
C5	727	compleme	C5D|C5a|C	9	Chemokines
CAMP	820	cathelicidir	CAP-18|C/	3	Chemokines
CCL1	6346	C-C motif	I-309|P500	17	Chemokines
CCL11	6356	C-C motif	SCYA11	17	Chemokines
CCL13	6357	C-C motif	CKb10|MC	17	Chemokines
CCL14	6358	C-C motif	CC-1|CC-3	17	Chemokines
CCL15-CC	348249	CCL15-CC	CCL15|HC(	17	Chemokines
CCL15	6359	C-C motif	HCC-2|HM	17	Chemokines
CCL16	6360	C-C motif	CKb12|HC(	17	Chemokines
CCL17	6361	C-C motif	A-152E5.3	16	Chemokines
CCL18	6362	C-C motif	AMAC-1|A	17	Chemokines
CCL19	6363	C-C motif	CKb11|ELC	9	Chemokines
CCL2	6347	C-C motif	GDCF-2|H(	17	Chemokines
CCL20	6364	C-C motif	CKb4|Exod	2	Chemokines
CCL21	6366	C-C motif	6Ckine|CKb	9	Chemokines
CCL22	6367	C-C motif	A-152E5.1	16	Chemokines
CCL23	6368	C-C motif	CK-BETA-8	17	Chemokines
CCL24	6369	C-C motif	Ckb-6|MPI	7	Chemokines
CCL25	6370	C-C motif	Ckb15|SCY	19	Chemokines
CCL26	10344	C-C motif	IMAC|MIP-	7	Chemokines
CCL27	10850	C-C motif	ALP|CTACk	9	Chemokines
CCL28	56477	C-C motif	CCK1|MEC	5	Chemokines
CCL3	6348	C-C motif	G0S19-1|LI	17	Chemokines
CCL3L1	6349	C-C motif	464.2|D17S	17	Chemokines
CCL3P1	390788	C-C motif	CCL3L2|G0	17	Chemokines
CCL3L3	414062	C-C motif	464.2|D17S	17	Chemokines
CCL4	6351	C-C motif	ACT2|AT74	17	Chemokines
CCL4L2	9560	C-C motif	AT744.2|C(	17	Chemokines
CCL4L1	388372	C-C motif	AT744.2|C(	17	Chemokines

CCL5	6352	C-C motif	D17S136E|	17	Chemokines
CCL7	6354	C-C motif	FIC|MARC|	17	Chemokines
CCL8	6355	C-C motif	HC14|MCP	17	Chemokines
CKLF	51192	chemokine	C32|CKLF1	16	Chemokines
CMA1	1215	chymase 1	CYH|MCT1	14	Chemokines
CTSG	1511	cathepsin (	CATG|CG	14	Chemokines
CX3CL1	6376	C-X3-C m	ABCD-3|C	16	Chemokines
CXCL1	2919	C-X-C mo	FSP|GRO1|	4	Chemokines
CXCL10	3627	C-X-C mo	C7|IFI10|IN	4	Chemokines
CXCL11	6373	C-X-C mo	H174|I-TA	4	Chemokines
CXCL12	6387	C-X-C mo	IRH|PBSF|S	10	Chemokines
CXCL13	10563	C-X-C mo	ANGIE|AN	4	Chemokines
CXCL14	9547	C-X-C mo	BMAC|BRA	5	Chemokines
CXCL16	58191	C-X-C mo	CXCLG16|S	17	Chemokines
CXCL17	284340	C-X-C mo	DMC|Dcip	19	Chemokines
CXCL2	2920	C-X-C mo	CINC-2a|G	4	Chemokines
CXCL3	2921	C-X-C mo	CINC-2b|G	4	Chemokines
CXCL5	6374	C-X-C mo	ENA-78|SC	4	Chemokines
CXCL6	6372	C-X-C mo	CKA-3|GC	4	Chemokines
CXCL9	4283	C-X-C mo	CMK|Humi	4	Chemokines
CCN1	3491	cellular co	CYR61|GIG	1	Chemokines
DEFA1	1667	defensin a	DEF1|DEFA	8	Chemokines
DEFA3	1668	defensin a	DEF3|HNP-	8	Chemokines
DEFA5	1670	defensin a	DEF5|HD-5	8	Chemokines
DEFB1	1672	defensin b	BD1|DEFB-	8	Chemokines
DEFB103B	55894	defensin b	BD-3|DEFE	8	Chemokines
DEFB104A	140596	defensin b	BD-4|DEFE	8	Chemokines
DEFB4A	1673	defensin b	BD-2|DEFE	8	Chemokines
EDN1	1906	endothelin	ARCND3|E	6	Chemokines
EDN2	1907	endothelin	ET-2|ET2|P	1	Chemokines
EDN3	1908	endothelin	ET-3|ET3|H	20	Chemokines
FGF10	2255	fibroblast g	-	5	Chemokines
FGF2	2247	fibroblast g	BFGF|FGF-	4	Chemokines
HTN3	3347	histatin 3	HIS2|HTN2	4	Chemokines
CXCL8	3576	C-X-C mo	GCP-1|GCI	4	Chemokines
LECT2	3950	leukocyte (	chm-II|chm	5	Chemokines
PF4	5196	platelet fac	CXCL4|PF-	4	Chemokines
PF4V1	5197	platelet fac	CXCL4L1|C	4	Chemokines
PLAU	5328	plasminog	ATF|BDPLT	10	Chemokines
PPBP	5473	pro-platel	B-TG1|Beta	4	Chemokines
PPBPP1	728045	pro-platel	PPBPL1|TG	4	Chemokines
PROK2	60675	prokinetici	BV8|HH4|K	3	Chemokines
RNASE2	6036	ribonuclea	EDN|RAF3|	14	Chemokines
SAA1	6288	serum amy	PIG4|SAA|S	11	Chemokines
SAA2	6289	serum amy	SAA|SAA1	11	Chemokines
SBDS	51119	SBDS ribos	CGI-97|SD	7	Chemokines
SEMA3A	10371	semaphori	COLL1|HH	7	Chemokines
SEMA3B	7869	semaphori	LUCA-1|SE	3	Chemokines
SEMA3C	10512	semaphori	SEMAE|Ser	7	Chemokines
SEMA3D	223117	semaphori	Sema-Z2|c	7	Chemokines
SEMA3E	9723	semaphori	M-SEMAH	7	Chemokines
SEMA3F	6405	semaphori	SEMA-IV|S	3	Chemokines
SEMA3G	56920	semaphori	sem2	3	Chemokines
SEMA4A	64218	semaphori	CORD10|R	1	Chemokines
SEMA4B	10509	semaphori	SEMAC|Se	15	Chemokines
SEMA4C	54910	semaphori	M-SEMA-I	2	Chemokines
SEMA4D	10507	semaphori	A8|BB18|C	9	Chemokines
SEMA4F	10505	ssemaphor	M-SEMA|P	2	Chemokines

SEMA4G	57715	semaphori	-	10	Chemokines
SEMA5A	9037	semaphori	SEMAF|sen	5	Chemokines
SEMA5B	54437	semaphori	SEMAG|Sei	3	Chemokines
SEMA6A	57556	semaphori	HT018|SEM	5	Chemokines
SEMA6B	10501	semaphori	EPM11|SEI	19	Chemokines
SEMA6C	10500	semaphori	SEMAY|m-	1	Chemokines
SEMA6D	80031	semaphori	-	15	Chemokines
SEMA7A	8482	semaphori	CD108|CD	15	Chemokines
SLIT1	6585	slit guidan	MEGF4|SLI	10	Chemokines
SLIT2	9353	slit guidan	SLIL3|Slit-2	4	Chemokines
TNC	3371	tenascin C	150-225|D	9	Chemokines
TYMP	1890	thymidine	ECGF|ECGI	22	Chemokines
XCL1	6375	X-C motif	ATAC|LPTN	1	Chemokines
XCL2	6846	X-C motif	SCM-1b|S(	1	Chemokines
C5AR1	728	compleme	C5A|C5AR|	19	Chemokine_Receptors
ACKR2	1238	atypical ch	CCBP2|CCI	3	Chemokine_Receptors
CCR1	1230	C-C motif	CD191|CKI	3	Chemokine_Receptors
CCR10	2826	C-C motif	GPR2	17	Chemokine_Receptors
CCR3	1232	C-C motif	C C CKR3|(	3	Chemokine_Receptors
CCR4	1233	C-C motif	CC-CKR-4	3	Chemokine_Receptors
CCR5	1234	C-C motif	CC-CKR-5	3	Chemokine_Receptors
CCR6	1235	C-C motif	BN-1|C-C	6	Chemokine_Receptors
CCR7	1236	C-C motif	BLR2|CC-C	17	Chemokine_Receptors
CCR8	1237	C-C motif	CC-CKR-8	3	Chemokine_Receptors
CCR9	10803	C-C motif	CC-CKR-9	3	Chemokine_Receptors
ACKR4	51554	atypical ch	CC-CKR-1	3	Chemokine_Receptors
CCRL2	9034	C-C motif	ACKR5|CKI	3	Chemokine_Receptors
CMKLR1	1240	chemerin c	CHEMERIN	12	Chemokine_Receptors
CX3CR1	1524	C-X3-C m	CCRL1|CM	3	Chemokine_Receptors
CXCR3	2833	C-X-C mo	CD182|CD:X		Chemokine_Receptors
CXCR4	7852	C-X-C mo	CD184|D2S	2	Chemokine_Receptors
CXCR5	643	C-X-C mo	BLR1|CD18	11	Chemokine_Receptors
CXCR6	10663	C-X-C mo	BONZO|CD	3	Chemokine_Receptors
ACKR3	57007	atypical ch	CMKOR1|C	2	Chemokine_Receptors
CYSLTR1	10800	cysteinyl le	CYSLT1|CY X		Chemokine_Receptors
CYSLTR2	57105	cysteinyl le	CYSLT2|CY	13	Chemokine_Receptors
ACKR1	2532	atypical ch	CCBP1|CD:	1	Chemokine_Receptors
EDNRA	1909	endothelin	ET-A|ETA|E	4	Chemokine_Receptors
EDNRB	1910	endothelin	ABCDS|ET-	13	Chemokine_Receptors
FPR1	2357	formyl pep	FMLP|FPR	19	Chemokine_Receptors
FPR2	2358	formyl pep	ALXR|FMLI	19	Chemokine_Receptors
FPR2	2358	formyl pep	ALXR|FMLI	19	Chemokine_Receptors
GPR17	2840	G protein-	-	2	Chemokine_Receptors
GPR32	2854	G protein-	RVDR1	19	Chemokine_Receptors
GPR33	2856	G protein-	-	14	Chemokine_Receptors
PTGDR2	11251	prostaglan	CD294|CRT	11	Chemokine_Receptors
C5AR2	27202	compleme	C5L2|GPF7	19	Chemokine_Receptors
CXCR1	3577	C-X-C mo	C-C|C-C-C	2	Chemokine_Receptors
CXCR2	3579	C-X-C mo	CD182|CD	2	Chemokine_Receptors
LTB4R	1241	leukotriene	BLT1|BLTR|	14	Chemokine_Receptors
LTB4R2	56413	leukotriene	BLT2|BLTR:	14	Chemokine_Receptors
PLAUR	5329	plasminog	CD87|U-PA	19	Chemokine_Receptors
PLXNA1	5361	plexin A1	NOV|NOVI	3	Chemokine_Receptors
PLXNA2	5362	plexin A2	OCT|PLXN:	1	Chemokine_Receptors
PLXNA3	55558	plexin A3	6.3|HSSEX(X		Chemokine_Receptors
PLXNA4	91584	plexin A4	FAYV2820|	7	Chemokine_Receptors
PLXNB1	5364	plexin B1	PLEXIN-B1	3	Chemokine_Receptors
PLXNB2	23654	plexin B2	MM1|Nbla	22	Chemokine_Receptors

PLXNB3	5365	plexin B3	PLEXB3|PLIX		Chemokine_Receptors
PLXNC1	10154	plexin C1	CD232|PLX	12	Chemokine_Receptors
PLXND1	23129	plexin D1	PLEXD1	3	Chemokine_Receptors
PTAFR	5724	platelet act	PAFR	1	Chemokine_Receptors
ROBO1	6091	roundabou	DUTT1|SAX	3	Chemokine_Receptors
ROBO2	6092	roundabou	SAX3	3	Chemokine_Receptors
ROBO3	64221	roundabou	HGPPS|HG	11	Chemokine_Receptors
RXFP3	51289	relaxin fam	GPCR135|F	5	Chemokine_Receptors
XCR1	2829	X-C motif	CCXCR1|GI	3	Chemokine_Receptors
ADIPOQ	9370	adiponecti	ACDC|ACR	3	Cytokines
ADM	133	adrenome	AM|PAMP	11	Cytokines
ADM2	79924	adrenome	AM2|dJ579	22	Cytokines
AGRP	181	agouti rela	AGRT|ART|	16	Cytokines
AGT	183	angiotensi	ANHU|SER	1	Cytokines
AMBN	258	ameloblast	AI1F	4	Cytokines
AMELX	265	amelogeni	AI1E|AIH1|X		Cytokines
AMH	268	anti-Mulle	MIF|MIS	19	Cytokines
ANGPTL5	253935	angiopoiet	-	11	Cytokines
ANGPTL7	10218	angiopoiet	AngX|CDT	1	Cytokines
APLN	8862	apelin	APEL|XNPEX		Cytokines
AREG	374	amphiregu	AR|AREGB|	4	Cytokines
MANF	7873	mesencepl	ARMET|AR	3	Cytokines
CDNF	441549	cerebral d	ARMETL1	10	Cytokines
ARTN	9048	artemin	ART|ENOV	1	Cytokines
AVP	551	arginine va	ADH|ARVP	20	Cytokines
AZU1	566	azurocidin	AZAMP|AZ	19	Cytokines
BDNF	627	brain deriv	ANON2|BL	11	Cytokines
BMP1	649	bone morp	OI13|PCOL	8	Cytokines
BMP10	27302	bone morp	-	2	Cytokines
BMP15	9210	bone morp	GDF9B|OD X		Cytokines
BMP2	650	bone morp	BDA2|BMP	20	Cytokines
BMP3	651	bone morp	BMP-3A	4	Cytokines
BMP4	652	bone morp	BMP2B|BM	14	Cytokines
BMP5	653	bone morp	-	6	Cytokines
BMP6	654	bone morp	VGR|VGR1	6	Cytokines
BMP7	655	bone morp	OP-1	20	Cytokines
BMP8A	353500	bone morp	OP-2	1	Cytokines
BMP8B	656	bone morp	BMP8|OP2	1	Cytokines
BTC	685	betacellulir	-	4	Cytokines
MYDGF	56005	myeloid de	C19orf10|E	19	Cytokines
C3	718	compleme	AHUS5|AR	19	Cytokines
C5	727	compleme	C5D|C5a|C	9	Cytokines
CALCA	796	calcitonin r	CALC1|CGI	11	Cytokines
CALCB	797	calcitonin r	CALC2|CGI	11	Cytokines
CAMP	820	cathelicidir	CAP-18|CA	3	Cytokines
CAT	847	catalase	-	11	Cytokines
CCK	885	cholecysto	-	3	Cytokines
CCL1	6346	C-C motif	I-309|P500	17	Cytokines
CCL11	6356	C-C motif	SCYA11	17	Cytokines
CCL13	6357	C-C motif	CKb10|MC	17	Cytokines
CCL14	6358	C-C motif	CC-1|CC-3	17	Cytokines
CCL15-CC	348249	CCL15-CC	CCL15|HC(	17	Cytokines
CCL15	6359	C-C motif	HCC-2|HM	17	Cytokines
CCL16	6360	C-C motif	CKb12|HC(	17	Cytokines
CCL17	6361	C-C motif	A-152E5.3	16	Cytokines
CCL18	6362	C-C motif	AMAC-1|A	17	Cytokines
CCL19	6363	C-C motif	CKb11|ELC	9	Cytokines
CCL2	6347	C-C motif	GDCF-2|H(	17	Cytokines

CCL20	6364	C-C motif	CKb4|Exod	2	Cytokines
CCL21	6366	C-C motif	6Ckine|CKb	9	Cytokines
CCL22	6367	C-C motif	A-152E5.1	16	Cytokines
CCL23	6368	C-C motif	CK-BETA-8	17	Cytokines
CCL24	6369	C-C motif	Ckb-6|MPI	7	Cytokines
CCL25	6370	C-C motif	Ckb15|SCY	19	Cytokines
CCL26	10344	C-C motif	IMAC|MIP-	7	Cytokines
CCL27	10850	C-C motif	ALP|CTACK	9	Cytokines
CCL28	56477	C-C motif	CCK1|MEC	5	Cytokines
CCL3	6348	C-C motif	G0S19-1|L	17	Cytokines
CCL3L1	6349	C-C motif	464.2|D17S	17	Cytokines
CCL3P1	390788	C-C motif	CCL3L2|G0	17	Cytokines
CCL3L3	414062	C-C motif	464.2|D17S	17	Cytokines
CCL4	6351	C-C motif	ACT2|AT74	17	Cytokines
CCL4L2	9560	C-C motif	AT744.2|C	17	Cytokines
CCL4L1	388372	C-C motif	AT744.2|C	17	Cytokines
CCL5	6352	C-C motif	D17S136E|	17	Cytokines
CCL7	6354	C-C motif	FIC|MARC|	17	Cytokines
CCL8	6355	C-C motif	HC14|MCP	17	Cytokines
CD320	51293	CD320 mo	8D6|8D6A|	19	Cytokines
CD40LG	959	CD40 ligan	CD154|CD4	X	Cytokines
CD70	970	CD70 mole	CD27-L|CD	19	Cytokines
ADA2	51816	adenosine	ADGF|CECI	22	Cytokines
CER1	9350	cerberus 1	DAND4	9	Cytokines
CGA	1081	glycoprote	CG-ALPHA	6	Cytokines
CGB3	1082	chorionic g	CGB|CGB5|	19	Cytokines
CGB1	114335	chorionic g	-	19	Cytokines
CGB2	114336	chorionic g	-	19	Cytokines
CGB5	93659	chorionic g	CGB|HCG|h	19	Cytokines
CGB7	94027	chorionic g	CG-beta-a	19	Cytokines
CGB8	94115	chorionic g	-	19	Cytokines
CHGA	1113	chromogra	CGA	14	Cytokines
CHGB	1114	chromogra	SCG1	20	Cytokines
CKLF	51192	chemokine	C32|CKLF1	16	Cytokines
CLCF1	23529	cardiotrop	BSF-3|BSF3	11	Cytokines
CLEC11A	6320	C-type lec	CLECSF3|L	19	Cytokines
CMA1	1215	chymase 1	CYH|MCT1	14	Cytokines
CMTM1	113540	CKLF like M	CKLFH|CKL	16	Cytokines
CMTM2	146225	CKLF like M	CKLFSF2	16	Cytokines
CMTM3	123920	CKLF like M	BNAS2|CKL	16	Cytokines
CMTM4	146223	CKLF like M	CKLFSF4	16	Cytokines
CMTM5	116173	CKLF like M	CKLFSF5	14	Cytokines
CMTM6	54918	CKLF like M	CKLFSF6|P	3	Cytokines
CMTM7	112616	CKLF like M	CKLFSF7	3	Cytokines
CMTM8	152189	CKLF like M	CKLFSF8|C	3	Cytokines
CNTF	1270	ciliary neur	HCNTF	11	Cytokines
CORT	1325	cortistatin	CST-14|CS	1	Cytokines
CRH	1392	corticotrop	CRF|CRH1	8	Cytokines
CSF1	1435	colony stin	CSF-1|MCS	1	Cytokines
CSF2	1437	colony stin	CSF|GMCS	5	Cytokines
CSF3	1440	colony stin	C17orf33|C	17	Cytokines
CSH1	1442	chorionic s	CS-1|CSA|	17	Cytokines
CSH2	1443	chorionic s	CS-2|CSB|	17	Cytokines
CSHL1	1444	chorionic s	CS-5|CSHP	17	Cytokines
CSPG5	10675	chondroitin	NGC	3	Cytokines
CTF1	1489	cardiotrop	CT-1|CT1	16	Cytokines
CCN2	1490	cellular cor	CTGF|HCS2	6	Cytokines
CTSG	1511	cathepsin C	CATG|CG	14	Cytokines

CX3CL1	6376	C-X3-C m	ABCD-3|C	16	Cytokines
CXCL1	2919	C-X-C mo	FSP|GRO1|	4	Cytokines
CXCL10	3627	C-X-C mo	C7|IFI10|IN	4	Cytokines
CXCL11	6373	C-X-C mo	H174|I-TA	4	Cytokines
CXCL12	6387	C-X-C mo	IRH|PBSF|S	10	Cytokines
CXCL13	10563	C-X-C mo	ANGIE|AN	4	Cytokines
CXCL14	9547	C-X-C mo	BMAC|BRA	5	Cytokines
CXCL16	58191	C-X-C mo	CXCLG16|S	17	Cytokines
CXCL17	284340	C-X-C mo	DMC|Dcip	19	Cytokines
CXCL2	2920	C-X-C mo	CINC-2a|G	4	Cytokines
CXCL3	2921	C-X-C mo	CINC-2b|G	4	Cytokines
CXCL5	6374	C-X-C mo	ENA-78|SC	4	Cytokines
CXCL6	6372	C-X-C mo	CKA-3|GC	4	Cytokines
CXCL9	4283	C-X-C mo	CMK|Humi	4	Cytokines
CCN1	3491	cellular co	CYR61|GIG	1	Cytokines
DEFA1	1667	defensin al	DEF1|DEFA	8	Cytokines
DEFA3	1668	defensin al	DEF3|HNP	8	Cytokines
DEFA5	1670	defensin al	DEF5|HD-	8	Cytokines
DEFB1	1672	defensin b	BD1|DEFB-	8	Cytokines
DEFB103B	55894	defensin b	BD-3|DEFE	8	Cytokines
DEFB104A	140596	defensin b	BD-4|DEFE	8	Cytokines
DEFB4A	1673	defensin b	BD-2|DEFE	8	Cytokines
DKK1	22943	dickkopf W	DKK-1|SK	10	Cytokines
EBI3	10148	Epstein-Ba	IL-27B|IL27	19	Cytokines
EDN1	1906	endothelin	ARCND3|E	6	Cytokines
EDN2	1907	endothelin	ET-2|ET2|P	1	Cytokines
EDN3	1908	endothelin	ET-3|ET3|H	20	Cytokines
EGF	1950	epidermal	HOMG4|UI	4	Cytokines
EPGN	255324	epithelial n	ALGV3072	4	Cytokines
EPO	2056	erythropoi	DBAL|ECYT	7	Cytokines
EREG	2069	epiregulin	EPR|ER|Ep	4	Cytokines
ESM1	11082	endothelia	endocan	5	Cytokines
FAM3B	54097	FAM3 met	2-21|C21o	21	Cytokines
FAM3C	10447	FAM3 met	GS3786|ILE	7	Cytokines
FAM3D	131177	FAM3 met	EF7|OIT1	3	Cytokines
FASLG	356	Fas ligand	ALPS1B|AP	1	Cytokines
FGF1	2246	fibroblast	AFGF|ECGF	5	Cytokines
FGF10	2255	fibroblast	-	5	Cytokines
FGF11	2256	fibroblast	FGF-11|FH	17	Cytokines
FGF12	2257	fibroblast	EIEE47|FGF	3	Cytokines
FGF13	2258	fibroblast	FGF-13|FG	X	Cytokines
FGF14	2259	fibroblast	FGF-14|FH	13	Cytokines
FGF16	8823	fibroblast	FGF-16|MF	X	Cytokines
FGF17	8822	fibroblast	FGF-13|FG	8	Cytokines
FGF18	8817	fibroblast	FGF-18|ZF	5	Cytokines
FGF19	9965	fibroblast	-	11	Cytokines
FGF2	2247	fibroblast	BFGF|FGF-	4	Cytokines
FGF20	26281	fibroblast	FGF-20|RH	8	Cytokines
FGF21	26291	fibroblast	-	19	Cytokines
FGF22	27006	fibroblast	-	19	Cytokines
FGF23	8074	fibroblast	ADHR|FGF	12	Cytokines
FGF3	2248	fibroblast	HBGF-3|IN	11	Cytokines
FGF4	2249	fibroblast	FGF-4|HBG	11	Cytokines
FGF5	2250	fibroblast	HBGF-5|Sm	4	Cytokines
FGF6	2251	fibroblast	HBGF-6|HS	12	Cytokines
FGF7	2252	fibroblast	HBGF-7|KG	15	Cytokines
FGF8	2253	fibroblast	AIGF|FGF-	10	Cytokines
FGF9	2254	fibroblast	FGF-9|GAF	13	Cytokines

VEGFD	2277	vascular er	FIGF|VEGF-X		Cytokines
FIGNL2	401720	fidgetin lik	-	12	Cytokines
FLT3LG	2323	fms relatec	FL|FLG3L|F	19	Cytokines
FSHB	2488	follicle stim	HH24	11	Cytokines
GAL	51083	galanin an	ETL8|GAL-	11	Cytokines
GALP	85569	galanin like	-	19	Cytokines
GAST	2520	gastrin	GAS	17	Cytokines
GCG	2641	glucagon	GLP-1|GLP	2	Cytokines
GDF1	2657	growth diff	CERS1|CHT	19	Cytokines
GDF10	2662	growth diff	BIP|BMP-3	10	Cytokines
GDF11	10220	growth diff	BMP-11|BM	12	Cytokines
GDF15	9518	growth diff	GDF-15|M	19	Cytokines
GDF2	2658	growth diff	BMP-9|BM	10	Cytokines
GDF3	9573	growth diff	KFS3|MCO	12	Cytokines
GDF5	8200	growth diff	BDA1C|BM	20	Cytokines
GDF6	392255	growth diff	BMP-13|BM	8	Cytokines
GDF7	151449	growth diff	BMP12	2	Cytokines
GDF9	2661	growth diff	POF14	5	Cytokines
GDNF	2668	glial cell de	ATF|ATF1|A	5	Cytokines
GH1	2688	growth ho	GH|GH-N|	17	Cytokines
GH2	2689	growth ho	GH-V|GHB	17	Cytokines
GHRH	2691	growth ho	GHRF|GRF|	20	Cytokines
GHRL	51738	ghrelin and	MTLRP	3	Cytokines
GIP	2695	gastric inhi	-	17	Cytokines
GKN1	56287	gastrokine	AMP18|BR	2	Cytokines
GMFB	2764	glia matura	GMF	14	Cytokines
GMFG	9535	glia matura	GMF-GAM	19	Cytokines
GNRH1	2796	gonadotro	GNRH|GRH	8	Cytokines
GNRH2	2797	gonadotro	GnRH-II|LH	20	Cytokines
GPHA2	170589	glycoprote	A2|GPA2|Z	11	Cytokines
GPHB5	122876	glycoprote	B5|GPB5|Z	14	Cytokines
GPI	2821	glucose-6-	AMF|GNPI|	19	Cytokines
GREM1	26585	gremlin 1,	C15DUPq|	15	Cytokines
GREM2	64388	gremlin 2,	CKTSF1B2|	1	Cytokines
GRN	2896	granulin pr	CLN11|GEP	17	Cytokines
GRP	2922	gastrin rele	BN|GRP-1	18	Cytokines
GUCA2A	2980	guanylate	GCAP-I|GU	1	Cytokines
HAMP	57817	hepcidin a	HEPC|HFE2	19	Cytokines
HBEGF	1839	heparin bir	DTR|DTS|D	5	Cytokines
HDGF	3068	heparin bir	HMG1L2	1	Cytokines
HDGFL3	50810	HDGF like	CGI-142|H	15	Cytokines
HGF	3082	hepatocyte	DFNB39|F-	7	Cytokines
HTN3	3347	histatin 3	HIS2|HTN2	4	Cytokines
IAPP	3375	islet amylo	DAP|IAP	12	Cytokines
IFNA1	3439	interferon	IFL|IFN|IFN	9	Cytokines
IFNA10	3446	interferon	IFN-alpha	9	Cytokines
IFNA13	3447	interferon	-	9	Cytokines
IFNA14	3448	interferon	IFN-alphaH	9	Cytokines
IFNA16	3449	interferon	IFN-alpha-	9	Cytokines
IFNA17	3451	interferon	IFN-alphaI	9	Cytokines
IFNA2	3440	interferon	IFN-alpha-	9	Cytokines
IFNA21	3452	interferon	IFN-alphaI	9	Cytokines
IFNA4	3441	interferon	IFN-alpha4	9	Cytokines
IFNA5	3442	interferon	IFN-alpha-	9	Cytokines
IFNA6	3443	interferon	IFN-alphaK	9	Cytokines
IFNA7	3444	interferon	IFN-alphaJ	9	Cytokines
IFNA8	3445	interferon	IFN-alphaB	9	Cytokines
IFNB1	3456	interferon	IFB|IFF|IFN-	9	Cytokines

IFNE	338376	interferon	IFN-E|IFNE	9 Cytokines
IFNG	3458	interferon	IFG|IFI	12 Cytokines
IFNK	56832	interferon	IFNT1|INFE	9 Cytokines
IFNW1	3467	interferon	-	9 Cytokines
IGF1	3479	insulin like	IGF|IGF-I|I	12 Cytokines
IGF2	3481	insulin like	C11orf43|	11 Cytokines
IL10	3586	interleukin	CSIF|GVHD	1 Cytokines
IL11	3589	interleukin	AGIF|IL-11	19 Cytokines
IL12A	3592	interleukin	CLMF|IL-1	3 Cytokines
IL12B	3593	interleukin	CLMF|CLM	5 Cytokines
IL13	3596	interleukin	IL-13|P600	5 Cytokines
IL15	3600	interleukin	IL-15	4 Cytokines
IL16	3603	interleukin	LCF|NIL16|	15 Cytokines
IL17A	3605	interleukin	CTLA-8|CT	6 Cytokines
IL17B	27190	interleukin	IL-17B|IL-	5 Cytokines
IL17C	27189	interleukin	CX2|IL-17	16 Cytokines
IL17D	53342	interleukin	IL-17D	13 Cytokines
IL17F	112744	interleukin	CANDF6|IL	6 Cytokines
IL18	3606	interleukin	IGIF|IL-18|	11 Cytokines
IL19	29949	interleukin	IL-10C|MD	1 Cytokines
IL1A	3552	interleukin	IL-1 alpha|	2 Cytokines
IL1B	3553	interleukin	IL-1|IL1-B	2 Cytokines
IL1F10	84639	interleukin	FIL1-theta|	2 Cytokines
IL36RN	26525	interleukin	FIL1|FIL1(D	2 Cytokines
IL36A	27179	interleukin	FIL1|FIL1(E	2 Cytokines
IL37	27178	interleukin	FIL1|FIL1(Z	2 Cytokines
IL36B	27177	interleukin	FIL1|FIL1-(	2 Cytokines
IL36G	56300	interleukin	IL-1F9|IL-1	2 Cytokines
IL1RN	3557	interleukin	DIRA|ICIL-	2 Cytokines
IL2	3558	interleukin	IL-2|TCGF|	4 Cytokines
IL20	50604	interleukin	IL-20|IL10	1 Cytokines
IL21	59067	interleukin	CVID11|IL-	4 Cytokines
IL22	50616	interleukin	IL-21|IL-2	12 Cytokines
IL23A	51561	interleukin	IL-23|IL-2	12 Cytokines
IL24	11009	interleukin	C49A|FISP|	1 Cytokines
IL25	64806	interleukin	IL17E	14 Cytokines
IL26	55801	interleukin	AK155|IL-	12 Cytokines
IL27	246778	interleukin	IL-27|IL-2	16 Cytokines
IFNL2	282616	interferon	IL-28A|IL2	19 Cytokines
IFNL3	282617	interferon	IFN-lambd	19 Cytokines
IFNL1	282618	interferon	IL-29|IL29	19 Cytokines
IL3	3562	interleukin	IL-3|MCGF	5 Cytokines
IL31	386653	interleukin	IL-31	12 Cytokines
IL32	9235	interleukin	IL-32alpha	16 Cytokines
IL33	90865	interleukin	C9orf26|D	9 Cytokines
IL34	146433	interleukin	C16orf77|I	16 Cytokines
IL4	3565	interleukin	BCGF-1|B	5 Cytokines
IL5	3567	interleukin	EDF|IL-5|T	5 Cytokines
IL6	3569	interleukin	BSF-2|BSF	7 Cytokines
IL6ST	3572	interleukin	CD130|CD	5 Cytokines
IL7	3574	interleukin	IL-7	8 Cytokines
CXCL8	3576	C-X-C mo	GCP-1|GC	4 Cytokines
IL9	3578	interleukin	HP40|IL-9|	5 Cytokines
INHA	3623	inhibin sub	-	2 Cytokines
INHBA	3624	inhibin sub	EDF|FRP	7 Cytokines
INHBB	3625	inhibin sub	-	2 Cytokines
INHBC	3626	inhibin sub	IHBC	12 Cytokines
INHBE	83729	inhibin sub	-	12 Cytokines

INS	3630	insulin	IDDM|IDDI	11	Cytokines
INS-IGF2	723961	INS-IGF2 r	INSIGF	11	Cytokines
INSL3	3640	insulin like	RLF|RLNL|I	19	Cytokines
INSL4	3641	insulin like	EPIL|PLACE	9	Cytokines
INSL5	10022	insulin like	PRO182|UI	1	Cytokines
INSL6	11172	insulin like	RIF1	9	Cytokines
JAG1	182	jagged car	AGS|AGS1|	20	Cytokines
JAG2	3714	jagged car	HJ2|SER2	14	Cytokines
FGF7P6	387628	fibroblast g	KGFLP1	9	Cytokines
FGF7P3	654466	fibroblast g	KGFLP2	9	Cytokines
KITLG	4254	KIT ligand	DCUA|DFN	12	Cytokines
KL	9365	klotho	HFTC3	13	Cytokines
LACRT	90070	lacritin	-	12	Cytokines
LECT2	3950	leukocyte c	chm-II|chm	5	Cytokines
LEFTY1	10637	left-right d	LEFTB|LEFT	1	Cytokines
LEFTY2	7044	left-right d	EBAF|LEFT/	1	Cytokines
LEP	3952	leptin	LEPD|OB|C	7	Cytokines
LHB	3972	luteinizing	CGB4|HH2	19	Cytokines
LIF	3976	LIF interleu	CDF|DIA|H	22	Cytokines
LRSAM1	90678	leucine rich	CMT2P|RIF	9	Cytokines
LTA	4049	lymphotox	LT|TNFB|TN	6	Cytokines
LTB	4050	lymphotox	TNFC|TNFS	6	Cytokines
LTBP1	4052	latent trans	-	2	Cytokines
LTBP2	4053	latent trans	C14orf141|	14	Cytokines
LTBP3	4054	latent trans	DASS|GPH	11	Cytokines
LTBP4	8425	latent trans	ARCL1C|LT	19	Cytokines
MDK	4192	midkine	ARAP|MK|N	11	Cytokines
MIA	8190	MIA SH3 d	CD-RAP	19	Cytokines
MIF	4282	macrophag	GIF|GLIF|M	22	Cytokines
MLN	4295	motilin	-	6	Cytokines
MSTN	2660	myostatin	GDF8|MSLI	2	Cytokines
NAMPT	10135	nicotinami	1110035O	7	Cytokines
NDP	4693	norrin cyst	EVR2|FEVR X		Cytokines
NENF	29937	neudesin n	CIR2|SCIRF	1	Cytokines
NGF	4803	nerve grow	Beta-NGF|	1	Cytokines
NMB	4828	neuromedi	-	15	Cytokines
NODAL	4838	nodal grow	HTX5	10	Cytokines
CCN3	4856	cellular co	IBP-9|IGFB	8	Cytokines
NPFF	8620	neuropept	FMRFAL	12	Cytokines
NPPA	4878	natriuretic	ANF|ANP|/	1	Cytokines
NPPB	4879	natriuretic	BNP	1	Cytokines
NPPC	4880	natriuretic	CNP|CNP2	2	Cytokines
NPY	4852	neuropept	PYY4	7	Cytokines
NRG1	3084	neuregulin	ARIA|GGF|	8	Cytokines
NRG2	9542	neuregulin	DON1|HRC	5	Cytokines
NRG3	10718	neuregulin	HRG3|pro-	10	Cytokines
NRG4	145957	neuregulin	HRG4	15	Cytokines
NRTN	4902	neurturin	NTN	19	Cytokines
NTF3	4908	neurotroph	HDNF|NGF	12	Cytokines
NTF4	4909	neurotroph	GLC10|GLC	19	Cytokines
NTS	4922	neurotensi	NMN-125|	12	Cytokines
NUDT6	11162	nudix hydr	ASFGF2|FG	4	Cytokines
OGN	4969	osteoglycin	OG|OIF|SLI	9	Cytokines
OSGIN1	29948	oxidative s	BDGI|OKL3	16	Cytokines
OSM	5008	oncostatin	-	22	Cytokines
OSTN	344901	osteocrin	MUSCLIN	3	Cytokines
OXT	5020	oxytocin/n	OT|OT-NP	20	Cytokines
ENDOU	8909	endonucle	P11|PP11|F	12	Cytokines

PDGFA	5154	platelet de	PDGF-A|PI	7	Cytokines
PDGFB	5155	platelet de	IBGC5|PDG	22	Cytokines
PDGFC	56034	platelet de	FALLOTEIN	4	Cytokines
PDGFD	80310	platelet de	IEGF|MSTP	11	Cytokines
PDGFRA	5156	platelet de	CD140A|PI	4	Cytokines
PDGFRB	5159	platelet de	CD140B|IB	5	Cytokines
PDGFRL	5157	platelet de	PDGRL|PRI	8	Cytokines
PDYN	5173	prodynorp	ADCA|PEN	20	Cytokines
PENK	5179	proenkeph	PE|PENK-A	8	Cytokines
PF4	5196	platelet fac	CXCL4|PF-	4	Cytokines
PF4V1	5197	platelet fac	CXCL4L1|C	4	Cytokines
PGF	5228	placental g	D12S1900|	14	Cytokines
PLAU	5328	plasminog	ATF|BDPLT	10	Cytokines
PMCH	5367	pro-melan	MCH|ppM	12	Cytokines
PNOC	5368	prepronoc	N/OFQ|NC	8	Cytokines
POMC	5443	proopiome	ACTH|CLIP	2	Cytokines
PPBP	5473	pro-platel	B-TG1|Beta	4	Cytokines
PPBPP1	728045	pro-platel	PPBPL1|TG	4	Cytokines
PPBPP2	10895	pro-platel	PPBPL2|SP	4	Cytokines
PPY	5539	pancreatic	PNP|PP	17	Cytokines
PRL	5617	prolactin	GHA1	6	Cytokines
PRLH	51052	prolactin re	PRH|PRRP	2	Cytokines
PROK1	84432	prokinetici	EGVEGF|PK	1	Cytokines
PROK2	60675	prokinetici	BV8|HH4|K	3	Cytokines
PSPN	5623	persephin	PSP	19	Cytokines
PTH	5741	parathyroi	FIH1|PTH1	11	Cytokines
PTH2	113091	parathyroi	TIP39	19	Cytokines
PTHLH	5744	parathyroi	BDE2|HHM	12	Cytokines
PTN	5764	pleiotrophi	HARP|HB-	7	Cytokines
PYY	5697	peptide YY	PYY-I|PYY1	17	Cytokines
QRFP	347148	pyroglutan	26RFa|P51	9	Cytokines
RABEP1	9135	rabaptin, R	RAB5EP|RA	17	Cytokines
RABEP2	79874	rabaptin, R	FRA	16	Cytokines
REG1A	5967	regeneratir	ICRF|P19|P	2	Cytokines
RETN	56729	resistin	ADSF|FIZZ	19	Cytokines
RETNLB	84666	resistin like	FIZZ1|FIZZ	3	Cytokines
RLN1	6013	relaxin 1	H1|H1RLX|	9	Cytokines
RLN2	6019	relaxin 2	H2|H2-RLX	9	Cytokines
RLN3	117579	relaxin 3	H3|RXN3|Z	19	Cytokines
RNASE2	6036	ribonuclea	EDN|RAF3|	14	Cytokines
S100A6	6277	S100 calciu	2A9|5B10|C	1	Cytokines
SAA1	6288	serum amy	PIG4|SAA|S	11	Cytokines
SAA2	6289	serum amy	SAA|SAA1	11	Cytokines
SBDS	51119	SBDS ribos	CGI-97|SD	7	Cytokines
SCG2	7857	secretogra	CHGC|EM6	2	Cytokines
SCGB3A1	92304	secretoglo	HIN-1|HIN	5	Cytokines
SCT	6343	secretin	-	11	Cytokines
AIMP1	9255	aminoacyl	EMAP2|EM	4	Cytokines
SECTM1	6398	secreted ar	K12|SECTM	17	Cytokines
SEMA3A	10371	semaphori	COLL1|HH	7	Cytokines
SEMA3B	7869	semaphori	LUCA-1|SE	3	Cytokines
SEMA3C	10512	semaphori	SEMAE|Ser	7	Cytokines
SEMA3D	223117	semaphori	Sema-Z2|c	7	Cytokines
SEMA3E	9723	semaphori	M-SEMAH	7	Cytokines
SEMA3F	6405	semaphori	SEMA-IV|S	3	Cytokines
SEMA3G	56920	semaphori	sem2	3	Cytokines
SEMA4A	64218	semaphori	CORD10|R	1	Cytokines
SEMA4B	10509	semaphori	SEMAC|Ser	15	Cytokines

SEMA4C	54910	semaphori	M-SEMA-I	2	Cytokines
SEMA4D	10507	semaphori	A8|BB18|C!	9	Cytokines
SEMA4F	10505	ssemaphor	M-SEMA|P	2	Cytokines
SEMA4G	57715	semaphori	-	10	Cytokines
SEMA5A	9037	semaphori	SEMAF|sen	5	Cytokines
SEMA5B	54437	semaphori	SEMAG|Sei	3	Cytokines
SEMA6A	57556	semaphori	HT018|SEN	5	Cytokines
SEMA6B	10501	semaphori	EPM11|SEN	19	Cytokines
SEMA6C	10500	semaphori	SEMAY|m-	1	Cytokines
SEMA6D	80031	semaphori	-	15	Cytokines
SEMA7A	8482	semaphori	CD108|CD\	15	Cytokines
SLIT1	6585	slit guidan	MEGF4|SLI	10	Cytokines
SLIT2	9353	slit guidan	SLIL3|Slit-2	4	Cytokines
SLURP1	57152	secreted L'	ANUP|ARS	8	Cytokines
SPP1	6696	secreted p	BNSP|BSPI|	4	Cytokines
SST	6750	somatostat	SMST	3	Cytokines
STC1	6781	stanniocalc	STC	8	Cytokines
STC2	8614	stanniocalc	STC-2|STC	5	Cytokines
TAC1	6863	tachykinin	Hs.2563|NI	7	Cytokines
TDGF1	6997	teratocarci	CR|CR-1|C	3	Cytokines
TDGF1P3	6998	teratocarci	CR-3|CRIP	X	Cytokines
TG	7038	thyroglobu	AITD3|TGN	8	Cytokines
TGFA	7039	transformi	TFGA	2	Cytokines
TGFB1	7040	transformi	CED|DPD1	19	Cytokines
TGFB2	7042	transformi	G-TSF|LDS	1	Cytokines
TGFB3	7043	transformi	ARVD|ARV	14	Cytokines
THPO	7066	thrombopo	MGDF|MK(	3	Cytokines
TNC	3371	tenascin C	150-225|D	9	Cytokines
TNF	7124	tumor nec	DIF|TNF-al	6	Cytokines
TNFRSF11I	4982	TNF recept	OCIF|OPG|	8	Cytokines
TNFSF10	8743	TNF super	APO2L|Apo	3	Cytokines
TNFSF11	8600	TNF super	CD254|OD	13	Cytokines
TNFSF12	8742	TNF super	APO3L|DR3	17	Cytokines
TNFSF13	8741	TNF super	APRIL|CD2	17	Cytokines
TNFSF13B	10673	TNF super	BAFF|BLYS|	13	Cytokines
TNFSF14	8740	TNF super	CD258|HVI	19	Cytokines
TNFSF15	9966	TNF super	TL1|TL1A|T	9	Cytokines
TNFSF18	8995	TNF super	AITRL|GITR	1	Cytokines
TNFSF4	7292	TNF super	CD134L|CD	1	Cytokines
TNFSF8	944	TNF super	CD153|CD3	9	Cytokines
TNFSF9	8744	TNF super	4-1BB-L|C	19	Cytokines
TOR2A	27433	torsin fami	TORP1	9	Cytokines
TRH	7200	thyrotropir	Pro-TRH|T	3	Cytokines
TSHB	7252	thyroid stir	TSH-B|TSH	1	Cytokines
TSLP	85480	thymic strc	-	5	Cytokines
TXLNA	200081	taxilin alph	IL14|TXLN	1	Cytokines
TYMP	1890	thymidine	ECGF|ECGI	22	Cytokines
UCN	7349	urocortin	UI|UROC	2	Cytokines
UCN2	90226	urocortin 2	SRP|UCN-I	3	Cytokines
UCN3	114131	urocortin 3	SCP|SPC|U	10	Cytokines
UTS2	10911	urotensin 2	PRO1068|U	1	Cytokines
UTS2B	257313	urotensin 2	U2B|URP|U	3	Cytokines
VEGFA	7422	vascular er	MVCD1|VE	6	Cytokines
VEGFB	7423	vascular er	VEGFL|VRF	11	Cytokines
VEGFC	7424	vascular er	Flt4-L|LMP	4	Cytokines
VGF	7425	VGF nerve	SCG7|SgVI	7	Cytokines
VIP	7432	vasoactive	PHM27	6	Cytokines
XCL1	6375	X-C motif	ATAC|LPTN	1	Cytokines

XCL2	6846	X-C motif	SCM-1b|S	1	Cytokines
ACVR1B	91	activin A re	ACTRIB|AC	12	Cytokine_Receptors
ACVR1C	130399	activin A re	ACVRLK7|A	2	Cytokine_Receptors
ACVR2A	92	activin A re	ACTRII|ACV	2	Cytokine_Receptors
ACVR2B	93	activin A re	ACTRIIB|Ac	3	Cytokine_Receptors
ACVRL1	94	activin A re	ACVRLK1|A	12	Cytokine_Receptors
ADCYAP1R	117	ADCYAP re	PAC1|PAC1	7	Cytokine_Receptors
ADIPOR1	51094	adiponecti	ACDCR1|C	1	Cytokine_Receptors
ADIPOR2	79602	adiponecti	ACDCR2|PA	12	Cytokine_Receptors
ADRB1	153	adrenocep	ADRB1R|B1	10	Cytokine_Receptors
ADRB2	154	adrenocep	ADRB2R|AI	5	Cytokine_Receptors
AGTR1	185	angiotensi	AG2S|AGTI	3	Cytokine_Receptors
AGTR2	186	angiotensi	AT2|ATGR2	X	Cytokine_Receptors
AMHR2	269	anti-Mulle	AMHR|MIS	12	Cytokine_Receptors
ANGPT1	284	angiopoiet	AGP1|AGP	8	Cytokine_Receptors
ANGPT4	51378	angiopoiet	ANG3|ANG	20	Cytokine_Receptors
ANGPTL1	9068	angiopoiet	ANG3|ANG	1	Cytokine_Receptors
ANGPTL2	23452	angiopoiet	ARP2|HARI	9	Cytokine_Receptors
ANGPTL3	27329	angiopoiet	ANG-5|AN	1	Cytokine_Receptors
ANGPTL4	51129	angiopoiet	ARP4|FIAF|	19	Cytokine_Receptors
ANGPTL6	83854	angiopoiet	AGF|ARP5	19	Cytokine_Receptors
APLNR	187	apelin rece	AGTRL1|AF	11	Cytokine_Receptors
AR	367	androgen	AIS|AR8|DH	X	Cytokine_Receptors
AVPR1A	552	arginine va	AVPR V1a|	12	Cytokine_Receptors
AVPR1B	553	arginine va	AVPR3|V1b	1	Cytokine_Receptors
AVPR2	554	arginine va	ADHR|DI1|	X	Cytokine_Receptors
BMPR1A	657	bone morp	10q23del|A	10	Cytokine_Receptors
BMPR1B	658	bone morp	ALK-6|ALK	4	Cytokine_Receptors
BMPR2	659	bone morp	BMPR-II|BN	2	Cytokine_Receptors
BRD8	10902	bromodom	SMAP|SMA	5	Cytokine_Receptors
C3AR1	719	compleme	AZ3B|C3AF	12	Cytokine_Receptors
C5AR1	728	compleme	C5A|C5AR|	19	Cytokine_Receptors
CALCR	799	calcitonin	CRT|CT-R|	7	Cytokine_Receptors
CALCRL	10203	calcitonin	CGRPR|CRI	2	Cytokine_Receptors
ACKR2	1238	atypical ch	CCBP2|CCF	3	Cytokine_Receptors
CCR1	1230	C-C motif	CD191|CKF	3	Cytokine_Receptors
CCR10	2826	C-C motif	GPR2	17	Cytokine_Receptors
CCR3	1232	C-C motif	C C CKR3|	3	Cytokine_Receptors
CCR4	1233	C-C motif	CC-CKR-4	3	Cytokine_Receptors
CCR5	1234	C-C motif	CC-CKR-5	3	Cytokine_Receptors
CCR6	1235	C-C motif	BN-1|C-C	6	Cytokine_Receptors
CCR7	1236	C-C motif	BLR2|CC-C	17	Cytokine_Receptors
CCR8	1237	C-C motif	CC-CKR-8	3	Cytokine_Receptors
CCR9	10803	C-C motif	CC-CKR-9	3	Cytokine_Receptors
ACKR4	51554	atypical ch	CC-CKR-1	3	Cytokine_Receptors
CCRL2	9034	C-C motif	ACKR5|CKF	3	Cytokine_Receptors
CD40	958	CD40 mole	Bp50|CDW	20	Cytokine_Receptors
CMKLR1	1240	chemerin c	CHEMERIN	12	Cytokine_Receptors
CNTFR	1271	ciliary neur	-	9	Cytokine_Receptors
CRHR1	1394	corticotrop	CRF-R|CRF	17	Cytokine_Receptors
CRHR2	1395	corticotrop	CRF-RB|CR	7	Cytokine_Receptors
CRIM1	51232	cysteine ric	CRIM-1|S5	2	Cytokine_Receptors
CRLF1	9244	cytokine re	CISS|CISS1	19	Cytokine_Receptors
CRLF2	64109	cytokine re	CRL2|CRLF	X|Y	Cytokine_Receptors
CRLF3	51379	cytokine re	CREME-9|	17	Cytokine_Receptors
CSF1R	1436	colony stin	BANDDOS	5	Cytokine_Receptors
CSF2RA	1438	colony stin	CD116|CD	X|Y	Cytokine_Receptors
CSF2RB	1439	colony stin	CD131|CD	22	Cytokine_Receptors

CSF3R	1441	colony stin	CD114|GC!	1	Cytokine_Receptors
CX3CR1	1524	C-X3-C m	CCRL1|CM	3	Cytokine_Receptors
CXCR3	2833	C-X-C mo	CD182|CD:X		Cytokine_Receptors
CXCR4	7852	C-X-C mo	CD184|D2S	2	Cytokine_Receptors
CXCR5	643	C-X-C mo	BLR1|CD18	11	Cytokine_Receptors
CXCR6	10663	C-X-C mo	BONZO|CD	3	Cytokine_Receptors
ACKR3	57007	atypical ch	CMKOR1|C	2	Cytokine_Receptors
CYSLTR1	10800	cysteinyl le	CYSLT1|CY X		Cytokine_Receptors
CYSLTR2	57105	cysteinyl le	CYSLT2|CY	13	Cytokine_Receptors
ACKR1	2532	atypical ch	CCBP1|CD2	1	Cytokine_Receptors
EDNRA	1909	endothelin	ET-A|ETA|E	4	Cytokine_Receptors
EDNRB	1910	endothelin	ABCDS|ET-	13	Cytokine_Receptors
EGFR	1956	epidermal	ERBB|ERBB	7	Cytokine_Receptors
ENG	2022	endoglin	END|HHT1	9	Cytokine_Receptors
EPOR	2057	erythropoi	EPO-R	19	Cytokine_Receptors
ESR1	2099	estrogen re	ER|ESR|ESR	6	Cytokine_Receptors
ESR2	2100	estrogen re	ER-BETA|E	14	Cytokine_Receptors
ESRRA	2101	estrogen re	ERR1|ERRa	11	Cytokine_Receptors
ESRRB	2103	estrogen re	DFNB35|EF	14	Cytokine_Receptors
ESRRG	2104	estrogen re	ERR-gamm	1	Cytokine_Receptors
FGFR1	2260	fibroblast g	BFGFR|CD3	8	Cytokine_Receptors
FGFR2	2263	fibroblast g	BBDS|BEK|I	10	Cytokine_Receptors
FGFR3	2261	fibroblast g	ACH|CD33	4	Cytokine_Receptors
FGFR4	2264	fibroblast g	CD334|JTK	5	Cytokine_Receptors
FGFRL1	53834	fibroblast g	FGFR-5|FG	4	Cytokine_Receptors
FLT1	2321	fms relatec	FLT|FLT-1|V	13	Cytokine_Receptors
FLT3	2322	fms relatec	CD135|FLK	13	Cytokine_Receptors
FLT4	2324	fms relatec	CHTD7|FLT	5	Cytokine_Receptors
FPR1	2357	formyl pep	FMLP|FPR	19	Cytokine_Receptors
FPR2	2358	formyl pep	ALXR|FMLI	19	Cytokine_Receptors
FPR2	2358	formyl pep	ALXR|FMLI	19	Cytokine_Receptors
FSHR	2492	follicle stim	FSHR1|FSH	2	Cytokine_Receptors
GALR2	8811	galanin rec	GAL2-R|GA	17	Cytokine_Receptors
GALR3	8484	galanin rec	-	22	Cytokine_Receptors
GCGR	2642	glucagon r	GGR|GL-R	17	Cytokine_Receptors
GHR	2690	growth ho	GHBP|GHII	5	Cytokine_Receptors
GHRHR	2692	growth ho	GHRFR|GR	7	Cytokine_Receptors
GHSR	2693	growth ho	GHDP	3	Cytokine_Receptors
GIPR	2696	gastric inhi	PGQTL2	19	Cytokine_Receptors
GLP1R	2740	glucagon l	GLP-1|GLP	6	Cytokine_Receptors
GLP2R	9340	glucagon l	-	17	Cytokine_Receptors
GNRHR	2798	gonadotro	GNRHR1|G	4	Cytokine_Receptors
GPER1	2852	G protein-	CEPR|CMK	7	Cytokine_Receptors
GPR17	2840	G protein-	-	2	Cytokine_Receptors
GPR32	2854	G protein-	RVDR1	19	Cytokine_Receptors
GPR33	2856	G protein-	-	14	Cytokine_Receptors
PTGDR2	11251	prostaglan	CD294|CRT	11	Cytokine_Receptors
C5AR2	27202	compleme	C5L2|GPF7	19	Cytokine_Receptors
HNF4A	3172	hepatocyte	FRTS4|HNI	20	Cytokine_Receptors
HNF4G	3174	hepatocyte	NR2A2|NR	8	Cytokine_Receptors
HTR3A	3359	5-hydroxy	5-HT-3|5-	11	Cytokine_Receptors
HTR3B	9177	5-hydroxy	5-HT3B	11	Cytokine_Receptors
HTR3C	170572	5-hydroxy	-	3	Cytokine_Receptors
HTR3D	200909	5-hydroxy	5HT3D	3	Cytokine_Receptors
HTR3E	285242	5-hydroxy	5-HT3-E|5	3	Cytokine_Receptors
IFNAR1	3454	interferon	AVP|IFN-a	21	Cytokine_Receptors
IFNAR2	3455	interferon	IFN-R|IFN-	21	Cytokine_Receptors
IFNGR1	3459	interferon	CD119|IFN	6	Cytokine_Receptors

IFNGR2	3460	interferon	AF-1|IFGR2	21	Cytokine_Receptors
IGF1R	3480	insulin like	CD221|IGF	15	Cytokine_Receptors
IGF2R	3482	insulin like	CD222|CI-I	6	Cytokine_Receptors
IL10RA	3587	interleukin	CD210|CD2	11	Cytokine_Receptors
IL10RB	3588	interleukin	CDW210B|	21	Cytokine_Receptors
IL11RA	3590	interleukin	CRSDA	9	Cytokine_Receptors
IL12RB1	3594	interleukin	CD212|IL-1	19	Cytokine_Receptors
IL12RB2	3595	interleukin	-	1	Cytokine_Receptors
IL13RA1	3597	interleukin	CD213A1|C	X	Cytokine_Receptors
IL13RA2	3598	interleukin	CD213A2|C	X	Cytokine_Receptors
IL15RA	3601	interleukin	CD215	10	Cytokine_Receptors
IL2RB	3560	interleukin	CD122|IL15	22	Cytokine_Receptors
IL17RA	23765	interleukin	CANDF5|C	22	Cytokine_Receptors
IL17RB	55540	interleukin	CRL4|EVI27	3	Cytokine_Receptors
IL17RC	84818	interleukin	CANDF9|IL	3	Cytokine_Receptors
IL17RD	54756	interleukin	HH18|IL-17	3	Cytokine_Receptors
IL17RE	132014	interleukin	-	3	Cytokine_Receptors
IL18R1	8809	interleukin	CD218a|CD	2	Cytokine_Receptors
IL18RAP	8807	interleukin	ACPL|CD21	2	Cytokine_Receptors
IL1R1	3554	interleukin	CD121A|D2	2	Cytokine_Receptors
IL1R2	7850	interleukin	CD121b|CD	2	Cytokine_Receptors
IL1RAP	3556	interleukin	C3orf13|IL-	3	Cytokine_Receptors
IL1RL1	9173	interleukin	DER4|FIT-1	2	Cytokine_Receptors
IL1RL2	8808	interleukin	IL-1Rrp2|IL	2	Cytokine_Receptors
IL20RA	53832	interleukin	CRF2-8|IL-	6	Cytokine_Receptors
IL20RB	53833	interleukin	DIRS1|FND	3	Cytokine_Receptors
IL21R	50615	interleukin	CD360|IMD	16	Cytokine_Receptors
IL22RA1	58985	interleukin	CRF2-9|IL2	1	Cytokine_Receptors
IL22RA2	116379	interleukin	CRF2-10|C	6	Cytokine_Receptors
IL23R	149233	interleukin	-	1	Cytokine_Receptors
IL27RA	9466	interleukin	CRL1|IL-27	19	Cytokine_Receptors
IFNLR1	163702	interferon	CRF2/12|IF	1	Cytokine_Receptors
IL2RA	3559	interleukin	CD25|IDDM	10	Cytokine_Receptors
IL2RB	3560	interleukin	CD122|IL15	22	Cytokine_Receptors
IL2RG	3561	interleukin	CD132|CID	X	Cytokine_Receptors
IL31RA	133396	interleukin	CRL|CRL3|C	5	Cytokine_Receptors
IL3RA	3563	interleukin	CD123|IL3F	X|Y	Cytokine_Receptors
IL4R	3566	interleukin	CD124|IL-4	16	Cytokine_Receptors
IL5RA	3568	interleukin	CD125|CDw	3	Cytokine_Receptors
IL6R	3570	interleukin	CD126|IL-6	1	Cytokine_Receptors
IL7R	3575	interleukin	CD127|CDW	5	Cytokine_Receptors
CXCR1	3577	C-X-C mo	C-C|C-C-C	2	Cytokine_Receptors
CXCR2	3579	C-X-C mo	CD182|CDw	2	Cytokine_Receptors
IL9R	3581	interleukin	CD129|IL-9	X|Y	Cytokine_Receptors
INSR	3643	insulin rec	CD220|HH	19	Cytokine_Receptors
KDR	3791	kinase inse	CD309|FLK	4	Cytokine_Receptors
LEPR	3953	leptin rece	CD295|LEP	1	Cytokine_Receptors
LGR4	55366	leucine rich	BNMD17|G	11	Cytokine_Receptors
LGR5	8549	leucine rich	FEX|GPR49	12	Cytokine_Receptors
LGR6	59352	leucine rich	GPCR|VTS2	1	Cytokine_Receptors
LHCGR	3973	luteinizing	HHG|LCGR	2	Cytokine_Receptors
LIFR	3977	LIF recepto	CD118|LIF-	5	Cytokine_Receptors
LTB4R	1241	leukotriene	BLT1|BLTR|	14	Cytokine_Receptors
LTB4R2	56413	leukotriene	BLT2|BLTR2	14	Cytokine_Receptors
LTBR	4055	lymphotox	D12S370|L	12	Cytokine_Receptors
MC1R	4157	melanocor	CMM5|MS	16	Cytokine_Receptors
MC2R	4158	melanocor	ACTHR	18	Cytokine_Receptors
MC3R	4159	melanocor	BMIQ9|MC	20	Cytokine_Receptors

MC4R	4160	melanocor	BMIQ20	18	Cytokine_Receptors
MCHR1	2847	melanin cc	GPR24|MC	22	Cytokine_Receptors
MCHR2	84539	melanin cc	GPR145|GI	6	Cytokine_Receptors
MET	4233	MET proto	AUTS9|DFI	7	Cytokine_Receptors
MLNR	2862	motilin rec	GPR38|MT	13	Cytokine_Receptors
MPL	4352	MPL proto	C-MPL|CD	1	Cytokine_Receptors
MTNR1A	4543	melatonin	MEL-1A-R	4	Cytokine_Receptors
MTNR1B	4544	melatonin	FGQTL2|M	11	Cytokine_Receptors
NGFR	4804	nerve grov	CD271|Gp8	17	Cytokine_Receptors
NMBR	4829	neuromedi	BB1|BB1R|I	6	Cytokine_Receptors
NPR1	4881	natriuretic	ANPRA|AN	1	Cytokine_Receptors
NPR3	4883	natriuretic	ANP-C|AN	5	Cytokine_Receptors
NR0B1	190	nuclear rec	AHC|AHCH	X	Cytokine_Receptors
NR0B2	8431	nuclear rec	SHP|SHP1	1	Cytokine_Receptors
NR1D1	9572	nuclear rec	EAR1|REVE	17	Cytokine_Receptors
NR1D2	9975	nuclear rec	BD73|EAR-	3	Cytokine_Receptors
NR1H2	7376	nuclear rec	LXR-b|LXR	19	Cytokine_Receptors
NR1H3	10062	nuclear rec	LXR-a|LXR	11	Cytokine_Receptors
NR1H4	9971	nuclear rec	BAR|FXR|H	12	Cytokine_Receptors
NR1I2	8856	nuclear rec	BXR|ONR1	3	Cytokine_Receptors
NR1I3	9970	nuclear rec	CAR|CAR1|	1	Cytokine_Receptors
NR2C1	7181	nuclear rec	TR2	12	Cytokine_Receptors
NR2C2	7182	nuclear rec	TAK1|TR4	3	Cytokine_Receptors
NR2E1	7101	nuclear rec	TLL|TLX|XT	6	Cytokine_Receptors
NR2E3	10002	nuclear rec	ESCS|PNR|I	15	Cytokine_Receptors
NR2F1	7025	nuclear rec	BBOAS|BBS	5	Cytokine_Receptors
NR2F2	7026	nuclear rec	ARP-1|ARF	15	Cytokine_Receptors
NR2F6	2063	nuclear rec	EAR-2|EAR	19	Cytokine_Receptors
NR3C1	2908	nuclear rec	GCCR|GCR	5	Cytokine_Receptors
NR3C2	4306	nuclear rec	MCR|MLR|I	4	Cytokine_Receptors
NR4A1	3164	nuclear rec	GFRP1|HM	12	Cytokine_Receptors
NR4A2	4929	nuclear rec	HZF-3|NO	2	Cytokine_Receptors
NR4A3	8013	nuclear rec	CHN|CSMF	9	Cytokine_Receptors
NR5A1	2516	nuclear rec	AD4BP|ELF	9	Cytokine_Receptors
NR5A2	2494	nuclear rec	B1F|B1F2|C	1	Cytokine_Receptors
NR6A1	2649	nuclear rec	CT150|GCI	9	Cytokine_Receptors
NRP1	8829	neuropilin	BDCA4|CD	10	Cytokine_Receptors
NRP2	8828	neuropilin	NP2|NPN2	2	Cytokine_Receptors
OGFR	11054	opioid gro	-	20	Cytokine_Receptors
OPRD1	4985	opioid rece	DOP|DOR|	1	Cytokine_Receptors
OPRK1	4986	opioid rece	K-OR-1|KC	8	Cytokine_Receptors
OPRL1	4987	opioid rela	KOR-3|KOI	20	Cytokine_Receptors
OPRM1	4988	opioid rece	LMOR|M-C	6	Cytokine_Receptors
OSMR	9180	oncostatin	IL-31R-bet	5	Cytokine_Receptors
OXTR	5021	oxytocin re	OT-R	3	Cytokine_Receptors
PGR	5241	progesterc	NR3C3|PR	11	Cytokine_Receptors
PGRMC2	10424	progesterc	DG6|PMBP	4	Cytokine_Receptors
PLAUR	5329	plasminog	CD87|U-PA	19	Cytokine_Receptors
PLXNA1	5361	plexin A1	NOV|NOVI	3	Cytokine_Receptors
PLXNA2	5362	plexin A2	OCT|PLXN	1	Cytokine_Receptors
PLXNA3	55558	plexin A3	6.3|HSSEX(	X	Cytokine_Receptors
PLXNA4	91584	plexin A4	FAYV2820|	7	Cytokine_Receptors
PLXNB1	5364	plexin B1	PLEXIN-B1	3	Cytokine_Receptors
PLXNB2	23654	plexin B2	MM1|Nbla	22	Cytokine_Receptors
PLXNB3	5365	plexin B3	PLEXB3|PLI	X	Cytokine_Receptors
PLXNC1	10154	plexin C1	CD232|PLX	12	Cytokine_Receptors
PLXND1	23129	plexin D1	PLEXD1	3	Cytokine_Receptors
PPARA	5465	peroxisom	NR1C1|PPA	22	Cytokine_Receptors

PPARD	5467	peroxisom	FAAR|NR1(	6	Cytokine_Receptors
PPARG	5468	peroxisom	CIMT1|GLN	3	Cytokine_Receptors
PRLHR	2834	prolactin r	GPR10|GR3	10	Cytokine_Receptors
PRLR	5618	prolactin r	HPRL|MFA	5	Cytokine_Receptors
PTAFR	5724	platelet act	PAFR	1	Cytokine_Receptors
PTGDR	5729	prostaglan	AS1|ASRT1	14	Cytokine_Receptors
PTGDS	5730	prostaglan	L-PGDS|LP	9	Cytokine_Receptors
PTGER1	5731	prostaglan	EP1	19	Cytokine_Receptors
PTGER2	5732	prostaglan	EP2	14	Cytokine_Receptors
PTGER3	5733	prostaglan	EP3|EP3-I|E	1	Cytokine_Receptors
PTGER4	5734	prostaglan	EP4|EP4R	5	Cytokine_Receptors
PTGFR	5737	prostaglan	FP	1	Cytokine_Receptors
PTH1R	5745	parathyroi	EKNS|PFE|F	3	Cytokine_Receptors
PTH2R	5746	parathyroi	PTHR2	2	Cytokine_Receptors
RARA	5914	retinoic aci	NR1B1|RAF	17	Cytokine_Receptors
RARB	5915	retinoic aci	HAP|MCOF	3	Cytokine_Receptors
RARG	5916	retinoic aci	NR1B3|RAF	12	Cytokine_Receptors
ROBO1	6091	roundabou	DUTT1|SAX	3	Cytokine_Receptors
ROBO2	6092	roundabou	SAX3	3	Cytokine_Receptors
ROBO3	64221	roundabou	HGPPS|HG	11	Cytokine_Receptors
RORA	6095	RAR relate	IDDECA|NF	15	Cytokine_Receptors
RORB	6096	RAR relate	EIG15|NR1	9	Cytokine_Receptors
RORC	6097	RAR relate	IMD42|NR1	1	Cytokine_Receptors
RXFP1	59350	relaxin fam	LGR7|RXFP	4	Cytokine_Receptors
RXFP2	122042	relaxin fam	GPR106|GF	13	Cytokine_Receptors
RXFP3	51289	relaxin fam	GPCR135|F	5	Cytokine_Receptors
RXRA	6256	retinoid X	NR2B1	9	Cytokine_Receptors
RXRB	6257	retinoid X	DAUDI6|H-	6	Cytokine_Receptors
RXRG	6258	retinoid X	NR2B3|RXF	1	Cytokine_Receptors
S1PR1	1901	sphingosin	CD363|CHI	1	Cytokine_Receptors
S1PR2	9294	sphingosin	AGR16|DFI	19	Cytokine_Receptors
SCTR	6344	secretin re	SR	2	Cytokine_Receptors
SDC1	6382	syndecan 1	CD138|SD(	2	Cytokine_Receptors
SDC2	6383	syndecan 2	CD362|HSF	8	Cytokine_Receptors
SDC3	9672	syndecan 3	SDCN|SYN	1	Cytokine_Receptors
SDC4	6385	syndecan 4	SYND4	20	Cytokine_Receptors
SORT1	6272	sortilin 1	Gp95|LDLC	1	Cytokine_Receptors
SSTR1	6751	somatostat	SRIF-2|SS-	14	Cytokine_Receptors
SSTR2	6752	somatostat	-	17	Cytokine_Receptors
SSTR5	6755	somatostat	SS-5-R	16	Cytokine_Receptors
ST2	6761	-	-	11	Cytokine_Receptors
TACR1	6869	tachykinin	NK1R|NKIF	2	Cytokine_Receptors
TEK	7010	TEK recept	CD202B|GL	9	Cytokine_Receptors
TGFBR1	7046	transformi	AAT5|ACVI	9	Cytokine_Receptors
TGFBR2	7048	transformi	AAT3|FAA3	3	Cytokine_Receptors
TGFBR3	7049	transformi	BGCAN|be	1	Cytokine_Receptors
THRA	7067	thyroid ho	AR7|CHNG	17	Cytokine_Receptors
THRB	7068	thyroid ho	C-ERBA-2|	3	Cytokine_Receptors
TIE1	7075	tyrosine ki	JTK14|TIE	1	Cytokine_Receptors
TNFRSF10A	8797	TNF recept	APO2|CD2	8	Cytokine_Receptors
TNFRSF10B	8795	TNF recept	CD262|DR5	8	Cytokine_Receptors
TNFRSF10C	8794	TNF recept	CD263|DCI	8	Cytokine_Receptors
TNFRSF10D	8793	TNF recept	CD264|DCI	8	Cytokine_Receptors
TNFRSF11A	8792	TNF recept	CD265|FEC	18	Cytokine_Receptors
TNFRSF12A	51330	TNF recept	CD266|FN1	16	Cytokine_Receptors
TNFRSF13B	23495	TNF recept	CD267|CVI	17	Cytokine_Receptors
TNFRSF13C	115650	TNF recept	BAFF-R|BA	22	Cytokine_Receptors
TNFRSF14	8764	TNF recept	ATAR|CD2	1	Cytokine_Receptors

TNFRSF17	608	TNF recept	BCM|BCM/	16	Cytokine_Receptors
TNFRSF18	8784	TNF recept	AITR|CD35	1	Cytokine_Receptors
TNFRSF19	55504	TNF recept	TAJ|TAJ-al	13	Cytokine_Receptors
TNFRSF1A	7132	TNF recept	CD120a|FP	12	Cytokine_Receptors
TNFRSF1B	7133	TNF recept	CD120b|TE	1	Cytokine_Receptors
TNFRSF21	27242	TNF recept	BM-018|CI	6	Cytokine_Receptors
TNFRSF25	8718	TNF recept	APO-3|DD	1	Cytokine_Receptors
TNFRSF4	7293	TNF recept	ACT35|CD1	1	Cytokine_Receptors
TNFRSF6B	8771	TNF recept	DCR3|DJ58	20	Cytokine_Receptors
TNFRSF8	943	TNF recept	CD30|D1S1	1	Cytokine_Receptors
TNFRSF9	3604	TNF recept	4-1BB|CD1	1	Cytokine_Receptors
TRHR	7201	thyrotropir	CHNG7|TR	8	Cytokine_Receptors
TSHR	7253	thyroid stir	CHNG1|LG	14	Cytokine_Receptors
TUBB3	10381	tubulin bet	CDCBM|CI	16	Cytokine_Receptors
VDR	7421	vitamin D r	NR1I1|PPP	12	Cytokine_Receptors
VIPR1	7433	vasoactive	HVR1|II|PA	3	Cytokine_Receptors
VIPR2	7434	vasoactive	C16DUPq3	7	Cytokine_Receptors
XCR1	2829	X-C motif	CCXCR1|GI	3	Cytokine_Receptors
IFNA10	3446	interferon	IFN-alpha(	9	Interferons
IFNA13	3447	interferon	-	9	Interferons
IFNA14	3448	interferon	IFN-alphaI	9	Interferons
IFNA16	3449	interferon	IFN-alpha-	9	Interferons
IFNA17	3451	interferon	IFN-alphaI	9	Interferons
IFNA2	3440	interferon	IFN-alpha-	9	Interferons
IFNA21	3452	interferon	IFN-alphaI	9	Interferons
IFNA4	3441	interferon	IFN-alpha4	9	Interferons
IFNA5	3442	interferon	IFN-alpha-	9	Interferons
IFNA6	3443	interferon	IFN-alphaI	9	Interferons
IFNA7	3444	interferon	IFN-alphaJ	9	Interferons
IFNA8	3445	interferon	IFN-alphaE	9	Interferons
IFNB1	3456	interferon	IFB|IFF|IFN-	9	Interferons
IFNE	338376	interferon	IFN-E|IFNE	9	Interferons
IFNG	3458	interferon	IFG|IFI	12	Interferons
IFNK	56832	interferon	IFNT1|INFE	9	Interferons
IFNW1	3467	interferon	-	9	Interferons
IFNAR2	3455	interferon	IFN-R|IFN-	21	Interferon_Receptor
IFNGR1	3459	interferon	CD119|IFN	6	Interferon_Receptor
IFNGR2	3460	interferon	AF-1|IFGR2	21	Interferon_Receptor
IL11	3589	interleukin	AGIF|IL-11	19	Interleukins
IL12A	3592	interleukin	CLMF|IL-12	3	Interleukins
IL12B	3593	interleukin	CLMF|CLM	5	Interleukins
IL13	3596	interleukin	IL-13|P600	5	Interleukins
IL15	3600	interleukin	IL-15	4	Interleukins
IL16	3603	interleukin	LCF|NIL16|	15	Interleukins
IL17A	3605	interleukin	CTLA-8|CT	6	Interleukins
IL17B	27190	interleukin	IL-17B|IL-2	5	Interleukins
IL17C	27189	interleukin	CX2|IL-17C	16	Interleukins
IL17D	53342	interleukin	IL-17D	13	Interleukins
IL17F	112744	interleukin	CANDF6|IL	6	Interleukins
IL18	3606	interleukin	IGIF|IL-18|I	11	Interleukins
IL19	29949	interleukin	IL-10C|MD	1	Interleukins
IL1A	3552	interleukin	IL-1 alpha|	2	Interleukins
IL1B	3553	interleukin	IL-1|IL1-BE	2	Interleukins
IL1F10	84639	interleukin	FIL1-theta|	2	Interleukins
IL36RN	26525	interleukin	FIL1|FIL1(D	2	Interleukins
IL36A	27179	interleukin	FIL1|FIL1(E	2	Interleukins
IL37	27178	interleukin	FIL1|FIL1(Z	2	Interleukins
IL36B	27177	interleukin	FIL1|FIL1-(I	2	Interleukins

IL36G	56300	interleukin	IL-1F9|IL-1	2 Interleukins
IL1RN	3557	interleukin	DIRA|ICIL-.	2 Interleukins
IL2	3558	interleukin	IL-2|TCGF|	4 Interleukins
IL20	50604	interleukin	IL-20|IL10D	1 Interleukins
IL21	59067	interleukin	CVID11|IL-	4 Interleukins
IL22	50616	interleukin	IL-21|IL-22	12 Interleukins
IL23A	51561	interleukin	IL-23|IL-23	12 Interleukins
IL24	11009	interleukin	C49A|FISP|	1 Interleukins
IL25	64806	interleukin	IL17E	14 Interleukins
IL26	55801	interleukin	AK155|IL-2	12 Interleukins
IL27	246778	interleukin	IL-27|IL-27	16 Interleukins
IFNL2	282616	interferon	IL-28A|IL28	19 Interleukins
IFNL3	282617	interferon	IFN-lambd	19 Interleukins
IFNL1	282618	interferon	IL-29|IL29	19 Interleukins
IL3	3562	interleukin	IL-3|MCGF	5 Interleukins
IL31	386653	interleukin	IL-31	12 Interleukins
IL32	9235	interleukin	IL-32alpha	16 Interleukins
IL33	90865	interleukin	C9orf26|DV	9 Interleukins
IL34	146433	interleukin	C16orf77|II	16 Interleukins
IL4	3565	interleukin	BCGF-1|BC	5 Interleukins
IL5	3567	interleukin	EDF|IL-5|TI	5 Interleukins
IL6	3569	interleukin	BSF-2|BSF2	7 Interleukins
IL6ST	3572	interleukin	CD130|CDV	5 Interleukins
IL7	3574	interleukin	IL-7	8 Interleukins
CXCL8	3576	C-X-C mo	GCP-1|GCI	4 Interleukins
IL9	3578	interleukin	HP40|IL-9|	5 Interleukins
TXLNA	200081	taxilin alph	IL14|TXLN	1 Interleukins
IL10RA	3587	interleukin	CD210|CD2	11 Interleukins_Receptor
IL10RB	3588	interleukin	CDW210B|	21 Interleukins_Receptor
IL11RA	3590	interleukin	CRSDA	9 Interleukins_Receptor
IL12RB1	3594	interleukin	CD212|IL-1	19 Interleukins_Receptor
IL12RB2	3595	interleukin	-	1 Interleukins_Receptor
IL13RA1	3597	interleukin	CD213A1|CX	Interleukins_Receptor
IL13RA2	3598	interleukin	CD213A2|CX	Interleukins_Receptor
IL15RA	3601	interleukin	CD215	10 Interleukins_Receptor
IL2RB	3560	interleukin	CD122|IL15	22 Interleukins_Receptor
IL17RA	23765	interleukin	CANDF5|C	22 Interleukins_Receptor
IL17RB	55540	interleukin	CRL4|EVI27	3 Interleukins_Receptor
IL17RC	84818	interleukin	CANDF9|IL	3 Interleukins_Receptor
IL17RD	54756	interleukin	HH18|IL-17	3 Interleukins_Receptor
IL17RE	132014	interleukin	-	3 Interleukins_Receptor
IL18R1	8809	interleukin	CD218a|CD	2 Interleukins_Receptor
IL18RAP	8807	interleukin	ACPL|CD21	2 Interleukins_Receptor
IL1R1	3554	interleukin	CD121A|D2	2 Interleukins_Receptor
IL1R2	7850	interleukin	CD121b|CD	2 Interleukins_Receptor
IL1RAP	3556	interleukin	C3orf13|IL-	3 Interleukins_Receptor
IL1RL1	9173	interleukin	DER4|FIT-1	2 Interleukins_Receptor
IL1RL2	8808	interleukin	IL-1Rrp2|IL	2 Interleukins_Receptor
IL20RA	53832	interleukin	CRF2-8|IL-	6 Interleukins_Receptor
IL20RB	53833	interleukin	DIRS1|FND	3 Interleukins_Receptor
IL21R	50615	interleukin	CD360|IMD	16 Interleukins_Receptor
IL22RA1	58985	interleukin	CRF2-9|IL2	1 Interleukins_Receptor
IL22RA2	116379	interleukin	CRF2-10|C	6 Interleukins_Receptor
IL23R	149233	interleukin	-	1 Interleukins_Receptor
IL27RA	9466	interleukin	CRL1|IL-27	19 Interleukins_Receptor
IFNLR1	163702	interferon	CRF2/12|IF	1 Interleukins_Receptor
IL2RA	3559	interleukin	CD25|IDDM	10 Interleukins_Receptor
IL2RB	3560	interleukin	CD122|IL15	22 Interleukins_Receptor

IL2RG	3561	interleukin	CD132|CIDX		Interleukins_Receptor
IL31RA	133396	interleukin	CRL|CRL3|(	5	Interleukins_Receptor
IL3RA	3563	interleukin	CD123|IL3RX|Y		Interleukins_Receptor
IL4R	3566	interleukin	CD124|IL-4	16	Interleukins_Receptor
IL5RA	3568	interleukin	CD125|CD\	3	Interleukins_Receptor
IL6R	3570	interleukin	CD126|IL-6	1	Interleukins_Receptor
IL7R	3575	interleukin	CD127|CD\	5	Interleukins_Receptor
CXCR1	3577	C-X-C mo	C-C|C-C-C	2	Interleukins_Receptor
CXCR2	3579	C-X-C mo	CD182|CD\	2	Interleukins_Receptor
IL9R	3581	interleukin	CD129|IL-9X|Y		Interleukins_Receptor
ST2	6761	-	-	11	Interleukins_Receptor
HLA-A	3105	major histo	HLAA	6	NaturalKiller_Cell_Cytotoxicity
HLA-B	3106	major histo	AS|B-4901	6	NaturalKiller_Cell_Cytotoxicity
HLA-C	3107	major histo	D6S204|HL	6	NaturalKiller_Cell_Cytotoxicity
HLA-E	3133	major histo	HLA-6.2|Q	6	NaturalKiller_Cell_Cytotoxicity
HLA-G	3135	major histo	MHC-G	6	NaturalKiller_Cell_Cytotoxicity
KIR3DL1	3811	killer cell ir	CD158E1|K	19	NaturalKiller_Cell_Cytotoxicity
KIR3DL2	3812	killer cell ir	3DL2|CD15	19	NaturalKiller_Cell_Cytotoxicity
KIR2DL1	3802	killer cell ir	CD158A|KI	19	NaturalKiller_Cell_Cytotoxicity
KIR2DL2	3803	killer cell ir	CD158B1|C	19	NaturalKiller_Cell_Cytotoxicity
KIR2DL3	3804	killer cell ir	CD158B2|C	19	NaturalKiller_Cell_Cytotoxicity
KIR2DL4	3805	killer cell ir	CD158D|G	19	NaturalKiller_Cell_Cytotoxicity
KIR2DL5A	57292	killer cell ir	CD158F|KI	19	NaturalKiller_Cell_Cytotoxicity
KLRC1	3821	killer cell le	CD159A|N	12	NaturalKiller_Cell_Cytotoxicity
KLRC2	3822	killer cell le	CD159c|Nk	12	NaturalKiller_Cell_Cytotoxicity
KLRC3	3823	killer cell le	NKG2-E|NI	12	NaturalKiller_Cell_Cytotoxicity
KLRD1	3824	killer cell le	CD94	12	NaturalKiller_Cell_Cytotoxicity
PTPN6	5777	protein tyr	HCP|HCPH	12	NaturalKiller_Cell_Cytotoxicity
PTPN11	5781	protein tyr	BPTP3|CFC	12	NaturalKiller_Cell_Cytotoxicity
ICAM1	3383	intercellula	BB2|CD54|I	19	NaturalKiller_Cell_Cytotoxicity
ICAM2	3384	intercellula	CD102	17	NaturalKiller_Cell_Cytotoxicity
ITGAL	3683	integrin su	CD11A|LFA	16	NaturalKiller_Cell_Cytotoxicity
ITGB2	3689	integrin su	CD18|LAD|	21	NaturalKiller_Cell_Cytotoxicity
PTK2B	2185	protein tyr	CADTK|CA	8	NaturalKiller_Cell_Cytotoxicity
VAV3	10451	vav guanin	-	1	NaturalKiller_Cell_Cytotoxicity
VAV1	7409	vav guanin	VAV	19	NaturalKiller_Cell_Cytotoxicity
VAV2	7410	vav guanin	VAV-2	9	NaturalKiller_Cell_Cytotoxicity
RAC1	5879	Rac family	MIG5|MRD	7	NaturalKiller_Cell_Cytotoxicity
RAC2	5880	Rac family	EN-7|Gx|H	22	NaturalKiller_Cell_Cytotoxicity
RAC3	5881	Rac family	-	17	NaturalKiller_Cell_Cytotoxicity
PAK1	5058	p21 (RAC1	IDDMSSD|I	11	NaturalKiller_Cell_Cytotoxicity
MAP2K1	5604	mitogen-a	CFC3|MAP	15	NaturalKiller_Cell_Cytotoxicity
MAP2K2	5605	mitogen-a	CFC4|MAP	19	NaturalKiller_Cell_Cytotoxicity
MAPK1	5594	mitogen-a	ERK|ERK-2	22	NaturalKiller_Cell_Cytotoxicity
MAPK3	5595	mitogen-a	ERK-1|ERK	16	NaturalKiller_Cell_Cytotoxicity
TNF	7124	tumor neci	DIF|TNF-al	6	NaturalKiller_Cell_Cytotoxicity
CSF2	1437	colony stin	CSF|GMCS	5	NaturalKiller_Cell_Cytotoxicity
IFNG	3458	interferon	IFG|IFI	12	NaturalKiller_Cell_Cytotoxicity
KIR2DS1	3806	killer cell ir	CD158H|CI	19	NaturalKiller_Cell_Cytotoxicity
KIR2DS3	3808	killer cell ir	NKAT7	19	NaturalKiller_Cell_Cytotoxicity
KIR2DS4	3809	killer cell ir	CD158I|KIF	19	NaturalKiller_Cell_Cytotoxicity
KIR2DS5	3810	killer cell ir	CD158G|N	19	NaturalKiller_Cell_Cytotoxicity
NCR2	9436	natural cyt	CD336|LY9	6	NaturalKiller_Cell_Cytotoxicity
TYROBP	7305	transmemb	DAP12|KAI	19	NaturalKiller_Cell_Cytotoxicity
LCK	3932	LCK proto-	IMD22|LSK	1	NaturalKiller_Cell_Cytotoxicity
FCGR3A	2214	Fc fragmer	CD16|CD16	1	NaturalKiller_Cell_Cytotoxicity
FCGR3B	2215	Fc fragmer	CD16|CD16	1	NaturalKiller_Cell_Cytotoxicity
NCR1	9437	natural cyt	CD335|LY9	19	NaturalKiller_Cell_Cytotoxicity

NCR3	259197	natural cyt	1C7|CD337	6	NaturalKiller_Cell_Cytotoxicity
FCER1G	2207	Fc fragmer	FCRG	1	NaturalKiller_Cell_Cytotoxicity
CD247	919	CD247 mo	CD3-ZETA	1	NaturalKiller_Cell_Cytotoxicity
ZAP70	7535	zeta chain	ADMIO2|IM	2	NaturalKiller_Cell_Cytotoxicity
SYK	6850	spleen ass	p72-Syk	9	NaturalKiller_Cell_Cytotoxicity
LCP2	3937	lymphocyt	SLP-76|SLF	5	NaturalKiller_Cell_Cytotoxicity
LAT	27040	linker for a	IMD52|LAT	16	NaturalKiller_Cell_Cytotoxicity
PLCG1	5335	phospholi	NCKAP3|PI	20	NaturalKiller_Cell_Cytotoxicity
PLCG2	5336	phospholi	APLAID|FC	16	NaturalKiller_Cell_Cytotoxicity
SH3BP2	6452	SH3 doma	3BP-2|3BP	4	NaturalKiller_Cell_Cytotoxicity
PIK3CA	5290	phosphatic	CLAPO|CL(	3	NaturalKiller_Cell_Cytotoxicity
PIK3CB	5291	phosphatic	P110BETA|	3	NaturalKiller_Cell_Cytotoxicity
PIK3CD	5293	phosphatic	APDS|IMD1	1	NaturalKiller_Cell_Cytotoxicity
PIK3CG	5294	phosphatic	PI3CG|PI3K	7	NaturalKiller_Cell_Cytotoxicity
PIK3R5	23533	phosphoin	F730038I1!	17	NaturalKiller_Cell_Cytotoxicity
PIK3R1	5295	phosphoin	AGM7|GRE	5	NaturalKiller_Cell_Cytotoxicity
PIK3R2	5296	phosphoin	MPPH|MPF	19	NaturalKiller_Cell_Cytotoxicity
PIK3R3	8503	phosphoin	p55|p55-G	1	NaturalKiller_Cell_Cytotoxicity
FYN	2534	FYN proto-	SLK|SYN|p5	6	NaturalKiller_Cell_Cytotoxicity
SHC2	25759	SHC adapt	SCK|SHCB|	19	NaturalKiller_Cell_Cytotoxicity
SHC4	399694	SHC adapt	RaLP|SHCD	15	NaturalKiller_Cell_Cytotoxicity
SHC3	53358	SHC adapt	N-Shc|NSH	9	NaturalKiller_Cell_Cytotoxicity
SHC1	6464	SHC adapt	SHC|SHCA	1	NaturalKiller_Cell_Cytotoxicity
GRB2	2885	growth fac	ASH|EGFRE	17	NaturalKiller_Cell_Cytotoxicity
SOS1	6654	SOS Ras/R	GF1|GGF1|	2	NaturalKiller_Cell_Cytotoxicity
SOS2	6655	SOS Ras/R	NS9|SOS-2	14	NaturalKiller_Cell_Cytotoxicity
HRAS	3265	HRas proto	C-BAS/HA	11	NaturalKiller_Cell_Cytotoxicity
KRAS	3845	KRAS prot	'C-K-RAS|(	12	NaturalKiller_Cell_Cytotoxicity
NRAS	4893	NRAS prot	ALPS4|CMI	1	NaturalKiller_Cell_Cytotoxicity
ARAF	369	A-Raf prot	A-RAF|ARA	X	NaturalKiller_Cell_Cytotoxicity
BRAF	673	B-Raf prot	B-RAF1|B-	7	NaturalKiller_Cell_Cytotoxicity
RAF1	5894	Raf-1 prot	CMD1NN|(	3	NaturalKiller_Cell_Cytotoxicity
MICA	1.01E+08	MHC class	MIC-A|PER	6	NaturalKiller_Cell_Cytotoxicity
MICB	4277	MHC class	PERB11.2	6	NaturalKiller_Cell_Cytotoxicity
ULBP3	79465	UL16 bindi	N2DL-3|NI	6	NaturalKiller_Cell_Cytotoxicity
ULBP2	80328	UL16 bindi	ALCAN-al	6	NaturalKiller_Cell_Cytotoxicity
ULBP1	80329	UL16 bindi	N2DL-1|NI	6	NaturalKiller_Cell_Cytotoxicity
KLRK1	22914	killer cell le	CD314|D12	12	NaturalKiller_Cell_Cytotoxicity
HCST	10870	hematopoi	DAP10|KAF	19	NaturalKiller_Cell_Cytotoxicity
CD48	962	CD48 mole	BCM1|BLA!	1	NaturalKiller_Cell_Cytotoxicity
CD244	51744	CD244 mo	2B4|NAIL|N	1	NaturalKiller_Cell_Cytotoxicity
PPP3CA	5530	protein ph	ACCIID|CA	4	NaturalKiller_Cell_Cytotoxicity
PPP3CB	5532	protein ph	CALNA2|C.	10	NaturalKiller_Cell_Cytotoxicity
PPP3CC	5533	protein ph	CALNA3|C	8	NaturalKiller_Cell_Cytotoxicity
CHP1	11261	calcineurin	CHP|SLC9A	15	NaturalKiller_Cell_Cytotoxicity
PPP3R1	5534	protein ph	CALNB1|CI	2	NaturalKiller_Cell_Cytotoxicity
PPP3R2	5535	protein ph	PPP3RL	9	NaturalKiller_Cell_Cytotoxicity
CHP2	63928	calcineurin	-	16	NaturalKiller_Cell_Cytotoxicity
NFAT5	10725	nuclear fac	NF-AT5|NI	16	NaturalKiller_Cell_Cytotoxicity
NFATC1	4772	nuclear fac	NF-ATC|NI	18	NaturalKiller_Cell_Cytotoxicity
NFATC2	4773	nuclear fac	NFAT1|NFA	20	NaturalKiller_Cell_Cytotoxicity
NFATC3	4775	nuclear fac	NF-AT4c|N	16	NaturalKiller_Cell_Cytotoxicity
NFATC4	4776	nuclear fac	NF-AT3|NI	14	NaturalKiller_Cell_Cytotoxicity
PRKCA	5578	protein kin	AAG6|PKC-	17	NaturalKiller_Cell_Cytotoxicity
PRKCB	5579	protein kin	PKC-beta|I	16	NaturalKiller_Cell_Cytotoxicity
PRKCG	5582	protein kin	PKC-gamn	19	NaturalKiller_Cell_Cytotoxicity
SH2D1B	117157	SH2 doma	EAT2	1	NaturalKiller_Cell_Cytotoxicity
SH2D1A	4068	SH2 doma	DSHP|EBV!	X	NaturalKiller_Cell_Cytotoxicity

IFNGR1	3459	interferon	CD119|IFN	6 NaturalKiller_Cell_Cytotoxicity
IFNGR2	3460	interferon	AF-1|IFGR2	21 NaturalKiller_Cell_Cytotoxicity
IFNA1	3439	interferon	IFL|IFN|IFN	9 NaturalKiller_Cell_Cytotoxicity
IFNA2	3440	interferon	IFN-alpha-	9 NaturalKiller_Cell_Cytotoxicity
IFNA4	3441	interferon	IFN-alpha4	9 NaturalKiller_Cell_Cytotoxicity
IFNA5	3442	interferon	IFN-alpha-	9 NaturalKiller_Cell_Cytotoxicity
IFNA6	3443	interferon	IFN-alphaK	9 NaturalKiller_Cell_Cytotoxicity
IFNA7	3444	interferon	IFN-alphaJ	9 NaturalKiller_Cell_Cytotoxicity
IFNA8	3445	interferon	IFN-alphaB	9 NaturalKiller_Cell_Cytotoxicity
IFNA10	3446	interferon	IFN-alphaC	9 NaturalKiller_Cell_Cytotoxicity
IFNA13	3447	interferon	-	9 NaturalKiller_Cell_Cytotoxicity
IFNA14	3448	interferon	IFN-alphaH	9 NaturalKiller_Cell_Cytotoxicity
IFNA16	3449	interferon	IFN-alpha-	9 NaturalKiller_Cell_Cytotoxicity
IFNA17	3451	interferon	IFN-alphaI	9 NaturalKiller_Cell_Cytotoxicity
IFNA21	3452	interferon	IFN-alphaI	9 NaturalKiller_Cell_Cytotoxicity
IFNB1	3456	interferon	IFB|IFF|IFN-	9 NaturalKiller_Cell_Cytotoxicity
IFNAR1	3454	interferon	AVP|IFN-a	21 NaturalKiller_Cell_Cytotoxicity
IFNAR2	3455	interferon	IFN-R|IFN-	21 NaturalKiller_Cell_Cytotoxicity
TNFSF10	8743	TNF superf	APO2L|Apo	3 NaturalKiller_Cell_Cytotoxicity
TNFRSF10D	8793	TNF recept	CD264|DCR	8 NaturalKiller_Cell_Cytotoxicity
TNFRSF10C	8794	TNF recept	CD263|DCR	8 NaturalKiller_Cell_Cytotoxicity
TNFRSF10B	8795	TNF recept	CD262|DR5	8 NaturalKiller_Cell_Cytotoxicity
TNFRSF10A	8797	TNF recept	APO2|CD2	8 NaturalKiller_Cell_Cytotoxicity
FASLG	356	Fas ligand	ALPS1B|AP	1 NaturalKiller_Cell_Cytotoxicity
FAS	355	Fas cell sur	ALPS1A|AP	10 NaturalKiller_Cell_Cytotoxicity
GZMB	3002	granzyme	C11|CCPI|C	14 NaturalKiller_Cell_Cytotoxicity
PRF1	5551	perforin 1	HPLH2|P1|	10 NaturalKiller_Cell_Cytotoxicity
CASP3	836	caspase 3	CPP32|CPP	4 NaturalKiller_Cell_Cytotoxicity
BID	637	BH3 intera	FP497	22 NaturalKiller_Cell_Cytotoxicity
CD3D	915	CD3d mole	CD3-DELTA	11 TCRsignalingPathway
CD3E	916	CD3e mole	IMD18|T3E	11 TCRsignalingPathway
CD3G	917	CD3g mole	CD3-GAM	11 TCRsignalingPathway
CD247	919	CD247 mo	CD3-ZETA	1 TCRsignalingPathway
CD4	920	CD4 molec	CD4mut	12 TCRsignalingPathway
CD8A	925	CD8a mole	CD8|Leu2|p	2 TCRsignalingPathway
CD8B	926	CD8b mole	CD8B1|LEU	2 TCRsignalingPathway
PTPRC	5788	protein tyr	B220|CD45	1 TCRsignalingPathway
LCK	3932	LCK proto-	IMD22|LSK	1 TCRsignalingPathway
FYN	2534	FYN proto-	SLK|SYN|p5	6 TCRsignalingPathway
ZAP70	7535	zeta chain	ADMIO2|IM	2 TCRsignalingPathway
LCP2	3937	lymphocyt	SLP-76|SLP	5 TCRsignalingPathway
LAT	27040	linker for a	IMD52|LAT	16 TCRsignalingPathway
ITK	3702	IL2 inducib	EMT|LPFS1	5 TCRsignalingPathway
TEC	7006	tec protein	PSCTK4	4 TCRsignalingPathway
NCK1	4690	NCK adapt	NCK|NCKa	3 TCRsignalingPathway
NCK2	8440	NCK adapt	GRB4|NCK	2 TCRsignalingPathway
VAV3	10451	vav guanin	-	1 TCRsignalingPathway
VAV1	7409	vav guanin	VAV	19 TCRsignalingPathway
VAV2	7410	vav guanin	VAV-2	9 TCRsignalingPathway
GRAP2	9402	GRB2 relat	GADS|GRA	22 TCRsignalingPathway
GRB2	2885	growth fac	ASH|EGFRE	17 TCRsignalingPathway
PAK1	5058	p21 (RAC1	IDDMSSD|I	11 TCRsignalingPathway
PAK2	5062	p21 (RAC1	PAK65|PAK	3 TCRsignalingPathway
PAK3	5063	p21 (RAC1	ARA|MRX3X	TCRsignalingPathway
PAK4	10298	p21 (RAC1	-	19 TCRsignalingPathway
PAK6	56924	p21 (RAC1	PAK5	15 TCRsignalingPathway
PAK5	57144	p21 (RAC1	PAK7	20 TCRsignalingPathway
RHOA	387	ras homolo	ARH12|ARI	3 TCRsignalingPathway

CDC42	998	cell divisio	CDC42Hs|(	1	TCRsignalingPathway
PPP3CA	5530	protein ph	ACCIID|CA	4	TCRsignalingPathway
PPP3CB	5532	protein ph	CALNA2|C	10	TCRsignalingPathway
PPP3CC	5533	protein ph	CALNA3|C	8	TCRsignalingPathway
CHP1	11261	calcineurin	CHP|SLC9	15	TCRsignalingPathway
PPP3R1	5534	protein ph	CALNB1|Cl	2	TCRsignalingPathway
PPP3R2	5535	protein ph	PPP3RL	9	TCRsignalingPathway
CHP2	63928	calcineurin	-	16	TCRsignalingPathway
NFAT5	10725	nuclear fac	NF-AT5|NI	16	TCRsignalingPathway
NFATC1	4772	nuclear fac	NF-ATC|NI	18	TCRsignalingPathway
NFATC2	4773	nuclear fac	NFAT1|NF	20	TCRsignalingPathway
NFATC3	4775	nuclear fac	NF-AT4c|N	16	TCRsignalingPathway
NFATC4	4776	nuclear fac	NF-AT3|NI	14	TCRsignalingPathway
SOS1	6654	SOS Ras/R	GF1|GGF1|	2	TCRsignalingPathway
SOS2	6655	SOS Ras/R	NS9|SOS-2	14	TCRsignalingPathway
HRAS	3265	HRas protc	C-BAS/HA	11	TCRsignalingPathway
KRAS	3845	KRAS proto	'C-K-RAS|(	12	TCRsignalingPathway
NRAS	4893	NRAS prot	ALPS4|CMI	1	TCRsignalingPathway
FOS	2353	Fos proto-	AP-1|C-FC	14	TCRsignalingPathway
JUN	3725	Jun proto-	AP-1|AP1|	1	TCRsignalingPathway
CARD11	84433	caspase re	BENTA|BIM	7	TCRsignalingPathway
BCL10	8915	BCL10 imm	CARMEN|C	1	TCRsignalingPathway
MALT1	10892	MALT1 par	IMD12|ML	18	TCRsignalingPathway
CHUK	1147	componen	IKBKA|IKK-	10	TCRsignalingPathway
IKBKB	3551	inhibitor o	IKK-beta|Ik	8	TCRsignalingPathway
IKBKG	8517	inhibitor o	AMCBX1|E	X	TCRsignalingPathway
NFKB1	4790	nuclear fac	CVID12|EB	4	TCRsignalingPathway
RELA	5970	RELA protc	CMCU|NFk	11	TCRsignalingPathway
NFKBIA	4792	NFKB inhib	EDAID2|IKI	14	TCRsignalingPathway
NFKBIB	4793	NFKB inhib	IKBB|TRIP9	19	TCRsignalingPathway
NFKBIE	4794	NFKB inhib	IKBE	6	TCRsignalingPathway
CD28	940	CD28 mole	Tp44	2	TCRsignalingPathway
ICOS	29851	inducible T	AILIM|CD2	2	TCRsignalingPathway
CD40LG	959	CD40 ligan	CD154|CD	X	TCRsignalingPathway
PIK3R5	23533	phosphoin	F730038I1	17	TCRsignalingPathway
PIK3R1	5295	phosphoin	AGM7|GRE	5	TCRsignalingPathway
PIK3R2	5296	phosphoin	MPPH|MPI	19	TCRsignalingPathway
PIK3R3	8503	phosphoin	p55|p55-G	1	TCRsignalingPathway
PIK3CA	5290	phosphatic	CLAPO|CL(	3	TCRsignalingPathway
PIK3CB	5291	phosphatic	P110BETA|	3	TCRsignalingPathway
PIK3CD	5293	phosphatic	APDS|IMD	1	TCRsignalingPathway
PIK3CG	5294	phosphatic	PI3CG|PI3k	7	TCRsignalingPathway
AKT3	10000	AKT serine	MPPH|MPI	1	TCRsignalingPathway
AKT1	207	AKT serine	AKT|CWS6	14	TCRsignalingPathway
AKT2	208	AKT serine	HIHGHH|P	19	TCRsignalingPathway
MAP3K8	1326	mitogen-a	AURA2|CO	10	TCRsignalingPathway
MAP3K14	9020	mitogen-a	FTDCR1B|H	17	TCRsignalingPathway
PDCD1	5133	programm	CD279|PD-	2	TCRsignalingPathway
CTLA4	1493	cytotoxic T	ALPS5|CD|	2	TCRsignalingPathway
PTPN6	5777	protein tyr	HCP|HCPH	12	TCRsignalingPathway
CBLC	23624	Cbl proto-	CBL-3|CBL	19	TCRsignalingPathway
CBL	867	Cbl proto-	C-CBL|CBL	11	TCRsignalingPathway
CBLB	868	Cbl proto-	Cbl-b|Nbla	3	TCRsignalingPathway
IL2	3558	interleukin	IL-2|TCGF|	4	TCRsignalingPathway
IL4	3565	interleukin	BCGF-1|BC	5	TCRsignalingPathway
IL5	3567	interleukin	EDF|IL-5|TI	5	TCRsignalingPathway
IL10	3586	interleukin	CSIF|GVHD	1	TCRsignalingPathway
IFNG	3458	interferon	IFG|IFI	12	TCRsignalingPathway

CSF2	1437	colony stin	CSF|GMCS	5 TCRsignalingPathway
TNF	7124	tumor neci	DIF|TNF-al	6 TCRsignalingPathway
CDK4	1019	cyclin depe	CMM3|PSK	12 TCRsignalingPathway
RASGRP1	10125	RAS guany	CALDAG-(	15 TCRsignalingPathway
PDK1	5163	pyruvate d	-	2 TCRsignalingPathway
PLCG1	5335	phospholip	NCKAP3|PI	20 TCRsignalingPathway
PRKCQ	5588	protein kin	PRKCT|nPK	10 TCRsignalingPathway
TRAC	28755	T cell recep	IMD7|TCRA	14 TCRsignalingPathway
TRAJ1	28754	T cell recep	-	14 TCRsignalingPathway
TRAJ2	28753	T cell recep	-	14 TCRsignalingPathway
TRAJ3	28752	T cell recep	-	14 TCRsignalingPathway
TRAJ4	28751	T cell recep	-	14 TCRsignalingPathway
TRAJ5	28750	T cell recep	-	14 TCRsignalingPathway
TRAJ6	28749	T cell recep	-	14 TCRsignalingPathway
TRAJ7	28748	T cell recep	-	14 TCRsignalingPathway
TRAJ8	28747	T cell recep	-	14 TCRsignalingPathway
TRAJ9	28746	T cell recep	-	14 TCRsignalingPathway
TRAJ10	28745	T cell recep	-	14 TCRsignalingPathway
TRAJ11	28744	T cell recep	-	14 TCRsignalingPathway
TRAJ12	28743	T cell recep	-	14 TCRsignalingPathway
TRAJ13	28742	T cell recep	-	14 TCRsignalingPathway
TRAJ14	28741	T cell recep	-	14 TCRsignalingPathway
TRAJ15	28740	T cell recep	-	14 TCRsignalingPathway
TRAJ16	28739	T cell recep	-	14 TCRsignalingPathway
TRAJ17	28738	T cell recep	-	14 TCRsignalingPathway
TRAJ18	28737	T cell recep	-	14 TCRsignalingPathway
TRAJ19	28736	T cell recep	-	14 TCRsignalingPathway
TRAJ20	28735	T cell recep	-	14 TCRsignalingPathway
TRAJ21	28734	T cell recep	-	14 TCRsignalingPathway
TRAJ22	28733	T cell recep	-	14 TCRsignalingPathway
TRAJ23	28732	T cell recep	-	14 TCRsignalingPathway
TRAJ24	28731	T cell recep	-	14 TCRsignalingPathway
TRAJ25	28730	T cell recep	-	14 TCRsignalingPathway
TRAJ26	28729	T cell recep	-	14 TCRsignalingPathway
TRAJ27	28728	T cell recep	-	14 TCRsignalingPathway
TRAJ28	28727	T cell recep	-	14 TCRsignalingPathway
TRAJ29	28726	T cell recep	-	14 TCRsignalingPathway
TRAJ30	28725	T cell recep	-	14 TCRsignalingPathway
TRAJ31	28724	T cell recep	-	14 TCRsignalingPathway
TRAJ32	28723	T cell recep	-	14 TCRsignalingPathway
TRAJ33	28722	T cell recep	-	14 TCRsignalingPathway
TRAJ34	28721	T cell recep	-	14 TCRsignalingPathway
TRAJ35	28720	T cell recep	-	14 TCRsignalingPathway
TRAJ36	28719	T cell recep	-	14 TCRsignalingPathway
TRAJ37	28718	T cell recep	-	14 TCRsignalingPathway
TRAJ38	28717	T cell recep	-	14 TCRsignalingPathway
TRAJ39	28716	T cell recep	-	14 TCRsignalingPathway
TRAJ40	28715	T cell recep	-	14 TCRsignalingPathway
TRAJ41	28714	T cell recep	-	14 TCRsignalingPathway
TRAJ42	28713	T cell recep	-	14 TCRsignalingPathway
TRAJ43	28712	T cell recep	-	14 TCRsignalingPathway
TRAJ44	28711	T cell recep	-	14 TCRsignalingPathway
TRAJ45	28710	T cell recep	-	14 TCRsignalingPathway
TRAJ46	28709	T cell recep	-	14 TCRsignalingPathway
TRAJ47	28708	T cell recep	-	14 TCRsignalingPathway
TRAJ48	28707	T cell recep	-	14 TCRsignalingPathway
TRAJ49	28706	T cell recep	-	14 TCRsignalingPathway
TRAJ50	28705	T cell recep	-	14 TCRsignalingPathway

TRAJ52	28703	T cell recep	-	14	TCRsignalingPathway
TRAJ53	28702	T cell recep	-	14	TCRsignalingPathway
TRAJ54	28701	T cell recep	-	14	TCRsignalingPathway
TRAJ56	28699	T cell recep	-	14	TCRsignalingPathway
TRAJ57	28698	T cell recep	-	14	TCRsignalingPathway
TRAJ58	28697	T cell recep	-	14	TCRsignalingPathway
TRAJ59	28696	T cell recep	-	14	TCRsignalingPathway
TRAJ61	28694	T cell recep	-	14	TCRsignalingPathway
TRAV1-1	28693	T cell recep	TCRAV1S1	14	TCRsignalingPathway
TRAV1-2	28692	T cell recep	TCRAV1S2	14	TCRsignalingPathway
TRAV2	28691	T cell recep	TCRAV11S	14	TCRsignalingPathway
TRAV3	28690	T cell recep	TCRAV16S	14	TCRsignalingPathway
TRAV4	28689	T cell recep	TCRAV20S	14	TCRsignalingPathway
TRAV5	28688	T cell recep	TCRAV15S	14	TCRsignalingPathway
TRAV7	28686	T cell recep	TCRAV7S1	14	TCRsignalingPathway
TRAV8-1	28685	T cell recep	TCRAV1S1	14	TCRsignalingPathway
TRAV8-2	28684	T cell recep	TCRAV1S5	14	TCRsignalingPathway
TRAV8-3	28683	T cell recep	TCRAV1S4	14	TCRsignalingPathway
TRAV8-4	28682	T cell recep	TCRAV1S2	14	TCRsignalingPathway
TRAV8-6	28680	T cell recep	TCRAV1S3	14	TCRsignalingPathway
TRAV8-7	28679	T cell recep	TCRAV8S7	14	TCRsignalingPathway
TRAV9-1	28678	T cell recep	TCRAV9S1	14	TCRsignalingPathway
TRAV9-2	28677	T cell recep	TCRAV22S	14	TCRsignalingPathway
TRAV10	28676	T cell recep	TCRAV10S	14	TCRsignalingPathway
TRAV12-1	28674	T cell recep	TCRAV12S	14	TCRsignalingPathway
TRAV12-2	28673	T cell recep	TCRAV12S	14	TCRsignalingPathway
TRAV12-3	28672	T cell recep	TCRAV12S	14	TCRsignalingPathway
TRAV13-1	28671	T cell recep	TCRAV13S	14	TCRsignalingPathway
TRAV13-2	28670	T cell recep	TCRAV13S	14	TCRsignalingPathway
TRAV14DV	28669	T cell recep	TCRAV6S1	14	TCRsignalingPathway
TRAV16	28667	T cell recep	TCRAV16S	14	TCRsignalingPathway
TRAV17	28666	T cell recep	TCRAV17S	14	TCRsignalingPathway
TRAV18	28665	T cell recep	TCRAV18S	14	TCRsignalingPathway
TRAV19	28664	T cell recep	TCRAV12S	14	TCRsignalingPathway
TRAV20	28663	T cell recep	TCRAV20S	14	TCRsignalingPathway
TRAV21	28662	T cell recep	TCRAV21S	14	TCRsignalingPathway
TRAV22	28661	T cell recep	TCRAV13S	14	TCRsignalingPathway
TRAV23DV	28660	T cell recep	TCRAV17S	14	TCRsignalingPathway
TRAV24	28659	T cell recep	TCRAV18S	14	TCRsignalingPathway
TRAV25	28658	T cell recep	TCRAV25S	14	TCRsignalingPathway
TRAV26-1	28657	T cell recep	TCRAV26S	14	TCRsignalingPathway
TRAV26-2	28656	T cell recep	TCRAV26S	14	TCRsignalingPathway
TRAV27	28655	T cell recep	TCRAV10S	14	TCRsignalingPathway
TRAV29DV	28653	T cell recep	TCRA|TCRA	14	TCRsignalingPathway
TRAV30	28652	T cell recep	TCRAV29S	14	TCRsignalingPathway
TRAV34	28648	T cell recep	TCRAV26S	14	TCRsignalingPathway
TRAV35	28647	T cell recep	TCRAV25S	14	TCRsignalingPathway
TRAV36DV	28646	T cell recep	TCRAV28S	14	TCRsignalingPathway
TRAV38-1	28644	T cell recep	TCRAV14S	14	TCRsignalingPathway
TRAV38-2I	28643	T cell recep	TCRAV14S	14	TCRsignalingPathway
TRAV39	28642	T cell recep	TCRAV27S	14	TCRsignalingPathway
TRAV40	28641	T cell recep	TCRAV31S	14	TCRsignalingPathway
TRAV41	28640	T cell recep	TCRAV19S	14	TCRsignalingPathway
TRBC1	28639	T cell recep	BV05S1J2.2	7	TCRsignalingPathway
TRBC2	28638	T cell recep	TCRBC2	7	TCRsignalingPathway
TRBD1	28637	T cell recep	TCRBD1	7	TCRsignalingPathway
TRBD2	28636	T cell recep	TCRBD2	7	TCRsignalingPathway
TRBJ1-1	28635	T cell recep	TCRBJ1S1|T	7	TCRsignalingPathway

TRBJ1-2	28634	T cell recep	TCRBJ1S2|	7	TCRsignalingPathway
TRBJ1-3	28633	T cell recep	TCRBJ1S3|	7	TCRsignalingPathway
TRBJ1-4	28632	T cell recep	TCRBJ1S4|	7	TCRsignalingPathway
TRBJ1-5	28631	T cell recep	TCRBJ1S5|	7	TCRsignalingPathway
TRBJ1-6	28630	T cell recep	TCRBJ1S6|	7	TCRsignalingPathway
TRBJ2-1	28629	T cell recep	TCRBJ2S1|	7	TCRsignalingPathway
TRBJ2-2	28628	T cell recep	TCRBJ2S2|	7	TCRsignalingPathway
TRBJ2-3	28626	T cell recep	TCRBJ2S3|	7	TCRsignalingPathway
TRBJ2-4	28625	T cell recep	TCRBJ2S4|	7	TCRsignalingPathway
TRBJ2-5	28624	T cell recep	TCRBJ2S5|	7	TCRsignalingPathway
TRBJ2-6	28623	T cell recep	TCRBJ2S6|	7	TCRsignalingPathway
TRBJ2-7	28622	T cell recep	TCRBJ2S7|	7	TCRsignalingPathway
TRBV2	28620	T cell recep	TCRBV22S	7	TCRsignalingPathway
TRBV3-1	28619	T cell recep	TCRBV3S1|	7	TCRsignalingPathway
TRBV4-1	28617	T cell recep	BV07S1J2.	7	TCRsignalingPathway
TRBV4-2	28616	T cell recep	TCRBV4S2|	7	TCRsignalingPathway
TRBV4-3	28615	T cell recep	TCRBV4S3|	7	TCRsignalingPathway
TRBV5-1	28614	T cell recep	TCRBV5S1|	7	TCRsignalingPathway
TRBV5-4	28611	T cell recep	TCRBV5S4|	7	TCRsignalingPathway
TRBV5-5	28610	T cell recep	TCRBV5S3	7	TCRsignalingPathway
TRBV5-6	28609	T cell recep	TCRBV5S2|	7	TCRsignalingPathway
TRBV5-7	28608	T cell recep	TCRBV5S7|	7	TCRsignalingPathway
TRBV5-8	28607	T cell recep	TCRBV5S4	7	TCRsignalingPathway
TRBV6-1	28606	T cell recep	TCRBV13S	7	TCRsignalingPathway
TRBV6-2	28605	T cell recep	TCRBV13S	7	TCRsignalingPathway
TRBV6-3	28604	T cell recep	TCRBV13S	7	TCRsignalingPathway
TRBV6-4	28603	T cell recep	TCRBV13S	7	TCRsignalingPathway
TRBV6-5	28602	T cell recep	TCRBV13S	7	TCRsignalingPathway
TRBV6-6	28601	T cell recep	TCRBV13S	7	TCRsignalingPathway
TRBV6-7	28600	T cell recep	TCRBV13S	7	TCRsignalingPathway
TRBV6-8	28599	T cell recep	TCRBV13S	7	TCRsignalingPathway
TRBV6-9	28598	T cell recep	TCRBV13S	7	TCRsignalingPathway
TRBV7-2	28596	T cell recep	TCRBV6S5	7	TCRsignalingPathway
TRBV7-3	28595	T cell recep	TCRBV6S1	7	TCRsignalingPathway
TRBV7-4	28594	T cell recep	TCRBV6S8	7	TCRsignalingPathway
TRBV7-6	28592	T cell recep	TCRBV6S3	7	TCRsignalingPathway
TRBV7-7	28591	T cell recep	TCRBV6S6	7	TCRsignalingPathway
TRBV7-8	28590	T cell recep	TCRBV6S2	7	TCRsignalingPathway
TRBV7-9	28589	T cell recep	TCRB|TCRE	7	TCRsignalingPathway
TRBV9	28586	T cell recep	TCRBV1S1	7	TCRsignalingPathway
TRBV10-1	28585	T cell recep	TCRBV10S	7	TCRsignalingPathway
TRBV10-2	28584	T cell recep	TCRBV10S	7	TCRsignalingPathway
TRBV10-3	28583	T cell recep	TCRBV10S	7	TCRsignalingPathway
TRBV11-1	28582	T cell recep	TCRBV11S	7	TCRsignalingPathway
TRBV11-2	28581	T cell recep	TCRBV11S	7	TCRsignalingPathway
TRBV11-3	28580	T cell recep	TCRBV11S	7	TCRsignalingPathway
TRBV12-3	28577	T cell recep	TCRBV12S	7	TCRsignalingPathway
TRBV12-4	28576	T cell recep	TCRBV12S	7	TCRsignalingPathway
TRBV12-5	28575	T cell recep	TCRBV12S	7	TCRsignalingPathway
TRBV13	28574	T cell recep	TCRBV13S	7	TCRsignalingPathway
TRBV14	28573	T cell recep	TCRBV14S	7	TCRsignalingPathway
TRBV15	28572	T cell recep	TCRBV15S	7	TCRsignalingPathway
TRBV16	28571	T cell recep	BV25S1J1.	7	TCRsignalingPathway
TRBV17	28570	T cell recep	TCRBV17S	7	TCRsignalingPathway
TRBV18	28569	T cell recep	TCRBV18S	7	TCRsignalingPathway
TRBV19	28568	T cell recep	TCRBV17S	7	TCRsignalingPathway
TRBV20-1	28567	T cell recep	TCRBV20S	7	TCRsignalingPathway
TRBV24-1	28563	T cell recep	TCRBV15S	7	TCRsignalingPathway

TRBV25-1	28562	T cell recep	TCRBV11S	7	TCRsignalingPathway
TRBV27	28560	T cell recep	TCRBV14S	7	TCRsignalingPathway
TRBV28	28559	T cell recep	TCRBV28S	7	TCRsignalingPathway
TRBV29-1	28558	T cell recep	TCRBV29S	7	TCRsignalingPathway
TRBV30	28557	T cell recep	TCRBV20S	7	TCRsignalingPathway
TRDC	28526	T cell recep	TCRD	14	TCRsignalingPathway
TRDD1	28525	T cell recep	-	14	TCRsignalingPathway
TRDD2	28524	T cell recep	-	14	TCRsignalingPathway
TRDD3	28523	T cell recep	TCRD	14	TCRsignalingPathway
TRDJ1	28522	T cell recep	TCRD	14	TCRsignalingPathway
TRDJ2	28521	T cell recep	-	14	TCRsignalingPathway
TRDJ3	28520	T cell recep	-	14	TCRsignalingPathway
TRDJ4	28519	T cell recep	-	14	TCRsignalingPathway
TRDV1	28518	T cell recep	hDV101S1	14	TCRsignalingPathway
TRDV2	28517	T cell recep	hDV102S1	14	TCRsignalingPathway
TRDV3	28516	T cell recep	hDV103S1	14	TCRsignalingPathway
TRGV9	6983	T cell recep	TCRGV9|T	7	TCRsignalingPathway
TRGV8	6982	T cell recep	TCRGV8|V	7	TCRsignalingPathway
TRGV5	6978	T cell recep	TCRGV5|V	7	TCRsignalingPathway
TRGV4	6977	T cell recep	TCRGV4|V	7	TCRsignalingPathway
TRGV3	6976	T cell recep	TCRGV3|V	7	TCRsignalingPathway
TRGV2	6974	T cell recep	TCRGV2|V	7	TCRsignalingPathway
TRGJP2	6972	T cell recep	JP2|TCRGJ	7	TCRsignalingPathway
TRGJP1	6971	T cell recep	JP1|TCRGJ	7	TCRsignalingPathway
TRGJP	6970	T cell recep	JP|TCRGJP	7	TCRsignalingPathway
TRGJ2	6969	T cell recep	J2|TCRGJ2	7	TCRsignalingPathway
TRGJ1	6968	T cell recep	J1|TCRGJ1	7	TCRsignalingPathway
TRGC2	6967	T cell recep	TCRGC2|T	7	TCRsignalingPathway
TRGC1	6966	T cell recep	C1|TCRG|T	7	TCRsignalingPathway
TRAV6	6956	T cell recep	TCRAV5S1	14	TCRsignalingPathway
BMP1	649	bone morp	OI13|PCOL	8	TGFb_Family_Member
BMP10	27302	bone morp	-	2	TGFb_Family_Member
BMP15	9210	bone morp	GDF9B|OD	X	TGFb_Family_Member
BMP2	650	bone morp	BDA2|BMP	20	TGFb_Family_Member
BMP3	651	bone morp	BMP-3A	4	TGFb_Family_Member
BMP4	652	bone morp	BMP2B|BM	14	TGFb_Family_Member
BMP5	653	bone morp	-	6	TGFb_Family_Member
BMP6	654	bone morp	VGR|VGR1	6	TGFb_Family_Member
BMP7	655	bone morp	OP-1	20	TGFb_Family_Member
BMP8A	353500	bone morp	OP-2	1	TGFb_Family_Member
BMP8B	656	bone morp	BMP8|OP2	1	TGFb_Family_Member
GDF1	2657	growth dif	CERS1|CHT	19	TGFb_Family_Member
GDF10	2662	growth dif	BIP|BMP-3	10	TGFb_Family_Member
GDF11	10220	growth dif	BMP-11|B	12	TGFb_Family_Member
GDF15	9518	growth dif	GDF-15|M	19	TGFb_Family_Member
GDF2	2658	growth dif	BMP-9|BM	10	TGFb_Family_Member
GDF3	9573	growth dif	KFS3|MCO	12	TGFb_Family_Member
GDF5	8200	growth dif	BDA1C|BM	20	TGFb_Family_Member
GDF6	392255	growth dif	BMP-13|B	8	TGFb_Family_Member
GDF7	151449	growth dif	BMP12	2	TGFb_Family_Member
GDF9	2661	growth dif	POF14	5	TGFb_Family_Member
GDNF	2668	glial cell de	ATF|ATF1|	5	TGFb_Family_Member
INHA	3623	inhibin sub	-	2	TGFb_Family_Member
INHBA	3624	inhibin sub	EDF|FRP	7	TGFb_Family_Member
INHBB	3625	inhibin sub	-	2	TGFb_Family_Member
INHBC	3626	inhibin sub	IHBC	12	TGFb_Family_Member
INHBE	83729	inhibin sub	-	12	TGFb_Family_Member
LEFTY1	10637	left-right c	LEFTB|LEFT	1	TGFb_Family_Member

LEFTY2	7044	left-right c	EBAF|LEFT/	1	TGFb_Family_Member
NODAL	4838	nodal grov	HTX5	10	TGFb_Family_Member
TGFB1	7040	transformi	CED|DPD1	19	TGFb_Family_Member
TGFB2	7042	transformi	G-TSF|LDS	1	TGFb_Family_Member
TGFB3	7043	transformi	ARVD|ARV	14	TGFb_Family_Member
ACVR1B	91	activin A re	ACTRIB|AC	12	TGFb_Family_Member_Receptor
ACVR1C	130399	activin A re	ACVRLK7|/	2	TGFb_Family_Member_Receptor
ACVR2A	92	activin A re	ACTRII|ACV	2	TGFb_Family_Member_Receptor
ACVR2B	93	activin A re	ACTRIIB|Ac	3	TGFb_Family_Member_Receptor
ACVRL1	94	activin A re	ACVRLK1|/	12	TGFb_Family_Member_Receptor
AMHR2	269	anti-Mulle	AMHR|MIS	12	TGFb_Family_Member_Receptor
BMPR1A	657	bone morp	10q23del|/	10	TGFb_Family_Member_Receptor
BMPR1B	658	bone morp	ALK-6|ALK	4	TGFb_Family_Member_Receptor
BMPR2	659	bone morp	BMPR-II|BI	2	TGFb_Family_Member_Receptor
TGFBR1	7046	transformi	AAT5|ACVI	9	TGFb_Family_Member_Receptor
TGFBR2	7048	transformi	AAT3|FAA3	3	TGFb_Family_Member_Receptor
TGFBR3	7049	transformi	BGCAN|be	1	TGFb_Family_Member_Receptor
TNFRSF11I	4982	TNF recept	OCIF|OPG|	8	TNF_Family_Members
TNFSF10	8743	TNF super	APO2L|Apo	3	TNF_Family_Members
TNFSF11	8600	TNF super	CD254|OD	13	TNF_Family_Members
TNFSF12	8742	TNF super	APO3L|DR3	17	TNF_Family_Members
TNFSF13	8741	TNF super	APRIL|CD2	17	TNF_Family_Members
TNFSF13B	10673	TNF super	BAFF|BLYS|	13	TNF_Family_Members
TNFSF14	8740	TNF super	CD258|HVI	19	TNF_Family_Members
TNFSF15	9966	TNF super	TL1|TL1A|T	9	TNF_Family_Members
TNFSF18	8995	TNF super	AITRL|GITR	1	TNF_Family_Members
TNFSF4	7292	TNF super	CD134L|CD	1	TNF_Family_Members
TNFSF8	944	TNF super	CD153|CD3	9	TNF_Family_Members
TNFSF9	8744	TNF super	4-1BB-L|C	19	TNF_Family_Members
TNFRSF10I	8795	TNF recept	CD262|DR5	8	TNF_Family_Members_Receptors
TNFRSF10(	8794	TNF recept	CD263|DCI	8	TNF_Family_Members_Receptors
TNFRSF10I	8793	TNF recept	CD264|DCI	8	TNF_Family_Members_Receptors
TNFRSF11/	8792	TNF recept	CD265|FEC	18	TNF_Family_Members_Receptors
TNFRSF12/	51330	TNF recept	CD266|FN1	16	TNF_Family_Members_Receptors
TNFRSF13I	23495	TNF recept	CD267|CVI	17	TNF_Family_Members_Receptors
TNFRSF13(	115650	TNF recept	BAFF-R|BA	22	TNF_Family_Members_Receptors
TNFRSF14	8764	TNF recept	ATAR|CD2	1	TNF_Family_Members_Receptors
TNFRSF17	608	TNF recept	BCM|BCM/	16	TNF_Family_Members_Receptors
TNFRSF18	8784	TNF recept	AITR|CD35	1	TNF_Family_Members_Receptors
TNFRSF19	55504	TNF recept	TAJ|TAJ-al	13	TNF_Family_Members_Receptors
TNFRSF1A	7132	TNF recept	CD120a|FP	12	TNF_Family_Members_Receptors
TNFRSF1B	7133	TNF recept	CD120b|TE	1	TNF_Family_Members_Receptors
TNFRSF21	27242	TNF recept	BM-018|CI	6	TNF_Family_Members_Receptors
TNFRSF25	8718	TNF recept	APO-3|DD	1	TNF_Family_Members_Receptors
TNFRSF4	7293	TNF recept	ACT35|CD1	1	TNF_Family_Members_Receptors
TNFRSF6B	8771	TNF recept	DCR3|DJ58	20	TNF_Family_Members_Receptors
TNFRSF8	943	TNF recept	CD30|D1S1	1	TNF_Family_Members_Receptors
TNFRSF9	3604	TNF recept	4-1BB|CD1	1	TNF_Family_Members_Receptors
