# Supplementary material for: The Potential Diagnostic Value of Immune-Related Genes in Interstitial Fibrosis and Tubular Atrophy after Kidney Transplantation
Source: J Immunol Res. 2022 Jun 17;2022:7212852. doi: 10.1155/2022/7212852 (PMC9232312; doi:10.1155/2022/7212852)
Supplement: Supplementary Materials — Supplementary Figure 1: GSEA enrichment analysis of the IF/TA group. Supplementary Figure 2: correlation analysis between ANGPTL3 and differentially expressed immune infiltrating cells. Supplementary Figure 3: correlation analysis between APOH and differentially expressed immune infiltrating cells. Supplementary Figure 4: correlation analysis between EGF and differentially expressed immune infiltrating cells. Supplementary Figure 5: correlation analysis between FCGR2B and differentially expressed immune infiltrating cells. Supplementary Figure 6: correlation analysis between HLA-DQA2 and differentially expressed immune infiltrating cells. Supplementary Figure 7: correlation analysis between LTF and differentially expressed immune infiltrating cells. Supplementary Figure 8: IPA analysis shows the interaction network of diagnostic genes: EGF and LTF (8A), ANGPTL3 (8B), FCGR2B and APOH (8C), and HLA-DQA2 (8D). Merged the above four independent networks to comprehensively analyze the interaction of diagnostic genes (8E). Supplementary Table 1: immune-related genes. Supplementary Table 2: KEGG pathway in normal group. Supplementary Table 3: pathway of ANGPTL3 gene. Supplementary Table 4: pathway of APOH gene. Supplementary Table 5: pathway of EGF gene. Supplementary Table 6: ingenuity canonical pathways. Supplementary Table 7: category. [file 7212852.f1.zip › 7212852.f1/supplementary table10.pdf]

ID	Descriptior	setSize	enrichmen	NES	pvalue	p.adjust	qvalues	rank
hsa05200	Pathways i	492	0.295736	1.616631	0.001095	0.00591	0.003437	4772
hsa05168	Herpes sim	427	0.439114	2.36653	0.001116	0.00591	0.003437	5822
hsa05165	Human pa	316	0.305755	1.602136	0.001172	0.00591	0.003437	3149
hsa05014	Amyotroph	305	0.289044	1.510376	0.001176	0.00591	0.003437	3896
hsa05132	Salmonella	244	0.456583	2.341669	0.001202	0.00591	0.003437	4126
hsa04144	Endocytosi	235	0.312757	1.598631	0.001205	0.00591	0.003437	3894
hsa04060	Cytokine-c	249	0.385874	1.982046	0.001206	0.00591	0.003437	1290
hsa05163	Human cyt	210	0.435654	2.200503	0.001238	0.00591	0.003437	3471
hsa05171	Coronaviru	195	0.529638	2.662479	0.001242	0.00591	0.003437	3149
hsa05166	Human T-(	211	0.504874	2.549485	0.001244	0.00591	0.003437	2675
hsa05131	Shigellosis	209	0.41623	2.096019	0.001247	0.00591	0.003437	3505
hsa05169	Epstein-Ba	194	0.584219	2.930995	0.001252	0.00591	0.003437	2796
hsa05170	Human im	191	0.431521	2.157866	0.001266	0.00591	0.003437	2288
hsa05130	Pathogenic	184	0.471388	2.346131	0.001269	0.00591	0.003437	3894
hsa04062	Chemokine	177	0.506104	2.511418	0.001271	0.00591	0.003437	2914
hsa05152	Tuberculos	171	0.530065	2.618164	0.001277	0.00591	0.003437	2682
hsa05167	Kaposi sarc	175	0.467968	2.316909	0.001284	0.00591	0.003437	3471
hsa04141	Protein prc	157	0.374081	1.823087	0.001294	0.00591	0.003437	4594
hsa05161	Hepatitis B	157	0.434555	2.11781	0.001294	0.00591	0.003437	3062
hsa04218	Cellular ser	148	0.383753	1.856578	0.001295	0.00591	0.003437	4341
hsa05202	Transcripti	158	0.384418	1.87462	0.001297	0.00591	0.003437	4484
hsa03013	RNA transp	152	0.494621	2.404478	0.0013	0.00591	0.003437	5149
hsa04530	Tight junct	161	0.346522	1.692651	0.0013	0.00591	0.003437	3451
hsa04621	NOD-like r	161	0.524042	2.559783	0.0013	0.00591	0.003437	2366
hsa05164	Influenza A	162	0.563871	2.758962	0.0013	0.00591	0.003437	3149
hsa05203	Viral carcir	161	0.494256	2.414285	0.0013	0.00591	0.003437	3114
hsa04110	Cell cycle	117	0.432473	2.016235	0.001309	0.00591	0.003437	4464
hsa04071	Sphingolip	116	0.360863	1.68211	0.001311	0.00591	0.003437	3471
hsa05135	Yersinia inf	130	0.496926	2.354542	0.001311	0.00591	0.003437	3505
hsa03040	Spliceosom	128	0.519514	2.456275	0.001312	0.00591	0.003437	4754
hsa04145	Phagosom	144	0.604152	2.908124	0.001312	0.00591	0.003437	3451
hsa05160	Hepatitis C	144	0.452304	2.177194	0.001312	0.00591	0.003437	3179
hsa05162	Measles	132	0.510052	2.419595	0.001314	0.00591	0.003437	3198
hsa05418	Fluid shear	132	0.412968	1.959045	0.001314	0.00591	0.003437	4712
hsa04217	Necroptos	124	0.485921	2.287571	0.001316	0.00591	0.003437	2832
hsa03010	Ribosome	110	0.400371	1.852194	0.001318	0.00591	0.003437	4820
hsa04611	Platelet act	114	0.383861	1.779203	0.001319	0.00591	0.003437	3471
hsa04380	Osteoclast	122	0.534672	2.508196	0.001321	0.00591	0.003437	4214
hsa04630	JAK-STAT s	139	0.347668	1.662914	0.001321	0.00591	0.003437	4429
hsa04210	Apoptosis	129	0.468533	2.214862	0.001323	0.00591	0.003437	3494
hsa04650	Natural kill	119	0.554374	2.585454	0.001326	0.00591	0.003437	1731
hsa04668	TNF signal	108	0.542696	2.496026	0.001326	0.00591	0.003437	4214
hsa05145	Toxoplasm	108	0.571434	2.628201	0.001326	0.00591	0.003437	2675
hsa04514	Cell adhesi	136	0.537185	2.559873	0.001328	0.00591	0.003437	1450
hsa04670	Leukocyte	105	0.486268	2.220208	0.00133	0.00591	0.003437	3599
hsa04933	AGE-RAGE	99	0.454297	2.042336	0.00135	0.00591	0.003437	4214
hsa04666	Fc gamma	95	0.473854	2.112641	0.001351	0.00591	0.003437	4214
hsa04659	Th17 cell d	96	0.61612	2.751636	0.001353	0.00591	0.003437	3859
hsa04660	T cell recep	96	0.493805	2.205368	0.001353	0.00591	0.003437	4422
hsa05142	Chagas dis	97	0.506993	2.267071	0.001357	0.00591	0.003437	4214
hsa04064	NF-kappa	98	0.607275	2.720501	0.001362	0.00591	0.003437	2648
hsa04620	Toll-like re	98	0.544364	2.438668	0.001362	0.00591	0.003437	3149
hsa04625	C-type lec	98	0.450391	2.017684	0.001362	0.00591	0.003437	4341
hsa05146	Amoebiasi	98	0.49209	2.20449	0.001362	0.00591	0.003437	3481
hsa04640	Hematopo	91	0.65766	2.907763	0.001366	0.00591	0.003437	1129
hsa04061	Viral prote	87	0.617044	2.715911	0.001376	0.00591	0.003437	1224
hsa05222	Small cell l	90	0.448008	1.978366	0.001376	0.00591	0.003437	3481

hsa05235	PD-L1 exp	87	0.506373	2.228794	0.001376	0.00591	0.003437	4214
hsa05323	Rheumatoi	90	0.631352	2.787997	0.001376	0.00591	0.003437	3272
hsa04657	IL-17 signa	86	0.421015	1.848356	0.001377	0.00591	0.003437	4364
hsa04658	Th1 and Th	86	0.612236	2.687863	0.001377	0.00591	0.003437	1611
hsa04662	B cell recep	79	0.487028	2.111269	0.001379	0.00591	0.003437	3042
hsa04610	Compleme	77	0.477925	2.062992	0.001397	0.00591	0.003437	3678
hsa04612	Antigen pr	77	0.716974	3.094862	0.001397	0.00591	0.003437	2796
hsa03018	RNA degra	69	0.447016	1.900848	0.001403	0.00591	0.003437	2985
hsa05100	Bacterial in	67	0.453939	1.919717	0.00141	0.00591	0.003437	3894
hsa05133	Pertussis	71	0.584187	2.489862	0.001414	0.00591	0.003437	2493
hsa03008	Ribosome	73	0.441636	1.88506	0.00142	0.00591	0.003437	4792
hsa05150	Staphylocc	73	0.697329	2.976449	0.00142	0.00591	0.003437	1032
hsa05140	Leishmania	74	0.697124	2.966292	0.001427	0.00591	0.003437	2826
hsa05416	Viral myoc	58	0.772023	3.151723	0.001451	0.00591	0.003437	826
hsa04623	Cytosolic D	56	0.436757	1.769941	0.001464	0.00591	0.003437	2752
hsa05321	Inflammato	55	0.695687	2.806165	0.001466	0.00591	0.003437	2769
hsa05134	Legionellos	57	0.592671	2.407956	0.001468	0.00591	0.003437	3325
hsa05144	Malaria	47	0.657733	2.582129	0.001481	0.00591	0.003437	2227
hsa05320	Autoimmu	44	0.7575	2.938145	0.001484	0.00591	0.003437	506
hsa03050	Proteasom	43	0.598605	2.296265	0.001493	0.00591	0.003437	3896
hsa05322	Systemic lu	48	0.769374	3.024588	0.001495	0.00591	0.003437	2675
hsa04940	Type I diab	41	0.744705	2.818062	0.001511	0.00591	0.003437	506
hsa05332	Graft-versu	41	0.831381	3.146058	0.001511	0.00591	0.003437	506
hsa04672	Intestinal in	42	0.663913	2.520162	0.001513	0.00591	0.003437	2675
hsa05340	Primary im	34	0.651069	2.36506	0.001536	0.00591	0.003437	1553
hsa03030	DNA replic	36	0.515901	1.902436	0.001538	0.00591	0.003437	5023
hsa05143	African tryp	33	0.569479	2.056526	0.00155	0.00591	0.003437	2227
hsa05330	Allograft re	33	0.833867	3.011296	0.00155	0.00591	0.003437	506
hsa05310	Asthma	23	0.813515	2.671741	0.001597	0.005987	0.003482	445
hsa03060	Protein exp	20	0.643217	2.032053	0.001608	0.005987	0.003482	3530
hsa04151	PI3K-Akt s	321	0.279877	1.468177	0.002342	0.008623	0.005014	3481
hsa00340	Histidine m	21	-0.59772	-2.09438	0.002591	0.00937	0.005449	6424
hsa04140	Autophagy	132	0.342328	1.623943	0.002628	0.00937	0.005449	4881
hsa04744	Phototrans	20	-0.56767	-1.92848	0.002632	0.00937	0.005449	5167
hsa00410	beta-Alani	28	-0.52315	-1.99016	0.002762	0.009729	0.005658	3621
hsa04975	Fat digesti	35	-0.47234	-1.90328	0.002793	0.009731	0.005659	4853
hsa04742	Taste trans	47	-0.46143	-2.0009	0.003058	0.010541	0.00613	4237
hsa04740	Olfactory t	73	-0.38819	-1.88289	0.003356	0.011445	0.006656	5167
hsa04260	Cardiac mu	68	-0.41188	-1.94305	0.003497	0.011801	0.006863	6039
hsa04810	Regulation	204	0.302093	1.521137	0.003713	0.012165	0.007075	3451
hsa05205	Proteoglyc	199	0.293673	1.474219	0.003727	0.012165	0.007075	4264
hsa00190	Oxidative p	98	-0.46061	-2.3563	0.003731	0.012165	0.007075	4850
hsa04015	Rap1 signa	194	0.311147	1.561005	0.003755	0.012165	0.007075	4399
hsa04723	Retrograde	121	-0.31956	-1.68644	0.004	0.012832	0.007462	4537
hsa04115	p53 signali	72	0.394005	1.677191	0.004267	0.013555	0.007883	5007
hsa04714	Thermoger	189	-0.31008	-1.76513	0.004717	0.014838	0.008629	5024
hsa04510	Focal adhe	196	0.300007	1.505764	0.004981	0.015519	0.009025	4214
hsa04020	Calcium sig	205	-0.24901	-1.43233	0.005102	0.015743	0.009156	2744
hsa04120	Ubiquitin r	132	0.328327	1.557523	0.005256	0.015937	0.009268	6151
hsa04977	Vitamin dig	20	-0.53178	-1.80656	0.005263	0.015937	0.009268	3450
hsa05033	Nicotine ad	25	-0.45658	-1.68548	0.005362	0.016086	0.009355	5781
hsa04080	Neuroactiv	239	-0.33612	-1.97887	0.00578	0.017182	0.009992	3536
hsa05220	Chronic my	76	0.382513	1.646476	0.006964	0.020512	0.011928	4214
hsa04622	RIG-I-like r	64	0.401198	1.67186	0.007163	0.020782	0.012086	3113
hsa04664	Fc epsilon	63	0.407044	1.685629	0.007184	0.020782	0.012086	4214
hsa05020	Prion disea	228	0.285526	1.449606	0.008589	0.024627	0.014322	3938
hsa05206	MicroRNAs	163	0.306973	1.504104	0.009103	0.025871	0.015045	4452
hsa05210	Colorectal	85	0.337945	1.479271	0.009736	0.027429	0.015951	4694

hsa04721	Synaptic ve	68	-0.31423	-1.4824	0.01049	0.029227	0.016997	3471
hsa05017	Spinocereb	123	0.3106	1.458229	0.010554	0.029227	0.016997	5712
hsa05212	Pancreatic	75	0.358626	1.535824	0.01122	0.030808	0.017916	2497
hsa00600	Sphingolip	44	0.422452	1.638581	0.011869	0.032317	0.018794	3771
hsa03015	mRNA surv	88	0.339113	1.49185	0.012465	0.033657	0.019573	4865
hsa04520	Adherens j	69	0.36185	1.538698	0.014025	0.037555	0.02184	3891
hsa04950	Maturity or	19	-0.52076	-1.74736	0.015584	0.041388	0.024069	3153

leading_edcore_enrichment
tags=38%, 7852/6772/5880/6775/330/3575/3600/1439/10161/3560/1438/83593/4790/3601/4792/
tags=55%, 3118/3113/3122/3108/3106/3115/3134/3135/3119/3126/3133/3109/3107/6352/3117/6
tags=28%, 3106/3134/3135/3133/3107/3105/6772/3659/10379/4790/1282/1284/355/4599/5734/5
tags=33%, 834/10376/84790/10189/71/7133/10010/2876/5687/10762/5216/79139/5879/79902/57
tags=44%, 5788/834/330/929/197259/7097/10376/6188/29108/84790/837/4790/302/71/4792/307
tags=32%, 3106/3134/3135/3133/7852/3107/3105/1234/3560/10109/10096/3310/387/58533/829/
tags=23%, 729230/7852/6352/6347/958/10673/3587/6363/3627/1234/1524/6373/3575/3600/4283
tags=39%, 3106/3134/3135/3133/7852/3107/6352/6347/3105/5880/3587/1234/6890/567/6351/47
tags=48%, 834/1536/6347/6772/3627/713/10379/7097/2212/6188/6175/4790/4792/6187/714/512
tags=38%, 3118/3113/3122/3108/3106/3115/3134/3135/3119/3126/3689/3133/3109/3107/3117/3
tags=39%, 834/6352/3059/929/29108/837/4790/71/4792/10109/10096/92610/3553/387/960/1145
tags=48%, 3118/3113/3122/3108/3106/3115/3134/3135/3119/3126/3133/3109/3107/3117/3105/6
tags=31%, 3106/3134/3135/60489/3133/7852/3107/3105/5880/919/1234/200316/915/6890/567/7
tags=45%, 834/3059/10376/2212/6188/29108/84790/837/4790/71/4542/4792/3071/10109/10096/
tags=41%, 729230/7852/6352/6347/6772/5880/1794/6363/3627/1234/1524/6373/4283/3702/3055
tags=39%, 3118/3113/3122/3108/3115/1520/3119/3126/3689/3109/3117/11151/6772/972/3587/9
tags=40%, 3106/3134/3135/3133/3107/3105/6772/942/1234/10379/3055/4067/4790/4792/3459/5
tags=40%, 3310/10130/6746/79139/23190/7991/6185/81567/9451/54431/5034/3703/27248/7184/
tags=38%, 6772/6775/7097/4790/4792/7534/5579/4609/4773/23586/7529/355/6774/10971/5111/
tags=40%, 3106/3134/3135/3133/3107/3105/83593/4790/4609/4773/6237/5728/3576/7048/5594/
tags=42%, 958/942/330/929/3002/3560/3684/4790/2209/597/2313/64919/4609/2120/64332/1436
tags=56%, 1973/6613/10189/10762/1207/3646/5901/8664/79902/4928/79023/9775/1967/23165/7
tags=30%, 911/3059/913/10376/912/84790/71/10109/10096/387/5111/7408/9076/5879/87/10092
tags=40%, 834/1536/6352/6347/6772/114769/2633/330/7128/115362/10379/3428/29108/837/263
tags=50%, 3118/3113/3122/3108/834/3115/3119/3126/3109/6352/3117/6347/6772/3627/10379/3
tags=43%, 3106/3134/3135/3133/3107/3105/7533/1234/10379/4067/4790/4792/7534/5366/387/7
tags=48%, 7533/7534/4609/10459/7529/4176/9184/10971/10926/5111/8317/4172/9232/9126/888
tags=34%, 5880/2207/4790/5579/2205/387/5728/10672/5332/5879/637/2773/259230/8560/1901/
tags=45%, 834/6347/5880/925/3937/3932/2212/29108/4790/71/4792/10109/10096/3553/4773/38
tags=52%, 9410/10189/57819/3310/6628/22938/10450/3190/9416/9775/1665/6426/10084/4686/2
tags=48%, 3118/3113/3122/3108/3106/3115/1520/3134/3135/3119/3126/1536/3689/3133/3109/3
tags=38%, 6772/7533/3627/10379/4790/4792/7534/4609/23586/7529/4939/4938/355/6774/4599/
tags=46%, 6772/7128/915/10379/7097/3560/917/4790/4792/51284/2213/3553/10399/3310/23586
tags=45%, 6347/5880/4688/6401/5175/4790/6613/71/653361/3553/7056/387/3383/7412/1906/58
tags=40%, 834/1536/6772/6775/330/7128/10379/197259/29108/3459/3553/3460/8743/355/6774/
tags=43%, 6188/6175/6187/6223/6193/6122/23521/6130/6203/51187/6191/6229/6125/65005/620
tags=42%, 3937/2207/2212/4067/71/113/387/6916/4638/10672/7408/5908/5332/103910/10235/3
tags=50%, 6772/3937/3932/4689/7305/10379/4688/10859/2212/2274/4790/9103/2215/2209/4792
tags=41%, 6772/6775/3587/3575/3600/1439/10379/3560/1438/3601/3459/3574/5771/4609/3460/
tags=39%, 1520/330/1439/10376/3002/84790/4790/597/71/4792/5551/5366/1075/1519/8743/355
tags=36%, 3106/3135/962/3689/3133/3107/3105/5880/3937/919/3932/3384/3824/2207/22914/73
tags=56%, 6352/6347/3659/3627/330/7128/3600/197259/6401/4790/4792/7133/3553/2919/2920/
tags=44%, 3118/3113/3122/3108/3115/3119/3126/3109/3117/6772/958/3587/1234/330/7097/312
tags=32%, 3118/3113/3122/3108/3106/5788/3115/3134/3135/3119/914/3126/3689/3133/3109/31
tags=49%, 1536/3689/7852/5880/4689/3702/4688/3683/83593/5175/3684/71/653361/5579/399/3
tags=43%, 1536/6347/6772/6401/4790/1282/1284/5579/3553/7056/6774/3383/7412/1906/3576/5
tags=51%, 5788/5880/3055/2212/4067/2215/2209/2214/10109/10096/653361/5579/2213/1072/74
tags=64%, 3118/3113/3122/3108/3115/3119/3126/3109/3117/6772/919/3932/915/3123/3560/479
tags=53%, 5788/925/3937/919/3932/915/3702/4794/917/4790/4792/4773/387/7535/4690/10892/
tags=52%, 6352/6347/919/915/713/7097/917/4790/4792/3459/714/3553/3460/355/6348/6349/41
tags=50%, 958/10673/6363/3932/330/929/7128/6351/4067/4790/597/4792/6366/5579/3553/2358
tags=47%, 6352/6772/958/942/3627/929/6373/4283/7097/6351/4790/4792/51284/51311/3553/70
tags=45%, 834/6772/3659/2207/10379/29108/4790/4792/64581/3553/4773/6237/387/114548/841
tags=42%, 3689/911/913/929/7097/912/3684/4790/1282/1284/5579/3553/2919/2920/5272/3576/
tags=42%, 3118/3113/3122/3108/3115/3119/914/3126/3109/911/3117/925/913/929/915/3575/91
tags=40%, 729230/7852/6352/6347/3587/6363/3627/1234/1524/6373/4283/6375/6846/6351/3560
tags=41%, 330/4790/4792/1282/1284/4609/5728/7185/3688/3551/7187/3673/1021/2335/7188/12

tags=55%, 6772/919/3932/915/7097/4794/917/4790/4792/3459/55509/4773/3460/6774/5728/753
tags=52%, 3118/3113/3122/3108/3115/3119/3126/3689/3109/6352/3117/6347/10673/942/3600/7
tags=47%, 6347/3627/7128/4790/4792/3553/2919/6356/2920/6372/3576/841/6426/3551/7187/68
tags=44%, 3118/3113/3122/3108/3115/3119/3126/3109/3117/6772/6775/919/3932/915/3123/356
tags=39%, 5880/10859/4067/4794/4790/4792/8519/5579/27071/2213/4773/118788/29760/5879/1
tags=43%, 3689/713/3684/11326/2/714/716/1604/1191/715/7056/3078/3075/966/730/2149/3426/
tags=61%, 3118/3113/3122/3108/3106/3115/5720/1520/3134/3135/3119/3126/3133/3109/3107/3
tags=38%, 694/57819/10950/2023/9337/25904/80349/22803/55802/4848/26986/5394/54464/8418
tags=46%, 3059/71/10109/10096/387/10459/5879/10092/9844/3688/2335/10552/858/5747/857/8
tags=48%, 834/3394/3689/3659/929/713/29108/3684/4790/714/716/1072/3553/715/387/6372/114
tags=53%, 5901/1736/55813/22803/29107/2091/10813/28987/55272/51119/10557/23560/54464/8
tags=45%, 3118/3113/3122/3108/3115/3119/3126/3689/3109/3117/713/3683/2212/3123/3112/36
tags=66%, 3118/3113/3122/3108/3115/3119/3126/1536/3689/3109/3117/6772/4689/7097/4688/2
tags=50%, 3118/3113/3122/3108/3106/3115/3134/3135/3119/3126/3689/3133/3109/3107/3117/3
tags=36%, 834/6352/3627/6351/29108/4790/4792/3553/23586/9447/3606/3551/10623/8737/5434
tags=62%, 3118/3113/3122/3108/3115/3119/3126/3109/3117/6772/6775/7097/3123/3112/4790/3
tags=51%, 834/3689/929/7097/29108/3684/4790/4792/3553/3310/2919/2920/3576/841/3606/709
tags=49%, 3689/6347/958/3820/22914/7097/3683/2995/6401/5175/3553/3383/7412/6403/3576/2
tags=50%, 3118/3113/3122/3108/3106/3115/3134/3135/3119/3126/3133/3109/3107/3117/3105/9
tags=56%, 5696/5720/5698/5699/5721/5687/5702/5689/3458/5685/5691/5686/5719/5682/5684/5
tags=73%, 3118/3113/3122/3108/3115/3119/3126/3109/3117/958/942/713/2212/3123/3112/6737
tags=54%, 3118/3113/3122/3108/3106/3115/3134/3135/3119/3126/3133/3109/3107/3117/3105/9
tags=59%, 3118/3113/3122/3108/3106/3115/3134/3135/3119/3126/3133/3109/3107/3117/3105/9
tags=62%, 3118/3113/3122/3108/3115/3119/3126/3109/7852/3117/958/10673/942/3600/3123/31
tags=50%, 5788/958/925/3932/915/3575/6890/4261/64421/7535/100/29760/5993/3543/8625/689
tags=64%, 4176/5982/5111/6118/4172/5557/6117/10535/5984/2237/4171/54107/4175/4174/5665
tags=45%, 3620/6401/8542/5579/3553/355/3383/7412/5332/3606/3458/4615/2150/2776/7124
tags=67%, 3118/3113/3122/3108/3106/3115/3134/3135/3119/3126/3133/3109/3107/3117/3105/9
tags=65%, 3118/3113/3122/3108/3115/3119/3126/3109/3117/958/2207/3123/3112/2205/6356
tags=60%, 23478/90701/3309/58477/5018/9789/6731/60559/11231/6729/28972/6726
tags=28%, 7533/3575/10161/7097/3560/4790/1282/3574/7534/1284/54331/4609/7529/5728/1097
tags=81%, 224/138199/4129/26/3176/4128/57571/221/84735/219/10841/222/223/144193/55748/
tags=41%, 10010/6237/5728/81671/3091/64422/22863/3146/6885/9451/5594/5567/29982/51100/
tags=70%, 805/808/5158/3000/2978/9626/1258/5148/2779/810/8787/6011/2979/6010
tags=46%, 51733/8310/221/84735/54498/219/339896/222/223/35/55748/2571/218
tags=60%, 38/5322/10999/5319/8694/4547/2168/5406/949/2169/8513/80168/84649/337/10554/1
tags=57%, 255022/8645/775/3354/2554/5137/6335/1131/9033/5566/5330/2911/5331/80835/3355
tags=48%, 805/808/283297/79541/79339/138804/26248/4992/26529/3000/26689/5137/158131/26
tags=63%, 10975/845/4624/4625/782/4633/6547/59283/7169/7134/23439/1339/59285/785/1337/
tags=31%, 3689/7852/5880/3683/3684/71/3071/10109/7114/10096/1072/6237/387/4638/10672/5
tags=36%, 3059/7097/71/5579/4609/6237/387/960/355/6774/5879/8826/4060/406991/3688/3091
tags=50%, 4704/1339/1337/479/9550/4725/84701/9377/4715/1346/374291/29796/4708/56901/47
tags=37%, 3689/5880/3937/3683/83593/3684/71/5579/113/6237/387/5216/1436/7408/3397/5908
tags=44%, 4725/2775/22999/779/2793/775/2892/778/774/5578/4715/374291/57406/2554/3763/4
tags=50%, 5366/355/5728/841/637/64065/472/1021/4616/6241/11200/1643/891/64393/8493/101
tags=39%, 126129/1352/53632/4704/1339/1337/84335/6009/2475/4725/84701/123096/29078/493
tags=36%, 5880/330/64098/71/1282/1284/5579/387/4638/5728/7408/5908/5879/87/3676/3371/10
tags=27%, 2767/5566/1950/2324/5330/816/2911/80271/85366/491/818/5331/3362/3706/340156/
tags=52%, 330/9246/7318/8881/7316/140739/92912/4214/10055/51366/868/7323/9616/8453/732
tags=55%, 113278/5406/949/80704/5948/337/151056/6573/335/25974/113235
tags=64%, 2560/2893/2561/773/2892/774/2554/2570/8973/116443/2563/57030/2905/2902/1141/
tags=40%, 4544/554/2918/4543/2570/2688/2689/6343/4295/8973/1131/11255/90226/623/7068/1
tags=38%, 4790/4792/4609/1488/3551/7048/5594/1021/3065/4089/5925/4616/1027/8503/1643/1
tags=36%, 3627/4790/4792/10010/23586/54941/3576/841/1540/64135/3551/9636/7187/6885/421
tags=41%, 241/5880/3937/2207/4067/2205/5879/5321/7409/27040/5594/8503/3635/4893/7124/2
tags=32%, 1536/6352/5880/4689/713/4688/10376/84790/714/653361/5687/3553/3310/5879/730/
tags=36%, 4790/1545/9839/5579/4609/7431/387/960/6774/5728/4082/3371/3190/4170/406991/3
tags=40%, 5880/5366/4609/387/5879/7048/5594/2353/4089/4616/8503/4436/1643/1499/572/514

tags=32%, 6844/6532/1759/6511/127124/245973/535/1212/57030/6531/10814/155066/527/5948[illegible]
tags=50%, 5579/5687/5332/5702/5689/22863/2903/9519/29982/3516/7419/5685/5691/8503/295[illegible]
tags=27%, 6772/5880/4790/6774/5879/9459/3551/7048/5594/3716/1021/4089/5925/4616/8503/1[illegible]
tags=43%, 7357/259230/8560/2720/253782/9517/8612/7368/55304/130367/8611/8877/91012/81[illegible]
tags=41%, 10189/9775/4686/29107/2521/53918/64895/5411/26986/23283/5527/132430/11052/1[illegible]
tags=39%, 5880/71/387/5879/87/8826/7048/6885/5594/4089/1495/10163/1499/283106/1457/745[illegible]
tags=58%, 4825/5080/3171/5313/5078/3651/2645/3170/3630/3172/6927

3459/1282/3574/5366/1284/5579/54331/113/4609/3460/387/6789/355/6774/5734/5728/10672/14
347/3105/6772/972/330/6890/567/10379/7097/3123/3112/4790/10189/4792/3459/10308/3553/23
728/841/3676/4600/3371/3688/3551/9636/7187/7098/8323/3673/5594/3716/472/5567/1021/951
02/4928/637/2882/79023/5689/23165/310/4686/84617/55860/22863/9451/220988/29107/2903/3
1/10109/10096/399/4609/3553/6237/387/8743/114548/8767/5216/23643/3576/841/5879/3606/10
378/11031/10092/29082/84313/8724/7048/163/26286/8766/93343/50807/3799/51100/868/10552
/1439/6375/6846/6351/3560/1438/3603/3601/7133/3459/3574/6366/3553/2919/3460/6356/2920/
90/4792/5579/54331/113/4609/3553/4773/387/355/6774/5734/10672/3588/6348/6349/414062/35
84/5579/716/51311/6223/3553/715/23586/4939/4938/6193/6774/114548/4599/6122/23521/6403/
105/958/3932/915/3600/567/3683/3123/4316/3560/3112/917/4790/3601/4792/113/4609/4773/33
48/8767/5216/3576/5332/5879/87/3554/3606/10892/10092/103910/9844/3688/831/3551/7316/22
772/958/919/3627/7128/915/6890/567/10379/7097/3683/3123/4067/4794/3112/917/4790/4792/4
097/25939/917/4790/4792/7133/5579/54331/1072/4773/355/841/5879/637/2773/3551/6891/6885
3553/387/8743/10972/355/114548/10672/9076/3576/4690/841/5879/3554/64005/3606/10092/368
/6375/6846/6351/4067/4790/4792/653361/6366/5579/54331/113/2268/2919/387/6356/2920/637
29/2207/7097/2212/3123/3112/3684/4790/4360/9103/2215/2209/2214/3459/64581/4261/2213/35
4331/4609/4773/2919/2920/355/6774/3383/3576/841/9976/5879/637/3551/7316/7187/7098/3091
7323/3309/55768/6748/3326/22872/27102/10483/23471/7321/5610/6500/10134/11231/6184/642
3576/841/353376/637/64135/3551/7187/7098/7048/1959/6885/5594/3716/4214/2353/7099/4089/
472/1021/5925/4616/7419/11200/8503/891/1875/3710/2309/23411/4893/1019/824/2113/890/592
/5090/3576/7185/8842/4086/3087/7048/7403/2521/472/3065/4616/5747/4211/1027/1025/8464/2
9760/4686/1968/29107/2521/9669/5411/9972/26986/387082/8487/10557/53371/57122/8892/231
/103910/3688/5567/4214/5564/4627/10552/7846/7430/51735/1365/10627/10095/3725/1739/421
4/4790/4792/115361/10010/3553/2919/387/2920/64127/4939/4938/114548/8767/10628/10135/3
123/29108/3112/4790/71/4792/3459/51284/5579/4261/3553/23586/3460/8743/3838/4939/4938/3
529/6774/10971/22938/841/5879/87/7185/3190/6672/7187/5902/9734/1959/5594/3716/5567/102
1/472/1021/3065/4089/5925/4616/1027/11200/891/1875/994/6500/4171/996/64682/1019/4085/4
5594/1903/8503/253782/9517/4363/2776/5527/4893/7124/55304/2206/130367/2770/8877/91012
7/114548/7535/3576/5879/3676/3606/10092/9844/3688/3551/7409/8935/27040/6885/5594/2335/
20988/494115/23350/2521/6427/6430/3183/51645/55660/10772/55119/3192/6434/23451/84844/
107/3117/11151/3105/4689/929/6890/7097/4688/10376/2212/3123/84790/3112/3684/4360/9103/
3646/10971/9076/841/4600/637/6041/3551/7187/7098/9451/5594/3716/1021/5925/3458/975/873
/4939/4938/355/6774/4599/841/4600/637/64135/3551/7187/6885/9451/3716/1021/2353/7099/93
79/3554/3551/6885/2353/7184/858/5747/3458/387082/2950/3326/857/8503/1499/7124/652/3725
114548/841/353376/1540/637/29082/5321/7098/3146/3716/2752/7099/7188/6778/128866/3717/3
2/6201/6124/6142/6194/6173/6146/6136/124995/6143/29088/6135/6189/11224/6228/6132/2009
688/2773/5321/2149/3673/5594/5567/64805/83706/8673/8503/10627/2776/3710/10125/2770/86
/2214/3459/653361/55423/2213/3553/4773/3460/1436/29760/5879/1540/3554/4982/3551/7048/
6774/3588/4170/3563/3716/6778/3717/3458/3594/3597/8503/3976/122809/6773/5156/3718/946
/841/7185/4170/637/3551/3563/9451/5594/472/2353/7846/4616/143/8737/8503/8837/3710/572/
05/3683/3002/2215/2214/3459/3821/3822/10870/5551/5579/4773/3460/8743/355/3383/7535/587
6372/64127/355/3383/7412/1906/841/7185/8809/3551/7187/7424/6885/5594/1326/6364/2353/71
3/3112/4790/4792/3459/4261/3310/3460/6774/23643/3588/841/3688/2773/3551/6885/5594/371
07/3117/3105/958/942/925/3384/6402/3683/3123/3112/6401/5175/3684/3383/7412/6403/9076/3
87/3383/7412/7408/5908/9076/5879/87/3676/103910/3688/2773/7409/4267/5747/7430/1365/85
332/5879/7424/7048/5594/2335/4089/1285/3717/1027/8503/4893/7124/3725/1286/1019/1281/1
08/4082/5879/10092/5321/7409/27040/5594/65108/50807/10552/8503/1398/3635/8612/10163/1
4/3112/917/4790/4792/3459/3553/196/4773/3460/6774/7535/3554/3551/3091/7048/27040/5594/
3551/7409/27040/6885/5594/1326/2353/868/3458/916/8503/4893/10125/29851/7124/3725/1739/
4062/3576/5332/841/2773/3551/7048/5594/2353/7099/3458/9630/916/4615/8503/8837/2776/511
6/2919/2920/3383/7535/7412/23643/29760/3576/353376/1540/3554/7185/10892/3551/7187/405
96/23643/6348/6349/414062/3576/841/353376/5879/3551/7187/7098/6885/5594/1326/2353/709
/1540/10892/26253/3551/1959/5594/868/5971/8503/3710/6773/4893/7124/3725/6850/1960/529
5332/87/3554/5567/2335/7099/1285/5747/3458/9630/8503/2776/7124/3912/3915/1286/3918/128
2/3123/1438/3112/917/3684/2209/3574/1604/3553/960/945/931/1436/966/3676/3554/952/3563/
/7133/6366/2919/6356/2920/6372/8743/8807/1436/6355/3588/6348/6349/414062/3576/3606/88
85/5925/4616/5747/1027/8503/1643/51426/3912/3915/1286/3685/3918/1019/5290/317/836/116

5/353376/29126/3551/10538/3091/27040/5594/3716/2353/7099/3717/3458/916/4615/8503/4893/
097/3683/3123/3112/3553/2919/2920/6372/3383/6348/6349/414062/3576/3606/4050/6364/2353/
85/5594/6364/2353/7184/7188/3458/3326/6280/3934/7124/3725/29110/3320/836/6374/5743/560
0/4794/3112/917/4790/4792/3459/864/4773/3460/7535/3551/27040/5594/3716/2353/6778/3717/
.0892/3551/7409/5594/2353/10288/975/8503/11025/3635/971/4893/3725/6850/10451/5777/5290
/5329/710/3080/5265/629/729/717/718/2162/10877/2244/7450/4179/5054/2266/5327
117/3105/972/925/3824/5721/6890/567/3123/3112/10437/3821/3822/4261/3310/5993/8625/6891
36/23404/25804/132430/5214/9652/7832/219988/246175/51013/29883/9125/22894
503/1495/1398/10163/1499/10095/391/5290/10097/10093/7414/63916/60/3611/867/10094
4548/23643/3576/353376/3688/2773/5594/2353/7099/10392/710/4615/717/51135/718/7124/3725
34128/55127/26354/283106/1457/51096/9724/134430/10482/29889/10199/79631/10528/10775/7
84/9103/2215/2209/2214/714/2213/716/715/2359/3383/2357/6403/3075/3880/3875/6404/3426
212/3123/3112/3684/4790/9103/2215/2209/4792/2214/3459/653361/5579/3553/3460/3676/3688/
105/5880/958/942/3683/3123/3112/71/5551/1604/3383/841/5879/637
l/3665/11035/29110/103/10621
459/3553/3460/64127/6774/8807/3606/8809/7099/6778/3458/3594/30009/7124/3725/3111/3125/
9/4615/718/4671/7124/9554/1937/1195/7100/317/836/3593/5861/3312
532/3606/7099/3458/975/4615/7058/7124
58/942/3123/3002/3112/5551/355
704/5700/5706/5714/5683/5718/5688/5710/5701
/2215/2209/2214/714/716/715/6628/87/730/2903/3458/6741/729/717/718/7124/3111/3125/3127
42/3123/3002/3112/5551/3553/355
42/3824/3123/3002/3112/3821/5551/3553/355
12/3601/3676/5284/608/56477/29851/3111/6387/3125/3127
1/916
55/4173/5985/79621/246243/5981/5427/57804/5422

58/942/3123/3002/3112/5551/355

71/1436/118788/5879/3676/3371/4170/3688/3551/2149/3563/11140/7424/4254/3673/5594/3716/
443/218
/8673/203228/10670/8503/8837/55014/572/11345/7405/4893/10325/4218/89849/29110/8678/105

208/376497/50487/335/1056/5407
5/3351/9568/6338/5153/114/170572/6337/954/3352/2784/2914/22953
5245/1258/5566/816/4993/123041/8392/818/26539/26740/26211/26476/2788/156/26664/26716/1
84701/4635/779/4634/9377/775/778/1346/29796/6548/9254/786/27091/125965/7381/783/7137/2
216/5879/87/3676/8826/10092/103910/3688/9459/2149/7409/3673/5594/2335/4627/10552/3696/
/8323/7409/27250/967/3673/5594/5329/5567/2335/7099/7474/858/5747/7430/857/6194/8503/16
731/4713/127124/245973/535/4706/4714/125965/4695/7381/4702/4707/4723/4718/155066/51079
/2357/5332/9771/5879/10235/3688/2773/2149/7424/7409/4254/51466/27040/5594/2903/51735/8
708/2570/56901/4731/4713/4706/4714/5566/4695/2563/5330/2911/4702/57030/4707/4723/4718/
9/901/92344/8795/3486/317/836/983/2810/545/896/5054/6477/25898/7057/894/9134/27244/419
3753/9377/5606/6196/4715/148327/1346/374291/29796/6598/4708/56901/4731/4713/4706/4714/
03910/3688/7424/7409/3673/5594/2335/1285/3696/858/5747/3694/857/8503/10627/1398/1499/6
2064/8074/4914/5731/3360/8822/2905/5260/553/93129/107/6915/56413/4843/219931/810/155/1
28/1643/4281/7334/7321/6500/55236/9320/996/64682/55958/51465/22954/90293/29945/27338/2

1137
87/83551/116443/7349/2488/4157/1815/5644/2563/2911/8698/23566/1135/9294/10316/1443/11
398/572/51426/4893/1019/5290/1487/9846/7040/867/7046/10000/5781/5293
.4/8737/3665/7124/1654/29110/9140/3593/9474
206/8605/6850/10451/5290/9846/5603/2534/6416/10000/5293
5702/5689/84617/9451/5594/2903/3831/5567/3799/7846/3309/858/7419/5685/5691/857/8503/72
551/27250/5594/472/1021/3065/2146/4853/7430/1786/1027/8503/1398/4363/994/23411/4893/27
26/4893/5899/3725/4292/5290/836/4087/7040/2956/7046/10000/5293/2932/374/6934/6198/260

55/6534/6512/6538/525/6530/160/23025/5864
9/5686/5719/5682/2776/3710/489/5684/5704/5700/8678/4217/5290/5706/5714/3708/5289/5683/1
643/572/51426/5899/1019
537/8879/2717/55331/8613/5660
1051/65110/23708/5500/26019/5499/55308/1478/10482/1477/11269/10914/2733/81608/65109/23
4/6591/5777/52/7414/7082/60/7525/10810/2534/7046/2033

436/1910/1906/3576/5332/841/5879/1488/7185/5732/637/10235/3688/2773/3551/7187/2149/3563
3586/162966/3460/4939/4938/355/79230/841/80264/5199/637/64135/148156/84527/57693/6041/
9/2335/3065/1285/3696/5925/7474/4853/5747/3694/3516/1027/8503/1499/1452/6696/7058/572/5
831/2521/29982/3799/7846/3309/9972/53371/5685/5691/57122/203228/10121/5686/9688/5719/8
0092/4074/103910/9648/9844/3551/84617/55860/6885/5594/3831/8677/55770/51626/2353/3799/
/9815/128866/858/9744/60682/64744/10015/857/51652/10565/6643/2350/116984/10095/57132/4
/6372/8743/355/8807/1436/6355/3588/6348/6349/414062/3576/3554/3606/8809/1236/939/94/498
576/5332/841/5879/3554/5732/637/2773/3551/6891/5594/3716/5567/1021/7188/5925/5747/2791/
/3576/6130/730/4600/6203/8829/64135/51187/6191/3551/9636/7187/7098/6885/5594/6229/3716/
83/5728/9184/5901/2114/9232/3554/8829/3551/8881/7048/5902/1959/5594/3716/472/5567/4214
2863/6885/5594/472/7099/10552/7188/7323/23048/10392/5747/4615/8737/10670/8503/10627/13
609/7431/864/23586/4939/960/4938/355/6774/3383/29760/22938/841/5879/5702/637/3551/9636
5/5594/472/1174/2353/7099/7188/8905/5747/2791/916/9616/4615/8737/85363/8503/1398/891/27
38/3551/2149/84617/6885/5594/2353/7099/4627/10552/7846/7430/10006/4615/8737/1365/23191
2/6774/6355/5908/6348/6349/414062/3576/5332/5879/1236/10235/9844/2773/3551/10563/7409/
553/7096/3460/387/64127/8767/3588/841/5993/3606/10892/637/26253/8625/5594/3716/4801/709
1/64422/22863/5594/3716/1021/2353/5925/3717/4277/2791/8503/3665/1499/3710/6773/718/5610
215/7993/30001/51465/824/3320/6745/2923/4217/10427/1965/821/6400/55968/3312/573/468/987
/6778/5925/3717/7419/4615/8503/3665/1643/572/6773/51135/64764/10488/4893/7124/1654/372
28/5500/5934/5499/3486/5290/983/10111/4683/545/4087/3708/5533/5603/896/5054/5534/7040/7
130/1643/4300/4297/25942/2530/51426/1655/905/2078/5966/2005/890/1848/3207/3486/6760/96
91/9688/8480/1977/96764/9984/8667/132430/55706/11260/6606/6607/65110/9631/1975/7341/57
3/50848/1019/7454/1741/5584/4217/9475/1364/51762/10097/10686/10093/5562/7082/4628/60
576/5332/841/9447/3606/6041/84674/3551/7187/9051/6885/5594/3716/22900/7099/7188/10392/
355/114548/3383/4599/3576/841/4600/3606/4928/637/64135/6041/3551/7187/7098/5594/29107/3
21/9519/3065/7188/5925/2961/3516/1027/7419/8503/2965/2959/3665/572/718/5610/64764/10488
4175/890/4174/5885/5934/5591/29945/983/2810/545/4087/4173/6502/7272/896/8454/7040/10274
/4217/8439/9475/5290/81537/8879/9846/5603
/2353/7099/10552/9815/5747/4615/8503/5586/1398/10163/2776/51135/10095/7124/3725/7454/2
/27316/55696/9984/10286/6432/1655/25804/84950/9716/57461/9343/1659/5093/29896/8683/849
/2215/2209/71/2214/64581/653361/2213/715/5879/4074/3688/6891/84617/3673/7099/7846/1781/
37/1365/8503/27102/3665/8837/1499/91543/572/6773/5610/4893/7124/1019/29110/3454/1965/52
367/868/916/1027/4615/8503/6504/27102/3665/572/6773/51135/5610/8667/3725/283106/1457/10
5/3685/3320/3690/7341/4217/5290/5562/4205/4257/60/1003/5603/4208/4318/5327/1843/6416/51
3458/7419/8737/3326/51652/8837/6773/5836/5610/11035/7124/57132/8605/824/3718/3320/3454
16/51081/6138/6159/6217/64963/29074/6224/65008/11222/9045/65003/6134/51263/55173/28998
05/54518/6850/3690/1281/2244/4659/5742/5500/5584/9475/5499/5290/7450/3708/60/5603
6885/5594/3716/2353/5971/3458/10288/8503/11025/6773/7124/3725/1385/6850/3690/3454/5290
5/3454/9180/5777/8835/5290/3593/3455/8027/3572/8554/3570/896/2273/2033/85480/3977/9021/
4893/7124/3725/824/835/1521/4217/8795/1965/5290/317/836/3708/60/468/4001
79/637/4068/7409/27040/5594/3823/4277/3458/117157/8503
188/8737/8503/8837/3976/64764/10488/11035/7124/3725/9586/1385/4217/5290/836/6374/6376/5
5/7099/3717/3458/4615/572/51135/7124/3912/3915/3918/3111/2770/3125/3127
676/29126/1462/9019/3688/6404/201633/965/79679/3696/4267
03/1495/10627/1499/7294/50848/2770/6387/10451/9475/5290/1364/10686/7414/60/1003/5603/43
35/5290/836/4087/5603/5054/7040/7046/1958/10000/5293
10095/8605/7454/8611/6850/8877/10451/5290/10097/10093/9846/382/10810/8613/10094/85477/1
/3716/2353/4089/6778/3717/3458/916/3594/3326/3662/30009/3725/3111/3718/3320/3125/3127/9
/1019/10451/1493/5777/5290/5533/5603/5534/2534/5588/5062/4775/10000/940/5293/2932/959/9
35/718/7124/3725/2770/5290/3593/4087/5603/5054/7040/7046/2771/6416/10000/356/5293
0/27040/6885/472/7099/7188/4616/5971/4615/8737/8837/51135/6357/7124/283106/1457/6850/6
9/4615/8737/8503/3665/6696/51135/7124/3725/29110/7100/3454/5290/3593/3455
0/3593/5743/3708/5533/5603/5534/4775/3709/10000/5781/5293/22808
1/5290/836/3593/5868/7414/3914/3908
100133941/4254/3673
09/1236/10563/6364
3/5743/6502/3914/3908

/10125/3725/283106/1457/27436/5777/5290/5533/5603/5534/5588/4775/10000/5781/940/5293
/7099/3458/10312/7124/3725/3111/6387/284/3125/3127/1493/528/51382/6374/7010
03/4318/6279/51433/23765/4312/10758/2932
/4853/3458/916/3516/3594

1/3823/4801/3309/3458/3326/4802/6892/7124/3111/1385/3320/3125/3127/2923

5/2770
514/10799/55131/51068/166378/4931/83732/55781/10556/3692/23195/1460

/6885/5594/3716/65108/2353/7099/3717/3458/4615/51135/718/7124/3725/3111/3125/3127/5777

/3127/7100

1021/2335/7099/7184/1285/3696/3717/5747/3694/2791/4602/1027/3326/6194/8503/5586/6696/7

33/1965/9140/5290/5861/9474/84938/5562/55255/3708/5289/5588/9706/10000/5293/8897/2280

126370/810/5153/815/26658/7932
27092/7139/1349/1340/488/1347/482/1351/1350/1329/27089
/5747/7430/3694/8503/10627/23191/1398/10163/10095/4893/2247/3685/5156/5305/6387/3690/1
34/1499/3710/1655/4893/7124/2247/3685/2535/3690/10855/1975/10451/4659/5500/5777/7078/9
0/527/4726/496/1349/495/1340/525/64077/1347/4696/4709/1351/4694/4697/4701/1353/1350/132
3503/1398/135/1499/2776/4893/5899/2247/1268/2770/5156/8631/54518/3690/284/10451/57568/5
/5331/3762/2788/51079/4726/6300/5595/107/114/2166/4696/5600/747/115/2915/4709/2784/4694
4/8797/51246
/5566/125965/4695/7381/51548/4702/4707/4723/4718/51241/6602/51079/4726/55811/6300/1349
696/7058/572/3912/3725/1293/3915/1286/3685/3918/5156/824/3690/10451/4659/5500/9475/549
153/488/5153/114/2902/2925/53373/3274/1956/26291/91807/56848/2251/815/115/8912/2915/891
6091/11065/9039/8554/6502/8454/55120/6477/867/11059/25898/9021/4734/51433/83737/55294/

36/5032/134860/3362/3640/5645/5697/9038/2912/7253/3355/6755/5731/51052/3360/1442/2905/

29/5686/5719/5682/3710/572/64764/203068/10488/7124/283106/3915/1457/9586/5684/1385/570
/44/5156/3690/7078/3236/5290/836/7168/5743/4318/5243/1788/2033/6768/5154/6935/9252/705
60/10018

10105/5718/7436/5688/10528/9706/5710/5701/115209/3709/10000/5293/7494/293/1600/9861/23

3049/23435/9939/29101/80336/10767/55110/2935

3/3091/7424/8323/4254/7048/3673/5594/3716/5567/1021/2335/2353/3065/4089/7184/7188/1285
6426/6672/3551/1967/7187/7098/6891/6885/9451/3716/7752/29992/6427/84671/7748/51427/643
5527/8638/6773/5610/64764/10488/10134/4893/10312/7124/3912/1739/1293/3915/1286/9586/36
3480/5682/3710/572/493869/6432/203068/11345/23064/7124/55706/4218/5684/5704/29110/9631
7099/9367/7184/10552/7846/6281/10392/1781/10006/4615/8737/3326/10627/10121/23191/388/1
4218/1175/6642/11311/5156/5584/23527/23362/10564/10097/8027/10093/5868/27243/25978/802
32/3563/10563/7048/4050/6364/608/9235
/8737/8503/1398/1499/2776/6892/3710/64764/10488/4893/7124/9586/3685/2770/1019/11214/13
/6125/2353/6202/7099/6201/6124/6142/6868/4615/6194/8503/6173/629/729/6146/6136/717/614
4/9519/4801/2353/4089/5925/5971/916/7419/11200/8503/64764/10488/4893/7124/3725/1739/20
98/10163/3710/7334/7321/2309/718/11345/6500/10095/4671/7124/3725/10325/7336/824/29110/
5/7187/953/965/6891/6885/3716/1021/3065/7188/5925/4616/5971/916/3516/1027/4615/8737/881
776/6892/3710/572/51135/6500/4893/7124/3725
./10163/4642/51135/203068/10095/4671/4644/7124/3725/4430/7100/5777/8795/10427/9475/836/
5594/5567/6364/3717/5747/2791/8503/1398/2776/572/6773/2309/6357/4893/56477/58191/2770/
99/3717/3458/4615/4802/572/51135/718/10312/7124/3111/1385/6850/8877/3125/3127/1054
0/11345/4893/3725/2247/1019/1385/29110/6850/8678/3454/131450/5290/836/3455/3572/5743/3
71/8454/80267/9601/201595/267/1603/8720/9532/5887/51128/3301/10277/7494/22926/9709/478
5/9586/1385/3718/29110/890/3454/1960/5290/317/836
7046/4775/3709/10000/894/5293/3805/10758/22808
511/4208/4318/5327/466/9915/4149/4291/51274/5154/6935/894/648/4286/4005/5468/5079
7510/8672/1965/26019/55746/8665/7175/55308/10482/51808/11218/11269/51095/54960/55520/1

/55669/7419/4615/8737/3326/5586/3665/51393/3710/6773/51135/11345/4671/11035/7124/3725/
3716/8766/1021/7099/3717/3458/4615/8503/3665/8480/91543/6773/51135/5610/7124/3841/3111
3/4893/1654/3725/1739/5966/9586/1019/1385/3718/890/6850/5700/5934/5922/1960/5290/836/2
4/2033/51433/894/23595/2932/5001

9110/391/10451/9475/5290/10097/10093/63916/60/5603/382
91/11325/3312/144983/58517/26121/6632/6431/151903/988/11017/5356/10915/51340/51691/51
/7058/718/203068/10312/9554/3685/3111/3690/3125/3127/528/821/51382/79659/1783/5868/511
290/317/836/1364/3455/10686
019/3718/29110/103/3454/1965/5290/317/836/3593/3455/4179
.54/10000/5293/6383/6385/2941/801/4780/90/25828
-/8795
3/6167

0/3455/814/9846/5533/5603/5534/7040/2534/7046/9021/3726/10000/5293
/5154/10000/5781/894/5293/9655/5159/3566

5743/468/5603/4318/6416/9021/840/3726/10000/9252/83737/5293

318/11069

10000/5293
0466/3572/4087/3570/5533/5603/5534/7040/5588/7046/4775
920

387

058/1977/572/5527/2309/64764/10488/4893/3912/2247/1293/3915/1286/9586/3685/3918/1019/1

3/64798/6198/3476/440275/140775/1612/55102/83452

0451/4659/5500/9475/5499/8516/5290/10097/1902/10093/7414/1730/2252/4628/60
9475/3236/5499/5290/836/3593/4087/3708/60/5603/4318/7040/867/8321/3709/3082/10000/5781/
29/27089
5584/889/5290/1902/7010/2252/60/5603/25780/11069/56034/23683/2771/3082/5154/10000/7057
4/4697/4701/5582

/107/1340/6199/8110/155/1347/353500/91942/3991/114/57104/4696/10818/5600/26291/90993/4
99/8516/5290/7450/7414/60/3914/3908/3611/896/2534/56034/5062/3082/5154/10000/7057/5574
13/3363/5582/3361/22953/147
/8452/10277/8450/55284/64750/7332/7319/1161/10477/57448/4193/10401/9040/10054/7322/892

553/1145/5646/3351/9568/1325/6915/165829/56413/1146/6753/57053/155/153/4987/2901/11757

4/5700/1965/5290/317/836/10963/5706/3312/5714/3708/5533/5683/468/5621/5603/7385/10105/
7/894/1945/5293/648/27086/5159/10253/6624

369/55102/5717/9698/60673/5692/7416/5707/6310/6667/25814/5649/9821

/6778/5925/4616/8202/3717/7474/4853/5747/3458/2791/8453/1027/3594/2950/3326/3597/8503/
30/7188/3717/146198/7770/7594/3458/7673/4615/94039/8503/8892/27102/3665/10793/6892/763
85/3918/5663/1019/1385/2535/29110/890/3690/5700/1741/3454/5934/5584/8516/528/51382/529
./5700/8678/4217/10718/51164/1965/55746/317/836/7175/5861/5868/5706/3181/25978/7802/104
1499/51135/203068/6500/10095/4671/11035/7124/5286/3725/3320/391/7100/10640/8795/51164/
223/3312/4087/382/57403/55737/5867/30844/9267/867/7046/11059/51510/10094

85/5156/29110/6387/3690/2923/9475/5290/836/6376/5743/3708/3570/5533/468/5603
3/6773/6135/51135/718/5610/2162/7124/3725/6189/11224/6228/6132/29110/6850/103/2244/200
05/9586/996/64682/3111/1019/4085/1385/2113/3718/890/3125/3127
7100/8678/9475/9140/5290/9474/11335/10097/10093/7414/63916/3708/5289/60/5603/3611/382
19/8503/3665/1643/5719/6892/6773/25942/51135/5610/51426/7124/3725/3111/1019/5704/3718/2

/1364/5861/10097/10686/1902/10093/10061/7082/2597/4628/60/5603/382/10810/9871/2534/926
7454/3718/6387/10451/6368/10663/9475

708/5533/5289/5603
0

10775/7514/2733/10799/65109/8669/23435/51068/8661/3837/10605/57187/9939/10556/80336/12

2635
./1019/29110/3125/3127/103/3454/8795/1965/7177/5290/317/836/3593/64499/3455
960/983

362/9879/3178/51729/57187/9939/6428/55110
.43/9341/5289/60

385/5156/3718/6850/3320/3690/284/1975/3454/5934/9180/8516/5290/8115/7450/3455/1902/556

356/7057/5293/6383

/1945/5293/196883/22808/5159

878/115/4709/84987/1351/4694/4697/4701/1353/1350/1329/57521/27089
2/894/5293

5/7337/55585/65264/26272/991/9354

9/3972/7434/2740/151/2902/4985/2925/2692/1813/1394/64106/5020/3274/2696/7442/8862/139

5534/5718/2534/4711/5688/5710/5701/1958

1495/4436/1643/1398/1499/2776/572/6773/51426/23401/6500/4893/10125/652/5899/3912/3725/
9/7559/572/55422/91661/6773/57209/51135/718/5610/6432/63934/55769/171392/7124/54925/8[illegible]
[illegible]0/836/26508/7450/3455
[illegible]82/5714/55255/144983/127602/5533/5289/60/5683/468/5603/7385/9896/5534/5718/4711/5688/[illegible]
9475/5290/79659/836/10097/1783/10093/5868/63916/51143/2597/5289/60/5603/382/10810/604[illegible]

[illegible]916/3454/185/6138/6159/6217/5290/3593/7450/3455

29110/890/6850/3125/3127/5700/3454/2923

7/5062/10094

[illegible]9401/5903/55110/8666/8890/6612/10419/25929/1915/8891

[illegible]2/7010/2252/3570/468/3914/3908

[illegible]2/2831/2147/3352/1141/2915/2690/1144/3363/2914/3361/7067/22953/1137/147

/2247/3915/1286/3685/3918/2770/1019/650/5156/2113/2535/3718/890/3320/6387/3454/185/2743
0110/65251/30832/10224/84874/10520/10795/7581/6940/3111/7699/162963/7766/29110/6850/36

847/9706/2733/5710/5701
12/64746/9267/6416/10094/3840/840/51429/58484/5287/10000

36/9475/4292/5290/317/836/3593/7175/1163/26508/3455/1902/3572/4087/5743/2252/1487/3570/
590/7767/57541/3125/3127/7539/91975/3454/5500/7562/2923/127396/79788/90592/7582/7571/7

/4257/6502/3914/3908/25780/896/10928/8454/4318/2271/7040/2956/1909/867/8321/7046/2771/[illegible]
695/8683/1965/51385/7596/389114/5499/90075/84924/7694/5290/317/836/3593/284406/284323/

2033/10681/9915/840/4149/3082/5154/10000/9252/356/894/5293/4312/196883/4286/2932/5159/
/3455/80818/58500/163081/140612/10482/126068/148266/390980/641339/6431/7637/339559/90[illegible]

3566/5468/2778/2941/6934/10319/801/4780/6198/26060/10018/999/9134/862/3595
333/148103/7556/7743/7691/9021/57232/93134/57547/10000/5781/57711/356/115509/7771/284[illegible]

[illegible]307/5293/7644/126295/7773/121274/9310/684/7574/158431/10172/23118/7568/7733/7587/1632[illegible]

27/440275/6428/340252/26152/342926/84914/8890/56242/284390/147948/9641/55786/10013182

7/345462/90594/3678/169270/8891/93474/136051/7592/7567/163087/80095/199704/199692/114

7/147929/7730/65243/5970/162993/79891/10780/4940/59348/57343/219749/90321/81931/1616/[illegible]

22869/147657/727/64170/7561/3592
