# Supplementary material for: The Potential Diagnostic Value of Immune-Related Genes in Interstitial Fibrosis and Tubular Atrophy after Kidney Transplantation
Source: J Immunol Res. 2022 Jun 17;2022:7212852. doi: 10.1155/2022/7212852 (PMC9232312; doi:10.1155/2022/7212852)
Supplement: Supplementary Materials — Supplementary Figure 1: GSEA enrichment analysis of the IF/TA group. Supplementary Figure 2: correlation analysis between ANGPTL3 and differentially expressed immune infiltrating cells. Supplementary Figure 3: correlation analysis between APOH and differentially expressed immune infiltrating cells. Supplementary Figure 4: correlation analysis between EGF and differentially expressed immune infiltrating cells. Supplementary Figure 5: correlation analysis between FCGR2B and differentially expressed immune infiltrating cells. Supplementary Figure 6: correlation analysis between HLA-DQA2 and differentially expressed immune infiltrating cells. Supplementary Figure 7: correlation analysis between LTF and differentially expressed immune infiltrating cells. Supplementary Figure 8: IPA analysis shows the interaction network of diagnostic genes: EGF and LTF (8A), ANGPTL3 (8B), FCGR2B and APOH (8C), and HLA-DQA2 (8D). Merged the above four independent networks to comprehensively analyze the interaction of diagnostic genes (8E). Supplementary Table 1: immune-related genes. Supplementary Table 2: KEGG pathway in normal group. Supplementary Table 3: pathway of ANGPTL3 gene. Supplementary Table 4: pathway of APOH gene. Supplementary Table 5: pathway of EGF gene. Supplementary Table 6: ingenuity canonical pathways. Supplementary Table 7: category. [file 7212852.f1.zip › 7212852.f1/supplementary table11.pdf]

ID	Descriptior	setSize	enrichmen	NES	pvalue	p.adjust	qvalues	rank
hsa05200	Pathways i	492	0.298313	1.646683	0.001319	0.007961	0.004061	3454
hsa05168	Herpes sir	427	0.415988	2.264992	0.001335	0.007961	0.004061	5725
hsa05165	Human pa	316	0.305065	1.614986	0.001404	0.007961	0.004061	3199
hsa04060	Cytokine-c	249	0.388902	2.017534	0.001416	0.007961	0.004061	3393
hsa05132	Salmonella	244	0.399936	2.068757	0.001418	0.007961	0.004061	3645
hsa04510	Focal adhe	196	0.321728	1.612719	0.001453	0.007961	0.004061	2746
hsa05163	Human cyt	210	0.393352	1.986707	0.001456	0.007961	0.004061	4102
hsa05166	Human T-(	211	0.492047	2.485744	0.001456	0.007961	0.004061	2852
hsa05131	Shigellosis	209	0.359561	1.817386	0.001458	0.007961	0.004061	3891
hsa05171	Coronaviru	195	0.510455	2.555869	0.001458	0.007961	0.004061	3247
hsa04015	Rap1 signa	194	0.341593	1.708855	0.00146	0.007961	0.004061	2756
hsa05169	Epstein-Ba	194	0.527837	2.640566	0.00146	0.007961	0.004061	2735
hsa05130	Pathogenic	184	0.463614	2.302486	0.001466	0.007961	0.004061	3118
hsa05170	Human im	191	0.433764	2.164024	0.001466	0.007961	0.004061	2489
hsa04062	Chemokine	177	0.450634	2.225044	0.001484	0.007961	0.004061	3972
hsa05167	Kaposi sarc	175	0.457778	2.260694	0.00149	0.007961	0.004061	3390
hsa05152	Tuberculos	171	0.443535	2.186616	0.001497	0.007961	0.004061	3393
hsa05135	Yersinia inf	130	0.466263	2.212726	0.001515	0.007961	0.004061	3134
hsa05164	Influenza A	162	0.528438	2.581985	0.001517	0.007961	0.004061	3449
hsa04210	Apoptosis	129	0.416385	1.97077	0.001524	0.007961	0.004061	3891
hsa05206	MicroRNAs	163	0.379579	1.853382	0.001524	0.007961	0.004061	3969
hsa04621	NOD-like r	161	0.473362	2.309382	0.001527	0.007961	0.004061	3365
hsa05203	Viral carcir	161	0.453599	2.212965	0.001527	0.007961	0.004061	4119
hsa05162	Measles	132	0.502592	2.388609	0.001534	0.007961	0.004061	3199
hsa04650	Natural kill	119	0.478762	2.235225	0.001538	0.007961	0.004061	2646
hsa04380	Osteoclast	122	0.55572	2.603478	0.001541	0.007961	0.004061	3664
hsa04514	Cell adhesi	136	0.499722	2.380976	0.001546	0.007961	0.004061	3822
hsa04145	Phagosom	144	0.500462	2.398906	0.001553	0.007961	0.004061	1980
hsa05160	Hepatitis C	144	0.419712	2.011839	0.001553	0.007961	0.004061	3199
hsa05202	Transcripti	158	0.390299	1.891903	0.001558	0.007961	0.004061	3957
hsa04666	Fc gamma	95	0.455184	2.030594	0.00156	0.007961	0.004061	2206
hsa05161	Hepatitis B	157	0.462291	2.240539	0.00156	0.007961	0.004061	3393
hsa04670	Leukocyte	105	0.453519	2.059687	0.001563	0.007961	0.004061	2250
hsa04668	TNF signal	108	0.567175	2.592221	0.00157	0.007961	0.004061	2873
hsa05142	Chagas dis	97	0.496593	2.221176	0.00157	0.007961	0.004061	3972
hsa05145	Toxoplasm	108	0.525696	2.402646	0.00157	0.007961	0.004061	2408
hsa04659	Th17 cell d	96	0.562285	2.511451	0.001572	0.007961	0.004061	3393
hsa04660	T cell recep	96	0.487228	2.176208	0.001572	0.007961	0.004061	3891
hsa04064	NF-kappa	98	0.543215	2.431265	0.001577	0.007961	0.004061	3664
hsa04620	Toll-like re	98	0.488286	2.18542	0.001577	0.007961	0.004061	3972
hsa04625	C-type lec	98	0.421823	1.887952	0.001577	0.007961	0.004061	4081
hsa05146	Amoebiasis	98	0.493575	2.209092	0.001577	0.007961	0.004061	3608
hsa04640	Hematopo	91	0.573388	2.528317	0.001582	0.007961	0.004061	2478
hsa04933	AGE-RAGE	99	0.402418	1.805328	0.001582	0.007961	0.004061	2932
hsa04657	IL-17 signa	86	0.482183	2.109081	0.001597	0.007961	0.004061	2433
hsa04658	Th1 and Th	86	0.556748	2.43523	0.001597	0.007961	0.004061	3375
hsa05222	Small cell l	90	0.449209	1.973763	0.001597	0.007961	0.004061	3136
hsa05323	Rheumatoi	90	0.497879	2.18761	0.001597	0.007961	0.004061	2133
hsa04061	Viral prote	87	0.560678	2.45187	0.001605	0.007961	0.004061	3344
hsa05235	PD-L1 exp	87	0.502732	2.198469	0.001605	0.007961	0.004061	4013
hsa04662	B cell recep	79	0.524783	2.249213	0.001653	0.007961	0.004061	4496
hsa04115	p53 signali	72	0.44478	1.865312	0.001681	0.007961	0.004061	3136
hsa04610	Compleme	77	0.449271	1.910074	0.001681	0.007961	0.004061	2862
hsa04612	Antigen pr	77	0.531802	2.260953	0.001681	0.007961	0.004061	2307
hsa05150	Staphyloco	73	0.635831	2.675075	0.001689	0.007961	0.004061	1994
hsa05133	Pertussis	71	0.575944	2.411382	0.001692	0.007961	0.004061	3134
hsa05321	Inflammato	55	0.597999	2.39961	0.001692	0.007961	0.004061	3934

hsa05140	Leishmania	74	0.655815	2.766026	0.001698	0.007961	0.004061	2373
hsa05416	Viral myoc	58	0.652177	2.63942	0.001712	0.007961	0.004061	2489
hsa05134	Legionello	57	0.518467	2.090567	0.001715	0.007961	0.004061	1387
hsa05320	Autoimmu	44	0.646251	2.468702	0.001715	0.007961	0.004061	2438
hsa05144	Malaria	47	0.555119	2.141365	0.001739	0.007961	0.004061	2133
hsa04672	Intestinal i	42	0.597435	2.25789	0.001745	0.007961	0.004061	1846
hsa04940	Type I diab	41	0.630697	2.371354	0.001748	0.007961	0.004061	2438
hsa05332	Graft-versu	41	0.643325	2.418834	0.001748	0.007961	0.004061	2438
hsa05322	Systemic lu	48	0.703905	2.717372	0.001773	0.007961	0.004061	3181
hsa05340	Primary im	34	0.635036	2.296074	0.001779	0.007961	0.004061	3481
hsa05330	Allograft re	33	0.74187	2.665867	0.001795	0.007961	0.004061	2438
hsa00532	Glycosamir	20	0.686132	2.170337	0.001825	0.007961	0.004061	2986
hsa05310	Asthma	23	0.715703	2.327229	0.001862	0.007961	0.004061	2433
hsa00360	Phenylalan	17	-0.69426	-2.15806	0.002119	0.007961	0.004061	4103
hsa01210	2-Oxocarb	18	-0.71102	-2.26167	0.002128	0.007961	0.004061	3176
hsa00650	Butanoate	24	-0.69103	-2.4153	0.002146	0.007961	0.004061	3434
hsa00053	Ascorbate	23	-0.58496	-2.0157	0.002151	0.007961	0.004061	6866
hsa00790	Folate bios	23	-0.73424	-2.53009	0.002151	0.007961	0.004061	3012
hsa00020	Citrate cyc	29	-0.65004	-2.42062	0.002169	0.007961	0.004061	3109
hsa00630	Glyoxylate	29	-0.70463	-2.6239	0.002169	0.007961	0.004061	3109
hsa00410	beta-Alani	28	-0.68886	-2.5316	0.002179	0.007961	0.004061	3434
hsa00640	Propanoat	30	-0.70713	-2.6339	0.002198	0.007961	0.004061	3434
hsa00220	Arginine b	20	-0.60924	-1.99633	0.002203	0.007961	0.004061	2910
hsa00340	Histidine m	21	-0.71623	-2.38525	0.002212	0.007961	0.004061	2401
hsa00620	Pyruvate m	36	-0.62281	-2.42823	0.002222	0.007961	0.004061	3109
hsa00350	Tyrosine m	33	-0.57379	-2.16565	0.002247	0.007961	0.004061	4373
hsa00260	Glycine, se	35	-0.70918	-2.71997	0.002268	0.007961	0.004061	3142
hsa04975	Fat digesti	35	-0.52554	-2.01564	0.002268	0.007961	0.004061	2829
hsa00250	Alanine, as	34	-0.48688	-1.8407	0.002273	0.007961	0.004061	2864
hsa00330	Arginine ar	48	-0.54645	-2.28205	0.002283	0.007961	0.004061	2864
hsa00380	Tryptophar	40	-0.52245	-2.09249	0.002288	0.007961	0.004061	3109
hsa00071	Fatty acid c	42	-0.6192	-2.49977	0.002331	0.007961	0.004061	3558
hsa00270	Cysteine ar	46	-0.42648	-1.75111	0.002364	0.007961	0.004061	5095
hsa00280	Valine, leu	46	-0.71143	-2.92109	0.002364	0.007961	0.004061	3434
hsa00010	Glycolysis /	57	-0.49924	-2.13173	0.002387	0.007961	0.004061	3195
hsa00310	Lysine deg	59	-0.41218	-1.76672	0.002415	0.007961	0.004061	3109
hsa01212	Fatty acid r	55	-0.43541	-1.83215	0.002433	0.007961	0.004061	5388
hsa04976	Bile secreti	71	-0.39093	-1.76816	0.002433	0.007961	0.004061	3165
hsa03320	PPAR signa	68	-0.46662	-2.0765	0.002439	0.007961	0.004061	4855
hsa04260	Cardiac mu	68	-0.41944	-1.86654	0.002439	0.007961	0.004061	6741
hsa04721	Synaptic ve	68	-0.38511	-1.71375	0.002439	0.007961	0.004061	4212
hsa04740	Olfactory t	73	-0.44595	-2.02481	0.002439	0.007961	0.004061	4806
hsa04146	Peroxisom	77	-0.55244	-2.51621	0.002457	0.007961	0.004061	4719
hsa01230	Biosynthes	65	-0.41514	-1.82127	0.002519	0.00808	0.004122	3408
hsa00190	Oxidative p	98	-0.58924	-2.83034	0.002717	0.008547	0.00436	4310
hsa04151	PI3K-Akt s	321	0.285017	1.512478	0.002786	0.008547	0.00436	3969
hsa01240	Biosynthes	142	-0.39216	-2.01067	0.002801	0.008547	0.00436	3408
hsa01200	Carbon me	110	-0.55856	-2.71545	0.002809	0.008547	0.00436	3195
hsa04010	MAPK sign	274	0.283225	1.482007	0.002841	0.008547	0.00436	3969
hsa04932	Non-alcoh	137	-0.33353	-1.6959	0.002841	0.008547	0.00436	4043
hsa04723	Retrograde	121	-0.32614	-1.61434	0.002849	0.008547	0.00436	4310
hsa04217	Necroptos	124	0.394407	1.857009	0.003053	0.008688	0.004431	3664
hsa05418	Fluid shear	132	0.33732	1.603143	0.003067	0.008688	0.004431	3041
hsa04110	Cell cycle	117	0.376701	1.747675	0.003086	0.008688	0.004431	3597
hsa04630	JAK-STAT s	139	0.368221	1.757681	0.003086	0.008688	0.004431	2987
hsa04218	Cellular se	148	0.367926	1.770102	0.003091	0.008688	0.004431	3910
hsa03040	Spliceosom	128	0.370563	1.747333	0.003096	0.008688	0.004431	5392
hsa04714	Thermoger	189	-0.40121	-2.12892	0.003106	0.008688	0.004431	3877

hsa04611	Platelet act	114	0.382702	1.763407	0.00311	0.008688	0.004431	2746
hsa05012	Parkinson c	203	-0.33347	-1.78819	0.003195	0.008847	0.004513	4310
hsa04020	Calcium sig	205	-0.29873	-1.59991	0.003247	0.008869	0.004524	2833
hsa04512	ECM-recep	82	0.390481	1.680974	0.003257	0.008869	0.004524	1838
hsa05220	Chronic my	76	0.388251	1.650207	0.003373	0.009106	0.004645	4907
hsa04080	Neuroactiv	239	-0.32141	-1.77335	0.003425	0.00917	0.004677	3684
hsa05022	Pathways c	402	-0.23539	-1.3772	0.003774	0.010022	0.005112	4198
hsa00900	Terpenoid	22	-0.55768	-1.88971	0.00432	0.011328	0.005778	5575
hsa05205	Proteoglyc	199	0.304879	1.529085	0.004354	0.011328	0.005778	2646
hsa04966	Collecting	27	-0.53245	-1.92301	0.004396	0.011328	0.005778	4212
hsa04744	Phototrans	20	-0.58383	-1.91307	0.004405	0.011328	0.005778	4399
hsa00040	Pentose ar	26	-0.50507	-1.79088	0.004474	0.011415	0.005822	6866
hsa00561	Glycerolipi	55	-0.40573	-1.70728	0.004866	0.012318	0.006283	3061
hsa05100	Bacterial in	67	0.409493	1.70508	0.005059	0.012706	0.006481	4007
hsa05010	Alzheimer	312	-0.23785	-1.35073	0.00678	0.016882	0.008611	4198
hsa05016	Huntingtor	254	-0.26383	-1.46614	0.006826	0.016882	0.008611	4310
hsa00982	Drug meta	57	-0.38427	-1.64082	0.00716	0.017574	0.008964	5406
hsa04810	Regulation	204	0.292752	1.476441	0.007215	0.017576	0.008965	2900
hsa05211	Renal cell c	65	0.384606	1.597829	0.008264	0.019835	0.010117	3900
hsa05221	Acute mye	65	0.387855	1.611326	0.008264	0.019835	0.010117	3891
hsa04664	Fc epsilon	63	0.405096	1.672824	0.008432	0.020087	0.010246	2206
hsa00830	Retinol me	50	-0.40592	-1.69287	0.009368	0.022154	0.0113	6866
hsa04926	Relaxin sig	119	0.328731	1.534766	0.010769	0.025284	0.012897	4674
hsa05215	Prostate ca	97	0.353007	1.578941	0.010989	0.025615	0.013065	4046
hsa04623	Cytosolic D	56	0.387382	1.556887	0.012007	0.027787	0.014174	3584
hsa04071	Sphingolip	116	0.328696	1.520499	0.012422	0.028545	0.01456	3979
hsa03013	RNA transp	152	0.301364	1.451839	0.013953	0.031837	0.016239	4948
hsa04742	Taste trans	47	-0.40259	-1.6591	0.014052	0.031837	0.016239	4838
hsa00760	Nicotinate	31	-0.43492	-1.63452	0.015521	0.034922	0.017813	4373
hsa00970	Aminoacyl	24	-0.45877	-1.60349	0.019313	0.043155	0.022012	4112
hsa04360	Axon guida	173	0.285733	1.411471	0.019461	0.043188	0.022029	3900
hsa00770	Pantothena	20	-0.50891	-1.66758	0.019824	0.043398	0.022136	3176
hsa04977	Vitamin dig	20	-0.50749	-1.66293	0.019824	0.043398	0.022136	3579
hsa00980	Metabolisr	60	-0.35002	-1.5122	0.021378	0.046486	0.023711	5406
hsa04622	RIG-I-like r	64	0.352051	1.459069	0.021776	0.047035	0.023991	3933
hsa00601	Glycosphir	27	-0.4426	-1.5985	0.021978	0.047158	0.024054	3858
hsa00730	Thiamine r	14	-0.57312	-1.67788	0.022727	0.048445	0.02471	4188

leading_edcore_enrichment
tags=29%, 1906/3918/3091/3576/330/3575/1284/7474/6775/5332/4792/5579/3725/5366/5880/46
tags=51%, 330/7097/4792/3665/3108/3109/9021/718/10379/3115/6890/6347/3455/3119/3118/49
tags=28%, 3918/1284/3694/7474/9586/3371/2535/3685/10379/5925/3065/896/10312/3455/7450/
tags=34%, 6372/2919/2920/3576/6364/3575/3976/3554/58191/729230/56477/7852/9180/6374/14
tags=33%, 3576/330/29108/7846/10109/5603/7097/4792/3725/302/4609/7100/10376/10095/6237
tags=25%, 3918/330/1284/3694/87/3371/5579/3725/5880/64098/3685/896/7450/1286/894/3915/
tags=39%, 3576/3554/5603/9586/5332/4792/5579/5880/4609/7852/4773/3685/3587/5925/3710/6
tags=39%, 4316/3554/9586/3932/4792/3725/4609/3108/3109/4773/5925/896/3115/917/8829/311
tags=35%, 3576/3059/29108/87/3554/10109/5603/5332/4792/3725/7100/718/92610/10095/3710/
tags=43%, 3576/715/5603/7097/4792/5579/3725/6203/718/10379/716/714/2212/51284/629/6136
tags=29%, 5603/5332/5579/5880/2357/10235/1268/1436/3937/3684/4254/6237/83593/2776/2247
tags=41%, 4794/7128/5603/7097/4792/3665/3725/4609/3108/3109/4067/10379/5925/3065/896/3
tags=35%, 9076/3576/1365/1364/3059/29108/3554/7846/10109/5603/4792/3725/7100/10376/10
tags=31%, 5603/7097/4792/5579/3725/5880/7852/25939/9582/4773/3710/6890/917/891/7133/89
tags=41%, 6372/2919/2920/3576/6364/58191/5332/4792/5579/729230/5880/56477/10235/7852/4
tags=38%, 2919/2920/3091/3576/5603/4792/3665/3725/4609/4067/718/4773/10379/5925/3710/3
tags=39%, 26253/11151/5603/7097/3108/3109/718/3587/2212/3115/3684/64581/10312/3119/311
tags=33%, 3576/29108/10109/5603/3932/4792/3725/5880/4773/10095/2212/3937/6347/834/984
tags=49%, 3576/29108/4792/5579/3665/3108/3109/56649/9021/10379/896/3115/51284/6347/34
tags=43%, 330/7846/4792/3725/5366/10376/3710/1439/839/317/2353/71/8797/7185/841/4001/6
tags=39%, 1545/3371/6659/5579/4363/4609/406991/3065/894/4082/6774/6768/3690/994/1945/1
tags=36%, 2919/2920/3576/330/29108/7128/5603/5332/4792/3665/3725/2634/3428/10379/3710/
tags=42%, 87/9586/4792/3665/3725/5366/4067/718/10379/5925/3065/896/5922/894/6774/5966/
tags=41%, 7128/7097/4792/3665/3725/3310/10379/896/51284/917/3455/4939/915/894/317/2353
tags=36%, 3932/5579/5880/4773/962/3937/3455/3383/919/10870/7305/3107/3683/3460/8797/22
tags=48%, 2355/2274/3554/5603/3932/4792/3725/55423/9021/4773/4689/1436/10379/2212/3937
tags=47%, 9076/1001/1462/1365/1364/6402/914/7412/3108/3109/3685/3115/3684/3119/958/311
tags=35%, 715/11151/7846/7097/3108/3109/718/4689/3685/10376/2212/3115/6890/3684/64581/
tags=33%, 9076/1365/1364/4792/3665/4609/9021/10379/5925/975/3455/4939/317/6774/6772/84
tags=40%, 64332/8842/3576/330/64919/4609/1436/3065/3684/958/3207/1848/894/2209/5966/84
tags=33%, 65108/8612/10109/5579/5880/4067/10095/2212/653361/4082/2209/2213/7408/5788/1
tags=38%, 3576/5603/6775/9586/7097/4792/5579/3665/3725/4609/4773/5925/317/2353/6774/46
tags=30%, 9076/1365/1364/87/7412/5603/5579/5880/7852/4689/3684/3702/3383/653361/83593/
tags=44%, 6372/2919/2920/1906/330/6364/3976/7128/7412/5603/9586/4792/3725/9021/6374/63
tags=47%, 3576/5603/7097/5332/4792/3725/718/714/917/713/6347/915/919/6352/2776/2353/46
tags=38%, 3918/330/5603/7097/4792/3108/3109/3310/3587/3115/3119/958/3118/3113/3915/677
tags=52%, 3091/4794/3554/5603/3932/4792/3725/3108/3109/4773/196/3115/917/3119/3118/915
tags=42%, 4794/5603/3932/4792/3725/4773/3937/917/3702/915/919/2353/1326/925/7535/5788/
tags=51%, 2919/2920/3576/330/7128/3554/7412/3932/4792/5579/4067/10673/958/3383/6363/23
tags=44%, 3576/5603/7097/4792/3665/3725/7100/51284/3455/958/6352/2353/1326/23643/4615/
tags=42%, 26253/29108/5603/4792/3725/4773/10379/3710/64581/6237/834/5971/6772/22808/22
tags=41%, 2919/2920/3918/3576/1284/87/3554/7097/5332/5579/912/911/5272/3684/1286/3915/
tags=48%, 3575/3554/914/912/3108/3109/911/1438/100133941/1436/3115/917/3684/4254/3119/
tags=33%, 1906/3576/1284/7412/5603/5332/5579/3725/6347/1958/3383/1286/6774/1282/1536/6
tags=34%, 6372/2919/3934/2920/3576/6364/7128/5603/4792/3725/10758/6374/23765/6347/235
tags=51%, 4794/5603/6775/3932/4792/3725/3108/3109/4773/3115/917/3119/3118/915/3113/919
tags=39%, 3918/330/1284/4792/4609/3685/5925/1286/3915/317/1282/3675/7185/1643/7187/718
tags=36%, 6372/2919/2920/3576/6364/7097/3725/3108/3109/6374/10673/3115/6347/10312/311
tags=47%, 6372/2919/2920/3576/6364/729230/56477/7852/6374/1436/3587/6347/7133/1236/63
tags=48%, 3091/4794/5603/7097/3932/4792/3725/4773/917/915/919/2353/6774/4615/3460/6772
tags=46%, 8519/4794/4792/5579/3725/5880/27071/4067/4773/975/2353/2213/29760/6850/5777/
tags=39%, 2810/5366/896/891/894/317/51246/8797/1643/5054/841/64065/472/581/9134/6241/6
tags=42%, 710/1191/715/11326/718/716/714/629/3684/713/3075/7450/729/2244/717/5329/3078
tags=44%, 3108/3109/3310/3115/6890/3119/3118/3113/5721/567/972/4261/925/3107/5720/3112
tags=51%, 715/3880/3108/3109/2357/718/716/714/2212/3115/629/3684/713/3119/3118/3075/33
tags=54%, 6372/3576/710/715/29108/5603/3725/718/6374/3394/716/714/3684/713/834/2353/23
tags=65%, 6775/7097/3725/3108/3109/7100/3115/3119/3118/3113/3566/6774/3460/6772/3112/3

tags=53%, 65108/5603/7097/4792/5579/3725/3108/3109/718/4689/2212/3115/3684/3119/3118/6
tags=55%, 5880/3108/3109/1525/3115/3119/958/3118/3383/3113/71/3107/3683/942/3112/841/3
tags=32%, 2919/2920/3576/29108/7097/4792/7100/718/3310/3684/834/317/4615/841/929/3606/
tags=50%, 3108/3109/3115/3119/958/3118/3113/3107/942/3112/3126/3105/3133/3134/3117/312
tags=43%, 3576/7412/7097/3820/975/6347/958/2532/3383/7058/4615/3683/6403/3606/3689/229
tags=48%, 56477/3108/3109/7852/10673/5284/3115/3119/958/3118/3113/608/3601/942/3112/31
tags=56%, 3108/3109/3115/3119/3118/3113/5799/3107/942/3112/3126/3105/3133/3134/3117/31
tags=59%, 3108/3109/3115/3119/3118/3113/3107/942/3112/3126/3105/3133/3821/3134/3117/31
tags=69%, 715/87/3108/6737/3109/718/716/714/2212/3115/713/3119/958/3118/729/3113/2209/
tags=62%, 3575/3932/64421/6890/958/915/4261/925/7535/29760/6891/5788/100/3543/916/8487
tags=67%, 3108/3109/3115/3119/958/3118/3113/3107/942/3112/3126/3105/3133/3134/3117/312
tags=60%, 51363/22856/55790/54480/64131/56548/50515/29940/113189/55501/126792/64132
tags=70%, 3108/3109/3115/3119/958/3118/3113/2207/3112/3126/2206/3117/3122/3123/2205/63
tags=76%, 4128/4282/10249/3242/221/137362/259307/6898/1644/4129/218/2805/5053
tags=72%, 587/95/137362/3418/3420/2875/162417/48/51166/2805/84706/50/3417
tags=71%, 18/5019/622/35/6296/39/3030/56898/1962/1892/2571/54988/79944/7915/3155/3033/
tags=87%, 54658/54657/54578/54659/54579/54575/9104/54600/54577/54576/54490/9365/55586
tags=74%, 2643/248/5805/4337/10243/5092/873/1719/84105/250/8836/4338/6697/8644/5053/58
tags=59%, 1738/5162/2271/6392/6389/3418/3420/4191/6390/5106/48/4967/8803/8801/8802/50/
tags=72%, 1738/6470/39/847/6472/81888/4191/51179/189/132158/48/5096/54363/84532/55902/
tags=68%, 18/221/35/1807/51/3030/1962/223/4329/1892/2571/51733/501/84735/218/26275/219
tags=83%, 18/35/1738/160287/39/1629/3945/51/3030/1962/594/4329/1892/79611/55862/5096/8
tags=50%, 95/137362/2747/2875/4843/445/162417/2746/2805/84706
tags=67%, 26/138199/223/4129/501/10841/84735/3176/218/144193/219/217/224/443
tags=64%, 1738/160287/5162/39/2271/3945/10873/223/4191/5106/501/84532/32/134526/55902/
tags=61%, 316/4128/220074/4282/3242/221/7306/137362/259307/6898/1644/81889/130/4129/54
tags=71%, 5224/55349/1738/6470/51268/6472/10993/212/635/23464/2628/5723/189/4129/1321
tags=40%, 39/1056/80168/2169/337/50487/2168/335/81579/84649/5407/84647/4547/38
tags=47%, 8659/137362/259307/2747/2571/2875/445/56954/189/8528/2746/2346/2805/7915/847
tags=50%, 8659/137362/26/2628/5831/223/4942/4843/4129/5625/501/113451/1610/84735/2593/
tags=68%, 169355/316/4128/883/1738/39/847/259307/26/3030/1644/1962/55526/223/1892/2349
tags=69%, 33/1632/2181/1376/35/126129/39/3032/51/3030/1962/223/1892/130/2180/501/2639/
tags=70%, 4143/51074/58478/4190/2937/2730/4548/113675/4507/64902/883/7263/4357/4144/5
tags=78%, 18/197322/5019/587/35/1738/27034/39/259307/3032/1629/64087/3030/1962/11112/2
tags=51%, 5213/221/5224/9562/1738/160287/5162/80201/3945/2645/223/5232/229/130/5236/10
tags=39%, 1738/51268/39/9757/5351/3030/55217/1962/55526/10919/223/1892/501/2639/12368
tags=65%, 9374/9415/9524/54898/84869/23205/6785/8310/23305/6319/6342/33/2181/1376/197
tags=31%, 482/358/5568/760/114/57835/115/10864/9376/2052/8431/9429/107/200931/6256/109
tags=53%, 23205/8310/23305/5468/5467/336/2170/6319/6342/11001/33/2181/1376/5465/12612
tags=72%, 3270/7169/4633/4625/10345/487/59285/4624/785/7137/845/6547/779/1346/1337/782
tags=46%, 528/526/1211/51382/22999/90423/23025/245972/6534/6540/6505/6512/6538/773/57
tags=51%, 390063/8392/4993/805/143503/81472/79339/808/1258/156/282763/26211/26248/138
tags=69%, 8310/23305/7498/8800/196743/5825/5193/390916/4598/255027/6342/11001/5194/83
tags=43%, 4144/5213/587/5224/6470/95/137362/6472/3418/10993/27430/3420/5831/5232/5723/
tags=62%, 51079/56901/528/526/55967/125965/7381/495/51382/4711/7384/4696/90423/374291/
tags=31%, 3918/3575/1284/3694/9586/7097/3371/4609/9180/3685/1436/896/3455/4254/3574/74
tags=38%, 4144/3242/587/1738/2643/248/6470/5805/124454/6472/11019/7390/212/4337/10243/
tags=55%, 5213/5224/35/1738/6470/5162/39/2271/847/137362/6472/6392/6389/80201/2747/51/
tags=31%, 3554/5603/5579/3725/5880/4609/10235/784/3310/1436/4254/6237/5922/1848/1847/2
tags=41%, 55967/6720/51422/125965/7381/4711/54205/3952/5465/7384/3630/4696/374291/153
tags=45%, 51079/56901/10681/3708/2563/55967/3709/4711/22999/2915/747/2560/4696/374291/
tags=37%, 330/29108/7128/6775/10379/3455/834/6774/1536/3460/8797/11035/6772/841/6778/5
tags=28%, 1906/3554/7412/5603/3725/5880/2950/3685/6347/3383/653361/2353/6613/71/3690/4
tags=33%, 2810/4609/5925/3065/896/891/8317/894/4174/994/4171/890/8379/9232/4087/10926/
tags=30%, 3575/3976/6775/4609/9021/9180/1438/3587/10379/896/3455/1439/3574/3566/894/67
tags=37%, 3576/5603/4609/4773/10758/5925/3710/896/891/6237/83593/894/3107/22808/5054/3
tags=47%, 3310/9416/55119/29896/9343/6628/1659/6430/10262/6626/10189/9775/23450/55110/
tags=42%, 51422/125965/7381/353500/5606/4711/137682/2181/1376/7384/4696/374291/1537/1

tags=33%, 5603/5332/10235/4067/64805/3710/2212/3937/7450/6916/2776/71/2244/3690/2207/5
tags=41%, 51079/56901/10105/3708/5683/4128/6622/808/5718/55967/7314/5708/125965/7381/5
tags=31%, 7416/5731/5568/91807/6915/2246/51196/773/55283/5336/816/2902/292/2254/801/11
tags=26%, 3918/1284/3694/3371/3685/961/7450/1286/3915/7058/1282/375790/3690/3675/3696/
tags=45%, 4792/4609/5925/3065/1643/867/1488/581/9846/5594/3845/10000/5970/7157/6776/10
tags=41%, 146/2488/2358/7349/3952/2915/5644/727/2560/9038/1145/153/2918/2912/51052/181
tags=33%, 10105/3708/146754/5683/6622/8326/808/5718/324/53349/55967/7314/5708/1768/125
tags=59%, 10654/91734/23463/9986/4598/23590/39/79947/4597/57107/3422/51449/38
tags=25%, 3091/3059/7474/5603/7097/5579/4609/967/2535/406991/3685/3710/6237/2247/6774/
tags=63%, 528/526/495/51382/1188/90423/245972/760/535/496/534/155066/529/525/245973/65
tags=65%, 805/808/1258/3000/6010/2978/801/5148/91860/2779/6011/2979/810
tags=77%, 54658/54657/54578/54659/54579/54575/6120/729020/54600/54577/54576/54490/936
tags=38%, 196051/64900/1056/80168/253558/223/1607/56894/4023/132158/10327/84649/501/57
tags=34%, 3059/10109/10095/9844/71/391/60/867/10094/10163/7414/63916/2335/8503/10096/9
tags=33%, 10105/3708/5683/6622/8326/808/5718/324/55967/322/5708/125965/7381/5715/5705/
tags=35%, 51079/56901/10105/3708/146754/5683/5718/55967/1211/5708/1768/125965/7381/57
tags=53%, 2947/54600/2953/2944/4257/54577/54576/4259/316/4128/2330/1558/1549/54490/221
tags=26%, 3694/87/10109/5880/7852/3685/7114/10095/3684/6237/2247/71/3071/3690/3675/368
tags=37%, 3091/3725/2113/3082/5594/3845/10000/7428/5062/5063/1026/8503/5908/6513/9915/
tags=38%, 4609/1436/3684/1848/2209/6774/929/890/5594/3845/10000/6932/5970/6776/597/537
tags=29%, 5603/5880/241/4067/3937/2207/5321/27040/6850/2206/9846/5594/7409/2205/240/38
tags=70%, 54658/54657/54578/54659/54579/1577/216/54575/51109/54884/157506/5959/54600/
tags=39%, 1906/1284/5603/9586/5332/4792/3725/1286/2353/1282/1910/2790/4312/4087/59/601
tags=37%, 9586/4792/2950/5925/5156/6692/5154/7184/9134/5594/64764/3845/10000/6932/1385
tags=29%, 29108/4792/3665/834/9447/6352/11035/3606/23586/103/3553/5970/3551/9641/6351/
tags=36%, 5603/5332/5579/4363/5880/91012/130367/2776/2207/140/2206/1901/10672/581/887
tags=38%, 6613/29107/387082/1973/23191/79902/8672/10250/11171/96764/53371/55706/13243
tags=57%, 775/50834/6338/80835/954/8645/3351/9568/5726/170572/9033/5568/773/114/255022
tags=55%, 316/23408/56953/133686/83594/5169/4860/23530/4907/23475/93100/54981/683/554
tags=67%, 23438/57505/54938/55278/123263/10352/118672/55157/124454/55699/79731/57038/
tags=33%, 7474/10509/5880/7852/4773/8829/6237/10507/2051/1945/1072/1969/1946/10505/39
tags=45%, 587/5169/1807/51733/79717/53354/219/217/224
tags=50%, 8029/5948/337/335/9963/6573/2346/25974/113235/686
tags=48%, 2947/54600/2953/2944/4257/54577/54576/4259/1549/54490/8574/221/1548/22977/2
tags=31%, 3576/5603/4792/3665/54941/4214/841/7187/23586/9636/338376/5970/8772/3551/964
tags=48%, 8704/2524/53947/6487/28/2651/2528/10402/2525/8702/2529/2527/79369
tags=64%, 205/79178/248/52/84284/250/204/9054/249

09/2535/10235/7852/2950/3685/1438/650/1436/5925/3065/896/3455/4254/1439/3574/1286/8359
39/10308/3113/6352/567/317/972/3690/4615/3107/3460/6772/6672/3112/80264/841/389114/689
1286/894/3915/7058/1282/3690/3675/3107/6772/841/3696/4599/3105/3676/3659/3133/890/8032
38/650/10673/1436/3587/23765/6347/3455/3624/958/1439/7133/3574/1236/9235/3566/6352/636
/834/388/9844/6281/9266/2353/71/23643/3071/4615/399/203068/8797/11035/841/929/3606/391
7058/1282/71/3690/3675/7408/3696/3676/60/5156/2316/5154/3911/5500/55742/3082/7791/4638
890/6347/6352/567/2776/6774/1234/3690/3107/841/6891/3105/3133/2790/5156/811/3134/113/1
9/958/3118/1958/3383/915/3113/706/894/567/2353/3601/5971/3107/4214/3683/3112/3126/3105
834/6352/9844/9266/71/4615/7322/929/3606/5216/60/8767/837/114548/960/472/7188/10094/58
/713/8829/6347/3455/834/7450/729/4939/2353/6774/6868/200916/6223/2244/1536/4615/51311/
/71/3690/2903/3683/1945/22808/51466/54518/51735/7408/5216/60/5900/3689/27040/5156/1969
115/6890/917/3455/3119/958/3118/3383/4939/915/3113/919/894/567/317/6774/4615/5971/3107
95/2212/64005/834/4542/9266/2353/55971/71/3071/4615/203068/8797/4642/841/3606/6188/60/
06/915/919/567/2776/2353/1234/4615/3107/85363/841/6891/3105/3133/60489/1072/2790/811/8
067/6374/6347/3702/1236/653361/6352/6368/9844/2776/6363/6774/10563/1234/6366/1794/677
455/3383/2247/2353/6774/1234/3107/6772/942/841/3105/3133/7187/2790/6850/3055/3134/7538
8/3113/317/2209/972/4261/4615/3460/6772/2207/1263/4360/2213/3112/841/929/3606/3126/910
/2776/2353/71/4615/925/7535/3606/391/3676/60/27040/114548/10094/51135/5594/7409/10163/
5/3119/3118/834/3383/4939/3113/6352/317/8766/71/4261/4615/3460/8797/6772/3112/841/3606
0/9451/1520/84790/1616/472/581/5594/332/4170/3845/10000/8743/836/4217/3002/5970/7132/3
786/7078/5156/1946/54541/960/23405/27086/5154/472/3925/1788/2146/7431/9839/9134/5594/4
6347/3455/834/4939/9447/114769/6352/84674/10135/1536/4615/11035/6772/841/10628/3606/8
1234/3107/7185/6672/841/3105/3133/890/9734/7187/8379/6850/5315/3134/85477/7188/581/753
/6774/4615/6772/2213/841/4478/4599/9451/4600/7187/23586/868/3560/581/51135/9134/10399/
07/7535/3105/3133/3689/27040/22914/6850/3821/3822/5777/5594/7409/3824/3135/2215/3845/4
/3455/653361/2209/2353/7305/3726/3690/5971/3460/6772/2213/29760/4688/9103/6850/814/108
8/79679/3383/3113/925/3107/3683/6403/942/9019/3112/3696/3126/3105/3676/3133/6404/5788/
10312/3119/3118/653361/3113/7058/2209/71/1536/3690/3107/203068/4360/2213/3112/929/6891
1/4599/9451/4600/7187/23586/581/7534/7533/5594/8554/3845/10000/836/5970/7132/355/7157/
64/2120/942/7185/1643/604/929/25942/890/4297/4300/6692/5154/472/2005/51274/3560/581/32
072/5321/27040/6850/3055/3985/85477/10094/8877/9846/5594/7409/50807/10163/2215/10000/
15/4214/6772/1643/841/6778/890/7187/23586/581/51135/7534/9134/5594/64764/1959/332/3533
71/1536/399/3683/7408/4478/4688/3676/60/3689/7409/2770/50848/6494/10627/7414/2771
47/7133/3383/6352/2353/1326/3726/11035/7185/841/3659/7187/197259/64127/8809/7188/6401/
15/3460/5054/841/4087/811/51135/5594/2770/916/10000/3553/2771/5970/7132/355/8503/7046/8
4/23643/1234/4261/4615/3460/6772/3112/841/3126/3117/3911/51135/3122/5594/3914/2770/312
/3113/919/3566/2353/6774/9466/3460/6772/7535/3112/6778/3126/27040/4087/3117/3560/3122/
27040/5777/868/8440/5594/7409/10125/916/3845/10000/5062/5970/5063/8503/1493/29851/3551
643/4615/5971/6366/7185/7535/29760/929/27040/7187/6850/23586/472/7188/51135/353376/405
51311/6772/942/841/929/7187/51135/5594/7096/6696/353376/10000/3553/5970/8503/7098/8772
207/1263/841/3659/6850/114548/868/5594/1959/3845/10000/3553/5743/5970/1960/8503/3551/96
2776/1282/929/913/3689/3911/3914/3553/836/7414/5970/10319/5869/2335/8503/7850/735/7099
3118/3574/915/3113/3566/2209/3690/925/3675/3112/945/929/913/3126/3676/931/951/960/3117
772/5054/4087/581/6401/5594/3845/10000/3553/836/5970/7056/6776/2335/8503/7046/3552/230
/841/6280/7187/4312/7188/7184/5594/3727/3553/836/5743/6279/5970/6356
/3566/2353/3460/6772/7535/3112/6778/3126/27040/864/3117/3560/9794/3122/55534/5594/3123
8/3911/581/9134/3914/10000/836/5743/5970/10319/7157/3673/5728/1026/2335/8503/1871/3551
/3118/3383/3113/6352/2353/3683/942/3112/3606/3126/3689/4312/3117/3122/3123/4050/3553
2/6368/6363/10563/1234/6366/8797/5197/1235/3606/51554/8809/3560/6846/1524/3570/6375/87
/7535/27040/5777/55509/29126/5594/10125/916/353376/3845/10000/5970/10538/5728/4215/850
10859/5594/7409/3845/10000/118788/3635/5970/11006/8503/10288/3551/5534/930/4790/4893/5
4393/8493/11200/836/355/7157/637/5728/1026/92344/8795/1021
/5054/2/3689/3080/2266/10877/1604/966/7056/730/10878/720/721/728/735
/6891/3126/3105/3133/1520/3309/811/3821/3822/3134/3117/10437/3122/3123/3824/3135/3106/
83/3113/2209/3875/717/3683/6403/2213/3112/3126/6404/9103/25984/3689/2266/3117/2359/312
643/4615/717/929/3659/3689/1072/114548/51135/5594/2770/10392/353376/3553/836/2771/5970
606/6778/3126/4087/64127/8809/3117/3122/3123/2625/3553/3594/8807/5970/3552/7099/7042/7

53361/3113/2209/2353/1536/4615/3460/6772/3112/4688/3126/3676/9103/3689/5777/3117/5113
126/3105/60/3133/3689/3134/3117/8672/3122/1604/3123/3135/3106/836/5551/637
3689/1937
2/3123/3135/3106/3002/355/5551
14/3082/6401/2995/3553
26/3676/3117/3122/3123
22/3123/3135/3553/3106/3002/355/5551
22/3123/3824/3135/3553/3106/3002/355/5551
717/2903/942/3112/3126/3117/6628/3122/3123/2215/730/720/721/735/2214/733
6/3718/29851/5993/920/930
2/3123/3135/3106/3002/355/5551

56

38
/223/10327/501/2990/219/217/224
60/249
3417
9380/275/2653/50/84693/38
/217/224
4532/32/55902/26275/8803/8801/8802/84693/38

5313/9380/219/217/197257/224/38/3029
409/218/2184/2805/1621/128
58/29968/501/1491/1610/29958/2593/9380/275/2653/1757

706/443
79814/58510/2805/112849/219/217/112483/224/8974
8/4129/501/8942/2639/51166/219/8564/217/3033/224/38
31616/128/219/10449/217/30/10455/3033/224/36/34/38
37/160287/23743/137362/259307/3945/6898/10993/635/27430/191/4191/29968/23382/1491/2729
23/594/4329/1892/501/5096/549/56922/26275/219/10449/217/30/3155/3033/3712/224/84693/36
327/2203/5106/501/84532/55902/218/5313/2538/128/219/130589/217/224
3/84787/9739/51166/219/217/3033/224/38
322/35/126129/39/3032/7923/51/3030/1962/27349/1892/54995/2180/60481/81616/51102/10449/
98/6580/6555/123264/3781/1244/1576
9/123/2169/51/1962/10873/2168/335/2180/4023/116519/5106/1593/81616/6256/364/2710/10998/
/23439/1340/9167/1339/775/84701/778/7134/27092/125965/7381/93589/444/786/7384/1537/482
030/6530/10497/10814/594855/535/160/534/6511/6532/155066/529/525/245973/127124/6809/58
304/3000/26658/26740/158131/5568/816/26476/26689/801/4992/26664/81285/79541/91860/815/
594/2181/5189/215/5052/5824/51268/847/10005/5826/2053/55825/51/3418/11264/1962/5191/92
2875/445/229/29968/162417/1491/48/5313/2805/5053/84706/50/3417
/1537/10975/4728/245972/7385/64077/6392/6389/4709/4700/4720/9377/7386/4716/4701/535/49
50/1286/3566/894/3915/7058/2247/1282/3690/3675/1945/3696/3676/374/2790/5156/1969/6850/
2235/27430/23475/1719/4522/4351/54995/9249/23498/250/204/8836/55163/93100/10327/4338/
3418/3030/10993/1962/3420/10873/2645/4329/5232/5723/1892/2875/4191/51179/229/189/13215
247/2353/1326/4615/994/5971/4214/1945/22808/929/374/5321/6789/5156/1969/1946/11184/161
7/10975/4728/7385/6392/6389/4709/4700/6945/4720/9377/7386/4716/4701/4704/51094/4712/47
/4728/3763/5568/4709/773/4700/4720/57030/114/4716/4701/115/5330/4704/4712/4713/3760/47
321/197259/114548/7188/581/353376/55072/8743/3553/29082/7132/355/6776/5834/637/823/37
688/60/387082/4205/5154/7184/6385/1843/6401/10000/3553/4217/5970/7056/7132/7157/659/85
7272/472/7534/9134/7533/991/23594/11200/7157/4172/1026/1871/996/7529/4175/4176/1021/10
74/3601/9466/3460/6772/6778/10401/5156/5777/5154/3560/5771/3570/8554/4170/8651/10000/
105/3133/890/2113/677/4087/3134/472/5500/9134/5594/3135/3845/10000/3106/11200/5970/71
/10946/51690/151903/8175/10915/220988/3178/10450/4116/6633/6632/57819/25949/23350/730
0975/29078/91942/2475/4728/126129/7385/5568/6392/6389/10818/4709/4700/4720/55811/6009/

4518/7408/83706/60/5321/2266/6850/113/10672/5500/5742/4638/5594/2770/10125/10627/1000
5715/5705/3709/81027/4711/54205/5694/7384/4696/5717/374291/1537/10975/4728/5701/7385/7
4/3360/488/4914/26291/155/5737/8911/8822/115/2064/8913/5330/3363/5733/291/1956/3361/29
3676/960/3911/6385/3914/6696
26/8503/1871/7046/3551/1021/7042/7040/4790/4893/4193/5293/2122/4616/4089/208/578/613/6
5/3640/553/3972/5697/9294/11255/1135/1392/5731/6752/5020/2692/6915/8973/2688/2689/2902
5965/7381/5715/5705/3709/4041/5606/81027/4711/25981/54205/2915/5694/27123/5861/7384/46

71/10855/3690/5329/22808/4060/4478/60/80326/7078/4087/960/1839/867/5777/2316/5500/6385
21/127124

5/51084/6652/10327/2990/51181/9942/27294
7104/2710/5407/219/217/224/80339/26007
99/3678/858/3611/5293/4233/857/10093
3709/4041/1147/81027/4711/54205/2915/5694/27123/57142/7384/3630/4696/5717/7855/374291
15/5705/81027/4711/25981/54205/2915/5694/7384/1387/5432/4696/5717/374291/1537/10975/24
/1548/2326/2948/130/2329/4129/1565/2938/9446/1555/218/128/4258/2940/1576
3/22808/4478/3696/5216/3676/60/3689/1072/5156/23191/3985/5154/85477/10672/5500/10094/4
405/7042/2033/7040/7030/7039/4893/5293/4233
1/8503/3551/7704/4790/11040/3728/862/4893/5293
45/10000/3635
54577/53630/1562/54576/316/1558/1549/54490/56603/1548/8228/54905/9249/130/1555/8608/12
9/113/54331/5594/64764/2770/408/3845/10000/2782/2771/1385/5970/8503/7046/7040/4790/128
5/5970/7157/367/5728/1026/8503/1871/7850/2308/3551/2078/2033/4790/2119/7039/4893/4193/5
4790
7/9846/5594/2770/2205/1903/3845/10000/8879/2771/5529/4217/5970/7132/7157/637/5728/8503/
0/10189/10762/9775/7514/26986/55110/10460/9688/8021/7329/9818/9631/22794/79228/5903/54
2/5330/2911/1131/3355/2914/5153/2784/3354/6335/3352/22953/6337
235/349565/23410/23409
10667/80222/51091/5188
35/8440/1808/6091/23380/5594/2770/6586/6469/5998/2242/3845/10627/57556/2041/29984/1942

948/873/130/1565/2052/2938/9446/1555/218/128/4258/27294/1553/2940/1576
41/7706/4790/1540/10010/64135

)3/23401/3566/894/3915/2776/2247/317/2353/6774/3601/1282/3675/3460/6772/7185/1910/1643/
)1/3126/94039/3105/3133/9451/7695/148156/7187/146198/54811/6850/811/58500/79088/3134/2
26/4600/7187/5315/3134/472/3911/9636/581/9794/9134/55534/5594/64764/9519/3914/6696/3135
58/608/6363/3601/10563/1234/9466/6366/3460/8797/5197/1235/3606/3603/939/51554/8809/951
1/5216/6188/60/5788/8767/197259/23191/837/114548/84790/2316/7184/10094/581/84617/51135/
/5594/7409/3914/6696/10627/10000/7414/5062/10319/5063/3673/5728/2335/8503/5908
0672/7188/54331/581/5594/64764/3570/5732/2770/11214/3135/3845/10000/2782/3553/3106/836
/3133/890/2113/3689/8379/9232/4087/811/2114/3134/113/3117/472/7538/2005/3560/200186/58
1/5594/10163/10392/55072/10627/10000/3553/3098/7414/26100/826/9252/5970/7132/7157/6391
717/6403/6772/4599/6188/6168/6202/4600/7187/4312/2266/6850/114548/23521/1839/2197/2358
9/1946/113/5154/9771/3082/5594/23683/7409/1902/2770/6494/3845/10000/9170/1942/2771/5574
/3683/6772/1643/29760/3112/841/6891/3126/3105/25942/3133/890/7187/864/6850/811/953/960
/23191/4641/837/114548/84790/5777/10972/10672/8440/10094/581/84617/51135/5594/1902/101
905/3134/3985/472/7188/54331/581/51135/27350/684/5594/2770/916/3135/3845/200316/10000/
2/5197/1235/2268/2790/3055/113/54331/6846/1524/5594/7409/2770/408/6375/10663/3845/1000
3/54331/581/5594/3135/3845/10000/2782/6932/4277/3106/836/5743/1385/5970/7132/355/7157/1
)3/8767/3689/1520/6850/64127/3117/581/8877/51135/3122/5594/7096/3123/2215/10000/3553/83
/10000/3553/5970/63916/2335/8503/10096/3551/7099/9815/148022
5/4599/3126/29107/60/4600/7187/5611/114548/23586/64499/3117/581/51135/3122/5594/3123/71
55/7157/4000/5551/3563/637/823/597/8503/8772/824/1509/3551/8795/1519/84823/142/9020/479
4170/3845/8651/1942/836/5743/1789/9252/7329/7157/5728/659/1026/8503/1871/4853/90427/355
767/7187/837/114548/64127/7188/51135/5594/115362/10392/7158/55072/3553/118429/22900/59
4/9134/7533/5594/64764/1959/9519/991/3135/3845/3106/836/1385/5970/7157/6776/1026/1960/
916/6504/103/10000/3553/836/5970/355/7157/6776/27102/637/8503/3718/8772/3552/3551/7099
4068/8743/3384/4277/3106/836/3002/355/5551/637/8503
359/9846/5594/3727/4982/2215/8651/10000/3553/1385/5970/7132/11006/8600/8503/10288/7046
3689/57502/3134/3117/6385/3122/6401/29126/5792/50848/3123/3135/6614/3384/3106/3897/923
1/4688/3126/3105/60/3133/9103/3689/1520/811/84790/3134/3117/84617/3122/9146/3123/3135/2
27102/637/1026/8503/1871/6041/7098/7529/8772/3551/148022/1021/9641/10971/5610
06/55589/2313/4086/3002/5970/5079/7157/597/1026/2530/5371/5090/6667/7850/2308/9915/421
3635
376/3845/10000/836/1385/5970/355/7157/6776/637/1026/1960/8503/1871/7046/3718/7098/7529/

/5594/64764/10000/3553/836/5743/4217/1385/9252/5970/7132/355/8503/4323/8772
8772/3551/7099/148022/7042/624/7040/4790/5293/6348/6349/414062
23/240/8651/10000/836/2771/5970/10319/7132
5594/3570/3123/916/2625/3553/3594/5970/6776/7046/3572/3718/3662/3551/920/5534/7040
1/920/5534/9020/4790/5588/4893/5293
50/3553/5743/5970/7329/7132/8600/597/23085/3551/7099/148022/6351/6357/7706/142/9020/47
2/3551/7099/148022/9641/6351/6373/4790/5293/6348/6349/414062
541/5534/9020/4790/1540/4893/4193/5293/30835/602
/3908/733/7042/7040/4790/1281
/3122/1604/3570/3123/916/3553/966/952/3563/3673
)8

3/916/2625/3594/5970/6776/4853/3718/3551/920/5534/182
1/3908/1021

743/8807/7132/6356/3572/3588/8795/6351/6373/6357
)3/3551/7099/148022/920/5534/4790/5588/4893/5293/1460
5293/971/11027/11025/208

1385
2/3123/2215/3872
)/720/721/3552/7099/148022
7040/4790/50615/30009

5/3122/5594/3123/2215/3553/5743/5970

0/2805/55256
/34/38

30/3033/36/34/38

/30/8309/34/345
2/10975/7385/4635/4634/9377/7386/488/27091/783/1350/1351/1329/1349/27089/7139/29796
64
/5153/26716/2788/7932/126370/83988/818/810
960/1891/4843/51179/2180/189/26063/283927/8528/1610/54363/5827/26061/5830/6647/30/3155

6/534/4704/4712/4713/4723/479/4714/4724/155066/529/525/4722/245973/6390/4715/127124/47
1946/54541/5154/4602/3911/3560/7184/54331/200186/3082/7534/9134/7533/5594/64764/1902/3
29968/8942/6697/9054/2729/79717/51805/2990/5313/554235/84274/53354/1723/219/8564/217/5
58/6390/29968/2203/48/5096/2746/54363/84532/4967/55902/5313/2805/26275/8803/128/275/265
16/2316/5154/3925/2005/1846/1850/3082/1843/51135/5594/3727/408/10125/1844/3845/10000/19
13/4723/4714/3643/4724/4722/6390/4715/4705/4731/1350/1351/4694/4702/2931/1329/5313/625
23/2911/4714/4724/2166/4722/2784/2892/4715/2788/4705/4731/2570/4694/107/4702/57406/630
18/5836/7098/8772/10616/3552/824/8795/7099/148022/5610/142/8605/2752/1540
503/7850/3552/2952/3551
0971/7042/2033/7040/6502/29945
3594/338376/6776/3563/1026/8503/3572/3718/85480/3588
57/5728/823/1026/678/8503/1871/7046/2305/3552/2308/824/1021/7042/5534/7040/90550/20485
7/3192/51645/494115/23020/27339/2521/10285/8559/4686/57461/9410/7919/58517/9129/27316/
/6199/9377/7386/114/26291/4716/155/4701/115/4704/4712/4713/25915/4723/3991/4714/4724/21

0/2771/84876/3673/5584/8503/5908

416/5568/9927/6392/7326/6389/147700/4709/4700/816/4720/10131/292/9377/801/7386/4716/47

25/2911/91860/887/4843/1131/2250/2905/219931/5260/3274/815/5153/84812/56848/3362/107/3

73

2/1136/7434/117579/3360/4543/256933/165829/155/5737/83551/3363/5733/3361/2925/113091/5

96/5717/7855/374291/1537/10975/2475/4728/5701/7385/7416/6712/847/5621/9927/27035/6392/

5/3082/8826/5594/7409/6469/3845/10000/836/7482/355/7157/3673/2066/1026/2335/8503

/1537/10975/2475/4728/5701/7385/7416/27035/6392/6389/147700/4709/4700/4720/2902/292/93

75/4728/5701/7385/3766/7416/6392/1742/6389/23186/147700/4709/4700/4720/2902/292/9377/7

4638/8826/5594/7409/1902/10163/3845/10627/9170/54961/7414/5062/55740/5063/3673/2335/85

28/145226/10901/317749/1551/1576

31/2791/4893/2783/5293/10488/111/1285/208/109/1288/112/1278/4313

5293/2260/5159/10488

/5525/1509/9517/624/8439/8560/4790/4893/5293/259230

4960/80336/4116/10482/3837/57510/10921/2521/9669/8761/4686/1207/7919/8664/1968/79760/2

/54961/2048/2771/3897/5062/55740/5361/5063/57522/659/8503/5163/7220/59277/91584/2047/6

/841/6778/890/2113/80326/5900/7187/2790/4312/6789/4087/5156/867/113/1488/5154/10672/718
3586/1616/3117/7188/5500/7694/136051/581/51135/6430/65243/90649/3122/51427/684/146540/
5/3845/1741/10000/6932/3280/26508/8638/3106/836/5743/5529/7482/1385/5970/10319/7132/35
8/3560/6846/1524/3570/4982/6375/10663/8771/4050/8743/3553/3594/944/338376/8807/7132/35
/5594/23207/10392/10627/10000/6932/8743/3553/836/5970/7132/63916/5063/29109/5869/27072

5/5743/2771/1385/5970/7132/355/7157/637/1026/8503/1871/6667/8772/3588/3551/1021/6351/55
1/9134/3122/5594/64764/1959/9519/3123/991/916/3135/3845/10000/64784/3106/7514/11200/13
16/11337/823/8503/10096/10616/23048/2308/9265/824/3551/7099/7321/4671/9181/831/4790/10
36/9636/51135/6146/5594/3570/103/3553/6158/5970/730/7132/11224/25873/8503/6187/6229/35
40/57568/5584/135/8503/10636/25865/5908/8631
/3134/23586/3117/7188/9636/7431/581/51135/9134/3122/3123/5713/916/3135/10000/3280/3106
.63/9170/8743/3553/836/4644/5062/5970/7132/355/5063/9871/10096/8772/9265/3551/8795/7099
/2782/3106/836/2771/5062/5970/7132/355/5063/637
0/2782/2771/5970/6356/8503/3718/5908/57580/3551/6351/6373/6357/4790/7074/2791/56288/63
.1337/637/1026/8503/1871/3572/7098/57580/8772/3551/9976/148022/1021/9641/5610/5534/203
36/1385/5970/7132/637/5869/8772/3552/3588/1509/2214/7099/5993/7042/5534/2033/7040

177/3838/103/10000/8743/3553/7514/836/11100/5970/7132/355/637/5371/8503/6041/7098/8772
90/1522/4893/5293
51/6624/1021/7042/2033/4790/4194/3678/5598/4893/4193/9759/5293/4233/5159
970/11337/6041/8772/10616/3551/7099/148022/9641/2633/4671/9051
/8503/3572/3718/7529/1021/10971/5610/2033/6502/4790/4893/4193/9759/5293/3516/10488/2968
9/1021/9641/5610

5/3552/3551/2214/7042/5534/7040/4286/9020/4790/1540
3/9369/1493/29851/5819/201633/920/5175/4267/999/23705/8516/23114/83700/80380
2215

.1/2078/4286/7704/7030/4790/8148/2119/3728/862/4193/4233/2521

/8772/3551/7099/148022/9641/10971/7042/2033/7040

90/1540

5/10455/1384/10901/5264/8309/3417

705/4731/1350/1351/4694/4702/1329/4697/1349/27089/4717/4695/29796/4706
3570/3914/6696/4170/3845/10000/118788/2782/9170/1942/5529/1385/5970/10319/7157/3563/36
57026/145226/51004/349565/224/249/210
53/4524/8801/84706/8802/50/84693/26007/414328/38/3417
942/3553/55799/836/5062/4217/9252/5970/7132/355/7157/2066/11221/4915/4215/7046/5908/35
56/4697/1349/27089/4717/4695/51085/29796/4706
0/5582/4697/4717/4695/4706

1/4790/4893/4193/5293/55957
3183/144983/6427/9716/57187/22827/51691/6431/3190/25766/1655/6432/3304/4670/6428/6636
180/4722/4878/6390/7248/4715/388753/285521/4705/4731/1350/1351/4694/107/4702/57104/132

'01/4704/291/4712/4713/4723/4714/91860/118424/4724/815/4129/4722/51807/6390/5693/4715/4
3706/2251/5582/340156/147/22953/56413/818/53373/8912/7423/7422/810/1950

7053/90226/2911/64106/1394/23566/887/1131/6755/5645/2905/2831/3355/5443/3274/3827/114[illegible]
'1742/7326/6389/147700/4709/4700/816/4720/342371/2902/10131/292/9377/801/7386/7473/841[illegible]

377/801/7386/7473/488/5664/4716/50507/4701/5330/4704/348/291/4712/4713/7472/55062/4723/
'386/4716/10540/4701/5330/160/4704/10120/291/4712/4713/9001/55062/4723/4714/10671/4724

03/1730/10096/221178/9459

3435/3646/10799/8563/4928/8666/51808/9972/1965/57187/8663/6612/8665

5259/5534/1948/103910/56288/3983/4893/3611/5293/4233

38/3911/3560/7184/54331/581/3082/9134/5594/1902/3570/3914/5732/2770/332/10125/6469/384
/81931/84914/3123/10224/3135/7752/7700/91975/10189/10000/163227/339559/162966/3553/759
5/7157/3673/5584/5728/1026/10134/2335/8503/4853/7098/8772/5525/8324/2308/1293/3551/390
55/6356/3563/8600/659/7046/3572/94/7850/3552/85480/3588/8795/1441/6351/6373/7042/920/63
2/8655/10096/64837/8772/9265/3551/8795/7099/4671/51626/347688/4790/103910/4074/5287/55

534/4790/2791/5734/6376/4893/4193/2783/5293/6348/6349/414062/10488/111
385/5970/7132/7157/6776/5728/1026/4215/8503/1871/7046/3718/996/6513/7850
3910/7307/3678/5588/7335/4193/3611/5293
72/720/721/728/7098/6175/735/6193/6181/6169/3551/7099/9641/733/5610/6157/6142

5/836/5970/355/7157/637/8819/1026/8503/1871/3718
9

376/4893/2783/5293/7454/6348/6349/414062
3

/3552/3551/8795/7099/148022/1021/9641/5610/2033/10482/7706/8106

3

73/2066/5728/4915/1026/2335/8503/3718/7529/10161/5525/1293/3551/7099/3908/1021/10971/1

52/9448/3551/7042/2323/5534/7040/57551/8605/9020/4790/8569/5598/7039/100137049/4893/42

9/79133/8110/6300/493753/4697/84987/1349/65260/27089/788/4717/51287/4695/51103/29796/4

4705/4731/79861/1350/1351/4694/4702/1329/1813/4697/1349/27089/818/4717/65018/4695/2979

5/2914/1144/2147/2642/2892/3354/2696/2901/6753/5646/7442/2570/3362/187/4985/5340/1141/1
34/488/5664/4716/10452/10540/50507/4701/596/5330/4704/10120/291/4712/4713/7472/9001/55

/4714/91860/177/4843/7480/1131/3643/2905/4724/4023/4722/51807/6390/5693/4715/10297/4705
/4294/4722/51807/2892/6390/196385/5693/4715/4705/4731/79861/1350/1351/4694/4702/27113/

5/10000/2782/6932/10023/3280/9170/26508/7428/836/3594/5743/2771/7482/9252/5970/10319/3
94/3106/63934/836/10838/81856/90321/55769/79230/25799/7551/5970/115509/7132/90338/7743
08/148022/1021/9641/5610
57/7040
770

[illegible]441/8115/2323/930/4790/2791/8516/3678/7039/4893/4193/2783/5293/4233/2260/2252/5159

[illegible]33/2260/2252/5159

[illegible]706

6/810/4137/4706

1325/1813/7067/1137/1443/5618/3352/147/22953/56413/2740/133/8862/7068/151/2690
062/4723/2911/4714/91860/177/4843/7480/1131/10671/118424/2905/4724/815/4294/4722/5180[illegible]

5/4731/5481/79861/1350/1351/4694/7479/4702/1329/7477/8322/4697/1349/27089/4717/4695/43
/1329/5435/4697/1349/27089/6647/4717/4695/2880/29796/4706

[illegible]55/7157/6776/3563/3673/637/367/5728/1026/5371/2335/8503/1871/7046/4853/3572/3718/6513/
[illegible]/355/7157/125893/26152/27102/342909/637/284307/9831/84671/91661/5371/8503/140612/6041

7/2892/6390/196385/5693/4715/11273/10297/4705/4731/5481/79861/1350/1351/4694/7479/4702

11/29796/810/4137/4706

/6667/10161/8772/8324/2308/2952/8202/3551/3908/1021/9915/1441/405/1909/7042/2323/624/1[illegible]
[illegible]/57547/5199/126017/163049/7098/7554/9534/8772/7559/3551/5819/84924/148022/90333/9641/[illegible]

/1329/7477/6300/5582/8322/4697/1349/10133/27089/818/6647/4717/65018/4695/2880/29796/81

[illegible]12/182/2033/7040/4286/7704/6502
[illegible]610/7699/349075/10172/10482/7539/170960/80818/4790/93134/29992/79788/57711/7773/4405[illegible]

.0/4137/4706

15/55659/3678/7767/163050/5293/7678/64135/55552/284406/9668/57343/121274/57335/92595/[illegible]

[illegible]451/284390/163081/7673/10520/80110/80778/57693/3459/282890/208/7633/345462/4938/3429[illegible]

26/578/7568/148268/90594/25888/6427/84527/26974/10793/1965/84874/7562/126231/6431/148[illegible]

103/6432/730051/55900/7738/79862/51710/3125/3127/93474/7637/207/6428/147694/8890/7549/

'3654/7771/147923/5499/3454/374900/440275/55762/7581/8427/3717/7730/57506/3593/7124/10

780/7582/7761/158431/84449
