# Supplementary material for: The Potential Diagnostic Value of Immune-Related Genes in Interstitial Fibrosis and Tubular Atrophy after Kidney Transplantation
Source: J Immunol Res. 2022 Jun 17;2022:7212852. doi: 10.1155/2022/7212852 (PMC9232312; doi:10.1155/2022/7212852)
Supplement: Supplementary Materials — Supplementary Figure 1: GSEA enrichment analysis of the IF/TA group. Supplementary Figure 2: correlation analysis between ANGPTL3 and differentially expressed immune infiltrating cells. Supplementary Figure 3: correlation analysis between APOH and differentially expressed immune infiltrating cells. Supplementary Figure 4: correlation analysis between EGF and differentially expressed immune infiltrating cells. Supplementary Figure 5: correlation analysis between FCGR2B and differentially expressed immune infiltrating cells. Supplementary Figure 6: correlation analysis between HLA-DQA2 and differentially expressed immune infiltrating cells. Supplementary Figure 7: correlation analysis between LTF and differentially expressed immune infiltrating cells. Supplementary Figure 8: IPA analysis shows the interaction network of diagnostic genes: EGF and LTF (8A), ANGPTL3 (8B), FCGR2B and APOH (8C), and HLA-DQA2 (8D). Merged the above four independent networks to comprehensively analyze the interaction of diagnostic genes (8E). Supplementary Table 1: immune-related genes. Supplementary Table 2: KEGG pathway in normal group. Supplementary Table 3: pathway of ANGPTL3 gene. Supplementary Table 4: pathway of APOH gene. Supplementary Table 5: pathway of EGF gene. Supplementary Table 6: ingenuity canonical pathways. Supplementary Table 7: category. [file 7212852.f1.zip › 7212852.f1/supplementary table12.pdf]

Ingenuity (	-log(p-va	Ratio	z-score	Molecules
Granulocyt	1.01E+01	7.51E-02	NaN	CCL19,CCL4,CCL5,CXCL1,CXCL10,CXCL11,CXCL2,CXCL
Agranulocy	9.52E+00	6.74E-02	NaN	CCL19,CCL4,CCL5,CXCL1,CXCL10,CXCL11,CXCL2,CXCL
Pathogene	8.94E+00	5.56E-01	NaN	CCL4,CCL5,CXCL10,CXCL11,CXCL9
Communic	8.00E+00	9.38E-02	NaN	CCL4,CCL5,CD8A,CXCL10,FCER1G,IGHG2,IGHG3,TNFRS
Primary Im	7.56E+00	1.40E-01	NaN	CD3D,CD8A,IGHG2,IGHG3,IGLL1/IGLL5,IL7R,PTPRC
Differential	4.91E+00	1.74E-01	2	CCL4,CCL5,CXCL1,LCN2
Hematopo	4.85E+00	1.02E-01	NaN	CD3D,CD8A,FCER1G,IGHG2,IGHG3
Systemic L	4.81E+00	3.93E-02	NaN	CD3D,FCER1G,FCGR2B,IGHG2,IGHG3,KLK1,PTPRC,RASD
Acute Phas	4.71E+00	4.44E-02	2.449	ALB,APOH,C3,CP,FGB,RASD1,SERPINA3,SERPING1
IL-17A Sig	4.69E+00	1.54E-01	NaN	CCL5,CXCL1,CXCL10,CXCL11
Compleme	4.07E+00	1.08E-01	1	C1QB,C1QC,C3,SERPING1
Role of IL-	3.81E+00	9.30E-02	NaN	CCL4,CXCL1,CXCL10,CXCL6
Differential	3.73E+00	1.67E-01	NaN	CCL4,CCL5,CXCL1
Role of Hy	3.67E+00	5.81E-02	2.236	CCL4,CCL5,CXCL10,ISG20,PYCARD
Natural Kill	3.58E+00	3.55E-02	2.449	FCER1G,HCST,KLRB1,KLRC4-KLRK1/KLRK1,RAC2,RASD
LXR/RXR A	2.99E+00	4.13E-02	-1.342	ALB,APOH,C3,LY96,LYZ
Th1 Pathw	2.99E+00	4.13E-02	NaN	CD3D,CD8A,HLA-DQA2,IL10RA,STAT4
Dendritic (	2.95E+00	3.26E-02	2	CD1C,FCER1G,FCGR2B,IGHG2,IGHG3,STAT4
Hepatic Fib	2.92E+00	3.23E-02	NaN	CCL5,CXCL9,EGF,IL10RA,LY96,TIMP1
B Cell Rece	2.92E+00	3.23E-02	NaN	FCGR2B,IGHG2,IGHG3,PTPRC,RAC2,RASD1
Phospholip	2.82E+00	2.63E-02	NaN	CD3D,FCER1G,FCGR2B,IGHG2,IGHG3,RAC2,RASD1
Phagosom	2.80E+00	3.76E-02	NaN	FCER1G,FCGR2B,IGHG2,IGHG3,RAC2
MSP-RON	2.79E+00	3.73E-02	1.342	CSF2RB,ELF3,KLK1,RASD1,SFN
STAT3 Path	2.77E+00	3.70E-02	NaN	CSF2RB,EGF,IL10RA,IL7R,RASD1
Inhibition (	2.72E+00	7.69E-02	NaN	MMP7,TFPI2,TIMP1
Allograft R	2.67E+00	4.65E-02	NaN	FCER1G,HLA-DQA2,IGHG2,IGHG3
Glucocortic	2.57E+00	1.95E-02	NaN	CCL5,CD3D,CSF2RB,EGF,G6PC,IL10RA,IL7R,RASD1,SLPI
Role of Pat	2.53E+00	3.25E-02	2	C1QB,C1QC,C3,CCL5,TNFSF13B
CCR5 Signa	2.52E+00	4.26E-02	NaN	CCL4,CCL5,CD3D,FCER1G
Neuroinfla	2.52E+00	2.33E-02	1.342	BIRC3,CCL5,CXCL10,GABBR1,GABRP,KLK1,PYCARD
Role of IL-	2.47E+00	1.43E-01	NaN	CXCL1,CXCL6
Autoimmu	2.44E+00	6.12E-02	NaN	FCER1G,IGHG2,IGHG3
T Cell Rece	2.34E+00	3.77E-02	NaN	CD3D,CD8A,PTPRC,RASD1
Th1 and Th	2.33E+00	2.92E-02	NaN	CD3D,CD8A,HLA-DQA2,IL10RA,STAT4
Erythropoi	2.31E+00	2.89E-02	1.342	BIRC3,CSF2RB,RAC2,RASD1,TNFSF13B
Role of IL-	2.29E+00	5.45E-02	NaN	CCL5,CXCL1,CXCL6
T Cell Exha	2.29E+00	2.86E-02	NaN	FCER1G,HLA-DQA2,IL10RA,RASD1,STAT4
NF-魏B Sig	2.25E+00	2.79E-02	1	EGF,FCER1G,RASD1,TNFRSF17,TNFSF13B
MSP-RON	2.24E+00	3.54E-02	-1	HLA-DQA2,KLK1,NFKBIZ,RASD1
Neuroprot	2.20E+00	3.45E-02	0	GZMA,GZMK,KLK1,SERPINA3
Role of Tis	2.20E+00	3.45E-02	NaN	CCN1,CXCL1,FGB,RASD1
PI3K/AKT S	2.20E+00	2.72E-02	NaN	CSF2RB,IL10RA,IL7R,RASD1,SFN
CD28 Signa	2.14E+00	3.31E-02	NaN	ARPC1B,CD3D,FCER1G,PTPRC
Clathrin-m	2.11E+00	2.59E-02	NaN	ALB,ARPC1B,EGF,LYZ,UBD
FXR/RXR A	2.08E+00	3.17E-02	NaN	ALB,APOH,C3,G6PC
ERK5 Signa	1.97E+00	4.17E-02	NaN	EGF,RASD1,SFN
T Helper C	1.95E+00	4.11E-02	NaN	FCER1G,IL10RA,STAT4
Glioma Inv	1.95E+00	4.11E-02	NaN	RAC2,RASD1,TIMP1
PI3K Signa	1.94E+00	2.90E-02	NaN	C3,FCGR2B,PTPRC,RASD1
Lipid Antig	1.94E+00	7.69E-02	NaN	CD1C,FCER1G
Role of MA	1.85E+00	3.75E-02	NaN	CCL5,CXCL10,RASD1
Chemokine	1.85E+00	3.75E-02	NaN	CCL4,CCL5,RASD1
Actin Nucle	1.83E+00	3.70E-02	NaN	ARPC1B,RAC2,RASD1
Role of Ma	1.83E+00	1.91E-02	NaN	CCL5,IGHG2,IGHG3,RASD1,SOST,TNFSF13B
PKC胃 Sign	1.77E+00	2.58E-02	NaN	CD3D,FCER1G,RAC2,RASD1
CTLA4 Sign	1.73E+00	3.37E-02	NaN	CD3D,CD8A,FCER1G

Crosstalk b	1.73E+00	3.37E-02	NaN	CD69,CSF2RB,KLRC4-KLRK1/KLRK1
Cytotoxic T	1.71E+00	5.88E-02	NaN	CD3D,FCER1G
Altered T C	1.71E+00	3.33E-02	NaN	FCER1G,TNFRSF17,TNFSF13B
OX40 Sign	1.71E+00	3.33E-02	NaN	CD3D,FCER1G,HLA-DQA2
B Cell Deve	1.66E+00	5.56E-02	NaN	IL7R,PTPRC
IL-17A Sig	1.66E+00	5.56E-02	NaN	LCN2,NFKBIZ
GABA Rece	1.65E+00	3.16E-02	NaN	GABBR1,GABRP,UBD
Bladder Ca	1.63E+00	3.09E-02	NaN	EGF,MMP7,RASD1
Antiprolife	1.60E+00	5.13E-02	NaN	CD3D,FCER1G
Antigen Pr	1.60E+00	5.13E-02	NaN	HLA-DQA2,NLRC5
Cdc42 Sigr	1.59E+00	2.27E-02	NaN	ARPC1B,CD3D,FCER1G,HLA-DQA2
SAPK/JNK	1.57E+00	2.94E-02	NaN	FCER1G,RAC2,RASD1
B Cell Activ	1.56E+00	4.88E-02	NaN	TNFRSF17,TNFSF13B
Role of NF	1.55E+00	2.21E-02	NaN	CD3D,FCER1G,FCGR2B,RASD1
IGF-1 Sign	1.55E+00	2.88E-02	NaN	CCN1,RASD1,SFN
Intrinsic Pr	1.54E+00	4.76E-02	NaN	FGB,KLK1
Telomerase	1.52E+00	2.80E-02	NaN	EGF,ELF3,RASD1
Systemic Lu	1.52E+00	1.82E-02	1.342	FCGR2B,ISG20,RAC2,RASD1,TNFSF13B
IL-17 Signa	1.51E+00	2.14E-02	2	CXCL1,LCN2,RASD1,TNFSF13B
Ephrin Rec	1.49E+00	2.12E-02	NaN	ARPC1B,EGF,RAC2,RASD1
iCOS-iCOS	1.48E+00	2.70E-02	NaN	CD3D,FCER1G,PTPRC
Breast Can	1.47E+00	1.36E-02	0.707	ACKR1,ADGRV1,EGF,GABBR1,GPR171,GPR183,RAC2,RA
Leukocyte	1.47E+00	2.07E-02	0	ARHGAP9,MMP7,RAC2,TIMP1
HER-2 Sigr	1.46E+00	2.06E-02	1	EGF,ELF3,FCER1G,RASD1
Fc Epsilon	1.42E+00	2.56E-02	NaN	FCER1G,RAC2,RASD1
IL-8 Signal	1.42E+00	2.00E-02	NaN	CXCL1,EGF,RAC2,RASD1
Airway Pat	1.41E+00	2.54E-02	NaN	CXCL1,LCN2,TNFSF13B
MSP-RON	1.28E+00	3.45E-02	NaN	CSF2RB,KLK1
IL-12 Signa	1.28E+00	2.26E-02	NaN	ALB,LYZ,STAT4
Th2 Pathwa	1.26E+00	2.21E-02	NaN	CD3D,HLA-DQA2,STAT4
SPINK1 Pa	1.26E+00	3.33E-02	NaN	CPA3,KLK1
Actin Cyto	1.25E+00	1.76E-02	NaN	ARPC1B,EGF,RAC2,RASD1
Autophagy	1.25E+00	3.28E-02	NaN	CTSS,CTSV
Ovarian Ca	1.24E+00	2.16E-02	NaN	EGF,MMP7,RASD1
Hereditary	1.23E+00	2.14E-02	NaN	RASD1,SFN,UBD
Systemic Lu	1.22E+00	1.50E-02	1	CD3D,FCER1G,HLA-DQA2,RAC2,RASD1
Role of PI3	1.21E+00	3.12E-02	NaN	CCL5,PLAC8
PXR/RXR A	1.20E+00	3.08E-02	NaN	CYP3A7,G6PC
IL-17A Sig	1.20E+00	3.08E-02	NaN	CXCL1,CXCL6
Calcium-in	1.19E+00	3.03E-02	NaN	CD3D,FCER1G
Nur77 Sigr	1.16E+00	2.94E-02	NaN	CD3D,FCER1G
Role of JAk	1.15E+00	2.90E-02	NaN	IL7R,RASD1
SPINK1 Ge	1.15E+00	2.90E-02	NaN	GZMA,RASD1
Epithelial A	1.14E+00	1.97E-02	NaN	ARPC1B,EGF,RASD1
IL-10 Signa	1.14E+00	2.86E-02	NaN	FCGR2B,IL10RA
Agrin Inter	1.14E+00	2.86E-02	NaN	RAC2,RASD1
GM-CSF Si	1.14E+00	2.86E-02	NaN	CSF2RB,RASD1
Colorectal	1.12E+00	1.58E-02	NaN	EGF,MMP7,RAC2,RASD1
Caveolar-r	1.11E+00	2.74E-02	NaN	ALB,EGF
Non-Small	1.11E+00	2.74E-02	NaN	EGF,RASD1
Regulation	1.10E+00	2.70E-02	NaN	EGF,RASD1
TREM1 Sig	1.09E+00	2.67E-02	NaN	FCGR2B,NLRC5
Fc纬RIIB Si	1.09E+00	2.67E-02	NaN	FCGR2B,RASD1
Role of MA	1.09E+00	2.67E-02	NaN	CCL5,CXCL10
Toll-like Re	1.08E+00	2.63E-02	NaN	LY96,UBD
Macropinc	1.08E+00	2.63E-02	NaN	EGF,RASD1
CREB Signa	1.08E+00	1.17E-02	1.134	ACKR1,ADGRV1,EGF,GABBR1,GPR171,GPR183,RASD1
HMGB1 Si	1.06E+00	1.82E-02	NaN	RAC2,RASD1,TNFSF13B

Glioblastor	1.06E+00	1.82E-02	NaN	EGF,RAC2,RASD1
VDR/RXR /	1.06E+00	2.56E-02	NaN	CCL5,CXCL10
IL-3 Signal	1.05E+00	2.53E-02	NaN	CSF2RB,RASD1
FLT3 Signa	1.04E+00	2.50E-02	NaN	RASD1,STAT4
Renal Cell	1.04E+00	2.50E-02	NaN	RASD1,UBD
JAK/Stat Si	1.04E+00	2.50E-02	NaN	RASD1,STAT4
Extrinsic Pr	1.02E+00	6.25E-02	NaN	FGB
Tec Kinase	1.01E+00	1.73E-02	NaN	FCER1G,RAC2,STAT4
Wnt/尾-ca	1.01E+00	1.73E-02	NaN	MMP7,SOX9,UBD
Tumor Mic	1.00E+00	1.70E-02	NaN	EGF,MMP7,RASD1
FAT10 Sigr	9.67E-01	5.56E-02	NaN	UBD
Acute Mye	9.63E-01	2.25E-02	NaN	CSF2RB,RASD1
Regulation	9.55E-01	2.22E-02	NaN	CD3D,RASD1
Granzyme	9.47E-01	5.26E-02	NaN	GZMA
DNA dama	9.47E-01	5.26E-02	NaN	SFN
Th17 Activ	9.47E-01	2.20E-02	NaN	FCER1G,STAT4
Regulation	9.36E-01	1.60E-02	NaN	EGF,RASD1,TNFSF13B
Productior	9.28E-01	1.59E-02	NaN	ALB,LYZ,RAC2
RhoGDI Sig	9.28E-01	1.59E-02	NaN	ARHGAP9,ARPC1B,RAC2
Fc纬 Recep	9.24E-01	2.13E-02	NaN	ARPC1B,RAC2
ErbB Signa	9.24E-01	2.13E-02	NaN	EGF,RASD1
The Visual	9.24E-01	5.00E-02	NaN	RDH12
Inflammaso	9.24E-01	5.00E-02	NaN	PYCARD
VEGF Signa	8.89E-01	2.02E-02	NaN	RASD1,SFN
Apoptosis	8.79E-01	2.00E-02	NaN	BIRC3,RASD1
Virus Entry	8.66E-01	1.96E-02	NaN	RAC2,RASD1
ERK/MAPK	8.66E-01	1.49E-02	NaN	ELF3,RAC2,RASD1
Regulation	8.60E-01	1.94E-02	NaN	ARPC1B,RAC2
Sumoylatic	8.60E-01	1.94E-02	NaN	ISG20,RAC2
HIF1伪 Sig	8.54E-01	1.46E-02	NaN	EGF,MMP7,RASD1
FAK Signal	8.54E-01	1.92E-02	NaN	EGF,RASD1
Neuregulir	8.48E-01	1.90E-02	NaN	EGF,RASD1
Thrombin S	8.39E-01	1.44E-02	NaN	EGF,RAC2,RASD1
Bupropion	8.36E-01	4.00E-02	NaN	CYP3A7
Integrin Sig	8.18E-01	1.41E-02	NaN	ARPC1B,RAC2,RASD1
Glioma Sig	8.15E-01	1.82E-02	NaN	EGF,RASD1
Type I Diak	8.10E-01	1.80E-02	NaN	CD3D,FCER1G
Endocanna	7.85E-01	1.74E-02	NaN	RAC2,RASD1
fMLP Signa	7.80E-01	1.72E-02	NaN	ARPC1B,RASD1
Renin-Ang	7.67E-01	1.69E-02	NaN	CCL5,RASD1
Cholecystc	7.62E-01	1.68E-02	NaN	RAC2,RASD1
TNFR2 Sigr	7.62E-01	3.33E-02	NaN	BIRC3
HGF Signa	7.57E-01	1.67E-02	NaN	ELF3,RASD1
Rac Signali	7.50E-01	1.65E-02	NaN	ARPC1B,RASD1
Acetone D	7.50E-01	3.23E-02	NaN	CYP3A7
Inhibition c	7.45E-01	1.64E-02	NaN	SFN,TNFSF13B
RhoA Signa	7.40E-01	1.63E-02	NaN	ARHGAP9,ARPC1B
G伪i Signa	7.28E-01	1.60E-02	NaN	GABBR1,RASD1
GP6 Signal	7.28E-01	1.60E-02	NaN	FCER1G,FGB
14-3-3-m	7.19E-01	1.57E-02	NaN	RASD1,SFN
Atheroscle	7.19E-01	1.57E-02	NaN	ALB,LYZ
MIF-media	7.12E-01	2.94E-02	NaN	LY96
Retinoate I	7.12E-01	2.94E-02	NaN	RDH12
p70S6K Sig	7.08E-01	1.55E-02	NaN	RASD1,SFN
Coagulatic	7.01E-01	2.86E-02	NaN	FGB
TWEAK Sig	7.01E-01	2.86E-02	NaN	BIRC3
Axonal Gui	6.93E-01	1.01E-02	NaN	ARPC1B,EGF,MMP7,RAC2,RASD1
Cardiac Hy	6.86E-01	1.01E-02	NaN	CSF2RB,IL10RA,IL7R,RASD1,TNFSF13B

PTEN Signa	6.74E-01	1.47E-02	NaN		RAC2,RASD1
Iron home	6.70E-01	1.46E-02	NaN		CP,EGF
Hepatic Fib	6.64E-01	1.06E-02		2	CCL5,RAC2,RASD1,TIMP1
April Media	6.50E-01	2.50E-02	NaN		TNFRSF17
MIF Regula	6.31E-01	2.38E-02	NaN		LY96
Estrogen B	6.31E-01	2.38E-02	NaN		CYP3A7
Oncostatin	6.22E-01	2.33E-02	NaN		RASD1
Retinol Bio	6.14E-01	2.27E-02	NaN		RDH12
IL-23 Signa	6.14E-01	2.27E-02	NaN		STAT4
Coronaviru	6.13E-01	1.33E-02	NaN		CCL5,PYCARD
Phagosom	6.07E-01	1.32E-02	NaN		CTSS,CTSV
iNOS Signa	6.06E-01	2.22E-02	NaN		LY96
FAT10 Can	5.97E-01	2.17E-02	NaN		UBD
nNOS Sign	5.88E-01	2.13E-02	NaN		RASD1
Necroptos	5.83E-01	1.27E-02	NaN		BIRC3,PYCARD
Graft-versu	5.82E-01	2.08E-02	NaN		FCER1G
Cell Cycle:	5.73E-01	2.04E-02	NaN		SFN
Melanoma	5.65E-01	2.00E-02	NaN		RASD1
TNFR1 Sig	5.65E-01	2.00E-02	NaN		BIRC3
UVC-Induc	5.59E-01	1.96E-02	NaN		RASD1
CXCR4 Sig	5.47E-01	1.20E-02	NaN		RAC2,RASD1
Lymphoto	5.45E-01	1.89E-02	NaN		CXCL1
Germ Cell-	5.32E-01	1.17E-02	NaN		RAC2,RASD1
EGF Signal	5.32E-01	1.82E-02	NaN		EGF
CNTF Signa	5.19E-01	1.75E-02	NaN		RASD1
Nicotine D	5.19E-01	1.75E-02	NaN		CYP3A7
Cancer Dru	5.11E-01	1.72E-02	NaN		RASD1
Semaphori	5.00E-01	1.67E-02	NaN		RAC2
Maturity O	5.00E-01	1.67E-02	NaN		APOH
Endometria	5.00E-01	1.67E-02	NaN		RASD1
Melatonin	5.00E-01	1.67E-02	NaN		CYP3A7
Induction c	4.93E-01	1.64E-02	NaN		BIRC3
IL-2 Signal	4.93E-01	1.64E-02	NaN		RASD1
Hepatic Ch	4.84E-01	1.08E-02	NaN		LY96,TNFSF13B
Thrombop	4.83E-01	1.59E-02	NaN		RASD1
NRF2-mec	4.75E-01	1.06E-02	NaN		MAFF,RASD1
ErbB2-ErbI	4.71E-01	1.54E-02	NaN		RASD1
Netrin Sign	4.71E-01	1.54E-02	NaN		RAC2
Nicotine D	4.71E-01	1.54E-02	NaN		CYP3A7
Superpathy	4.71E-01	1.54E-02	NaN		CYP3A7
Pyridoxal 5	4.66E-01	1.52E-02	NaN		G6PC
Regulation	4.66E-01	1.04E-02	NaN		EGF,RASD1
Estrogen R	4.66E-01	9.15E-03	NaN		EGF,MMP7,RASD1
ErbB4 Sign	4.61E-01	1.49E-02	NaN		RASD1
RAR Activa	4.60E-01	1.03E-02	NaN		CSF2RB,RDH12
Remodelin	4.56E-01	1.47E-02	NaN		ARPC1B
Adrenome	4.51E-01	1.02E-02	NaN		C3,RASD1
Gap Juncti	4.49E-01	1.01E-02	NaN		EGF,RASD1
Ephrin B Si	4.35E-01	1.39E-02	NaN		RAC2
IL-15 Signa	4.23E-01	1.33E-02	NaN		RASD1
Angiopoie	4.23E-01	1.33E-02	NaN		RASD1
Estrogen-D	4.23E-01	1.33E-02	NaN		RASD1
NF-魏B Ac	4.18E-01	1.32E-02	NaN		RASD1
GDNF Fam	4.18E-01	1.32E-02	NaN		RASD1
Neurotrop	4.18E-01	1.32E-02	NaN		RASD1
mTOR Sign	4.17E-01	9.52E-03	NaN		RAC2,RASD1
Antiprolife	4.13E-01	1.30E-02	NaN		RASD1
IL-7 Signal	4.09E-01	1.28E-02	NaN		IL7R

Thyroid Ca	4.05E-01	1.27E-02	NaN	RASD1
BEX2 Signa	4.05E-01	1.27E-02	NaN	KLK1
Role of Os	3.97E-01	9.17E-03	NaN	BIRC3,SOST
Prolactin S	3.96E-01	1.23E-02	NaN	RASD1
Osteoarthr	3.93E-01	9.09E-03	NaN	ELF3,SOX9
PEDF Signa	3.91E-01	1.22E-02	NaN	RASD1
LPS-stimul	3.88E-01	1.20E-02	NaN	RASD1
TR/RXR Ac	3.84E-01	1.19E-02	NaN	G6PC
VEGF Fami	3.84E-01	1.19E-02	NaN	RASD1
LPS/IL-1 M	3.81E-01	8.89E-03	NaN	CYP3A7,LY96
HIPPO sigr	3.80E-01	1.18E-02	NaN	SFN
IL-4 Signal	3.80E-01	1.18E-02	NaN	RASD1
BMP signa	3.80E-01	1.18E-02	NaN	RASD1
PDGF Sign	3.77E-01	1.16E-02	NaN	RASD1
Ceramide S	3.69E-01	1.14E-02	NaN	RASD1
RANK Sign	3.66E-01	1.12E-02	NaN	BIRC3
Prostate Ca	3.58E-01	1.10E-02	NaN	RASD1
Death Rece	3.55E-01	1.09E-02	NaN	BIRC3
Huntingtor	3.51E-01	8.37E-03	NaN	EGF,UBD
Cardiac Hy	3.48E-01	8.33E-03	NaN	RAC2,RASD1
Melanocyt	3.48E-01	1.06E-02	NaN	RASD1
伪-Adrene	3.44E-01	1.05E-02	NaN	RASD1
TGF-尾 Sig	3.41E-01	1.04E-02	NaN	RASD1
Amyotroph	3.37E-01	1.03E-02	NaN	BIRC3
p53 Signali	3.34E-01	1.02E-02	NaN	SFN
Salvage Pa	3.34E-01	1.02E-02	NaN	G6PC
UVA-Induc	3.34E-01	1.02E-02	NaN	RASD1
Opioid Sig	3.34E-01	8.10E-03	NaN	RAC2,RASD1
Molecular	3.33E-01	7.50E-03	NaN	BIRC3,RAC2,RASD1
Signaling b	3.22E-01	7.91E-03	NaN	ARPC1B,RAC2
Chronic My	3.19E-01	9.71E-03	NaN	RASD1
Mouse Em	3.19E-01	9.71E-03	NaN	RASD1
PPAR Signa	3.13E-01	9.52E-03	NaN	RASD1
PAK Signal	3.10E-01	9.43E-03	NaN	RASD1
PD-1, PD-	3.10E-01	9.43E-03	NaN	HLA-DQA2
CDK5 Sign	3.05E-01	9.26E-03	NaN	RASD1
Paxillin Sig	3.05E-01	9.26E-03	NaN	RASD1
Pancreatic	3.02E-01	9.17E-03	NaN	EGF
Antioxidan	3.02E-01	9.17E-03	NaN	CSF2RB
Role of MA	2.99E-01	9.09E-03	NaN	RASD1
NGF Signa	2.88E-01	8.77E-03	NaN	RASD1
Protein Ub	2.87E-01	7.33E-03	NaN	BIRC3,UBD
G-Protein	2.86E-01	7.30E-03	NaN	GABBR1,RASD1
Apelin End	2.86E-01	8.70E-03	NaN	RASD1
Senescenc	2.84E-01	7.27E-03	NaN	ELF3,RASD1
Sphingosir	2.81E-01	8.55E-03	NaN	RAC2
Role of PKI	2.78E-01	8.47E-03	NaN	PYCARD
Role of NA	2.76E-01	8.40E-03	NaN	RASD1
Reelin Sigr	2.68E-01	8.20E-03	NaN	ARPC1B
G Beta Gar	2.68E-01	8.20E-03	NaN	RASD1
Xenobiotic	2.65E-01	6.97E-03	NaN	CYP3A7,RASD1
CCR3 Signa	2.64E-01	8.06E-03	NaN	RASD1
IL-6 Signal	2.59E-01	7.94E-03	NaN	RASD1
Ferroptosis	2.59E-01	7.94E-03	NaN	RASD1
P2Y Purige	2.57E-01	7.87E-03	NaN	RASD1
Synaptic Lo	2.53E-01	7.75E-03	NaN	RASD1
White Adip	2.53E-01	7.75E-03	NaN	FCER1G
G伪12/13 S	2.48E-01	7.63E-03	NaN	RASD1

Adipogene	2.42E-01	7.46E-03	NaN	SOX9
Insulin Rec	2.32E-01	7.19E-03	NaN	RASD1
Semaphori	2.32E-01	7.19E-03	NaN	VCAN
Synaptoge	2.31E-01	6.41E-03	NaN	ARPC1B,RASD1
D-myo-inc	2.26E-01	7.04E-03	NaN	PTPRC
D-myo-inc	2.26E-01	7.04E-03	NaN	PTPRC
Xenobiotic	2.24E-01	6.99E-03	NaN	RASD1
3-phosphc	2.01E-01	6.41E-03	NaN	PTPRC
D-myo-inc	2.00E-01	6.37E-03	NaN	PTPRC
G伪q Signa	2.00E-01	6.37E-03	NaN	RAC2
Synaptic Lc	0.00E+00	5.29E-03	NaN	RASD1
PPAR伪/RX	0.00E+00	5.24E-03	NaN	RASD1
Endothelin	0.00E+00	5.32E-03	NaN	RASD1
GNRH Sigr	0.00E+00	5.78E-03	NaN	RASD1
ILK Signalir	0.00E+00	5.26E-03	NaN	RAC2
EIF2 Signal	0.00E+00	4.46E-03	NaN	RASD1
Protein Kin	0.00E+00	5.00E-03	NaN	PTPRC,SFN
Regulation	0.00E+00	6.02E-03	NaN	RASD1
Role of NF.	0.00E+00	4.67E-03	NaN	RASD1
Sertoli Cell	0.00E+00	5.15E-03	NaN	RASD1
Superpath\	0.00E+00	5.03E-03	NaN	PTPRC
3-phosphc	0.00E+00	6.02E-03	NaN	PTPRC
cAMP-mec	0.00E+00	4.37E-03	NaN	GABBR1
HOTAIR Re	0.00E+00	6.25E-03	NaN	MMP7
Xenobiotic	0.00E+00	5.29E-03	NaN	CYP3A7
Xenobiotic	0.00E+00	5.21E-03	NaN	CYP3A7
Insulin Sec	0.00E+00	4.10E-03	NaN	STAT4

6,CXCL9,MMP7,SELL,XCL1,XCL2
6,CXCL9,MMP7,SELL,XCL1,XCL2

SF17,TNFSF13B

D1,TNFSF13B

1,STAT4

ASD1
