# Supplementary material for: The Potential Diagnostic Value of Immune-Related Genes in Interstitial Fibrosis and Tubular Atrophy after Kidney Transplantation
Source: J Immunol Res. 2022 Jun 17;2022:7212852. doi: 10.1155/2022/7212852 (PMC9232312; doi:10.1155/2022/7212852)
Supplement: Supplementary Materials — Supplementary Figure 1: GSEA enrichment analysis of the IF/TA group. Supplementary Figure 2: correlation analysis between ANGPTL3 and differentially expressed immune infiltrating cells. Supplementary Figure 3: correlation analysis between APOH and differentially expressed immune infiltrating cells. Supplementary Figure 4: correlation analysis between EGF and differentially expressed immune infiltrating cells. Supplementary Figure 5: correlation analysis between FCGR2B and differentially expressed immune infiltrating cells. Supplementary Figure 6: correlation analysis between HLA-DQA2 and differentially expressed immune infiltrating cells. Supplementary Figure 7: correlation analysis between LTF and differentially expressed immune infiltrating cells. Supplementary Figure 8: IPA analysis shows the interaction network of diagnostic genes: EGF and LTF (8A), ANGPTL3 (8B), FCGR2B and APOH (8C), and HLA-DQA2 (8D). Merged the above four independent networks to comprehensively analyze the interaction of diagnostic genes (8E). Supplementary Table 1: immune-related genes. Supplementary Table 2: KEGG pathway in normal group. Supplementary Table 3: pathway of ANGPTL3 gene. Supplementary Table 4: pathway of APOH gene. Supplementary Table 5: pathway of EGF gene. Supplementary Table 6: ingenuity canonical pathways. Supplementary Table 7: category. [file 7212852.f1.zip › 7212852.f1/supplementary table13.pdf]

Category	p-value	Molecules
Cellular Mc	1.39E-32-2	ACKR1,AGR2,ALB,ANGPTL3,APOH,ARL4C,ARPC1B,C3,CCL19,CCL4,CCL5,CCN
Immune Cc	1.39E-32-1	ACKR1,ALB,APOH,C3,CCL19,CCL4,CCL5,CCN1,CD1C,CD2,CD69,CD8A,CORO1
Inflammato	7.75E-30-1	ACKR1,AGR2,ALB,APOH,ARHGAP9,BIRC3,C1QB,C1QC,C3,CCL19,CCL4,CCL5,C
Organisma	7.75E-30-2	ACKR1,ADGRV1,AFM,AGR2,AGR3,ALB,APOH,ARHGAP9,ARL4C,ARPC1B,BIRC3
Gastrointe	9.41E-26-1	ACKR1,AGR2,ALB,APOH,BIRC3,C1orf162,C3,CCL19,CCL4,CCL5,CCN1,CD3D,C
Hematolog	5.13E-25-1	ACKR1,AGR2,ALB,APOH,BIRC3,C1QC,C3,CCL19,CCL4,CCL5,CCN1,CD1C,CD2,C
Tissue Mor	5.13E-25-1	AGR2,BIRC3,C3,CCL19,CCL4,CCL5,CD3D,CD69,CD8A,CORO1A,CP,CSF2RB,CT
Immunolog	5.57E-24-2	ADGRV1,ALB,APOH,ARL4C,ARPC1B,BIRC3,C1orf162,C1QB,C1QC,C3,CCL19,C
Cellular De	2.68E-23-1	ALB,APOH,BIRC3,C1QC,C3,CCL19,CCL4,CCL5,CCN1,CD2,CD3D,CD69,CD8A,C
Cellular Gr	2.68E-23-1	ALB,APOH,BIRC3,C1QC,C3,CCL19,CCL4,CCL5,CCN1,CD2,CD3D,CD52,CD69,C
Lymphoid	2.68E-23-1	AGR2,ALB,APOH,BIRC3,C1QC,C3,CCL19,CCL4,CCL5,CCN1,CD2,CD3D,CD69,C
Endocrine	1.63E-22-2	ADGRV1,AFM,AGR2,AGR3,ALB,APOH,ARL4C,C16orf54,C1orf162,C3,CCL19,C
Metabolic	1.63E-22-1	AFM,ALB,C1orf162,C3,CCL19,CCL4,CCL5,CCN1,CD3D,CD52,CP,CSF2RB,CST7,
Cell-To-Ce	1.84E-19-1	ACKR1,ALB,ANGPTL3,APOH,BIRC3,C3,CCL19,CCL4,CCL5,CCN1,CD1C,CD2,CD
Inflammato	2.32E-18-1	ACKR1,AGR2,ALB,APOH,ARL4C,BIRC3,C1QB,C3,CCL19,CCL4,CCL5,CCN1,CD1
Cellular Fu	2.87E-18-7	ACKR1,ALB,APOH,BIRC3,C3,CCL19,CCL4,CCL5,CCN1,CD2,CD3D,CD52,CD69,C
Connective	1.21E-17-6	ALB,APOH,C1QB,C3,CCL19,CCL4,CCL5,CD1C,CD3D,CD52,CD69,CSF2RB,CTSS
Skeletal an	1.21E-17-5	AFM,ALB,APOH,C1QB,C3,CCL19,CCL4,CCL5,CD1C,CD3D,CD52,CD69,CP,CSF2
Cell Death	1.64E-17-2	AGR2,ALB,BIRC3,C3,CCL19,CCL4,CCL5,CCN1,CD2,CD52,CD53,CD69,CD8A,CC
Hematopo	8.97E-16-1	C1QC,CCL19,CCL5,CCN1,CD2,CD3D,CD69,CD8A,CSF2RB,CTSV,CXCL1,CXCL1
Tissue Dev	8.97E-16-1	C1QC,C3,CCL19,CCL5,CCN1,CD2,CD3D,CD69,CD8A,CSF2RB,CTSV,CXCL1,CXC
Infectious I	2.48E-15-2	ALB,APOH,ARL4C,ARPC1B,BIRC3,C3,CCL4,CCL5,CD3D,CD69,CD8A,CSF2RB,C
Humoral Ir	2.68E-15-1	C1QB,C1QC,C3,CCL19,CD69,CSF2RB,CTSS,CXCL10,DOCK2,FCER1G,FCGR2B,G
Cellular Co	6.2E-15-1.	ALB,APOH,ARHGAP9,C3,CCL4,CCL5,CD2,CD53,CD69,CORO1A,CTSS,CXCL1,C
Digestive S	1.65E-14-1	ACKR1,ALB,BIRC3,C3,CCL4,CCL5,CCN1,CSF2RB,CXCL1,CXCL10,CXCL11,CXCL
Hepatic Sy	1.65E-14-1	ACKR1,ALB,BIRC3,C3,CCL4,CCL5,CCN1,CSF2RB,CXCL1,CXCL10,CXCL11,CXCL
Organ Dev	1.65E-14-1	ACKR1,ALB,BIRC3,C3,CCL19,CCL4,CCL5,CCN1,CD2,CD3D,CD69,CD8A,CSF2RE
Antimicrob	1.71E-14-9	BIRC3,CCL19,CCL4,CCL5,CD8A,CXCL10,FCER1G,GZMA,IGKV3-20,IL10RA,ISG2
Hypersensi	2.97E-14-1	ALB,C3,CCL19,CCL4,CCL5,CD2,CXCL10,CXCL11,CXCL2,CXCL9,LTF,RAC2,SPON
Hepatic Sy	5.95E-14-1	ACKR1,ALB,BIRC3,C3,CCL4,CCL5,CCN1,CSF2RB,CXCL1,CXCL10,CXCL11,CXCL
Ophthalmi	7.86E-14-7	C3,CCL19,CCL4,CCL5,CD69,CXCL10,CXCL6,EGF,KLRB1,STAT4,TIMP1,TNFRSF1
Cell Signali	4.93E-13-1	ACKR1,ALB,C3,CCL19,CCL4,CCL5,CD2,CD8A,CSF2RB,CXCL1,CXCL10,CXCL11,C
Molecular	4.93E-13-5	ACKR1,ALB,C3,CCL19,CCL4,CCL5,CD2,CD8A,CP,CXCL1,CXCL10,CXCL11,CXCL
Vitamin an	4.93E-13-5	ACKR1,ALB,C3,CCL19,CCL4,CCL5,CD2,CD8A,CXCL1,CXCL10,CXCL11,CXCL2,C
Cell-media	5.73E-12-1	ACKR1,C3,CCL19,CCL4,CCL5,CCN1,CD2,CD3D,CD69,CD8A,CORO1A,CTSS,CT
Hematolog	7.12E-12-1	ACKR1,ADGRV1,ALB,ARHGAP9,ARPC1B,BIRC3,C3,CCL19,CCL4,CCL5,CCN1,CD
Respirator	8.21E-12-7	ACKR1,ALB,APOH,ARL4C,C3,CCL4,CCL5,CD2,CD69,CD8A,CSF2RB,CTSS,CXCL
Embryonic	1.95E-11-2	CCL19,CCL5,CCN1,CD2,CD3D,CD69,CD8A,CTSV,CXCL1,CXCL10,DOCK2,ELF3,
Organisma	1.95E-11-1	AGR2,ALB,ANGPTL3,C3,CCL19,CCL5,CCN1,CD2,CD3D,CD69,CD8A,CP,CSF2RE
Dermatolo	2.66E-10-6	ARPC1B,C3,CCL4,CCL5,CD2,CSF2RB,CSTA,CTSS,CTSV,CXCL1,CXCL10,CXCL11
Cancer	2.82E-10-2	ACKR1,ADGRV1,AFM,AGR2,AGR3,ALB,APOH,ARHGAP9,ARL4C,BIRC3,C16orf5
Cardiovasc	3.03E-10-1	ALB,APOH,ARL4C,C3,CCL5,CD52,CD53,CSF2RB,CTSS,CXCL10,CYTIP,EVI2A,EV
Hereditary	7.76E-10-8	ALB,C1QB,C1QC,C3,CD3D,CD8A,CORO1A,CP,DOCK2,G6PC,IGKC,IGLL1/IGLL5
Organisma	2.05E-09-8	ADGRV1,AGR2,APOH,BIRC3,C3,CCN1,CD2,CD8A,CPA3,CSF2RB,CTSV,CXCL10
Skeletal an	1.02E-08-1	ACKR1,CCL5,CCN1,CTSV,CXCL1,CXCL11,CXCL2,EGF,MMP7,SERPINA3,TFPI2,T
Protein Syr	6.88E-08-1	ALB,C3,CCN1,CD69,CP,CSF2RB,CTSS,CXCL10,FCER1G,FCGR2B,IGKC,IGLL1/IGI
Neurologic	7.93E-08-8	AFM,ALB,C3,CCL4,CCL5,CCN1,CD52,CD8A,CORO1A,CP,CSF2RB,CTSS,CXCL1,
Reproduct	2.76E-07-2	ACKR1,ADGRV1,AFM,AGR2,AGR3,ALB,APOH,ARL4C,C16orf54,C3,CCL19,CCL
Cell Morpl	5.7E-07-1.	C3,CCL19,CCL4,CCL5,CD8A,CP,CPA3,CTSS,CXCL10,CXCL11,CXCL9,CYTIP,DO
Cardiovasc	6.65E-07-3	ACKR1,ANGPTL3,C3,CCL4,CCL5,CCN1,CSF2RB,CXCL1,CXCL10,CXCL11,CXCL2
Free Radic	9.51E-07-2	ALB,C3,CCL4,CCL5,CCN1,CD52,CXCL2,CXCL9,DOCK2,EGF,FCGR2B,GZMA,GZ
Developm	9.8E-07-2.	ALB,C1QB,C1QC,C3,CD3D,CP,CYTIP,DOCK2,G6PC,IGKC,IGLL1/IGLL5,IL7R,LTF,
Organ Mo	9.94E-07-1	C3,CD8A,CSF2RB,DOCK2,FCER1G,FCGR2B,HCST,IGKC,IL7R,LCN2,NFKBIZ,NLR
Renal and	2.07E-06-1	ALB,C3,CP,CTSS,EGF,FGB,LCN2,LYZ,SLC34A2,TIMP1,UBD
Tumor Mo	2.44E-06-2	CXCL1,CXCL2,CXCL6
Renal and	3.4E-06-3.	CTSV,CXCL10,CXCL2,CXCL9

Carbohydr	3.61E-06-1	APOH,CCL19,CCL5,CSF2RB,CXCL1,CXCL10,CXCL9,EGF,GABBR1,LTF,LY96,PRN
Small Mole	5.46E-06-8	C3,CCL19,CCL4,CCL5,CXCL10,CXCL11,CXCL9,EGF,FCER1G,HCLS1,LTF,PRNP,P
Drug Meta	7.41E-06-7	CCL5,CXCL10,EGF,LTF,SFN
Nucleic Ac	8.46E-06-8	CXCL10,CXCL11,CXCL9
Cellular As	1.01E-05-1	CCL5,CXCL10,CXCL11,EGF,RASD1,TNFRSF17
Hair and Sl	1.01E-05-2	CCL5,CXCL1,CXCL10,CXCL11,EGF,LTF,SELL,TIMP1
Post-Trans	1.17E-05-1	CCL4,CCL5,CD2,CD8A,EGF,FCER1G,PTPRC,SELL,SLC12A3

1,CD2,CD69,CD8A,CORO1A,CSF2RB,CTSS,CTSV,CXCL1,CXCL10,CXCL11,CXCL2,CXCL6,CXCL9,CYTIP,
LA,CSF2RB,CTSS,CTSV,CXCL1,CXCL10,CXCL11,CXCL2,CXCL6,CXCL9,CYTIP,DOCK2,EGF,FCER1G,FCGF
CN1,CD1C,CD2,CD3D,CD53,CD69,CD8A,CORO1A,CPA3,CSF2RB,CTSS,CTSV,CXCL1,CXCL10,CXCL11
3,C16orf54,C1orf162,C1QB,C1QC,C3,CCL19,CCL4,CCL5,CCN1,CD1C,CD2,CD3D,CD52,CD53,CD69,CD
D52,CD53,CD69,CD8A,CORO1A,CP,CPA3,CSF2RB,CTSS,CTSV,CXCL1,CXCL10,CXCL11,CXCL2,CXCL6,
CD3D,CD52,CD69,CD8A,CORO1A,CSF2RB,CTSS,CTSV,CXCL1,CXCL10,CXCL11,CXCL2,CXCL6,CXCL9,
SS,CTSV,CXCL1,CXCL10,CXCL2,CXCL6,CYTIP,DOCK2,EGF,FCER1G,FCGR2B,GPR183,HCLS1,HCST,IGK,
CL4,CCL5,CCN1,CD1C,CD2,CD3D,CD52,CD53,CD69,CD8A,CORO1A,CP,CPA3,CSF2RB,CTSS,CTSV,CX
ORO1A,CSF2RB,CTSV,CXCL1,CXCL10,CXCL11,CXCL2,CYTIP,DOCK2,ELF3,EVI2B,FCER1G,FCGR2B,GPF
D8A,CORO1A,CSF2RB,CTSV,CXCL1,CXCL10,CXCL11,CXCL2,CYTIP,DOCK2,EGF,ELF3,EVI2B,FCER1G,F
D8A,CORO1A,CSF2RB,CTSS,CTSV,CXCL1,CXCL10,CXCL11,CXCL2,CXCL6,CXCL9,CYTIP,DOCK2,EGF,E
L4,CCL5,CCN1,CD3D,CD52,CP,CSF2RB,CTSS,CTSV,CXCL1,CXCL10,CXCL11,CXCL9,EGF,ELF3,EVI2A,F
CSTA,CTSS,CTSV,CXCL1,CXCL10,CXCL9,DOCK2,EVI2A,FCER1G,FCGR2B,FXYD5,G6PC,GABBR1,GABR
52,CD69,CD8A,CORO1A,CP,CSF2RB,CTSS,CTSV,CXCL1,CXCL10,CXCL11,CXCL2,CXCL6,CXCL9,CYTIP,
C,CD2,CD3D,CD52,CD53,CD69,CD8A,CORO1A,CPA3,CSF2RB,CTSS,CTSV,CXCL1,CXCL10,CXCL11,CX
D8A,CORO1A,CP,CSF2RB,CTSS,CTSV,CXCL1,CXCL10,CXCL2,CXCL6,DOCK2,EGF,ELF3,FCER1G,FCGR
,CXCL1,CXCL10,CXCL11,CXCL2,CXCL6,CXCL9,ELF3,EVI2A,FCER1G,FCGR2B,FGB,GABRP,GZMA,HCLS1
RB,CTSS,CXCL1,CXCL10,CXCL11,CXCL2,CXCL6,CXCL9,ELF3,ETNPPL,FCER1G,FCGR2B,FGB,G6PC,GAB
RO1A,CSF2RB,CSTA,CTSS,CTSV,CXCL1,CXCL10,CXCL11,CXCL2,CXCL9,DOCK2,EGF,ELF3,EVI2A,FCER
0,CXCL11,CXCL9,DOCK2,ELF3,EVI2B,FCER1G,FCGR2B,GPR183,HCLS1,IGKC,IGLL1/IGLL5,IL10RA,IL7R,
L10,CXCL11,CXCL2,DOCK2,EGF,ELF3,EVI2B,FCER1G,FCGR2B,GPR183,HCLS1,IGKC,IGLL1/IGLL5,IL10F
TSS,CTSV,CXCL1,CXCL10,CXCL11,CXCL2,CXCL6,CXCL9,EGF,FCER1G,FCGR2B,GABBR1,GABRP,GZMA,
PR183,HCLS1,IGHG2,IGHG3,IGHV1-69,IGHV3-23,IGK,IGKC,IGKV3-20,IGLL1/IGLL5,IGLV1-44,IGLV2-
XCL2,DOCK2,EGF,FCER1G,FCGR2B,FGB,G6PC,GPR183,GZMA,HCLS1,HCST,IGHG3,JCHAIN,KLRB1,KL
2,CXCL6,CXCL9,DOCK2,FCGR2B,GABBR1,GABRP,IGKC,IL10RA,IL7R,KLRB1,PTPRC,PYCARD,SELL,STAT
2,CXCL6,CXCL9,FCGR2B,GABBR1,GABRP,IL10RA,IL7R,KLRB1,PTPRC,STAT4,TIMP1,XCL1
3,CTSV,CXCL1,CXCL10,CXCL11,CXCL2,CXCL6,CXCL9,DOCK2,ELF3,FCER1G,FCGR2B,GABBR1,GABRP,G
0,JCHAIN,KLRB1,LCN2,LTF,LYZ,MMP7,MPEG1,PLAC8,PTPRC,PYCARD,RNASE6,SELL,SLPI,SPON2,UBD
N2
2,CXCL6,CXCL9,EGF,FCGR2B,GABBR1,GABRP,IL7R,KLRB1,PTPRC,PYCARD,SERPING1,STAT4,TIMP1,XC
7,TNFSF13B,XCL1
XCL2,CXCL9,EGF,FCER1G,FCGR2B,G6PC,GPR183,HCLS1,IGKC,IL10RA,KLRC4-KLRK1/KLRK1,LCN2,LY
2,CXCL9,EGF,FCER1G,FCGR2B,G6PC,GPR183,HCLS1,IGKC,KLRC4-KLRK1/KLRK1,LCN2,LTF,LYZ,PRNP,
XCL9,EGF,FCER1G,FCGR2B,G6PC,GPR183,HCLS1,IGKC,KLRC4-KLRK1/KLRK1,LYZ,PRNP,PTPRC,RAC2,
SV,CXCL1,CXCL10,CXCL11,CXCL9,DOCK2,ELF3,FCER1G,FCGR2B,GPR183,HCLS1,IGKC,IL10RA,IL7R,N
2,CD3D,CD52,CD53,CD69,CD8A,CORO1A,CP,CPA3,CSF2RB,CTSS,CXCL1,CXCL10,CXCL11,CXCL2,CX
1,CXCL10,CXCL9,ELF3,FCGR2B,GABRP,GZMA,IL10RA,JCHAIN,LCN2,LTF,LY96,LYZ,MMP7,MNDA,NFK
FCER1G,FCGR2B,GPR183,IGKC,IGLL1/IGLL5,IL10RA,IL7R,MZB1,NFKBIZ,PRNP,PTPRC,SASH3,SFN,STA
3,CTSV,CXCL1,CXCL10,DOCK2,EGF,ELF3,FCER1G,FCGR2B,G6PC,GPR183,HCST,HOPX,IGKC,IGLL1/IGL
,CXCL6,CXCL9,CYTIP,FCER1G,FCGR2B,IGHG3,IGK,IGKC,IGLL1/IGLL5,ISG20,KLK1,LCN2,LTF,LY86,NFKB
4,C1orf162,C3,CCL19,CCL4,CCL5,CCN1,CD2,CD3D,CD52,CD53,CD69,CD8A,CORO1A,CP,CPA3,CSF2
2B,FCER1G,FCGR2B,GABRP,HCLS1,IL7R,LAPTM5,LCN2,LY96,MMP7,NNMT,PRNP,PTPRC,SERPINA3,S
,IL7R,LTF,PTPRC,RAC2,REG1A,SERPING1
,CXCL11,CXCL2,CXCL6,CXCL9,DOCK2,FCER1G,FCGR2B,G6PC,GABBR1,GPR183,GZMA,HCST,HOPX,I
IMP1,VCAN
L5,IL7R,JCHAIN,LTF,LY86,LYZ,NFKBIZ,PTPRC,PYCARD,SASH3,SFN,SLC34A2,TIMP1,TNFSF13B,VCAN
CXCL10,CXCL2,ETNPPL,FCER1G,FCGR2B,G6PC,GABBR1,GABRP,HCST,HLA-DQA2,IGKC,IL7R,KLRB1,K
,CCN1,CP,CSF2RB,CXCL1,CXCL10,CXCL11,CXCL9,EGF,ELF3,FCGR2B,G6PC,GABBR1,GZMA,HCLS1,HL
K2,FCER1G,IGLL1/IGLL5,IL7R,LCN2,NFKBIZ,PTPRC,SASH3,SLA,STAT4,TIMP1
,CXCL9,EGF,SELL,SERPING1,SLPI
MK,LTF,PRNP,PYCARD,RAC2,SERPINA3
PTPRC,RAC2,REG1A,SASH3,SELL,SERPING1
C5,PTPRC,PYCARD,SASH3,SELL,SFN,SLA,TNFSF13B

P,SERPING1,SFN,SLPI,VCAN
TPRC,SFN,XCL1

,DOCK2,EGF,ELF3,FCER1G,FCGR2B,FGB,G6PC,GPR183,HCLS1,IGHV1-69,IGHV3-23,IGKC,IGKV3-20,IG
R2B,FGB,G6PC,GPR183,GZMA,HCLS1,IGHV1-69,IGHV3-23,IGKC,IGKV3-20,IGLL1/IGLL5,IGLV1-44,IGL
,CXCL2,CXCL6,CXCL9,CYTIP,DOCK2,EGF,ELF3,FCER1G,FCGR2B,FGB,G6PC,GABBR1,GABRP,GPR183,C
)8A,CORO1A,CP,CPA3,CSF2RB,CST7,CSTA,CTSS,CTSV,CXCL1,CXCL10,CXCL11,CXCL2,CXCL6,CXCL9,
CXCL9,EGF,ELF3,EVI2A,FCER1G,FCGR2B,FXYD5,GABBR1,GABRP,GZMA,HCLS1,HCST,HLA-DQA2,IGK
CYTIP,DOCK2,EGF,ELF3,EVI2B,FCER1G,FCGR2B,FGB,G6PC,GPR183,GZMA,HCLS1,HCST,IGK,IGKC,IGLL
IGKC,IGLL1/IGLL5,IL10RA,IL7R,JCHAIN,LCN2,MMP7,MZB1,NFKBIZ,NLRC5,PLAC8,PRNP,PTPRC,PYCA
CL1,CXCL10,CXCL11,CXCL2,CXCL6,CXCL9,CYTIP,DOCK2,EGF,EMB,EVI2A,FCER1G,FCGR2B,FGB,FXYD
R183,HCLS1,IGKC,IGLL1/IGLL5,IL10RA,IL7R,ISG20,KLRB1,KLRC4-KLRK1/KLRK1,LAPTM5,LTF,LY86,LY96
CGR2B,GPR183,HCLS1,IGKC,IGLL1/IGLL5,IL10RA,IL7R,ISG20,KLRB1,KLRC4-KLRK1/KLRK1,LAPTM5,LCN
LF3,EVI2B,FCER1G,FCGR2B,GPR183,HCLS1,HCST,IGK,IGKC,IGLL1/IGLL5,IL10RA,IL7R,ISG20,JCHAIN,KL
CER1G,FCGR2B,FXYD5,G6PC,GABBR1,GABRP,GZMA,HCLS1,HCST,HLA-DQA2,IGLL1/IGLL5,IL10RA,IL
P,GZMA,HCLS1,HCST,HLA-DQA2,IGKC,IL10RA,IL7R,KLRB1,LCN2,LTF,LY86,LYZ,MPEG1,MS4A6A,MZB
,DOCK2,EGF,FCER1G,FCGR2B,FGB,FXYD5,G6PC,GABBR1,GPR171,GPR183,GZMA,HCLS1,IGHG3,IGK,IL
CL2,CXCL6,CXCL9,CYTIP,EGF,ELF3,FCER1G,FCGR2B,FGB,GABBR1,GABRP,GZMA,HCLS1,HCST,HLA-D
2B,G6PC,GPR183,GZMA,GZMK,HCST,IGHG3,IGHV1-69,IGHV3-23,IGKC,IGKV3-20,IGLV1-44,IGLV2-2
,HLA-DQA2,IGKC,IL10RA,IL7R,ISG20,KLRB1,LCN2,LTF,LYZ,MMP7,MS4A6A,MS4A7,NLRC5,PRNP,PTP
BR1,GABRP,GZMA,HCLS1,HLA-DQA2,IGKC,IL10RA,IL7R,ISG20,KLRB1,LCN2,LTF,LYZ,MAFF,MMP7,MS
1G,FCGR2B,G6PC,GABBR1,GABRP,GZMA,GZMK,HCLS1,HCST,HOPX,IGHG3,IGK,IL10RA,IL7R,KLK1,KL
LTF,MZB1,NFKBIZ,PRNP,PTPRC,RAC2,SASH3,SELL,SFN,STAT4,TIMP1,TNFRSF17,TNFSF13B,UBD
A,IL7R,KLK1,LCN2,LTF,MMP7,MZB1,NFKBIZ,PRNP,PTPRC,RAC2,SASH3,SELL,SERPING1,SFN,SLPI,SOX
IGHG3,IGK,IGLL1/IGLL5,IL10RA,IL7R,ISG20,JCHAIN,KLRC4-KLRK1/KLRK1,LAPTM5,LCN2,LTF,LY86,LY9
23,IGLV3-25,IL7R,JCHAIN,LCN2,LY86,LY96,MNDA,MZB1,NFKBIZ,PRNP,PTPRC,PYCARD,RAC2,SASH3
RC4-KLRK1/KLRK1,LCN2,LTF,LYZ,MAFF,MNDA,PLAC8,PTPRC,PYCARD,RAC2,SASH3,SELL,SERPINA3,
4,TIMP1,XCL1

PR183,IGKC,IGLL1/IGLL5,IL10RA,IL7R,KLRB1,MZB1,NFKBIZ,PRNP,PTPRC,SASH3,SFN,STAT4,TIMP1,T
),XCL1

L1

Z,PRNP,PTPRC,RAC2,SELL,SLA,SLC12A3,STAT4,TIMP1,XCL1,XCL2
PTPRC,RAC2,SELL,SLA,SLC12A3,XCL1,XCL2
SELL,SLA,XCL1,XCL2
KBIZ,PRNP,PTPRC,PYCARD,RAC2,SASH3,SELL,STAT4,TIMP1,TNFRSF17,TNFSF13B,XCL1
CL6,CXCL9,DOCK2,EGF,EMB,EVI2A,FCER1G,FCGR2B,FGB,GABRP,GPR183,GZMA,GZMK,HCLS1,HLA-
IZ,PTPRC,PYCARD,RAC2,SELL,SERPING1,SLPI,SPON2,STAT4,TIMP1
T4,TNFRSF17,TNFSF13B
L5,IL10RA,IL7R,LCN2,MMP7,MZB1,NFKBIZ,NLRC5,PRNP,PROM1,PTPRC,PYCARD,REG1A,SASH3,SELL
IZ,PROM1,PTPRC,SELL,SFN,SLPI,TIMP1,TNFRSF17,UBD
RB,CST7,CSTA,CTSS,CTSV,CXCL1,CXCL10,CXCL11,CXCL2,CXCL6,CXCL9,CYP3A7,CYTIP,DOCK2,EGF,E
LA,SLC12A3,TIMP1,VCAN,VSIG4

K,IL7R,KLRB1,LCN2,LTF,LY96,LYZ,MAFF,MMP7,NFKBIZ,NLRC5,PRNP,PROM1,PTPRC,PYCARD,RAC2,

LRC4-KLRK1/KLRK1,LCN2,LTF,MAFF,MS4A6A,NFKBIZ,NNMT,PRNP,PTPRC,PVALB,PYCARD,RDH12,R
A-DQA2,HOPX,IGHG2,IGLL1/IGLL5,ISG20,LAPTM5,LCN2,LTF,LY86,MMP7,MS4A7,OTOGL,PHLDA2,P

GLL1/IGLL5,IGLV1-44,IGLV2-23,IGLV3-25,IL10RA,IL7R,JCHAIN,KLK1,KLRC4-KLRK1/KLRK1,LCN2,LTF,L
V2-23,IGLV3-25,IL10RA,IL7R,JCHAIN,KLK1,KLRB1,KLRC4-KLRK1/KLRK1,LAPTM5,LCN2,LTF,LY96,LYZ
GZMA,HCLS1,HCST,HLA-DQA2,IGHG2,IGHG3,IGHV1-69,IGHV3-23,IGKC,IGKV3-20,IGLV1-44,IGLV2-
CYP3A7,CYTIP,DOCK2,EGF,ELF3,EMB,ETNPPL,EVI2A,EVI2B,FCER1G,FCGR2B,FGB,FXYD5,G6PC,GABBR
C,IL10RA,IL7R,JCHAIN,KLRB1,LAPTM5,LCN2,LTF,LY86,LY96,LYZ,MAFF,MMP7,MPEG1,MS4A6A,MZB1,
1/IGLL5,IL10RA,IL7R,ISG20,JCHAIN,KLK1,KLRB1,KLRC4-KLRK1/KLRK1,LAPTM5,LCN2,LTF,LY86,LY96,L
RD,RAC2,SASH3,SELL,SERPING1,SFN,SLA,SLPI,SOST,SOX9,SPON2,STAT4,TFPI2,TIMP1,TNFRSF17,TN
5,GABBR1,GABRP,GPR183,GZMA,GZMK,HCLS1,HCST,HLA-DQA2,IGKC,IGLL1/IGLL5,IGLV3-25,IL10R
6,MNDA,MZB1,NFKBIZ,PRNP,PTPRC,PYCARD,RAC2,SASH3,SELL,SFN,SLPI,STAT4,TIMP1,TNFRSF17,TN
N2,LTF,LY86,LY96,MNDA,MZB1,NFKBIZ,PRNP,PTPRC,PYCARD,RAC2,SASH3,SELL,SFN,SLPI,STAT4,TIM
LRB1,KLRC4-KLRK1/KLRK1,LAPTM5,LCN2,LTF,LY86,LY96,MNDA,MZB1,NFKBIZ,NLRC5,PRNP,PTPRC,P
7R,KLRB1,LAPTM5,LCN2,LTF,LY86,LYZ,MMP7,MPEG1,MS4A6A,MS4A7,MZB1,NNMT,OTOGL,PROM1,
1,NNMT,PRNP,PTPRC,PYCARD,RAC2,REG1A,RNASE6,SELL,SERPINA3,SERPING1,SLC12A3,STAT4,TIM
L10RA,IL7R,KLK1,KLRB1,KLRC4-KLRK1/KLRK1,LAPTM5,LCN2,LTF,LY96,LYZ,MMP7,NFKBIZ,NLRC5,PPP
QA2,IGKC,IL10RA,IL7R,ISG20,JCHAIN,KLK1,KLRB1,KLRC4-KLRK1/KLRK1,LAPTM5,LCN2,LTF,LY86,LY9
3,IGLV3-25,IL10RA,IL7R,JCHAIN,KLRB1,KLRC4-KLRK1/KLRK1,LCN2,LTF,LY96,MMP7,NFKBIZ,NLRC5,P
RC,PYCARD,SELL,SERPING1,STAT4,TFPI2,TIMP1,TNFSF13B,VSIG4,XCL1
S4A6A,MS4A7,NLRC5,PRNP,PTPRC,PVALB,PYCARD,RDH12,SELL,SERPINA3,SERPING1,SOX9,STAT4,T
RB1,KLRC4-KLRK1/KLRK1,LCN2,LTF,LY96,LYZ,MMP7,MNDA,MZB1,NFKBIZ,PHLDA2,PLAC8,PRNP,PR

X9,STAT4,TIMP1,TNFRSF17,TNFSF13B,UBD
96,MMP7,MNDA,NFKBIZ,NLRC5,PLAC8,PRNP,PTPRC,PVALB,PYCARD,RAC2,SELL,SERPINA3,SERPING
,SFN,SLA,SLPI,TIMP1,TNFRSF17,TNFSF13B,XCL1
SERPING1,SFN,SLA,SLPI,STAT4,TIMP1,XCL1

NFRSF17,TNFSF13B,XCL1

DQA2,IGKC,IGLL1/IGLL5,IGLV3-25,IL7R,ISG20,JCHAIN,KLK1,KLRC4-KLRK1/KLRK1,LAPTM5,LCN2,LTF

,SFN,SLA,SLC12A3,SOST,SOX9,STAT4,TIMP1,TNFRSF17,TNFSF13B,TRIM50

LF3,EMB,ETNPPL,FCER1G,FCGR2B,FXYD5,G6PC,GABBR1,GABRP,GPR183,GZMA,GZMK,HCLS1,HCST,

SASH3,SERPINA3,SERPING1,SLC34A2,SLPI,SOX9,STAT4,TFPI2,TIMP1,TMPRSS4,TNFRSF17,TNFSF13B,

EG1A,SELL,SERPINA3,SERPING1,SOX9,STAT4,TIMP1,TNFRSF17,VCAN,VSIG4
ROM1,RDH12,SELL,SERPING1,SFN,SLC34A2,SLPI,STAT4,TFPI2,TIMP1,TMPRSS4,TNFRSF17,TNFSF13B

.Y96,LYZ,MMP7,NFKBIZ,NNMT,PHLDA2,PRNP,PROM1,PTPRC,PYCARD,RAC2,SELL,SERPINA3,SERPIN
,MMP7,NFKBIZ,PRNP,PTPRC,PYCARD,RAC2,SASH3,SELL,SERPINA3,SERPING1,SLPI,SPON2,STAT4,TIN
-23,IGLV3-25,IL10RA,IL7R,ISG20,JCHAIN,KLK1,KLRB1,KLRC4-KLRK1/KLRK1,LAPTM5,LCN2,LTF,LY86,L
21,GABRP,GPR183,GZMA,GZMK,HCLS1,HCST,HLA-DQA2,HOPX,IGHG2,IGHG3,IGK,IGKC,IGLL1/IGLL5
,NFKBIZ,NKG7,NLRC5,NNMT,PLAC8,PTPRC,PYCARD,RAC2,REG1A,RNASE6,SELL,SERPINA3,SERPING1
.YZ,MMP7,MNDA,MZB1,NFKBIZ,NLRC5,PLAC8,PRNP,PTPRC,PYCARD,RAC2,SASH3,SELL,SERPINA3,SI
FSF13B,VCAN,VSIG4,XCL1
A,IL7R,ISG20,JCHAIN,KLK1,KLRB1,KLRC4-KLRK1/KLRK1,LAPTM5,LCN2,LTF,LY86,LY96,LYZ,MPEG1,MS
NFSF13B,UBD,VSIG4,XCL1
MP1,TNFRSF17,TNFSF13B,UBD,VSIG4,XCL1
YCARD,RAC2,SASH3,SELL,SFN,SLA,SLPI,STAT4,TIMP1,TNFRSF17,TNFSF13B,UBD,VSIG4,XCL1
,PTPRC,RAC2,RDH12,REG1A,RNASE6,SELL,SERPINA3,SERPING1,SLC12A3,SLC34A2,SLPI,STAT4,TFPI2
IP1,TNFSF13B,XCL1
1R18,PRNP,PTPRC,PYCARD,RAC2,RASD1,SASH3,SELL,SERPING1,SFN,SLPI,SOST,SOX9,SPON2,STAT4
5,LYZ,MMP7,MNDA,MPEG1,MS4A6A,MS4A7,MZB1,NFKBIZ,NLRC5,PRNP,PROM1,PTPRC,PYCARD,RE
PRNP,PTPRC,PYCARD,RAC2,REG1A,SASH3,SELL,SERPINA3,SLC12A3,SLC34A2,SLPI,STAT4,TNFRSF17,T

FPI2,TIMP1,TNFSF13B,VCAN,VSIG4,XCL1
OM1,PTPRC,PYCARD,RAC2,RASD1,RDH12,REG1A,SELL,SERPINA3,SFN,SLPI,SOST,SOX9,STAT4,TFPI2,

1,SLPI,SPON2,STAT4,TIMP1,TMPRSS4,TNFRSF17,TNFSF13B,UBD,VCAN,VSIG4,XCL1

,LYZ,MNDA,MPEG1,MS4A6A,NNMT,PLAC8,PROM1,PTPRC,RAC2,SASH3,SELL,SERPINA3,SLA,SLC34A

HLA-DQA2,HOPX,IGKC,IGLL1/IGLL5,IGLV3-25,IL7R,ISG20,JCHAIN,KLK1,KLRC4-KLRK1/KLRK1,LAPTM

,UBD,VCAN,VSIG4

3,VCAN,XCL1,XCL2

G1,SFN,SLPI,SOX9,SPON2,TFPI2,TIMP1,TMPRSS4,TNFSF13B,VCAN,XCL1,XCL2
MP1,TNFSF13B,VCAN,XCL1
Y96,LYZ,MMP7,MNDA,MPEG1,MS4A6A,MS4A7,MZB1,NFKBIZ,NLRC5,PLAC8,PRNP,PROM1,PTPRC,P
,IGLV3-25,IL10RA,IL7R,ISG20,JCHAIN,KLK1,KLRB1,KLRC4-KLRK1/KLRK1,LAPTM5,LCN2,LTF,LY86,LY9
1,SLC12A3,SLPI,STAT4,TIMP1,TNFRSF17,TNFSF13B,UBD,VSIG4,XCL1
ERPING1,SFN,SLA,SLPI,SPON2,STAT4,TIMP1,TNFRSF17,TNFSF13B,UBD,VCAN,VSIG4,XCL1

4A6A,MS4A7,MZB1,NFKBIZ,NLRC5,NNMT,PLAC8,PRNP,PROM1,PTPRC,PYCARD,RAC2,RNASE6,SAS

,TIMP1,TMPRSS4,TNFSF13B,VCAN,XCL1

,TIMP1,TNFRSF17,TNFSF13B,VCAN,XCL1,XCL2
G1A,SELL,SERPINA3,SERPING1,SFN,SLPI,SPON2,STAT4,TFPI2,TIMP1,TNFRSF17,TNFSF13B,UBD,VSIG
TNFSF13B,UBD,VSIG4

TIMP1,TNFRSF17,TNFSF13B,UBD,VCAN,VSIG4,XCL1

2,SLC7A13,SLPI,SOST,STAT4,TIMP1,TNFRSF17,TNFSF13B,UBD,VCAN

15,LCN2,LTF,LY86,LYZ,MMP7,MNDA,MPEG1,MS4A6A,MS4A7,NLRC5,NNMT,OTOGL,PLAC8,PRNP,P

YCARD,RAC2,REG1A,RNASE6,SASH3,SELL,SERPINA3,SERPING1,SFN,SLA,SLPI,SPON2,STAT4,TFPI2,TI
6,LYZ,MAFF,MMP7,MNDA,MPEG1,MS4A6A,MS4A7,MZB1,NFKBIZ,NKG7,NLRC5,NNMT,OTOGL,PHLD

H3,SELL,SERPINA3,SERPING1,SFN,SLA,SLC7A13,SLPI,SPON2,STAT4,TIMP1,TNFRSF17,TNFSF13B,UBD

4,XCL1

ROM1,PTPRC,PYCARD,RAC2,RDH12,SASH3,SELL,SERPINA3,SERPING1,SFN,SLA,SLC12A3,SLC34A2,SL

MP1,TNFRSF17,TNFSF13B,TRIM50,UBD,VCAN,VSIG4,XCL1
)A2,PLAC8,PRNP,PROM1,PTPRC,PYCARD,RAC2,RDH12,REG1A,RNASE6,SASH3,SELL,SERPINA3,SERP

),VCAN,VSIG4,XCL1

.C7A13,SLPI,SOST,SOX9,STAT4,TFPI2,TIMP1,TMPRSS4,TNFRSF17,TNFSF13B,UBD,VCAN

ING1,SFN,SLA,SLC12A3,SLC34A2,SLC7A13,SLPI,SOST,SOX9,SPON2,STAT4,TFPI2,TIMP1,TMPRSS4,TN

[illegible]FRSF17,TNFSF13B,TRIM50,UBD,VCAN,VSIG4,XCL1,XCL2
