# Supplementary material for: The Potential Diagnostic Value of Immune-Related Genes in Interstitial Fibrosis and Tubular Atrophy after Kidney Transplantation
Source: J Immunol Res. 2022 Jun 17;2022:7212852. doi: 10.1155/2022/7212852 (PMC9232312; doi:10.1155/2022/7212852)
Supplement: Supplementary Materials — Supplementary Figure 1: GSEA enrichment analysis of the IF/TA group. Supplementary Figure 2: correlation analysis between ANGPTL3 and differentially expressed immune infiltrating cells. Supplementary Figure 3: correlation analysis between APOH and differentially expressed immune infiltrating cells. Supplementary Figure 4: correlation analysis between EGF and differentially expressed immune infiltrating cells. Supplementary Figure 5: correlation analysis between FCGR2B and differentially expressed immune infiltrating cells. Supplementary Figure 6: correlation analysis between HLA-DQA2 and differentially expressed immune infiltrating cells. Supplementary Figure 7: correlation analysis between LTF and differentially expressed immune infiltrating cells. Supplementary Figure 8: IPA analysis shows the interaction network of diagnostic genes: EGF and LTF (8A), ANGPTL3 (8B), FCGR2B and APOH (8C), and HLA-DQA2 (8D). Merged the above four independent networks to comprehensively analyze the interaction of diagnostic genes (8E). Supplementary Table 1: immune-related genes. Supplementary Table 2: KEGG pathway in normal group. Supplementary Table 3: pathway of ANGPTL3 gene. Supplementary Table 4: pathway of APOH gene. Supplementary Table 5: pathway of EGF gene. Supplementary Table 6: ingenuity canonical pathways. Supplementary Table 7: category. [file 7212852.f1.zip › 7212852.f1/supplementary table2.pdf]

Symbol	logFC	AveExpr	t	P.Value	adj.P.Val	B
ISG20	1.643677	5.008916	10.40607	3.23E-19	5.72E-15	32.93751
HCLS1	1.401327	6.75469	8.9593	1.64E-15	5.65E-12	24.71009
CORO1A	1.64865	5.491596	8.94176	1.81E-15	5.65E-12	24.61195
IGKV3-20	2.650888	8.160569	8.932295	1.91E-15	5.65E-12	24.55901
LOC10029	2.650888	8.160569	8.932295	1.91E-15	5.65E-12	24.55901
IGLL1	2.031837	6.293726	8.815638	3.76E-15	8.32E-12	23.90777
IGLL3P	2.031837	6.293726	8.815638	3.76E-15	8.32E-12	23.90777
GABBR1	1.052582	6.542972	8.744705	5.66E-15	1.11E-11	23.51292
SOX9	1.388878	6.068638	8.631303	1.09E-14	1.92E-11	22.88355
LOC10029	1.988109	5.876297	8.524948	2.00E-14	3.22E-11	22.29549
IGK	1.741313	5.499596	8.393452	4.24E-14	5.77E-11	21.57151
FXYD5	1.278359	5.421235	8.342639	5.66E-14	7.13E-11	21.2927
AGR2	1.33725	5.476603	8.31746	6.53E-14	7.13E-11	21.15475
AGR3	1.264506	4.179186	8.310847	6.78E-14	7.13E-11	21.11854
IL7R	1.775496	5.626119	8.307197	6.92E-14	7.13E-11	21.09856
IGKC	1.433969	5.038679	8.289466	7.65E-14	7.13E-11	21.00154
GPR171	1.352982	4.689944	8.224177	1.11E-13	8.82E-11	20.64488
CD2	1.568924	5.346959	8.218096	1.15E-13	8.82E-11	20.61171
CP	1.176229	4.45803	8.147051	1.71E-13	1.26E-10	20.22478
ARHGAP3(	1.151178	5.452932	8.139427	1.79E-13	1.27E-10	20.18334
BIRC3	1.140153	6.679633	8.108323	2.13E-13	1.45E-10	20.01436
RAC2	1.058787	5.229493	8.05165	2.93E-13	1.92E-10	19.70706
CD69	1.254967	4.101764	7.989756	4.14E-13	2.54E-10	19.37234
MAFF	1.115823	4.866629	7.988837	4.17E-13	2.54E-10	19.36737
ARL4C	1.267695	5.648968	7.976268	4.47E-13	2.64E-10	19.29952
LOC10013	2.525731	5.637721	7.936241	5.59E-13	3.09E-10	19.08369
LOC10028	2.120061	6.335188	7.93067	5.77E-13	3.09E-10	19.05368
SERPING1	1.00124	8.519553	7.908896	6.51E-13	3.28E-10	18.93647
NFKBIZ	1.282996	7.058616	7.901259	6.80E-13	3.28E-10	18.89539
CXCL1	1.519235	4.22142	7.899436	6.87E-13	3.28E-10	18.88558
IGLV2-23	2.401045	6.184724	7.888478	7.30E-13	3.31E-10	18.82667
LOC10029	2.401045	6.184724	7.888478	7.30E-13	3.31E-10	18.82667
STAT4	1.003133	4.372517	7.856816	8.71E-13	3.82E-10	18.65661
SLA	1.0238	6.013869	7.853791	8.86E-13	3.82E-10	18.64038
LOC10029	1.8539	5.1947	7.848691	9.11E-13	3.84E-10	18.61301
C16orf54	1.27821	4.91697	7.797769	1.21E-12	4.55E-10	18.34017
SFN	1.255256	4.88395	7.732011	1.74E-12	6.42E-10	17.98884
LOC65249	1.287074	4.479517	7.727734	1.78E-12	6.44E-10	17.96603
MZB1	1.40341	4.106535	7.681104	2.31E-12	8.01E-10	17.71766
CCL5	1.682794	6.370392	7.619396	3.24E-12	1.08E-09	17.38991
PPP1R18	1.080726	6.537565	7.59578	3.69E-12	1.19E-09	17.26475
PYCARD	1.028666	5.656879	7.589711	3.82E-12	1.21E-09	17.23262
SASH3	1.076265	4.636625	7.553219	4.66E-12	1.40E-09	17.03961
IGHV4-31	1.108883	4.622045	7.415285	9.91E-12	2.58E-09	16.31358
NLRC5	1.072501	5.988091	7.377701	1.22E-11	3.03E-09	16.11674
EGF	-1.53347	8.71765	-7.34663	1.44E-11	3.49E-09	15.95432
UBD	1.445074	7.845847	7.21203	2.98E-11	6.19E-09	15.25431
CXCL2	1.09345	4.100886	7.210214	3.01E-11	6.19E-09	15.2449
PHLDA2	1.001019	4.538297	7.148166	4.20E-11	8.07E-09	14.9242
TMPRSS4	1.056347	6.437153	7.145436	4.26E-11	8.10E-09	14.91011
DOCK2	1.052539	4.951707	7.142786	4.32E-11	8.13E-09	14.89645
IGHG2	1.804937	4.638779	7.093289	5.63E-11	9.96E-09	14.64162
LOC10029	1.804937	4.638779	7.093289	5.63E-11	9.96E-09	14.64162
LTF	2.584649	7.251204	7.079502	6.06E-11	1.05E-08	14.57078
LOC10029	1.458731	3.996365	7.070347	6.36E-11	1.05E-08	14.52378
LOC10029	1.458731	3.996365	7.070347	6.36E-11	1.05E-08	14.52378
PVALB	-1.17454	7.931683	-7.06745	6.46E-11	1.06E-08	14.50893

CPA3	1.669115	5.486965	7.032297	7.79E-11	1.20E-08	14.32874
CSF2RB	1.21343	4.88673	7.011658	8.69E-11	1.32E-08	14.22315
CD8A	1.271655	4.717732	7.000881	9.21E-11	1.38E-08	14.16808
CXCL6	1.796922	4.600191	6.991153	9.69E-11	1.43E-08	14.1184
LAPTM5	1.231011	7.374877	6.980418	1.03E-10	1.45E-08	14.06361
TIMP1	1.105695	9.672495	6.951732	1.19E-10	1.65E-08	13.91742
SERPINA3	2.000388	5.97566	6.944704	1.24E-10	1.69E-08	13.88164
RNASE6	1.286707	7.444698	6.929296	1.35E-10	1.76E-08	13.80328
IGLL5	1.689619	4.908784	6.927042	1.36E-10	1.76E-08	13.79182
IGLV3-16	1.689619	4.908784	6.927042	1.36E-10	1.76E-08	13.79182
IGLV3-25	1.689619	4.908784	6.927042	1.36E-10	1.76E-08	13.79182
GABRP	1.111616	4.254822	6.881684	1.73E-10	2.10E-08	13.56164
ARHGAP9	1.026785	4.91578	6.871773	1.82E-10	2.16E-08	13.51145
C1orf162	1.145272	6.192983	6.842686	2.12E-10	2.39E-08	13.36433
ACKR1	1.732087	5.249549	6.789484	2.81E-10	3.07E-08	13.09606
IL10RA	1.199425	5.741818	6.779605	2.96E-10	3.19E-08	13.04635
CD53	1.263399	7.648005	6.766748	3.16E-10	3.38E-08	12.98172
CYTIP	1.123809	5.34351	6.761608	3.25E-10	3.44E-08	12.9559
LOC10029(	1.249217	4.64878	6.739012	3.65E-10	3.72E-08	12.84251
LOC10029:	1.249217	4.64878	6.739012	3.65E-10	3.72E-08	12.84251
EVI2B	1.492502	6.107587	6.724561	3.94E-10	3.96E-08	12.77009
JCHAIN	2.447171	7.608297	6.703854	4.39E-10	4.27E-08	12.66645
KLRB1	1.285532	5.674368	6.694835	4.60E-10	4.38E-08	12.62137
NKG7	1.107629	4.733653	6.691938	4.67E-10	4.40E-08	12.60689
TNFRSF17	1.306585	3.719876	6.648211	5.86E-10	5.37E-08	12.38879
RASD1	1.087053	6.401649	6.615218	6.95E-10	6.18E-08	12.2247
ELF3	1.057963	5.670122	6.604324	7.35E-10	6.48E-08	12.17062
GZMA	1.468193	5.331318	6.602269	7.43E-10	6.49E-08	12.16042
CST7	1.009963	4.993537	6.602007	7.44E-10	6.49E-08	12.15912
SELL	1.265318	5.282165	6.599709	7.53E-10	6.50E-08	12.14772
CYR61	1.032628	5.769575	6.584479	8.15E-10	6.93E-08	12.07221
IGHG3	1.028892	4.305828	6.499609	1.26E-09	1.01E-07	11.65313
MMP7	1.436137	9.259862	6.468423	1.48E-09	1.15E-07	11.49986
IGHV3-23	1.295825	4.283386	6.452275	1.61E-09	1.24E-07	11.42065
NNMT	1.136048	5.3127	6.44525	1.66E-09	1.27E-07	11.38623
LY86	1.056297	6.039907	6.389551	2.21E-09	1.60E-07	11.11399
KLRK1	1.095805	5.175653	6.367198	2.48E-09	1.76E-07	11.0051
SPON2	1.19525	6.65135	6.356565	2.61E-09	1.85E-07	10.95338
C1QB	1.285945	7.526016	6.323566	3.09E-09	2.10E-07	10.79315
HCST	1.010738	6.425965	6.310219	3.30E-09	2.23E-07	10.72848
CD1C	1.089171	5.570196	6.305742	3.38E-09	2.27E-07	10.7068
HOPX	1.388761	6.565986	6.273291	3.98E-09	2.61E-07	10.54994
PLAC8	1.422655	5.301152	6.249027	4.50E-09	2.86E-07	10.43293
EVI2A	1.124315	5.759986	6.248882	4.50E-09	2.86E-07	10.43224
CYAT1	1.104033	4.22491	6.24781	4.52E-09	2.86E-07	10.42708
IGLV1-44	1.104033	4.22491	6.24781	4.52E-09	2.86E-07	10.42708
LOC10029(	1.324428	4.309547	6.171942	6.62E-09	3.83E-07	10.06293
FCGR2B	1.133324	4.979643	6.166645	6.79E-09	3.92E-07	10.03761
C1QC	1.111573	6.06066	6.15517	7.19E-09	4.12E-07	9.982776
SLC34A2	1.028367	4.427865	6.117757	8.66E-09	4.78E-07	9.804413
IGHV1-69	1.301475	4.039939	6.112682	8.89E-09	4.82E-07	9.780267
EMB	1.116572	6.211703	6.111715	8.93E-09	4.83E-07	9.775668
TRIM50	-1.08752	5.315961	-6.10945	9.03E-09	4.87E-07	9.764892
XCL2	1.250388	5.033975	6.080869	1.04E-08	5.45E-07	9.629168
GPR183	1.121723	4.421846	6.07826	1.05E-08	5.50E-07	9.616796
TNFSF13B	1.005315	6.148947	6.064785	1.13E-08	5.80E-07	9.552945
ADGRV1	-1.39099	7.10956	-6.00022	1.55E-08	7.54E-07	9.24816
CD3D	1.371134	6.231555	5.990025	1.63E-08	7.85E-07	9.200213

XCL1	1.143033	5.051431	5.971362	1.78E-08	8.47E-07	9.112559
CTSV	-1.14424	7.410939	-5.88293	2.75E-08	1.21E-06	8.699403
REG1A	1.60207	5.018889	5.880299	2.78E-08	1.22E-06	8.687189
VSIG4	1.146077	6.661703	5.871291	2.91E-08	1.27E-06	8.64532
ARPC1B	1.29889	7.315583	5.841541	3.36E-08	1.44E-06	8.507331
SLPI	1.661132	7.33028	5.795427	4.20E-08	1.72E-06	8.294279
LOC10029(	1.068802	4.280704	5.744584	5.36E-08	2.12E-06	8.060568
KLK1	-1.43723	7.992011	-5.7343	5.63E-08	2.21E-06	8.013429
TFPI2	1.11749	5.252751	5.721668	5.98E-08	2.30E-06	7.955647
AFM	-1.63014	7.731089	-5.71176	6.27E-08	2.40E-06	7.910379
FCER1G	1.097626	6.568819	5.680184	7.29E-08	2.67E-06	7.766371
CD52	1.54885	7.006629	5.672286	7.57E-08	2.75E-06	7.73043
LCN2	1.267345	4.767688	5.657462	8.13E-08	2.91E-06	7.663059
MPEG1	1.096905	6.503437	5.644132	8.66E-08	3.06E-06	7.602574
MNDA	1.041656	5.193684	5.627751	9.36E-08	3.27E-06	7.528361
LOC72782(	1.024755	6.981671	5.620153	9.70E-08	3.37E-06	7.493988
MRO	-1.28624	6.787138	-5.61297	1.00E-07	3.46E-06	7.46151
MS4A6A	1.011672	6.869942	5.608228	1.03E-07	3.51E-06	7.440099
ANGPTL3	-1.02928	6.588582	-5.49967	1.71E-07	5.42E-06	6.95286
ALB	-2.04183	8.321406	-5.48786	1.81E-07	5.66E-06	6.90021
HLA-DQA:	1.22473	9.368021	5.466436	2.00E-07	6.14E-06	6.804896
PROM1	1.190974	8.930425	5.426883	2.40E-07	7.15E-06	6.629573
GZMK	1.265949	6.091295	5.337117	3.63E-07	1.01E-05	6.234755
CXCL11	1.526859	4.735319	5.187393	7.19E-07	1.78E-05	5.585954
LOC72794	-1.1308	7.38698	-5.15832	8.19E-07	1.99E-05	5.461425
CSTA	1.05016	5.582389	5.156246	8.27E-07	2.01E-05	5.452546
TMEM207	-1.19713	6.532351	-5.13401	9.14E-07	2.17E-05	5.357637
CCL4	1.291272	5.433156	5.114512	9.97E-07	2.33E-05	5.274647
VCAN	1.41001	6.004765	5.094953	1.09E-06	2.49E-05	5.191612
LY96	1.032781	6.11806	5.074002	1.19E-06	2.69E-05	5.102909
MS4A7	1.080739	6.746472	5.056219	1.29E-06	2.86E-05	5.027814
CYP3A7	-1.0359	6.612956	-5.04939	1.33E-06	2.93E-05	4.999035
APOH	-1.28341	8.555293	-5.02096	1.51E-06	3.24E-05	4.879463
SLC12A3	-1.03486	6.797852	-4.98133	1.80E-06	3.70E-05	4.713593
PTPRC	1.179617	6.021686	4.97864	1.82E-06	3.72E-05	4.702353
C3	1.062424	8.419015	4.87578	2.86E-06	5.46E-05	4.276263
FGB	1.458114	5.063497	4.871016	2.92E-06	5.53E-05	4.25668
CCL19	1.37014	7.732347	4.777125	4.37E-06	7.67E-05	3.873522
G6PC	-1.04407	7.188796	-4.69133	6.30E-06	0.000102	3.528082
SOST	-1.07818	6.936848	-4.65771	7.26E-06	0.000114	3.393958
CTSS	1.017767	6.993899	4.462472	1.63E-05	0.000215	2.629174
RDH12	-1.01091	7.718154	-4.38014	2.28E-05	0.000277	2.314018
LYZ	1.185593	7.6265	4.260202	3.69E-05	0.00041	1.862867
OTOGL	-1.01385	5.064083	-4.08598	7.29E-05	0.000715	1.22481
PRNP	-1.24065	7.399553	-4.04064	8.68E-05	0.000821	1.062187
ETNPPL	-1.01551	6.534343	-3.99467	0.000103	0.000942	0.898789
CXCL9	1.476748	7.196679	3.949344	0.000123	0.001076	0.739102
CXCL10	1.124406	7.401317	3.713679	0.000292	0.002144	-0.06738
SLC7A13	-1.18606	8.959636	-3.29334	0.001249	0.006683	-1.40332
