# Supplementary material for: The Potential Diagnostic Value of Immune-Related Genes in Interstitial Fibrosis and Tubular Atrophy after Kidney Transplantation
Source: J Immunol Res. 2022 Jun 17;2022:7212852. doi: 10.1155/2022/7212852 (PMC9232312; doi:10.1155/2022/7212852)
Supplement: Supplementary Materials — Supplementary Figure 1: GSEA enrichment analysis of the IF/TA group. Supplementary Figure 2: correlation analysis between ANGPTL3 and differentially expressed immune infiltrating cells. Supplementary Figure 3: correlation analysis between APOH and differentially expressed immune infiltrating cells. Supplementary Figure 4: correlation analysis between EGF and differentially expressed immune infiltrating cells. Supplementary Figure 5: correlation analysis between FCGR2B and differentially expressed immune infiltrating cells. Supplementary Figure 6: correlation analysis between HLA-DQA2 and differentially expressed immune infiltrating cells. Supplementary Figure 7: correlation analysis between LTF and differentially expressed immune infiltrating cells. Supplementary Figure 8: IPA analysis shows the interaction network of diagnostic genes: EGF and LTF (8A), ANGPTL3 (8B), FCGR2B and APOH (8C), and HLA-DQA2 (8D). Merged the above four independent networks to comprehensively analyze the interaction of diagnostic genes (8E). Supplementary Table 1: immune-related genes. Supplementary Table 2: KEGG pathway in normal group. Supplementary Table 3: pathway of ANGPTL3 gene. Supplementary Table 4: pathway of APOH gene. Supplementary Table 5: pathway of EGF gene. Supplementary Table 6: ingenuity canonical pathways. Supplementary Table 7: category. [file 7212852.f1.zip › 7212852.f1/supplementary table3.pdf]

Symbol	logFC	AveExpr	t	P.Value	adj.P.Val	B
KRT17P5	-42.9528	75.04348	-4.17698	0.000125	0.999016	-4.59452
RP5-1118D	51.25628	100.7396	3.927364	0.000276	0.999016	-4.59457
TMPRSS13	-39.2168	67.52532	-3.75106	0.000477	0.999016	-4.59462
LOC10013	-32.1718	53.2153	-3.72094	0.000523	0.999016	-4.59462
NOBOX	-30.6522	52.7602	-3.69177	0.000571	0.999016	-4.59463
SERPINA7	-24.3801	52.25114	-3.57735	0.000808	0.999016	-4.59465
LOC10013	-37.3926	90.36205	-3.56706	0.000834	0.999016	-4.59466
LCE1B	-27.4107	47.98259	-3.55552	0.000863	0.999016	-4.59466
FLJ43315	-21.04	28.55254	-3.46021	0.001147	0.999016	-4.59468
LOC44070	-10.6101	9.317385	-3.45186	0.001176	0.999016	-4.59468
IDH1-AS1	-28.7626	55.93655	-3.43824	0.001224	0.999016	-4.59469
LOC10192	-21.5052	33.28268	-3.30879	0.001788	0.999016	-4.59472
FAM71F2	-13.733	22.39132	-3.29462	0.001863	0.999016	-4.59472
ADORA2B	29.74976	49.01925	3.260485	0.002055	0.999016	-4.59473
LCT	-28.1295	54.50184	-3.24713	0.002136	0.999016	-4.59473
SERPINA3	256.1172	329.4074	3.190582	0.00251	0.999016	-4.59474
CHRM5	-22.6432	43.09086	-3.1901	0.002514	0.999016	-4.59474
LRFN1	-26.0948	48.27602	-3.16697	0.002685	0.999016	-4.59475
C15orf27	-62.4593	166.6657	-3.14716	0.002839	0.999016	-4.59475
LOC10192	-39.5183	143.3279	-3.12358	0.003035	0.999016	-4.59476
MIR4313	-20.7239	35.5426	-3.10304	0.003215	0.999016	-4.59476
EU250746	-25.1865	80.11572	-3.07074	0.003519	0.999016	-4.59477
TBC1D29	-30.4876	72.42854	-3.06975	0.003529	0.999016	-4.59477
LOC10192	-34.2811	72.18692	-3.06093	0.003617	0.999016	-4.59477
BC028670	-17.7586	27.80433	-3.05319	0.003695	0.999016	-4.59477
C1QTNF7	-84.7769	239.5343	-3.0528	0.003699	0.999016	-4.59477
FGF3	-11.2703	15.97071	-3.03035	0.003937	0.999016	-4.59478
AK125860	-17.874	29.01106	-3.02313	0.004017	0.999016	-4.59478
SLC26A8	-13.8437	18.19411	-3.0215	0.004035	0.999016	-4.59478
XRCC2	-82.1284	342.2921	-3.01725	0.004082	0.999016	-4.59478
LINC00964	-16.4623	36.14829	-3.0157	0.0041	0.999016	-4.59478
LINC00636	-25.0433	98.63393	-2.98199	0.004499	0.999016	-4.59479
RP5-1092A	-157.638	443.1292	-2.97139	0.004632	0.999016	-4.59479
FAS-AS1	-19.611	40.555	-2.96821	0.004672	0.999016	-4.59479
EXOSC6	-84.2575	413.9853	-2.95817	0.004802	0.999016	-4.59479
FAM166A	-19.0197	39.70964	-2.95339	0.004866	0.999016	-4.59479
PRAMEF11	-27.0253	85.0163	-2.94456	0.004984	0.999016	-4.5948
AX748417	-25.8674	68.42319	-2.92833	0.00521	0.999016	-4.5948
LOC10192	-48.738	223.5055	-2.91282	0.005434	0.999016	-4.5948
OR4D1	-14.1384	42.35553	-2.89708	0.00567	0.999016	-4.59481
LOC64275	-18.0309	19.92311	-2.8851	0.005857	0.999016	-4.59481
RTTN	-81.4917	375.9194	-2.88241	0.005899	0.999016	-4.59481
LOC10192	-31.8568	97.88733	-2.87212	0.006065	0.999016	-4.59481
AK025117	-72.9022	294.6878	-2.86018	0.006263	0.999016	-4.59481
LOC10192	-22.1176	31.91583	-2.85772	0.006305	0.999016	-4.59481
SPTBN4	-24.6448	106.8494	-2.8476	0.006478	0.999016	-4.59482
CHRNB4	-16.8713	41.93428	-2.84425	0.006536	0.999016	-4.59482
LOC38964	-27.1017	63.09472	-2.83931	0.006623	0.999016	-4.59482
ERICH6	-31.6146	57.35751	-2.83234	0.006748	0.999016	-4.59482
C19orf84	-29.7005	48.54293	-2.8226	0.006925	0.999016	-4.59482
DMRTB1	-18.8747	33.68519	-2.81122	0.007139	0.999016	-4.59482
PLAC8L1	-32.1359	126.283	-2.80916	0.007178	0.999016	-4.59482
AC002059.	-37.9071	111.1328	-2.80501	0.007257	0.999016	-4.59483
PCDHGA1	-12.1089	14.45025	-2.80357	0.007285	0.999016	-4.59483
DUX1	-47.6156	99.84023	-2.79315	0.007489	0.999016	-4.59483
LOC10192	-16.5969	34.04448	-2.78724	0.007607	0.999016	-4.59483
SLX4	-24.0286	84.41179	-2.78672	0.007618	0.999016	-4.59483

LA16c-83F	-26.2057	85.3961	-2.78669	0.007619	0.999016	-4.59483
KCNN1	-64.8471	120.9151	-2.78614	0.00763	0.999016	-4.59483
AKAP12	498.8013	1700.963	2.78418	0.007669	0.999016	-4.59483
NMNAT3	-86.6043	346.7995	-2.78314	0.00769	0.999016	-4.59483
ANKRD20A	-25.2806	48.41329	-2.78287	0.007696	0.999016	-4.59483
CNBD2	-14.7115	40.06462	-2.78271	0.007699	0.999016	-4.59483
ZNF138	-28.766	148.4404	-2.7817	0.00772	0.999016	-4.59483
TMEM238	11.57918	14.8306	2.779311	0.007769	0.999016	-4.59483
VSIG10L	37.15298	89.88	2.779112	0.007773	0.999016	-4.59483
C3orf20	-15.604	28.36265	-2.77164	0.007927	0.999016	-4.59483
PYCRL	60.96854	126.7954	2.759584	0.008183	0.999016	-4.59483
LOC10192	-28.6963	69.20504	-2.75477	0.008288	0.999016	-4.59484
LOC10106	-6.64939	10.14375	-2.75306	0.008325	0.999016	-4.59484
RP11-480A	-57.2878	253.4486	-2.75249	0.008338	0.999016	-4.59484
LUZP4	-28.2007	28.22965	-2.7504	0.008383	0.999016	-4.59484
SNORD3A	-17.9669	34.0414	-2.74494	0.008505	0.999016	-4.59484
LINC00659	13.58049	20.98586	2.743547	0.008536	0.999016	-4.59484
CENPP	-44.4695	163.0477	-2.73791	0.008663	0.999016	-4.59484
BGLT3	-34.2527	102.9943	-2.73027	0.008838	0.999016	-4.59484
LOC10272	-25.2149	102.6097	-2.72849	0.008879	0.999016	-4.59484
LOC44093	-24.3762	82.94451	-2.72167	0.009039	0.999016	-4.59484
CTC-471C	-11.1808	16.48991	-2.71329	0.009239	0.999016	-4.59484
LOC10050	-14.1924	26.11775	-2.71305	0.009244	0.999016	-4.59484
CNGA4	-20.3114	46.06622	-2.71012	0.009315	0.999016	-4.59484
SCN11A	-17.477	60.96436	-2.69971	0.009571	0.999016	-4.59485
AMELX	-16.186	44.85705	-2.69962	0.009573	0.999016	-4.59485
C12orf54	-11.2045	33.5022	-2.69931	0.009581	0.999016	-4.59485
OR2B4P	-32.0763	74.48554	-2.69626	0.009657	0.999016	-4.59485
SNORA21	-23.3085	83.63817	-2.69071	0.009798	0.999016	-4.59485
RP11-304C	-17.2661	23.36695	-2.68712	0.009889	0.999016	-4.59485
TKT	130.1126	644.939	2.679562	0.010085	0.999016	-4.59485
LOC10192	-38.0852	108.1819	-2.67634	0.010169	0.999016	-4.59485
MGC57346	-23.0028	68.87904	-2.67115	0.010307	0.999016	-4.59485
GUSBP2	-23.0047	76.61124	-2.67078	0.010316	0.999016	-4.59485
LRRC52	-61.1948	175.8973	-2.66879	0.01037	0.999016	-4.59485
MS4A7	162.4808	384.9156	2.667676	0.0104	0.999016	-4.59485
LOC10050	-18.7534	68.92499	-2.65816	0.010658	0.999016	-4.59486
RAPGEF4	-40.3675	201.5071	-2.65476	0.010752	0.999016	-4.59486
KCNQ3	-17.1233	86.59888	-2.64944	0.0109	0.999016	-4.59486
NAPA-AS1	-21.1981	73.71513	-2.64695	0.01097	0.999016	-4.59486
MESP1	-26.746	84.34498	-2.64366	0.011063	0.999016	-4.59486
KRT76	-11.1309	15.43821	-2.64222	0.011104	0.999016	-4.59486
GRK7	-12.9556	25.6948	-2.64092	0.011141	0.999016	-4.59486
C8orf17	-16.2875	34.08705	-2.63606	0.011281	0.999016	-4.59486
AC068831.	-22.2018	37.05541	-2.63403	0.01134	0.999016	-4.59486
PTOV1-AS	-21.0465	83.35741	-2.63033	0.011448	0.999016	-4.59486
LOC10050	-36.7939	178.9763	-2.62612	0.011572	0.999016	-4.59486
LINC00551	-19.9208	55.62942	-2.62099	0.011725	0.999016	-4.59486
AP000265.	11.89944	24.15342	2.620967	0.011726	0.999016	-4.59486
LOC28569	-17.3167	44.83213	-2.61951	0.01177	0.999016	-4.59486
MICA	-189.128	784.856	-2.61811	0.011812	0.999016	-4.59486
SH2D4B	-15.0414	43.2134	-2.61131	0.012019	0.999016	-4.59487
HIST1H2AI	-11.4217	37.3009	-2.60885	0.012094	0.999016	-4.59487
BRD1	-44.514	153.2096	-2.60866	0.0121	0.999016	-4.59487
LINC00629	-17.7219	50.33816	-2.60184	0.012312	0.999016	-4.59487
LOC10013	-12.7331	30.27022	-2.59603	0.012495	0.999016	-4.59487
FGF8	-6.48691	9.113177	-2.59414	0.012556	0.999016	-4.59487
BC031864	-28.4145	72.27819	-2.59234	0.012613	0.999016	-4.59487

REEP2	-62.1299	121.0607	-2.59119	0.01265	0.999016	-4.59487
TNNT2	-41.2614	125.3014	-2.59075	0.012664	0.999016	-4.59487
HOXA-AS3	-28.142	49.57154	-2.58929	0.012711	0.999016	-4.59487
FAM47B	-11.0987	20.87379	-2.58485	0.012855	0.999016	-4.59487
BCL2L10	-21.7346	100.1647	-2.57965	0.013026	0.999016	-4.59487
SLIT2-IT1	-15.4022	29.30286	-2.57627	0.013138	0.999016	-4.59487
LINC00998	-329.778	1533.713	-2.57351	0.01323	0.999016	-4.59487
LOC10050	-13.6811	32.8886	-2.5721	0.013277	0.999016	-4.59487
EPS8L3	-30.0478	67.69691	-2.56892	0.013384	0.999016	-4.59487
KIF20A	24.88384	42.60298	2.568782	0.013389	0.999016	-4.59487
LINC00202	-13.6665	35.24474	-2.56866	0.013393	0.999016	-4.59487
MRM1	-49.7714	193.5763	-2.56721	0.013443	0.999016	-4.59487
LOC10265	17.08219	31.88334	2.560056	0.013687	0.999016	-4.59488
HMX1	-17.2169	35.24868	-2.55983	0.013695	0.999016	-4.59488
CTAGE1	-12.7223	41.53128	-2.55833	0.013747	0.999016	-4.59488
NHLH2	-11.3556	24.57988	-2.55663	0.013806	0.999016	-4.59488
CDH4	-10.8909	43.84293	-2.55504	0.013862	0.999016	-4.59488
RPS29P28	-13.0071	21.76605	-2.55413	0.013893	0.999016	-4.59488
SLC7A11	-13.9862	42.84491	-2.55392	0.013901	0.999016	-4.59488
RP11-164F	-51.8531	119.2288	-2.55153	0.013985	0.999016	-4.59488
CCDC42B	-21.2287	62.16969	-2.55031	0.014028	0.999016	-4.59488
LINC01304	-17.7327	33.65095	-2.54935	0.014062	0.999016	-4.59488
FAM9B	-8.34893	19.36948	-2.54908	0.014071	0.999016	-4.59488
OSTCP1	15.40753	39.91453	2.546577	0.01416	0.999016	-4.59488
OR2H4P	-21.1649	74.57499	-2.54461	0.01423	0.999016	-4.59488
CACNG8	-17.0243	61.31463	-2.54342	0.014273	0.999016	-4.59488
LOC39070	-20.9333	77.54971	-2.5397	0.014407	0.999016	-4.59488
RP3-334F4	-61.667	252.973	-2.53753	0.014486	0.999016	-4.59488
KCNJ4	-11.0312	23.78104	-2.53712	0.014501	0.999016	-4.59488
AC012531.	-9.58465	23.11866	-2.53656	0.014521	0.999016	-4.59488
ANKRD18E	-28.4795	79.64662	-2.53254	0.014668	0.999016	-4.59488
KIAA0895	-116.763	597.792	-2.53183	0.014694	0.999016	-4.59488
LOC10192	30.89896	47.17521	2.530327	0.01475	0.999016	-4.59488
ENO1-AS1	-34.3617	123.9854	-2.52859	0.014814	0.999016	-4.59488
MAPK15	-22.151	74.65362	-2.51616	0.015282	0.999016	-4.59488
EP400NL	-12.8683	55.76901	-2.51403	0.015363	0.999016	-4.59488
LOC40131	-25.9632	81.05446	-2.51128	0.015469	0.999016	-4.59489
LINC00348	-8.96665	20.0046	-2.51103	0.015479	0.999016	-4.59489
DTX2	-17.1218	41.31919	-2.50331	0.015779	0.999016	-4.59489
CTXN1	26.4606	33.63257	2.496211	0.016061	0.999016	-4.59489
GLIPR1L1	-25.2871	83.14094	-2.4951	0.016105	0.999016	-4.59489
AC002064.	-14.0712	34.05206	-2.49074	0.016281	0.999016	-4.59489
PSD	-17.4187	38.0433	-2.48701	0.016432	0.999016	-4.59489
RP11-96H1	-11.5302	23.16613	-2.48668	0.016445	0.999016	-4.59489
MGC34796	-19.0373	36.81117	-2.48641	0.016456	0.999016	-4.59489
C2orf50	-4.88596	10.0827	-2.48618	0.016466	0.999016	-4.59489
LINC01015	-20.9279	32.64672	-2.48375	0.016565	0.999016	-4.59489
RFXAP	-40.1307	235.2047	-2.48137	0.016663	0.999016	-4.59489
ADAM5	-18.1964	56.5397	-2.47852	0.016781	0.999016	-4.59489
RP11-330C	-16.4385	68.66668	-2.47653	0.016864	0.999016	-4.59489
GOLGA4	-240.183	1421.645	-2.47358	0.016988	0.999016	-4.59489
RP1-155D2	-22.3757	100.9581	-2.47289	0.017017	0.999016	-4.59489
LOC10192	-21.8334	48.82625	-2.47275	0.017023	0.999016	-4.59489
LOC10050	-19.8216	52.63715	-2.47036	0.017123	0.999016	-4.59489
BEST4	9.3811	16.28946	2.46648	0.017288	0.999016	-4.59489
GOT2	-1581.14	6424.421	-2.46635	0.017294	0.999016	-4.59489
VIP	-16.6748	33.52804	-2.46331	0.017424	0.999016	-4.5949
LOC14869	-30.2903	81.22494	-2.46069	0.017537	0.999016	-4.5949

TMPRSS15	-27.9998	100.4007	-2.45643	0.017722	0.999016	-4.5949
FAM209A	-10.2142	25.39369	-2.45519	0.017776	0.999016	-4.5949
DYNLL2	-237.776	1323.048	-2.45406	0.017826	0.999016	-4.5949
LINGO2	-55.4777	180.4925	-2.45343	0.017853	0.999016	-4.5949
NTRK1	-18.024	31.07313	-2.44857	0.018068	0.999016	-4.5949
IER3	643.7898	2207.366	2.447207	0.018129	0.999016	-4.5949
DDX59	-90.8965	636.8156	-2.4455	0.018205	0.999016	-4.5949
LINC01091	-22.1718	45.77384	-2.44486	0.018234	0.999016	-4.5949
SCEL	7.266257	22.17366	2.439389	0.01848	0.999016	-4.5949
RPL7AL2	-63.2573	277.6635	-2.43679	0.018598	0.999016	-4.5949
LOC10029	-16.8412	40.10333	-2.43675	0.0186	0.999016	-4.5949
MROH6	-53.6281	208.3849	-2.42838	0.018985	0.999016	-4.5949
LOC10012	-19.3321	31.50627	-2.42306	0.019234	0.999016	-4.5949
SNRPN	-15.2763	63.10998	-2.42248	0.019261	0.999016	-4.5949
DGCR14	-42.3792	130.3341	-2.41975	0.01939	0.999016	-4.5949
IL36G	17.09072	27.93766	2.417689	0.019488	0.999016	-4.5949
FAM83F	-40.205	161.1346	-2.41385	0.019671	0.999016	-4.5949
ARHGAP28	-38.4784	165.9006	-2.41154	0.019782	0.999016	-4.59491
LOC10050	-67.801	261.7517	-2.40806	0.019951	0.999016	-4.59491
AX747826	24.29945	67.09094	2.407384	0.019984	0.999016	-4.59491
RBBP8NL	-17.2316	26.09512	-2.4043	0.020134	0.999016	-4.59491
LINC01019	-14.6362	30.77771	-2.40248	0.020223	0.999016	-4.59491
C6orf163	-22.8635	104.2388	-2.40128	0.020282	0.999016	-4.59491
LOC72986	10.1351	17.03116	2.396723	0.020508	0.999016	-4.59491
OCA2	-33.5259	75.07241	-2.39522	0.020583	0.999016	-4.59491
FAM228A	-21.2206	72.21572	-2.39451	0.020619	0.999016	-4.59491
GPLD1	-10.6368	55.66359	-2.39432	0.020628	0.999016	-4.59491
ZNF485	-20.0354	53.2643	-2.39256	0.020716	0.999016	-4.59491
PKMYT1	-69.793	225.3682	-2.39163	0.020763	0.999016	-4.59491
POLR3E	-5.63086	9.737078	-2.38862	0.020915	0.999016	-4.59491
LOC10192	-24.1142	65.43158	-2.3886	0.020916	0.999016	-4.59491
RP11-672L	-118.266	575.7246	-2.38842	0.020925	0.999016	-4.59491
LOC10050	-24.6639	122.5222	-2.38446	0.021127	0.999016	-4.59491
FLJ42351	-27.6226	131.8082	-2.38323	0.02119	0.999016	-4.59491
MIR302B	-29.7921	148.2507	-2.38305	0.021199	0.999016	-4.59491
LINC00948	-453.146	609.7772	-2.38199	0.021254	0.999016	-4.59491
AMH	13.29283	22.9434	2.381328	0.021288	0.999016	-4.59491
LINC00299	-14.7326	42.91917	-2.38113	0.021298	0.999016	-4.59491
CTC-550B	-25.3066	63.76184	-2.38074	0.021318	0.999016	-4.59491
ADAMTS16	31.52416	76.30534	2.379635	0.021375	0.999016	-4.59491
MMP7	1799.941	3602.791	2.37781	0.02147	0.999016	-4.59491
SOCS7	-142.631	904.7813	-2.37776	0.021472	0.999016	-4.59491
RP11-506	-38.6946	163.9045	-2.37335	0.021702	0.999016	-4.59491
AP006547.	-15.8766	35.74472	-2.37254	0.021745	0.999016	-4.59491
LOC10050	299.1624	562.4242	2.370321	0.021861	0.999016	-4.59491
MTSS1L	9.863543	39.6279	2.368108	0.021978	0.999016	-4.59491
GK-AS1	-27.2947	109.4782	-2.36808	0.02198	0.999016	-4.59491
LOC10050	-51.2716	268.9886	-2.36636	0.022071	0.999016	-4.59491
LINC00924	182.7142	532.5404	2.366027	0.022089	0.999016	-4.59491
ASB15	-38.0781	80.56304	-2.36145	0.022333	0.999016	-4.59492
BC041363	-11.8193	24.32792	-2.35742	0.022552	0.999016	-4.59492
MRC1	362.521	1306.206	2.357409	0.022552	0.999016	-4.59492
VPS37D	43.14435	152.6287	2.357196	0.022563	0.999016	-4.59492
TMEM255	-37.9411	90.3517	-2.35532	0.022665	0.999016	-4.59492
CYCSP33	-38.3024	135.2743	-2.35482	0.022692	0.999016	-4.59492
RP11-308D	-26.361	100.8969	-2.35229	0.022831	0.999016	-4.59492
PPFIA2	-15.2193	69.71951	-2.35109	0.022897	0.999016	-4.59492
ANKAR	-10.0444	44.02417	-2.3494	0.02299	0.999016	-4.59492

NKX6-3	-37.6027	151.7175	-2.34727	0.023108	0.999016	-4.59492
GS1-279B7	-31.5575	88.29428	-2.34718	0.023113	0.999016	-4.59492
MICU2	-1211.98	8344.11	-2.34447	0.023263	0.999016	-4.59492
LOC10272	14.6883	26.34679	2.341491	0.02343	0.999016	-4.59492
CDHR1	-16.3673	38.75989	-2.34069	0.023475	0.999016	-4.59492
RP5-855D2	-18.5	46.1593	-2.34055	0.023483	0.999016	-4.59492
OPTC	-32.8664	141.5395	-2.3398	0.023525	0.999016	-4.59492
RABGEF1	-66.5921	270.459	-2.33909	0.023565	0.999016	-4.59492
LOC64365	-14.2703	14.6155	-2.3384	0.023604	0.999016	-4.59492
ATF6B	-20.6501	90.23036	-2.33636	0.02372	0.999016	-4.59492
ZNF169	-17.4105	73.27052	-2.3355	0.023768	0.999016	-4.59492
SNX29	-19.8012	100.5149	-2.33423	0.02384	0.999016	-4.59492
LOC28550	-14.54	26.69953	-2.33387	0.023861	0.999016	-4.59492
TMEM262	-6.99847	13.3098	-2.32937	0.024119	0.999016	-4.59492
IL20	-18.8346	44.60032	-2.32891	0.024146	0.999016	-4.59492
ZNF583	-21.8248	110.5902	-2.32471	0.024389	0.999016	-4.59492
LOC10050	-77.0348	390.3983	-2.3231	0.024483	0.999016	-4.59492
PPY	-14.8098	26.10526	-2.32304	0.024486	0.999016	-4.59492
LOC10050	-27.1002	90.15587	-2.32244	0.024521	0.999016	-4.59492
ZFHX2	-31.0027	160.5079	-2.32225	0.024532	0.999016	-4.59492
MYH15	-16.9002	58.71157	-2.32185	0.024556	0.999016	-4.59492
EFR3B	-20.1413	114.2129	-2.32087	0.024613	0.999016	-4.59492
FRMD7	-38.1785	133.5765	-2.32056	0.024631	0.999016	-4.59492
NBPF8	-13.1521	42.35002	-2.31987	0.024672	0.999016	-4.59492
RP11-102M	-21.684	43.93555	-2.31684	0.02485	0.999016	-4.59492
USP2-AS1	-34.9876	82.90303	-2.31657	0.024866	0.999016	-4.59492
BC037861	-24.0053	88.50107	-2.31476	0.024974	0.999016	-4.59492
EVX1	-15.7879	84.64106	-2.30574	0.025515	0.999016	-4.59493
GLTPD2	-59.8581	111.5867	-2.30548	0.025531	0.999016	-4.59493
LOC10192	-11.6962	20.83497	-2.3029	0.025687	0.999016	-4.59493
KRTAP4-6	-26.1738	42.50504	-2.30285	0.02569	0.999016	-4.59493
TCL1A	23.0734	68.81839	2.302058	0.025739	0.999016	-4.59493
LEMD1	-22.4976	93.89129	-2.30171	0.02576	0.999016	-4.59493
SNORD116	-29.1326	72.08899	-2.30002	0.025863	0.999016	-4.59493
COX3	-1971.08	9495.46	-2.29886	0.025934	0.999016	-4.59493
GPR34	189.0767	748.5625	2.296841	0.026059	0.999016	-4.59493
ZSCAN10	-14.1992	36.34598	-2.29667	0.02607	0.999016	-4.59493
CARD11	-11.9548	39.4318	-2.29627	0.026094	0.999016	-4.59493
PCNXL3	12.46938	22.47208	2.294872	0.026181	0.999016	-4.59493
LOC10192	-7.03031	9.592128	-2.29468	0.026192	0.999016	-4.59493
LOC10050	-17.5697	36.9316	-2.29322	0.026283	0.999016	-4.59493
DNAJC30	-41.4499	263.9713	-2.29149	0.026391	0.999016	-4.59493
RP11-391M	-118.83	463.9415	-2.29091	0.026427	0.999016	-4.59493
ORAOV1	-24.2358	136.5283	-2.29083	0.026432	0.999016	-4.59493
KIAA2026	-62.3313	412.6609	-2.29009	0.026479	0.999016	-4.59493
LINC00960	34.14606	99.59673	2.289687	0.026504	0.999016	-4.59493
SHC3	-30.0074	124.2904	-2.28812	0.026602	0.999016	-4.59493
VWCE	26.17472	73.02907	2.28367	0.026883	0.999016	-4.59493
LOC44079	-14.5849	47.46847	-2.28151	0.02702	0.999016	-4.59493
RP11-664D	-25.9764	78.04789	-2.28053	0.027083	0.999016	-4.59493
RP11-410L	-101.899	466.1866	-2.2781	0.027238	0.999016	-4.59493
LOC10272	13.67793	32.91807	2.27705	0.027306	0.999016	-4.59493
RP11-114H	-27.2162	81.71753	-2.27674	0.027326	0.999016	-4.59493
LOC10192	-22.8096	55.52792	-2.27561	0.027399	0.999016	-4.59493
STEAP3	-73.09	202.2216	-2.27558	0.027401	0.999016	-4.59493
PRDM12	-43.1574	119.1464	-2.27418	0.027491	0.999016	-4.59493
RP5-894A1	13.49744	22.38859	2.273566	0.027531	0.999016	-4.59493
LINC01023	-35.8267	140.1082	-2.27311	0.027561	0.999016	-4.59493

KIAA0101	52.42334	258.1121	2.273015	0.027567	0.999016	-4.59493
ZNF324B	-18.5275	67.21895	-2.27299	0.027568	0.999016	-4.59493
ZNF625	-44.3393	212.5235	-2.2711	0.027691	0.999016	-4.59493
RPS6KA3	-194.229	1099.572	-2.27083	0.027709	0.999016	-4.59493
NAT16	-47.4558	133.3327	-2.27059	0.027724	0.999016	-4.59493
SIK3	-50.1444	390.2119	-2.26932	0.027807	0.999016	-4.59493
TNFRSF4	-11.0804	30.80096	-2.26852	0.02786	0.999016	-4.59493
KRTAP19-3	-12.0684	27.20346	-2.26729	0.02794	0.999016	-4.59493
CLDN18	-12.2428	41.72194	-2.26672	0.027977	0.999016	-4.59493
GBP3	670.1624	1722.945	2.266057	0.028021	0.999016	-4.59493
TRAV8-3	-17.4467	56.9832	-2.26382	0.028169	0.999016	-4.59493
ERICH1	-46.1316	248.4192	-2.26297	0.028225	0.999016	-4.59493
NCK1-AS1	-35.5572	175.5776	-2.26184	0.0283	0.999016	-4.59493
TCEANC	-17.3717	96.76827	-2.26168	0.028311	0.999016	-4.59493
RP11-115A	-21.2713	46.80726	-2.25891	0.028495	0.999016	-4.59493
CTU2	-24.6571	129.7223	-2.25873	0.028507	0.999016	-4.59493
RP11-38P2	-47.0513	163.6705	-2.2574	0.028596	0.999016	-4.59493
IL24	-19.459	44.7747	-2.25607	0.028685	0.999016	-4.59494
RP13-436F	-20.8177	49.76061	-2.25592	0.028696	0.999016	-4.59494
LOC10192	-11.3726	32.1549	-2.25562	0.028716	0.999016	-4.59494
TSG101	-774.676	5313.547	-2.25522	0.028742	0.999016	-4.59494
ASAH2B	-32.2236	212.1737	-2.25237	0.028935	0.999016	-4.59494
RP11-385F	-33.0891	136.1689	-2.25227	0.028942	0.999016	-4.59494
LOC10013	-31.8746	126.763	-2.25198	0.028962	0.999016	-4.59494
GFM1	-262.506	1950.563	-2.2515	0.028994	0.999016	-4.59494
LINC0061C	13.86288	26.25306	2.25025	0.029079	0.999016	-4.59494
LOC10099	46.6621	230.93	2.248462	0.029201	0.999016	-4.59494
PPAP2C	42.98429	110.502	2.247827	0.029244	0.999016	-4.59494
SLC9C2	-10.5911	42.31218	-2.24587	0.029378	0.999016	-4.59494
FGF4	-23.3281	90.06902	-2.24566	0.029393	0.999016	-4.59494
LOC10192	-5.83508	12.98896	-2.24542	0.02941	0.999016	-4.59494
CAPN10-A	-19.5221	75.36298	-2.24186	0.029655	0.999016	-4.59494
LOC10192	-32.746	92.27668	-2.24162	0.029672	0.999016	-4.59494
NPVF	-11.6667	19.54434	-2.24129	0.029694	0.999016	-4.59494
LINC01222	-16.3687	44.4854	-2.23692	0.029998	0.999016	-4.59494
C2orf16	-31.5351	121.7393	-2.23671	0.030013	0.999016	-4.59494
KCNN2	-114.035	298.2306	-2.23516	0.030122	0.999016	-4.59494
GRTP1-AS	-6.31262	7.97287	-2.23418	0.030191	0.999016	-4.59494
LOC10028	-7.97785	21.48855	-2.23367	0.030227	0.999016	-4.59494
LINC01281	-8.25375	12.12397	-2.23348	0.03024	0.999016	-4.59494
MEF2D	-42.1812	185.2586	-2.23295	0.030278	0.999016	-4.59494
GPR112	-12.4367	20.31563	-2.22987	0.030495	0.999016	-4.59494
MZT2B	-62.4947	433.2447	-2.22843	0.030597	0.999016	-4.59494
CEMIP	16.07852	35.52339	2.226905	0.030706	0.999016	-4.59494
RP6-91H8.	-13.5462	39.21682	-2.2246	0.030872	0.999016	-4.59494
PMM2	62.90476	300.3201	2.223047	0.030983	0.999016	-4.59494
FCGR2A	26.167	113.7598	2.221801	0.031073	0.999016	-4.59494
AX747031	-13.7388	36.75262	-2.22088	0.031139	0.999016	-4.59494
FLG	-7.53391	17.49153	-2.22077	0.031147	0.999016	-4.59494
RSG1	-33.4978	216.7808	-2.22001	0.031202	0.999016	-4.59494
SLMO1	19.71768	49.90597	2.217334	0.031397	0.999016	-4.59494
ITGB6	296.8197	792.4612	2.211779	0.031803	0.999016	-4.59494
OSMR	105.1191	496.5308	2.211777	0.031803	0.999016	-4.59494
MS4A4A	182.8538	533.7856	2.210065	0.03193	0.999016	-4.59494
CMC4	-20.5997	55.80842	-2.20989	0.031943	0.999016	-4.59494
STPG2	-30.9376	99.33267	-2.20931	0.031986	0.999016	-4.59494
HLA-DPB1	1225.418	3212.141	2.208492	0.032046	0.999016	-4.59494
ADAM3B	-9.6793	19.29146	-2.20697	0.032159	0.999016	-4.59494

GS1-164F2	-29.7779	105.5576	-2.20629	0.03221	0.999016	-4.59494
NNAT	-32.7762	95.51243	-2.20566	0.032257	0.999016	-4.59494
CCDC152	-170.798	991.8894	-2.20489	0.032314	0.999016	-4.59494
PABPC1P1	-42.0801	130.9146	-2.20403	0.032379	0.999016	-4.59494
LINC00572	-17.2874	66.22836	-2.20348	0.03242	0.999016	-4.59494
GNRH2	-16.6185	38.0179	-2.20344	0.032423	0.999016	-4.59494
VPS13A-A	-18.4825	46.95945	-2.2033	0.032433	0.999016	-4.59494
ATN1	-16.0827	89.08095	-2.2029	0.032463	0.999016	-4.59495
ZNF428	-15.9531	54.14934	-2.2023	0.032509	0.999016	-4.59495
ZFAND6	-534.861	3822.185	-2.20138	0.032577	0.999016	-4.59495
XDH	-27.3076	106.416	-2.19998	0.032683	0.999016	-4.59495
VPS9D1-A	-32.7937	106.2756	-2.19935	0.03273	0.999016	-4.59495
WFDC2	351.5735	956.0483	2.199251	0.032738	0.999016	-4.59495
NRG2	-13.1137	47.60323	-2.19858	0.032789	0.999016	-4.59495
DEFB108B	-8.41746	9.938004	-2.19824	0.032815	0.999016	-4.59495
ANKMY1	-11.3532	42.77997	-2.19706	0.032904	0.999016	-4.59495
LOC10192	9.760678	22.96549	2.195875	0.032994	0.999016	-4.59495
PRKAA2	-210.61	1078.331	-2.19567	0.03301	0.999016	-4.59495
TEX40	-54.4617	167.3817	-2.19472	0.033082	0.999016	-4.59495
GPT2	-214.691	727.8316	-2.18961	0.033474	0.999016	-4.59495
GPR83	-28.3821	67.28078	-2.18731	0.033651	0.999016	-4.59495
HIST1H4I	-23.7476	73.14314	-2.18655	0.03371	0.999016	-4.59495
HIST1H2BI	-15.7625	45.01183	-2.18396	0.033911	0.999016	-4.59495
SLCO4A1-	-26.2982	77.5182	-2.18126	0.034123	0.999016	-4.59495
LOC10192	-22.5171	61.54834	-2.17856	0.034334	0.999016	-4.59495
CHRDL2	-12.1369	28.50165	-2.17836	0.03435	0.999016	-4.59495
FOXA1	-7.58683	18.51877	-2.17689	0.034467	0.999016	-4.59495
C11orf74	-159.664	834.0212	-2.1759	0.034545	0.999016	-4.59495
EPHA1-AS	-10.3374	32.68125	-2.17502	0.034615	0.999016	-4.59495
NPHP1	-22.9698	109.9306	-2.1745	0.034656	0.999016	-4.59495
TMEM155	-16.8162	38.4584	-2.17435	0.034668	0.999016	-4.59495
ZNF132	-17.7008	102.938	-2.17335	0.034748	0.999016	-4.59495
RHOT1	-174.577	1224.709	-2.17153	0.034892	0.999016	-4.59495
CEBPZOS	-39.1934	253.5327	-2.17112	0.034926	0.999016	-4.59495
NAALAD2	-9.2873	48.95844	-2.17095	0.03494	0.999016	-4.59495
LOC64307	-24.1623	131.3994	-2.16964	0.035044	0.999016	-4.59495
STX1B	-10.2581	31.85342	-2.16934	0.035068	0.999016	-4.59495
EFCAB3	5.374274	8.122737	2.168001	0.035176	0.999016	-4.59495
MAGOH2	-24.1979	149.4721	-2.16542	0.035384	0.999016	-4.59495
KIAA0319	-9.26592	24.39026	-2.165	0.035418	0.999016	-4.59495
CCT8L2	-15.0792	26.22514	-2.16421	0.035483	0.999016	-4.59495
PRDM8	-5.13688	12.29226	-2.16306	0.035576	0.999016	-4.59495
DGCR7	-8.65852	31.01539	-2.1628	0.035597	0.999016	-4.59495
VSIG4	176.3711	693.8659	2.16098	0.035745	0.999016	-4.59495
BC037214	-14.0146	26.90659	-2.16071	0.035767	0.999016	-4.59495
NEK10	-21.6313	74.35632	-2.16048	0.035786	0.999016	-4.59495
RFESD	-27.6968	138.6565	-2.15883	0.035921	0.999016	-4.59495
BARX1-AS	-11.691	25.86138	-2.15663	0.036102	0.999016	-4.59495
CTD-3203I	-14.2427	29.4599	-2.15573	0.036176	0.999016	-4.59495
PLD5	-14.2127	52.55438	-2.15409	0.036312	0.999016	-4.59495
RS1	-10.5306	37.44666	-2.15253	0.03644	0.999016	-4.59495
PSORS1C2	-8.62667	29.77475	-2.14939	0.036702	0.999016	-4.59495
ERMARD	-134.094	985.2704	-2.1475	0.036861	0.999016	-4.59496
LOC10192	-13.1865	29.73753	-2.14609	0.036979	0.999016	-4.59496
DPP9	-34.6097	169.561	-2.14581	0.037003	0.999016	-4.59496
CSMD3	-10.7922	33.58525	-2.14437	0.037124	0.999016	-4.59496
MIR6834	-78.5707	399.1272	-2.14312	0.03723	0.999016	-4.59496
RP11-769C	-15.0007	55.3409	-2.14232	0.037297	0.999016	-4.59496

LINC00652	-13.657	27.7179	-2.14222	0.037306	0.999016	-4.59496
NKX3-1	-27.6924	128.762	-2.14162	0.037356	0.999016	-4.59496
DLX3	-15.1844	28.16004	-2.14148	0.037369	0.999016	-4.59496
FGF6	-30.818	73.21844	-2.14115	0.037397	0.999016	-4.59496
FAM209B	-20.6752	101.4988	-2.14044	0.037457	0.999016	-4.59496
SLC25A2	-13.6637	48.42635	-2.13707	0.037745	0.999016	-4.59496
UBBP1	-381.582	1953.638	-2.1354	0.037888	0.999016	-4.59496
TPTEP1	-25.2451	139.7971	-2.13519	0.037906	0.999016	-4.59496
LOC10192	8.258715	14.18271	2.133182	0.038079	0.999016	-4.59496
VMP1	82.47041	303.4495	2.133155	0.038081	0.999016	-4.59496
LOC10192	-90.7832	383.1477	-2.13244	0.038143	0.999016	-4.59496
MBD3L2	6.029723	12.66658	2.13197	0.038184	0.999016	-4.59496
CLPX	-176.061	1345.087	-2.1315	0.038224	0.999016	-4.59496
BC042022	-9.40923	23.29403	-2.12985	0.038367	0.999016	-4.59496
C10orf113	-17.179	27.59252	-2.12935	0.038411	0.999016	-4.59496
REG3G	17.22315	51.4322	2.128476	0.038487	0.999016	-4.59496
RP11-334(	31.85064	140.7381	2.127679	0.038557	0.999016	-4.59496
MISP	116.2783	388.7865	2.125491	0.038748	0.999016	-4.59496
LINC00599	-10.9682	40.33918	-2.12442	0.038842	0.999016	-4.59496
FLJ38668	-27.1271	116.9803	-2.12399	0.03888	0.999016	-4.59496
ATP5S	-48.4516	282.7621	-2.1233	0.03894	0.999016	-4.59496
SFN	60.67745	292.6317	2.122574	0.039004	0.999016	-4.59496
CPNE7	-46.3584	186.8871	-2.12115	0.03913	0.999016	-4.59496
UGGT1	-64.3844	472.9783	-2.12113	0.039132	0.999016	-4.59496
PDAP1	-20.7491	96.86171	-2.11938	0.039286	0.999016	-4.59496
RP11-203E	-11.562	22.47979	-2.11869	0.039348	0.999016	-4.59496
GOLGA8CI	-65.8659	395.1359	-2.11855	0.03936	0.999016	-4.59496
LOC10192	-11.7363	26.38872	-2.11813	0.039398	0.999016	-4.59496
KNOP1	-34.2493	239.8059	-2.11805	0.039405	0.999016	-4.59496
CHRFAM7/	21.57012	52.60623	2.118048	0.039405	0.999016	-4.59496
CHAMP1	-37.9171	316.233	-2.11792	0.039417	0.999016	-4.59496
TEX41	-14.6173	45.20852	-2.11749	0.039454	0.999016	-4.59496
DDN	-152.788	253.4016	-2.11717	0.039483	0.999016	-4.59496
CFB	112.5014	442.9338	2.115998	0.039588	0.999016	-4.59496
NDUFAF4	-205.655	1321.913	-2.11585	0.039601	0.999016	-4.59496
WI2-89031	-9.17231	18.52558	-2.11504	0.039674	0.999016	-4.59496
LOC10013	-10.358	36.31146	-2.11368	0.039795	0.999016	-4.59496
NXPH3	-28.7821	132.8728	-2.11352	0.039809	0.999016	-4.59496
KRT78	-8.27224	24.85172	-2.11338	0.039822	0.999016	-4.59496
ELAVL3	-5.22628	15.7816	-2.1117	0.039973	0.999016	-4.59496
LOC10192	-8.0079	31.81648	-2.11134	0.040006	0.999016	-4.59496
LOC10050(	8.100327	16.18083	2.110696	0.040064	0.999016	-4.59496
ZNF595	-68.2458	98.31985	-2.11015	0.040113	0.999016	-4.59496
C1QC	102.5785	325.9824	2.109804	0.040144	0.999016	-4.59496
MTRF1	-28.6853	214.9408	-2.10891	0.040226	0.999016	-4.59496
LIPI	7.889029	20.52239	2.108723	0.040242	0.999016	-4.59496
SHISA2	-84.0845	379.1415	-2.1087	0.040245	0.999016	-4.59496
LARP4B	-41.8304	336.3588	-2.10839	0.040272	0.999016	-4.59496
LOC10272	-12.0847	26.10706	-2.10776	0.04033	0.999016	-4.59496
RP11-255(	-15.7331	80.87969	-2.1039	0.040681	0.999016	-4.59496
CTNNA2	-10.8783	28.61399	-2.10335	0.040732	0.999016	-4.59496
UBQLN3	-12.4683	23.20491	-2.10269	0.040792	0.999016	-4.59496
BPIFB9P	-17.43	42.33038	-2.10226	0.040831	0.999016	-4.59496
ACTR3B	-43.1229	296.3974	-2.10219	0.040838	0.999016	-4.59496
LOC33987	-11.2883	45.74674	-2.10192	0.040863	0.999016	-4.59496
RP11-359k	7.277704	12.80685	2.1018	0.040874	0.999016	-4.59496
BC040311	-5.95592	9.719448	-2.10111	0.040937	0.999016	-4.59496
PF4V1	-13.6322	58.01229	-2.10069	0.040975	0.999016	-4.59496

SPAM1	-12.4525	46.87129	-2.1006	0.040984	0.999016	-4.59496
FAM57A	47.74656	196.9578	2.100537	0.04099	0.999016	-4.59496
LOC64262(	-8.78738	14.38269	-2.09981	0.041057	0.999016	-4.59496
FAM27E2	-29.5332	158.7025	-2.09973	0.041064	0.999016	-4.59496
ZNF718	26.08586	100.6349	2.098119	0.041213	0.999016	-4.59496
C1orf145	-10.4332	35.12583	-2.0974	0.04128	0.999016	-4.59496
LRIG1	-27.5581	171.5806	-2.09595	0.041414	0.999016	-4.59496
LOC10193(	-14.8104	55.85691	-2.09441	0.041558	0.999016	-4.59496
AIMP2	-368.821	2128.722	-2.09385	0.04161	0.999016	-4.59496
HBZ	-7.97029	14.5471	-2.0919	0.041791	0.999016	-4.59497
PIH2	6.825511	18.22229	2.091415	0.041837	0.999016	-4.59497
OIT3	-11.6394	30.51905	-2.09122	0.041856	0.999016	-4.59497
ST3GAL3	-21.2668	123.6356	-2.09104	0.041872	0.999016	-4.59497
PCDHB18	-11.3826	35.47693	-2.09068	0.041906	0.999016	-4.59497
LOC10192ε	6.606328	7.868341	2.090653	0.041909	0.999016	-4.59497
KRTAP2-1	5.903143	8.128912	2.08968	0.042	0.999016	-4.59497
LOC28635!	-18.0184	76.2764	-2.08671	0.04228	0.999016	-4.59497
ARHGEF37	-55.6942	372.1733	-2.08671	0.04228	0.999016	-4.59497
OR4D2	-22.7038	67.95607	-2.08442	0.042497	0.999016	-4.59497
C21orf91-(	-11.2594	43.47918	-2.08373	0.042563	0.999016	-4.59497
SSU72	-134.784	1062.552	-2.08333	0.042601	0.999016	-4.59497
CDNF	-33.3349	105.0825	-2.08309	0.042623	0.999016	-4.59497
GRM7	-11.7912	35.87608	-2.0804	0.04288	0.999016	-4.59497
AX746699	-10.7742	22.27707	-2.07952	0.042965	0.999016	-4.59497
PCBD2	-61.6773	429.0792	-2.07937	0.042979	0.999016	-4.59497
BOLL	-21.3854	75.0534	-2.07852	0.043061	0.999016	-4.59497
RP11-731J	-9.15202	36.22632	-2.07727	0.043181	0.999016	-4.59497
PLCL2	-143.947	1015.565	-2.07671	0.043235	0.999016	-4.59497
SNCB	-8.08603	20.6596	-2.0747	0.04343	0.999016	-4.59497
MRPL30	-117.675	917.5907	-2.07418	0.04348	0.999016	-4.59497
BTBD18	-24.4704	107.5057	-2.07365	0.043532	0.999016	-4.59497
LOC55310:	-35.1588	195.2573	-2.07297	0.043598	0.999016	-4.59497
RP11-489E	-13.0488	56.36056	-2.07176	0.043715	0.999016	-4.59497
LINC01339	-26.7601	78.84137	-2.07169	0.043722	0.999016	-4.59497
ZSCAN20	-12.1004	71.08058	-2.07167	0.043724	0.999016	-4.59497
ISOC1	-885.816	5370.868	-2.06866	0.044017	0.999016	-4.59497
C12orf80	-14.8419	55.54936	-2.06863	0.044021	0.999016	-4.59497
TRIML2	-6.20587	8.741109	-2.0684	0.044043	0.999016	-4.59497
IMMP1L	-128.041	900.6386	-2.06713	0.044168	0.999016	-4.59497
LOC10192ε	-22.1791	86.34621	-2.06625	0.044254	0.999016	-4.59497
CTD-2035I	-16.8912	50.4694	-2.06611	0.044268	0.999016	-4.59497
LOC10192ε	-16.8343	37.04153	-2.06533	0.044345	0.999016	-4.59497
GNB1L	-18.142	75.22807	-2.06473	0.044404	0.999016	-4.59497
ADAMTS1!	-6.27391	14.76501	-2.06342	0.044534	0.999016	-4.59497
RP4-798A1	-42.4182	215.5622	-2.06337	0.044539	0.999016	-4.59497
SLC17A8	15.73018	20.5585	2.062235	0.044651	0.999016	-4.59497
LOC28348	-8.5475	21.85248	-2.06169	0.044705	0.999016	-4.59497
MEG8	-8.5241	15.17601	-2.06149	0.044725	0.999016	-4.59497
LTF	1077.139	1005.069	2.061476	0.044727	0.999016	-4.59497
LOC25489(	-21.3862	78.66156	-2.06127	0.044748	0.999016	-4.59497
LSM14B	-13.4502	80.22399	-2.06081	0.044792	0.999016	-4.59497
CCDC89	14.76318	64.27994	2.060765	0.044797	0.999016	-4.59497
PRO1483	7.254376	12.59659	2.060658	0.044808	0.999016	-4.59497
NOP58	-348.982	2630.944	-2.06058	0.044816	0.999016	-4.59497
PRAMEF10	-23.9378	53.20705	-2.05998	0.044876	0.999016	-4.59497
LINC00115	-16.2908	90.02246	-2.05975	0.044898	0.999016	-4.59497
PDCD5	-39.5839	278.1812	-2.05902	0.044971	0.999016	-4.59497
PITPNM3	-22.6898	119.5903	-2.05782	0.045091	0.999016	-4.59497

PTPRU	-62.8245	267.6208	-2.05775	0.045098	0.999016	-4.59497
FSIP1	12.34836	38.13124	2.057584	0.045115	0.999016	-4.59497
NDUFB6	-527.95	2684.235	-2.05747	0.045126	0.999016	-4.59497
ZNF354B	-51.5935	249.6726	-2.05531	0.045343	0.999016	-4.59497
NPHP3-AS	-11.2357	32.62829	-2.05228	0.045648	0.999016	-4.59497
KY	-32.7375	72.79133	-2.05215	0.045662	0.999016	-4.59497
TIMM8A	-41.0228	222.4939	-2.05204	0.045673	0.999016	-4.59497
DPCR1	-9.17176	29.09051	-2.05154	0.045723	0.999016	-4.59497
ABHD17B	-118.917	855.9923	-2.0511	0.045768	0.999016	-4.59497
LOC10192	-10.0162	14.00285	-2.05109	0.045769	0.999016	-4.59497
ATP5J	-2226.44	15134.68	-2.05077	0.045802	0.999016	-4.59497
FAM221A	-60.9315	308.3719	-2.04952	0.045928	0.999016	-4.59497
SFRP1	523.9286	2619.97	2.049168	0.045964	0.999016	-4.59497
LOC10192	-21.615	66.27353	-2.04836	0.046046	0.999016	-4.59497
LOC10272	-20.7474	40.2712	-2.04803	0.04608	0.999016	-4.59497
RP11-167H	-17.9344	40.93475	-2.0476	0.046124	0.999016	-4.59497
LINC00673	-13.0465	26.02674	-2.04711	0.046174	0.999016	-4.59497
NFE4	-10.7316	21.88654	-2.04629	0.046257	0.999016	-4.59497
LOC28576	-14.6895	48.99688	-2.04563	0.046325	0.999016	-4.59497
AC083949.	-21.5433	72.37815	-2.04457	0.046434	0.999016	-4.59497
LOC10192	-14.4089	27.29844	-2.04429	0.046463	0.999016	-4.59497
FIBCD1	-8.92509	27.36742	-2.04412	0.04648	0.999016	-4.59497
KRTAP5-2	-18.2433	50.9765	-2.044	0.046492	0.999016	-4.59497
CCL24	-16.8053	34.38346	-2.04387	0.046505	0.999016	-4.59497
SLC34A2	133.542	348.1631	2.042808	0.046615	0.999016	-4.59497
CFLAR	-83.387	716.6289	-2.0427	0.046626	0.999016	-4.59497
RP11-195M	-27.8782	118.5887	-2.04269	0.046627	0.999016	-4.59497
SPRYD7	-47.5511	259.4328	-2.04247	0.04665	0.999016	-4.59497
LOC10192	-12.1949	36.51449	-2.04242	0.046655	0.999016	-4.59497
ASMTL-AS	-14.1517	84.47016	-2.04155	0.046745	0.999016	-4.59497
LOC28608	8.751507	20.16817	2.041453	0.046755	0.999016	-4.59497
SPG20	-132.771	972.4857	-2.04124	0.046777	0.999016	-4.59497
RLTPR	13.35625	44.82716	2.03986	0.046919	0.999016	-4.59497
DNAJC9-A	-32.1691	130.9254	-2.03884	0.047025	0.999016	-4.59497
SELP	142.5924	630.7433	2.038007	0.047112	0.999016	-4.59497
LRRC4C	-14.6635	64.31855	-2.03754	0.047161	0.999016	-4.59497
CECR5-AS	12.32913	30.95826	2.036945	0.047222	0.999016	-4.59498
CHEK1	-22.0506	159.9965	-2.03584	0.047337	0.999016	-4.59498
PCNXL4	-38.4475	295.1132	-2.03462	0.047464	0.999016	-4.59498
T-18	-11.2308	38.71188	-2.03432	0.047496	0.999016	-4.59498
INTS2	-51.6657	310.9425	-2.03412	0.047517	0.999016	-4.59498
LOC10050	-20.8312	76.98359	-2.03254	0.047683	0.999016	-4.59498
FUBP3	-136.747	854.1586	-2.03079	0.047867	0.999016	-4.59498
EGF	-1696	4737.993	-2.02798	0.048163	0.999016	-4.59498
ASH2L	-295.301	1854.277	-2.02697	0.048271	0.999016	-4.59498
FAM205A	-8.34039	20.29565	-2.02542	0.048435	0.999016	-4.59498
CPEB4	-279.827	1698.378	-2.02524	0.048455	0.999016	-4.59498
SIGLEC16	-11.3455	21.41081	-2.02524	0.048455	0.999016	-4.59498
GS1-24F4.	-12.6719	24.54733	-2.02486	0.048495	0.999016	-4.59498
C19orf73	-24.2638	55.82575	-2.02471	0.048511	0.999016	-4.59498
BC042825	-7.27916	12.8525	-2.02425	0.048561	0.999016	-4.59498
ALS2CL	-50.9604	295.1632	-2.02399	0.048588	0.999016	-4.59498
SRMP1	23.96854	55.79644	2.023729	0.048616	0.999016	-4.59498
ESCO2	-6.51486	27.42492	-2.02338	0.048653	0.999016	-4.59498
PRND	-13.5014	45.50118	-2.0228	0.048715	0.999016	-4.59498
RP3-337H	-12.6221	39.06052	-2.02238	0.04876	0.999016	-4.59498
LINC00086	-276.224	1720.849	-2.0222	0.04878	0.999016	-4.59498
MTCL1	85.99695	461.7978	2.021597	0.048844	0.999016	-4.59498

LOC28466[illegible]	-9.47091	42.62485	-2.02125	0.048881	0.999016	-4.59498
PDX1	-6.36787	23.31242	-2.02096	0.048913	0.999016	-4.59498
PLGLB1	-50.9346	272.6725	-2.02046	0.048966	0.999016	-4.59498
TOP2A	10.56155	33.73787	2.020286	0.048985	0.999016	-4.59498
FSTL5	-13.6245	20.98028	-2.0199	0.049026	0.999016	-4.59498
AGR2	47.8039	151.0285	2.019652	0.049053	0.999016	-4.59498
BC043223	-14.4756	36.40796	-2.01883	0.049142	0.999016	-4.59498
MYC	71.36436	256.085	2.018541	0.049173	0.999016	-4.59498
CYP4F62P	-13.1934	21.86377	-2.0181	0.049221	0.999016	-4.59498
OR2S2	-17.5739	64.09716	-2.01737	0.049299	0.999016	-4.59498
LOC64308[illegible]	-19.9796	70.30488	-2.01652	0.049391	0.999016	-4.59498
CFTR	6.899075	22.7315	2.015777	0.049472	0.999016	-4.59498
GRIP2	-91.8463	271.7164	-2.01552	0.0495	0.999016	-4.59498
STRIP2	-53.0096	117.8941	-2.01544	0.049508	0.999016	-4.59498
DMBT1	-50.5416	232.1294	-2.0148	0.049578	0.999016	-4.59498
SLC19A2	-463.485	1925.718	-2.0147	0.049588	0.999016	-4.59498
PRR34-AS[illegible]	-389.514	1811.727	-2.01377	0.04969	0.999016	-4.59498
ADORA1	-22.5853	116.6643	-2.01353	0.049716	0.999016	-4.59498
TRAPPC1	104.5071	482.0578	2.01209	0.049873	0.999016	-4.59498
TMEM196	5.881105	17.29268	2.011602	0.049926	0.999016	-4.59498
AX747444	-13.3527	40.22705	-2.01153	0.049934	0.999016	-4.59498
