# Supplementary material for: The Potential Diagnostic Value of Immune-Related Genes in Interstitial Fibrosis and Tubular Atrophy after Kidney Transplantation
Source: J Immunol Res. 2022 Jun 17;2022:7212852. doi: 10.1155/2022/7212852 (PMC9232312; doi:10.1155/2022/7212852)
Supplement: Supplementary Materials — Supplementary Figure 1: GSEA enrichment analysis of the IF/TA group. Supplementary Figure 2: correlation analysis between ANGPTL3 and differentially expressed immune infiltrating cells. Supplementary Figure 3: correlation analysis between APOH and differentially expressed immune infiltrating cells. Supplementary Figure 4: correlation analysis between EGF and differentially expressed immune infiltrating cells. Supplementary Figure 5: correlation analysis between FCGR2B and differentially expressed immune infiltrating cells. Supplementary Figure 6: correlation analysis between HLA-DQA2 and differentially expressed immune infiltrating cells. Supplementary Figure 7: correlation analysis between LTF and differentially expressed immune infiltrating cells. Supplementary Figure 8: IPA analysis shows the interaction network of diagnostic genes: EGF and LTF (8A), ANGPTL3 (8B), FCGR2B and APOH (8C), and HLA-DQA2 (8D). Merged the above four independent networks to comprehensively analyze the interaction of diagnostic genes (8E). Supplementary Table 1: immune-related genes. Supplementary Table 2: KEGG pathway in normal group. Supplementary Table 3: pathway of ANGPTL3 gene. Supplementary Table 4: pathway of APOH gene. Supplementary Table 5: pathway of EGF gene. Supplementary Table 6: ingenuity canonical pathways. Supplementary Table 7: category. [file 7212852.f1.zip › 7212852.f1/supplementary table4.pdf]

NAME	GS<br>follow link to MSigDB	GS DETAIL
KEGG ANT	KEGG ANTIGEN PROCESSING AND PRESENTATION	Details ...
KEGG AUT	KEGG AUTOIMMUNE THYROID DISEASE	Details ...
KEGG TOL	KEGG TOLL LIKE RECEPTOR SIGNALING PATHWAY	Details ...
KEGG NAT	KEGG NATURAL KILLER CELL MEDIATED CYTOTOXICITY	Details ...
KEGG CEL	KEGG CELL ADHESION MOLECULES CAMS	Details ...
KEGG CYT	KEGG CYTOSOLIC DNA SENSING PATHWAY	Details ...
KEGG CHE	KEGG CHEMOKINE SIGNALING PATHWAY	Details ...
KEGG NOD	KEGG NOD LIKE RECEPTOR SIGNALING PATHWAY	Details ...
KEGG T CE	KEGG T CELL RECEPTOR SIGNALING PATHWAY	Details ...
KEGG CYT	KEGG CYTOKINE CYTOKINE RECEPTOR INTERACTION	Details ...
KEGG B CE	KEGG B CELL RECEPTOR SIGNALING PATHWAY	Details ...
KEGG INTE	KEGG INTESTINAL IMMUNE NETWORK FOR IGA PRODUCTION	Details ...
KEGG SYS	KEGG SYSTEMIC LUPUS ERYTHEMATOSUS	Details ...
KEGG ALL	KEGG ALLOGRAFT REJECTION	Details ...
KEGG TYP	KEGG TYPE I DIABETES MELLITUS	Details ...
KEGG FC G	KEGG FC GAMMA R MEDIATED PHAGOCYTOSIS	Details ...
KEGG HEM	KEGG HEMATOPOIETIC CELL LINEAGE	Details ...
KEGG GRA	KEGG GRAFT VERSUS HOST DISEASE	Details ...
KEGG LEIS	KEGG LEISHMANIA INFECTION	Details ...
KEGG PAT	KEGG PATHOGENIC ESCHERICHIA COLI INFECTION	Details ...
KEGG PRIO	KEGG PRION DISEASES	Details ...
KEGG GLY	KEGG GLYCOSAMINOGLYCAN BIOSYNTHESIS CHONDROITIN SULFATE	Details ...
KEGG JAK S	KEGG JAK STAT SIGNALING PATHWAY	Details ...
KEGG APO	KEGG APOPTOSIS	Details ...
KEGG AST	KEGG ASTHMA	Details ...
KEGG LEU	KEGG LEUKOCYTE TRANSENDOTHELIAL MIGRATION	Details ...
KEGG PRIM	KEGG PRIMARY IMMUNODEFICIENCY	Details ...
KEGG FC E	KEGG FC EPSILON RI SIGNALING PATHWAY	Details ...
KEGG MAP	KEGG MAPK SIGNALING PATHWAY	Details ...
KEGG PAT	KEGG PATHWAYS IN CANCER	Details ...

SIZE	ES	NES	NOM p-va	FDR q-val	FWER p-va	RANK AT M	LEADING EDGE
86	0.637674	1.80744	0.01626	0.198152	0.144	2895	tags=44%, list=16%, sig
44	0.74458	1.80474	0.008147	0.101305	0.148	2555	tags=57%, list=14%, sig
98	0.607373	1.796019	0.004124	0.075449	0.159	2903	tags=34%, list=16%, sig
127	0.607632	1.766854	0.004184	0.079395	0.206	2742	tags=35%, list=15%, sig
128	0.612923	1.758741	0.004107	0.070061	0.22	2904	tags=47%, list=16%, sig
50	0.621646	1.752431	0.006466	0.063044	0.232	1804	tags=22%, list=10%, sig
175	0.643299	1.720948	0.002137	0.078175	0.289	1901	tags=34%, list=11%, sig
60	0.679667	1.718522	0.008316	0.070382	0.297	1946	tags=38%, list=11%, sig
102	0.589614	1.707745	0.008493	0.070875	0.326	2904	tags=37%, list=16%, sig
227	0.608139	1.704341	0.00211	0.066931	0.339	2153	tags=37%, list=12%, sig
74	0.558901	1.688296	0.010504	0.070246	0.368	3066	tags=38%, list=17%, sig
42	0.74136	1.687857	0.012295	0.064579	0.368	2572	tags=62%, list=15%, sig
52	0.790584	1.682236	0.004115	0.063299	0.383	1880	tags=65%, list=11%, sig
33	0.81183	1.673049	0.008163	0.06476	0.407	2571	tags=79%, list=15%, sig
41	0.717802	1.662224	0.018256	0.067585	0.434	2571	tags=63%, list=15%, sig
95	0.544154	1.653037	0.012346	0.069252	0.456	1570	tags=25%, list=9%, sign
80	0.671442	1.652822	0.004149	0.065357	0.457	1889	tags=49%, list=11%, sig
40	0.775701	1.647167	0.014706	0.065922	0.473	2571	tags=68%, list=15%, sig
70	0.731231	1.642194	0.014433	0.065617	0.486	2575	tags=60%, list=15%, sig
54	0.533335	1.638392	0.018	0.064872	0.497	1614	tags=22%, list=9%, sign
34	0.606233	1.637546	0.012422	0.062426	0.5	847	tags=24%, list=5%, sign
22	0.627394	1.634535	0.024048	0.06142	0.509	3458	tags=55%, list=20%, sig
131	0.467417	1.623149	0.014199	0.065394	0.534	2786	tags=31%, list=16%, sig
83	0.444357	1.59466	0.034765	0.081487	0.592	2807	tags=30%, list=16%, sig
23	0.812747	1.586863	0.025794	0.083455	0.603	1872	tags=78%, list=11%, sig
109	0.53842	1.584039	0.006085	0.082522	0.609	1214	tags=23%, list=7%, sign
33	0.765356	1.569317	0.021277	0.091066	0.653	1248	tags=52%, list=7%, sign
70	0.445848	1.527942	0.0375	0.121978	0.739	1570	tags=19%, list=9%, sign
245	0.343546	1.401129	0.046939	0.225204	0.911	2865	tags=25%, list=16%, sig
306	0.345007	1.398404	0.045098	0.222292	0.916	3472	tags=31%, list=20%, sig

gnal=53%
gnal=66%
gnal=40%
gnal=41%
gnal=56%
gnal=24%
gnal=37%
gnal=43%
gnal=44%
gnal=42%
gnal=46%
gnal=72%
gnal=73%
gnal=92%
gnal=74%
nal=28%
gnal=54%
gnal=79%
gnal=70%
nal=24%
nal=25%
gnal=68%
gnal=36%
gnal=36%
gnal=87%
nal=24%
nal=55%
nal=20%
gnal=29%
gnal=38%
