# Supplementary material for: The Potential Diagnostic Value of Immune-Related Genes in Interstitial Fibrosis and Tubular Atrophy after Kidney Transplantation
Source: J Immunol Res. 2022 Jun 17;2022:7212852. doi: 10.1155/2022/7212852 (PMC9232312; doi:10.1155/2022/7212852)
Supplement: Supplementary Materials — Supplementary Figure 1: GSEA enrichment analysis of the IF/TA group. Supplementary Figure 2: correlation analysis between ANGPTL3 and differentially expressed immune infiltrating cells. Supplementary Figure 3: correlation analysis between APOH and differentially expressed immune infiltrating cells. Supplementary Figure 4: correlation analysis between EGF and differentially expressed immune infiltrating cells. Supplementary Figure 5: correlation analysis between FCGR2B and differentially expressed immune infiltrating cells. Supplementary Figure 6: correlation analysis between HLA-DQA2 and differentially expressed immune infiltrating cells. Supplementary Figure 7: correlation analysis between LTF and differentially expressed immune infiltrating cells. Supplementary Figure 8: IPA analysis shows the interaction network of diagnostic genes: EGF and LTF (8A), ANGPTL3 (8B), FCGR2B and APOH (8C), and HLA-DQA2 (8D). Merged the above four independent networks to comprehensively analyze the interaction of diagnostic genes (8E). Supplementary Table 1: immune-related genes. Supplementary Table 2: KEGG pathway in normal group. Supplementary Table 3: pathway of ANGPTL3 gene. Supplementary Table 4: pathway of APOH gene. Supplementary Table 5: pathway of EGF gene. Supplementary Table 6: ingenuity canonical pathways. Supplementary Table 7: category. [file 7212852.f1.zip › 7212852.f1/supplementary table5.pdf]

NAME	GS<br>fo	GS DETAIL	SIZE	ES	NES	NOM p-va	FDR q-val	FWER p-va
KEGG OXII	KEGG OXII	Details ...	95	-0.47599	-1.90175	0.008602	0.050945	0.053
KEGG PARI	KEGG PARI	Details ...	88	-0.41383	-1.82593	0.01996	0.07391	0.127
KEGG VALI	KEGG VALI	Details ...	43	-0.66442	-1.6959	0.02449	0.21618	0.364
KEGG CITR	KEGG CITR	Details ...	29	-0.64524	-1.64753	0.032129	0.257977	0.458
KEGG PYRU	KEGG PYRU	Details ...	37	-0.57823	-1.63627	0.035052	0.226979	0.485
KEGG LYSII	KEGG LYSII	Details ...	42	-0.51681	-1.5968	0.042424	0.272186	0.591
KEGG ARG	KEGG ARG	Details ...	51	-0.56793	-1.58384	0.042339	0.262352	0.625
KEGG PRO	KEGG PRO	Details ...	28	-0.62151	-1.56635	0.046843	0.267578	0.655
KEGG HIST	KEGG HIST	Details ...	26	-0.64429	-1.56565	0.03681	0.239476	0.655
KEGG TERI	KEGG TERI	Details ...	15	-0.63185	-1.52638	0.058594	0.301051	0.747
KEGG GLY(	KEGG GLY(	Details ...	30	-0.69368	-1.51246	0.072435	0.30218	0.765
KEGG BUT/	KEGG BUT/	Details ...	31	-0.62814	-1.50771	0.066398	0.286475	0.769
KEGG GLY(	KEGG GLY(	Details ...	26	-0.51893	-1.46156	0.036822	0.37503	0.843
KEGG TYR(	KEGG TYR(	Details ...	36	-0.52326	-1.45354	0.06544	0.366268	0.856
KEGG PRO	KEGG PRO	Details ...	20	-0.62909	-1.45092	0.062753	0.347621	0.862
KEGG CYS	KEGG CYS	Details ...	32	-0.4563	-1.43381	0.07332	0.363087	0.883
KEGG AMII	KEGG AMII	Details ...	21	-0.53882	-1.41227	0.117647	0.388466	0.905
KEGG BET/	KEGG BET/	Details ...	20	-0.59901	-1.3945	0.149194	0.405966	0.924
KEGG PER(	KEGG PER(	Details ...	75	-0.47311	-1.38568	0.184676	0.404871	0.927
KEGG GLY(	KEGG GLY(	Details ...	54	-0.44851	-1.35959	0.138776	0.442679	0.94
KEGG BIOS	KEGG BIOS	Details ...	21	-0.4847	-1.35021	0.137795	0.442744	0.941
KEGG MAT	KEGG MAT	Details ...	19	-0.54201	-1.34135	0.108738	0.444408	0.945
KEGG SNA	KEGG SNA	Details ...	35	-0.41927	-1.33947	0.140845	0.429216	0.947
KEGG GLY(	KEGG GLY(	Details ...	44	-0.40736	-1.33895	0.08866	0.412226	0.948
KEGG GAL	KEGG GAL	Details ...	23	-0.45	-1.33574	0.131474	0.401619	0.948
KEGG OLF/	KEGG OLF/	Details ...	60	-0.42947	-1.30105	0.161943	0.462228	0.965
KEGG PHEI	KEGG PHEI	Details ...	17	-0.53924	-1.2851	0.174349	0.480008	0.969
KEGG AMII	KEGG AMII	Details ...	43	-0.39414	-1.28394	0.188716	0.465673	0.969
KEGG HUN	KEGG HUN	Details ...	149	-0.24054	-1.2621	0.172708	0.497078	0.974
KEGG REGI	KEGG REGI	Details ...	31	-0.34926	-1.25523	0.225673	0.493866	0.976
KEGG ALZI	KEGG ALZI	Details ...	135	-0.28248	-1.23299	0.144309	0.524726	0.982
KEGG ALAI	KEGG ALAI	Details ...	29	-0.47685	-1.19514	0.269076	0.593486	0.992
KEGG FAT	KEGG FAT	Details ...	41	-0.41157	-1.15651	0.338614	0.666054	0.997
KEGG TRYI	KEGG TRYI	Details ...	37	-0.46383	-1.15622	0.336634	0.647264	0.997
KEGG NITF	KEGG NITF	Details ...	22	-0.42294	-1.14833	0.291837	0.646094	0.997
KEGG LINC	KEGG LINC	Details ...	22	-0.47585	-1.10295	0.347015	0.733865	0.999
KEGG PPAI	KEGG PPAI	Details ...	63	-0.39639	-1.0663	0.363813	0.803466	1
KEGG TYPI	KEGG TYPI	Details ...	45	-0.2853	-1.0656	0.312621	0.784018	1
KEGG RETI	KEGG RETI	Details ...	50	-0.45565	-1.05799	0.446721	0.781876	1
KEGG GLY(	KEGG GLY(	Details ...	25	-0.32545	-1.05767	0.39839	0.763006	1
KEGG CAR	KEGG CAR	Details ...	60	-0.47129	-1.05265	0.447791	0.756141	1
KEGG FRU(	KEGG FRU(	Details ...	32	-0.33596	-1.04492	0.422311	0.756061	1
KEGG PEN	KEGG PEN	Details ...	22	-0.53943	-1.02966	0.5125	0.771867	1
KEGG INSL	KEGG INSL	Details ...	133	-0.26633	-1.01936	0.437113	0.776523	1
KEGG ASC	KEGG ASC	Details ...	19	-0.5741	-1.01521	0.533881	0.768642	1
KEGG CAL(	KEGG CAL(	Details ...	152	-0.30962	-1.00748	0.447525	0.769756	1
KEGG MISI	KEGG MISI	Details ...	23	-0.3679	-0.98629	0.501062	0.799317	1
KEGG GLY(	KEGG GLY(	Details ...	21	-0.32421	-0.98535	0.479612	0.784607	1
KEGG PEN	KEGG PEN	Details ...	25	-0.3161	-0.97882	0.484314	0.78281	1
KEGG VIBR	KEGG VIBR	Details ...	52	-0.27708	-0.97841	0.486869	0.768164	1
KEGG SELE	KEGG SELE	Details ...	23	-0.37034	-0.97396	0.514286	0.762393	1
KEGG DRU	KEGG DRU	Details ...	62	-0.39963	-0.96447	0.504219	0.767634	1
KEGG SPHI	KEGG SPHI	Details ...	35	-0.33608	-0.95139	0.516393	0.780827	1
KEGG PAN	KEGG PAN	Details ...	16	-0.39317	-0.93231	0.579158	0.804987	1
KEGG GLU	KEGG GLU	Details ...	47	-0.29684	-0.92731	0.567251	0.80055	1
KEGG ONE	KEGG ONE	Details ...	17	-0.34743	-0.92286	0.531621	0.795413	1
KEGG NOT	KEGG NOT	Details ...	46	-0.25199	-0.90399	0.580952	0.819487	1

KEGG NUC	KEGG NUC	Details ...	42	-0.28198	-0.89975	0.582441	0.81353	1
KEGG HED	KEGG HED	Details ...	49	-0.25815	-0.89495	0.631579	0.808906	1
KEGG NEU	KEGG NEU	Details ...	192	-0.26925	-0.89176	0.624528	0.801036	1
KEGG NIC(	KEGG NIC(	Details ...	22	-0.33067	-0.85907	0.648221	0.850923	1
KEGG STEF	KEGG STEF	Details ...	17	-0.34148	-0.85369	0.672584	0.847762	1
KEGG GLIC	KEGG GLIC	Details ...	64	-0.22683	-0.84484	0.727451	0.851494	1
KEGG TAS	KEGG TAS	Details ...	26	-0.28523	-0.8252	0.727092	0.873799	1
KEGG MET	KEGG MET	Details ...	58	-0.33131	-0.82431	0.66875	0.861862	1
KEGG PUR	KEGG PUR	Details ...	144	-0.18266	-0.81956	0.821138	0.856696	1
KEGG DRU	KEGG DRU	Details ...	44	-0.35394	-0.81575	0.636735	0.850124	1
KEGG STAI	KEGG STAI	Details ...	43	-0.33151	-0.8009	0.7119	0.862624	1
KEGG MEL	KEGG MEL	Details ...	65	-0.20416	-0.78895	0.84585	0.869839	1
KEGG INO:	KEGG INO:	Details ...	52	-0.20172	-0.78859	0.816456	0.85795	1
KEGG HON	KEGG HON	Details ...	24	-0.21741	-0.78171	0.763975	0.857223	1
KEGG GLY(	KEGG GLY(	Details ...	25	-0.23271	-0.72587	0.876228	0.926841	1
KEGG STEF	KEGG STEF	Details ...	46	-0.26707	-0.70373	0.798	0.944218	1
KEGG PRO	KEGG PRO	Details ...	20	-0.23657	-0.69597	0.757455	0.940982	1
KEGG POR	KEGG POR	Details ...	33	-0.28597	-0.66112	0.760915	0.966335	1
KEGG OO(	KEGG OO(	Details ...	102	-0.1717	-0.63634	0.983087	0.977598	1
KEGG BASI	KEGG BASI	Details ...	33	-0.18085	-0.62348	0.90297	0.975964	1
KEGG MTC	KEGG MTC	Details ...	50	-0.15943	-0.60851	0.975758	0.974978	1
KEGG HYP	KEGG HYP	Details ...	76	-0.26349	-0.6071	0.89391	0.963528	1
KEGG BAS/	KEGG BAS/	Details ...	31	-0.18076	-0.53354	0.934461	0.991708	1
KEGG RNA	KEGG RNA	Details ...	53	-0.15084	-0.48693	0.948498	0.993821	1
KEGG N GI	KEGG N GI	Details ...	44	-0.12166	-0.43653	0.99211	0.991181	1

RANK AT [illegible]	LEADING EDGE
7057	tags=76%, list=40%, signal=125%
7057	tags=75%, list=40%, signal=124%
4021	tags=72%, list=23%, signal=93%
5119	tags=69%, list=29%, signal=97%
4769	tags=62%, list=27%, signal=85%
4021	tags=40%, list=23%, signal=52%
4525	tags=55%, list=26%, signal=74%
4747	tags=71%, list=27%, signal=97%
3775	tags=65%, list=21%, signal=83%
5873	tags=80%, list=33%, signal=120%
3616	tags=73%, list=20%, signal=92%
4021	tags=65%, list=23%, signal=83%
3523	tags=54%, list=20%, signal=67%
3728	tags=50%, list=21%, signal=63%
4303	tags=50%, list=24%, signal=66%
4103	tags=44%, list=23%, signal=57%
6400	tags=71%, list=36%, signal=112%
3885	tags=60%, list=22%, signal=77%
3497	tags=49%, list=20%, signal=61%
3775	tags=44%, list=21%, signal=56%
3803	tags=48%, list=21%, signal=61%
3049	tags=42%, list=17%, signal=51%
1177	tags=14%, list=7%, signal=15%
1549	tags=27%, list=9%, signal=30%
4202	tags=39%, list=24%, signal=51%
5958	tags=60%, list=34%, signal=90%
3593	tags=53%, list=20%, signal=66%
4588	tags=40%, list=26%, signal=53%
7254	tags=58%, list=41%, signal=98%
7463	tags=55%, list=42%, signal=95%
6036	tags=52%, list=34%, signal=78%
1711	tags=34%, list=10%, signal=38%
4021	tags=56%, list=23%, signal=72%
2192	tags=46%, list=12%, signal=52%
4589	tags=45%, list=26%, signal=61%
607	tags=18%, list=3%, signal=19%
3934	tags=46%, list=22%, signal=59%
4805	tags=33%, list=27%, signal=46%
6263	tags=64%, list=35%, signal=99%
6313	tags=48%, list=36%, signal=75%
5973	tags=65%, list=34%, signal=98%
3278	tags=25%, list=19%, signal=31%
4405	tags=50%, list=25%, signal=66%
4805	tags=30%, list=27%, signal=41%
7396	tags=95%, list=42%, signal=163%
3389	tags=38%, list=19%, signal=47%
5170	tags=43%, list=29%, signal=61%
4090	tags=43%, list=23%, signal=56%
3699	tags=36%, list=21%, signal=45%
3237	tags=25%, list=18%, signal=31%
5451	tags=57%, list=31%, signal=82%
5073	tags=52%, list=29%, signal=72%
1715	tags=26%, list=10%, signal=28%
2432	tags=31%, list=14%, signal=36%
3670	tags=34%, list=21%, signal=43%
4518	tags=59%, list=26%, signal=79%
2875	tags=20%, list=16%, signal=23%

4575 tags=33%, list=26%, signal=45%
4102 tags=35%, list=23%, signal=45%
3083 tags=35%, list=17%, signal=42%
3755 tags=55%, list=21%, signal=69%
2049 tags=18%, list=12%, signal=20%
2615 tags=16%, list=15%, signal=18%
2664 tags=27%, list=15%, signal=32%
6263 tags=55%, list=35%, signal=85%
2175 tags=13%, list=12%, signal=14%
4476 tags=43%, list=25%, signal=58%
7396 tags=67%, list=42%, signal=116%
3631 tags=20%, list=21%, signal=25%
6092 tags=40%, list=34%, signal=61%
4575 tags=29%, list=26%, signal=39%
2290 tags=20%, list=13%, signal=23%
4730 tags=35%, list=27%, signal=47%
8650 tags=55%, list=49%, signal=107%
6263 tags=64%, list=35%, signal=98%
2958 tags=15%, list=17%, signal=18%
4761 tags=27%, list=27%, signal=37%
1353 tags=8%, list=8%, signal=9%
4687 tags=42%, list=26%, signal=57%
13158 tags=97%, list=74%, signal=377%
5344 tags=26%, list=30%, signal=38%
2195 tags=9%, list=12%, signal=10%
