# Supplementary material for: The Potential Diagnostic Value of Immune-Related Genes in Interstitial Fibrosis and Tubular Atrophy after Kidney Transplantation
Source: J Immunol Res. 2022 Jun 17;2022:7212852. doi: 10.1155/2022/7212852 (PMC9232312; doi:10.1155/2022/7212852)
Supplement: Supplementary Materials — Supplementary Figure 1: GSEA enrichment analysis of the IF/TA group. Supplementary Figure 2: correlation analysis between ANGPTL3 and differentially expressed immune infiltrating cells. Supplementary Figure 3: correlation analysis between APOH and differentially expressed immune infiltrating cells. Supplementary Figure 4: correlation analysis between EGF and differentially expressed immune infiltrating cells. Supplementary Figure 5: correlation analysis between FCGR2B and differentially expressed immune infiltrating cells. Supplementary Figure 6: correlation analysis between HLA-DQA2 and differentially expressed immune infiltrating cells. Supplementary Figure 7: correlation analysis between LTF and differentially expressed immune infiltrating cells. Supplementary Figure 8: IPA analysis shows the interaction network of diagnostic genes: EGF and LTF (8A), ANGPTL3 (8B), FCGR2B and APOH (8C), and HLA-DQA2 (8D). Merged the above four independent networks to comprehensively analyze the interaction of diagnostic genes (8E). Supplementary Table 1: immune-related genes. Supplementary Table 2: KEGG pathway in normal group. Supplementary Table 3: pathway of ANGPTL3 gene. Supplementary Table 4: pathway of APOH gene. Supplementary Table 5: pathway of EGF gene. Supplementary Table 6: ingenuity canonical pathways. Supplementary Table 7: category. [file 7212852.f1.zip › 7212852.f1/supplementary table6.pdf]

ID	Descriptior	setSize	enrichmen	NES	pvalue	p.adjust	qvalues	rank
hsa01240	Biosynthes	142	0.584365	2.724171	0.001592	0.007936	0.003738	2374
hsa01200	Carbon me	110	0.580654	2.607929	0.0016	0.007936	0.003738	3283
hsa00190	Oxidative p	98	0.578637	2.564096	0.00161	0.007936	0.003738	5477
hsa04146	Peroxisom	77	0.654296	2.789063	0.001629	0.007936	0.003738	3762
hsa04976	Bile secreti	71	0.588415	2.468983	0.001667	0.007936	0.003738	1922
hsa03320	PPAR signa	68	0.490048	2.038171	0.001695	0.007936	0.003738	2406
hsa00983	Drug meta	69	0.569506	2.371484	0.001701	0.007936	0.003738	1662
hsa05204	Chemical c	69	0.569418	2.37112	0.001701	0.007936	0.003738	1484
hsa01230	Biosynthes	65	0.484698	1.986952	0.001712	0.007936	0.003738	2108
hsa01212	Fatty acid r	55	0.524659	2.066382	0.001724	0.007936	0.003738	3254
hsa00010	Glycolysis /	57	0.474755	1.888683	0.00173	0.007936	0.003738	809
hsa00982	Drug meta	57	0.671856	2.672795	0.00173	0.007936	0.003738	1484
hsa00480	Glutathion	53	0.539265	2.110269	0.001736	0.007936	0.003738	1864
hsa00071	Fatty acid d	42	0.625735	2.334117	0.001751	0.007936	0.003738	2283
hsa00140	Steroid hor	50	0.573833	2.223669	0.001751	0.007936	0.003738	2874
hsa00830	Retinol me	50	0.654682	2.536968	0.001751	0.007936	0.003738	1457
hsa04978	Mineral ab	50	0.570825	2.21201	0.001751	0.007936	0.003738	3065
hsa00270	Cysteine ar	46	0.551743	2.087537	0.001776	0.007936	0.003738	1898
hsa00280	Valine, leu	46	0.768067	2.906006	0.001776	0.007936	0.003738	2677
hsa00980	Metabolism	60	0.608855	2.429341	0.001776	0.007936	0.003738	1484
hsa00310	Lysine deg	59	0.47209	1.871878	0.001786	0.007936	0.003738	1178
hsa00330	Arginine ar	48	0.64363	2.457741	0.001786	0.007936	0.003738	1833
hsa00380	Tryptophan	40	0.720794	2.656316	0.001789	0.007936	0.003738	1180
hsa00020	Citrate cyc	29	0.686685	2.358988	0.001792	0.007936	0.003738	3283
hsa00630	Glyoxylate	29	0.75506	2.593878	0.001792	0.007936	0.003738	2677
hsa00350	Tyrosine m	33	0.620835	2.202011	0.001795	0.007936	0.003738	1578
hsa00360	Phenylalan	17	0.697864	2.110117	0.001799	0.007936	0.003738	1409
hsa00760	Nicotinate	31	0.584295	2.043163	0.001808	0.007936	0.003738	2124
hsa00450	Selenocom	16	0.702609	2.082988	0.001812	0.007936	0.003738	1820
hsa00620	Pyruvate m	36	0.621057	2.226502	0.001812	0.007936	0.003738	2834
hsa00220	Arginine b	20	0.688797	2.181083	0.001815	0.007936	0.003738	2016
hsa00770	Pantothena	20	0.68002	2.15329	0.001815	0.007936	0.003738	2655
hsa04964	Proximal tu	20	0.717089	2.27067	0.001815	0.007936	0.003738	2708
hsa00640	Propanoate	30	0.754818	2.612103	0.001828	0.007936	0.003738	2677
hsa00650	Butanoate	24	0.779394	2.579341	0.001828	0.007936	0.003738	3031
hsa00053	Ascorbate	23	0.908035	2.984193	0.001832	0.007936	0.003738	1093
hsa00250	Alanine, as	34	0.608282	2.151383	0.001832	0.007936	0.003738	2571
hsa00790	Folate bios	23	0.676148	2.222112	0.001832	0.007936	0.003738	2243
hsa00860	Porphyrin a	34	0.641011	2.267142	0.001832	0.007936	0.003738	2388
hsa00410	beta-Alani	28	0.719032	2.453176	0.001835	0.007936	0.003738	2571
hsa00260	Glycine, se	35	0.811553	2.883377	0.001842	0.007936	0.003738	1112
hsa00040	Pentose ar	26	0.797585	2.680225	0.001848	0.007936	0.003738	1093
hsa04966	Collecting	27	0.681333	2.301014	0.001855	0.007936	0.003738	3164
hsa00340	Histidine m	21	0.811067	2.579902	0.00188	0.007936	0.003738	1263
hsa05310	Asthma	23	-0.6705	-2.22809	0.002193	0.007936	0.003738	3838
hsa05340	Primary im	34	-0.58737	-2.19691	0.002193	0.007936	0.003738	5328
hsa05330	Allograft re	33	-0.68786	-2.5158	0.002247	0.007936	0.003738	3838
hsa05322	Systemic lu	48	-0.6246	-2.53276	0.002262	0.007936	0.003738	2950
hsa04940	Type I diab	41	-0.61134	-2.37292	0.002288	0.007936	0.003738	3838
hsa05332	Graft-versu	41	-0.62243	-2.41597	0.002288	0.007936	0.003738	3838
hsa05144	Malaria	47	-0.52371	-2.11274	0.002304	0.007936	0.003738	3963
hsa04672	Intestinal ir	42	-0.60074	-2.34377	0.00232	0.007936	0.003738	4025
hsa05320	Autoimmu	44	-0.63035	-2.48714	0.002326	0.007936	0.003738	3838
hsa05416	Viral myoc	58	-0.63902	-2.7046	0.002331	0.007936	0.003738	2354
hsa05134	Legionellos	57	-0.39402	-1.65622	0.002358	0.007936	0.003738	3839
hsa05321	Inflammatc	55	-0.57528	-2.405	0.00237	0.007936	0.003738	4075
hsa05100	Bacterial in	67	-0.42141	-1.85743	0.002392	0.007936	0.003738	3176

hsa05412	Arrhythmo	68	-0.39022	-1.71985	0.002427	0.007936	0.003738	3008
hsa04061	Viral prote	87	-0.46819	-2.13726	0.002481	0.007936	0.003738	4752
hsa05140	Leishmania	74	-0.61735	-2.7415	0.002481	0.007936	0.003738	3963
hsa05235	PD-L1 exp	87	-0.49387	-2.25447	0.002481	0.007936	0.003738	4032
hsa05414	Dilated car	87	-0.39434	-1.80013	0.002481	0.007936	0.003738	4213
hsa05133	Pertussis	71	-0.48928	-2.17494	0.002488	0.007936	0.003738	3925
hsa04657	IL-17 signa	86	-0.37336	-1.69944	0.0025	0.007936	0.003738	4816
hsa04658	Th1 and Th	86	-0.53192	-2.42116	0.0025	0.007936	0.003738	4451
hsa04540	Gap junctio	81	-0.40543	-1.83182	0.002506	0.007936	0.003738	4623
hsa05150	Staphyloco	73	-0.54177	-2.40565	0.002506	0.007936	0.003738	2026
hsa04512	ECM-recep	82	-0.46417	-2.0975	0.002525	0.007936	0.003738	3375
hsa04640	Hematopo	91	-0.52798	-2.41627	0.002525	0.007936	0.003738	3838
hsa05410	Hypertroph	82	-0.37205	-1.68125	0.002525	0.007936	0.003738	2746
hsa05222	Small cell l	90	-0.35967	-1.64056	0.002545	0.007936	0.003738	4542
hsa05323	Rheumatoi	90	-0.38438	-1.75328	0.002545	0.007936	0.003738	4144
hsa04612	Antigen pr	77	-0.4469	-1.9841	0.002577	0.007936	0.003738	4342
hsa04666	Fc gamma	95	-0.4178	-1.92482	0.002577	0.007936	0.003738	2977
hsa04659	Th17 cell d	96	-0.54041	-2.48811	0.002604	0.007936	0.003738	4432
hsa04660	T cell recep	96	-0.3821	-1.75924	0.002604	0.007936	0.003738	4813
hsa04662	B cell recep	79	-0.43094	-1.92398	0.002604	0.007936	0.003738	3434
hsa04064	NF-kappa	98	-0.44543	-2.06006	0.002625	0.007936	0.003738	5316
hsa04620	Toll-like re	98	-0.43652	-2.01887	0.002625	0.007936	0.003738	3856
hsa04625	C-type lec	98	-0.36894	-1.70632	0.002625	0.007936	0.003738	4414
hsa05146	Amoebiasis	98	-0.47148	-2.18056	0.002625	0.007936	0.003738	4624
hsa04611	Platelet act	114	-0.3706	-1.76289	0.002639	0.007936	0.003738	2517
hsa04218	Cellular ser	148	-0.33633	-1.66431	0.002646	0.007936	0.003738	4963
hsa04933	AGE-RAGE	99	-0.36513	-1.68688	0.002646	0.007936	0.003738	4542
hsa04145	Phagosom	144	-0.40811	-2.01049	0.002653	0.007936	0.003738	2354
hsa04668	TNF signal	108	-0.4975	-2.3331	0.002653	0.007936	0.003738	4849
hsa05142	Chagas dis	97	-0.41058	-1.88876	0.002653	0.007936	0.003738	4695
hsa05145	Toxoplasm	108	-0.46201	-2.16665	0.002653	0.007936	0.003738	3963
hsa04670	Leukocyte	105	-0.46352	-2.15928	0.002681	0.007936	0.003738	1539
hsa04380	Osteoclast	122	-0.50287	-2.40669	0.002695	0.007936	0.003738	4910
hsa05135	Yersinia inf	130	-0.39641	-1.91282	0.00271	0.007936	0.003738	4060
hsa04210	Apoptosis	129	-0.33843	-1.62859	0.002725	0.007936	0.003738	4536
hsa04630	JAK-STAT s	139	-0.34513	-1.68307	0.002725	0.007936	0.003738	2087
hsa05161	Hepatitis B	157	-0.42056	-2.11014	0.002732	0.007936	0.003738	4784
hsa05162	Measles	132	-0.41314	-2.00115	0.002732	0.007936	0.003738	3878
hsa04621	NOD-like r	161	-0.43057	-2.16535	0.00274	0.007936	0.003738	3974
hsa05202	Transcripti	158	-0.3822	-1.9165	0.00274	0.007936	0.003738	3425
hsa05203	Viral carcir	161	-0.39431	-1.983	0.00274	0.007936	0.003738	2514
hsa04921	Oxytocin s	138	-0.36217	-1.7669	0.002747	0.007936	0.003738	4322
hsa05164	Influenza A	162	-0.43666	-2.19926	0.002747	0.007936	0.003738	3925
hsa04015	Rap1 signa	194	-0.31669	-1.64168	0.00277	0.007936	0.003738	3366
hsa04514	Cell adhesi	136	-0.435	-2.10788	0.00277	0.007936	0.003738	3136
hsa05169	Epstein-Ba	194	-0.4495	-2.33013	0.00277	0.007936	0.003738	4943
hsa04650	Natural kill	119	-0.4493	-2.13215	0.002778	0.007936	0.003738	2670
hsa04926	Relaxin sig	119	-0.38373	-1.821	0.002778	0.007936	0.003738	4726
hsa05205	Proteoglyc	199	-0.33604	-1.74909	0.002778	0.007936	0.003738	2547
hsa05171	Coronaviru	195	-0.45905	-2.38053	0.002786	0.007936	0.003738	3925
hsa04062	Chemokine	177	-0.41101	-2.09437	0.002793	0.007936	0.003738	4783
hsa05152	Tuberculos	171	-0.35902	-1.81859	0.002793	0.007936	0.003738	4279
hsa04510	Focal adhe	196	-0.39404	-2.04579	0.002801	0.007936	0.003738	2425
hsa05206	MicroRNAs	163	-0.3671	-1.84769	0.002809	0.007936	0.003738	3388
hsa05130	Pathogenic	184	-0.353	-1.80715	0.002825	0.007936	0.003738	1525
hsa05170	Human im	191	-0.31077	-1.60512	0.002825	0.007936	0.003738	4060
hsa05167	Kaposi sarc	175	-0.39105	-1.989	0.002833	0.007936	0.003738	2406
hsa05163	Human cyt	210	-0.35233	-1.84734	0.002865	0.007936	0.003738	4279

hsa05131	Shigellosis	209	-0.3367	-1.76273	0.002874	0.007936	0.003738	2878
hsa04810	Regulation	204	-0.32536	-1.69161	0.002882	0.007936	0.003738	2764
hsa05166	Human T-c	211	-0.47734	-2.49668	0.00289	0.007936	0.003738	4616
hsa04010	MAPK sign	274	-0.29712	-1.61812	0.002994	0.008114	0.003822	4442
hsa04060	Cytokine-c	249	-0.37053	-1.98859	0.003012	0.008114	0.003822	4783
hsa05132	Salmonella	244	-0.37734	-2.01699	0.00303	0.008114	0.003822	3562
hsa04932	Non-alcoh	137	0.330555	1.537087	0.003096	0.008206	0.003866	5477
hsa04714	Thermogen	189	0.323783	1.565041	0.003115	0.008206	0.003866	5477
hsa05165	Human pa	316	-0.27096	-1.4988	0.003165	0.008269	0.003895	2995
hsa04151	PI3K-Akt s	321	-0.26964	-1.49092	0.0033	0.008554	0.00403	3425
hsa05168	Herpes sim	427	-0.37145	-2.12781	0.003559	0.009151	0.004311	5252
hsa00970	Aminoacyl	24	0.605679	2.004446	0.003656	0.009251	0.004358	4247
hsa05200	Pathways i	492	-0.27798	-1.61875	0.003663	0.009251	0.004358	4462
hsa04975	Fat digestio	35	0.503899	1.790308	0.003683	0.009251	0.004358	2677
hsa00532	Glycosamir	20	-0.61837	-1.97726	0.004435	0.011052	0.005207	2743
hsa04142	Lysosome	125	0.335495	1.540994	0.004724	0.011685	0.005505	2271
hsa04350	TGF-beta s	87	-0.34163	-1.55951	0.004963	0.012181	0.005738	4907
hsa04610	Compleme	77	-0.36076	-1.60169	0.005155	0.012435	0.005858	1715
hsa00561	Glycerolipi	55	0.431293	1.698658	0.005172	0.012435	0.005858	2724
hsa05220	Chronic my	76	-0.35873	-1.58695	0.005181	0.012435	0.005858	5678
hsa03040	Spliceosom	128	-0.32984	-1.58868	0.005405	0.012878	0.006066	6753
hsa04072	Phospholip	138	-0.31486	-1.53609	0.005495	0.012924	0.006088	2517
hsa00120	Primary bil	14	0.658246	1.850456	0.005505	0.012924	0.006088	2596
hsa00900	Terpenoid	22	0.612283	1.967848	0.00565	0.013169	0.006204	2931
hsa04360	Axon guida	173	-0.29192	-1.48112	0.005714	0.013224	0.00623	3441
hsa04150	mTOR sign	146	0.311396	1.45339	0.00639	0.014683	0.006917	4312
hsa05221	Acute mye	65	-0.36759	-1.6038	0.007177	0.01633	0.007693	4695
hsa01210	2-Oxocarb	18	0.607482	1.872002	0.007207	0.01633	0.007693	4470
hsa04977	Vitamin dig	20	0.620694	1.965434	0.00726	0.016334	0.007695	1839
hsa04550	Signaling p	130	-0.31284	-1.50955	0.00813	0.018167	0.008558	4907
hsa00910	Nitrogen m	16	0.606064	1.796765	0.009058	0.020101	0.009469	2344
hsa00062	Fatty acid e	25	0.563031	1.885661	0.009191	0.020121	0.009479	3254
hsa01040	Biosynthes	25	0.551446	1.846864	0.009191	0.020121	0.009479	3254
hsa04721	Synaptic ve	68	0.39662	1.649593	0.010169	0.022114	0.010417	4216
hsa04950	Maturity or	19	0.591207	1.842105	0.010889	0.02336	0.011004	890
hsa05418	Fluid shear	132	-0.29215	-1.41512	0.010929	0.02336	0.011004	3446
hsa04110	Cell cycle	117	-0.32702	-1.556	0.010959	0.02336	0.011004	4154
hsa04623	Cytosolic D	56	-0.3555	-1.49408	0.01171	0.024797	0.011681	3894
hsa00670	One carbo	19	0.577749	1.800172	0.014519	0.03035	0.014297	3426
hsa04614	Renin-ang	20	0.562231	1.780312	0.014519	0.03035	0.014297	1864
hsa04270	Vascular sr	116	-0.30039	-1.43547	0.015957	0.033142	0.015613	3468
hsa04664	Fc epsilon	63	-0.3615	-1.56702	0.016588	0.034232	0.016126	2437
hsa05160	Hepatitis C	144	-0.28023	-1.38052	0.018568	0.038075	0.017937	3964
hsa04217	Necroptos	124	-0.29083	-1.39557	0.019231	0.038942	0.018345	4223
hsa04979	Cholestero	44	0.412738	1.554538	0.019231	0.038942	0.018345	1839
hsa04744	Phototrans	20	0.534225	1.691631	0.019964	0.040175	0.018926	4682
hsa00601	Glycosphir	27	0.470208	1.587997	0.020408	0.040816	0.019228	3761
hsa00730	Thiamine r	14	0.581461	1.634598	0.022018	0.043767	0.020618	2736

leading_edcore_enrichment
tags=48%, 55163/23475/8836/8564/54490/2643/210/205/5313/145226/224/219/6470/217/249/10
tags=57%, 8802/48/414328/1962/4329/5313/3417/229/8801/26007/2731/5096/6470/2203/26275/
tags=71%, 127124/155066/1337/1349/245972/9167/245973/50617/529/525/64077/29796/4706/2
tags=71%, 1962/5827/3155/1610/8309/51268/3417/11001/5264/51179/5830/51/26061/6647/30/1
tags=45%, 123264/1576/1244/200931/54490/8671/6555/9376/79799/54575/54658/54657/10864/
tags=40%, 1962/5106/8309/2168/345/11001/34/1582/51/30/2710/5105/33/364/6256/336/2170/1
tags=39%, 7172/1576/54490/2940/2938/1807/151531/79799/51733/54575/54658/54657/8824/54
tags=39%, 1576/54490/130/2940/1551/2938/79799/54575/128/54658/54657/54578/54576/54600
tags=38%, 48/5053/162417/5313/3417/1491/229/445/6470/29968/95/384/435/84706/50/113675/
tags=49%, 36/1962/34/27349/51/1892/38/30/7923/84869/3033/33/54995/1376/51102/10449/799
tags=30%, 5106/130589/130/2538/5313/229/224/223/219/217/2203/3945/10327/9562/128/501/5
tags=49%, 4129/4128/2329/1576/1555/54490/130/2940/2938/2326/79799/54575/128/54658/5465
tags=40%, 124975/2940/51471/3417/2938/79017/9027/2729/2936/4258/2878/2678/2948/119391
tags=55%, 36/1962/130/224/34/223/219/217/51/2639/1892/38/30/128/501/10455/3033/33/1376/
tags=50%, 1576/54490/1551/79799/8644/54575/54658/54657/54578/54576/54600/54577/54659/
tags=50%, 1576/1555/54490/130/1551/145226/79799/54575/10901/317749/128/54658/54657/82
tags=58%, 340024/6569/142680/7421/1317/475/481/2495/261729/113235/4494/55630/115019/1
tags=46%, 55256/1491/64902/23743/635/7263/3945/29968/2729/4357/113675/191/56267/23382
tags=76%, 36/3712/1962/18/3155/4329/224/549/34/223/219/64902/5096/217/26275/1892/38/30
tags=47%, 1576/1555/54490/130/27294/22977/2940/2938/79799/54575/128/54658/54657/54578
tags=29%, 55526/1962/51268/224/223/123688/219/217/2639/1892/8424/38/501/3033/51166/101
tags=46%, 4129/112483/4128/84735/1610/58510/79814/112817/224/8659/223/219/8974/217/26/
tags=57%, 4129/4128/55526/1962/64577/8564/224/223/219/130013/217/26/2639/1892/38/1644/
tags=72%, 8802/48/5106/3417/8801/55753/5105/50/4967/5091/8803/4191/6392/1738/3418/47/6
tags=76%, 48/112817/2731/5096/6470/51179/2653/132158/38/275/9380/54363/5095/50/6472/18
tags=45%, 4129/4128/130/81889/2184/3081/3242/1644/128/316/4282/221/220074/2805/7306
tags=53%, 4129/4128/5053/10249/3242/1644/4282/221/2805
tags=52%, 23475/4907/5169/4860/23409/316/683/93100/349565/54981/64802/23410/554235/51
tags=50%, 22928/1491/51540/51091/22929/56267/118672/10587
tags=56%, 5106/5313/3029/224/223/219/217/134526/3945/38/197257/9380/501/5105/5091/5590
tags=55%, 162417/445/2746/95/384/435/84706/2744/2805/2747/2875
tags=60%, 5169/224/1807/219/217/51733/53354/80347/79717/5167/8876/60496
tags=70%, 5106/8671/2746/1468/358/481/5105/760/2744/762/486/2747/10991/482
tags=73%, 8802/1962/18/4329/79611/8801/5096/26275/3945/51/1892/38/5095/1629/594/84693/
tags=75%, 1962/18/56898/3155/54988/123876/348158/79944/1892/38/3033/7915/6296/622/659
tags=91%, 9365/54490/55586/224/223/219/217/10327/79799/54575/54658/54657/501/54578/54
tags=56%, 2346/18/443/445/8659/64902/2746/8528/435/84706/56954/7915/189/2744/2805/2747
tags=61%, 5053/8836/2643/249/8644/6697/1719/5860/4337/873/4338/5092/10243/84105
tags=50%, 54490/210/79799/54575/54658/54657/54578/54576/54600/54577/54659/54579/1244
tags=64%, 1962/84735/18/4329/224/1807/223/219/217/26275/51/1892/51733/501/221/8310/557
tags=66%, 4129/4128/29958/1610/51268/1491/55349/2731/64902/6470/635/2653/132158/29968
tags=73%, 9365/6652/54490/27294/51084/9942/10327/51181/79799/54575/54658/54657/54578/
tags=78%, 127124/155066/6521/245972/245973/50617/760/529/525/535/10723/528/496/526/52
tags=71%, 4129/144193/4128/84735/443/224/223/219/217/26/10841/3176/138199/501/221
tags=74%, 3125/3127/6356/3117/3122/3118/3112/3123/3126/958/3119/2206/3113/3115/2207/31
tags=71%, 8625/29851/959/695/29760/930/5993/6891/916/920/3543/925/915/5788/4261/3718/8
tags=70%, 3125/3127/5551/3117/940/3122/3118/3112/3106/3135/3123/3126/958/3119/942/3107
tags=56%, 3117/2903/940/6628/3122/1511/3118/3112/6737/3123/729/3126/958/3119/942/2212/
tags=63%, 3125/3127/3552/3553/5551/3117/940/1363/3122/5799/3118/3112/3106/3135/3123/31
tags=61%, 3125/3127/3552/3553/5551/3117/940/3821/3122/3118/3112/3106/3135/3123/3126/31
tags=57%, 7040/7099/4615/3553/6401/948/22914/3820/3576/4035/7059/958/3082/7097/1311/70
tags=60%, 3600/7040/3125/3127/9020/3117/940/3122/3118/608/3112/3123/3126/958/3119/7852
tags=52%, 3125/3127/5551/3117/940/3122/3118/3112/3106/3135/3123/3126/958/3119/942/3107
tags=60%, 3122/6442/6444/3118/27/1525/3112/637/3106/3135/3123/3126/958/1756/3119/942/7
tags=42%, 7099/4615/5970/1195/4790/3553/840/4671/3576/1937/7097/3310/3689/929/834/836/
tags=69%, 3594/4772/7040/7099/3125/3127/8807/3459/50615/3552/8809/5970/6778/4790/3553/
tags=31%, 859/10096/8218/63916/867/399694/26052/3611/71/10095/10163/60/858/391/3059/98

tags=37%, 786/3679/782/55799/3696/83439/6442/8515/6444/3685/6547/3675/489/1756/3694/69
tags=53%, 3606/6354/53833/3627/2921/57007/6351/1235/8807/6356/8809/729230/5197/6374/63
tags=64%, 7040/7099/3125/3127/4615/3459/51135/3552/5970/4790/3553/3717/3117/5603/3122/
tags=49%, 4772/10538/7099/3265/4615/3459/5970/4790/3717/916/920/915/940/5603/4794/5550
tags=44%, 1674/112/111/779/7040/6445/7273/786/3679/782/55799/108/3696/6442/8515/6444/3
tags=51%, 114548/7099/4615/51135/3552/5970/4790/6374/3553/840/2771/1072/5603/3576/5594
tags=47%, 7184/6885/6354/3627/2921/29761/53342/4322/5743/5598/2354/6356/5970/4790/6374
tags=63%, 182/3516/5530/3594/6777/4772/3125/3127/3459/5970/6778/4790/3717/916/920/3117
tags=47%, 2770/10381/347688/112/5598/2776/111/23236/3265/80310/7277/3357/3356/2771/55
tags=41%, 3118/3112/3123/3880/3126/9103/3119/1675/2357/3689/2212/6404/2359/3113/2209/3
tags=43%, 9900/3913/3914/3910/948/5649/3679/3696/8515/3685/1278/7059/3675/3694/3676/13
tags=56%, 3125/3127/931/910/3552/1441/951/4254/3574/3553/2323/916/920/966/3117/925/915/
tags=33%, 3679/782/55799/3696/53632/6442/8515/6444/3685/6547/7168/7171/3675/489/1756/3
tags=41%, 1019/207/3911/5743/1288/1870/10319/578/5970/4790/3913/3914/3910/7157/6502/36
tags=51%, 8600/3600/7040/7099/3125/3127/3552/6374/3553/6364/4314/3117/940/3576/3122/63
tags=47%, 3306/1385/5993/3125/3127/6891/920/3117/925/10437/5720/3821/3822/4261/3122/31
tags=37%, 7454/10096/273/5788/1072/100137049/3985/3635/5335/5594/5321/8605/7408/10000/
tags=61%, 7048/53342/5530/3594/6777/4772/7040/3125/3127/3459/50615/3662/5970/6778/4790
tags=45%, 5062/6885/208/1019/207/5530/4772/3265/9020/5970/4790/916/920/925/915/940/578
tags=47%, 695/29760/208/930/207/5530/4772/3265/11006/5970/4790/971/10990/10288/4794/36
tags=57%, 4791/4050/4616/959/695/29760/7412/6885/2921/142/5743/8600/6351/7099/4615/902
tags=37%, 6351/7099/4615/7096/51135/5970/4790/3553/6373/5603/3576/6352/51311/51284/55
tags=43%, 207/53342/5530/5743/4772/64170/114548/3265/9020/5970/4790/1540/3553/64581/56
tags=50%, 913/5869/2921/3911/1288/2769/2776/1277/7040/10319/23236/7099/910/5970/4790/3
tags=32%, 108/100137049/4638/5603/5500/1278/5594/2778/84876/5321/8605/1281/7408/10672/
tags=43%, 7249/2308/4193/9133/208/1019/204851/7048/207/5530/55957/2309/8900/4772/1870/
tags=45%, 1019/7048/207/7424/1288/6777/4772/1277/7040/23236/3265/3552/5970/4790/3553/3
tags=38%, 3122/23480/3685/3118/81035/3112/7059/3106/3135/3123/3126/338382/9103/30835/1
tags=52%, 7412/6885/208/64764/3627/602/2921/182/207/7424/1385/5743/3600/9020/8809/5970
tags=48%, 208/2770/7048/207/5515/2769/2776/7040/23236/624/7099/4615/3459/51135/5970/47
tags=53%, 6885/208/2770/207/3306/3911/7040/10319/7099/3125/3127/4615/3459/51135/5970/4
tags=31%, 7408/1536/9076/90952/3702/653361/1003/7852/3676/71/3689/1364/7409/4688/4313/
tags=56%, 695/29760/2215/6885/208/4286/7048/207/1385/5530/8600/2354/4772/7040/9020/345
tags=36%, 2776/4772/114548/7099/6196/4615/51135/10094/5970/4790/3553/920/7454/925/100
tags=43%, 4170/835/2081/142/207/3002/4001/1519/578/3265/9020/7277/5970/4790/5551/840/9
tags=38%, 53833/4170/2273/207/53342/3594/6777/3600/3265/3459/50615/1441/6778/3574/3717
tags=39%, 10971/6885/208/64764/7048/4214/207/1385/8900/6777/4772/1870/7040/7099/3265/4
tags=38%, 578/7099/4615/23586/4940/103/51135/3552/5970/4790/3553/6504/916/9451/915/715
tags=36%, 64170/114548/23236/7099/4615/4940/11337/5027/51135/5970/9051/4790/3553/4671/
tags=35%, 5970/8148/4790/25942/6935/4318/2530/4314/2005/1848/7157/51804/3560/5154/596
tags=27%, 6502/9759/468/5594/3718/10379/3106/5925/7534/3135/3065/7185/1234/6850/4067/6
tags=41%, 3768/5530/5743/112/5598/2776/4772/111/779/23236/3265/4659/8681/4881/817/6261
tags=43%, 114548/578/7099/3125/3127/4615/23586/3459/8766/4940/103/51135/3552/5970/4790
tags=31%, 136/4254/54518/1945/135/8817/51735/2903/2771/5154/6494/57568/108/5603/2846/2
tags=42%, 920/6401/1001/3117/925/201633/940/57689/5788/3696/3122/80381/3685/7122/3118/
tags=49%, 5700/4193/695/29760/10018/6885/208/3627/1019/930/5710/207/3516/8900/1870/578
tags=31%, 3821/3822/22914/919/5335/399694/5594/5777/637/3106/3135/5533/962/6850/7535/4
tags=45%, 2783/208/1910/2770/64764/7048/207/7424/1385/4322/112/1288/2769/1277/111/7040
tags=28%, 117581/1655/867/7291/5603/5335/3685/5500/1278/5594/5777/80326/3082/4087/882
tags=43%, 114548/6157/7099/6130/4615/6165/23586/6154/4940/103/51135/5970/4790/3553/612
tags=49%, 10663/2783/6354/208/2770/3627/2921/207/112/2309/6777/2776/111/23236/6351/123
tags=42%, 1385/8411/5530/5993/64170/7040/7099/3125/3127/4615/7096/3459/51135/3552/597
tags=30%, 3696/4638/8515/399694/3685/5500/1278/5594/5728/7059/824/3675/3082/5228/7408/
tags=36%, 4790/6935/7329/6768/1945/4318/4194/7157/1789/5154/4855/54541/672/3667/9759/9
tags=34%, 51135/10094/7277/5970/4790/3553/840/4627/7132/10096/4671/55971/5603/3576/464
tags=38%, 9133/5062/6885/2783/208/2770/207/200315/5530/684/2776/4772/85363/891/578/709
tags=29%, 83439/5603/3576/5335/5594/10379/9641/2790/6772/637/3106/5925/3135/5533/1234/
tags=35%, 1385/5530/5743/112/2776/4772/111/1870/23236/578/6351/3265/6891/5970/4790/355

tags=27%, 10096/7157/4671/823/8767/63916/5603/3576/23048/6352/5335/7322/5594/824/7321/
tags=27%, 5154/3679/85464/1072/1730/3696/4638/3985/2846/8515/3685/5500/5594/3675/8826/
tags=50%, 64764/5901/1019/7048/4214/207/1385/5530/112/8900/6777/4772/3600/111/1870/704
tags=36%, 4909/7048/4214/207/7424/3306/5530/7786/5598/57551/4772/779/7040/1849/3265/61
tags=43%, 10663/3606/944/6354/8718/93/53833/3627/657/2921/7048/133396/53342/2662/8600/
tags=32%, 51135/10094/7277/5970/4790/8677/3553/1639/840/112574/7132/10096/4671/8767/63
tags=49%, 5313/1337/1349/9167/5602/3643/6256/29796/4706/27089/1351/9377/6720/51085/472
tags=49%, 493753/65260/1337/1349/9167/51287/51103/29796/51241/4706/27089/10891/1376/28
tags=27%, 7483/3913/1741/3914/3280/8638/7132/3910/5933/7157/5649/5525/3679/4855/8324/9
tags=27%, 5970/1441/4790/4254/3574/2323/3913/3717/3914/1945/8817/3910/200186/7157/5649
tags=39%, 80778/57547/7637/7567/57615/284406/148254/147694/7571/146540/10780/163050/7
tags=67%, 51091/124454/10667/23395/118672/55157/5188/54938/123263/80222/10352/57176/2
tags=34%, 8648/182/7048/374654/5979/207/7424/1612/3911/5743/112/10161/1288/8313/23604/
tags=31%, 4547/84647/2168/38/81579/335/50487/5407/1056/80168/39
tags=55%, 54480/55454/337876/50515/64132/113189/64131/56548/55790/22856/51363
tags=27%, 26503/1515/4669/3373/5641/9476/3920/10053/1512/6609/1508/245972/5476/411/151
tags=43%, 4093/83729/93/657/7048/1634/5515/57154/7040/3397/4090/64750/652/4089/5933/56
tags=29%, 729/1675/3689/3078/3687/5054/1604/3080/728/716/713/3075/714/5329/3684/718/11
tags=36%, 80339/56894/224/26007/223/219/217/10327/132158/253558/501/2710/196051/3990/1
tags=49%, 6654/6776/5604/1021/25759/2122/4616/4193/208/1019/7048/207/6777/1870/7040/57
tags=52%, 11325/3190/9092/22985/2521/6625/10262/57187/57461/1665/51691/22827/22938/49
tags=27%, 108/100137049/3576/2846/5335/399694/2917/5594/2778/5321/56895/26052/8605/10
tags=64%, 8309/1582/10858/1593/10005/10998/23600/6342/51302
tags=45%, 57107/38/51449/79947/10269/23590/10654/4598/39/3156
tags=35%, 8633/64218/5530/10509/54910/54961/57522/3265/54361/1949/29984/5362/5998/817/
tags=35%, 55437/79109/10542/220441/2887/127124/6520/7855/57600/1147/3643/83667/245973/
tags=38%, 208/7704/207/8900/6777/3265/5970/4790/1848/597/83439/5594/10000/6932/3551/58
tags=78%, 48/162417/3417/95/84706/50/51166/2805/2875/3418/3420/137362/3421/2806
tags=40%, 2346/9963/686/8029/6948/25974/113235/335
tags=37%, 4093/4617/83729/208/93/657/207/648/8313/3397/4090/3265/54361/55183/7994/7483
tags=44%, 2746/760/377677/771/762/2747/766
tags=48%, 122970/11332/1892/3033/641371/10965/51102/10449/79993/51144/51495/60481
tags=52%, 122970/11332/51/30/641371/10965/79993/6342/8310/51144/51495/6319/60481
tags=44%, 6809/6540/127124/6505/155066/245972/10497/245973/50617/529/525/535/5864/528/
tags=26%, 6514/5313/3174/6927/3172
tags=32%, 3552/5970/4790/3553/859/652/4318/6401/7132/7056/7157/5154/6612/2949/5603/368
tags=34%, 10926/8900/1870/7040/701/891/990/4175/4089/11200/5933/29945/7157/7272/6502/9
tags=32%, 11277/6351/23586/103/5970/4790/90865/3553/6352/9641/9447/3551/834/84265/4792
tags=63%, 10588/6470/10841/10840/275/1719/6472/4524/123263/7298/4522/200895
tags=45%, 4311/59272/3816/5476/2028/1636/183/5972/290
tags=34%, 4659/136/8681/4881/135/4627/5592/140465/108/100137049/4638/94274/5500/5594/2
tags=30%, 100137049/5603/3635/5335/5594/5321/8605/10000/6850/4067/5894/9846/7409/2206/
tags=28%, 1870/578/3265/23586/4940/3646/5970/4790/9451/7132/7157/8554/91543/4599/5594/
tags=31%, 55072/10616/6777/114548/7099/3459/29082/3552/6778/1540/90865/3553/8681/3717/
tags=27%, 27329/350/345/4018/348/4036/336/1593/4043/3990/344/335
tags=50%, 91860/810/6011/2979/2779/9187/5158/2978/801/3000
tags=44%, 2525/8702/2651/2528/10402/79369/53947/2527/28/8706/2529/10678
tags=50%, 205/249/204/9054/84284/79178/52

327/79799/29968/3242/54575/2729/53354/54658/54657/204/23498/54578/54576/8942/54600/90
51179/2653/132158/51/29968/55753/2746/1892/38/275/128/54363/5095/84706/50/113675/9104/
7089/535/1351/9377/4720/1350/528/4704/4718/496/4719/4694/526/6392/4695/527/7386/4709/92
0901/2053/8528/54363/26063/10455/55825/5194/283927/5191/390916/189/1384/5825/196743/1
358/54578/54576/54600/54577/54659/54579/481/3781/6580/6256/760/9429/5243/2052/10998/48
376/1593/10998/335/6342/81616/8310/5465/1622/5170
578/54576/54600/54577/4258/54659/54579/9/83549/2948/119391/9446/4257/1854
/54577/4258/54659/54579/9/56267/2948/119391/2052/221/873/9446/4257
5723/6472/27430/5091/2805/4144/729020/2875/6120
93/197322/6342/81616/8310/51144/39/51495/35/6319/60481
105
57/54578/54576/54600/54577/4258/54659/54579/316/2948/119391/221/9446/4257
/2730/2937/9446/2880/4257/26873/290
10449/1632/81616/8310
54579/1586/7923/3291/6716/220074/79154/3284/1577/51144/6715/1585
28/54578/54576/54600/54577/54659/54579/316/51109/9249/157506/54884
181/540/4495/3163/26872/486/7779/4496/645745/140803/2512/4501/6546/482/3162/4493
/2730/27430/2937/2805/4144/4191/10768
/56922/501/5095/316/3033/64087/1629/594/27034/3028/84693/10449/197322/65985/1738/11112
/54576/54600/54577/4258/54659/54579/8574/2948/119391/2052/221/873/9446/4257
57/79723
112849/384/501/2628/2805/5625/4942
23498/501/8942/316/3033/51166/56267
391/2271/1743/6390/3420
9/84693/55902/81888/4191/1738/39

57/83594/23408

2/4191/1738/39/2271

55862/8803/55902/8310/1738/39
35/2571/39/35
576/54600/54577/54659/54579/9104/7358
/2875/80150/2571

4/3163/2235/326625/645
48/2571
/275/9380/501/1757/113675/5723/6472/2628/189
54576/54600/54577/54659/54579/7358
7/9296/523/9550/1188/534/51382

08/3109
4876/958/7535/64421/3575/3932/6890/100
/3105/3113/3134/3115/3108/3133/3109
3113/2209/3115/716/3108/713/714/718/3109/715/87
26/3119/942/3107/3105/3113/3134/3115/3108/3133/3109
19/942/3107/3105/3113/3134/3115/3108/3133/3109
43/3689/7042/3683/3383/5175/975/6347/6403/7058/2532/2995
/3676/942/3113/3115/10673/3108/3601/56477/3109
/3105/3113/3134/3115/3108/3133/3109
1/3107/3689/3105/3113/836/3134/3115/1604/60/3683/3383/8672/5880/3908/3108/3133/857/310
317/2919/7100/2920/3684/718/4792/29108
3117/3122/3118/3112/6772/3123/3126/4087/7097/3119/64127/7043/3113/3115/7042/7100/6775/
44/7414/2335/857/10109/3678

32/3676/71/22801/60/4000/3908/2697/784/3678
355/6364/6373/10563/7132/1236/3560/6846/6375/6363/3576/6352/6362/6348/6349/414062/3588/
/5594/3118/5777/3112/6772/3123/3126/9103/1536/7097/3119/653361/3676/7043/3689/2212/4688
9/917/919/5335/5594/5728/5777/6772/1460/4215/5533/10000/7097/29126/7535/3551/5894/2704
685/6547/7168/2778/7171/3675/489/1756/109/3694/3676/7043/71/22801/7042/60/196883/4000/3
4/3659/6372/3689/3394/929/834/836/353376/716/713/23643/714/3684/718/2353/715/3678/10392
4/3553/6364/3727/4318/4314/5603/3576/5594/4312/9641/6280/3551/6372/23765/836/2919/3934/
/915/3560/9794/5603/3122/4794/917/919/5335/5594/3718/3118/3112/6772/3123/3126/5533/371
92/5154/108/5594/2778/109/113457/112714/5894/7278/5156/84790/196883/1902/5332/2697/557
115/3683/3383/728/716/3108/713/3075/714/3684/2213/718/6403/3109/715
11/22801/131873/7148/1292/1293/1284/9899/1282/3908/960/3339/961/3918/2335/7058/3678/33
/948/952/3122/928/917/3590/3118/3112/911/3123/3675/1438/3126/947/914/3119/3676/945/929/
694/3676/7043/71/22801/7042/60/4000/3908/784/1906/3678
85/5728/5925/3675/7185/10000/3551/9134/836/317/1284/1282/3908/1026/330/3918/7188/2335/
52/6348/6349/414062/3118/4312/3112/3123/3126/1513/7097/3119/942/7043/6372/8792/3689/31
18/3112/3106/3135/3123/3126/972/3119/1520/3310/3107/3105/3113/3134/3115/567/5721/3108/
/6850/4067/653361/5894/3055/2212/9846/7409/10095/50807/2209/8612/10163/27040/5880/5579
0/3553/3717/916/920/4089/3117/915/3560/5603/3122/4794/917/919/5335/5594/3718/3118/3112/
8/5603/4794/917/919/5335/5594/5777/868/5533/10000/7535/3702/3551/5894/5063/7409/27040/3
35/5594/5777/10859/5533/11025/10000/6850/4067/3551/5894/7409/5880/975/27071/5579/2213/
0/23586/51135/5970/4790/1540/3553/7329/7132/597/6363/3576/5335/5971/1460/958/6366/7185
94/6348/6349/414062/9641/6772/958/1513/10000/7097/3551/942/929/7100/353376/3455/23643/1
603/5971/5594/10379/9641/6772/4046/3659/868/5533/30835/10000/6850/3551/5894/834/2207/19
553/3913/3914/3910/3576/1511/1278/5272/2778/911/338382/1281/7097/7043/3689/929/3315/83
/10000/109/6850/6786/4067/71/2212/2207/3937/103910/60/64805/196883/5332/83706/6916/102
7040/891/3265/2113/5928/3552/5970/4790/11200/5933/7157/823/5603/3576/5500/5594/5728/31
717/4089/6401/7056/5603/3576/5335/1278/5594/6772/4087/1281/1536/10000/7043/4313/836/70
1536/7097/3119/113457/112714/1520/653361/1311/71/3107/3689/2212/7278/929/4688/3105/436
/4790/6374/3553/6364/840/4318/6401/7132/4314/5603/6352/468/5594/3659/7185/10000/64127/
90/3553/916/7132/2771/915/5603/3576/917/919/6352/5594/6348/6349/414062/2778/4087/10000
790/3913/3717/240/3914/7132/3910/3117/2771/4261/5603/3122/5594/3118/3112/6772/3588/312
1365/4267/103910/60/3683/3383/10398/5880/5175/83593/5579/3684/7414/10627/4689/83700/58
9/3552/11006/5970/4790/1540/3553/3727/7132/10990/10326/10288/5603/55423/5971/5594/103
96/63916/5603/3576/5335/5594/9815/10000/7535/3551/3676/71/2212/7409/834/10095/10163/270
451/7132/7157/332/823/597/6709/468/5594/1521/637/824/7185/1513/10000/113457/112714/152
7/8554/3560/5154/3953/3590/3718/10379/9180/338376/5777/6772/5617/3588/1438/5771/10000/9
615/23586/51135/5970/6778/4790/3717/4318/4089/7157/332/5603/3576/468/5594/3718/9641/67
7/3560/940/10399/917/51284/4599/3718/10379/9641/6772/637/1460/868/30835/51209/10000/70
/22900/8767/22861/115362/51393/5603/3576/6352/5594/10379/9641/6772/9447/118429/10135/1
5/597/1655/5546/4211/6929/3576/2138/3087/8842/2313/51274/3065/958/8013/3207/7185/2120/5
672/3107/5423/8379/9134/890/5922/3105/836/7533/3134/1960/9586/896/5366/3133/6774/894/1
/5021/786/2771/782/140465/55799/108/952/100137049/4638/53632/6263/5500/5594/2778/5321/
0/90865/3553/3717/7132/3117/56649/4261/3576/3122/6352/91543/51284/4599/5594/10379/3118/
5865/5335/64411/5594/2778/9771/3082/5228/7408/10000/109/23683/2357/9170/5894/71/3689/7
/3112/3106/3135/3123/3126/958/947/6402/4684/914/9076/3119/29126/90952/1003/3676/942/310
/3125/3127/4615/9020/23586/4940/51135/6891/5970/4790/25942/3280/916/3117/11047/915/715
277/5894/3107/3689/7409/3105/836/2207/27040/3937/3683/3383/3932/5880/10870/3133/3455/7
/23236/3265/5970/4790/4318/2771/59345/6019/108/5603/468/399694/1278/5594/2778/4312/279
5/10000/7097/10855/5894/71/7078/7409/4313/3316/4660/836/7074/7042/11211/60/2817/858/309
24/7132/4314/6217/6181/6191/200916/6138/5603/3576/5335/8829/51311/6173/51284/4599/6187/
5/3265/6356/5908/5970/4790/729230/5197/6374/6355/3717/6364/6373/10563/7454/2870/2771/1
0/4790/3553/3717/817/7132/3117/64581/8767/4261/5603/3122/5594/3118/3112/6772/4046/637/3
/10000/3694/3676/3611/5894/1311/71/22801/131873/5063/7148/1292/7409/1293/4660/1284/1039
0427/5335/399694/5594/7168/5728/9839/23414/3065/8434/3925/1786/2146/472/3551/5894/9252
44/2846/5594/7122/5777/10672/9076/113457/112714/64005/9170/3551/71/5063/2212/1364/7278/
9/3265/4615/51135/6891/5970/4790/916/920/7132/2771/915/60489/59345/1072/5603/3985/917/
/10000/6850/4067/6932/3551/942/4277/5894/3055/3107/3105/836/3134/2919/9976/3091/3383/75
3/7132/2771/7157/59345/108/5732/5603/3576/6352/468/3685/5594/6348/6349/414062/2778/279

/10000/472/3551/3611/9252/71/831/929/5216/834/10095/3099/10163/7100/103910/5588/60/837/
/10672/3694/9170/7852/3676/5894/71/22801/3689/5063/3687/7409/5216/4660/10095/7074/1016
0/701/3125/3127/3265/9020/2113/5970/4790/4487/916/920/4089/11200/7132/2005/3117/20018
96/4615/2252/9020/11184/51135/80310/5908/3552/5970/4790/4254/3553/8681/2323/3727/1850/
/57007/3594/3600/7292/7040/6351/1235/8807/3459/6356/50615/2660/3552/8809/1441/729230/5
916/399/5788/23207/83439/5603/3576/388/5287/5594/79443/338382/51209/10000/10121/7097/
20/1350/4704/4718/4719/4694/2931/6392/4695/5465/7386/4709/5291/51422/4717/6391/4714/10
35521/1351/9377/79133/84987/4720/1350/4878/4704/4718/6009/388753/4719/8110/25915/4694/
794/23462/3696/83439/8515/3685/4599/1278/5594/10379/5728/2778/9641/6772/7059/3659/265
/5525/3560/5154/3679/59345/54541/672/3667/3696/4915/468/2846/8515/3685/1278/5594/3718/
728/7730/7249/7673/147948/6432/7633/6885/7559/208/79862/10520/29990/90987/25799/84449/
83459/79731/2193/55699
/3594/8900/6777/2776/3600/111/1870/7040/10319/23236/624/578/3265/54361/2252/3459/2113/1

4/50617/53/2799/8907/535/1497/23062/285362/79158/2519/164/2581/256471/54/10239/3916/8
963/9241/654/9765/7050/3625/2200/285704/5308/5594/4087/26585/4086/7043/10468/2331/704
91/11326/2/715/7450/710
607/5407/1056/80168/55326/55750
8/3265/1488/5970/4790/4089/7157/867/399694/5594/5925/3065/10000/3551/5894/7043/9846/7
115/58517/55110/23451/4686/25766/3192/7307/6432/6632/11017/6636/3306/9984/10465/5164
572/10000/109/6850/9170/5894/9846/8527/5900/2206/8612/2207/5156/196883/9266/9265/1902/

/1945/56963/2771/10512/10371/57689/85464/1072/2041/10507/3985/6259/5163/5335/8829/5594
/529/525/79726/7976/7479/8649/6396/8322/528/6009/526/9470/6195/5170/64121/5291/5582/92
94/890/929/5371/2209/6774/862/3684/1436/4609

/3717/652/4089/8324/6498/4211/6929/5603/3625/5594/3718/1857/80326/4087/10000/6932/397

/6532/526/527/9296/523/9550/6538/534/51382/6511/10814/90423/1213/594855/160/6539

5/10000/4208/653361/1003/3551/71/4688/2950/4313/387082/4205/60/2817/6613/858/3383/588
96/8317/4174/4176/4171/5925/7534/3065/4087/472/23594/7043/4172/8379/9134/890/7533/704
/11035/3665/29108

778/3778/1909/5321/72/8605/5583/10672/109/800/4882/5894/10203/59/3779/4660/5588/19688
/2207/27040/3937/241/5880
/10379/7122/9641/6772/637/5925/7534/10000/9076/3551/5894/1364/1365/836/7533/317/4939/97
/817/7132/823/100137049/3718/10379/6772/637/824/5321/8605/1536/5836/834/353376/6775/67

54/1723/54577/54659/54579/124454/6697/51109/55312/57026/1719/4337/9104/6472/54995/735[illegible]
/5723/6472/4967/189/84693/5091/8803/2098/2805/55902/2747/4524/4191/80201/729020/2875/6[illegible]
296/523/9550/4717/6391/4714/534/10975/4705/4728/4697/51382/6390/4715/374291/4696/1537/
0005/92960/5824/23600/11264/83594/5826/5189/6342/255027/8310/10654/5192/5052/4598/341[illegible]
6/8431/5244

/39

9

/3108/6774/3460/3566/3109/3725

/6366/1234/7852/6372/51554/6357/6368/2919/6358/3587/6347/2920/1436/56477/7133
3/3113/2209/3115/7042/3108/5579/3684/718/3460/4792/2353/4689/3109/65108/3725
40/5588/353376/3091/3932/6774/3460/4792/2353/4773/3725
3908/784/113/3678
2/29108/3725/710
/6347/2920/7188/7128/4792/2353/10758/3725
4/3119/7535/3551/864/4853/4854/3113/3115/27040/5588/3932/6775/3108/3460/3566/4792/2353
9/5159/113/3710/10376/84617/7846/203068

371/7450/3915
/3563/3113/3575/2209/3115/912/1604/3108/960/3684/3554/3566/1436/3109/3678

1643/4792/3915/4609
13/2919/3115/7042/10673/3683/3383/3108/6347/2920/2353/3109/10312/3725
6890/3133/3109
/8877/2213/4082/10109/65108
6772/3123/3126/5533/4087/9466/3119/7535/3551/3113/3115/27040/5588/3091/3932/3108/196/6
3937/5588/8440/3932/1326/4792/2353/4773/3725
/4792/2353/8519/4773/3725
5/6850/7535/4067/472/3551/8792/929/6357/2919/27040/10673/5588/353376/3383/3932/23643/2
1326/4792/2353/3665/3725
960/1263/26253/4792/1959/6237/3710/22808/29108/4773/3725
36/2919/912/7042/1284/1282/3908/5332/2920/5579/3684/3554/3918/7414/2335/3915/87
35/113/5742/10627/3710/7450
06/824/5925/3135/5533/4087/10000/677/472/5894/7043/3107/9134/890/678/3105/3134/7042/50
042/1284/5054/3383/1282/5332/6774/6347/5579/1958/2335/1906/3725
0/3113/3134/2209/3115/11151/60/84790/3108/6890/3133/3684/2213/718/7058/4689/3109/715/1
3551/3976/9252/6372/836/3726/2919/9586/3383/9021/6347/2920/330/7188/197259/1326/7128/4
0/7097/3551/7043/7042/5054/5332/713/714/6347/718/3460/4792/2353/3725
23/3126/958/1234/10000/7097/3119/3551/3310/7043/3113/836/3115/7042/3587/3908/3108/6774/
3494/87
79/126014/6772/10859/54209/5533/9103/11025/1513/814/10000/6850/653361/3551/8792/2212/9
040/3937/60/3932/5880/6347/391/9844/2335/4792/2353/10109/3678/29108/4773/3725
0/472/3551/5894/71/7278/3563/836/1439/317/1616/60/84790/4000/5366/330/4792/2353/3710/1
9466/3976/5894/3563/3575/1439/10401/3587/5156/896/6775/6774/9021/3455/894/1026/5159/36
72/637/5925/7534/10000/7097/3551/5894/7043/9134/890/836/317/7042/1960/9586/353376/6775
097/3551/3310/9134/836/317/4939/896/4600/6774/3455/894/2213/7128/4792/2353/3665/3725
536/64127/3551/24145/7158/114769/834/2919/4939/10628/837/5332/3455/6347/2920/330/7188/
5081/4208/472/942/2078/5090/4086/890/929/64919/5371/2209/4297/894/1026/862/330/3684/643
026/718/7188/9734/4792/3665/1959/5315/3725/87
/5533/8605/814/109/4208/4882/5894/71/3759/4660/60/196883/10398/5332/784/1026/5579/113/2
/7514/9641/3112/6772/637/3123/3126/11100/10000/3119/3551/5894/71/29107/10482/7177/3113
/409/1969/5900/5216/7074/27040/3937/1268/60/3683/5156/196883/2260/1902/5880/285/5332/22
07/3689/1364/6404/3105/3113/1365/3134/57502/4267/3115/3683/3383/5175/3108/3133/3384/368
57/6502/5719/5603/3122/4794/917/919/5971/3718/10379/3118/9641/3112/6772/637/3106/5925/3
/305/3384/5579/3460/4773
90/4087/1281/10000/109/5894/408/59/4313/1284/9586/196883/1282/5332/54331/113/4792/2353/
91/2260/4060/960/406991/6774/3339/2247/1026/2535/5329/3059/1839/5579/967/2335/857/2316/
/5594/10379/140801/4312/6175/9641/6772/6146/6159/729/6168/1536/6193/6850/7097/1675/623
236/59345/6846/6375/108/6363/3576/6352/6362/5335/399694/5594/3718/6348/6349/414062/27
3588/3123/3126/5533/9103/972/30835/10000/6850/7097/3119/64127/1520/5894/7043/3689/3687/
910/60/5156/858/896/1282/10398/5880/7791/3908/64098/894/330/5579/3918/5159/7414/2335/85
2/6624/4853/7078/4854/9134/7148/836/406996/1545/7042/5156/1788/960/406991/6774/894/102
/1365/834/836/10095/10163/9871/3071/7100/60/837/84790/8440/9266/9265/1902/4542/6188/91
/919/5335/5594/2790/637/3106/3135/5533/1234/10000/7097/472/3551/7852/5894/9582/3107/506
538/285/3133/6774/3455/2247/1026/2920/54331/718/4792/2353/3665/3710/2791/4609/4773/3725
90/637/3106/5925/3588/3135/5533/1234/10672/10000/109/3551/7852/5894/11214/3107/3105/836

/9266/9265/10398/92610/5332/960/9181/3059/3554/718/7188/9844/7414/4792/10627/3710/3098/
3/3071/103910/60/3683/5156/2260/1902/10398/5880/2247/3684/5159/9459/7414/2335/10627/62
5/29945/915/7157/3560/108/6929/996/3122/917/468/2114/8829/5971/5594/3718/3118/5728/7514
/1945/8817/786/7132/2005/1848/7157/5154/782/55799/100137049/5603/4915/468/5971/8569/55
197/6374/90865/3574/3553/6355/6364/652/920/10220/6373/3557/10563/7132/1236/3560/6846/6
113457/112714/6932/3551/5894/71/5063/7278/929/5216/834/836/10095/57381/3071/3831/7100/
975/4705/4728/4697/8660/6390/4715/54205/374291/4696/1537/1329/4723/4701/4711/5562/6389
/6392/4695/107/6195/7386/4709/51422/137682/9658/4717/6391/4714/10975/4705/4728/29078/4
08/3106/1857/5925/80326/3135/3675/3065/10000/3694/6932/472/3551/3676/5894/1311/9636/31
/9180/5728/2790/7059/5617/7534/3675/3082/5228/4602/10000/6850/7097/3694/9170/3551/3676/
/7743/84874/5451/207/285268/57232/7773/57677/684/349075/390980/64170/7539/55786/578/31

1488/5970/1441/6778/4790/4254/3574/2323/7483/3913/3717/8202/3914/817/840/3280/652/4318/

4572/427/527
2/3399/3624/4052/4681/4609

042/1026/1643/4792/4609
5/4670/27316/23350/9775/199746/9410/25949/1659/23450/10450/9343/8175/144983/8559/22098
1606/5332/8877/5159/113/6237/22808

1/2048/7220/5533/6586/2042/7852/3611/5894/57556/5063/1969/2047/91653/103910/8440/5361/1
96/55615/523/9550/4041/534/7477/10641/51382/58528/3480/51256/28956/7473/90423/9663/556

5/5894/4086/3198/11211/3720/3399/3624/2260/6774/2247/2535/463/7474/9314/4609

0/5175/9181/6347/3554/1843/857/2353/1906/3725
2/9232/896/894/1026/994/2810/4609

3/10398/5332/5579/113/1906/3710/10267

75/4600/6774/9021/3455/1026/4792/3665/4609
74/3455/330/7188/3460/197259/7128/11035/29108

3/93100/80347/9249/349565/64802/79717/2730/27430/2937/554235/2677/4144/4338/2235/8427[illegible]
120/6392/8310/1738/5232/3418/39/6391/2271/5230/35/1743/6390/5631/3420
1329/90423/4723/4701/5464/4711/495/6389/4722/4700/4713/4702/479/1353/4708/125965/7381/
3/10478/55670/8800/4358/4843/847/1891/54677/2180

3/3109/4773/3725

6774/3554/3460/3566/4792/2353/3109/4773/3725

920/330/5579/3554/7188/7128/4792

054/896/3133/894/1026/83593/90550/6237/3710/10758/22808/4609/4773

0376/3678/10312/84617/7846/203068
4792/2353/1906/11035/7133/3725

/23643/330/3918/3460/4792/3109/3915

0846/4688/3726/2209/7042/3937/3932/9021/3455/7305/3554/2213/3460/2355/1436/4792/2353/46

0376/3725/7846
01/3460/3566/85480/4609
5/6774/3339/1026/5579/1643/4792/2353/3665/1959/4609/4773/3725

/90550/7128/4792/11035/3665/3710/84674/2634/3428/10392/29108/3725
332/1436/1643/4609/604

29904/5997/2353/3710/57118/4773/3725
3/834/836/5371/317/3115/4939/60/64499/896/3383/3108/4600/9021/3455/6347/5579/3460/4792/
247/83593/5579/3684/10235/5159/113/51466/1436/6237/55740/22808
34/6403/3109/6614/9019/83700/58494/1462
3135/3123/3065/3126/958/10000/6850/7097/3119/4067/3551/864/9636/3107/9134/890/3105/311

/1906/2791/3725
/6237/3710/7474/3678/22808/4609
30/2197/3551/6868/9636/23521/2212/6224/834/4939/6142/11224/728/716/25873/6203/713/4600/
90/6772/6366/1234/10000/109/3702/4067/653361/3551/7852/5894/408/3055/6372/7409/6357/63
/2212/929/4360/3113/836/317/2209/3115/2207/7042/11151/3587/1263/26253/3108/3684/8877/2
57/10627/7058/2316/3678/3371/7450/3915/3725/87
6/5579/5159/994/4363/4082/7431/6659/3678/3371/4609
81/4642/4641/3059/3554/4792/2353/10109/10376/29108/84617/3725/7846/203068
53/3105/836/3134/567/25939/5880/8906/6890/3133/27350/5579/54331/7188/4792/2353/7133/37
5
6/3134/567/9586/3587/5156/196883/5880/5332/6890/3133/6774/6347/1026/5579/3554/54331/71

/10109/3678/10392/29108/3725/87
37/55740/10109/3678/7114/22808/87
4/3112/3106/5925/3135/3123/3126/958/4215/5533/4087/10000/109/3119/472/3551/7043/3107/54
94/1847/5321/3082/4215/5228/5533/8605/10000/4208/3925/6789/3551/5894/3310/408/9252/184
54/6375/7293/939/6363/3576/8771/6352/3953/3625/6362/3590/6348/6349/414062/9180/338376/
/103910/60/837/84790/9266/9265/10398/6188/23643/391/330/197259/9844/4792/2353/10627/11
0/4722/51094/4700/6945/10062/4713/83737/4702/4217/3630/4708/125965/7381/5564/5296/7385/
697/6599/6390/4715/788/374291/4696/1537/1329/26291/6300/2180/4723/4701/4711/5562/28958
07/22801/131873/4853/4854/9134/890/7148/1292/1293/3105/836/3134/1284/11211/9586/896/12
/5894/1311/22801/131873/374/9134/7148/1292/1969/3563/1293/7533/3575/1284/9586/5156/896
25/3127/7695/55762/4615/57693/23586/162993/3459/4940/282890/219749/84914/51135/16296

/4089/8817/3910/2771/7157/3560/405/332/5154/4855/59345/10023/8324/23462/108/6502/867/5

38/6100/57819/6628/10915/1655/10594/3178/23020/151903/9129/55119/10189/6626/6430/9416/

10398/6091/5880/2242/64221/23380/10505/2051/10627/6237/55740/7474/1808/4773
52/8325/9681/6199/8131

4/10243/51004/53630/4351/11019/326625/1738

/9114/7385/7384/4731/4712

;89/2274/4773/3725

3665/3109/29108

3/836/3134/317/3115/4939/567/3683/896/3383/3108/6890/3133/960/6774/3455/894/1026/953/71

'6188/6774/3455/714/6347/1839/5579/718/6134/6136/4792/2353/6403/715/6202/6223/7450/3725
368/7074/2919/6358/58191/1794/196883/5880/5332/6774/6347/2920/5579/10235/54331/2268/113
213/718/3460/3109/10312

10/8905/2791/4773/3725

88/113/4792/3710/2791/4609/4773

423/3689/8379/4488/9134/890/3105/3113/3134/3115/7042/567/9232/9586/3683/896/196883/3383
46/7043/374/1844/5922/1969/929/3315/836/7042/1616/5156/2260/5880/285/784/2247/11221/557
7608/5617/3588/1438/958/6366/9235/1234/9466/7852/3976/7043/3603/6372/8792/51554/6357/23
035/2316/6237/113146/10109/10376/29109/4609/10392/29108/84617/3725/7846/302/203068/628
/7384/4731/4712
3/6389/4722/152831/57104/6199/4700/4713/7248/5568/2475/4702/4893/4217/2885/2181/5606/13
282/3955/3908/4600/3133/3455/894/1026/2535/3918/5159/2335/7058/7474/3678/3371/7450/5315
6/2260/1902/1282/285/3908/3455/894/2247/1026/54331/3918/5159/3566/1436/2335/7058/2791/3
6/126070/6891/284307/9831/54753/5970/125893/4790/63934/3553/3717/57335/79788/84671/940

732/23401/83439/2949/3576/2846/5335/3685/5594/3718/5728/2778/4312/2790/6772/637/2736/2

29896/6633/6427/10946/3310/7919/51690

.88/7128/1643/4792/3665/3109/7431/4609/3725

5
3/9844/56477/4792/2791

3/3932/7538/64784/3108/3133/894/1026/3554/3601/1958/4316/113/4792/2353/3109/1959/706/46
'9/10235/3554/1843/5159/1326/994/9448/1436/2353/2316/6237/22808/4609/3725
3765/3563/94/6368/3575/1439/2919/7042/6358/58191/10673/3587/3624/3455/6347/2920/3554/36
31

374/1353/4708/125965/23305/6655/7381/5564/63976/55471/7385/7384/353500/4731/4712
5/3915/10312
3678/3371/7450/3915/4609
)39/5199/9451/163071/140612/7132/79088/3117/7157/7767/121274/55900/55659/342909/8890/7

6508/1857/5925/80326/3675/1909/3065/1438/3082/5228/7185/4087/3714/10672/10000/109/6932

509/4773/3725

501/3460/3566/1436/85480/56477/7133

574/7692/84527/7694/10308/7752/115509/55769/126017/65243/81931/3122/163227/6352/54811

2/6789/9170/3551/7852/5894/9252/7043/4853/4854/9134/890/5900/3563/2950/4313/836/3575/14

/90649/91975/29992/170960/339559/57209/5500/81856/146198/10379/3118/9641/136051/3112/[illegible]

[illegible]39/5371/317/7042/1284/11211/5156/896/196883/3091/2260/1902/1282/5880/6775/3908/5332/5[illegible]

6772/637/3106/3135/10189/3123/6430/3126/972/51427/10000/6427/6850/7097/3119/3551/1626[illegible]

366/6774/3455/894/2247/1026/2535/862/83593/330/5579/10235/54331/3918/5159/3601/7188/11[illegible]

[illegible]5/6672/3107/80818/26152/90321/148156/90338/10793/10482/3105/3113/836/3134/5371/317/80

3/3460/3566/1436/2335/1643/4792/2353/1906/7474/2791/3915/4609/3725

264/3115/4939/567/389114/1616/3108/6890/3133/9021/3455/6347/330/718/7188/3460/4792/36[illegible]

[illegible]5/3109/3678
