# Supplementary material for: The Potential Diagnostic Value of Immune-Related Genes in Interstitial Fibrosis and Tubular Atrophy after Kidney Transplantation
Source: J Immunol Res. 2022 Jun 17;2022:7212852. doi: 10.1155/2022/7212852 (PMC9232312; doi:10.1155/2022/7212852)
Supplement: Supplementary Materials — Supplementary Figure 1: GSEA enrichment analysis of the IF/TA group. Supplementary Figure 2: correlation analysis between ANGPTL3 and differentially expressed immune infiltrating cells. Supplementary Figure 3: correlation analysis between APOH and differentially expressed immune infiltrating cells. Supplementary Figure 4: correlation analysis between EGF and differentially expressed immune infiltrating cells. Supplementary Figure 5: correlation analysis between FCGR2B and differentially expressed immune infiltrating cells. Supplementary Figure 6: correlation analysis between HLA-DQA2 and differentially expressed immune infiltrating cells. Supplementary Figure 7: correlation analysis between LTF and differentially expressed immune infiltrating cells. Supplementary Figure 8: IPA analysis shows the interaction network of diagnostic genes: EGF and LTF (8A), ANGPTL3 (8B), FCGR2B and APOH (8C), and HLA-DQA2 (8D). Merged the above four independent networks to comprehensively analyze the interaction of diagnostic genes (8E). Supplementary Table 1: immune-related genes. Supplementary Table 2: KEGG pathway in normal group. Supplementary Table 3: pathway of ANGPTL3 gene. Supplementary Table 4: pathway of APOH gene. Supplementary Table 5: pathway of EGF gene. Supplementary Table 6: ingenuity canonical pathways. Supplementary Table 7: category. [file 7212852.f1.zip › 7212852.f1/supplementary table7.pdf]

ID	Descriptior	setSize	enrichmen	NES	pvalue	p.adjust	qvalues	rank
hsa01240	Biosynthes	142	0.618439	2.889374	0.001433	0.009559	0.004224	2731
hsa01200	Carbon me	110	0.613343	2.755941	0.001484	0.009559	0.004224	2369
hsa00190	Oxidative p	98	0.582297	2.564267	0.001515	0.009559	0.004224	4286
hsa04146	Peroxisom	77	0.660703	2.769133	0.00155	0.009559	0.004224	2614
hsa00983	Drug meta	69	0.614971	2.517995	0.001558	0.009559	0.004224	1053
hsa05204	Chemical c	69	0.62515	2.559675	0.001558	0.009559	0.004224	1974
hsa04976	Bile secreti	71	0.603031	2.484144	0.001563	0.009559	0.004224	1608
hsa00071	Fatty acid c	42	0.690356	2.554009	0.001577	0.009559	0.004224	1458
hsa03320	PPAR signa	68	0.486715	1.982446	0.001577	0.009559	0.004224	1953
hsa00310	Lysine deg	59	0.442005	1.762923	0.001585	0.009559	0.004224	1454
hsa00270	Cysteine ar	46	0.552752	2.079402	0.00159	0.009559	0.004224	4306
hsa00280	Valine, leu	46	0.777012	2.923052	0.00159	0.009559	0.004224	1811
hsa00980	Metabolisr	60	0.68006	2.714795	0.00159	0.009559	0.004224	2091
hsa00140	Steroid ho	50	0.589899	2.255535	0.001592	0.009559	0.004224	1895
hsa00830	Retinol me	50	0.680946	2.603663	0.001592	0.009559	0.004224	2555
hsa04978	Mineral ab	50	0.643913	2.462063	0.001592	0.009559	0.004224	2227
hsa00010	Glycolysis /	57	0.530474	2.092994	0.001595	0.009559	0.004224	1803
hsa00982	Drug meta	57	0.727868	2.871817	0.001595	0.009559	0.004224	764
hsa00380	Tryptophar	40	0.719565	2.618659	0.001597	0.009559	0.004224	1255
hsa00640	Propanoat	30	0.7555	2.590059	0.0016	0.009559	0.004224	1271
hsa01230	Biosynthes	65	0.546763	2.207659	0.001603	0.009559	0.004224	2173
hsa00330	Arginine ar	48	0.665939	2.512357	0.001605	0.009559	0.004224	1693
hsa00760	Nicotinate	31	0.543599	1.88529	0.001605	0.009559	0.004224	1039
hsa00020	Citrate cyc	29	0.689358	2.350209	0.001608	0.009559	0.004224	3338
hsa00062	Fatty acid e	25	0.579764	1.901915	0.001608	0.009559	0.004224	3760
hsa00630	Glyoxylate	29	0.79552	2.712144	0.001608	0.009559	0.004224	1255
hsa00620	Pyruvate m	36	0.65353	2.323807	0.00161	0.009559	0.004224	2615
hsa00053	Ascorbate	23	0.899049	2.911575	0.001618	0.009559	0.004224	1361
hsa00790	Folate bios	23	0.662317	2.144918	0.001618	0.009559	0.004224	1965
hsa00410	beta-Alani	28	0.738414	2.502502	0.001621	0.009559	0.004224	2358
hsa00650	Butanoate	24	0.783167	2.533822	0.001621	0.009559	0.004224	2358
hsa00260	Glycine, se	35	0.820255	2.88183	0.001623	0.009559	0.004224	1478
hsa00480	Glutathion	53	0.560926	2.16545	0.001623	0.009559	0.004224	2203
hsa00561	Glycerolipi	55	0.442077	1.721743	0.001626	0.009559	0.004224	878
hsa01212	Fatty acid r	55	0.540029	2.103234	0.001626	0.009559	0.004224	3509
hsa00350	Tyrosine m	33	0.650796	2.261502	0.001629	0.009559	0.004224	1223
hsa00250	Alanine, as	34	0.648693	2.262751	0.001631	0.009559	0.004224	1223
hsa00860	Porphyrin a	34	0.671249	2.341432	0.001631	0.009559	0.004224	1002
hsa00040	Pentose ar	26	0.818333	2.71063	0.001634	0.009559	0.004224	1763
hsa04966	Collecting	27	0.668635	2.23543	0.001642	0.009559	0.004224	4181
hsa00220	Arginine b	20	0.687544	2.138307	0.001664	0.009559	0.004224	2111
hsa00360	Phenylalan	17	0.725374	2.173129	0.001664	0.009559	0.004224	1223
hsa00770	Pantothena	20	0.733571	2.281452	0.001664	0.009559	0.004224	825
hsa04964	Proximal tu	20	0.734192	2.283384	0.001664	0.009559	0.004224	2227
hsa04977	Vitamin dig	20	0.6336	1.970538	0.001664	0.009559	0.004224	2312
hsa00340	Histidine m	21	0.803042	2.537354	0.001667	0.009559	0.004224	1575
hsa00450	Selenocom	16	0.66971	1.967554	0.001672	0.009559	0.004224	1691
hsa00900	Terpenoid	22	0.626749	1.98498	0.001675	0.009559	0.004224	4843
hsa01210	2-Oxocarb	18	0.635577	1.911435	0.001704	0.009559	0.004224	2111
hsa00670	One carbo	19	0.619562	1.887175	0.001706	0.009559	0.004224	2669
hsa00120	Primary bil	14	0.714484	2.001	0.001751	0.009559	0.004224	1978
hsa05340	Primary im	34	-0.55304	-2.11676	0.002571	0.009559	0.004224	5043
hsa05330	Allograft re	33	-0.58581	-2.22693	0.002577	0.009559	0.004224	5783
hsa05321	Inflammato	55	-0.48496	-2.09143	0.002584	0.009559	0.004224	4402
hsa05310	Asthma	23	-0.55362	-1.88739	0.002604	0.009559	0.004224	5526
hsa05322	Systemic lu	48	-0.50527	-2.09606	0.002639	0.009559	0.004224	5596
hsa04664	Fc epsilon	63	-0.40925	-1.81471	0.002646	0.009559	0.004224	2994

hsa05144	Malaria	47	-0.43865	-1.80844	0.002646	0.009559	0.004224	4156
hsa05221	Acute mye	65	-0.39679	-1.76725	0.002646	0.009559	0.004224	2655
hsa05416	Viral myoc	58	-0.54261	-2.34663	0.00266	0.009559	0.004224	4689
hsa05320	Autoimmu	44	-0.47876	-1.94365	0.002674	0.009559	0.004224	4689
hsa05012	Parkinson (	203	0.323691	1.590482	0.002706	0.009559	0.004224	3989
hsa05220	Chronic my	76	-0.38207	-1.75465	0.002755	0.009559	0.004224	4719
hsa04714	Thermoger	189	0.331101	1.609794	0.002762	0.009559	0.004224	5346
hsa05133	Pertussis	71	-0.43975	-1.98296	0.002762	0.009559	0.004224	3454
hsa04662	B cell recep	79	-0.44683	-2.06859	0.00277	0.009559	0.004224	4335
hsa05150	Staphylocc	73	-0.41362	-1.872	0.002778	0.009559	0.004224	5366
hsa05332	Graft-versu	41	-0.47119	-1.86042	0.002778	0.009559	0.004224	5854
hsa04512	ECM-recep	82	-0.4384	-2.04002	0.002817	0.009559	0.004224	4226
hsa04540	Gap junctio	81	-0.37561	-1.74159	0.002825	0.009559	0.004224	3325
hsa05140	Leishmania	74	-0.53913	-2.44314	0.002849	0.009559	0.004224	3524
hsa04640	Hematopo	91	-0.39136	-1.86004	0.002882	0.009559	0.004224	5064
hsa05222	Small cell l	90	-0.4008	-1.89676	0.00289	0.009559	0.004224	4226
hsa01522	Endocrine	94	-0.3676	-1.75845	0.002907	0.009559	0.004224	3186
hsa04670	Leukocyte	105	-0.41794	-2.04186	0.002907	0.009559	0.004224	2922
hsa04064	NF-kappa	98	-0.43621	-2.10538	0.002924	0.009559	0.004224	5413
hsa04620	Toll-like re	98	-0.40706	-1.96465	0.002924	0.009559	0.004224	4495
hsa04625	C-type lec	98	-0.36949	-1.78334	0.002924	0.009559	0.004224	2669
hsa05146	Amoebiasis	98	-0.42892	-2.07019	0.002924	0.009559	0.004224	4764
hsa04666	Fc gamma	95	-0.40976	-1.96714	0.002959	0.009559	0.004224	2994
hsa04668	TNF signal	108	-0.48309	-2.37199	0.002959	0.009559	0.004224	4499
hsa05145	Toxoplasm	108	-0.41522	-2.03878	0.002959	0.009559	0.004224	4226
hsa04061	Viral prote	87	-0.39878	-1.86413	0.002967	0.009559	0.004224	5192
hsa04658	Th1 and Th	86	-0.47652	-2.21727	0.002967	0.009559	0.004224	4719
hsa05235	PD-L1 exp	87	-0.51222	-2.3944	0.002967	0.009559	0.004224	4335
hsa04933	AGE-RAGE	99	-0.36187	-1.74364	0.002976	0.009559	0.004224	4719
hsa05142	Chagas dis	97	-0.39904	-1.91605	0.002985	0.009559	0.004224	4023
hsa04659	Th17 cell d	96	-0.48955	-2.3467	0.003003	0.009559	0.004224	4746
hsa04660	T cell recep	96	-0.43507	-2.08554	0.003003	0.009559	0.004224	4335
hsa04650	Natural kill	119	-0.38376	-1.9137	0.003058	0.009559	0.004224	4394
hsa04926	Relaxin sig	119	-0.37375	-1.86381	0.003058	0.009559	0.004224	4715
hsa04611	Platelet act	114	-0.38577	-1.90359	0.003077	0.009559	0.004224	3240
hsa04110	Cell cycle	117	-0.33202	-1.64492	0.003145	0.009559	0.004224	4052
hsa04380	Osteoclast	122	-0.47133	-2.35293	0.003175	0.009559	0.004224	3598
hsa01040	Biosynthes	25	0.562857	1.846451	0.003215	0.009559	0.004224	3509
hsa04550	Signaling p	130	-0.33503	-1.68834	0.003215	0.009559	0.004224	5298
hsa05135	Yersinia inf	130	-0.38045	-1.91722	0.003215	0.009559	0.004224	5386
hsa04072	Phospholip	138	-0.32883	-1.67781	0.003236	0.009559	0.004224	3919
hsa04921	Oxytocin s	138	-0.32357	-1.65097	0.003236	0.009559	0.004224	5021
hsa00970	Aminoacyl	24	0.585357	1.893836	0.003241	0.009559	0.004224	2995
hsa04210	Apoptosis	129	-0.31414	-1.58153	0.003247	0.009559	0.004224	4335
hsa04630	JAK-STAT s	139	-0.32916	-1.67552	0.003289	0.009559	0.004224	4844
hsa05162	Measles	132	-0.40495	-2.04568	0.003289	0.009559	0.004224	4862
hsa04614	Renin-ang	20	0.606565	1.886458	0.003328	0.009559	0.004224	1549
hsa00910	Nitrogen n	16	0.614975	1.806746	0.003344	0.009559	0.004224	3962
hsa04621	NOD-like r	161	-0.37896	-1.96941	0.003344	0.009559	0.004224	2669
hsa05203	Viral carcir	161	-0.38903	-2.02171	0.003344	0.009559	0.004224	4428
hsa05152	Tuberculos	171	-0.29821	-1.56147	0.003378	0.009559	0.004224	4463
hsa04218	Cellular ser	148	-0.35381	-1.81505	0.00339	0.009559	0.004224	4335
hsa05161	Hepatitis B	157	-0.40255	-2.0834	0.003401	0.009559	0.004224	4444
hsa05202	Transcripti	158	-0.35666	-1.84562	0.003413	0.009559	0.004224	3920
hsa05164	Influenza A	162	-0.38974	-2.0239	0.003425	0.009559	0.004224	5366
hsa05206	MicroRNAs	163	-0.40061	-2.07319	0.00346	0.009559	0.004224	4056
hsa05167	Kaposi sarc	175	-0.37164	-1.94696	0.003484	0.009559	0.004224	4339
hsa04360	Axon guida	173	-0.29931	-1.56011	0.003497	0.009559	0.004224	4061

hsa04062	Chemokine	177	-0.37992	-1.996	0.003509	0.009559	0.004224	4719
hsa05130	Pathogenic	184	-0.33676	-1.78799	0.003571	0.009559	0.004224	4023
hsa05170	Human im	191	-0.30514	-1.62752	0.003636	0.009559	0.004224	4335
hsa05163	Human cyt	210	-0.36087	-1.9546	0.00365	0.009559	0.004224	3977
hsa05166	Human T-(	211	-0.42656	-2.31591	0.003676	0.009559	0.004224	4402
hsa04810	Regulation	204	-0.29813	-1.60735	0.00369	0.009559	0.004224	3104
hsa05131	Shigellosis	209	-0.31013	-1.67141	0.00369	0.009559	0.004224	4148
hsa05171	Coronaviru	195	-0.36922	-1.97132	0.00369	0.009559	0.004224	4873
hsa04015	Rap1 signa	194	-0.32454	-1.73189	0.003704	0.009559	0.004224	3306
hsa05169	Epstein-Ba	194	-0.38973	-2.07974	0.003704	0.009559	0.004224	5102
hsa04510	Focal adhe	196	-0.35993	-1.92476	0.003717	0.009559	0.004224	2820
hsa05205	Proteoglyc	199	-0.34676	-1.85134	0.003802	0.0097	0.004286	2859
hsa04060	Cytokine-c	249	-0.28026	-1.54778	0.003861	0.009773	0.004318	5202
hsa05132	Salmonella	244	-0.34749	-1.90562	0.003953	0.009927	0.004386	4210
hsa04010	MAPK sign	274	-0.30031	-1.68003	0.004	0.009969	0.004405	2729
hsa04151	PI3K-Akt s	321	-0.267	-1.52504	0.004032	0.009973	0.004406	4231
hsa05165	Human pa	316	-0.29025	-1.64967	0.004065	0.009978	0.004409	4226
hsa05168	Herpes sir	427	-0.3596	-2.10685	0.004405	0.010732	0.004742	5681
hsa05200	Pathways i	492	-0.27596	-1.64596	0.004739	0.011459	0.005063	4232
hsa01523	Antifolate r	29	0.529322	1.804602	0.004823	0.011576	0.005115	1295
hsa04975	Fat digesti	35	0.488832	1.717429	0.00487	0.011602	0.005126	6657
hsa00532	Glycosamir	20	-0.5818	-1.90679	0.004988	0.011795	0.005212	4953
hsa05134	Legionello:	57	-0.38369	-1.65378	0.005333	0.012522	0.005533	4026
hsa04672	Intestinal ir	42	-0.45387	-1.80451	0.005435	0.012668	0.005597	5526
hsa04115	p53 signali	72	-0.35136	-1.5849	0.00551	0.012751	0.005634	5441
hsa04940	Type I diak	41	-0.46799	-1.8478	0.005556	0.012766	0.005641	4689
hsa05210	Colorectal	85	-0.35278	-1.62856	0.00597	0.013622	0.006019	2655
hsa04721	Synaptic ve	68	0.39329	1.601915	0.006309	0.014295	0.006316	4336
hsa04514	Cell adhesi	136	-0.31576	-1.6039	0.006645	0.014897	0.006582	3620
hsa04145	Phagosom	144	-0.29641	-1.51608	0.006667	0.014897	0.006582	3865
hsa04657	IL-17 signa	86	-0.33076	-1.53904	0.008902	0.019755	0.008729	2729
hsa04919	Thyroid ho	114	-0.30141	-1.48731	0.009231	0.020345	0.00899	2979
hsa05224	Breast canc	135	-0.30027	-1.5254	0.009868	0.021604	0.009546	3061
hsa04932	Non-alcoh	137	0.323095	1.503688	0.01	0.021745	0.009608	4759
hsa04330	Notch sign	52	-0.36985	-1.56628	0.010582	0.022766	0.010059	2979
hsa05100	Bacterial in	67	-0.3583	-1.60531	0.01061	0.022766	0.010059	4509
hsa05016	Huntingtor	254	0.287287	1.454768	0.010724	0.022859	0.0101	4189
hsa05414	Dilated car	87	-0.31749	-1.48412	0.011869	0.025135	0.011106	3958
hsa04935	Growth ho	117	-0.29478	-1.46043	0.012579	0.026464	0.011693	3311
hsa05412	Arrhythmo	68	-0.33784	-1.51647	0.013587	0.028354	0.012528	2392
hsa04950	Maturity or	19	0.537327	1.636688	0.013652	0.028354	0.012528	1114
hsa04066	HIF-1 signa	100	-0.30689	-1.47887	0.014925	0.030801	0.013609	2662
hsa00730	Thiamine r	14	0.595226	1.667005	0.015762	0.032322	0.014281	1739
hsa00030	Pentose pl	28	0.478261	1.620836	0.016207	0.033027	0.014593	2062
hsa00601	Glycosphir	27	0.473136	1.581823	0.01642	0.033251	0.014692	4687
hsa05160	Hepatitis C	144	-0.28114	-1.43799	0.016667	0.03354	0.01482	4339
hsa05231	Choline me	94	-0.31383	-1.50124	0.017442	0.034884	0.015413	3728
hsa04350	TGF-beta s	87	-0.31007	-1.44944	0.017804	0.03539	0.015637	4053
hsa04974	Protein dig	87	0.355001	1.536533	0.018045	0.03565	0.015752	977
hsa05211	Renal cell (	65	-0.33181	-1.47786	0.018519	0.036364	0.016067	4335
hsa04912	GnRH sign	89	-0.30943	-1.46117	0.020408	0.039833	0.0176	3311
hsa05215	Prostate ca	97	-0.29376	-1.41053	0.020896	0.04054	0.017912	4150
hsa05110	Vibrio chol	49	0.396331	1.503971	0.024116	0.046509	0.02055	1751
hsa00061	Fatty acid l	16	0.533991	1.568821	0.025084	0.048089	0.021248	3118
hsa05323	Rheumatoi	90	-0.29922	-1.41604	0.026012	0.049575	0.021904	4023

leading_edcore_enrichment
tags=51%, 210/8564/8836/23475/54490/10327/29968/3242/224/219/217/8942/55163/54575/205/
tags=54%, 8802/414328/2653/51179/1962/229/2203/29968/4329/26275/48/5096/5313/6470/1892
tags=63%, 127124/4706/529/155066/29796/525/1337/245972/27089/9167/528/64077/4695/5061
tags=60%, 1610/8309/3155/51268/51179/1962/30/5827/11001/10901/5264/26061/8528/6647/104
tags=36%, 2938/2940/7172/1807/54490/1576/54575/54658/54657/54600/54576/54659/54579/545
tags=43%, 2938/2940/130/54490/1576/1551/54575/54658/54657/54600/54576/54659/54579/545
tags=41%, 123264/1244/6555/200931/54490/1576/9376/54575/54658/54657/54600/54576/54659
tags=55%, 130/36/1962/224/30/219/217/501/1892/10455/223/128/3033/34/38/51/10449/2639/33
tags=34%, 8309/5106/2168/1962/30/11001/345/1582/2710/34/51/364/5105/1593/33/81616/1376
tags=29%, 51268/1962/224/219/217/8424/501/1892/223/123688/3033/55526/38/2639/51166/39/
tags=61%, 55256/635/29968/64902/23743/7263/1491/191/2729/3945/113675/4357/2937/2805/23
tags=70%, 3155/36/1962/4329/64902/26275/224/30/3712/219/217/18/5096/501/1892/223/3033/
tags=52%, 2938/2940/130/1555/54490/1576/22977/27294/54575/54658/54657/54600/54576/546
tags=42%, 8644/54490/1576/1551/54575/54658/54657/54600/54576/54659/54579/54577/54578/
tags=58%, 130/1555/54490/1576/10901/317749/1551/54575/54658/54657/54600/54576/54659/5
tags=56%, 340024/6569/142680/7421/4495/2495/475/26872/4494/2512/1317/4496/645745/1132
tags=37%, 130/130589/5106/10327/229/2203/224/219/217/2538/5313/501/223/128/3945/5105/9
tags=46%, 2938/2940/130/1555/54490/1576/4129/2329/2326/54575/54658/54657/54600/54576/
tags=57%, 8564/1962/4129/224/130013/64577/1644/219/217/8942/501/1892/223/3033/55526/31
tags=63%, 8802/1962/4329/26275/18/5096/1892/8801/79611/38/51/3945/84693/55862/594/5590
tags=45%, 5053/445/229/29968/48/95/5313/6470/162417/3417/84706/1491/435/113675/6472/50
tags=46%, 58510/1610/112483/79814/4129/8974/224/84735/219/217/8659/112849/501/223/412
tags=35%, 23475/4907/5169/683/4860/316/54981/93100/349565/23409/554235
tags=72%, 8802/5106/48/8801/3417/55753/5105/50/8803/47/2271/6390/4967/3420/5091/6392/
tags=52%, 122970/11332/1892/3033/10449/641371/10965/51102/9524/60481/3030/79993/11714
tags=69%, 2653/51179/48/5096/6470/9380/132158/2731/38/112817/275/84693/54363/6472/559
tags=56%, 5106/224/219/217/3029/5313/501/223/9380/38/3945/5105/197257/134526/55902/39/
tags=91%, 54490/9365/55586/10327/224/219/217/54575/54658/54657/501/54600/54576/54659/
tags=57%, 8836/5053/8644/249/2643/5092/6697/10243/1719/873/5860/4337/4338
tags=64%, 1807/1962/4329/26275/224/84735/219/217/18/501/1892/223/51/51733/221/2571/557
tags=71%, 3155/1962/54988/123876/348158/56898/18/1892/3033/38/79944/6296/7915/39/2571/
tags=69%, 1610/29958/51268/2653/635/4129/29968/64902/6470/501/9380/1491/132158/4128/27
tags=40%, 2938/2940/51471/2878/9027/3417/4258/4257/79017/2729/124975/9446/2936/2948/2
tags=24%, 10327/224/219/80339/217/2710/501/223/26007/132158/56894/253558/196051
tags=51%, 36/1962/30/27349/1892/3033/7923/34/38/51/10449/54995/51102/33/81616/1376/39/
tags=42%, 130/81889/4129/2184/3242/1644/3081/128/316/4128/4282/220074/221/2805
tags=47%, 445/443/2346/64902/56954/8528/18/8659/2746/84706/435/7915/2744/189/2747/2805
tags=44%, 210/54490/54575/54658/54657/54600/54576/54659/54579/54577/54578/79799/1244
tags=81%, 54490/9365/6652/10327/27294/51084/54575/51181/54658/54657/54600/54576/9942/
tags=89%, 127124/529/760/155066/525/6521/245972/528/50617/526/535/9296/245973/9550/51
tags=55%, 445/95/162417/2746/84706/435/2744/2747/2805/384/2875
tags=53%, 5053/10249/4129/3242/1644/4128/4282/221/2805
tags=45%, 1807/224/219/217/5169/53354/51733/8876/79717
tags=60%, 5106/2746/358/762/1468/5105/760/8671/2744/2747/486/481
tags=45%, 9963/686/2346/8029/6948/113235/25974/335/6573
tags=71%, 144193/443/4129/224/84735/219/217/10841/501/223/4128/26/3176/221/138199
tags=44%, 22928/1491/51540/51091/22929/118672/56267
tags=68%, 57107/38/51449/23590/39/79947/10654/2224/3422/10269/3156/4598/23463/116150/
tags=56%, 48/95/162417/3417/84706/51166/50/2805/3420/2875
tags=58%, 10588/10841/6470/10840/275/6472/4524/7298/1719/123263/4522
tags=64%, 8309/1582/1593/10858/10998/10005/6342/23600/51302
tags=65%, 29851/930/7374/5993/29760/915/916/920/5788/958/3718/3543/925/6891/7535/84876
tags=67%, 3002/3122/3117/3118/3123/5551/3106/3135/3126/958/3119/3112/942/940/3113/3134
tags=55%, 3123/6778/4772/3594/8809/8807/7099/7043/3126/50615/3553/3552/3119/3112/3113/
tags=65%, 3122/3117/3118/3123/6356/3126/958/3119/3112/3113/3115/2207/2206/3108/3109
tags=62%, 6628/3122/3117/2903/2215/717/1511/735/3118/3123/3126/958/3119/3112/942/940/2
tags=37%, 3635/5335/5321/5594/207/6654/241/208/8605/2207/2206/7409/8681/5880/5894/9846

tags=51%, 948/6401/7099/7043/1311/7059/3820/3553/958/3689/3082/22914/5175/6347/3576/29
tags=43%, 597/11040/6777/6776/5604/7704/3845/8900/5594/207/6654/1848/5970/208/83439/92
tags=62%, 3118/6442/6444/3123/637/5551/3106/3135/3126/958/3119/3112/3689/942/940/1604/
tags=50%, 3002/3122/3117/3118/3123/5551/3106/3135/3126/958/3119/3112/942/940/3113/3134
tags=37%, 91860/79861/4129/4137/810/4128/292/4706/29796/5602/1337/5715/27089/9167/818/
tags=41%, 6777/6776/4616/25759/5604/1021/7043/1488/578/3845/399694/5594/207/4089/6654/
tags=47%, 51287/65260/493753/4706/51103/29796/25915/84987/285521/79133/1376/1337/5124
tags=45%, 840/3553/3552/2770/3689/6374/834/5594/51135/5970/3659/2771/3576/3394/6372/35
tags=43%, 5604/4772/11006/5530/3845/4794/10859/971/3635/11025/5594/207/6654/5777/5970/
tags=58%, 3872/3122/3117/3866/3858/2215/717/125115/3118/3861/629/9103/3123/3126/1828/3
tags=71%, 3569/3125/3127/115653/3824/3002/3122/3117/3118/3123/5551/3106/3135/3126/3821
tags=46%, 10319/1278/3914/948/3913/1286/1287/1311/7059/1288/9900/960/8515/1292/131873/
tags=37%, 7278/23236/2778/113457/112714/2770/109/84790/5598/5154/5594/6654/111/112/277
tags=47%, 3126/5743/3553/3552/3119/3112/3689/3717/5594/51135/5777/2212/1536/3113/5970/
tags=55%, 100133941/930/7850/3574/2057/3118/1441/931/3123/915/948/916/951/3590/952/966
tags=41%, 10319/3914/5728/3913/1286/1021/1287/578/5743/1288/6502/207/7157/5970/208/718
tags=32%, 2778/6667/399694/109/5594/207/6654/5469/111/112/7157/208/4313/8202/182/4855/
tags=35%, 6494/3689/5335/10398/103910/5175/4478/71/7852/1536/3702/2771/4313/1003/1365/
tags=56%, 7412/9020/55367/6363/148022/7706/4615/597/4616/29760/2921/6351/6366/7099/230
tags=38%, 6351/9641/5604/6373/3661/7099/7096/3553/958/6348/6349/414062/6352/942/5594/2
tags=31%, 834/5594/207/1540/64581/5970/208/3659/2207/10379/5533/5971/5894/6237/5603/67
tags=53%, 5747/22798/2769/7850/1285/1511/735/5331/2921/10319/1278/3914/3913/3315/7099/
tags=37%, 3635/5335/5321/5594/207/273/4651/2212/208/8605/2213/8612/10163/7409/7408/305
tags=44%, 2921/602/5604/6401/8809/4314/64764/6364/7132/5743/840/3553/6352/6374/5594/20
tags=42%, 10319/3914/3913/7099/7043/8651/3306/3588/3126/7132/958/2770/3119/3112/3717/5
tags=52%, 6363/3606/6362/1235/3572/2921/6351/6366/6373/729230/8797/10563/8809/8807/570
tags=57%, 6777/6776/3118/3123/6778/915/4772/3594/916/5530/4794/920/55534/3560/3126/371
tags=51%, 5604/915/4772/5728/10538/7099/916/5530/3845/4794/920/1460/917/919/3717/5335/
tags=46%, 6777/6776/5331/4846/7056/4772/1278/6401/1286/7043/1287/1281/3845/2308/1288/3
tags=43%, 5515/5331/915/7099/7043/916/7132/3553/23236/2778/2770/917/6348/6349/414062/9
tags=54%, 3572/6777/6776/3118/3123/6778/915/4772/3662/3594/916/5530/4794/920/3560/3126
tags=42%, 5604/915/4772/916/5530/3845/4794/920/5788/925/917/919/5335/940/5594/207/6654/
tags=38%, 25759/5604/637/5551/4772/8797/3106/3135/5530/3845/3821/3822/399694/919/3689/
tags=43%, 108/1910/5331/4846/25759/5604/6013/1278/1286/1287/64764/1281/3845/2782/1288/
tags=37%, 23236/2778/2770/109/5584/5321/5594/103910/207/111/112/71/2212/2776/208/8605/
tags=34%, 10274/4176/1021/10735/996/7043/8900/7533/10926/7534/991/29945/701/6502/2033/
tags=43%, 54209/7132/126014/3553/10859/3552/8600/11025/10326/5594/207/55423/4286/1540/
tags=48%, 122970/11332/30/51/641371/10965/6342/9524/60481/6319/8310/79993
tags=42%, 1855/8313/3977/658/81029/652/4093/657/3572/4211/5604/79923/55183/6926/4090/3
tags=45%, 5747/9564/7456/148022/3606/8874/7454/5585/4615/10096/1793/5604/4772/3661/709
tags=36%, 9267/2917/3845/2846/8526/7249/23236/2778/399694/4254/109/5335/5321/5154/5594
tags=43%, 782/6261/8536/3762/4638/108/4659/5331/4846/6263/5604/4772/53632/5530/3845/81
tags=50%, 10667/51091/5188/124454/118672/55157/80222/123263/79731/10352/55699/54938
tags=40%, 5604/637/5551/1521/8797/4170/578/3845/2081/7132/840/7278/113457/112714/4001/
tags=38%, 2057/5771/3572/6777/6776/1441/6778/338376/4170/3594/8651/3590/3560/3588/5061
tags=44%, 4615/57506/4940/6777/6776/10399/9641/637/915/3661/6504/7099/1021/916/578/330
tags=45%, 4311/59272/2028/3816/5972/5476/290/1636/183
tags=56%, 2746/762/760/2747/771/766/377677/768/1373
tags=26%, 834/5594/51135/115362/114769/1536/5970/837/6347/3576/10379/4671/24145/11842
tags=39%, 85477/51564/27044/2967/3661/1021/3106/64764/3135/578/3845/8900/7533/23513/75
tags=38%, 9103/537/3123/972/637/7099/7043/8411/5530/7096/23545/817/3588/3126/7132/4046
tags=39%, 5604/4772/5728/5928/1021/7043/3106/3135/5530/2309/3845/8900/7249/2308/2305/3
tags=39%, 4615/57506/6777/6776/537/6778/9641/5604/637/4772/3661/7099/7043/64764/3845/8
tags=37%, 4314/8013/221037/578/2138/2313/5328/1655/6692/8900/3560/5546/3206/2308/958/4
tags=51%, 3117/79671/5610/581/148022/3606/7706/56649/5611/4615/57506/10241/4940/3838/3
tags=44%, 4170/1021/8651/7168/672/6541/578/1945/3845/5328/90427/1946/10253/5743/9839/3
tags=39%, 9641/5604/637/4772/3661/57580/1021/3106/3135/5530/578/3845/2782/7132/5743/37
tags=34%, 8829/56963/6586/3611/29984/5530/1949/1945/3845/3983/817/10371/1946/64218/54

tags=46%, 6777/108/2868/5331/2921/6351/25759/6366/5604/6373/729230/10563/57580/6358/63
tags=37%, 7099/114548/10972/9267/9138/347688/10094/578/2846/7132/840/5010/3553/7278/11
tags=35%, 5604/637/915/4772/200315/3661/7099/684/916/3106/3135/5530/578/3845/2782/920/
tags=36%, 1021/9138/3106/64764/3135/5530/578/3845/2782/3588/7132/7249/5743/3553/23236/
tags=45%, 3123/5604/915/4772/5728/8829/996/7043/916/3106/64764/3135/5530/3845/920/8900
tags=28%, 4627/23191/3689/8515/10398/5154/5594/103910/54961/6654/624/3696/4478/1730/71
tags=33%, 55072/3661/10616/7099/114548/9267/3611/10094/2309/7132/5216/2308/3553/23236/
tags=41%, 23521/4615/57506/4940/3572/735/6142/629/6139/9641/140801/3661/8829/7099/114
tags=32%, 9693/57568/23236/2778/2770/4254/109/6494/3689/5335/5584/9771/5154/3082/5594/
tags=45%, 898/930/3516/11047/4615/57506/4940/3118/4616/29760/3123/9641/637/915/5719/36
tags=29%, 8515/10398/5154/3082/5594/103910/1292/207/131873/6654/1293/3696/71/7148/208/
tags=29%, 5335/960/3082/5594/207/6654/5777/4478/71/6548/7157/208/5500/3316/4060/4313/8
tags=41%, 3977/6363/3606/6362/8718/10148/658/7850/652/3574/1235/2057/657/3572/2921/635
tags=36%, 2317/64746/10121/8797/7184/79443/7099/114548/399/9267/347688/10094/578/7132/
tags=25%, 5598/5321/5154/3082/5594/207/6654/1848/51135/3727/7157/5970/208/8605/2005/21
tags=32%, 29941/10319/1278/3914/5728/3913/7184/4170/7099/1286/1021/1287/64764/672/230
tags=34%, 10319/1278/3914/5728/3913/3661/1286/1021/1452/1287/3106/64764/3135/578/1311/
tags=43%, 162972/5819/8503/84911/79744/6431/55786/7592/3122/91120/64170/57232/284306/
tags=32%, 7428/10319/7704/3914/5728/3913/7184/3594/1286/1021/7043/9138/1287/1488/1123
tags=38%, 8836/1244/6470/2348/9429/6472/113235/4524/7298/1719/1147
tags=63%, 84647/4547/2168/38/39/335/81579/50487/80168/5407/8613/2169/1056/2806/5319/29
tags=75%, 26229/10090/29940/55454/337876/55501/64132/50515/54480/56548/113189/55790/6
tags=44%, 1195/7099/3306/840/3297/3553/57396/1937/3689/834/5970/3576/4671/929/7097/331
tags=55%, 3122/3600/9020/29851/3117/3118/3123/3126/958/3119/3112/942/608/940/7852/3113
tags=50%, 55240/55367/143686/5268/11200/581/898/27244/3732/4616/50484/8493/637/5728/87
tags=54%, 3118/3123/5551/1363/5799/3106/3135/3126/3553/3552/3119/3112/942/940/3113/313
tags=28%, 5594/207/4089/6654/10018/7157/208/332/83439/374/6932/5880/5894/7042/10000/83
tags=44%, 6809/6540/127124/6505/529/155066/525/10497/245972/528/50617/6532/526/535/92
tags=38%, 3106/3135/920/3126/5010/5788/958/925/80381/3119/3112/23705/3689/4684/942/940
tags=40%, 3106/8411/347688/3135/1311/23545/3126/338382/7059/6441/7278/113457/112714/3
tags=28%, 5598/6374/5594/6280/3727/5970/6347/3576/6372/5603/3551/3934/29761/836/4312/2
tags=29%, 8850/5335/9882/2033/5594/207/3065/5469/71/6548/7157/208/25942/8202/4855/489/
tags=27%, 6667/8600/399694/26508/5594/207/6654/7157/208/83439/8202/3280/182/23462/803
tags=41%, 5313/4706/29796/5602/3643/1337/27089/9167/4695/4705/4728/4704/51085/4717/62
tags=35%, 8850/26508/2033/3065/3280/182/23462/4855/3955/9794/6310/4853/1857/3714/6868/
tags=34%, 10096/1793/25759/3611/10094/8218/63916/399694/71/858/10163/857/391/9844/100
tags=34%, 79861/2878/6647/292/4706/29796/5602/5432/2892/1337/5715/27089/9167/10671/46
tags=36%, 3679/108/6442/6444/7043/7168/6547/7171/2778/109/8515/111/3696/112/71/22801/3
tags=28%, 4214/23236/2778/2770/399694/109/3717/5335/2033/5594/207/6654/111/112/1385/27
tags=26%, 3696/71/22801/83439/2697/3676/3675/3694/489/3908/6932/55799/4000/1756/60/367
tags=26%, 6514/5313/3174/3172/6927
tags=28%, 5335/2033/5594/207/405/1536/5970/208/3099/6513/226/5163/285/3939/10000/8569/
tags=43%, 205/249/9054/204/84284/52
tags=36%, 414328/51071/229/64080/2203/132158/9104/729020/6120/5631
tags=48%, 8702/2525/2528/79369/10402/2651/2529/2527/53947/28/8706/10678/6487
tags=30%, 9641/5604/637/3661/1021/578/3845/7533/7132/5010/7534/23586/5594/207/6654/945
tags=36%, 204962/3845/8526/7249/6667/5335/5321/5154/5594/207/6654/1609/208/1120/8605/5
tags=36%, 56963/7043/57154/2200/5308/4090/285704/3399/3625/93/26585/6667/2331/2033/55
tags=18%, 4311/340024/9056/11136/1803/7512/6520/59272/23428/8645/6505/117247/10008/56
tags=35%, 5604/7428/7043/112399/3845/5546/3082/2033/5594/201163/207/6654/2113/405/208/
tags=33%, 3845/817/4214/23236/2778/109/5598/5321/5594/6654/111/112/2776/8605/4313/8681
tags=33%, 5728/7184/4314/64764/3845/5328/6692/2308/5154/2033/5594/207/6654/1385/7157/2
tags=29%, 127124/529/155066/5336/525/245972/528/50617/526/535/9296/245973/9550/51382
tags=50%, 27349/7923/54995/51102/81616/84869/2180/197322
tags=42%, 7099/7043/4314/6364/23545/3126/3553/3552/3119/6348/6349/414062/8600/3112/63

54658/54657/5313/6470/54600/54576/54659/54579/145226/54577/249/54578/53354/2643/51109
2/8801/2746/3417/26007/128/84706/132158/2731/38/51/55753/275/113675/84693/54363/6472/5
7/526/535/9296/4705/4728/4704/4717/245973/6390/9550/1349/51382/1350/4720/4722/6392/473
455/3417/5830/51/2053/26063/55825/283927/54363/5191/189/1384/1891/5194/10005/5052/6342
577/54578/4258/4257/151531/51733/8824/79799/9446/2948/9/83549
77/128/54578/4258/4257/79799/9446/2948/9/2052/221/873/64816/56267/1553/1577/119391
/54579/54577/54578/10864/358/79799/6580/760/9429/8671/3781/2052/5243/10998/5244/6256
3/81616/1376/39/1632
/10998/2170/6342/6256/335/1622
79723
3382/27430/4144/56267/2730/4507/4191/6898/58478/259307/51074/883/137362/6611
549/316/34/38/10449/84693/3028/594/56922/27034/5095/39/64087/1629/11112
59/54579/54577/128/54578/4258/4257/79799/8574/9446/2948/2052/221/873/1553/1577/119391/
7923/1586/79799/3291/220074/1577/3284/6716
4579/145226/54577/128/54578/316/51109/79799/8228/9249/54884/1577/157506/53630/54905/15
35/3163/4501/55630/115019/140803/4493/1181/7779/261729/4502/486/540/3162/481
562/55902/221/80201/5232
54659/54579/54577/128/54578/316/4128/4258/4257/79799/9446/2948
6/4128/38/26/2639/23498/51166/39
2/5095/39/8803
/5723/2805/27430/4144/384/729020/6120/5232/3420/5091/5631/2875/5230
3/112817/26/2628/2805/2593/384/4942

162/4191/3421/6391/1738
5
02/50/189/81888/5095/39
2271/5091/5162/4191
54579/223/54577/54578/79799/7358/9104

48/35
622/35
731/55349/275/2628/113675/6472/189/1757/5723/2593
578/2937/290/2880/119391/9588/2730

5342/84869/35/9524/2180/60481/6319/197322/3030/8310/79993

4/3163/645
54659/54579/54577/54578/79799/7358/729020/6120
382/496/10723/1188/534/9114/527/523/90423/495

4597

/3575/4261/100/3932/64421/6890
/3115/3133/3108/3107/3105/3109
5970/3115/3108/7097/6775/6772/7042/64127/4087/6774/3460/3109/7100/3725/3566

212/3113/729/3115/3108/716/6737/718/2209/713/714/3109/715/87
/100137049/5603/10000/27040/4067/3937/6850

95/6403/7097/7058/2532/7042/975/3683/3383
9/6932/5894/5371/2209/3551/10000/862/4609/6774/3684/890/1436
71/3113/3134/3115/3133/27/1525/3108/3908/3107/5880/857/3105/836/3683/3383/1756/60/3109/
/3115/3133/3108/3107/3105/3109
4695/4705/4728/4704/5683/4717/6390/5686/5701/1349/1350/10131/65018/5705/4720/4722/639
3065/7157/5970/208/2122/5894/9846/3551/7042/10000/4609/867/1026/5925/1643/4792
1/27089/9167/6009/788/4695/388753/4705/4728/4704/4717/6390/1349/1350/4878/4720/4722/81
3376/929/716/718/23643/5603/836/713/2353/3684/714/3678/715/29108/10392/3725/710
208/2213/10288/5533/7409/5880/5894/3551/10000/4067/2353/975/27071/4792/8519/6850/4773/
119/25984/3112/3689/1675/3075/2359/3880/2212/3113/2213/3115/6403/6404/3108/716/2357/71
/3553/3552/3119/3112/942/940/3113/3134/3115/3133/3108/3107/3105/3109
1293/3696/7148/22801/375790/3676/3675/3694/3908/7058/9899/3911/3339/1282/2335/3678/36
6/1902/2771/2697/5894/5159/196883/113/5332/10376/5156/84617/7846/3710/5579/203068
3115/4688/3108/3676/653361/7097/718/2209/5603/6772/7042/4689/2353/3684/3460/3109/4792/
/920/3126/3553/2323/911/3552/925/917/3119/3112/4254/960/914/1438/1604/3113/947/3115/35
5/3675/3908/3911/3551/10000/836/4609/1282/2335/1026/9134/330/5925/1643/3685/4792/7188/
5894/4853/10498/5603/196883/113/3714/10000/2353/4854/1026/1839/5925/3725
4688/7409/7408/3676/653361/9076/5880/5603/58494/83593/4689/90952/10627/1364/3684/7414/
85/5328/7132/5743/3553/6357/958/1460/23586/142/8600/5335/51135/1540/5970/3576/7185/75
07/51135/5970/208/3576/51311/51284/353376/929/7097/23643/5603/6772/3551/10000/2353/34
72/1263/3551/1960/10000/868/1959/4792/26253/29108/6850/22808/3710/4773/3725
1286/7043/1287/1281/338382/1288/3553/23236/911/2778/5269/3689/5869/2776/5970/3576/929/
5/653361/8681/5880/5894/9846/100137049/2209/10000/27040/10095/50807/4067/8877/10109/3
7/1385/5970/208/3659/6347/7185/182/6372/5603/3551/64127/10000/836/9252/3976/9586/2353/
594/207/51135/3113/5970/208/2771/3115/1234/3108/3908/3587/7097/3310/23643/4261/5603/67
07/6358/6356/6364/1236/3560/3588/7132/6357/6348/6349/414062/6375/6846/6355/6352/6374/
8/917/3119/919/3112/3717/5335/5594/3113/5970/3115/864/182/7535/5533/9794/3108/4853/677
940/5594/55509/207/5777/5970/208/7535/5533/353376/7097/5894/5603/6772/3551/10000/2704
553/23236/3552/3717/5335/5594/207/4089/1536/5970/208/6347/4313/3576/5603/6772/7042/10
19/6352/5594/207/51135/624/2776/5970/208/6347/2771/3576/7097/718/5603/3551/7042/10000/
/50615/3553/3718/917/3119/919/3112/3717/5335/5594/4089/3113/5970/3115/7535/9466/5533/3
/5777/3702/5970/208/5063/7535/5533/7409/5894/5603/3551/10000/8440/27040/5588/2353/3932
5335/962/5594/6654/3384/22914/5777/2207/3133/7535/5533/7409/7305/4277/3107/10870/5880/
6019/23236/2778/2770/399694/109/5594/207/6654/111/112/1385/5970/208/2771/4313/2790/27
5500/2771/2207/84876/7408/8681/10672/6786/100137049/5603/196883/113/6916/10000/5332/4
4089/3065/891/7157/4172/4171/8317/4174/7272/5933/7042/8379/4087/4609/9232/1026/9134/5
3727/2212/1385/5970/208/2213/10288/4688/10379/5533/8792/5971/7305/653361/9846/814/220

845/8324/3399/3718/3625/5462/93/3717/7994/648/5594/207/659/4089/6498/4086/5460/208/362
9/114548/9138/10094/920/3553/63916/925/9815/5335/834/5594/207/51135/71/2212/2776/5970/
/207/6654/111/1609/112/208/8605/1902/3576/2207/2206/8612/8681/10672/5894/5159/6237/98
7/952/5743/4881/4208/23236/2778/2770/109/5021/4882/10398/5598/5321/5594/111/112/71/277

142/839/1520/84790/6709/5594/207/5783/9451/10018/71/7157/5970/208/332/3563/7185/824/14
5/3718/3717/5154/2033/207/6654/5777/1438/5617/3953/208/3563/8554/10379/9466/85480/918
6/3560/51209/3553/3718/1460/3552/917/23586/30835/940/103/207/51135/9451/4478/7157/597

9/22900/4939/5603/10628/6772/3551/9447/64127/5332/90550/3455/10135/2919/7158/330/4792/
34/991/3718/728340/730394/8850/5423/6502/2033/5594/3065/1385/7157/5970/3134/1234/7185/
/3553/3552/3119/30835/3112/1520/3689/3717/2033/5594/207/5869/51135/2212/64581/1385/311
552/204851/5594/207/2113/891/7157/5970/208/5500/3134/3576/3133/5533/824/3107/5894/623
900/4214/7534/3718/23586/3717/2033/5594/207/4089/6654/51135/1385/7157/5970/208/332/357
208/3087/6667/5081/51274/942/5154/648/1848/4286/3065/4086/7157/5970/25942/2005/3576/71
118/3123/9641/5604/637/8106/8797/3661/7099/1021/114548/578/23633/3126/7132/7177/3553/
99694/1789/5335/960/406996/5598/5154/648/2033/5594/659/6654/27086/54541/3065/6768/100
17/942/5335/2033/5594/207/9976/1385/7157/5970/208/3134/1234/2790/3576/83439/2791/3133/
10/2770/2042/5335/10398/5594/103910/54961/10154/91584/659/7852/85464/5063/2771/2041/6

356/6364/2309/3845/2782/1236/6357/3718/23236/2870/2770/6348/6349/414062/399694/6375/68
13457/112714/4627/23191/6188/84790/834/5594/51135/5777/4651/71/2212/5970/837/5063/1902
7132/2770/917/919/5335/5594/207/51135/891/85363/7852/2776/5970/208/5063/6891/2771/3134
2778/2770/6348/6349/414062/6667/6352/109/5732/5594/207/6654/111/112/7852/1385/2776/715
/3560/3126/7132/958/4214/991/3718/917/3119/29945/701/8850/3112/109/3689/5423/4488/2114
1/6548/221178/7852/22801/5500/85464/5063/1902/3687/7074/10163/7409/3676/3675/3694/4660
/63916/6352/5335/960/10398/7321/834/5594/103910/207/71/7157/5970/208/837/3576/831/7307/
548/6217/4314/6169/11224/6230/7132/6229/3553/6181/200916/6146/6157/23586/6188/2197/533
135/207/111/25865/112/71/2776/208/1902/2771/7074/64411/7409/7408/9732/5880/5894/2357/5
61/1021/916/3106/3135/578/4794/8900/3126/958/3718/917/23586/3119/919/3112/960/6502/207
22801/5500/5063/858/7409/7408/3676/3675/3694/4660/3908/824/5880/5894/857/5159/7058/522
58/7074/80326/7409/4660/10855/8826/7097/5894/857/6237/2817/5603/7042/11211/10000/836/9
1/1441/6366/6373/729230/338376/8797/10563/3594/8809/8807/57007/7043/6358/6356/6364/35
/338382/5216/840/5788/388/51209/3553/7278/63916/113457/112714/23191/3836/6188/1639/847
22/1847/5533/5971/1844/57551/374/3925/8681/11221/929/1846/5880/5894/55799/5159/6237/28
9/1945/1311/3845/5529/2846/2782/1946/3560/7533/6794/7249/7059/1288/2323/7534/3718/4254/
3845/5529/23545/55534/8900/7132/7249/7059/8638/5743/23513/2308/1288/8324/2778/5584/85
730051/3117/5610/7549/581/148022/54753/8890/57573/27102/57615/93134/285268/148254/575
99/578/3845/817/2846/2782/8900/3560/5743/840/2308/1288/8324/2323/3718/23236/2778/23604/

881/337/10554/338/1208/8513/84649
4131/51363/22856
0/718/836/3684/2919/4792/29108/7100/317/2920
/3115/3108/3676/10673/3601/3109/56477
797/1021/7249/6241/92344/891/7157/51246/64065/64393/836/1026/9134/5054/4194/1643/894/4
4/3115/3133/3108/3107/3105/3109
6/4087/2353/4609/5900/1026/1643/5366/3725
96/245973/9550/51382/10814/6538/6511/534/5864/594855/9114/527/523/90423/6530/773
/3384/914/5175/3696/1001/3113/947/3134/3115/6402/23114/6403/1003/6404/3133/1365/57502/
119/30835/81035/3112/1520/3689/84790/5869/71/2212/1536/64581/3113/6891/2213/3134/3115/
353/2919/23765/10758/4792/7188/7128/2920/3725
6513/5894/4853/10499/6772/9969/10000/5332/4609/4854/60/3685/9968/3091/5214/5579/23389
26/4855/6932/10023/5894/4853/23401/1857/11211/3714/10000/2353/4609/2260/4854/1026/5925
56/6390/1349/1350/4720/4722/6392/2931/4731/4711/7386/4694/1351/374291/10975/51422/1006
4854/151636/9253
95/867/7414/2335/26052/10109/3059/60/3678
95/55062/55081/4705/4728/4704/5683/2880/4717/6390/5686/5701/1349/1350/5705/4720/4722/63
676/3675/3694/489/3908/55799/196883/113/7042/4000/1756/60/3678/3685/784
76/208/2771/5894/5603/6772/196883/113/10000/5332/9586/2353/9021/3667/6774/3726/3710/55
8/3685/784

6774/1026/5054/3460/5209/7076/1906/3091/5214/5579/2026/3098

1/91543/7157/5970/208/4599/8554/1365/10379/9076/5894/4939/6772/3551/10000/836/4600/902
6261/8612/10163/8681/5880/5894/5159/100137049/6581/10000/8527/1606/2353/5900/5156/571
94/9241/659/4089/64750/4086/10468/3624/654/5933/7042/4087/4609/4681/9765/7050/4052
46/6519/5645
5063/6513/5894/7042/10000/1026/3091/3725
/5894/100137049/5603/196883/113/5332/4215/1839/1958/3710/5579/3725/2796
950/5970/208/83439/6932/5894/5159/2078/3551/10000/9586/2260/5156/1026/9134/5925/4792

52/3689/942/940/6374/3113/6347/3115/3576/8792/3108/6372/7097/10673/7042/4312/2353/2919/

)/57026/2729/79799/9054/204/23498/54995/93100/6697/79717/6472/1723/349565/124454/84274
5902/50/189/4524/2098/5095/5723/2747/2805/39/8803/9104/2271/6390/4967/729020/80201/612
1/479/4711/496/7386/4694/5464/1351/374291/10975/534/1537/7385/6391/4723/4709/126328/12
/5825/92960/4358/23600/5826/255027/196743/11264/847/10654/5192/5189/8800/2180/8504

1565

558

/8672

2/4731/6622/4711/7386/4694/5694/347733/118424/1351/10213/7416/374291/10975/7295/5704/1

.10/6392/137682/4731/4711/7386/4694/2180/1351/6599/374291/10975/57104/51422/26291/1537

/5579/3725
.8/2209/728/713/3684/3683/3383/714/3109/715

35/7450/1284/3918/961/3915/3371

/65108/5579/3725
63/945/928/3108/3676/3675/929/3575/912/2209/3684/3678/3554/3109/1436/3566
1284/317/3918/3915

/3683/3383/83700/60/5579/87
35/8792/5971/353376/929/7329/23643/10673/3551/27040/5588/4067/3932/2919/3383/330/3554/
55/4792/7100/3665/1326/3725

/3908/7097/912/5272/3911/7042/836/5332/1282/3684/2919/7414/2335/3554/1284/3918/2920/55
985/4082/6850/65108/5579
9021/3726/2919/197259/3383/330/4792/7188/7128/7133/2920/11035/1906/1326/3725
72/3911/3551/7042/10000/836/6774/330/3460/3109/4792/3918/3915
7852/6368/5197/6347/1234/51554/3576/6372/3587/2919/1436/7133/2920/56477
5/5603/6772/3551/3714/27040/5588/2353/3932/4854/3460/3109/4792/4773/3725/3566
)/29126/5588/2353/6774/3932/4215/3460/4792/3091/4773/3725
)00/836/5332/4087/6774/1282/3383/2335/5054/1284/1958/1906/5579/3725
5332/713/4087/2353/714/5054/3460/4792/3725
108/5603/6772/3551/27040/196/4087/5588/2353/6774/3932/3460/3554/3109/4792/3091/4773/37
/3937/868/4792/4773/1326/3725
/5894/3105/27040/836/3455/3932/3937/3683/3383/3460/6850/4773/5579
)1/54331/5894/5603/196883/113/408/10000/59/5332/4087/9586/4312/2353/1282/4792/1284/190
)67/64805/10627/3937/60/10235/7450/83706/6850/5742/3710
)25/994/894/23594/472/896/890/2810
9/5603/6772/3551/7042/10000/4689/2353/9021/3455/3932/3937/3726/2274/3460/3554/4792/143

4/6929/80326/3198/6932/5894/5603/1857/11211/10000/3976/4087/4609/6774/2260/2535/9314/3
/208/6347/3576/7535/10163/7409/3676/5880/6196/5603/3551/10000/391/27040/9844/10095/235
16/100137049/196883/113/10000/8527/56895/1606/5332/8877/9170/5900/5156/26052/9265/9266
6/8605/5500/2771/5533/4660/8681/3759/5894/55799/814/100137049/196883/113/5332/2353/10

39/5894/1616/3551/10000/836/10376/2353/4000/823/330/60/4792/472/317/5366/7846/3710/372
)/1439/3587/5894/5159/3575/6775/6772/10000/3976/9021/4609/3455/6774/3601/5156/1026/346
)/208/2213/4599/10379/51284/7097/3310/4939/6772/3551/10000/836/4600/2353/3455/6774/868/

3428/7188/29108/7128/10392/2634/2920/11035/3665/3710/84674/3725
/3133/10379/9759/3107/718/3105/5933/5966/1960/8379/836/9586/4067/6774/9734/1026/9134/6
.3/5970/208/2213/3115/3687/2207/5533/3108/4360/929/3587/7097/5894/718/2209/4261/5603/11
/3105/5603/5933/7042/677/10000/678/83593/4087/4609/90550/1026/9134/823/5054/5925/1075
76/353376/7097/5894/6775/5603/6772/3551/7042/1960/10000/836/9586/2353/4609/6774/3339/1
.85/3207/6929/2120/8842/5090/4300/929/5371/8148/2209/2078/5966/862/4609/3684/1026/6491
3552/23586/3119/3836/3112/6352/3717/103/834/2033/5594/29107/207/51135/64499/91543/71/3
18/7148/7157/8434/4855/6624/7431/3925/7329/9759/5894/4853/5159/3551/7042/1545/836/925
10379/5533/3055/4277/6932/54331/3107/5894/285/718/3105/5603/6772/3551/10000/836/4067/2
091/2048/57556/5533/10509/7220/91653/5880/5894/5163/6237/5362/57522/8440/10627/10507/2

46/6355/6352/109/3717/5335/6374/5594/207/6654/111/112/7852/6368/2776/3702/5197/5970/20

/4644/3576/4642/10163/1365/4671/9076/10672/3071/9871/5603/3551/8440/836/10095/10376/64

/1234/2790/2791/3133/5533/25939/54331/3107/5880/7097/5894/3105/5603/9582/3551/10000/83

57/5970/208/6347/6891/2771/3134/1234/2790/3576/2791/3133/5533/10672/54331/3107/3587/58

/2033/5594/207/4089/111/2113/112/1385/7157/3113/5970/208/2005/3134/3115/6929/3133/2001

/10672/5880/8826/5894/3071/5159/6237/10095/10627/9459/2260/9170/3684/7414/55740/5156/3

/10163/4671/3099/929/824/718/7322/5603/3551/23048/10000/9844/9252/10095/5332/5588/1062

5/1675/103/834/5594/25873/51135/6232/2212/1536/5970/729/6347/3576/6223/6403/6203/4599/

159/6237/285/51735/5228/5603/196883/113/10000/27040/5332/83593/1268/2260/3937/9170/59

/51135/3065/10018/7157/3113/5970/208/5713/25942/6891/3134/3115/864/3280/3133/10379/310

28/3911/10000/10627/1282/7414/5156/7791/2335/330/60/3678/64098/3685/894/7450/1284/896/2

67/4087/5329/4609/6774/867/3339/2260/406991/2335/1026/3059/1839/60/3678/7078/3685/2535

90/920/10220/1236/3560/3588/9573/7132/9235/939/50615/133396/3553/6357/3557/958/3552/36

790/10398/834/5594/103910/207/5869/51135/71/5970/208/837/5063/3576/83439/64837/3831/467

35/3310/5228/100137049/6196/5603/1616/1850/3551/7042/408/10000/836/9252/6789/4915/8569

4602/3717/8515/5523/5154/3082/5594/1292/207/131873/6654/54541/1293/5617/3696/10018/13

15/5523/26508/2033/5594/1292/207/131873/6654/3065/1293/3696/1741/1385/7148/7157/5970/2

47/4615/163081/57506/7594/284390/10189/4940/125893/7730/26974/3118/440515/7637/55762/6

2770/6667/1612/4254/109/3717/5335/6502/2736/5732/26508/5154/3082/2033/5594/207/4089/6

72/896/317/5366/2810

3108/3676/9076/9019/3107/3105/29126/58494/90952/1364/3684/3683/3383/83700/3109/3685/6

9146/3133/4688/3108/4360/653361/929/3107/7097/7058/718/3105/2209/11151/55176/10376/46

/1643/2535/2247/7474/3725

2/1537/7385/6391/4723/4709/126328/125965/9377/7384/4700/4697/6945/51094/4713/4715/469

392/4731/5440/5435/4711/7386/4694/5694/3766/347733/1351/10213/7416/374291/10975/2902/5

579

21/4609/3455/6774/1364/975/1026/5925/4792/317/3665

53/3091/5579/3725

3683/3383/3109/2920/3725/10312

/10243/2937/554235/1719/7358/51004/27430/9104/1728/9249/4337/51805/4144/5372/7389/7372
0/5232/3420/5091/847/5631/6392/2875/5230/35/5162
25965/9377/7384/9114/527/4700/523/4697/4713/4715/4696/4701/90423/495/4716

.813/1537/25828/10105/3709/5568/7385/6391/4723/7280/4709/147700/801/7314/126328/125965

/6300/5568/7385/6391/9658/29078/4723/107/4709/126328/125965/9377/5468/7384/4700/4697/4

4792/7188/472/7128/6850/2920/5579

79/3915/87

'25/3566

5/3725

36/6850/4773/2355/3725

3720/2247/7474/463
3/3932/3937/2335/10109/60/3678/4792/29108/4773/3725
5/6850/22808
26/60/5997/784/29904/57118/3710/4773/5579/3725

5
0/894/896/10401/3566
/9134/4792/894/896/7128/317/3665/3725

672/5925/1959/4792/894/7188/896/890/5366/6850/5922/3665/5315/3725/87
.151/6772/1263/7042/64127/10000/836/8877/3684/3460/3109/26253/317/6850/10312
3/894/472/896/890/22808/3710/4773
026/9134/5925/1959/1643/4792/890/317/3665/4773/5579/3725
9/330/1643/894/472/4297/890/1436/64332/604
113/5970/208/6347/3115/3576/4599/10379/8766/3108/51284/5894/5371/10482/4939/4261/6772/
/23405/1788/3667/4609/6774/4854/1786/5156/406991/1026/9134/2146/3678/7078/4194/994/894
2353/4609/3455/6774/2919/3383/1026/7538/5925/4792/6850/2920/2247/3665/3091/3710/4773/3
2047/55740/64221/3985/2242/1808/1969/5361/10505/2051/4773/7474/23380

8/6347/2771/1234/2790/3576/2791/7074/7409/3055/653361/6372/54331/1794/5880/5894/6772/1
005/2353/9170/9181/1364/10109/4542/3059/60/3554/9265/9266/55971/4792/84617/29108/7100/
36/2353/6890/3985/27350/567/4792/7188/8906/472/7133/8905/3710/4773/5579/3725
30/5894/11214/3105/5603/196883/113/3551/10000/836/5332/9586/4609/6774/5156/1026/6890/5
86/5533/3108/5971/6513/3107/3105/196883/113/3551/7042/4316/10000/8379/4087/9586/2353/4
683/2335/7114/10109/60/3678/3985/3685/2247/22808/87
7/9181/7414/823/92610/10109/3059/60/3678/3554/9265/9266/4792/7188/472/29108/10392/7100
10379/51311/51284/9636/716/7097/6202/718/4939/5603/6772/728/3551/6134/6168/713/4600/61
00/3684/55740/5156/3683/23683/60/10235/1969/1436/2247/22808/51466/5579
08/5971/7431/9636/3107/7097/3105/4939/5603/6772/3551/10000/836/4067/4609/3455/6774/368
2316/3918/5579/3915/3371/3725/87
/2316/2247/22808/3091/3710/5579/7474
25/93/6348/6349/414062/8600/6375/6846/6355/6352/8771/608/94/6374/659/1438/7293/5617/39
71/23207/112574/929/6932/7097/5894/3071/6237/23643/57381/5603/3551/10000/391/9844/836/
/2353/4609/2260/5156/4215/3554/9448/994/1843/10235/784/2316/1969/1436/5922/2247/22808/
85/7148/7157/5970/208/22801/1902/3563/2790/2791/200186/55012/3676/3675/374/3694/9180/3
08/22801/3659/3134/83439/3280/182/23462/4599/3133/80326/55012/10379/4855/3955/9794/367
432/55659/10520/162966/6429/100289635/3123/972/9641/637/8427/10308/57677/10838/7699/8
54/4286/3065/624/1438/111/2113/405/112/10018/7852/2776/7157/2950/5970/208/1902/2771/3

614/1462
39/3684/6890/60/3678/3109/3685/84617/715/7846/203068/10312

/4701/6720/4716/3630/5465/5564/4714/1329/84701

704/146754/4294/7019/1537/10105/7802/7385/5441/6391/1768/4723/6875/7280/4709/147700/1

2/4338/55312/11019/53630/2730/80347/4351/7390/80308/4522/5805

/9377/7384/5692/5702/4700/7326/4697/4713/3708/842/4715/4696/4701

713/6195/10891/1374/4715/4696/4701/63976/84699/6199/91942/4716/28958/5564/4714/4893/1

3551/11100/10000/836/4600/9021/3455/3383/7514/3460/60/3109/4792/896/29108/317/3665/55
/472/4082/6659/5579/3371/4363
725

96883/113/3551/408/10000/9844/5332/2268/4067/6774/2919/58191/10235/4792/2920/5579/564
/4641/7846/203068/3725

925/3554/3685/567/4792/7188/3710/4773/5579
609/9232/3932/3601/3683/3383/1026/7538/9134/7514/4215/5925/1959/3554/3109/567/4792/89

/3710/3098/3725/87
36/6868/4312/2353/3455/6774/714/1839/4792/7450/715/6850/5579/3725

/3383/1026/9134/6890/5925/1643/3109/567/4792/894/7188/953/896/890/7128/317/6850/3665/

53/7852/6368/5197/7292/6347/3603/3563/1234/51554/3576/3624/9466/8792/85480/654/6372/91
10095/10376/5287/2353/10627/4609/113146/197259/10109/330/60/9265/9266/4792/302/84617/
5579/1326/3725
908/54331/5525/7097/5894/5159/3575/285/7058/5228/3911/3551/10000/4915/9586/3667/4609/
76/3675/3694/9636/3908/6932/3107/5525/5894/4853/5159/7058/3105/6772/3911/5933/1857/355
4527/163049/3661/219749/684/84874/390980/3106/349075/94039/3135/79788/578/284307/577
32/3563/4313/2790/3576/83439/7185/8202/3280/1909/2122/2791/182/23462/80326/4855/3675/1

26328/125965/9377/5468/7384/5692/5702/4700/4697/9001/4713/27113/3708/10891/842/5437/47

329/84701/57492/155/126129/353500/4702/55811/23305/6194/4718/4719

'9

77

4/472/896/890/706/1958/64784/4773/3725

3725

80/1439/3587/3575/10673/7042/3976/3455/3601/2919/58191/3460/3554/23765/1436/7133/2920/
29108/2316/10392/7100/29109/11035/7846/203068/3725/6281

3455/1282/2260/9170/5156/2335/1026/9134/3678/3685/894/7450/1284/896/1969/1436/3918/685
1/11211/10000/836/4600/9586/3455/1282/4854/2335/1026/9134/5925/3678/3685/894/7450/2535
11/339559/84671/7574/3126/7132/7249/65243/163071/29990/3553/57209/7559/163050/5199/107
0672/6513/3908/6932/54331/1439/10023/5880/5894/5371/4853/5159/3575/6775/23401/5228/677

715/4696/4701/10120/84699/8678/5693/5717

/56477/3566

0/2247/3915/3371/3566
/1284/472/896/890/3918/5315/3915/7474/3371/10312
/80/23586/3119/126070/126231/3112/90987/6352/115509/7695/3717/140612/29992/7551/57335/
72/3911/196883/113/1857/3551/7042/11211/3714/10000/836/9252/6789/5332/83593/4087/4312/[illegible]

[illegible]6427/81931/207/7692/51135/342909/9831/7700/84924/9451/84449/55769/7752/5451/146540/71[illegible]
[illegible]2353/862/4609/3455/6774/867/1282/2260/9170/3601/4854/5900/5156/2335/1026/9134/330/3460

57/25799/3113/80818/5970/208/5500/6347/6891/3134/3115/63934/90649/90321/3133/10793/12[illegible]
/5925/1643/10235/3685/4792/894/7188/2535/1284/896/890/1436/317/5366/3918/1906/2247/309

[illegible]017/7694/10379/84914/163227/3108/91975/7767/282890/6430/3107/146198/136051/7097/5371/[illegible]
[illegible]1/5579/3915/7474/3725/3566

/10482/9534/718/148156/3105/4939/26152/79088/1616/6772/3551/55900/121274/10000/51427/8

36/9021/3455/80264/162655/6890/330/6672/3460/3678/3109/170960/567/4792/7188/90338/818[illegible]

56/54811/389114/317/6850/3665
