# Supplementary material for: The Potential Diagnostic Value of Immune-Related Genes in Interstitial Fibrosis and Tubular Atrophy after Kidney Transplantation
Source: J Immunol Res. 2022 Jun 17;2022:7212852. doi: 10.1155/2022/7212852 (PMC9232312; doi:10.1155/2022/7212852)
Supplement: Supplementary Materials — Supplementary Figure 1: GSEA enrichment analysis of the IF/TA group. Supplementary Figure 2: correlation analysis between ANGPTL3 and differentially expressed immune infiltrating cells. Supplementary Figure 3: correlation analysis between APOH and differentially expressed immune infiltrating cells. Supplementary Figure 4: correlation analysis between EGF and differentially expressed immune infiltrating cells. Supplementary Figure 5: correlation analysis between FCGR2B and differentially expressed immune infiltrating cells. Supplementary Figure 6: correlation analysis between HLA-DQA2 and differentially expressed immune infiltrating cells. Supplementary Figure 7: correlation analysis between LTF and differentially expressed immune infiltrating cells. Supplementary Figure 8: IPA analysis shows the interaction network of diagnostic genes: EGF and LTF (8A), ANGPTL3 (8B), FCGR2B and APOH (8C), and HLA-DQA2 (8D). Merged the above four independent networks to comprehensively analyze the interaction of diagnostic genes (8E). Supplementary Table 1: immune-related genes. Supplementary Table 2: KEGG pathway in normal group. Supplementary Table 3: pathway of ANGPTL3 gene. Supplementary Table 4: pathway of APOH gene. Supplementary Table 5: pathway of EGF gene. Supplementary Table 6: ingenuity canonical pathways. Supplementary Table 7: category. [file 7212852.f1.zip › 7212852.f1/supplementary table8.pdf]

ID	Descriptior	setSize	enrichmen	NES	pvalue	p.adjust	qvalues	rank
hsa05022	Pathways c	402	0.291082	1.544246	0.001422	0.008143	0.003836	4219
hsa05010	Alzheimer	312	0.344131	1.785856	0.001453	0.008143	0.003836	4219
hsa05014	Amyotroph	305	0.304613	1.572003	0.001479	0.008143	0.003836	4176
hsa05016	Huntingtor	254	0.374759	1.895532	0.001493	0.008143	0.003836	4176
hsa05012	Parkinson (	203	0.417162	2.041583	0.001515	0.008143	0.003836	2996
hsa05020	Prion disea	228	0.344295	1.712522	0.001517	0.008143	0.003836	3089
hsa04714	Thermoger	189	0.455631	2.20935	0.001527	0.008143	0.003836	2966
hsa04152	AMPK sign	115	0.364479	1.653893	0.001577	0.008143	0.003836	2479
hsa01200	Carbon me	110	0.642886	2.898094	0.001585	0.008143	0.003836	2824
hsa05017	Spinocereb	123	0.349146	1.598803	0.001587	0.008143	0.003836	4532
hsa01240	Biosynthes	142	0.515782	2.408857	0.001603	0.008143	0.003836	4074
hsa04932	Non-alcoh	137	0.443882	2.065397	0.001603	0.008143	0.003836	3276
hsa04723	Retrograde	121	0.365388	1.665639	0.001605	0.008143	0.003836	2687
hsa00310	Lysine deg	59	0.453493	1.826277	0.001616	0.008143	0.003836	4200
hsa00010	Glycolysis /	57	0.591389	2.361714	0.001623	0.008143	0.003836	2836
hsa00260	Glycine, se	35	0.664328	2.399917	0.001623	0.008143	0.003836	4036
hsa00330	Arginine ar	48	0.630963	2.429887	0.001623	0.008143	0.003836	4282
hsa00982	Drug meta	57	0.483445	1.93064	0.001623	0.008143	0.003836	4746
hsa00071	Fatty acid (	42	0.682449	2.574924	0.001631	0.008143	0.003836	2836
hsa00250	Alanine, as	34	0.58333	2.097282	0.001634	0.008143	0.003836	4972
hsa00270	Cysteine ar	46	0.549838	2.100552	0.001634	0.008143	0.003836	4914
hsa00280	Valine, leu(	46	0.76213	2.911573	0.001634	0.008143	0.003836	3058
hsa00380	Tryptophar	40	0.635627	2.367247	0.001634	0.008143	0.003836	3328
hsa00830	Retinol me	50	0.528714	2.053509	0.001634	0.008143	0.003836	4386
hsa04976	Bile secreti	71	0.499468	2.089775	0.001634	0.008143	0.003836	3724
hsa00561	Glycerolipi	55	0.436571	1.728251	0.001637	0.008143	0.003836	2908
hsa00980	Metabolisr	60	0.455679	1.832226	0.001637	0.008143	0.003836	3724
hsa01212	Fatty acid r	55	0.578581	2.290422	0.001637	0.008143	0.003836	2655
hsa04146	Peroxisom	77	0.61642	2.614934	0.001639	0.008143	0.003836	4361
hsa00480	Glutathion	53	0.432425	1.698625	0.001642	0.008143	0.003836	4070
hsa00620	Pyruvate m	36	0.73282	2.656743	0.001642	0.008143	0.003836	1915
hsa00190	Oxidative p	98	0.621308	2.735771	0.00165	0.008143	0.003836	3232
hsa00983	Drug meta	69	0.428113	1.772981	0.00165	0.008143	0.003836	3803
hsa05204	Chemical c	69	0.432895	1.792785	0.00165	0.008143	0.003836	4569
hsa01230	Biosynthes	65	0.530593	2.168965	0.001653	0.008143	0.003836	3702
hsa00350	Tyrosine m	33	0.555028	1.974358	0.001656	0.008143	0.003836	4663
hsa00020	Citrate cyc	29	0.766057	2.654095	0.001661	0.008143	0.003836	2824
hsa00630	Glyoxylate	29	0.706501	2.447756	0.001661	0.008143	0.003836	3375
hsa00640	Propanoat	30	0.794009	2.769829	0.001664	0.008143	0.003836	2720
hsa00410	beta-Alani	28	0.662484	2.261554	0.001667	0.008143	0.003836	3135
hsa03320	PPAR signa	68	0.435837	1.789146	0.001669	0.008143	0.003836	2655
hsa04260	Cardiac mu	68	0.439389	1.803726	0.001669	0.008143	0.003836	1102
hsa00900	Terpenoid	22	0.70478	2.280815	0.001692	0.008143	0.003836	4570
hsa04966	Collecting	27	0.586752	1.988947	0.001692	0.008143	0.003836	1593
hsa00062	Fatty acid e	25	0.597268	1.985857	0.001701	0.008143	0.003836	3283
hsa00053	Ascorbate	23	0.701125	2.28669	0.001715	0.008143	0.003836	4074
hsa00790	Folate bios	23	0.615221	2.006518	0.001715	0.008143	0.003836	1866
hsa00340	Histidine m	21	0.687124	2.189629	0.001718	0.008143	0.003836	2836
hsa00650	Butanoate	24	0.802579	2.63302	0.001721	0.008143	0.003836	2593
hsa00970	Aminoacyl	24	0.608552	1.996476	0.001721	0.008143	0.003836	4450
hsa00040	Pentose ar	26	0.683261	2.282377	0.001727	0.008143	0.003836	4074
hsa01210	2-Oxocarb	18	0.67843	2.084903	0.001745	0.008143	0.003836	2640
hsa00061	Fatty acid k	16	0.643727	1.90813	0.001754	0.008143	0.003836	1627
hsa00450	Selenocom	16	0.625435	1.853909	0.001754	0.008143	0.003836	4983
hsa00130	Ubiquinon	11	0.665495	1.75256	0.001812	0.008143	0.003836	1692
hsa05310	Asthma	23	-0.69877	-2.39533	0.002387	0.008143	0.003836	2835
hsa00532	Glycosamir	20	-0.65693	-2.16513	0.002392	0.008143	0.003836	4659

hsa04640	Hematopo	91	-0.57933	-2.69626	0.0025	0.008143	0.003836	2654
hsa04933	AGE-RAGE	99	-0.3763	-1.78846	0.002513	0.008143	0.003836	4129
hsa05142	Chagas dis	97	-0.42537	-2.01083	0.002513	0.008143	0.003836	3029
hsa05330	Allograft re	33	-0.67554	-2.52578	0.002513	0.008143	0.003836	2833
hsa04657	IL-17 signa	86	-0.4651	-2.12941	0.002519	0.008143	0.003836	3656
hsa04658	Th1 and Th	86	-0.55019	-2.51898	0.002519	0.008143	0.003836	3982
hsa05140	Leishmania	74	-0.63122	-2.83112	0.002519	0.008143	0.003836	2833
hsa05323	Rheumatoi	90	-0.47257	-2.19632	0.002519	0.008143	0.003836	2833
hsa04064	NF-kappa	98	-0.5306	-2.51282	0.002525	0.008143	0.003836	2104
hsa04620	Toll-like re	98	-0.46078	-2.18215	0.002525	0.008143	0.003836	4321
hsa04625	C-type lec	98	-0.41178	-1.95007	0.002525	0.008143	0.003836	4043
hsa05146	Amoebiasis	98	-0.43677	-2.06844	0.002525	0.008143	0.003836	4129
hsa05150	Staphyloco	73	-0.6058	-2.70988	0.002525	0.008143	0.003836	3261
hsa04662	B cell recep	79	-0.50666	-2.29862	0.002532	0.008143	0.003836	4043
hsa04659	Th17 cell d	96	-0.55373	-2.60586	0.002538	0.008143	0.003836	3982
hsa04660	T cell recep	96	-0.46881	-2.20622	0.002538	0.008143	0.003836	2176
hsa04610	Compleme	77	-0.42345	-1.91509	0.002551	0.008143	0.003836	2368
hsa04612	Antigen pr	77	-0.41272	-1.86657	0.002551	0.008143	0.003836	2550
hsa04061	Viral prote	87	-0.69586	-3.18899	0.002558	0.008143	0.003836	3577
hsa05235	PD-L1 exp	87	-0.44672	-2.04724	0.002558	0.008143	0.003836	2858
hsa05321	Inflammato	55	-0.60352	-2.52145	0.002558	0.008143	0.003836	4477
hsa04940	Type I diab	41	-0.57786	-2.25072	0.002564	0.008143	0.003836	2833
hsa05133	Pertussis	71	-0.55948	-2.48163	0.002564	0.008143	0.003836	2581
hsa05144	Malaria	47	-0.58845	-2.37223	0.002564	0.008143	0.003836	2956
hsa05332	Graft-versu	41	-0.6379	-2.48455	0.002564	0.008143	0.003836	4490
hsa05340	Primary im	34	-0.76651	-2.86824	0.002564	0.008143	0.003836	2319
hsa04672	Intestinal in	42	-0.66909	-2.62229	0.002571	0.008143	0.003836	3982
hsa05416	Viral myoc	58	-0.52835	-2.23202	0.002571	0.008143	0.003836	3106
hsa04670	Leukocyte	105	-0.39572	-1.88854	0.002584	0.008143	0.003836	3250
hsa05134	Legionellos	57	-0.40362	-1.69492	0.002591	0.008143	0.003836	3029
hsa05322	Systemic lu	48	-0.6647	-2.68537	0.002591	0.008143	0.003836	3982
hsa05320	Autoimmu	44	-0.63699	-2.51753	0.002597	0.008143	0.003836	4490
hsa05162	Measles	132	-0.44211	-2.19164	0.002604	0.008143	0.003836	4604
hsa04623	Cytosolic D	56	-0.43351	-1.81421	0.002611	0.008143	0.003836	4284
hsa04630	JAK-STAT s	139	-0.37938	-1.90376	0.002625	0.008143	0.003836	2905
hsa05135	Yersinia inf	130	-0.38528	-1.90397	0.002625	0.008143	0.003836	2176
hsa04650	Natural kill	119	-0.4846	-2.36414	0.002632	0.008143	0.003836	4521
hsa04514	Cell adhesi	136	-0.48062	-2.3977	0.002639	0.008143	0.003836	2683
hsa04210	Apoptosis	129	-0.35008	-1.72214	0.00266	0.008143	0.003836	4124
hsa04668	TNF signal	108	-0.53497	-2.55719	0.00266	0.008143	0.003836	4124
hsa05145	Toxoplasm	108	-0.38942	-1.86141	0.00266	0.008143	0.003836	3042
hsa04145	Phagosom	144	-0.37088	-1.8601	0.002667	0.008143	0.003836	2833
hsa04380	Osteoclast	122	-0.53902	-2.62655	0.002688	0.008143	0.003836	4043
hsa04611	Platelet act	114	-0.35258	-1.70831	0.002703	0.008143	0.003836	4217
hsa05161	Hepatitis B	157	-0.40529	-2.06719	0.002703	0.008143	0.003836	4321
hsa05152	Tuberculos	171	-0.3932	-2.02278	0.002732	0.008143	0.003836	2870
hsa05202	Transcripti	158	-0.3643	-1.85831	0.002732	0.008143	0.003836	3824
hsa04621	NOD-like r	161	-0.40936	-2.1024	0.002778	0.008143	0.003836	4604
hsa05203	Viral carcin	161	-0.35034	-1.79924	0.002778	0.008143	0.003836	4043
hsa05164	Influenza A	162	-0.45071	-2.31327	0.002793	0.008143	0.003836	4604
hsa05206	MicroRNAs	163	-0.33326	-1.70467	0.002793	0.008143	0.003836	4176
hsa04062	Chemokine	177	-0.48329	-2.49036	0.002833	0.008143	0.003836	3454
hsa05167	Kaposi sarc	175	-0.37843	-1.94959	0.002833	0.008143	0.003836	4043
hsa05169	Epstein-Ba	194	-0.44505	-2.32447	0.002865	0.008143	0.003836	4321
hsa05171	Coronaviru	195	-0.50253	-2.62665	0.002865	0.008143	0.003836	4321
hsa05166	Human T-c	211	-0.44099	-2.35038	0.002882	0.008143	0.003836	4191
hsa05163	Human cyt	210	-0.33152	-1.76307	0.00289	0.008143	0.003836	4490
hsa05170	Human im	191	-0.32016	-1.66805	0.00289	0.008143	0.003836	4359

hsa05132	Salmonella	244	-0.3184	-1.72742	0.002994	0.008363	0.003939	3444
hsa04060	Cytokine-c	249	-0.50624	-2.73626	0.003096	0.008573	0.004039	3577
hsa04010	MAPK sign	274	-0.28457	-1.55855	0.003125	0.008581	0.004042	4267
hsa04150	mTOR sigr	146	0.334328	1.559727	0.00321	0.008741	0.004118	4598
hsa04973	Carbohydr	38	0.464181	1.699776	0.003284	0.008806	0.004148	1771
hsa00760	Nicotinate	31	0.511954	1.794551	0.003295	0.008806	0.004148	4101
hsa04130	SNARE inte	30	0.521137	1.817938	0.003328	0.008806	0.004148	2190
hsa01040	Biosynthes	25	0.54786	1.82158	0.003401	0.008806	0.004148	3283
hsa00220	Arginine b	20	0.598656	1.88413	0.003425	0.008806	0.004148	4357
hsa00770	Pantothena	20	0.609998	1.919825	0.003425	0.008806	0.004148	3245
hsa04964	Proximal tu	20	0.610665	1.921925	0.003425	0.008806	0.004148	5384
hsa05168	Herpes sim	427	-0.26018	-1.49432	0.003472	0.008858	0.004173	4614
hsa05200	Pathways i	492	-0.26414	-1.54082	0.003846	0.009736	0.004586	2905
hsa00140	Steroid hor	50	0.430555	1.672265	0.004902	0.012217	0.005755	4663
hsa00860	Porphyrin a	34	0.503666	1.810861	0.004902	0.012217	0.005755	5555
hsa04977	Vitamin dig	20	0.553202	1.741076	0.005137	0.012631	0.00595	1726
hsa03430	Mismatch r	23	0.522505	1.704129	0.005146	0.012631	0.00595	2322
hsa05160	Hepatitis C	144	-0.30988	-1.55414	0.005333	0.012992	0.006121	4550
hsa04360	Axon guida	173	-0.26974	-1.38987	0.00551	0.013322	0.006276	4082
hsa05130	Pathogenic	184	-0.32396	-1.68287	0.005682	0.013553	0.006385	3581
hsa04510	Focal adhe	196	-0.26906	-1.41005	0.005714	0.013553	0.006385	3374
hsa04015	Rap1 signa	194	-0.2801	-1.46296	0.005731	0.013553	0.006385	2981
hsa05205	Proteoglyc	199	-0.26956	-1.41484	0.005814	0.01365	0.00643	2981
hsa04910	Insulin sigr	133	0.315188	1.454589	0.006525	0.01521	0.007165	5126
hsa00360	Phenylalan	17	0.574231	1.739019	0.00692	0.016016	0.007545	4569
hsa04664	Fc epsilon	63	-0.38016	-1.64153	0.0075	0.017208	0.008106	2176
hsa05221	Acute mye	65	-0.39944	-1.7389	0.007557	0.017208	0.008106	2156
hsa04666	Fc gamma	95	-0.34185	-1.6035	0.007595	0.017208	0.008106	2215
hsa04217	Necroptos	124	-0.31047	-1.517	0.008108	0.018243	0.008594	3573
hsa05131	Shigellosis	209	-0.25501	-1.3566	0.008475	0.018936	0.008921	2185
hsa00500	Starch and	32	0.468059	1.654066	0.011551	0.025634	0.012076	3245
hsa05220	Chronic my	76	-0.32416	-1.45955	0.012658	0.0279	0.013143	2156
hsa00730	Thiamine r	14	0.595335	1.694462	0.014159	0.030997	0.014602	2325
hsa04218	Cellular ser	148	-0.28837	-1.46357	0.015464	0.033626	0.015841	4359
hsa03013	RNA transp	152	0.29946	1.405613	0.015924	0.034395	0.016203	6074
hsa05222	Small cell l	90	-0.30794	-1.43118	0.017632	0.037833	0.017823	4550
hsa04922	Glucagon s	96	0.32495	1.426871	0.018092	0.038565	0.018167	2485
hsa05165	Human pa	316	-0.22651	-1.26373	0.019108	0.040465	0.019062	3374
hsa04512	ECM-recep	82	-0.3159	-1.43592	0.020408	0.042937	0.020227	3607

leading_edcore_enrichment
tags=32%, 1349/7386/4717/2911/54205/4720/29796/6390/9377/4706/4722/1537/292/10105/291/
tags=36%, 1349/7386/4717/54205/4720/29796/4023/6390/9377/4706/4722/1537/292/10105/291/
tags=34%, 1349/7386/4717/54205/4720/29796/6390/9377/4706/4722/1537/6389/4702/65018/471
tags=38%, 1349/7386/4717/10891/54205/4720/29796/6390/9377/4706/4722/3766/1537/292/1010
tags=36%, 1349/7386/4717/54205/4720/29796/6390/9377/4706/4722/1537/292/10105/291/6389/
tags=31%, 1349/7386/4717/54205/4720/29796/6390/9377/4706/4722/1537/292/10105/291/6389/
tags=36%, 1349/7386/4717/10891/4720/29796/493753/6390/9377/4706/4722/1537/6389/5563/47
tags=24%, 10891/5563/5862/2538/51422/1994/7248/32/5208/3643/55844/5468/6009/2203/3156/
tags=61%, 2805/38/3418/414328/8801/6390/84693/26007/8803/8802/128/6389/26275/1738/275/
tags=41%, 2911/54205/292/10105/291/10939/1600/3708/115209/5701/3709/8678/5717/7416/570
tags=53%, 2235/4338/51805/217/84274/9054/5805/204/1738/8566/210/53354/51004/54995/1101
tags=41%, 1349/7386/4717/54205/4720/29796/6390/9377/4706/4722/1537/6389/5563/4702/4715
tags=29%, 4717/2911/4720/4706/4722/2788/4702/3760/4715/2560/3708/10681/3709/4705/4714/
tags=46%, 38/217/3033/1738/501/224/55526/51166/2639/1892/1962/123688/10157/39/219/9869
tags=51%, 3945/217/128/1738/2538/501/1737/224/5162/84532/130589/5230/2597/2203/2023/51
tags=74%, 1491/9380/1738/23464/275/501/29958/4129/6470/189/2653/29968/4128/1610/1757/6
tags=67%, 2805/217/501/4129/224/6611/84735/112849/8974/5625/112483/4953/4942/4128/1610
tags=53%, 128/4129/2947/1576/2948/9446/2940/2330/4128/2329/130/4259/2938/54576/2941/54
tags=67%, 38/34/36/10455/217/33/3033/128/10449/501/224/1632/3032/2639/2181/2180/30/1892
tags=71%, 2805/339983/2346/7915/2746/189/2875/2747/443/18/2571/8528/56954/445/64902/54
tags=67%, 2805/3945/1491/55256/4191/6611/27430/58478/4144/29968/191/23743/51074/4507/4
tags=78%, 5019/56922/549/38/34/36/217/84693/3712/27034/594/11112/3033/26275/1738/10449
tags=68%, 38/217/3033/1738/501/4129/224/55526/1644/51166/2639/8942/847/4128/1892/1962/
tags=56%, 128/1551/9249/1576/216/145226/8228/157506/10901/317749/51109/130/56603/5457
tags=45%, 760/358/476/481/1576/9429/1244/3781/8431/10864/3156/10998/107/6523/5243/5457
tags=40%, 4023/217/26007/501/80339/224/2710/56894/129642/219/1607/116255/10327/253558/
tags=43%, 873/128/27294/2947/1576/2948/9446/2940/22977/130/4259/2938/54576/8574/2941/5
tags=49%, 38/34/36/33/3033/10449/54995/84869/3032/2181/2180/6342/30/1892/27349/1962/81
tags=70%, 3418/283927/5191/10455/2053/5827/3155/55825/3417/83594/8309/6647/189/5189/21
tags=51%, 3418/3417/124975/2947/6611/79017/2948/9446/4953/2940/26873/2729/4259/2938/62
tags=64%, 38/3945/217/9380/1738/501/2271/1737/4191/224/3029/10873/5162/32/2739/84532/1
tags=54%, 1349/7386/4717/4720/29796/6390/9377/4706/4722/1537/6389/245973/4702/4715/52
tags=48%, 151531/2947/83549/1576/2948/9446/2940/7172/9/4259/2938/7372/6240/54576/2941/
tags=48%, 873/128/1551/2947/1576/2948/9446/2940/9/130/4259/2938/54576/2941/54577/54600
tags=52%, 2805/3418/1491/3420/3417/6470/2875/27430/4144/29968/50/95/5230/2597/729020/2
tags=61%, 7306/2805/128/4129/1644/4282/4128/130/81889/3081/2184/220074/5409/316/1621/2
tags=79%, 3418/8801/6390/8803/8802/6389/1738/3420/2271/1737/3417/4191/5162/4967/50/639
tags=76%, 38/84693/9380/1738/275/4191/6470/189/5096/84532/2653/50/847/51179/4190/39/48
tags=80%, 38/3945/8801/84693/8803/594/8802/26275/1738/79611/1629/32/5096/84532/55862/4
tags=61%, 217/26275/501/224/84735/4329/1892/18/2571/1962/51/219/3030/8310/223/51733/18
tags=34%, 34/4023/33/10873/8309/2710/2181/2180/6342/5468/30/1962/81616/51/10998/11001/
tags=19%, 1349/7386/783/29796/9377/1537/476/1327/7385/481/1351/1329/7384
tags=82%, 38/51449/3422/57107/3156/39/79947/23463/4598/2224/116150/2342/2339/10654/235
tags=44%, 1188/245973/760/6521/529/245972/50617/127124/523/51382/526/528
tags=56%, 3033/10449/3032/122970/11332/1892/641371/10965/79993/51102/117145/3030/5114
tags=83%, 217/501/224/55586/9365/219/10327/54576/54577/54600/223/54578/54575/54659/545
tags=43%, 873/4338/5805/249/84105/10243/1719/5053/8836/6697
tags=62%, 217/501/4129/224/84735/443/138199/4128/219/144193/26/10841/223
tags=79%, 5019/38/622/79944/3033/3155/56898/65985/6296/7915/1892/18/2571/1962/39/3030/
tags=71%, 5188/10667/51091/79731/55699/124454/55157/23438/80222/23395/57038/55278/219
tags=77%, 51181/27294/51084/6652/9942/729020/9365/6120/10327/54576/54577/54600/54578/
tags=61%, 2805/3418/3420/3417/2875/51166/50/95/162417/48/3419
tags=56%, 54995/32/84869/2181/2180/27349/81616/7923/51102
tags=69%, 22929/1491/51091/22928/4548/10587/51540/883/56267/118672/92935
tags=36%, 51805/84274/51004/10229
tags=83%, 7124/3125/3127/2205/3118/3117/3126/3123/6356/3113/958/3119/3115/2207/959/310
tags=70%, 26229/126792/29940/337876/64132/113189/55501/55790/51363/22856/56548/64131/

tags=53%, 910/3559/3570/4254/3117/3126/3123/1604/929/913/947/3113/3690/3552/1438/3553/9
tags=40%, 7043/5331/5293/10000/4087/8503/4772/3569/6777/2308/5333/6774/7040/5054/1284/
tags=39%, 841/2774/7040/8772/148022/3460/5054/355/5603/5594/7042/4792/5970/5332/2769/2
tags=79%, 356/3593/7124/3125/3127/3118/3117/3126/3123/355/3134/3002/3113/5551/3133/942
tags=47%, 4314/3569/7188/6354/8061/841/1673/7187/3627/8772/2921/7184/4318/5598/6356/56
tags=56%, 3125/3127/4772/30009/5534/5530/6777/55534/3118/3559/3117/3126/3123/3460/3595
tags=53%, 3118/7040/3117/3126/3123/3460/5603/5594/7042/4792/5970/6772/1536/3689/3113/3
tags=48%, 3118/7040/2921/51561/3117/3126/3123/1493/7042/3689/3113/3606/3552/4050/8992/
tags=38%, 4792/5335/5970/929/6351/4050/9020/3553/8792/958/597/695/6363/5743/4067/6357/9
tags=49%, 7124/3452/3439/3447/7100/5293/10000/3661/1513/8503/3569/3448/3654/4283/841/7
tags=46%, 5293/10000/338339/4193/1540/8503/4772/4791/5534/3569/5530/6367/841/868/51561
tags=47%, 22798/3593/7124/7043/5331/5293/3911/8503/735/3569/338382/7414/10319/2774/391
tags=56%, 720/721/1673/3118/5724/3117/3126/125115/2266/3123/3689/3859/3113/2359/3872/1
tags=46%, 5293/10000/353514/8503/4772/5534/5530/11027/971/10990/11006/5594/4792/5970/9
tags=59%, 3125/3127/4087/4772/30009/5534/3569/5530/6777/6774/3118/7040/3559/51561/3570
tags=32%, 5603/1493/5594/5788/4792/5335/5970/29851/9020/3845/940/1326/925/5063/916/479
tags=40%, 2266/2161/2243/5054/7056/1604/3689/2244/1675/10878/716/629/3687/11326/10877/
tags=36%, 3824/3117/3126/3123/3134/1520/10437/3113/3821/3822/972/3133/6891/3107/4261/3
tags=67%, 3569/1435/1233/6370/6355/4283/6354/8795/7852/6367/3627/2921/29949/8764/3559/
tags=38%, 6774/116071/148022/1460/3460/4215/10538/5603/5594/4792/29126/5335/5970/6772/
tags=75%, 3593/7124/7100/7043/3125/3127/4087/4772/30009/3569/6774/3118/7040/51561/3117
tags=54%, 3118/3117/3126/3123/355/3134/5799/3002/3113/3552/5551/3553/3133/942/3107/940
tags=48%, 51561/148022/1072/5603/5594/5970/929/3689/114548/3552/836/716/3553/2771/3659
tags=55%, 2995/7040/5175/4233/1311/7042/975/3689/3606/3553/958/7097/6403/6401/22914/63
tags=71%, 356/7124/115653/3125/3127/3569/3118/3824/3117/3126/3123/355/3134/3002/3113/3
tags=65%, 64421/5788/973/29851/6891/930/958/4261/925/695/916/84876/959/920/100/7535/354
tags=67%, 3125/3127/3569/6370/7852/3118/7040/3117/3126/3123/56477/3113/29851/5284/9020
tags=53%, 8672/841/637/3118/3117/3126/3123/857/1525/3134/1604/3689/3113/836/5551/3133/9
tags=37%, 5906/7414/7852/1500/6494/5175/4318/58494/5603/5335/10627/1536/83700/3689/426
tags=40%, 841/1937/58484/2921/4792/5970/929/81876/3689/3606/836/3553/7097/834/317/3310
tags=71%, 3125/3127/735/2215/720/721/733/3118/3117/3126/3123/3113/1511/716/942/958/940/
tags=70%, 356/3452/3439/3447/1081/3125/3127/3448/3118/7253/3117/3126/3123/355/3134/149
tags=53%, 51135/1019/1965/51209/356/3593/3452/3439/3447/5293/10000/898/3661/8503/23586
tags=46%, 3452/3439/3447/51082/3661/23586/3569/3448/10622/3627/84265/90865/103/4792/59
tags=31%, 5156/6774/29949/3559/51561/3570/5159/5154/5771/3460/85480/3595/6778/6772/677
tags=28%, 5603/5594/4792/5335/5970/114548/3606/2776/9815/3553/7456/925/834/6347/10094/
tags=50%, 117157/25759/80328/6464/356/7124/3452/3439/3447/5293/115653/8503/4772/51744/
tags=40%, 5175/57863/3117/3126/3123/58494/23114/3134/1493/5788/29126/22829/947/83700/3
tags=42%, 4217/5293/10000/1513/8503/71/142/472/578/8739/113457/112714/8795/841/637/727
tags=52%, 4217/8986/5293/10000/8503/9252/4314/3569/7188/1435/841/90993/7187/3627/8772/
tags=36%, 10319/841/6774/3914/3118/7040/3117/3126/3123/3460/5603/5594/3915/7042/4792/5
tags=37%, 3118/7278/5869/6441/4360/4481/3117/3126/3123/1311/3134/1520/929/1536/3689/31
tags=53%, 5293/10000/1540/1513/353514/8503/4772/4791/5534/5530/2215/1435/8061/11027/70
tags=38%, 5742/5908/5331/5293/10000/5592/8503/71/9138/10672/7094/5906/5321/5023/2266/1
tags=42%, 356/6554/7124/3452/3439/3447/4214/7043/5293/10000/898/3661/8503/4772/23586/3
tags=34%, 637/3118/7040/8772/5869/51561/4360/3117/3126/3123/7096/3460/5603/817/5594/70
tags=39%, 8842/5546/5090/4314/472/3569/84444/1031/578/2308/6929/2005/6692/2078/2313/67
tags=48%, 51135/79792/64170/115361/22900/7158/7124/3452/5585/3439/3447/5331/3661/7295/
tags=37%, 5293/898/4193/3661/8503/7531/4791/7188/1233/578/5315/6777/5610/841/90993/718
tags=54%, 51135/1019/1965/356/3593/7124/3836/3452/9230/3439/3447/5293/10000/3661/3125/
tags=39%, 54541/4854/5728/5293/11186/898/4193/4325/8503/6541/659/9252/7329/995/472/672
tags=44%, 1233/6370/57580/6355/6777/4283/6354/5906/7852/408/6367/10663/7074/6774/3627/
tags=41%, 5293/10000/3661/59345/8503/4772/2247/131450/5534/3569/3448/5530/1233/578/575
tags=49%, 7124/6502/3452/3439/3447/5293/10000/898/4193/3661/3125/3127/8503/5713/4791/2
tags=52%, 7124/3452/3439/3447/6159/6146/5293/3661/8503/6868/4312/6227/735/23586/4314/3
tags=47%, 4214/5728/7043/5293/10000/898/3125/3127/4087/8503/4772/4791/64784/4487/5534/
tags=41%, 356/3579/108/1230/7124/3452/6667/3439/3447/5331/5293/9826/10000/4193/3661/59
tags=41%, 8945/7124/9133/3452/3439/3447/140564/5293/10000/3661/59345/8503/4772/5534/99

tags=31%, 578/3654/89953/113457/112714/338382/8795/3800/841/6188/8655/58484/127829/72
tags=46%, 3569/3448/1435/1233/6370/6355/1442/4283/6354/8795/7852/3557/6367/3625/10663/
tags=35%, 2322/2249/5908/4214/7043/4217/8986/10000/5923/3164/409/4772/2247/4791/9252/5
tags=34%, 58528/5563/245973/8649/1147/529/7248/57600/10542/3643/127124/523/51382/2733
tags=32%, 6518/2538/476/481/8972/2542/276/277/278/279/6523/280
tags=58%, 23410/23530/83594/23409/4860/349565/133686/64802/23475/683/93100/54981/316/
tags=37%, 53407/9570/10490/6845/6809/9341/9527/8417/10652/8773/10282
tags=52%, 6342/122970/30/11332/51/641371/10965/79993/3295/79966/8310/51144/51495
tags=60%, 2805/2746/2875/2747/95/162417/384/445/435/2806/84706/2744
tags=50%, 217/53354/224/79717/80347/219/79646/51733/1807/5169
tags=70%, 760/358/476/481/2746/2747/4190/5106/5105/486/2744/1468/27165/482
tags=32%, 148268/51135/55769/1965/64170/356/7710/3593/349075/92595/29992/7124/3452/34
tags=25%, 5156/5228/637/7187/6774/7483/3914/8324/7040/7428/2768/8772/80326/57121/3559/
tags=48%, 1551/1576/1586/7923/3291/79154/54576/220074/54577/54600/3294/374875/54578/5
tags=68%, 2235/210/326625/124454/7389/54576/54577/54600/644/54578/54575/54659/54579/2
tags=35%, 25974/2346/686/9963/113235/5948/8029
tags=35%, 5395/4437/6119/5981/10714/5985/2956/4292
tags=39%, 1019/1965/356/7124/3452/3439/3447/5293/10000/3661/6041/91543/8503/7531/10197
tags=34%, 56963/5293/1948/8503/64221/6586/5361/659/2048/5534/85464/2044/5530/91653/747
tags=35%, 8976/3569/578/3654/4644/10672/113457/112714/8795/8440/841/6188/5062/10163/72
tags=27%, 3371/8516/7094/5906/7414/3611/131873/10319/5062/858/5156/5228/3914/8515/564
tags=28%, 7074/5156/5228/1500/6494/10636/57121/57568/4233/5159/4254/5154/196883/5603/5
tags=27%, 7074/858/6774/7483/8324/7040/3339/80326/7078/4233/4318/355/857/5603/817/5594
tags=35%, 10891/5563/2538/51422/7248/32/801/3643/5573/8835/9470/6009/1399/2203/6720/81
tags=59%, 2805/4129/1644/4282/4128/5053/2806/10249/3242/221
tags=27%, 5603/5594/5335/3845/8605/241/240/3635/695/4067/2207/7409/9846/27040/3937/220
tags=26%, 5594/862/5970/929/6776/3845/8900/11040/597/890/2209/3684/6932/1436/5371/1848
tags=27%, 1072/5594/5788/5335/7408/8605/3635/2209/4067/4082/3055/10094/2212/653361/557
tags=40%, 7124/3452/3439/3447/1540/91782/142/7188/3448/29082/6777/5321/5610/8795/841/5
tags=21%, 3099/5603/5594/4792/5335/5970/929/10627/9181/5332/837/7322/114548/3606/1039
tags=41%, 2538/8972/2632/276/277/278/279/280/5236/57733/178/80201/5169
tags=32%, 7043/5293/10000/4193/8503/578/6777/7040/5594/7042/4792/5970/6776/3845/39969
tags=43%, 9054/204/52/84284/249/205
tags=36%, 8945/2305/9133/5728/7043/5293/10000/898/4193/4087/8503/4772/5534/472/3569/55
tags=45%, 26999/59343/11102/10284/9470/7341/65110/9939/10419/6396/2332/8891/1977/6510
tags=39%, 1019/22798/6502/5728/5293/10000/898/3911/8503/3685/7188/578/10319/7187/3914/
tags=21%, 3945/10891/5563/2642/2538/51422/3708/3709/5162/32/801/2203/5160/1387/5213/81
tags=24%, 3371/5315/8516/2308/5610/7474/55534/5529/131873/10319/841/3955/90993/7187/74
tags=35%, 3685/1758/3371/8516/131873/10319/3914/3339/8515/9900/5649/1292/9899/1311/71

6389/4702/65018/4715/3708/5701/1327/7385/3709/6647/347733/5861/4705/5621/10671/801/728
6389/4702/4715/1147/3708/5701/1327/7385/3709/347733/4705/801/7280/3643/8678/1351/4714/
5/5701/1327/7385/6647/347733/5861/4705/10671/7280/8678/1351/4714/4700/4712/1329/4724/4
5/291/6389/4702/4715/3708/5701/1327/7385/6647/347733/4705/10671/7280/8678/1351/4714/47
4702/65018/4715/3708/5701/1327/4129/7385/3709/347733/4705/801/7280/1351/4714/4700/4712
3306/4702/4715/3708/5701/1327/7385/3709/6647/347733/4705/5621/7280/1351/4714/4700/4712
02/4715/51422/51103/1327/7385/79133/7248/4705/1351/4714/4700/4712/2181/29078/2180/132
6720/5516/55437/5213/79602/5564/51094/51719/5170/79966/5520/3172/5106
3420/2271/1737/3417/4191/6470/10873/5162/2746/189/2875/5096/84532/2747/2653/4329/2996
5/5683/342371/6095/5718/7436/55102/5702/7419/5602/23369/5601/5706/9698/293/5599/488/9
9/224/6470/9249/79717/27430/50808/4144/326625/8942/145226/349565/29968/133686/249/102
/51422/1327/7385/4705/3643/6945/1351/4714/4700/4712/51085/1329/4724/4711/4704/4698/73
4700/4712/4724/4711/4704/4698/2555/4728/107/57406/4695/4723/126328/222236/4694/4713/4
/51111/3030/223/9739/10919/84787/51268/8424/29072/79813/2145
60/130/83440/5213/219/5313/5236/10327/9562/229/5106/80201/7167/223
35/2628/132158/55349/5723/64902/51268/2593/2731/63826/6472
/58510/79814/219/2628/384/26/223/1152/113451/112817/2593/8659/29920/2806/51056/262/50
577/54600/119391/316/54578/54575/54659/54579/4257/54658/54657/54490/1555/221/2326
/1962/81616/51/130/39/219/1376/3030/8310/223
71/80150/435/8659/2806/84706/2744/2673/158
548/2729/635/4190/10768/7263/4357/64902/883/2730/2806/262/56267/84245/2937/113675/160
/501/1629/3155/65985/224/5096/3032/4329/30/1892/18/1962/39/219/3028/3030/5095/223/316/
169355/8564/39/219/64577/3030/26/130013/223/316/883
/54577/54600/316/54905/54578/54575/54659/54579/54884/54658/54657/54490/1555/8854
6/54577/54600/200931/5244/6555/54578/54575/54659/54579/6256/9376/486/6580/123264/5465
9663/132158/8613/55750/84513/223/1056/3990
4577/54600/119391/374875/54578/54575/54659/54579/4257/54658/54657
616/51/79993/3295/7923/51102/39/1376/79966/3030/8310
81/8504/2180/6342/5264/26061/30/847/51179/5830/1610/1962/10901/5825/51/5193/1891/3295/
240/5226/9588/2941/51471/119391/2936/2730/9027/4257/50484/51056/2880
34526/5160/197257/4190/39/219/5313
/1327/7385/245972/4705/50617/1351/4714/4700/127124/4712/1329/4724/4711/523/51382/4704
54577/54600/119391/51733/8824/54578/1807/54575/54659/54579/10/54963/1854/4257/2990/54
/119391/374875/54578/6799/54575/54659/54579/10/883/4257/54658/54657/56267/54490/6818/
023/4548/5053/162417/5213/6120/5313/5631/48/384/229/3419/7167/5723/445/435/5634/29920/
806/3242/2954/221/1312
2/47/5160/4190/48/5106/3419/55753
/132158/5095/112817/54363/2731
329/1892/18/1962/51/39/3030/8310/5095
07
345/1376/5170/79966/364/5106/8310

90/4597/10269/3157

4/51495
79/2990/54658/54657/54490

54988/123876/348158
3/123263/10352/54938/118672
54575/54659/54579/2990/54658/54657/54490

8/3109/3112/2206
54480/50515

930/3119/952/3563/3115/1441/2209/925/2323/3574/916/960/951/931/917/3554/3108/3678/3109/
7056/5603/5594/7042/5335/5970/6772/6776/1536/5332/3552/836/3845/581/3553/6401/6347/431
775/2776/3553/2771/6348/6349/414062/7097/916/6347/919/811/6352/917/713/915/7132/714/23
/958/3107/940/3119/3115/3105/959/3108/3109/3112
03/5594/4792/5970/2354/836/3553/9641/6279/5743/6347/3727/23765/6374/6364/10758/2353/62
/5603/5594/6778/4792/5335/5970/6772/6776/3113/9794/3594/3119/3560/3115/916/4794/919/91
552/9103/3553/3119/3115/2209/7097/5743/4688/2212/653361/3108/5579/3109/5777/3684/3112/
3553/942/8792/940/3119/3115/6348/6349/414062/7097/6347/6352/3108/3109/8600/3683/10673/
959/3554/5579/5971/8600/7535/10673/3932/27040/7132/7185/6366/23643/4615/3383/7128/7412
187/3663/3627/8772/148022/7096/5603/5594/4792/5970/6772/6373/929/6351/3553/942/958/132
/5603/5594/4792/5970/6772/114548/64581/9020/3845/3710/4046/3553/3659/1960/9641/5743/26
4/733/7040/2921/5869/910/1284/3915/7042/5970/929/913/3689/5332/2769/2776/836/3315/1511
675/25984/716/9103/3119/629/3115/2209/6403/2212/3108/728/3109/713/3684/3683/6404/3112/
975/973/11024/3845/10859/930/3635/695/10288/4067/4794/11025/5579/7409/5777/4773/2213/23
/3117/3126/3123/3460/5603/5594/6778/4792/5335/5970/6772/6776/3572/3113/3594/3553/3119/
4/919/959/917/7409/920/7535/5777/915/3932/27040/3937/3702/4773/2353/3725
2/1191/3078/728/713/3684/5329/3075/3080/714/715/717/718/7450/729/710
119/3115/925/3105/567/3310/811/5721/3108/3109/920/6890/3112
3570/6356/56477/6373/6359/6351/3572/1524/8809/10563/3606/6362/5197/3560/6846/6348/634
/3845/940/55509/7097/3091/916/4794/919/917/920/7535/5777/915/3932/27040/4773/2353/4615/
/3126/3123/3460/3595/6778/7042/5970/6772/3113/8809/3606/3552/3594/64127/3553/3119/311
/3119/3115/3105/3108/3109/3112
/834/3394/10392/3678/713/3684/6374/714/2353/715/717/23643/4615/718/3725/3576/29108/710
47/959/3082/3683/3820/7058/4615/3383/7412/3576/2532
552/3821/5551/3553/3133/942/3107/940/3119/3115/3105/3108/3109/3112
43/915/3718/3932/6890/3575
/942/958/940/3119/3115/959/3108/3109/10673/3601/3112/3676/608
942/958/3107/940/3119/3115/3105/959/3108/60/3109/3683/3112/3383/5880
7/4478/7408/10398/2771/1365/1364/4688/4313/653361/60/5579/7409/399/3684/3683/3702/367
/3684/4615/718/3576/29108/2920/2919
/3119/3115/2209/2903/2212/959/3108/3109/713/6737/3112/714/715/717/718/729/87
3/3002/3113/5551/3133/942/958/3107/940/3119/3115/3105/959/3108/3109/3112
/3569/3448/578/3654/6777/5610/841/868/637/7187/6774/4940/8772/3559/103/9451/1460/355/4
970/6351/3606/11277/3553/9641/834/6352/11035/9447/3665/29108
6/3572/1438/3594/3560/3563/10401/1441/338376/9180/3574/9466/10379/3976/5777/3718/5061
2212/60/3678/7409/920/7535/3932/27040/7454/3937/9844/4773/3676/2353/10095/4615/3725/10
5534/3448/5530/2215/3823/8795/3384/637/3824/4277/3460/355/5594/4068/5335/3002/3689/83
689/4267/3113/29851/57689/9019/80380/3133/942/958/3107/940/3119/3115/925/1365/201633/3
8/8772/9451/355/5594/4792/1520/5970/3002/332/9020/836/3845/3710/1509/581/5551/10376/53
2921/4318/355/5603/5594/4792/5970/4323/8809/9020/836/197259/64127/3553/1326/3659/5743/
970/6772/3113/2775/836/2771/958/4261/3119/240/3115/7097/1234/3310/959/3108/3109/3112/7
13/3690/64581/203068/8992/23480/9103/10376/84617/3133/6891/3107/3119/3115/2209/7097/9
040/126014/10326/10990/11006/3460/5603/5594/7042/4792/5970/6772/11024/54209/2354/3690/
96883/2243/5603/5594/10627/5332/2244/7408/3690/2776/8605/3710/2771/695/4067/64805/848
569/3448/3654/6777/841/90993/637/7187/6774/7040/3339/8772/148022/4318/355/5603/5594/67
42/1520/5970/6772/929/3689/3113/3606/3552/64581/836/4046/1509/581/9103/64127/972/3553/
56/4233/25942/2115/4318/5154/8464/5081/862/5970/929/3002/4297/604/8900/5079/64919/581/
6041/7531/4210/3569/7188/3448/5027/10628/841/55072/1673/58484/7187/4940/8772/2633/292
7/6774/9519/23352/3134/27044/5594/4792/5970/6776/3572/836/3845/8900/5925/581/3133/536
3127/6041/91543/8503/71/8766/23586/3569/3448/578/5610/8795/841/637/7187/4940/3118/362
/578/3371/1786/3162/5156/6774/7078/4233/5159/4318/5154/5598/7148/5594/7042/9839/5335/2
2921/196883/6356/5594/4792/56477/5335/5970/6772/6373/6359/6351/2782/5332/1524/10563/2
580/5610/841/637/7187/6774/8772/2921/148022/4277/355/5603/3134/5594/4792/5335/5970/677
3586/3569/7188/3448/578/3654/11047/5610/841/637/7187/6774/4940/3118/3627/8772/25942/31
569/3448/3654/6134/23521/720/721/5610/6138/6188/2162/6158/7187/6774/4940/733/3627/103/
472/3569/1031/5530/6777/7094/8061/11200/90993/6929/2005/3118/7040/701/996/9519/3559/31
345/8503/4772/9138/5534/3685/3569/7188/3448/5530/578/10672/7852/841/90993/5156/637/677
5/472/7188/3448/200315/5530/578/3654/7852/841/5062/637/85363/8772/891/3985/355/1072/56

78/8772/5869/1639/7184/3798/64837/5603/5594/5788/4792/5970/929/10627/6993/837/23207/11
94/9966/8784/8742/3627/7040/10148/2921/29949/8764/90865/3559/51561/3570/130399/6356/34
534/2872/5530/1435/3654/9064/59285/5906/5321/408/775/5062/5156/2005/5228/7040/1843/276
0/220441/9470/6009/526/528/55437/10641/8140/8322/6396/79726/51719/5170/1977/79109/9663

5169/554235/56953/4907/23408

39/3447/10224/147948/7700/146540/162993/148156/5293/121274/58500/10000/3661/3125/3127/
64399/51561/4233/3570/5159/7184/4254/4318/2949/5154/1909/196883/3460/355/3595/1284/81
1144/54575/54659/54579/6716/54658/54657/8644/54490/1585/1312
990/54658/54657/3163/54490/7390/3145/645/211/79799

7/23586/3448/578/5610/949/841/637/7187/6774/4940/3627/8772/148022/9451/355/5594/4792/59
74/7852/3611/8440/5062/219699/10154/10505/57522/10371/10509/3985/4233/1949/7223/1072/8
278/2768/8772/57121/355/5603/5594/4792/5970/9181/1902/9871/837/114548/3606/836/203068/
9/4233/5159/1292/5154/1311/857/7148/1284/5594/3915/55742/10627/3694/7408/3690/10398/39
594/25865/5335/3689/2252/5332/1902/1945/7408/2775/3690/2776/3845/9170/5898/2771/51466/
/7042/117581/3316/5335/4478/3690/836/3845/3710/4060/10855/2260/7097/3091/7482/960/4313
0/5313/5564/5170/1977/91860/90673/5106/1398/5602/80201/5501/5601/5599/8660/5105/5590/2

6/5880
/4609
79/7409/9846/8612/27040/7454/8877/2213/10095/10109/5880/65108
5072/124044/637/6774/8772/90865/148022/5478/3460/355/5834/817/6778/6772/6776/1536/583
3/3710/581/3553/8767/11337/834/10094/960/9266/6352/5216/3554/10392/60/3678/10616/7335/7

4/5925/581/867/9846/1871/7157/1026/1643/4609

30/2308/11200/7040/891/678/55957/5054/5603/3134/5594/7042/5970/3552/3845/8900/3710/592
9/10605/348995/2733/79833/55520/51367/1964/9883/100101267/11269/8894/51095/55308/1294
1284/3915/4792/5970/1164/836/5925/581/9134/5743/317/1871/7157/7185/1026/3675/1643/391
0/5564/91860/5106/57223
483/3914/8324/8515/8772/80326/9519/5649/148022/5159/1292/23352/1311/355/7148/1284/3134/
48/1284/375790/3915/3694/3690/3696/960/3678/3675/3676/7058/7450/961/3918

30/8678/1351/4714/4700/4712/5481/1329/4724/4711/842/4704/55081/4698/847/7384/6392/5717/
/57142/4700/4712/5481/1329/4724/4711/842/4704/4698/7384/2597/6392/5717/7416/5705/4728/4
4711/842/4704/4698/847/7384/6392/5717/4747/7416/5705/10013/4728/11345/5683/4695/4723/1
700/4712/1329/4724/5468/4711/842/4704/55081/4698/23186/7384/6392/5717/7416/5705/4728/5
2/1329/4724/4711/842/4704/4698/4128/7384/6392/5717/7416/5705/4728/5683/4695/4723/810/12
2/1329/4724/4711/842/4704/4698/7384/10963/6392/5717/7416/5705/4728/5683/4695/4723/1263
9/4724/5468/4711/4704/65260/27330/25915/4698/6009/7384/788/6392/4728/6595/107/4695/472

3/4967/50/847/51179/1892/2098/5230/2597/1962/729020/6392/2203/2023/51/5160/83440/4190/5
321/5684/5688/4976/2905/2475/9861/10524/5582/55062/2959/5715/5289/5700/5693/5714/5682/
243/1719/221264/124454/51109/2729/169355/8564/55163/80347/64802/8836/10229/23475/7372/
34/6392/6720/4728/4695/4723/126328/79602/4694/5313/5564/51094/9167/4713/4716/55967/560
716/55967/5602/4697

33

287
54902

8/54657

/8528/11264/5052/11001/5826/5828/8800/10005/8310/10478/4598/92960/5194/51268/54363/106

/4698/7384/6392/526/4728/528/4695/4723/126328/4694/9167/4713/4716/55967/7381/4697/1097
658/54657/50484
445329/221
/2806

/920/945/3684/915/911/3566/912/3112/3675/914/3676/1436/3575
.3/5579/1906/3725/3383/7412/3576/1958
53/4615/718/3725/3576

280/3725/7128/3934/3576/6372/2920/2919
.7/3108/3109/920/7535/915/3718/3566/3932/27040/3112/4773/864/2353/6775/3725
/3676/2353/4615/718/3725/4689/65108
/6374/6364/3112/2353/3725/3383/10312/3576/6372/2920/2919
2/3576/330/2920/2919
26/6348/6349/414062/9641/7097/51284/51311/6352/2353/23643/4615/3665/3725/3576/3455
6253/834/2207/602/10379/5971/1263/4773/22808/1959/30835/6237/3725/29108
./3553/7097/3554/5579/3684/911/912/5272/87/3918/3576/2920/2919
/3075/714/2213/2357/715/717/718/3383
353/3725/27071/5880/8519
/3560/3115/3091/916/4794/919/9466/3662/917/3554/3108/196/3109/920/7535/915/3718/50615/3

9/414062/6375/729230/6363/1234/6357/1235/1236/6347/6358/6352/8807/6374/8797/7133/6364/
/3725
5/7097/3108/3109/8807/50615/3566/3112/6775/3725

0/6372

5/4689/3383/87/9076/5880/7412/83593

792/5970/6772/4599/6776/4478/3552/836/581/3553/940/9134/3560/9641/7097/51284/916/317/3

5/896/3566/3601/1439/1026/9021/6775/3587/894/3575/4609/3455
0109/5880/3576/29108/391
5/3845/399694/3821/3822/5551/3133/3107/3105/22914/919/2207/7305/5579/7409/7535/5777/36
3105/1364/6403/3696/6401/959/3108/3109/923/920/3684/3683/6404/1001/3112/1462/6614/914/3
66/1522/6709/3563/597/839/4001/1521/317/60/4000/1616/7157/8797/1439/7132/7185/2353/372
/6401/6347/602/6352/11035/3976/6374/7133/6364/1906/7132/7185/2353/9021/3725/3383/3726/7
132/3587/23643/4615/3918/330
146/3105/4688/811/2212/653361/3108/60/3678/3109/3684/6890/3112/2213/30835/715/7058/111
3552/9020/10859/9103/3553/8792/2274/2209/695/10288/11025/4688/3727/2212/10379/3554/653
76/2207/2212/60/113/6916/3937/10235/6786/7450/83706/54518
78/7042/4792/5970/6772/6776/332/836/3845/8900/5925/581/9134/890/1960/9641/7097/317/557
4261/3119/8767/3115/2209/7097/3687/26253/317/2207/2212/3108/3109/3684/1263/3112/8877/7
2530/942/7030/958/3560/597/890/2120/2209/904/5966/2119/51274/55589/3684/8013/7157/7185
1/148022/9051/5603/5594/4792/5970/6772/1536/5332/118429/837/51393/114548/3606/3710/641
5/3107/9134/6672/890/8379/1960/3105/4067/1234/5966/991/10379/3718/896/7157/7185/1026/59
7/8772/90865/103/148022/3117/3126/3123/3460/355/5594/4792/5970/6772/4599/29107/3113/114
3405/90427/1789/1545/1945/3690/7431/836/3845/8434/399694/1788/2146/4363/9134/6624/5743
776/3845/6362/399694/5197/2771/6846/6348/6349/414062/6375/729230/2791/6363/4067/1234/6
2/3572/2782/836/3845/3710/5925/581/3133/942/3107/11337/9641/2791/3105/3091/5743/4067/12
.17/3126/3123/355/5603/3134/4792/5970/6772/3113/7431/9020/836/8900/9636/5925/581/3133/6
3570/6173/2266/6224/2243/5603/5594/4792/6142/11224/5335/5970/6772/4599/3572/1536/2244/
.17/3126/3123/196883/4215/3134/5594/7042/4792/5970/6776/3689/3113/9020/3845/8900/5925/5
4/2768/8772/3570/196883/355/5603/3134/5594/4792/5970/6351/2782/5332/2775/3690/2776/836
503/3134/5594/4792/8905/5335/5970/2782/60489/2775/2776/836/3845/3710/684/581/3133/6891/

4548/3606/836/10398/203068/197259/581/5898/10376/84617/6281/3553/29109/8767/7097/302/5
460/355/85480/3595/7042/56477/6373/6359/6351/3572/9518/1524/8809/10563/3606/3552/4050/
58/4233/5159/4254/5154/5598/1849/355/4215/57551/4296/5603/5594/7042/5970/929/2252/1945/
/7855/7477/2887/6520/10670/7479/525/10325/2475/8323/5582/5604/5562/3667/9296/55615/387

/6041/285268/389114/390980/8503/7752/25799/148254/117608/79973/23586/342909/163050/35
7/5594/6778/3915/7042/862/4792/5335/5970/6772/374654/6776/3572/2252/2782/5332/2950/190

970/6772/4599/975/836/3845/5925/581/9641/1365/1364/317/10379/4600/1871/7157/7132/1026/9
17/5594/5335/10627/54910/2051/54961/1945/1808/5998/3845/10398/57689/10507/2242/57556/2
9170/581/10376/84617/3553/7456/1365/4641/1364/5063/834/10094/2212/9266/3554/60/5777/30
9694/5063/3696/3082/60/3678/2316/5579/7409/7791/896/64098/3675/3676/7058/3725/7450/87/8
/2260/2903/55740/1268/1969/5216/3082/60/5579/7409/113/5900/3684/3683/27040/3937/22808/
3/2535/867/406991/3082/60/3678/2316/5579/7409/5777/967/5329/6469/7157/3059/1026/22808/1
2475/5770/5604/5562/3667/5257/5577/6198/53358/6199/6194/572/805/79660/3632

5/114548/3552/8605/197259/581/3553/834/10379/11035/10616/3718/8797/7132/6775/7128/2910
7157/3059/9844/7132/10095/4615/718/3725/87/10109/3576/29108

25/3133/3107/9134/890/3105/2113/90550/1871/896/7157/4773/10758/1026/22808/677/6237/894/
01/26019/8892/4928/1983/22985/1915/8661/10775/56001/5411/1967/138716/8086/7175/22916/8
3/330/4609

/5594/3915/5970/6772/4599/3694/9794/3690/836/3845/8900/8992/9636/5925/581/3133/3107/91

/4747/7416/5705/4728/11345/5683/4695/4723/810/342371/126328/10133/8322/4694/5663/9167/
4311/5683/4695/4723/810/126328/8322/4694/5663/9167/5718/4713/51107/79861/7855/55102/41
26328/10133/6396/4694/9167/5718/4713/10120/79861/55102/5702/4716/348995/5217/55860/273
683/1387/4695/4723/1213/126328/4694/9167/5718/5431/4713/10120/79861/55102/5702/4716/55
26328/4694/9167/5718/4713/79861/7326/4137/5702/4716/91860/7419/55967/118424/25828/5602
28/727/3312/4694/9167/5718/4713/79861/5702/4716/7419/55967/5602/7381/4697/10975/1337/5
23/285521/126328/4694/1376/5564/51780/9167/4713/4716/6597/388753/55967/51287/7381/4697

5213/39/6120/5313/4524/5631/48/5226/229/132158/3030/80201/3419/8310/7167/5095/5723/557
29982/773
219/6697/1723/5313/7389/10327/54576/205/93100/54577/54600/79646/5372/57026/51601/5457
02/7381/4697/10975/1337/5601/5599/8660/6256

54/1384/26063/5824/4358/5192/25824

75/1337/155066/27068/525/479

3566/3932/27040/3112/4773/2353/3725

7132/1436/3587/6366/51554/6368/3576/6372/2920/2919

310/10379/4600/917/6504/915/3718/896/7157/2213/2353/30835/4939/4615/3665/3725/7128/894

83/3932/27040/8797/3937/4773/962/3383/10870/5880/3455
3676/6402/3383/9076/7412/57502
5/330/7846
7128/7412/330/9586/6372/2920/2919

51/718/4689/10312/7846
3361/7305/5971/8600/9846/3932/3937/7132/4773/55423/2213/2353/9021/1436/3725/4689/3726/

79/3718/1871/7157/4773/1026/1959/2353/6775/4615/1643/3665/3725/3576/4609/9586
7132/2213/30835/3587/11151/4615/718/10312
5/1026/1436/1643/5371/894/64332/3576/1848/330/4609
127/3553/115362/8767/11337/9641/22861/84674/834/6347/10379/6352/11035/10392/3428/1061
922/9734/1959/3665/718/3725/87/894/9586
4548/3606/3552/836/581/3553/4261/3119/3115/9641/51284/834/11100/317/6347/5611/10379/46
3/3925/4082/27086/960/406991/3678/5579/1871/6659/7157/1026/994/894/4609
6357/1235/3055/1236/6347/2790/6358/2870/54331/6352/653361/5579/7409/113/1794/3718/6374/
234/3055/2790/9976/10379/54331/1871/7157/6932/7132/4773/1026/2353/3665/718/3725/3383/3
6891/930/958/3107/3119/9134/3115/890/695/9641/7097/3105/567/4067/916/4794/953/317/960/9
/6165/114548/6230/6154/6205/1675/6144/9636/716/6202/3553/51065/6223/629/6203/9641/7097/
581/200186/3133/958/3107/3119/9134/3560/9232/3115/890/8379/4488/3105/567/916/991/2113/
6/3845/3710/11214/5925/581/3553/3133/6891/2771/3107/6348/6349/414062/2791/3105/567/574
/2771/3107/7097/2791/3105/567/1234/5063/916/2790/919/811/54331/917/5579/920/9582/27350/

5063/834/10094/388/9266/5216/11035/10392/60/2316/113146/399/3071/8797/6932/9844/7132/2
6362/133396/7293/944/1438/3594/3553/5197/8792/958/8771/3560/3563/6846/6348/6349/414062
/3552/9020/836/3845/8605/3315/1850/3553/6789/5778/1326/2260/2323/11184/3925/1969/1847/3
7/6198/90423/6199

69/7188/3448/55762/80818/10838/578/3654/80264/84503/162655/84449/5610/841/26152/16307
2/1164/332/2776/836/3845/8900/5979/11040/1438/3594/9170/367/5925/581/5898/6789/2771/53

9021/4939/3665/9076/4609/3455
2771/22885/55740/5063/1969/6091/8829/6469/4773/23380/6237/5880
71/8797/4642/3059/7132/2353/10095/64005/4615/4542/3725/9076/10109/3576/29108/7846
394/3918/5880/330
10235/2357/1436/6237/5880/83593/54518
1839/6237/4609

08/330/3455

/3576/83593/4609
84321/25929/8480/5976/728343/728689/55998/10940/9775/51068/10557/10556/9984/23225/5712

34/3659/890/9641/3105/5743/7482/3696/2535/10379/4600/26508/3678/896/7157/6932/7132/102

'5718/4713/10120/79861/7326/7855/55102/4137/5702/4716/55860/7477/91860/7419/140775/559
.37/5702/4716/7477/91860/7419/55967/3028/5602/25825/7381/4697/10975/5664/1337/5601/570
33/140775/55967/7381/4697/10975/9883/100101267/1337/5706/375189/9821/5684/5688/129401/
5860/7419/55967/5602/5435/7381/4697/10975/1337/5601/5706/293/5599/9821/5684/5688/51164
2/7381/4697/10975/1337/10131/7345/5601/5706/293
580/5601/5706/293/1457/5599
/10975/1337/137682/84987/9658

53

8/54575/54659/54579/1503/2730/554235/112724/2990/23498/54658/54657/248/3242/6472/5449

[illegible]/3455

[illegible]2355/3455

[illegible]/90550/114769/9447/4939/4615/3665/3725/2634/7128/3576/29108/330/2920/2919/3455

[illegible]00/6352/3108/60/5579/3109/896/8797/7177/3112/7132/64499/9021/4939/4615/3665/5371/56649[illegible]

[illegible]/7454/3702/9844/6364/10235/2268/6366/6368/5880/3576/58191/6372/2920/2919
[illegible]576/7538/4609/2920/2919/3455
[illegible]19/811/10379/917/3108/3109/5971/3683/915/3718/1871/896/7157/6890/3112/1026/864/4939/46[illegible]
[illegible]/51284/834/6403/6169/6347/6157/51311/2212/10379/6181/4600/140801/728/5579/713/25873/88[illegible]
[illegible]311/917/3554/3108/3109/5971/920/113/3683/915/3718/1871/896/8829/3932/706/7157/3601/311[illegible]
[illegible]3/1234/6347/2790/811/54331/6352/3554/5579/113/1871/7157/6890/7132/4773/1026/3587/5880/[illegible]
[illegible]/915/8906/6890/7133/7132/4773/2353/4615/3725/25939/5880

353/10095/23643/4615/6237/3725/10109/3576/29108/391/330/4609/7846
2/1441/6375/729230/338376/6363/9180/3574/1234/6357/1235/1236/6347/9466/6358/6352/959/3
3310/3727/3554/11221/1846/9448/3082/2316/5579/7786/5971/374/1616/7157/1844/7132/22808/

1/637/7187/4940/7695/3118/126017/9534/284307/8772/9831/8764/81856/148022/9451/84914/31
66/9134/3560/3563/2260/890/1441/2323/2791/3091/3574/5743/7482/317/2113/405/2790/4313/2

22/55746/9972/5901/1974/79023/11260/1981/55916/81929/23636/8667

26/3675/3676/7058/8638/7450/894/3918/10312/9586/3455

67/3028/118424/5602/7381/4697/10975/5664/1337/10131/7345/5601/5706/293/1457/5599/1020/
6/293/1457/5599/1020/488/9821/7479/8660/5684/5688/2905/2475/8323/4709/9861/8883/348/56
/51164/2905/2475/5868/84134/4709/9861/4928/10452/3181/3084/310/5606/55062/29979/25978/
/2475/4709/9861/55062/5715/4719/4710/5440/5289/5700/2880/5693/6874/27089/5714

0

/3383/3576/29108/3455

15/1643/3665/3725/3383/7128/894/4609/3455
29/2197/6168/200916/7132/714/2353/715/4939/1839/717/4615/718/3725/7450/729/6136/3576/3
2/7132/4773/1026/1959/2353/3725/4316/3383/894/7538/4609/9586/1958
3576/4609/9586

554/23765/3976/920/8600/650/8807/10673/3624/50615/6374/3566/8797/939/7133/3601/6364/14
5922/10235/2353/1436/4615/784/6237/3725/994/5880/1848/4609

17/3126/54811/115509/170960/3123/3460/81931/140612/125893/355/339559/3134/4792/79088/
535/867/54331/3082/26508/5579/113/5900/650/3718/1871/896/3566/6469/7157/6932/3601/1906

[illegible]488/9821/7479/5684/5688/51164/2905/2475/5868/84134/8323/4709/9861/10452/5582/5606/731[illegible]
[illegible]4/55062/5715/4719/1131/4710/5289/5700/3667/5693/27089/5714/50507
[illegible]5715/56001/4719/4710/5289/5700/2880/5693/27089/7175/5714

39/7132/3603/1436/3587/6366/51554/608/6368/3575/3576/58191/9235/6372/2920/2919/3455

5970/6772/147660/55659/3113/90321/3690/836/90338/90827/684/90649/581/972/3553/3133/68[illegible]
[illegible]/1439/7185/1026/3675/10235/2353/6775/1436/1643/3725/5371/894/3918/5880/3575/3576/8359[illegible]

6/5604/55062/25978/5715/4719/1131/4710/5289/5700/2880/5693/27089/5714/55669/50507

[illegible]91/5199/3107/3119/6672/65243/3115/9641/7097/3105/567/163227/317/6347/811/10379/6352/31[illegible]
[illegible]/330/4609/3455

08/3678/3109/10308/1616/94039/7157/6890/3112/7132/9021/4939/4615/3665/718/5371/330/345

5
