# Supplementary material for: The Potential Diagnostic Value of Immune-Related Genes in Interstitial Fibrosis and Tubular Atrophy after Kidney Transplantation
Source: J Immunol Res. 2022 Jun 17;2022:7212852. doi: 10.1155/2022/7212852 (PMC9232312; doi:10.1155/2022/7212852)
Supplement: Supplementary Materials — Supplementary Figure 1: GSEA enrichment analysis of the IF/TA group. Supplementary Figure 2: correlation analysis between ANGPTL3 and differentially expressed immune infiltrating cells. Supplementary Figure 3: correlation analysis between APOH and differentially expressed immune infiltrating cells. Supplementary Figure 4: correlation analysis between EGF and differentially expressed immune infiltrating cells. Supplementary Figure 5: correlation analysis between FCGR2B and differentially expressed immune infiltrating cells. Supplementary Figure 6: correlation analysis between HLA-DQA2 and differentially expressed immune infiltrating cells. Supplementary Figure 7: correlation analysis between LTF and differentially expressed immune infiltrating cells. Supplementary Figure 8: IPA analysis shows the interaction network of diagnostic genes: EGF and LTF (8A), ANGPTL3 (8B), FCGR2B and APOH (8C), and HLA-DQA2 (8D). Merged the above four independent networks to comprehensively analyze the interaction of diagnostic genes (8E). Supplementary Table 1: immune-related genes. Supplementary Table 2: KEGG pathway in normal group. Supplementary Table 3: pathway of ANGPTL3 gene. Supplementary Table 4: pathway of APOH gene. Supplementary Table 5: pathway of EGF gene. Supplementary Table 6: ingenuity canonical pathways. Supplementary Table 7: category. [file 7212852.f1.zip › 7212852.f1/supplementary table9.pdf]

ID	Descriptior	setSize	enrichmen	NES	pvalue	p.adjust	qvalues	rank
hsa05200	Pathways i	492	0.288704	1.63771	0.001416	0.007052	0.00362	4319
hsa05168	Herpes sir	427	0.407695	2.285798	0.001435	0.007052	0.00362	5578
hsa04010	MAPK sign	274	0.295127	1.57405	0.001546	0.007052	0.00362	3189
hsa04060	Cytokine-c	249	0.459573	2.434882	0.00155	0.007052	0.00362	3361
hsa05132	Salmonella	244	0.416601	2.193588	0.001577	0.007052	0.00362	4389
hsa04510	Focal adhe	196	0.348798	1.786559	0.0016	0.007052	0.00362	3725
hsa05166	Human T-(	211	0.491889	2.546843	0.001603	0.007052	0.00362	4283
hsa05205	Proteoglyc	199	0.336046	1.725415	0.001605	0.007052	0.00362	3551
hsa05131	Shigellosis	209	0.354854	1.835231	0.001608	0.007052	0.00362	4251
hsa05171	Coronaviru	195	0.536612	2.741961	0.001608	0.007052	0.00362	3407
hsa05163	Human cyt	210	0.379942	1.964246	0.00161	0.007052	0.00362	4283
hsa05170	Human im	191	0.373705	1.909919	0.001616	0.007052	0.00362	3171
hsa05169	Epstein-Ba	194	0.513406	2.619817	0.001626	0.007052	0.00362	4210
hsa05164	Influenza A	162	0.488962	2.42679	0.001629	0.007052	0.00362	2410
hsa04810	Regulation	204	0.302854	1.55813	0.001634	0.007052	0.00362	2304
hsa05130	Pathogenic	184	0.399709	2.019545	0.001637	0.007052	0.00362	4495
hsa04621	NOD-like r	161	0.498645	2.470922	0.001639	0.007052	0.00362	3084
hsa05203	Viral carcir	161	0.408156	2.022524	0.001639	0.007052	0.00362	4288
hsa03013	RNA transp	152	0.357822	1.767416	0.001642	0.007052	0.00362	5638
hsa04514	Cell adhesi	136	0.519878	2.531358	0.001645	0.007052	0.00362	1835
hsa05161	Hepatitis B	157	0.405473	2.00926	0.001645	0.007052	0.00362	3112
hsa04630	JAK-STAT :	139	0.397912	1.940622	0.00165	0.007052	0.00362	3551
hsa05135	Yersinia inf	130	0.433014	2.09059	0.001653	0.007052	0.00362	4238
hsa05152	Tuberculos	171	0.513311	2.559124	0.001653	0.007052	0.00362	1840
hsa04062	Chemokine	177	0.472258	2.367425	0.001656	0.007052	0.00362	3147
hsa04650	Natural kill	119	0.522944	2.475983	0.001658	0.007052	0.00362	2746
hsa05206	MicroRNAs	163	0.370581	1.834216	0.001658	0.007052	0.00362	3153
hsa04110	Cell cycle	117	0.400527	1.885877	0.001664	0.007052	0.00362	5000
hsa04145	Phagosom	144	0.569409	2.783252	0.001664	0.007052	0.00362	2458
hsa04210	Apoptosis	129	0.411661	1.979463	0.001664	0.007052	0.00362	3582
hsa04218	Cellular ser	148	0.350543	1.721002	0.001667	0.007052	0.00362	3153
hsa05167	Kaposi sarc	175	0.43195	2.158284	0.001669	0.007052	0.00362	4271
hsa03040	Spliceosom	128	0.349741	1.679384	0.001672	0.007052	0.00362	6438
hsa04217	Necroptos	124	0.399959	1.910349	0.001672	0.007052	0.00362	2974
hsa04380	Osteoclast	122	0.561521	2.670978	0.001672	0.007052	0.00362	3582
hsa05162	Measles	132	0.490758	2.373564	0.001672	0.007052	0.00362	3084
hsa04610	Compleme	77	0.540288	2.374447	0.001675	0.007052	0.00362	1374
hsa04612	Antigen pr	77	0.639973	2.812539	0.001675	0.007052	0.00362	2532
hsa05202	Transcripti	158	0.423478	2.091156	0.001675	0.007052	0.00362	2771
hsa05133	Pertussis	71	0.636801	2.751419	0.001684	0.007052	0.00362	1476
hsa04668	TNF signal	108	0.519188	2.412559	0.001695	0.007052	0.00362	3171
hsa05140	Leishmania	74	0.705174	3.066181	0.001695	0.007052	0.00362	2539
hsa05145	Toxoplasm	108	0.516344	2.399344	0.001695	0.007052	0.00362	3331
hsa04657	IL-17 signa	86	0.43611	1.947457	0.001698	0.007052	0.00362	3084
hsa04658	Th1 and Th	86	0.57906	2.585802	0.001698	0.007052	0.00362	2146
hsa05134	Legionellos	57	0.572679	2.371016	0.001698	0.007052	0.00362	3215
hsa04061	Viral prote	87	0.630103	2.81618	0.001701	0.007052	0.00362	2042
hsa05235	PD-L1 exp	87	0.512585	2.290944	0.001701	0.007052	0.00362	3084
hsa05414	Dilated car	87	0.392346	1.753551	0.001701	0.007052	0.00362	4677
hsa04670	Leukocyte	105	0.42182	1.948764	0.001704	0.007052	0.00362	1507
hsa05416	Viral myoc	58	0.679317	2.820205	0.001704	0.007052	0.00362	3484
hsa04611	Platelet act	114	0.414755	1.932947	0.001706	0.007052	0.00362	4166
hsa04662	B cell recep	79	0.486606	2.131165	0.001706	0.007052	0.00362	3624
hsa03010	Ribosome	110	0.364183	1.686646	0.001709	0.007052	0.00362	4512
hsa04512	ECM-recep	82	0.401109	1.765811	0.001709	0.007052	0.00362	3158
hsa05410	Hypertroph	82	0.410956	1.809162	0.001709	0.007052	0.00362	3741
hsa05150	Staphylocc	73	0.708493	3.070399	0.001715	0.007052	0.00362	1835

hsa04640	Hematopo	91	0.61745	2.783194	0.001724	0.007052	0.00362	2177
hsa04666	Fc gamma	95	0.414546	1.880011	0.001724	0.007052	0.00362	2119
hsa05323	Rheumatoi	90	0.580316	2.598251	0.00173	0.007052	0.00362	2136
hsa04659	Th17 cell d	96	0.577157	2.622631	0.001733	0.007052	0.00362	3084
hsa04660	T cell recep	96	0.442938	2.012733	0.001733	0.007052	0.00362	4277
hsa04064	NF-kappa	98	0.542017	2.463989	0.001742	0.007052	0.00362	2442
hsa04620	Toll-like re	98	0.521666	2.371475	0.001742	0.007052	0.00362	3215
hsa04625	C-type lect	98	0.461367	2.097358	0.001742	0.007052	0.00362	4166
hsa04933	AGE-RAGE	99	0.434776	1.98581	0.001742	0.007052	0.00362	2760
hsa05146	Amoebiasis	98	0.501684	2.280636	0.001742	0.007052	0.00362	2760
hsa05142	Chagas dis	97	0.500707	2.273293	0.001745	0.007052	0.00362	3215
hsa05322	Systemic lu	48	0.77094	3.075403	0.001745	0.007052	0.00362	2136
hsa05144	Malaria	47	0.672157	2.67504	0.001748	0.007052	0.00362	1716
hsa05321	Inflammato	55	0.663962	2.712362	0.001754	0.007052	0.00362	2436
hsa04672	Intestinal ir	42	0.649702	2.503836	0.001783	0.007052	0.00362	1835
hsa04940	Type I diab	41	0.697649	2.671138	0.001783	0.007052	0.00362	3215
hsa05332	Graft-versu	41	0.7668	2.935898	0.001783	0.007052	0.00362	2136
hsa05320	Autoimmu	44	0.720346	2.806441	0.001792	0.007052	0.00362	1835
hsa05340	Primary im	34	0.61656	2.25984	0.001795	0.007052	0.00362	2890
hsa05330	Allograft re	33	0.812025	2.953563	0.001812	0.007052	0.00362	2136
hsa05310	Asthma	23	0.817058	2.76112	0.001845	0.007052	0.00362	1835
hsa00532	Glycosamir	20	0.626082	2.047928	0.001887	0.007052	0.00362	1242
hsa00360	Phenylalan	17	-0.67266	-2.13332	0.00207	0.007052	0.00362	4543
hsa00340	Histidine m	21	-0.65056	-2.23464	0.002083	0.007052	0.00362	2833
hsa01210	2-Oxocarb	18	-0.64464	-2.0862	0.002092	0.007052	0.00362	2111
hsa00220	Arginine b	20	-0.67829	-2.28376	0.002119	0.007052	0.00362	3535
hsa04744	Phototrans	20	-0.60806	-2.04732	0.002119	0.007052	0.00362	6585
hsa04977	Vitamin dig	20	-0.62327	-2.0985	0.002119	0.007052	0.00362	2640
hsa00040	Pentose ar	26	-0.58114	-2.07972	0.002165	0.007052	0.00362	4801
hsa00053	Ascorbate	23	-0.66496	-2.29322	0.002174	0.007052	0.00362	4801
hsa00790	Folate bios	23	-0.61661	-2.12648	0.002174	0.007052	0.00362	3681
hsa04966	Collecting	27	-0.63932	-2.29001	0.002198	0.007052	0.00362	1571
hsa00640	Propanoate	30	-0.60366	-2.20927	0.002212	0.007052	0.00362	3624
hsa00260	Glycine, se	35	-0.57222	-2.23196	0.002222	0.007052	0.00362	4855
hsa00350	Tyrosine m	33	-0.5719	-2.20027	0.002222	0.007052	0.00362	4543
hsa00410	beta-Alani	28	-0.62022	-2.23608	0.002222	0.007052	0.00362	4220
hsa00650	Butanoate	24	-0.72548	-2.53474	0.002237	0.007052	0.00362	4033
hsa00020	Citrate cyc	29	-0.62251	-2.25928	0.002247	0.007052	0.00362	3720
hsa00250	Alanine, as	34	-0.52294	-2.02405	0.002247	0.007052	0.00362	2912
hsa00630	Glyoxylate	29	-0.65739	-2.38589	0.002247	0.007052	0.00362	2522
hsa00071	Fatty acid (	42	-0.50826	-2.0708	0.002268	0.007052	0.00362	4033
hsa00380	Tryptophar	40	-0.48311	-1.9421	0.002273	0.007052	0.00362	4622
hsa00270	Cysteine ar	46	-0.43518	-1.82303	0.002278	0.007052	0.00362	4322
hsa00280	Valine, leu	46	-0.65452	-2.74186	0.002278	0.007052	0.00362	4033
hsa00620	Pyruvate m	36	-0.53948	-2.10078	0.002309	0.007052	0.00362	3584
hsa00330	Arginine ar	48	-0.5437	-2.30999	0.002331	0.007052	0.00362	3867
hsa00190	Oxidative p	98	-0.59272	-2.91465	0.002336	0.007052	0.00362	4665
hsa00830	Retinol me	50	-0.4229	-1.79807	0.002358	0.007052	0.00362	4801
hsa00980	Metabolisr	60	-0.44619	-1.95351	0.002364	0.007052	0.00362	4801
hsa01230	Biosynthes	65	-0.46937	-2.09331	0.00237	0.007052	0.00362	2330
hsa01200	Carbon me	110	-0.52915	-2.67333	0.002398	0.007052	0.00362	3965
hsa04721	Synaptic ve	68	-0.41132	-1.84392	0.002415	0.007052	0.00362	3551
hsa00010	Glycolysis /	57	-0.42894	-1.86114	0.002421	0.007052	0.00362	3781
hsa00982	Drug meta	57	-0.46829	-2.03187	0.002421	0.007052	0.00362	4801
hsa04976	Bile secreti	71	-0.42032	-1.90171	0.002451	0.007052	0.00362	5533
hsa04146	Peroxisome	77	-0.49028	-2.24572	0.002469	0.007052	0.00362	3625
hsa01240	Biosynthes	142	-0.36795	-1.91669	0.002481	0.007052	0.00362	5150
hsa04932	Non-alcoh	137	-0.31714	-1.63619	0.002538	0.007151	0.003671	4665

hsa04714	Thermoge	189	-0.37539	-2.03162	0.002591	0.007236	0.003714	5726
hsa05016	Huntingtor	254	-0.27492	-1.54784	0.002786	0.007714	0.00396	4976
hsa04151	PI3K-Akt s	321	0.274689	1.497101	0.003003	0.008246	0.004233	4283
hsa04015	Rap1 signa	194	0.303889	1.550691	0.003252	0.008838	0.004537	3096
hsa04921	Oxytocin s	138	0.337701	1.648578	0.003273	0.008838	0.004537	4121
hsa05160	Hepatitis C	144	0.332322	1.624378	0.003328	0.008911	0.004574	3841
hsa05100	Bacterial in	67	0.409178	1.738011	0.003396	0.00896	0.004599	4238
hsa04115	p53 signali	72	0.379519	1.644094	0.003401	0.00896	0.004599	4170
hsa04540	Gap junctio	81	0.383811	1.684758	0.003436	0.008979	0.004609	3028
hsa03050	Proteasom	43	0.46989	1.808553	0.003636	0.009425	0.004838	5680
hsa00601	Glycosphir	27	-0.49502	-1.77313	0.004396	0.011303	0.005802	3087
hsa04960	Aldosteror	35	-0.44244	-1.72576	0.004444	0.011339	0.00582	3042
hsa01212	Fatty acid r	55	-0.41036	-1.77893	0.00463	0.011666	0.005989	4033
hsa04742	Taste trans	47	-0.40025	-1.68098	0.004651	0.011666	0.005989	5833
hsa00140	Steroid hor	50	-0.40592	-1.72588	0.004717	0.011666	0.005989	4801
hsa04978	Mineral ab	50	-0.41099	-1.74742	0.004717	0.011666	0.005989	4800
hsa05204	Chemical c	69	-0.38993	-1.75915	0.004808	0.011801	0.006058	4801
hsa04120	Ubiquitin r	132	0.319629	1.545893	0.005017	0.01213	0.006227	4244
hsa05418	Fluid shear	132	0.321153	1.553264	0.005017	0.01213	0.006227	4210
hsa04623	Cytosolic D	56	0.406643	1.677497	0.005119	0.012287	0.006307	4106
hsa04964	Proximal tu	20	-0.5828	-1.96225	0.006356	0.015142	0.007773	3818
hsa04926	Relaxin sig	119	0.328762	1.556588	0.006633	0.015688	0.008053	4283
hsa05222	Small cell l	90	0.354492	1.58717	0.00692	0.016248	0.00834	3158
hsa05143	African tryp	33	0.472921	1.720147	0.007246	0.016891	0.00867	2248
hsa04723	Retrograde	121	-0.31092	-1.5841	0.007444	0.017228	0.008843	5923
hsa05110	Vibrio chol	49	-0.36595	-1.55202	0.009434	0.021678	0.011128	1218
hsa03018	RNA degra	69	0.365464	1.566384	0.010239	0.023362	0.011992	5957
hsa03030	DNA replic	36	0.453267	1.691589	0.010545	0.023892	0.012264	3666
hsa00030	Pentose ph	28	-0.45263	-1.63185	0.011111	0.024823	0.012742	2873
hsa04975	Fat digesti	35	-0.41785	-1.62985	0.011111	0.024823	0.012742	3577
hsa00970	Aminoacyl	24	-0.49729	-1.73748	0.011186	0.024823	0.012742	5582
hsa04350	TGF-beta s	87	0.34979	1.563349	0.011905	0.026239	0.013469	3909
hsa04150	mTOR sigr	146	-0.27424	-1.43059	0.012563	0.027502	0.014118	4030
hsa05012	Parkinson	203	-0.24252	-1.32796	0.015625	0.033977	0.017441	5179
hsa00561	Glycerolipi	55	-0.35012	-1.51779	0.018519	0.039956	0.02051	5004
hsa05412	Arrhythmo	68	0.349985	1.495028	0.018707	0.039956	0.02051	3858
hsa00480	Glutathion	53	-0.3567	-1.53302	0.018779	0.039956	0.02051	2907
hsa03008	Ribosome	73	0.350451	1.518753	0.018868	0.039956	0.02051	5175
hsa00860	Porphyrin a	34	-0.40278	-1.55899	0.020225	0.042551	0.021842	4839
hsa00983	Drug meta	69	-0.32435	-1.46329	0.021635	0.045223	0.023214	4801
hsa00062	Fatty acid e	25	-0.44201	-1.55878	0.022026	0.045747	0.023483	5528

leading_edcore_enrichment
tags=34%, 113/54331/1439/5880/6775/83593/4609/1436/330/3575/1902/1438/2252/637/3725/78
tags=49%, 330/718/10308/4939/3115/7097/3113/637/3109/6347/3108/6352/3460/836/6672/2999
tags=27%, 929/5880/11221/4609/1436/3554/3310/2252/3725/4208/836/2353/9448/5579/5156/18
tags=41%, 3587/10673/1439/7133/1436/3554/3575/1234/1438/3603/90865/729230/7852/6362/35
tags=44%, 23643/10109/834/7846/302/197259/929/5788/29108/4609/84617/330/9844/3071/1037
tags=35%, 5880/7058/64098/330/857/3676/3725/3908/1293/894/858/7450/2335/7409/5579/1062
tags=49%, 113/3689/4773/4609/3554/1959/8829/3115/3113/3109/3725/3108/958/894/3601/3683
tags=35%, 960/5329/4060/3059/4609/857/7097/836/858/7099/2335/7409/5579/4318/3082/10855
tags=38%, 960/10109/834/3059/929/29108/9844/3554/718/837/8767/3725/6352/10392/7099/732
tags=48%, 2212/1536/716/834/713/714/4600/715/718/51311/8829/51284/4939/7097/3725/6223/
tags=40%, 113/3587/54331/5880/4773/4609/3554/1234/637/7852/10672/6347/6352/836/2791/63
tags=31%, 25939/54331/5880/4773/7133/1234/7097/637/3725/7852/836/60489/919/7099/2791/2
tags=52%, 960/7431/4609/7128/4939/3115/7097/3113/637/3109/3725/953/4067/3108/958/894/3
tags=40%, 834/29108/4600/51284/4939/90865/3115/3113/637/3109/6347/3108/6352/3460/836/7
tags=23%, 3684/10109/3689/5880/9459/1902/3071/3676/2252/7852/7114/10672/3683/2335/7409
tags=45%, 2212/10109/834/7846/3059/29108/84617/3554/1902/3071/9871/10376/4542/837/3725
tags=39%, 3428/1536/834/2634/114769/29108/7128/330/4939/837/8767/3725/6347/9447/6352/1
tags=43%, 9734/718/1959/1234/3725/4067/894/836/1960/6672/5366/7188/3133/87/5594/5925/8
tags=49%, 8761/9631/3646/26986/9669/79902/6613/1973/8672/8487/10762/53371/1965/387082/
tags=38%, 3684/942/3689/5788/1462/6614/6402/5175/4267/3115/914/3113/3676/3109/57502/31
tags=32%, 6775/4773/4609/1959/7097/637/3725/353376/836/7099/1960/2353/5579/3576/4318/1
tags=38%, 3587/1439/6775/4609/3575/1438/5771/3574/3460/894/3601/5156/3560/3563/3455/36
tags=43%, 2212/10109/834/3937/5880/4773/29108/9844/3676/3725/6347/7099/2353/3932/2335/
tags=35%, 2213/3684/3587/9103/1520/2212/2207/4360/3689/929/718/11151/3115/7097/3113/70
tags=40%, 113/54331/1794/3055/5880/2268/9844/1234/729230/7852/6362/4067/6347/3702/6352
tags=39%, 7305/2207/3689/3937/5880/4773/10870/962/637/3460/3683/836/919/3932/7409/5579
tags=33%, 960/7431/4609/4082/9839/23414/894/836/5579/2146/5728/5156/406996/4318/406991
tags=50%, 4609/4175/894/10926/9232/11200/5925/890/9134/8317/6502/7533/996/7534/7042/99
tags=44%, 2213/3684/4689/4688/9103/1520/2212/1536/4360/7846/3689/929/7058/84617/715/71
tags=40%, 1520/1439/7846/330/597/10376/637/3725/836/1513/2353/5366/3002/3563/84790/945
tags=29%, 83593/4773/4609/894/5728/3576/3133/11200/5594/6237/5925/890/5500/9134/22808/
tags=43%, 9976/54331/942/3055/4773/4609/718/1234/637/3725/4067/836/2791/2353/3383/2920
tags=55%, 3310/11017/6632/10189/1655/3178/10946/10285/23350/55119/4686/51690/10915/494
tags=29%, 1536/834/197259/6775/29108/7128/330/90865/637/5836/3460/353376/7099/1540/718
tags=48%, 2213/4689/4688/7305/9103/2212/3937/4773/1436/10859/3554/2274/11025/3725/1028
tags=40%, 2213/4600/7128/3310/51284/4939/7097/637/3725/894/836/7099/2353/30835/917/915
tags=34%, 3684/3075/3078/5329/716/11326/3080/3689/713/714/715/718/7056/1604/7450/1675/
tags=48%, 1520/3310/3115/3113/3109/3108/567/6890/925/3126/972/3123/3821/3822/3119/3133
tags=32%, 3684/942/929/2313/4609/1436/330/597/5090/2209/958/894/3087/4208/862/3576/300
tags=42%, 3684/23643/716/834/3689/713/929/714/29108/715/718/3394/3725/353376/836/10392
tags=45%, 197259/7133/7128/330/3725/6347/6352/836/2353/3383/2920/64127/2919/7188/6372/
tags=65%, 3684/4689/4688/9103/2212/1536/3689/718/3115/7097/3113/3676/3109/3725/3108/22
tags=46%, 3587/23643/330/3310/1234/3115/7097/3113/3109/3908/3108/958/3460/836/7099/312
tags=41%, 7128/3725/6347/6280/836/2353/2920/2919/7188/6372/3576/4318/5594/6356/3627/62
tags=48%, 6775/4773/3115/3113/3109/3725/3108/3460/919/2353/3932/917/915/3126/3123/864/
tags=47%, 3684/834/3689/929/29108/3310/718/7097/836/7099/2920/2919/3576/317/4615/3606/
tags=47%, 3587/7133/1436/1234/729230/7852/6362/6347/6352/6366/6357/6348/6349/414062/29
tags=44%, 4773/7097/3725/3460/353376/919/7099/2353/3932/917/915/5728/55509/940/920/559
tags=49%, 113/3676/3908/3479/7168/3678/6444/7171/7042/3685/7043/3694/108/7273/6445/782
tags=28%, 3684/4689/4688/1536/3689/5880/83593/5175/4267/3676/7852/3702/3683/7409/5579/
tags=74%, 942/3689/5880/857/3115/3113/637/3109/3908/1604/3108/958/3683/836/3383/3126/3
tags=46%, 113/2212/2207/3937/6916/83706/10672/4067/7450/64805/10627/5908/5332/1281/545
tags=43%, 2213/5880/4773/27071/10859/8519/11025/3725/10288/4067/2353/7409/5579/971/559
tags=38%, 6223/200916/6202/6173/11224/6136/6224/23521/6188/6175/6142/25873/6193/6159/6
tags=33%, 960/7058/3676/3908/1293/7450/2335/3915/1311/1282/948/961/1278/3371/1292/3678
tags=44%, 3676/3908/3479/1906/7168/3678/6444/7171/7042/3685/7043/3694/7273/6445/782/65
tags=49%, 2213/3684/9103/2359/3075/2212/716/3689/713/714/715/718/2357/3115/3113/3109/3

tags=48%, 3684/960/912/945/929/1436/3554/3575/1438/3115/914/3113/3676/911/3109/1604/35
tags=28%, 2213/2212/10109/3055/5880/5788/4082/50807/4067/2209/7409/5579/8877/2215/653
tags=48%, 10673/942/3689/3115/7097/3113/3109/3725/6347/3108/6352/3683/1513/7099/2353/6
tags=56%, 4773/196/3554/3115/3113/3109/3725/3108/3460/919/2353/3932/917/915/3126/3123/
tags=48%, 3937/5788/4773/3725/3702/919/2353/3932/7409/917/925/915/868/940/920/5594/553
tags=41%, 23643/10673/929/7128/330/597/3554/4067/958/6366/353376/6357/7099/3932/8792/5
tags=43%, 23643/942/929/51311/51284/7097/7096/3725/958/6352/353376/1513/7099/2353/634
tags=46%, 2207/834/4773/29108/1959/3725/1960/30835/1540/868/114548/64581/5594/6237/365
tags=34%, 1536/7056/3725/6347/836/2335/5579/3383/3576/6401/5332/1281/1282/1958/5594/43
tags=38%, 3684/912/3689/929/3554/7097/911/3908/836/7099/2335/5579/2920/2919/3576/913/3
tags=44%, 713/714/718/7097/3725/6347/6352/3460/919/7099/2353/6348/6349/414062/917/915/
tags=69%, 2212/942/716/713/714/715/718/3115/3113/3109/3108/2209/958/2215/3126/3123/311
tags=53%, 3689/7058/2532/5175/7097/2995/3820/6347/958/3683/7099/3383/22914/3576/6403/3
tags=62%, 6775/3115/7097/3113/3109/3725/3108/3460/7099/64127/3126/3123/3119/3118/3122/
tags=52%, 10673/942/3115/3113/3676/3109/7852/3108/958/3601/3126/3123/3119/3118/940/608
tags=73%, 942/3115/3113/3109/3108/3126/3002/3123/3119/3133/3118/940/3122/3112/3134/355
tags=68%, 942/3115/3113/3109/3108/3126/3002/3123/3821/3119/3133/3824/3118/940/3122/311
tags=57%, 942/3115/3113/3109/3108/958/3126/3002/3123/3119/3133/3118/940/3122/3112/3134
tags=56%, 100/5788/3575/958/3932/6890/925/915/64421/920/3543/7535/916/4261/6891/5993/2
tags=79%, 942/3115/3113/3109/3108/958/3126/3002/3123/3119/3133/3118/940/3122/3112/3134
tags=78%, 2207/3115/3113/3109/3108/958/3126/3123/3119/3118/3122/3112/6356/2206/2205/31
tags=45%, 56548/51363/55454/64131/29940/22856/337876/50515/55790
tags=88%, 1644/6898/137362/5053/218/222/3242/10249/259307/2806/221/2805/4128/4282/412
tags=76%, 55748/501/218/222/224/223/219/144193/221/217/26/84735/10841/443/4128/4129
tags=78%, 3420/137362/84706/3417/3421/587/48/2806/2805/95/2875/3418/50/162417
tags=65%, 137362/2746/445/84706/435/2806/2805/4843/27165/384/95/2875/162417
tags=75%, 1258/51806/9187/8787/9626/805/2978/91860/6010/5158/3000/2979/2779/6011/810
tags=55%, 8029/2346/335/6948/25974/9963/6573/686/5948/113278/113235
tags=73%, 79799/54600/54659/54579/54576/54578/54658/54657/54577/54575/2990/10327/994
tags=91%, 79799/54600/54659/54579/54576/54578/54658/54657/54577/54575/501/2990/9104/1
tags=61%, 8836/5092/5860/8644/84105/5053/2643/250/4337/2356/248/249/873/6697
tags=48%, 495/9296/127124/529/535/245972/527/1188/50617/6521/525/245973/155066
tags=63%, 8310/79611/51/26275/8801/1962/5095/35/18/5096/32/593/4329/55902/1892/84532/3
tags=66%, 2593/5723/2653/635/29968/501/64902/189/113675/1610/51268/6470/29958/1491/27
tags=73%, 1644/6898/1312/128/5409/137362/218/222/3242/130/81889/2954/3081/1621/259307/
tags=79%, 55748/54498/8310/501/1807/51/218/26275/222/1962/224/35/223/219/18/221/51733/
tags=79%, 6296/3033/64064/5019/123876/348158/56898/54988/7915/79944/1962/35/18/2571/1
tags=59%, 6389/3420/5105/1743/6391/8801/4191/5091/3417/3421/4967/5106/48/55753/3418/88
tags=56%, 2746/64902/2346/189/80150/445/7915/84706/435/18/8659/259307/2806/2571/2805/2
tags=62%, 189/4191/125061/5095/6470/51179/48/5096/2731/55902/81888/84532/9380/112817/
tags=57%, 3033/128/1376/8310/10455/501/33/51/81616/130/1962/224/35/223/10449/219/34/30/
tags=62%, 51166/1644/56267/3033/501/1743/8564/130013/125061/1962/224/223/219/259307/64
tags=52%, 56267/6898/635/29968/137362/10768/64902/191/23743/4191/113675/4143/587/2593
tags=74%, 3033/64064/5019/501/27034/26275/64902/64087/197322/587/1962/5095/224/35/223/
tags=56%, 501/5105/134526/10873/4191/5091/224/223/5106/219/32/217/55902/84532/9380/531
tags=54%, 4953/54498/501/137362/113451/8974/1152/1610/79814/224/58510/223/219/8659/28
tags=63%, 1327/6390/56901/4698/4717/7381/4728/1347/6389/7384/496/4722/533/479/4726/537
tags=60%, 79799/54600/54659/54579/1549/54576/54578/54658/54657/54577/128/54575/9249/5
tags=62%, 79799/2944/54600/54659/54579/4259/1549/54576/2948/54578/54658/54657/54577/1
tags=37%, 445/84706/5091/113675/4143/229/5232/3417/3421/587/6470/435/48/2806/2805/1491
tags=51%, 29968/54363/128/6389/3420/8310/283871/137362/51/1743/10873/2746/9104/26275/
tags=38%, 10814/533/160/23025/6530/161/6505/6809/6538/773/6511/9296/594855/127124/529/
tags=46%, 128/501/92579/5105/218/222/7167/10327/130/2203/229/5232/224/223/5106/219/253
tags=61%, 79799/2944/54600/54659/54579/4259/1549/54576/2326/2948/54578/54658/54657/23
tags=63%, 6548/6554/5243/6344/115/9429/111/79799/6523/114/343/54600/54659/54579/54576/
tags=53%, 255027/8310/10455/5194/10478/51/5191/5825/26061/6647/189/5824/8309/11001/10
tags=51%, 64802/4338/29926/158067/4144/169355/84274/79799/54600/54659/54579/51805/553
tags=43%, 1327/6390/56901/4698/3630/4717/54205/7381/4728/1347/6389/5602/7384/51094/47

tags=53%, 4708/7249/125965/6598/4707/57492/5600/788/84335/115/29078/79133/116228/2895
tags=39%, 3066/126328/2892/7019/56171/293/774/5693/26100/1327/6390/56901/6874/4698/471
tags=32%, 54331/7058/4609/1436/3575/1902/7097/3676/2252/3908/1293/3574/894/7450/7099/2
tags=28%, 3684/113/3689/3937/5880/83593/1436/1902/2357/2252/3683/7409/5579/5156/3082/5
tags=38%, 113/4773/57118/3725/4208/3759/2353/5579/5332/817/5997/952/29904/5594/5500/86
tags=35%, 4609/4600/4939/637/836/3455/9451/5594/5925/3665/3646/9076/317/23586/3627/677
tags=43%, 10109/3059/9844/857/858/2335/10096/391/3678/10095/7414/71/5290/399694/859/36
tags=40%, 637/894/836/5366/5728/1643/11200/92344/3479/9134/27244/317/6241/5054/355/643
tags=32%, 113/7846/84617/1902/10376/2697/5579/3357/5156/5332/84790/5594/203068/80310/
tags=53%, 5699/5698/5687/5696/5721/5720/3458/5685/5682/23198/5689/5706/5707/5700/1021
tags=48%, 28/8703/10331/2528/8704/79369/6484/53947/2524/2525/2529/2527/2651
tags=49%, 4306/3643/5578/5296/5170/5595/5291/476/5582/486/482/6340/6337/481/3291/23327
tags=55%, 51144/51495/2194/23205/60481/126129/3033/79966/54995/1376/8310/33/51/79993/
tags=57%, 112/255022/5137/9033/50834/6335/114/6326/1131/2555/3351/5568/5331/3354/8083
tags=54%, 79799/54600/54659/54579/54576/1584/3292/54578/54658/54657/54577/1312/54575/
tags=62%, 6523/1317/7779/4502/2495/3163/491/4493/261729/540/140803/115019/4501/4495/44
tags=52%, 79799/2944/54600/54659/54579/4259/1549/54576/56267/9/2948/54578/54658/54657
tags=35%, 330/92912/7321/9246/868/1643/22954/55294/4281/6502/9021/55958/996/8453/5146
tags=36%, 4688/5880/3554/5175/857/7056/3725/6347/4208/858/2353/3383/4318/6401/653361/4
tags=43%, 834/29108/90865/9447/6352/6351/84265/3665/23586/3627/3606/11035/3553/4792/4
tags=70%, 760/762/5105/2746/8671/476/358/5106/27165/10991/486/482/1468/481
tags=36%, 113/54331/3725/2791/2353/4318/5332/1281/1282/2790/5594/4313/1278/1906/9586/7
tags=31%, 4609/330/3908/836/2335/7188/5728/1643/3915/1282/5925/9134/317/6502/7185/4792
tags=39%, 5579/3383/8542/6401/5332/3620/4615/3606/355/3553/7412/3458/2776
tags=54%, 4716/4719/112/4708/2570/4707/5600/2563/10681/115/111/126328/3763/2892/774/11
tags=22%, 9296/127124/529/535/245972/527/50617/525/3784/245973/155066
tags=54%, 8761/5394/7832/26986/4848/84186/246175/9125/55802/132430/694/51690/2026/113
tags=44%, 4175/5557/29935/5427/5422/10535/6117/6118/5982/5984/79621/5111/54107/4174/41
tags=29%, 9104/2203/229/51071/414328/5211/132158/64080
tags=43%, 8694/10554/80168/10555/2169/2168/1056/84647/335/10999/4547/50487/2806/5407/
tags=54%, 79587/118672/25973/51091/10667/5188/57505/80222/2193/124454/57176/23395/549
tags=39%, 4609/10468/3624/2200/5594/1634/2331/4681/7042/4086/7043/26585/5308/7048/345
tags=32%, 9681/8408/3480/57600/64900/58528/7480/10641/8140/3643/8131/7855/5578/6195/1
tags=39%, 10105/5708/89953/111/6531/126328/293/5693/1327/6390/56901/805/4698/4717/556
tags=45%, 253558/196051/3990/2710/8525/129642/64900/501/8694/10554/80168/10555/10327/
tags=40%, 2697/3676/3908/3678/6444/3685/3694/6445/782/6547/71/88/3696/6442/784/4000/36
tags=36%, 2938/2936/9027/2678/2880/3417/4258/373156/2947/51471/79094/2940/79017/2686/
tags=49%, 26354/83732/10813/29889/23560/55127/134430/84128/51602/55813/29107/10199/23
tags=59%, 645/79799/54600/54659/54579/3163/54576/7390/54578/54658/54657/54577/54575/2
tags=46%, 79799/2944/978/54600/54659/54579/4259/1549/54576/9/2948/54578/54658/54657/54
tags=56%, 51144/51495/60481/3033/641371/10965/11332/79993/51102/9524/10449/122970/937

52/3908/3574/10672/3460/894/3601/836/2791/2353/5366/2335/5579/862/10161/7188/5728/3576
2/567/6890/3126/7188/972/3123/80818/3119/3133/80264/3118/3122/3112/3455/9451/5500/3665
44/3082/5908/1847/1846/285/5594/10235/3479/6237/8605/374/6789/5321/80310/22808/5533/92
574/6347/3624/958/6352/3460/6366/3601/6357/6348/6349/414062/8792/2920/2919/51554/6375/6
76/7097/837/8767/3725/836/10392/7099/2353/10627/399/6281/3576/114548/84790/113146/5594
7/5728/5156/3082/5908/3915/1311/1282/87/5594/3479/5500/1278/3371/103910/80310/55742/74
/2353/3932/567/917/3383/915/3126/5728/3123/9232/3119/3560/3133/11200/3118/3122/3112/19
/817/7291/406991/5594/1634/4313/3479/6237/5500/1278/1839/22808/355/3678/8826/117581/96
1/10627/7188/3576/92610/114548/831/5332/87/5594/3099/103910/10096/9252/4615/5216/3606/
6347/7450/7099/2353/5579/1675/200916/6202/3576/2162/728/114548/6403/3455/5594/6173/963
48/6349/414062/567/5579/6890/7188/3576/5156/5332/3133/2790/6351/5594/5925/5533/3134/55
353/567/917/5579/6890/8905/915/7188/3133/2790/920/5594/9582/5533/3134/4615/5530/355/31
683/836/919/567/917/3383/6890/915/3126/7188/3123/864/1643/3119/3133/3118/3122/3112/345
099/5579/3383/3126/3576/3123/114548/3119/3118/3122/3112/3455/5594/3665/317/23586/3627/
9/10627/5156/87/3687/5594/1730/6237/5500/103910/10096/80310/22808/55740/5216/3678/8826
5/10672/4644/836/7099/2353/3576/4642/114548/64005/84790/5594/203068/9076/10096/4615/36
0392/7099/84674/2920/64127/2919/2633/7188/3576/115362/51393/114548/118429/5332/24145/
90/9134/3665/6502/7533/3134/7185/9586/3516/5423/3105/3107/7534/4792/10379/3106/2960/31
/8664/29107/10189/57122/132430/11260/4686/8669/3837/1207/8665/23165/10799/5901/26019/6
08/958/3683/9019/3383/925/3126/3123/6403/6401/3119/3133/6404/3118/940/3384/3122/3112/5
643/5594/5925/890/9134/3665/317/23586/3717/6772/4615/9586/355/51135/7534/4792/7042/704
00/3717/6772/9021/4170/9180/3588/5159/2273/10379/3459/9655/85480/6774/50615/3594/1026/
7409/9815/925/3576/114548/920/5594/10096/4615/391/3606/3678/3553/51135/4792/10095/7535
96/637/8767/3109/3108/2209/3460/836/7099/30835/64127/8877/2215/3126/972/3123/3119/3118
2/6366/6357/2791/6348/6349/414062/7409/5579/2920/2919/6375/6372/3576/6846/6373/5908/636
/3383/2215/22914/3002/3821/3822/3133/3824/3384/3455/5594/5533/5530/355/3105/5551/3107/
1/1545/5594/6935/9134/3371/6624/4853/9252/4170/7168/3678/5159/7042/10253/994/1786/4363/
4/7043/472/1026/891/7272/5933/7532/4087/2810/701/1027/5111/10274/29945/10459/7529/2359
8/11151/10376/3115/7097/3113/3109/3108/2209/7099/30835/6890/2215/3126/3123/3119/1311/6
1/5594/317/7185/4170/840/355/113457/112714/5551/4792/1965/7278/1519/472/1075/4001/71/4
5533/3134/5054/5530/90550/3105/3107/3106/7042/3135/7043/677/10758/7048/2113/472/1026/8
/2919/3576/3133/285/2790/3455/5594/5925/3665/3717/6772/5533/3134/5530/7538/355/3105/31
4115/3183/220988/22938/3192/9410/57461/51645/10569/6633/23451/6100/57819/51691/144983/
38/114548/817/3455/8605/5321/3717/6772/11035/355/3553/10379/3459/6774/3458/2752/142/314
38/2209/54209/3460/1513/2353/3932/8792/1540/55423/2215/653361/3455/5594/6772/5533/9021
/868/3560/940/3455/9451/9134/3665/317/23586/6772/4615/4599/355/3553/6504/51135/4792/10
2/2162/728/710/10544/3687/729/5054/717/2149
/3824/3118/3122/3112/10437/920/3134/5721/3105/3107/3117/3106/3135/5720/3125/3127/4261/
2/2120/4318/2530/1643/3560/64332/3479/890/6935/7185/64919/2078/8013/4211/55589/1848/60
/7099/2353/6372/3576/114548/710/5594/3659/4615/840/3678/3553/717/51135
4318/6401/5594/3600/1906/3659/3627/7185/9252/1326/8809/9021/9586/3726/7424/11035/840/3
09/3460/7099/2353/5579/2215/3126/3123/3119/653361/3118/3122/3112/5594/65108/3717/6772/
6/3123/3119/3915/3118/3122/3112/5594/3717/6772/4615/3588/51135/3117/4792/3459/7042/704
79/3553/23765/4792/6364/7187/3934/10758/6374/6354/5743/3458/4790/9641/6885/53342/4314/
3119/3560/3118/3122/3112/920/5594/3717/6772/5533/4853/5530/3516/3117/4792/3459/7535/91
840/3553/4792/58484/4790/4671/841/7100/1195/10767/3593
920/2919/51554/6375/6372/3576/6846/6373/3560/6363/6351/1236/8807/6356/1524/10563/3627/6
4/29126/10538/3717/6772/5533/4615/5530/4792/3459/7535/6774/916/27040/3091/4794/3458/47
/6547/71/3696/6442/784/7170/4000/4624/7169/3688/487/5350/4625/1674/60/1756/779/786/463
3383/10627/399/4318/5908/653361/87/58494/4313/103910/9076/1003/7412/83700
123/3119/3133/3118/940/3122/3112/3134/6444/3105/5551/3107/3117/8672/3106/3135/3125/312
518/5594/10235/5500/1278/8605/103910/5321/5592/2149/108/4659/2776/71/3710/5742/5290/226
94/5533/5530/10990/11006/4792/4794/3635/4790/5777/5290/25780/118788/4893/5534/3551/108
6229/2197/6189/6135/6187/6130/6203/6154/6166/51187/6138/6191/6123/6124/6230/6143/6194/6
/131873/3685/3694/9899/7060/3918/3696/7148/1284/3688/3910
47/71/3696/53632/6442/784/7170/4000/4624/7169/3688/487/4625/1674/60/5565/1756/779/786/4
108/2209/3683/3383/1675/2215/3126/3123/728/6403/3119/6404/3118/3122/3112/717/2214/3117

74/3108/2209/917/925/915/3126/3123/931/913/3119/3563/3118/952/3122/3112/948/920/910/367
361/5594/8605/5321/10096/65108/2214/10095/9846/27040/7454/3635/8612
348/6349/414062/8792/3383/2920/2919/3126/6372/3576/3123/3119/3118/940/3122/3112/10312/
3119/3560/3118/3122/3112/920/5594/3717/6772/5533/5530/3553/3117/4792/3459/7535/6774/91
3/1326/5530/4690/5062/4792/7535/916/5063/27040/4794/3458/4790/29851/6885/5777/5290/489
579/3383/2920/1540/2919/7188/3576/6363/6351/23586/7185/4615/3553/51135/7412/4792/7535/
3/6349/414062/3576/6373/6351/3455/5594/3665/3627/6772/1326/4615/4283/3553/51135/4792/71
59/22808/6772/5533/5530/3553/26253/4792/10379/4046/5743/5971/4790/9641/3710/53342/5290/
13/1278/1906/3717/6772/5054/7424/3553/7412/7042/7043/6774/7048/4790/4087/5290/7046/102
915/5332/1281/5272/1282/87/1278/910/338382/3553/1511/7042/7043/3458/7414/2776/4790/391
3576/5332/5594/5054/4615/355/3553/51135/4792/3459/7042/7043/916/7048/3458/2776/4790/40
9/3118/940/87/3122/3112/2903/729/717/6737/2214/1511/3117/3125/3127/6632/3458
082/6401/1311/948/4615/3606/3553/7412/7042/7043
3112/8807/6772/8809/3606/3553/3117/3459/7042/7043/6774/3125/3127/50615/3594/3458/3566/
/3122/3112/3600/3117/3125/3127
/3553/3105/5551/3107/3117/3106/3135/3125/3127/3458/5799/1363/941/3593
2/3134/355/3553/3105/5551/3107/3117/3106/3135/3125/3127/3458
/355/3105/5551/3107/3117/3106/3135/3125/3127
9851/3718/84876
/355/3105/5551/3107/3117/3106/3135/3125/3127/3458
17/3125/3127

9

/54490/9365/6652/51181/27294/51084
0327/54490/224/223/219/9365/55586/217

945/38/8802
31/23464/55349/9380/4128/4129/132158/1757/275
220074/2806/221/7306/2184/2805/4128/4282/4129
2571/4329/217/1892/84735
392/65985/38/3155/622
02/50
7165/443/2875/339983
38/50/132158/275
/217/1892/2639/36/38/1632
4577/217/1892/2639/23498/26/4128/55526/883/38/4129
07/2806/2805/2937/1491/4357/84245/3945/7263/883/55256
10449/219/18/549/259307/56922/5096/34/30/593/4329/217/1892/36/65985/38/3028/3155/3712
3/3945/38/197257/3029
06/2805/4843/217/26/384/84735/4128/112817/4129/112483/5625
/1353/4701/4696/4700/4715/1340/374291/6391/4713/4706/4705/4702/7386/4709/4695/4697/510
959/8694/51109/54884/157506/130/1551/10901/317749/54490/1576/1548/54905/145226/8608/15
28/54575/2938/218/222/130/1553/8574/4258/373156/2947/374875/54490/2052/2940/1576/221/1
/384/5313/5211/95/2875/3418/50/162417
7167/189/6391/8801/4191/84706/4524/2203/5091/113675/229/5232/3417/3421/1962/4967/5095/6
10497/535/245972/527/50617/6540/525/5864/6539/245973/155066
8/221/80201/217/55902/130589/84532/5313/3945/5211
30/54577/128/54575/2938/218/222/130/4258/373156/2947/54490/2940/1576/221/1548/2329/156
5568/54578/54658/54657/54577/760/54575/57835/3781/6522/6580/8671/6555/1244/476/358/107
54/283927/3417/55825/55670/196743/10901/1610/373156/51268/1962/11264/51179/2053/5830/
12/54576/7390/4351/54578/54658/54657/54577/54995/29968/204/54575/9249/8836/2990/2643/5
22/4726/3643/4701/4696/4700/4715/5296/1340/374291/5563/6391/4713/4706/5465/4705/4702/52

3/57521/111/6602/285521/126129/126328/63976/114/1327/6390/8110/56901/10818/4698/155/65
.7/7280/5438/54205/10383/25981/8408/7381/5331/246721/347733/4728/1347/6389/5602/5609/2
791/2335/10161/5728/5156/3082/3915/3560/3563/1311/285/1282/2790/3455/5594/3479/1278/91
908/5332/285/54518/5594/10235/3479/6237/2903/51466/57568/80310/22808/1268/55740/5216/7
05/5321/5533/5530/6263/108/4659/5743/1026/782/4660/2776/71/6261/3710/53632/5021/4882/7
2/7533/9021/91543/4599/355/7534/4792/10379/6774/7187/1965/1026/3458/6041/8554/4938/479
88/10459/1499/10163/60/63916/867/8218/10092/10094/387/26052/5879/998
93/472/1026/891/2810/841/8493/51246/896/1021/4193/143686/6477/8797
5592/113457/112714/5159/108/7278/5154/2776/3710/2771/2773/4893
3/5684/5710/5718/5686/5719/5691/9861/5688

/6338
31616/197322/51102/9524/1962/35/10449/9374/34/30/1892/27349/84869/36/7923/38
5/3352/5153/2784/773/2914/954/8645/6340/6337/22953/2911/6338
3284/8644/6820/1551/6716/374875/54490/1586/220074/1576/1585/79154/7923/3291
496/645745/4494/476/55630/55503/475/1181/7421/6569/65010/486/482/481/113235/340024/142
/54577/128/54575/2938/218/222/130/1551/1553/4258/373156/2947/374875/54490/2052/2940/15
5/11065/7318/7322/4591/55120/5371/10054/8554/55236/9039/672/140739/29945/26259/64750/1
313/1906/1003/3553/7412/6613/4205/3685/387082/3458/5154/1843/71/4790/2949/6885/5290/85
790/9641/51082/29110/3551/10623/10622/10621/8737/3569

424/4792/1910/108/7048/4790/4087/2769/5290/7046/399694/1284/2771/468/2773/4893/10000/5
/3685/7187/5743/1026/4790/3918/5290/1027/1284/3551/3688/3910

4/2555/56901/22999/4698/4717/3762/5568/2562/5331/776/4728/5602/2560/4722/6300/4726/470

40/10950/167227/54464/5393/29883/9337/196513/57819/51691/51013/9652/2027/25904/22803/1
.72/4176

38
38
3/56963/5933/4090/3399/4087/653/7046/9241/650/657/130399/64750/285704/6198/5515/4089/92
356/27330/5296/9894/64223/5170/5563/83667/5595/5291/84219/4041/2475/6009/6520/6199/929
3/7280/54205/10383/7317/7381/347733/91860/4728/1347/6389/5602/25828/7384/818/4722/598/9
116255/1056/224/223/219/57104/217/5407/56894/80339/132158/26007
88/487/89/1499/1674/60/1756/779/786/55799/6932
2937/3418/119391/26873/124975
160/55131/2091/10799/5901/28987/1736/54464/29102/84135/51119/4931/55781/51077/79631/1
990/3145/210/54490/124454/2235/326625
4577/54575/2990/1807/2938/4832/151531/4258/2947/83549/54490/2940/1576/8824/51733/1548/
4/1892

5/5156/4318/1643/3082/3915/3560/3563/5332/1282/2790/817/3455/5594/4313/3600/10235/3479/
5/317/23586/3717/6772/7752/3134/4615/9021/284406/355/162993/3678/339559/3553/3105/3107/
252/1326/4615/5530/7424/355/3553/5159/51135/5062/1848/7042/994/7043/6197/1849/5922/7048
6372/3576/6846/6373/3560/3563/6363/608/6351/3455/1236/920/3600/8807/939/6356/1524/1056
/6237/203068/103910/10096/4615/391/5216/3606/11035/3839/840/338382/29109/3836/3553/113
424/1292/3678/131873/5159/5062/3685/3694/5063/4659/7791/5154/7414/4660/71/7060/3918/369
58/4316/920/5594/3600/5925/890/9134/5533/3134/5530/9586/7538/996/5423/3105/3107/3117/4
67/3316/7042/3685/2535/7474/6774/7074/3091/27250/4659/1026/4660/2247/7078/71/8324/1655/
/3678/3553/4792/10095/7322/472/58484/7414/71/4790/3710/6885/7336/5290/3101/4671/7100/29
36/1839/11224/23586/3627/6136/6772/729/4615/6224/23521/4599/3553/717/6868/6188/6175/511
530/9586/11214/355/3553/3588/3105/3107/4792/3106/3135/3685/6774/108/6891/5734/5743/1026
05/3107/51135/5062/4792/3106/3135/200316/916/5063/6891/472/891/2776/85363/4790/3710/68
5/5925/890/9134/3665/9636/317/6502/23586/3627/6772/3134/4615/3516/355/3105/3107/51135/3
/3717/6772/64499/3838/4615/9021/91543/3606/4599/355/7177/3836/3553/51135/3117/4792/103
/2149/5159/5062/10095/3685/3694/85464/5063/7074/4659/5154/7414/4660/2247/71
06/840/355/3553/113457/112714/2149/6188/51135/4690/5062/4792/10095/5063/7278/71/4790/6
3455/5594/3665/6772/4615/3606/11035/90550/10010/3553/10135/51135/4792/10379/9051/10628
35/6774/7187/5922/5966/1026/4790/5933/7532/9519/5290/3718/1027/9759/841/468/4893/22938
606/6607/79023/10250/55706/25929/8667/9984/5411/1975/9818/8661/79760/7919/1967/7175/23
8494/920/29126/9076/3134/1003/257194/3105/3107/7412/83700/3117/3106/3135/3685/201633/3
43/6774/7187/7048/64135/1026/4790/9641/6885/5290/3718/7046/5111/841/468/4893/29110/3551
/3458/3566/5154/8554/3953/53342/5777/3597/9466/1441/5290/3718/8027/5617/338376/10401/3
5/6197/27040/7454/58484/2776/71/4790/6885/5290/7456/29110/3551/3688/10163/60/63916/100
3/64581/817/3122/3112/10312/3687/5594/317/3717/6772/5533/4615/5530/3606/3553/26253/3588
63/5332/653361/2790/6351/1236/5594/10235/6356/1524/10563/3627/3717/6772/6368/6355/4283
2214/3459/3106/3135/7535/27040/4277/4068/117157/3458/3823/5777/5290/399694
/6774/4325/27250/472/5743/1026/5154/648/7078/4790/3925/5290/672/1788/1027/7148/399694/9
95/4174/9126/9184/4172/991/4176/8379/4089/896/1021/4085/8900/4193/8881/64682/23594/109
653361/3133/3118/64581/84790/3122/3112/81035/10312/948/203068/3134/338382/3678/113457/
4790/3710/142/5290/4000/841/468/4893/4217/3551/332/1521/7132/1616/60/143/10000/9020
891/4790/5933/3710/4087/5290/7046/4893/4683/5534/23411
07/4792/10379/3459/3106/3135/6774/7187/131450/4277/3091/5743/1026/2247/4790/9641/3710/
/9984/58517/7919/10286/9416/9775/55110/10465/55696/1665/6427/57187/51340/27316/84991/2
46/3718/5837/29082/841
/5530/3726/3553/10990/2214/11006/4792/10379/3459/7042/9846/7048/3458/5971/4790/6885/52
379/6774/916/7187/1965/64135/51209/4938/4790/9641/6885/440275/5290/4940/3718/1027/1039

6891/3458/5993/3823
4/4086/5081/7048/472/5966/1026/25942/2005/5154/648/5371/1655/4790/2138/1027/51274/4314

355/3553/7412/4792/6364/7187/6374/5743/4790/6885/5290/4314/841/468/4217/3551/7132
/4615/3553/2214/51135/3117/4792/3459/7042/7043/3125/3127/5743/3458/4790/6885/5777
43/6774/3125/3127/4261/3458/4790/6885/3918/2771/841/2773/3551/3688/3910/7132/3593/5294
/841/29110/3551
6/3125/3127/27040/3594/4794/3458/3566

6368/8809/3606/6355/4283/3588/6358/6364/6374/6354
790/5777/5290/4893/5534/3551
3/55799/196883/5567/2778/7134/22801/3679/109

7/6445/71/6442/841/4624/941/4625/60/1756
66/4638/84876/2771/2244/2773/3688/5294/6786/60/695/5906/10000/7408/5501/387/1277/19688
92/695/10000/975
6192/6125/6134/6144/6146/6122/6228/6201/65005/6168/140801

4633/55799
/3125/3127

78/3553/951/3117/916/3125/3127/3566/2323

3600/3606/3553/3117/7042/6364/7043/3125/3127/6374/3458
6/3125/3127/27040/50615/3091/3594/7048/4794/3662/3458/3566/4790/4087/53342/9466/3718/7
3/5534/3551/10892/10000/9020/8440/387/1493/5603/998/5588/2534
7187/27040/472/5743/5971/4790/6885/142
187/4790/9641/6885/5290/841/7100/29110/3551/941/3593
/841/4893/5534/3551/10892/3593/1263/10000/9020/4193/387/3569/64170/5603
27/1284
8/2769/5290/1284
87/2769/5290/7046/2771/841/2773/3551/7132/3593

4790/4087

79/495/1537/4720/4704/10975/4718/9167/64077/9296/29796/4714/4694/1350/127124/529/2708
55/8228
548/1565/1555/873/22977/119391/27294

470/51179/35/48/2806/5096/80201/2805/4329/2731/55902/1892/84532/55753/414328/5313/521

5/1555/4128/119391/4129
7/54490/9376/2052/6256/123264/1576/8431/486/482/10864/200931/481/10998
5192/10005/30/4843/5264/26063/1384/3418/3155/4598/92960/5827
1109/3145/9104/112724/4832/3242/10327/250/8564/5372/125061/51004/4143/4337/210/587/90
291/7386/4709/4695/4697/51079/1537/4720/4704/10975/6256/4718/9167/29796/4714/4694/1350

260/4717/5568/7381/4728/1347/6389/1376/51103/7384/51287/4722/6300/4726/1353/3991/2629
878/7384/4722/160/4726/5439/3064/4701/548644/4696/4700/10540/5441/6647/4715/161/1340/3
34/374/3371/80310/3717/7533/9586/7424/1292/4170/3678/9180/131873/2149/4602/5159/7534/
7424/2149/5159/9771/27040/108/7074/51735/5154/2776/2247/71/5900/5898/2846/5290/25780/2
84/4638/2771/2773/4893/5534/140465/5294/60/5565/779/10398/786/5501/55799/387/196883/55
0/7532/9641/440275/5290/4940/841/4893/29110/3551/7132/1499/7529/10000/5515/975/1021/5

680
76/221/1548/873/883/119391
0401/9320/867/991/51366/4734/4193/4214/8881/26272/7324/64682/6477/9616/11059/7316
9/657/4217/3551/7132/1499/60/10000/7184/92/387/6612/9181/5603/5879

9/408/1277/196883/5567/2778/5603/59345/1385/4312/64764

1/5578/4696/4700/222236/4715/374291/5595/4713/4706/2915/2784/4705/3760/4702/773/4709/

70506/22894/219988/23404/87178/5214/80349/5213/255967

2/4093/387
6/10542/8322/79109/2887/79726/127124/529/5582/220441/525/55615/7477/55437/7479/245973
9927/4726/4701/4696/4700/4715/1340/374291/5715/6391/4713/4706/842/4705/4702/7386/4709/

0885/9724/23195/22803/7514/10556/51096/10557/10482

7172/119391

/5925/890/9134/6789/1906/317/6502/3717/6772/4853/7185/9252/7424/840/355/8453/2149/5159/
/51135/5199/3117/4792/10379/3459/3106/162966/3135/219749/390980/7187/79230/3125/3127/1
3/2005/5154/782/2323/2247/1843/5971/4790/6885/3925/25780/7046/8817/784/5921/11184/468/4
3/3627/6368/8809/3606/6355/355/9180/4283/3553/9235/94/3588/58191/6358/23765/3459/7042/
3457/112714/3831/6188/51135/57381/4792/10095/3840/5063/7278/58484/51209/71/4790/6885/5
96/5290/7148/399694/1284/859/4638/3688/3910/1499/29895/60/5906/10000/10398/7408/4633/5
792/3106/7042/3135/7043/4488/916/3125/3127/108/7048/2113/472/1026/2005/706/5971/4790/4
/3710/4087/7483/5777/5290/286/859/11211/4893/3688/3593/6194/1499/7472/60/29102/6198/867
9110/11146/3551/3688/7132/10892/22863/10163/29895/60/9265/6198/63916/3098/10000/10398/
135/6142/4792/10379/25873/6193/6774/7187/6159/6229/2197/64135/6189/6135/6187/6130/6203
5/2776/4790/3710/5290/5732/2771/841/468/2773/4893/29110/5534/3551/1230/7132/1499/3570/6
385/5290/2771/841/2773/4893/29110/5534/3551/7132
3117/4792/10379/3106/3135/6774/916/7187/3125/3127/8819/6891/4794/1026/25942/5971/4938/
79/3459/7187/3125/3127/4261/1965/64135/3458/6041/5371/29107/71/4938/4790/9641

6885/2846/5777/4671/841/7100/7456/3551/3688/7132/10006/10163/60/9265/7082/10802/4430/10
3/7187/58484/6041/22900/4938/4790/9641/3710/6885/115361/4940/5027/140609/4671/841/2911
3/7529/3572/991/8379/896/1021/8900/5610/5315/4193/387/5567/5700/5879/1385/10971/998/647
3435/9775/55110/6612/9086/55746/9972/57187/51808/8891/96764/9688/7514/8087/10556/1917/
3125/3127
1/332
593/3570/3976/3572/10000
00/10092/10094/387/3569/5603/5879/998
3/2214/51135/3117/3459/7042/7043/4046/3125/3127/4261
3/58191/6358/4792/6364/6774/7454/108/7074/6374/6354/2776/4790/1235/5290/3718/399694/277

9759/27086/4893/3551/23411
71/1871/4171/545/1031/7157/983/2033/2932/5934
/112714/3105/3107/2214/3117/3106/3135/3685/3125/3127/6891/7278/4481/71/8411/7060

/5290/841/4893/29110/5534/3551/1230/7132/22863/1499/5294/3572/10000/1021/64422/5610/69
29896/55660/3190/11325/6628/84950/6636/6426/25949/6428/10772/988/6432/51729/6434/10450

290/7046/2355/126014/5534/3551/10326/7132/8600/695/10000/9020
99/841/29110/3551

4

3/2243/5567/2778/5603

7046/5534/3556/3551

9/535/245972/1329/527/50617/4723/525/1337/9377/1349/245973/155066/1351

1/2875/3418/38/8802/50/132158/26007/275

54/54490/6470/224/124454/219/205/2356/248/145226/2937/249/217/53354/2235/23498/23475/1
0/27089/6720/1329/5313/2931/4723/1337/51085/9377/1349/1351

1/4701/6195/4696/4700/4715/27330/1340/374291/64223/5606/5563/6391/4713/4706/353500/470
74291/146754/5715/6391/4713/4706/9001/842/2915/4705/148327/4702/7386/2880/4709/4695/4
3115/3685/3694/1026/3566/5154/2323/2247/4790/7532/7060/2846/3918/3696/1441/5290/672/37
3683/8817/2771/2773/4893/3688
67/2778
10

4695/4697/51079/107/4720/4704/4718/747/4714/4694/57406/5582/4723/2911/2166/2788

4695/4697/51079/1813/10131/1537/147700/65018/4720/4704/10975/4718/9167/79861/29796/47

/4792/3459/7042/3685/7043/2535/1910/7474/6774/7187/108/1909/3091/3594/7048/2113/5734/5
1965/10780/6891/10793/64135/94039/3458/641339/6041/148156/5371/7773/10189/84671/4938/5
4893/5534/4217/3556/3551/7132/1616
6364/7043/85480/3557/50615/3594/7048/6374/6354/8771/3458/3566/3953/944/53342/3597/123
898/5290/388/55770/4671/841/7100/3551/3799/7132/10006/1499/79443/29895/5294/60/9265/86
501/896
087/9519/5290/701/3718/7046/4487/468/5901/4893/29945/5534/2114/3551/7132/4801/10000/91
7/10000
/23048/4633/10092/4193/11335/10094/387/8737/9181/5603/5879/998/7316/5588
/6154/6166/51187/4938/4790/9641/6885/6138/5290/4940/2266/6191/6123/4314/6124/6230/224
198/10000/1021/4193/387/196883/5567/8737/3569/2778/5603/59345/5879/1385/1871/64764

/4790/9541/9641/6885/5290/4940/3718/1027/841/29110/3551/22938/965/695/10000/9020/5706/

0092/10972/10094/8440/387/8737/10427/9181/3569/5603/8797/5879/998/2534/1365/8743/4691/
0/3551
/64/2961
/11218/1983/10557/1977/10482/8666/51068/5903/10460/8563/10289/65109/23191/1968

/1/5197/2773/4893/3551/1230

32/57580/3569/5603/59345/5879/1385/7316/1871
/6430/51639/9879/6635/2521/9343/23450/84844/3312/6431/9716/51503

.723/8566/349565/554235/5313/326625/57026/55163/93100/6697/80347

)5/148327/4702/51548/7386/4709/4695/4697/51079/493753/51241/107/2475/6009/25915/123096
597/51079/5432/4294/1387/2475/1537/27019/2902/147700/4720/4704/10975/5435/4718/9167/27
18/1027/7148/1284/8817/5617/118788/468/4893/3551/3688/3910/6194/3570/5294/7529/6198/1C

14/4137/4694/1350/27089/810/1329/292/4128/4723/1337/4129/9377/1349/118424/1351

743/1026/3458/3566/5154/2323/5371/2776/2247/26508/8324/4790/2949/5900/4087/7483/5898/2
1427/7633/4790/9641/6885/284307/7574/90321/440275/163227/29915/5290/282890/4940/12601

/9466/1441/653/7046/5617/650/338376/3604/5197/657/130399/3556/1230/7132/3593/4050/357
55/63916/1781/10000/10398/7184/4633/10092/5287/6932/10640/64746/10094/387/51143/11257

84/9020/991/8379/4089/896/4085/8900/4214/196883/8881/5567/3569/64682/1385/1871/64764

/29110/3551/6143/7132/3593/6194/3570/730/6192/6125/6134

734/896/1021/8900/5707/5610/4193/930/8737/3569/5700/10213/5603/5879

267/2768

/1537/4720/4704/10975/4718/9167/6199/57104/29796/4714/55811/4694/1350/4878/27089/1329/
'113/79861/29796/4714/10126/1742/4694/1350/5437/27089/1329/10891/292/4723/10120/1337/9[illegible]
[illegible]000/5515/4254/7184/1975/896/1021/4193/930/1277/3914/5529/3569/5649/59345/5879/1385/10[illegible]

846/3918/3597/1441/8202/5290/3718/25780/7046/1027/5732/1284/8817/11211/650/7170/2771/8
.7/65243/389114/80110/57209/9831/7559/84527/841/146198/7767/7673/29110/84924/3551/2615

0/8600/3976
4/8737/3569/2316/1783/5603/8797/9648/5879/998/51164/8743/10121

/10891/84987/4723/1337/9377/1349/1351
377/1349/3766/1351
971/2249/64764

[illegible]41/4436/2773/4893/2736/3551/3688/332/3910/1488/3593/1499/7472/3570/6198/867/3572/1000[illegible]
[illegible]2/7594/90317/7132/57547/1616/3593/115509/81931/342909/162655/10172/90649/163081/1000[illegible]

[illegible]/185/4254/7184/405/4089/896/1021/8900/6932/367/5979/4193/5888/387/7175/3914/196883/55[illegible]
[illegible]/7695/58500/7571/147929/349075/7562/5501/345462/30832/140612/147923/10224/91661/7596/

[illegible]7/3595/3569/4292/2778/59345/5879/4312/998/2249/1871/11040/7482/7296
[illegible]5610/90075/1967/163071/57693/54811/57711/7770/55769/91975/117608/3569/64170/55659/774

[illegible]8/6427/7639/7567/22835/8891/199704/7637/10520/6940/79788/55422/84911/7733/148266/8487

4/7699/7771/329/57232/147694/148103/7157/121274/91120/79862/93134/27102/684/342926/25[illegible]

888/6426/3111/5970/7539/10482/163087/147660/7743/55786/58492/6428/7738/90987/147948/7[illegible]

9088/3690/3716/3454/57677/125893/6432/6773/6850/7098/57573/148268/7553/162963
